# Supplementary material for: Transcriptomic Evaluation of Pulmonary Fibrosis-Related Genes: Utilization of Transgenic Mice with Modifying p38 Signal in the Lungs
Source: Int J Mol Sci. 2020 Sep 14;21(18):6746. doi: 10.3390/ijms21186746 (PMC7555042; doi:10.3390/ijms21186746)
Supplement: Supplementary file 1 [file ijms-21-06746-s001.zip › Supplementary Figures S1 and S2.pdf]

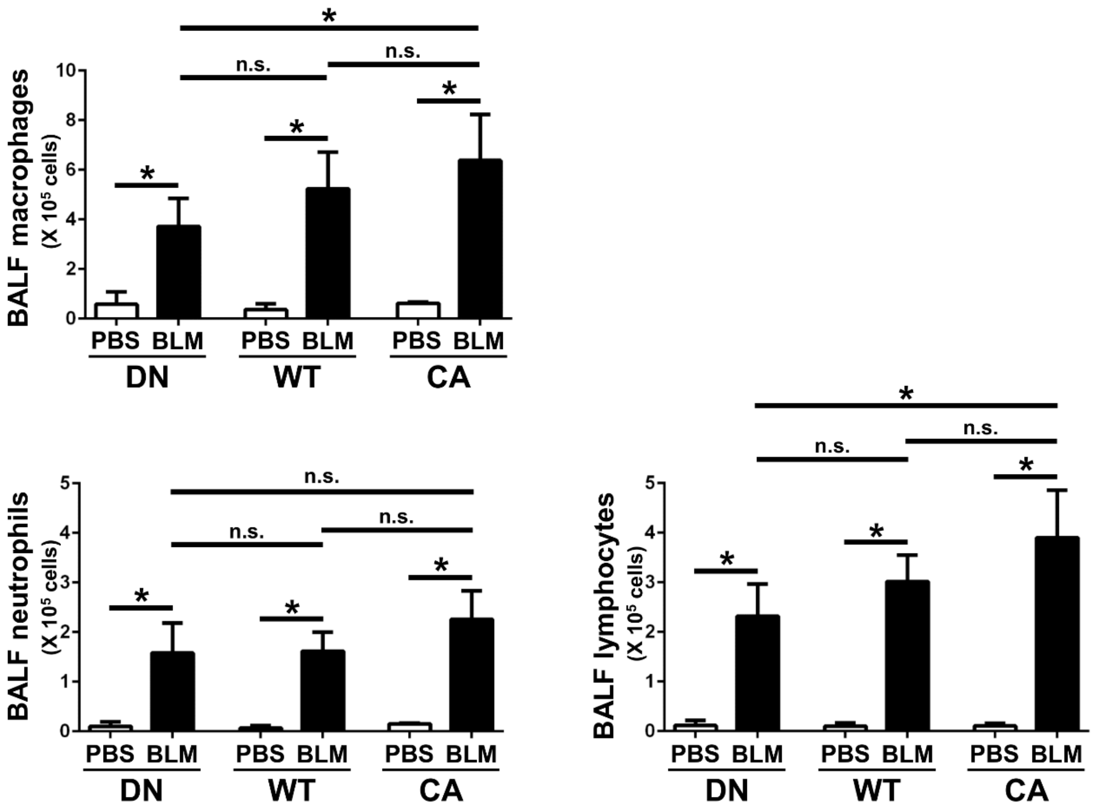

Supplementary Figure S1. Differential cell counts in BALF. The numbers of macrophages, lymphocytes, and neutrophils in BALF at 8 dpi of BLM and PBS were compared among three mouse groups. Data are represented as means ± SEM (n = 5). $*p < 0.05$, n.s., no significant difference (measured by one-way ANOVA followed by Tukey's test or unpaired Student's t-test).

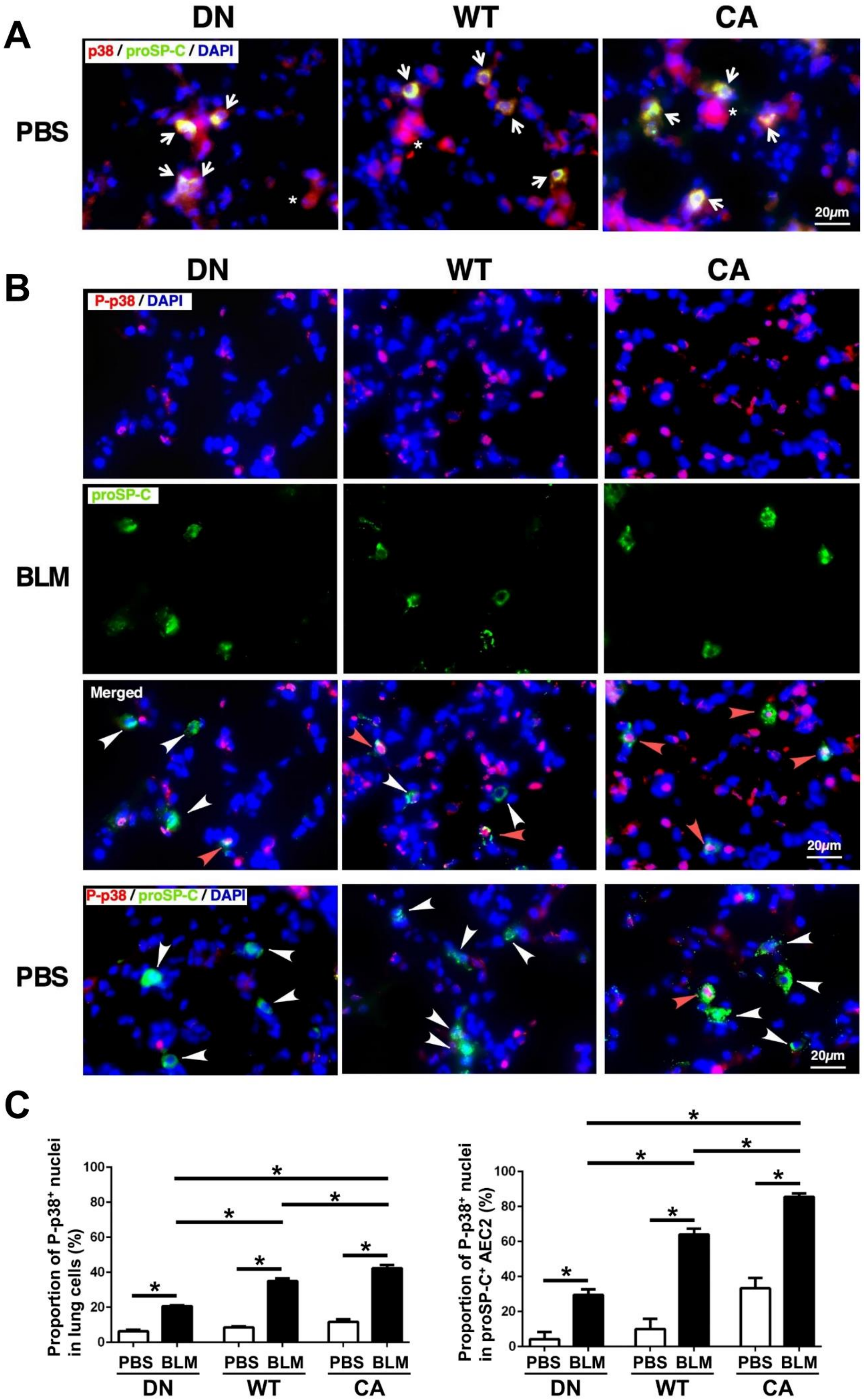
A
DN
WT
CA
p38 / proSP-C / DAPI
PBS
20μm
B
P-p38 / DAPI
proSP-C
Merged
BLM
P-p38 / proSP-C / DAPI
PBS
C
Proportion of P-p38⁺ nuclei in lung cells (%)
Proportion of P-p38⁺ nuclei in proSP-C⁺ AEC2 (%)
PBS BLM
DN
WT
CA

Supplementary Figure S2. The expression and activity of p38 in the lungs with different genotypes. The three mouse genotype groups; DN, WT and CA, were intratracheally administrated PBS and BLM. (**A**) The lung sections of the PBS-treated groups at 8 dpi were subjected to immunofluorescence staining for p38 (red) and prosurfactant protein-C (proSP-C, green). DAPI (blue) stains nuclei. Arrows indicate $p38^{+}$ proSP-C^{+} cells. Asterisks indicate alveolar macrophages. Among the three mouse genotypes, the lung distribution of p38 expression was almost unchanged in alveolar epithelial type II cells (AEC II), macrophages, and some other parenchymal cells. (**B**) The lung sections of the BLM- and PBS-treated groups at 8 dpi were subjected to immunofluorescence staining for phospho-p38 (P-p38, red) and proSP-C (green). DAPI (blue) stains nuclei. Arrow heads indicate SP-C^{+} cells. In particular, red arrow heads indicate SP-C^{+} cells with P-$p38^{+}$ nuclei, revealing p38 activation in AEC II. Few AEC II showed P-$p38^{+}$ nuclei in the PBS-treated DN and WT groups, while modestly increased number of them were observed in the PBS-treated CA group. Additionally, the number of AEC II with P-$p38^{+}$ nuclei was increased in the BLM-treated groups as compared with the PBS groups in all genotypes. At least in AEC II, BLM-induced stepwise activation of p38 was dependent on the genetically modified intrinsic p38 activity. (**C**) Proportion of nuclear localization of P-p38 in lung cells and AEC II. To quantify BLM-induced phosphorylation/nuclear translocation of p38, the ratio of P-p38-positive nuclei to total DAPI-positive nuclei, and proSP-C-positive cells with P-p38-positive nuclei to all proSP-C-positive cells were evaluated in 4 random fields ($1.7 mm^2$ each per field) under fluorescence microscopy (400× magnification). Data are represented as means ± SEM. *$p < 0.05$, n.s., no significant difference (measured by one-way ANOVA followed by Tukey's test or unpaired Student's t-test).
