# Supplementary material for: Transcriptomic Evaluation of Pulmonary Fibrosis-Related Genes: Utilization of Transgenic Mice with Modifying p38 Signal in the Lungs
Source: Int J Mol Sci. 2020 Sep 14;21(18):6746. doi: 10.3390/ijms21186746 (PMC7555042; doi:10.3390/ijms21186746)
Supplement: Supplementary file 1 [file ijms-21-06746-s001.zip › Supplementary Table S1.pdf]

Supplementary Table S1. BLM-induced cytokine molecules in BALF that were correlated with intrinsic p38 activity in the lungs.

**Pro-fibrotic cytokines**	Induction Index (Arbitary unit)		
Name	DN	WT	CA
**IL-13**	5.2	6.5	15.0
**IL-17**	8.5	32.2	43.5
**SDF-1/CXCL12**	2.9	5.4	7.2

**Pro-inflammatory mediators**	Induction Index (Arbitary unit)		
Name	DN	WT	CA
CCL1/TCA-3	2.5	5.8	6.8
Eotaxin/CCL11	4.0	39.0	62.0
**IFN-γ**	6.7	8.6	14.2
IL-15	3.5	6.0	14.0
**KC/CXCL1**	5.8	7.6	11.7
LIX/CXCL5	2.2	2.7	4.3
**MIG/CXCL9**	2.9	5.0	11.9
**MIP-1α/CCL3**	17.6	29.0	33.6
MIP-2/CXCL2	3.5	5.2	6.4
**RANTES/CCL5**	37.4	49.0	65.7
Thymus chemokine-1/CXCL7	1.4	2.8	5.4

**Fibrosis-related proteins**	Induction Index (Arbitary unit)		
Name	DN	WT	CA
Activin A	1.3	2.4	4.2
CXCL14	3.1	10.4	11.6
IGFBP-2	4.3	5.8	8.3
IGFBP-3	4.2	5.6	8.1
IGFBP-5	2.3	26.6	64.1
IL-22	11.0	26.0	49.0
**MMP-2**	3.7	6.5	9.4
**MMP-3**	15.0	41.0	88.2
**MMP-9**	4.0	17.0	93.7

**Others**	Induction Index (Arbitary unit)		
Name	Ind_DN	Ind_WT	Ind_CA
Cardiotrophin-1	7.5	30.0	42.5
**CCL28**	6.1	13.3	16.4
CCR9	2.7	3.5	5.5
CD11b	3.2	4.1	6.4
CD27 Ligand	3.5	6.7	8.6
IL-2R γ	9.7	17.3	23.1
CTACK	3.5	9.3	10.4
Dtk	2.5	3.9	5.3
EGF R	11.3	30.6	39.7
EG-VEGF	10.7	14.0	20.7
Endogrin/CD105	4.2	18.8	27.2
Eotaxin-2	10.2	31.6	34.3
FGF R3	6.7	8.6	15.2
Follistatin	1.2	3.8	8.1
Frizzled-1	1.2	4.9	8.5

GDF-5	28.5	47.5	61.0
GDF-8	27.8	46.7	65.9
GDF-9	9.0	33.0	42.0
GDNF Rα-2	1.7	24.0	58.0
IFN-γ R1	3.4	4.3	6.7
IGFBP-1	3.6	5.0	8.0
IGFBP-6	5.0	29.0	43.5
IL-1 RII	4.4	7.4	10.2
IL-3 Rα	8.3	86.4	113.6
IL-15 Rα	2.3	3.9	6.0
IL-16	4.6	7.5	10.1
IL-23 R	2.1	2.8	3.9
Kremen-1	4.9	17.4	28.3
Lungkine/CXCL15	3.2	4.0	5.8
Lymphotoxin βR	3.8	4.9	7.3
MAdCAM-1	5.7	18.7	25.7
MDC/CCL22	14.5	19.5	43.5
Osteoactivin	2.4	3.3	5.9
Osteoporotegerin	29.0	44.0	81.5
PDGF C	4.7	9.5	13.0
PDGF Rα	4.4	6.3	8.2
PIGF 2	5.0	14.3	22.7
Prolactin	2.3	3.2	4.6
P-selectin	2.2	2.7	4.1
Resistin	13.4	20.6	29.6
SIGIRR	3.5	24.5	32.3
Soggy-1	1.3	3.5	4.8
Spinesin Ectodomain	1.6	2.0	4.5
TGF-β3	6.0	22.1	35.6
TGF-β RII	2.5	3.5	4.7
TIMP-2	4.1	6.7	7.8
TIMP-4	24.1	44.1	52.7
TLiA/TNFSF15	6.7	16.0	31.0
TNF RI	1.9	6.8	13.1
TNF-β	1.5	3.6	5.7
TRANCE/TNFSF11	8.8	19.0	36.0
TWEAK	30.0	62.0	70.0
TWEAK R	2.9	4.0	4.9
WIF-1	15.3	34.7	57.3
