# Supplementary material for: Transcriptomic Evaluation of Pulmonary Fibrosis-Related Genes: Utilization of Transgenic Mice with Modifying p38 Signal in the Lungs
Source: Int J Mol Sci. 2020 Sep 14;21(18):6746. doi: 10.3390/ijms21186746 (PMC7555042; doi:10.3390/ijms21186746)
Supplement: Supplementary file 1 [file ijms-21-06746-s001.zip › Supplementary Table S5.pdf]

Supplemenary Table S5. The gene symbol list of 493 common DEGs upregulated by BLM among MKK6-CA, WT, and p38-DN groups.

A2m	Ccl22	Clec5a	Ereg	Gyg
Abcb1b	Ccl6	Clec7a	Esco2	H2-Eb2
Ackr1	Ccl7	Cmah	Espl1	Has2
Ackr3	Ccl9	Cmtm3	Etv4	Havcr2
Adam12	Ccna2	Col12a1	F10	Hbegf
Adamts12	Ccnb2	Col28a1	F7	Hcn1
Adamts4	Ccne1	Col3a1	Fabp5	Hells
Adgre1	Ccnf	Col4a1	Fam105a	Hexb
AI506816	Ccng1	Col5a2	Fam20c	Hist1h1a
Ano5	Ccr1	Col5a3	Fam49b	Hist1h1b
Anxa1	Ccr2	Col8a1	Fbn1	Hist1h2ab
Aoah	Ccr5	Coro1a	Fcer1g	Hist1h2ak
Apbb1ip	Cd180	Cotl1	Fcgr1	Hist1h2bk
Apobec1	Cd200r1	Cpxm1	Fcgr2b	Hist1h2bm
Apoe	Cd200r4	Csf2rb	Fcgr3	Hist1h3d
Arg1	Cd248	Csf2rb2	Fcrls	Hist1h4f
Arhgdib	Cd276	Csmd1	Fermt3	Hpgds
Armcx4	Cd300a	Cstb	Fgr	Hsd17b7
Arpc1b	Cd300c2	Cthrc1	Fhl3	Hvcn1
Aspm	Cd300lf	Ctps	Fignl1	I830077J02Rik
Atg9b	Cd38	Ctsb	Fkbp10	Icam1
Atp1a3	Cd48	Ctsc	Fn1	Icos
Atp6v0c	Cd52	Ctsd	Foxm1	Ier3
Atp6v0d2	Cd53	Ctss	Frzb	Ifi204
B2m	Cd68	Ctsz	Fscn1	Ifi207
B3gnt5	Cd84	Cxcl10	Fst	Ifi209
B430306N03Rik	Cd86	Cxcl13	Fstl1	Ifi27l2a
Bard1	Cdc20	Cxcl15	Fzd1	Ifi30
Bcl2a1b	Cdca3	Cxcl16	Gas2l3	Ifit2
Bgn	Cdh11	Cxcl9	Gcnt4	Ifitm3
Birc5	Cdk1	Cybb	Gda	Igf1
Bmper	Cdkn1a	Cyp4f18	Gdf15	Igfbp2
Brinp1	Cemip	Cyp7b1	Gimap9	Igha
Bst1	Cenpf	Dab2	Gja1	Ighg2b
Btk	Cenpl	Dck	Gla	Ighv1-53
Btla	Cers6	Ddias	Glipr2	Ighv1-64
Bub1	Cfp	Dhfr	Glrx	Igkv10-96
C1qa	Ch25h	Diaph3	Gm10718	Igkv1-110
C1qb	Chl1	Disp2	Gm14548	Igkv1-117
C1qc	Chodl	Dlgap5	Gm6377	Igkv8-30
C1qtnf6	Chrna7	Ear2	Gnpda1	Iglc2
C330027C09Rik	Chsy3	Eda2r	Gpnmb	Igsf6
C3ar1	Ckap2	Egr2	Gpr141	Il1rn
C430002N11Rik	Ckap4	Eln	Gpr176	Il21r
C5ar1	Clca3a1	Emcn	Gpr35	Il2rg
Capg	Cldn4	Enc1	Gpr65	Il4ra
Capn6	Clec4a2	Eno1	Grn	Ildr2
Casp3	Clec4a3	Enpp1	Grrp1	Irf5
Ccdc80	Clec4e	Epha3	Gtse1	Isg15
Ccl17	Clec4n	Ercc6l	Gusb	Itga5

Itgam_1
Itgax
Itgb2
Itgb6
Itm2a
Kcnk13
Kctd12b
Kif11
Kif15
Kif22
Kif4
Knl1
Kntc1
Kynu
Lair1
Lama1
Lamb3
Lamc2
Laptm5
Lcn2
Lgals3
Lgals9
Lgmn
Lhfpl2
Lhx6
Lilrb4a
Lipa
Litaf
Lox
Loxl2
Lpcat2
Lpl
Lpxn
Lrg1
Lrp12
Lrp2
Lrrc25
Lrrc8c
Ltbp2
Ly6a
Ly6c1
Ly6c2
Ly86
Lyz2
Malt1
Map2
March1
Marcksl1
Mcemp1
Mcm5
Mdga1
Megf10
Mest
Mgp
Mki67
Mmp12
Mmp14
Mmp19
Mmp8
Mpeg1
Ms4a1
Ms4a6b
Ms4a6c
Ms4a6d
Ms4a7
Msr1
Mt2
Mthfd2
Mybl1
Myc
Myo5a
Naip2
Napsa
Ncapg
Ncapg2
Ncf2
Nckap1l
Ndc1
Ndnf
Nek6
Nfam1
Nme1
Nme2
Nos2
Nrcam
Nrep
Nt5e
Ntrk2
Nuf2
Nusap1
Nxpe4
Nxpe5
Oas3
Olfm1
Olr1
Osm
P2ry6
P4ha3
Pappa2
Pdgfc
Pdpn
Pgf
Phex
Phlda3
Pik3cg
Pilra
Pim1
Pirb
Pla1a
Plat
Plau
Plbd1
Pld3
Pld4
Plek
Plxnc1
Pole
Pou2af1
Ppa1
Prc1
Prcp
Prkcb
Prr11
Prrx2
Prss22
Psap
Psat1
Psmb8
Psrc1
Ptafr
Ptger4
Ptgir
Ptprc
Rab32
Rab3il1
Rab8b
Rac2
Racgap1
Rap2b
Rcan1
Rgs16
Rhoh
Rpl39
Rrm2
Rsad2
Rtn4rl2
S100a4
S100a6
Saa3
Samsn1
Sash3
Scarb1
Scn3b
Scube1
Sdcbp
Sdk2
Selp
Selplg
Serpina3g
Serpina3n
Serpinb6b
Serpinb9
Serpine1
Serpine2
Sfrp1
Sgk1
Sgpl1
Sh2b2
Sh2d1b1
Sh3pxd2b
Shc4
Shcbp1
Siglec1
Sirpa
Sirpb1c
Slamf9
Slc11a1
Slc15a3
Slc27a6
Slc29a3
Slc35f6
Slc37a2
Slc39a14
Slc7a8
Slfn1
Slfn10-ps
Slfn2
Slfn8
Slfn9
Sparc
Spc25
Spi1
Spp1
Sprr1a
Spry4
St8sia4
Stra6l
Svop
Syt12
Tfec
Tgfb1
Tgfbi
Tgm1
Thbs1
Thbs4
Tifab
Timp1
Tlr1
Tlr13
Tlr2
Tlr7
Tlr8
Tmem106a
Tmem173
Tmem26
Tmem37
Tmem86a
Tmsb10
Tnc
Tnfaip8l2
Tnfrsf11b
Tnfrsf12a
Tnfrsf13b
Tnfrsf1b
Tnfsf8
Tnip3
Top2a
Tpx2
Trem2
Trim59
Tspan8
Ttyh2
Tyrobp
Ubash3b
Ugcg
Ulbp1
Vcan
Vstm4
Wfdc17
Wisp1
Wnt10a
Wnt7a
Zfp385a
