# Supplementary material for: Transcriptomic Evaluation of Pulmonary Fibrosis-Related Genes: Utilization of Transgenic Mice with Modifying p38 Signal in the Lungs
Source: Int J Mol Sci. 2020 Sep 14;21(18):6746. doi: 10.3390/ijms21186746 (PMC7555042; doi:10.3390/ijms21186746)
Supplement: Supplementary file 1 [file ijms-21-06746-s001.zip › Supplementary Table S6.pdf]

Supplemantary Table S6. Transformed expression values in 2,722 genes of the cluster that BLM-induced gene expression was correlated with intrinsic p38 activity in the lungs.

Name (symbol)	DN	WT	CN
0610007P14Rik	-0.603	-0.181	0.784
0610010K14Rik	-0.539	-0.372	0.911
1110008F13Rik	-0.651	-0.168	0.818
1110012L19Rik	-0.673	-0.316	0.989
1110038B12Rik	-0.730	0.076	0.654
1500009L16Rik	-0.812	0.001	0.810
1500011K16Rik	-0.463	-0.332	0.794
1600014C10Rik	-0.598	-0.185	0.783
1700017B05Rik	-0.717	-0.075	0.792
1700027J19Rik	-0.642	-0.151	0.792
1700056N10Rik	-0.864	0.138	0.726
1700086O06Rik	-0.452	-0.345	0.797
1700123O20Rik	-0.724	-0.258	0.982
2210013O21Rik	-0.600	-0.102	0.702
2310009A05Rik	-0.677	-0.203	0.880
2310057M21Rik	-0.604	-0.412	1.015
2410006H16Rik	-0.712	-0.136	0.848
2510002D24Rik	-0.523	-0.367	0.890
2610001J05Rik	-0.770	-0.164	0.934
2610002M06Rik	-0.472	-0.334	0.805
2610021A01Rik	-0.665	-0.063	0.728
2610301B20Rik	-0.507	-0.245	0.751
2810002D19Rik	-0.905	0.128	0.777
2810004N23Rik	-0.600	-0.124	0.724
2810428I15Rik	-0.612	-0.307	0.919
3300002I08Rik	-0.522	-0.319	0.841
3425401B19Rik	-0.626	-0.290	0.916
3830406C13Rik	-0.863	0.051	0.812
4833439L19Rik	-0.580	-0.175	0.755
4921513I03Rik	-0.747	0.040	0.707
4930430E12Rik	-0.960	0.147	0.814
4930453N24Rik	-0.714	-0.034	0.748
4930539J05Rik	-0.844	0.100	0.744
4931406P16Rik	-0.585	-0.147	0.732
4933421O10Rik	-0.599	-0.098	0.696
5530601H04Rik	-0.711	0.052	0.659
5730405O15Rik	-0.480	-0.304	0.784
6030408B16Rik	-0.706	-0.197	0.902
6030445D17Rik	-0.619	-0.109	0.729
6430548M08Rik	-0.556	-0.222	0.778
6530409C15Rik	-0.716	0.056	0.660
8430419K02Rik	-0.505	-0.431	0.936
9230110C19Rik	-0.909	0.076	0.833
9330102E08Rik	-0.501	-0.306	0.807
9330182L06Rik	-0.680	-0.205	0.885
9430016H08Rik	-0.633	-0.245	0.879
9430020K01Rik	-0.585	-0.209	0.794
A130010J15Rik	-0.621	-0.245	0.867
A430005L14Rik	-0.733	-0.203	0.936
A4galt	-0.744	-0.157	0.901

A730020M07Rik	-0.556	-0.287	0.844
A930011G23Rik	-0.579	-0.169	0.748
Aamdc	-0.528	-0.395	0.924
Aars	-0.546	-0.249	0.795
Aarsd1	-0.603	-0.085	0.688
Aasdhppt	-0.831	0.033	0.798
Abca6	-0.459	-0.345	0.804
Abca8a	-0.595	-0.326	0.921
Abca8b	-0.703	-0.274	0.976
Abcb1a	-0.630	-0.328	0.958
Abcb1b	-0.564	-0.175	0.739
Abcb7	-0.769	-0.130	0.899
Abcd2	-0.719	-0.237	0.956
Abcd3	-0.529	-0.320	0.849
Abcf2	-0.569	-0.145	0.715
Abhd13	-0.645	-0.151	0.797
Abhd17c	-0.594	-0.130	0.724
Abhd4	-0.607	-0.190	0.797
Abhd5	-0.588	-0.307	0.895
Abhd6	-0.538	-0.235	0.773
Abi1	-0.786	-0.170	0.956
Abi2	-0.740	-0.141	0.881
Abracl	-0.664	-0.106	0.770
Acaa2	-0.535	-0.381	0.915
Acaca	-0.516	-0.264	0.780
Acadl	-0.752	-0.197	0.950
Acadvl	-0.610	-0.107	0.717
Acap2	-0.597	-0.165	0.762
Acbd6	-0.714	-0.119	0.833
Acer2	-0.720	-0.011	0.731
Acer3	-0.881	-0.014	0.895
Ackr2	-0.878	-0.007	0.885
Acly	-0.637	-0.202	0.839
Aco2	-0.548	-0.257	0.806
Acot11	-0.525	-0.198	0.723
Acot13	-0.530	-0.289	0.819
Acot2	-0.483	-0.271	0.754
Acp2	-0.681	-0.073	0.754
Acsl4	-0.690	-0.254	0.943
Acta2	-0.534	-0.348	0.882
Actg1	-0.750	-0.159	0.909
Actl6a	-0.647	-0.259	0.906
Actn1	-0.712	-0.141	0.854
Actn4	-0.740	-0.226	0.966
Actr2	-0.748	-0.172	0.920
Actr3	-0.611	-0.073	0.683
Actr6	-0.737	0.044	0.693
Acvr2a	-0.644	-0.056	0.700
Acyp1	-0.854	-0.035	0.890
Acyp2	-0.733	-0.117	0.850
Adam10	-0.800	-0.100	0.900
Adam12	-0.702	0.030	0.672
Adam15	-0.744	-0.023	0.767
Adam17	-0.639	-0.181	0.819
Adam9	-0.641	-0.157	0.798
Adamts15	-0.831	-0.109	0.939

Adamts9	-0.760	0.002	0.759
Add3	-0.572	-0.236	0.807
Adgrd1	-0.683	-0.302	0.986
Adgrg6	-0.605	-0.080	0.685
Adh1	-0.483	-0.322	0.805
Adhfe1	-0.536	-0.183	0.719
Adipor2	-0.686	-0.109	0.795
Adk	-0.616	-0.334	0.950
Adprh	-0.494	-0.249	0.743
Adprm	-0.681	-0.271	0.952
Adra2b	-0.784	-0.118	0.902
Adrb2	-0.693	-0.116	0.808
Adrm1	-0.594	-0.119	0.714
Aes	-0.703	-0.206	0.909
Aff1	-0.637	-0.133	0.769
Aff2	-1.069	0.223	0.846
Aff3	-0.582	-0.208	0.790
Aff4	-0.744	-0.155	0.899
Afmid	-0.947	0.183	0.764
Aga	-0.614	-0.196	0.810
Agfg1	-0.633	-0.149	0.781
Agpat4	-0.752	-0.149	0.901
Agpat5	-0.678	-0.107	0.785
Agpat9	-0.785	-0.061	0.846
Agps	-0.662	-0.106	0.768
Ahcy	-0.686	-0.310	0.996
Ahcyl1	-0.571	-0.195	0.766
Ahsa1	-0.609	-0.137	0.746
AI413582	-0.595	-0.254	0.849
AI504432	-0.556	-0.417	0.973
AI597479	-0.485	-0.273	0.758
AI661453	-0.455	-0.325	0.779
AI837181	-0.660	-0.248	0.907
AI987944	-0.658	-0.156	0.814
Aida	-0.695	-0.162	0.857
Aip	-0.471	-0.337	0.808
Ak3	-0.520	-0.231	0.750
Ak6	-0.693	-0.301	0.994
Akap11	-0.572	-0.134	0.706
Akirin2	-0.611	-0.070	0.681
Akr1b3	-0.582	-0.300	0.882
Akr1e1	-0.545	-0.210	0.754
Alas2	-0.598	-0.352	0.950
Alcam	-0.519	-0.215	0.734
Aldh18a1	-0.684	-0.063	0.747
Aldh6a1	-0.589	-0.481	1.070
Aldh9a1	-0.568	-0.189	0.757
Aldoa	-0.663	-0.282	0.945
Alg14	-0.698	-0.120	0.818
Alg3	-0.620	-0.274	0.894
Alg8	-0.622	-0.064	0.686
Alox5	-0.570	-0.449	1.020
Alyref	-0.615	-0.222	0.837
Amica1	-0.734	0.012	0.722
Ammecr1	-0.665	0.016	0.649
Anapc10	-0.486	-0.384	0.870

Anapc16	-0.699	-0.037	0.735
Ang	-0.722	-0.134	0.856
Angptl2	-0.577	-0.250	0.827
Ankrd13a	-0.568	-0.264	0.832
Ankrd34a	-0.885	0.109	0.776
Ankrd49	-0.699	-0.071	0.770
Ankrd55	-0.645	-0.117	0.762
Anln	-0.578	-0.186	0.764
Ano10	-0.573	-0.137	0.710
Anpep	-0.617	-0.201	0.818
Anxa4	-0.540	-0.232	0.772
Anxa5	-0.578	-0.264	0.843
Ap1s3	-0.839	0.088	0.751
Ap2a2	-0.608	-0.092	0.700
Ap2m1	-0.635	-0.280	0.916
Ap5m1	-0.842	0.049	0.793
Apbb1ip	-0.532	-0.179	0.711
Apex1	-0.745	-0.182	0.928
Apol9a	-0.496	-0.370	0.866
Apold1	-0.571	-0.180	0.751
Apool	-0.697	-0.327	1.024
App	-0.623	-0.205	0.828
Appbp2	-0.680	-0.088	0.768
Aqp5	-0.558	-0.279	0.837
Arcn1	-0.713	-0.214	0.927
Arf1	-0.623	-0.264	0.887
Arf2	-0.603	-0.089	0.693
Arfgap3	-0.581	-0.230	0.811
Arfip1	-0.546	-0.224	0.770
Arhgap1	-0.590	-0.163	0.754
Arhgap29	-0.557	-0.211	0.768
Arhgap31	-0.656	-0.176	0.833
Arhgap5	-0.511	-0.217	0.729
Arhgdia	-0.724	-0.243	0.967
Arhgef3	-0.597	-0.228	0.825
Arl1	-0.685	-0.259	0.944
Arl3	-0.528	-0.355	0.883
Arl5b	-0.570	-0.182	0.752
Arl6ip5	-0.639	-0.162	0.801
Arl6ip6	-0.633	-0.096	0.729
Arl8a	-0.665	-0.131	0.795
Arl8b	-0.718	-0.119	0.837
Armc1	-0.712	-0.272	0.984
Armcx6	-0.514	-0.256	0.770
Arpc1a	-0.554	-0.202	0.756
Arpc1b	-0.738	-0.132	0.870
Arpc4	-0.721	-0.181	0.902
Arrdc3	-0.644	-0.218	0.862
Arsa	-0.660	-0.121	0.782
Arsg	-0.779	-0.030	0.809
Art4	-0.736	-0.129	0.865
Asah2	-0.681	-0.130	0.811
Asna1	-0.721	-0.167	0.888
Ass1	-0.593	-0.124	0.717
Atad1	-0.701	-0.205	0.906
Atad3aos	-0.463	-0.376	0.839

Ate1	-0.524	-0.198	0.722
Atf2	-0.604	-0.088	0.691
Atf6	-0.672	-0.184	0.856
Atg10	-0.606	-0.139	0.745
Atg3	-0.682	-0.109	0.791
Atg4a	-0.492	-0.341	0.833
Atl2	-0.662	-0.053	0.715
Atl3	-0.554	-0.150	0.704
Atp11c	-0.619	-0.118	0.737
Atp1a1	-0.664	-0.207	0.871
Atp1a3	-0.711	-0.122	0.834
Atp1b1	-0.494	-0.348	0.842
Atp1b3	-0.698	-0.092	0.790
Atp5b	-0.557	-0.317	0.874
Atp5d	-0.596	-0.285	0.881
Atp5g3	-0.686	-0.194	0.879
Atp6ap1	-0.720	-0.196	0.916
Atp6ap2	-0.801	-0.167	0.968
Atp6v0d1	-0.714	-0.235	0.949
Atp6v1a	-0.650	-0.193	0.843
Atp6v1c1	-0.625	-0.158	0.783
Atp6v1e1	-0.675	-0.268	0.942
Atp6v1f	-0.701	-0.181	0.882
Atp7a	-0.602	-0.147	0.749
Atp8b1	-0.661	0.012	0.649
Atraid	-0.653	-0.238	0.891
Atrn	-0.599	-0.191	0.789
Atxn10	-0.571	-0.226	0.797
AU021092	-0.561	-0.370	0.930
Aurka	-0.620	-0.169	0.788
Avpr1a	-0.772	0.002	0.771
AW112010	-0.849	-0.056	0.906
AW549877	-0.571	-0.215	0.786
B230118H07Rik	-0.456	-0.358	0.813
B3galnt1	-0.662	-0.364	1.025
B3glct	-0.652	-0.080	0.732
B3gnt2	-0.537	-0.187	0.724
B3gnt3	-0.699	-0.256	0.955
B4galt1	-0.747	-0.132	0.878
Bag1	-0.539	-0.214	0.753
Bag3	-0.705	-0.151	0.856
Bag5	-0.674	-0.163	0.837
Basp1	-0.706	-0.022	0.728
Batf	-0.792	-0.099	0.891
Bbs5	-0.684	-0.048	0.732
BC004004	-0.599	-0.200	0.800
BC028528	-0.766	-0.060	0.826
BC029722	-0.704	-0.111	0.815
BC031181	-0.547	-0.317	0.864
Bcam	-0.536	-0.298	0.834
Bcap29	-0.768	-0.148	0.916
Bcap31	-0.634	-0.306	0.940
Bcar3	-0.769	-0.026	0.795
Bckdha	-0.710	-0.122	0.833
Bcl10	-0.630	-0.112	0.742
Bcl2a1a	-0.933	0.107	0.825

Bcs1l	-0.996	0.137	0.859
Bdnf	-0.607	-0.071	0.678
Bend3	-0.591	-0.132	0.723
Best1	-0.581	-0.393	0.974
Bet1	-0.799	-0.077	0.876
Bet1l	-0.706	-0.226	0.932
Bex4	-0.594	-0.364	0.958
Bhlhe40	-0.543	-0.205	0.748
Bicc1	-0.515	-0.396	0.911
Bicd1	-0.451	-0.321	0.773
Birc3	-0.701	0.026	0.675
Bivm	-0.558	-0.183	0.741
Blm	-0.627	-0.138	0.765
Bloc1s1	-0.606	-0.404	1.010
Bloc1s5	-0.593	-0.155	0.748
Bloc1s6	-0.593	-0.132	0.725
Blzf1	-0.618	-0.214	0.832
Bmi1	-0.678	-0.119	0.797
Bmp5	-0.605	-0.335	0.940
Bmpr1a	-0.603	-0.224	0.827
Bnip3l	-0.585	-0.123	0.708
Borcs8	-0.638	-0.128	0.766
Bpnt1	-0.664	-0.329	0.993
Brcc3	-0.739	-0.060	0.799
Bre	-0.583	-0.220	0.803
Brox	-0.784	-0.122	0.907
Bsg	-0.534	-0.325	0.859
Btbd1	-0.685	-0.139	0.823
Btf3	-0.648	-0.245	0.892
Btf3l4	-0.594	-0.337	0.932
Btg1	-0.685	0.031	0.655
Bub1	-0.728	-0.140	0.868
Bub3	-0.587	-0.379	0.966
Bysl	-0.618	-0.245	0.863
Bzw1	-0.782	-0.114	0.896
Bzw2	-0.555	-0.228	0.783
C1galt1	-0.579	-0.225	0.805
C1qa	-0.839	-0.048	0.887
C1qb	-0.832	-0.030	0.863
C1qbp	-0.667	-0.266	0.933
C1qtnf1	-0.465	-0.315	0.780
C1qtnf2	-0.677	-0.144	0.821
C4b	-0.515	-0.351	0.865
C5ar2	-1.013	0.167	0.846
Cab39	-0.714	-0.194	0.908
Cacul1	-0.687	-0.125	0.812
Cacybp	-0.657	-0.144	0.801
Cadm3	-0.756	-0.154	0.910
Cald1	-0.716	-0.094	0.810
Calhm2	-0.712	-0.025	0.737
Calm1	-0.722	-0.262	0.984
Calm2	-0.674	-0.360	1.034
Calr	-0.722	-0.169	0.891
Canx	-0.686	-0.147	0.833
Capn2	-0.657	-0.196	0.854
Capn5	-0.738	-0.023	0.761

Caprin1	-0.806	-0.124	0.931
Capzb	-0.663	-0.221	0.883
Car2	-0.704	-0.227	0.931
Car5b	-0.628	-0.257	0.886
Card19	-0.662	-0.208	0.870
Carnmt1	-0.677	-0.202	0.879
Casc4	-0.525	-0.277	0.801
Cask	-0.589	-0.187	0.776
Caskin2	-0.569	-0.146	0.715
Casp4	-0.523	-0.293	0.816
Cass4	-0.596	-0.174	0.770
Cast	-0.599	-0.154	0.753
Cbfb	-0.645	-0.143	0.787
Cblb	-0.675	-0.010	0.685
Cbll1	-0.689	-0.135	0.824
Cbx3	-0.494	-0.286	0.780
Cbx6	-0.490	-0.258	0.748
Ccdc109b	-0.692	-0.252	0.945
Ccdc115	-0.581	-0.232	0.813
Ccdc124	-0.472	-0.299	0.771
Ccdc127	-0.571	-0.162	0.734
Ccdc186	-0.650	-0.137	0.787
Ccdc32	-0.602	-0.107	0.709
Ccdc42	-0.639	-0.199	0.838
Ccdc47	-0.609	-0.333	0.941
Ccdc53	-0.535	-0.191	0.726
Ccdc58	-0.728	-0.093	0.820
Ccdc68	-0.630	-0.075	0.705
Ccdc71l	-0.676	0.018	0.657
Ccdc86	-0.695	-0.224	0.918
Ccdc90b	-0.747	-0.117	0.864
Ccna2	-0.701	-0.107	0.808
Ccnb1	-0.536	-0.385	0.921
Ccnc	-0.613	-0.119	0.732
Ccne1	-0.749	-0.059	0.808
Ccne2	-0.725	-0.065	0.790
Ccng1	-0.571	-0.229	0.800
Ccnh	-0.657	-0.114	0.770
Ccny	-0.789	-0.150	0.939
Cct2	-0.607	-0.220	0.827
Cct3	-0.691	-0.261	0.953
Cct4	-0.659	-0.145	0.805
Cct5	-0.691	-0.204	0.895
Cct6a	-0.660	-0.149	0.809
Cct7	-0.536	-0.176	0.712
Cct8	-0.755	-0.169	0.924
Ccz1	-0.585	-0.217	0.802
Cd101	-0.635	-0.094	0.729
Cd200r4	-0.829	0.041	0.788
Cd300a	-0.688	0.032	0.656
Cd300lf	-0.596	-0.152	0.748
Cd38	-0.730	-0.057	0.788
Cd3eap	-0.840	-0.018	0.858
Cd46	-0.651	-0.337	0.989
Cd47	-0.753	-0.191	0.944
Cd48	-0.657	-0.026	0.682

Cd63	-0.751	-0.159	0.910
Cd81	-0.770	-0.177	0.946
Cdc27	-0.683	-0.163	0.846
Cdc42	-0.630	-0.135	0.764
Cdc42bpa	-0.537	-0.186	0.723
Cdc42ep1	-0.627	-0.168	0.795
Cdc42ep4	-0.763	0.016	0.747
Cdca3	-0.664	-0.109	0.773
Cdcp1	-0.616	-0.194	0.810
Cdh13	-0.629	-0.296	0.925
Cdk1	-0.703	0.028	0.675
Cdk19	-0.564	-0.172	0.736
Cdk2ap2	-0.685	-0.214	0.898
Cdkn2d	-0.457	-0.316	0.773
Cdr2	-0.565	-0.229	0.794
Cebpb	-0.671	-0.152	0.823
Cebpz	-0.584	-0.127	0.712
Celf2	-0.732	-0.036	0.768
Cenph	-0.499	-0.240	0.738
Cenpl	-0.512	-0.222	0.734
Cenpq	-0.549	-0.227	0.776
Cep170	-0.672	-0.017	0.689
Cep41	-0.866	0.029	0.836
Cers2	-0.735	-0.251	0.986
Cers6	-0.831	-0.087	0.918
Ces1d	-0.465	-0.375	0.840
Cetn3	-0.595	-0.180	0.775
Cfap97	-0.603	-0.118	0.721
Cfl2	-0.757	-0.143	0.900
Cflar	-0.612	-0.104	0.716
Chchd1	-0.699	-0.075	0.774
Chchd4	-0.691	-0.098	0.789
Chchd7	-0.676	-0.091	0.767
Chm	-0.641	-0.316	0.957
Chmp2a	-0.482	-0.316	0.798
Chmp2b	-0.653	-0.174	0.827
Chmp3	-0.532	-0.224	0.755
Chmp5	-0.605	-0.191	0.796
Chp1	-0.790	-0.159	0.950
Chpt1	-0.743	-0.125	0.868
Chrac1	-0.559	-0.249	0.808
Chrnb1	-0.669	0.017	0.653
Chst11	-0.823	-0.089	0.912
Chsy1	-0.786	-0.053	0.839
Chtf8	-0.572	-0.150	0.722
Churc1	-0.607	-0.303	0.909
Cib2	-0.690	-0.032	0.723
Cinp	-0.695	-0.265	0.960
Cirh1a	-0.711	-0.202	0.914
Cisd1	-0.599	-0.107	0.706
Cisd3	-0.651	-0.217	0.868
Ckap4	-0.627	-0.078	0.705
Cks1b	-0.580	-0.215	0.795
Clcc1	-0.539	-0.195	0.733
Clcn5	-0.672	-0.096	0.768
Cldn10	-0.485	-0.352	0.837

Cldn15	-0.639	-0.085	0.724
Cldn23	-0.543	-0.164	0.707
Cldn34c1	-0.918	0.084	0.834
Cldn6	-0.623	-0.152	0.775
Cldnd1	-0.670	-0.192	0.862
Clec11a	-0.491	-0.264	0.755
Clec12a	-0.762	-0.095	0.856
Clec14a	-0.489	-0.315	0.804
Clec4a1	-0.794	-0.010	0.804
Clec5a	-0.690	-0.123	0.813
Clec7a	-0.735	0.070	0.665
Clic1	-0.790	-0.125	0.914
Clic5	-0.558	-0.309	0.867
Clint1	-0.647	-0.111	0.758
Clmp	-0.619	-0.280	0.899
Cln5	-0.667	-0.290	0.957
Cln6	-0.478	-0.366	0.844
Clock	-0.761	-0.176	0.937
Clp1	-0.816	0.013	0.803
Clpp	-0.659	-0.135	0.794
Clptm1	-0.825	-0.113	0.939
Clptm1l	-0.589	-0.158	0.747
Cltb	-0.545	-0.226	0.771
Cltc	-0.736	-0.249	0.985
Clybl	-0.662	-0.088	0.751
Cmc4	-0.728	-0.001	0.730
Cmklr1	-0.708	-0.198	0.906
Cmpk1	-0.789	-0.023	0.812
Cmtm3	-0.632	-0.075	0.707
Cnbp	-0.575	-0.188	0.764
Cndp2	-0.580	-0.302	0.882
Cnep1r1	-0.731	-0.047	0.778
Cnih1	-0.711	-0.139	0.850
Cnih4	-0.699	-0.207	0.906
Cnn2	-0.660	-0.279	0.939
Cnot11	-0.613	-0.159	0.772
Cnot2	-0.551	-0.261	0.812
Cnot6	-0.631	-0.082	0.712
Cnot6l	-0.628	-0.057	0.685
Cnpy2	-0.582	-0.167	0.749
Cnrip1	-0.553	-0.243	0.797
Coa4	-0.644	-0.123	0.767
Coa5	-0.604	-0.086	0.690
Coa6	-0.691	-0.338	1.029
Cobll1	-0.592	-0.108	0.700
Cog5	-0.559	-0.177	0.736
Col12a1	-0.861	-0.051	0.913
Col23a1	-0.636	-0.297	0.933
Col4a1	-0.668	-0.085	0.752
Col4a2	-0.684	-0.109	0.793
Col6a1	-0.586	-0.194	0.780
Col6a2	-0.620	-0.160	0.780
Col6a3	-0.645	-0.200	0.845
Col6a5	-0.766	0.059	0.707
Commd10	-0.482	-0.352	0.834
Commd3	-0.611	-0.216	0.828

Commd6	-0.603	-0.241	0.844
Commd9	-0.534	-0.183	0.717
Comt	-0.702	-0.120	0.822
Copb2	-0.632	-0.257	0.889
Cope	-0.604	-0.090	0.694
Copg1	-0.588	-0.132	0.719
Cops3	-0.537	-0.300	0.836
Cops4	-0.650	-0.196	0.846
Cops5	-0.584	-0.222	0.806
Cops6	-0.755	-0.203	0.958
Coq6	-0.745	-0.092	0.837
Coq9	-0.562	-0.148	0.710
Coro1b	-0.575	-0.137	0.712
Coro1c	-0.563	-0.161	0.723
Coro2b	-0.586	-0.132	0.719
Cotl1	-0.720	-0.155	0.874
Cox19	-0.531	-0.192	0.723
Cox4i2	-0.502	-0.239	0.741
Cox5a	-0.654	-0.262	0.916
Cox6c	-0.611	-0.320	0.931
Cpd	-0.657	-0.204	0.861
Cpne3	-0.778	-0.159	0.937
Cpne8	-0.738	-0.114	0.852
Cpped1	-0.537	-0.182	0.719
Cpsf3	-0.538	-0.171	0.710
Cpt1a	-0.775	-0.176	0.951
Cpt2	-0.630	-0.104	0.735
Cr1l	-0.694	-0.108	0.802
Crat	-0.557	-0.239	0.796
Crbn	-0.687	-0.168	0.854
Crebbp	-0.603	-0.092	0.695
Creg1	-0.476	-0.303	0.779
Crem	-0.843	-0.065	0.908
Crip2	-0.488	-0.313	0.801
Cript	-0.703	-0.226	0.929
Crk	-0.612	-0.095	0.707
Crls1	-0.654	-0.234	0.889
Crtap	-0.594	-0.290	0.883
Cry1	-0.618	-0.168	0.786
Crybg3_1	-0.580	-0.179	0.759
Csde1	-0.651	-0.191	0.843
Csf1	-0.614	-0.157	0.771
Csgalnact1	-0.719	-0.079	0.798
Csnk2b	-0.690	-0.208	0.898
Csrp1	-0.552	-0.257	0.810
Cstf2	-0.527	-0.244	0.771
Ctbp2	-0.615	-0.065	0.681
Ctla4	-0.654	0.002	0.652
Ctnna1	-0.700	-0.298	0.998
Ctnnb1	-0.670	-0.327	0.997
Ctnnd1	-0.630	-0.072	0.703
Ctsa	-0.616	-0.112	0.729
Ctsc	-0.683	-0.177	0.860
Ctse	-0.580	-0.194	0.773
Ctsh	-0.605	-0.115	0.720
Ctsk	-0.707	-0.298	1.005

Ctss	-0.656	-0.015	0.672
Ctsz	-0.667	-0.170	0.837
Cttnbp2nl	-0.792	-0.055	0.847
Cubn	-0.793	0.054	0.739
Cul3	-0.653	-0.118	0.771
Cul4b	-0.689	-0.150	0.839
Cul5	-0.698	-0.115	0.813
Cwc15	-0.581	-0.223	0.805
Cxcr4	-0.660	-0.103	0.763
Cyb5r3	-0.576	-0.149	0.725
Cyb5r4	-0.642	-0.120	0.762
Cyba	-0.656	-0.254	0.910
Cyc1	-0.581	-0.158	0.739
Cyfip1	-0.651	-0.243	0.894
Cyp2d22	-0.551	-0.407	0.958
Cyp2j6	-0.614	-0.153	0.767
Cyp2u1	-0.566	-0.230	0.796
Cyp51	-0.658	-0.114	0.773
Cyth3	-0.580	-0.188	0.768
Cytip	-0.778	-0.009	0.788
Cyyr1	-0.771	-0.172	0.943
D3Ertd254e	-0.543	-0.252	0.794
D8Ertd738e	-0.615	-0.202	0.818
Daam1	-0.551	-0.163	0.714
Dad1	-0.628	-0.240	0.868
Dag1	-0.592	-0.138	0.730
Dars	-0.573	-0.287	0.860
Dbr1	-0.735	-0.147	0.882
Dbt	-0.627	-0.264	0.891
Dcaf10	-0.629	-0.124	0.753
Dcaf13	-0.569	-0.187	0.756
Dchs1	-0.623	-0.045	0.669
Dclre1b	-0.736	-0.126	0.861
Dctd	-0.679	-0.249	0.928
Dctn4	-0.635	-0.215	0.850
Dctn6	-0.629	-0.149	0.778
Dcun1d1	-0.598	-0.219	0.817
Dda1	-0.625	-0.182	0.808
Ddah1	-0.698	-0.205	0.902
Ddb1	-0.682	-0.211	0.893
Ddias	-0.528	-0.321	0.849
Ddit3	-0.750	-0.008	0.758
Ddit4	-0.782	-0.006	0.788
Ddost	-0.737	-0.239	0.975
Ddx1	-0.641	-0.243	0.883
Ddx18	-0.582	-0.141	0.722
Ddx21	-0.742	-0.207	0.950
Ddx6	-0.609	-0.112	0.720
Deb1	-0.677	-0.027	0.704
Def8	-0.625	-0.160	0.786
Dennd4a	-0.673	-0.045	0.718
Dennd4c	-0.608	-0.166	0.774
Depdc1a	-0.790	0.034	0.756
Deptor	-0.580	-0.199	0.779
Dera	-0.718	-0.033	0.751
Derl1	-0.615	-0.176	0.791

Desi2	-0.677	-0.100	0.777
Dhcr7	-0.576	-0.131	0.707
Dhdh	-0.497	-0.266	0.763
Dhx29	-0.543	-0.301	0.844
Dhx40	-0.705	-0.196	0.901
Dhx9	-0.614	-0.106	0.720
Disp2	-0.750	-0.155	0.905
Dkc1	-0.672	-0.183	0.855
Dkk3	-0.584	-0.125	0.709
Dlat	-0.603	-0.134	0.738
Dld	-0.812	-0.150	0.962
Dll1	-0.792	-0.049	0.841
Dmxl2	-0.604	-0.141	0.744
Dnaja2	-0.660	-0.129	0.789
Dnaja3	-0.546	-0.240	0.786
Dnajb1	-0.644	-0.055	0.699
Dnajb11	-0.707	-0.191	0.898
Dnajb13	-0.712	-0.031	0.743
Dnajb4	-0.663	-0.196	0.858
Dnajc15	-0.707	-0.214	0.921
Dnajc25	-0.850	0.033	0.817
Dnajc3	-0.568	-0.226	0.794
Dnajc6	-0.813	0.049	0.764
Dnm1l	-0.769	-0.145	0.914
Dpep2	-0.707	-0.009	0.715
Dph3	-0.755	-0.139	0.894
Dph5	-0.641	-0.121	0.763
Dph6	-0.646	-0.154	0.799
Dpm2	-0.561	-0.438	0.999
Dpy19l1	-0.626	-0.209	0.835
Dpy30	-0.627	-0.296	0.923
Dpysl2	-0.497	-0.289	0.785
Dpysl3	-0.590	-0.185	0.775
Dpysl5	-0.671	-0.180	0.851
Dr1	-0.712	-0.074	0.786
Drap1	-0.584	-0.191	0.775
Drg1	-0.628	-0.101	0.729
Dse	-0.751	-0.201	0.952
Dsel	-0.606	-0.182	0.788
Dsp	-0.515	-0.215	0.730
Dstn	-0.609	-0.347	0.957
Dtd1	-0.507	-0.244	0.751
Dtx4	-0.672	-0.189	0.861
Dus4l	-0.759	0.041	0.718
Dusp22	-0.592	-0.190	0.782
Dusp3	-0.621	-0.219	0.841
Dusp7	-0.601	-0.134	0.736
Dusp8	-0.636	-0.105	0.741
Dync1i2	-0.608	-0.124	0.732
Dync1li1	-0.643	-0.116	0.758
E030013I19Rik	-0.632	-0.233	0.864
E130311K13Rik	-0.825	0.110	0.716
E430018J23Rik	-0.573	-0.165	0.738
E430025E21Rik	-0.533	-0.243	0.775
Ebi3	-0.539	-0.248	0.787
Ecd	-0.550	-0.183	0.734

Ece1	-0.704	-0.277	0.981
Ece2	-0.787	-0.056	0.843
Ech1	-0.641	-0.098	0.739
Echs1	-0.630	-0.144	0.775
Eci1	-0.612	-0.445	1.057
Eci2	-0.649	-0.243	0.892
Eda2r	-0.634	-0.120	0.754
Edem3	-0.752	-0.140	0.892
Ednra	-0.484	-0.354	0.838
Ednrb	-0.621	-0.379	1.000
Eea1	-0.666	-0.153	0.819
Eed	-0.555	-0.189	0.744
Eef1a1	-0.557	-0.147	0.703
Eef1e1	-0.727	-0.162	0.889
Efcab10	-0.579	-0.297	0.876
Efcab14	-0.644	-0.176	0.820
Efhd2	-0.625	-0.075	0.699
Efnb1	-0.624	-0.136	0.761
Efnb2	-0.507	-0.233	0.740
Efr3a	-0.573	-0.210	0.783
Eftud2	-0.627	-0.162	0.789
Egflam	-0.684	-0.142	0.826
Egr2	-0.669	0.005	0.664
Ehd1	-0.652	-0.219	0.872
Ehd2	-0.688	-0.228	0.916
Ehd3	-0.628	-0.057	0.685
Ehd4	-0.552	-0.214	0.766
Eif1	-0.770	-0.151	0.921
Eif1ax	-0.670	-0.238	0.908
Eif1b	-0.578	-0.321	0.899
Eif2a	-0.739	-0.135	0.873
Eif2ak1	-0.648	-0.226	0.874
Eif2b1	-0.677	-0.262	0.939
Eif2s2	-0.653	-0.262	0.916
Eif2s3x	-0.635	-0.148	0.782
Eif3b	-0.529	-0.219	0.748
Eif3c	-0.608	-0.248	0.855
Eif3d	-0.607	-0.179	0.786
Eif3e	-0.802	-0.109	0.912
Eif3f	-0.673	-0.180	0.853
Eif3g	-0.568	-0.273	0.841
Eif3h	-0.623	-0.181	0.804
Eif3j1	-0.624	-0.180	0.804
Eif3m	-0.599	-0.180	0.779
Eif4a3	-0.639	-0.372	1.011
Eif4e2	-0.610	-0.129	0.739
Eif4g2	-0.686	-0.137	0.824
Eif5	-0.652	-0.221	0.873
Eif5a2	-0.869	-0.040	0.910
Eif6	-0.618	-0.153	0.772
Elavl1	-0.789	-0.085	0.873
Elf1	-0.643	-0.084	0.727
Elfn1	-0.749	-0.038	0.787
Elk4	-0.680	-0.189	0.869
Ell2	-0.704	-0.206	0.909
Elmo2	-0.680	-0.077	0.757

Elovl5	-0.690	-0.160	0.851
Elovl6	-0.617	-0.228	0.846
Elovl7	-0.586	-0.262	0.848
Elp4	-0.745	-0.097	0.842
Elp5	-0.635	-0.056	0.691
Emb	-0.478	-0.335	0.813
Emc2	-0.587	-0.313	0.900
Emc8	-0.681	-0.104	0.785
Emilin1	-0.816	-0.127	0.942
Eml1	-0.522	-0.319	0.841
Emp2	-0.655	-0.192	0.847
Enc1	-0.711	-0.157	0.868
Enpep	-0.472	-0.370	0.842
Enpp1	-0.689	0.036	0.653
Enpp2	-0.613	-0.189	0.802
Enpp4	-0.564	-0.297	0.861
Enpp5	-0.545	-0.432	0.977
Entpd3	-0.842	0.014	0.828
Eny2	-0.658	-0.270	0.927
Epas1	-0.687	-0.283	0.970
Epb41	-0.614	-0.107	0.721
Epb41l2	-0.658	-0.079	0.737
Epha2	-0.626	-0.093	0.719
Epha3	-0.809	-0.095	0.904
Ephb2	-0.615	-0.059	0.674
Ephx2	-0.689	-0.006	0.695
Eprs	-0.641	-0.216	0.856
Eps8	-0.667	-0.232	0.898
Eral1	-0.630	-0.179	0.809
Erbb4	-0.564	-0.174	0.738
Ercc6l	-0.746	-0.017	0.763
Ercc6l2	-0.564	-0.243	0.807
Ercc8	-0.807	-0.145	0.952
Erg	-0.690	-0.083	0.773
Ergic2	-0.634	-0.077	0.711
Ergic3	-0.545	-0.227	0.772
Eri1	-0.595	-0.151	0.746
Erlin1	-0.772	-0.023	0.796
Ero1l	-0.708	-0.141	0.849
Esyt2	-0.601	-0.125	0.726
Etf1	-0.746	-0.202	0.948
Etfdh	-0.758	-0.158	0.916
Ethe1	-0.706	-0.292	0.999
Ets2	-0.686	-0.269	0.955
Etv1	-0.822	-0.136	0.958
Evi2a	-0.763	-0.009	0.771
Exoc5	-0.705	-0.111	0.816
Exosc1	-0.551	-0.270	0.821
Exosc4	-0.728	-0.037	0.765
Ext2	-0.719	-0.118	0.838
F5	-0.552	-0.420	0.972
Faap24	-0.866	0.041	0.824
Fads1	-0.536	-0.289	0.826
Fads2	-0.753	-0.221	0.974
Faim	-0.662	-0.168	0.829
Fam105a	-0.662	-0.040	0.702

Fam110c	-0.742	-0.023	0.765
Fam117b	-0.862	0.013	0.849
Fam118b	-0.664	-0.102	0.765
Fam126b	-0.770	-0.125	0.894
Fam129b	-0.552	-0.160	0.712
Fam136a	-0.765	-0.118	0.883
Fam169a	-0.574	-0.258	0.831
Fam171b	-0.632	-0.308	0.940
Fam175a	-0.569	-0.202	0.771
Fam198b	-0.696	-0.073	0.769
Fam199x	-0.783	0.031	0.752
Fam206a	-0.737	-0.180	0.916
Fam210b	-0.683	-0.223	0.906
Fam220a	-0.525	-0.291	0.816
Fam26f	-0.506	-0.247	0.753
Fam45a	-0.645	-0.238	0.883
Fam49a	-0.688	-0.198	0.886
Fam49b	-0.799	0.036	0.763
Fam58b	-0.598	-0.293	0.891
Fam63b	-0.684	-0.009	0.693
Fam65a	-0.593	-0.109	0.702
Fam78b	-0.828	-0.078	0.906
Fam83b	-0.916	0.046	0.871
Fam8a1	-0.721	-0.098	0.819
Fam91a1	-0.659	-0.146	0.804
Fam96b	-0.718	-0.175	0.892
Fam98a	-0.632	-0.142	0.774
Fam98b	-0.497	-0.336	0.833
Fancl	-0.726	-0.042	0.768
Far1	-0.614	-0.058	0.673
Farp1	-0.555	-0.160	0.715
Fasl	-0.769	-0.153	0.921
Fasn	-0.463	-0.357	0.820
Fastkd2	-0.609	-0.089	0.698
Fbl	-0.591	-0.197	0.788
Fbln2	-0.822	0.047	0.776
Fbln5	-0.684	-0.085	0.769
Fbn1	-0.719	-0.058	0.777
Fbxl5	-0.638	-0.134	0.772
Fbxo10	-0.521	-0.199	0.720
Fbxo11	-0.592	-0.115	0.707
Fbxo25	-0.628	-0.280	0.908
Fbxo28	-0.565	-0.183	0.748
Fbxo31	-0.549	-0.310	0.860
Fbxo33	-0.714	-0.134	0.849
Fbxo8	-0.513	-0.237	0.750
Fbxw11	-0.637	-0.126	0.763
Fcf1	-0.637	-0.266	0.903
Fech	-0.637	-0.203	0.840
Fermt2	-0.698	-0.292	0.989
Fgd4	-0.650	-0.102	0.752
Fgf10	-0.575	-0.275	0.849
Fgr	-0.631	-0.032	0.663
Fh1	-0.561	-0.288	0.849
Fhl1	-0.542	-0.170	0.712
Ficd	-0.583	-0.149	0.732

Filip1l	-0.532	-0.266	0.798
Fkbp10	-0.654	-0.067	0.721
Fkbp1a	-0.687	-0.247	0.934
Fkbp1b	-0.600	-0.257	0.857
Fkbp3	-0.565	-0.376	0.940
Fli1	-0.716	-0.190	0.906
Flna	-0.553	-0.158	0.712
Flnb	-0.745	-0.099	0.844
Flrt2	-0.585	-0.231	0.816
Flrt3	-0.683	-0.012	0.696
Flt4	-0.620	-0.262	0.882
Fmo4	-0.550	-0.365	0.915
Fnbp1l	-0.763	-0.079	0.842
Fndc3a	-0.601	-0.146	0.747
Fndc3b	-0.790	-0.077	0.867
Fnta	-0.506	-0.333	0.839
Fntb	-0.538	-0.222	0.760
Fosl1	-0.526	-0.190	0.716
Foxk2	-0.646	-0.091	0.737
Fpgt	-0.686	-0.156	0.842
Frg1	-0.504	-0.252	0.756
Frmd6	-0.580	-0.144	0.725
Frs2	-0.594	-0.108	0.703
Fstl1	-0.685	0.003	0.683
Fstl3	-0.693	-0.156	0.849
Fth1	-0.599	-0.422	1.021
Fuca2	-0.668	-0.170	0.838
Fundc2	-0.633	-0.176	0.809
Furin	-0.632	-0.066	0.698
Fut8	-0.718	-0.227	0.945
Fxyd6	-0.633	-0.039	0.672
Fzd4	-0.588	-0.238	0.825
G6pdx	-0.671	-0.242	0.913
Gaa	-0.623	-0.191	0.814
Gab2	-0.628	-0.094	0.722
Gabarap	-0.616	-0.185	0.801
Gabarapl1	-0.485	-0.303	0.788
Gadd45gip1	-0.543	-0.225	0.768
Galns	-0.829	-0.089	0.918
Galnt1	-0.602	-0.190	0.792
Galnt15	-0.478	-0.333	0.811
Galnt2	-0.641	-0.148	0.789
Ganab	-0.619	-0.149	0.767
Gar1	-0.909	0.077	0.832
Gars	-0.685	-0.210	0.895
Gas7	-0.643	-0.150	0.793
Gatad2a	-0.582	-0.172	0.754
Gatc	-0.740	-0.078	0.819
Gatm	-0.773	-0.107	0.879
Gba	-0.576	-0.193	0.768
Gbas	-0.662	-0.215	0.877
Gbe1	-0.534	-0.248	0.782
Gbp2	-0.573	-0.202	0.775
Gch1	-0.605	-0.370	0.975
Gclc	-0.521	-0.203	0.724
Gcnt1	-0.623	-0.116	0.739

Gcnt2	-0.498	-0.267	0.764
Gcsam	-0.549	-0.392	0.941
Gdap2	-0.660	-0.211	0.871
Gdf15	-0.860	-0.048	0.908
Gdi2	-0.657	-0.115	0.772
Gdnf	-0.531	-0.246	0.776
Gdpd1	-0.642	-0.189	0.831
Gdpd2	-0.582	-0.250	0.832
Gemin6	-0.755	-0.092	0.847
Gemin7	-0.764	-0.007	0.771
Get4	-0.609	-0.210	0.819
Gfm1	-0.616	-0.168	0.784
Gfra2	-0.632	-0.178	0.810
Ggps1	-0.722	-0.140	0.862
Ggta1	-0.633	-0.073	0.706
Ghitm	-0.629	-0.262	0.891
Ghr	-0.548	-0.193	0.741
Ghrl	-0.519	-0.216	0.735
Gin1	-0.843	-0.015	0.858
Ginm1	-0.615	-0.342	0.957
Gins1	-0.628	-0.221	0.849
Gins2	-0.635	-0.129	0.763
Gja1	-0.641	-0.330	0.971
Gk	-0.771	-0.057	0.828
Gkap1	-0.506	-0.254	0.760
Gla	-0.622	-0.200	0.822
Glb1	-0.645	-0.248	0.893
Glce	-0.751	-0.157	0.908
Glg1	-0.626	-0.122	0.748
Gli3	-0.790	-0.021	0.811
Glipr2	-0.698	-0.185	0.883
Glmp	-0.703	-0.216	0.919
Glod4	-0.528	-0.191	0.719
Glp2r	-0.502	-0.349	0.852
Glrx3	-0.618	-0.187	0.805
Glt8d1	-0.599	-0.131	0.730
Gltp	-0.664	-0.267	0.931
Gltscr1l	-0.630	-0.136	0.767
Glud1	-0.724	-0.172	0.896
Glul	-0.741	-0.210	0.952
Gm11110	-0.563	-0.337	0.901
Gm11696	-0.668	-0.134	0.802
Gm14005	-0.651	-0.164	0.815
Gm14295	-0.784	0.031	0.753
Gm14306	-0.493	-0.298	0.790
Gm14418	-0.621	-0.157	0.779
Gm14698	-0.774	0.024	0.750
Gm15441	-0.721	-0.055	0.776
Gm15706	-0.580	-0.247	0.827
Gm15931	-0.710	0.007	0.703
Gm16083	-0.639	-0.111	0.751
Gm26566	-0.821	-0.089	0.911
Gm26809	-0.813	-0.034	0.847
Gm28856	-0.975	0.152	0.823
Gm2a	-0.743	-0.157	0.900
Gm3604	-0.879	0.121	0.757

Gm37768	-0.756	0.049	0.708
Gm42031	-0.727	-0.124	0.851
Gm4285	-0.674	-0.008	0.682
Gm44226	-0.703	-0.044	0.747
Gm4956	-0.655	-0.325	0.980
Gm5617	-0.729	-0.012	0.741
Gm6209	-0.682	-0.261	0.944
Gm6712	-0.721	-0.068	0.789
Gm8797	-0.751	-0.092	0.844
Gm9776	-0.654	-0.031	0.685
Gm9938	-0.627	-0.034	0.661
Gmfb	-0.585	-0.147	0.731
Gmfg	-0.621	-0.148	0.769
Gmpr2	-0.629	-0.266	0.895
Gmps	-0.568	-0.202	0.770
Gnai1	-0.704	0.029	0.675
Gnai2	-0.666	-0.178	0.844
Gnai3	-0.591	-0.178	0.769
Gnb1	-0.635	-0.246	0.881
Gnb2	-0.655	-0.235	0.890
Gnl3l	-0.604	-0.163	0.767
Gnpda2	-0.550	-0.197	0.747
Gnptg	-0.634	-0.283	0.916
Gns	-0.594	-0.176	0.770
Golga5	-0.596	-0.146	0.742
Golim4	-0.663	-0.050	0.713
Golm1	-0.533	-0.173	0.705
Golph3	-0.566	-0.159	0.724
Golph3l	-0.634	-0.075	0.709
Golt1b	-0.798	-0.098	0.895
Gopc	-0.552	-0.158	0.710
Got2	-0.689	-0.255	0.944
Gpalpp1	-0.710	-0.144	0.854
Gpbp1	-0.540	-0.238	0.778
Gpc1	-0.821	0.086	0.735
Gpc3	-0.717	-0.267	0.984
Gpcpd1	-0.590	-0.149	0.739
Gpi1	-0.583	-0.146	0.729
Gpnmb	-0.559	-0.232	0.791
Gpr180	-0.538	-0.275	0.814
Gpr3	-0.767	-0.101	0.868
Gpr65	-0.889	0.060	0.829
Gpr88	-0.897	0.057	0.840
Gpr89	-0.801	0.041	0.760
Gps1	-0.560	-0.274	0.834
Gpx1	-0.613	-0.198	0.810
Gpx7	-0.644	-0.318	0.961
Grb10	-0.710	-0.084	0.794
Grhpr	-0.664	-0.146	0.810
Gria3	-0.781	0.059	0.722
Grina	-0.670	-0.134	0.803
Grn	-0.684	-0.194	0.878
Grp	-0.718	-0.033	0.751
Gsk3a	-0.575	-0.188	0.763
Gskip	-0.673	0.039	0.634
Gspt1	-0.669	-0.258	0.927

Gsr	-0.682	-0.273	0.955
Gstp1	-0.507	-0.390	0.897
Gstt1	-0.609	-0.242	0.850
Gtf2a1	-0.586	-0.211	0.796
Gtf2a2	-0.757	-0.115	0.871
Gtf2b	-0.544	-0.263	0.807
Gtf2f2	-0.672	-0.266	0.938
Gtf2h4	-0.526	-0.318	0.845
Gtf2h5	-0.591	-0.201	0.792
Gtf3a	-0.776	0.048	0.728
Gtf3c6	-0.757	-0.199	0.957
Gtpbp10	-0.583	-0.226	0.809
Gtpbp8	-0.729	0.072	0.657
Gucy1a3	-0.540	-0.183	0.723
Gucy1b3	-0.699	-0.231	0.930
Gusb	-0.679	-0.130	0.809
Gypc	-0.729	-0.060	0.789
H1f0	-0.496	-0.288	0.784
H3f3b	-0.639	-0.242	0.881
Hadh	-0.512	-0.254	0.766
Hadha	-0.515	-0.209	0.724
Hadhb	-0.700	-0.091	0.790
Hars	-0.583	-0.147	0.730
Has2	-0.834	-0.074	0.908
Hat1	-0.712	-0.112	0.824
Haus2	-0.798	-0.108	0.906
Haus3	-0.775	-0.108	0.883
Havcr2	-0.730	-0.058	0.788
Hbp1	-0.789	-0.153	0.942
Hccs	-0.707	-0.054	0.762
Hcfc1	-0.664	-0.166	0.830
Hdlbp	-0.716	-0.206	0.922
Heatr1	-0.634	-0.181	0.815
Hecw2	-0.818	-0.074	0.892
Heg1	-0.564	-0.142	0.706
Heph	-0.701	-0.090	0.791
Herpud1	-0.675	-0.124	0.799
Herpud2	-0.704	-0.215	0.919
Hey2	-0.603	-0.254	0.857
Hibadh	-0.656	-0.248	0.904
Higd2a	-0.577	-0.294	0.871
Hikeshi	-0.774	-0.120	0.894
Hipk3	-0.688	-0.164	0.852
Hist1h1a	-0.481	-0.320	0.801
Hist1h1b	-0.576	-0.350	0.926
Hist1h1c	-0.643	-0.193	0.835
Hist1h1d	-0.698	-0.133	0.831
Hist1h1e	-0.526	-0.248	0.774
Hist1h2ak	-0.680	-0.116	0.797
Hist1h2bl	-0.649	-0.196	0.846
Hist1h3g	-0.514	-0.334	0.848
Hmga1	-0.537	-0.307	0.844
Hmgb1	-0.545	-0.197	0.743
Hmgcs1	-0.499	-0.286	0.785
Hmgcs2	-0.569	-0.366	0.935
Hmgn1	-0.512	-0.232	0.744

Hn1l	-0.758	-0.159	0.918
Hnrnpa3	-0.651	-0.216	0.867
Hnrnpab	-0.560	-0.250	0.810
Hnrnpd	-0.583	-0.197	0.780
Hnrnph2	-0.803	-0.106	0.909
Hnrnpk	-0.720	-0.124	0.844
Hopx	-0.524	-0.338	0.862
Hotairm1	-0.776	-0.053	0.829
Hpgds	-0.753	0.022	0.730
Hprt	-0.693	-0.054	0.746
Hscb	-0.828	-0.053	0.881
Hsd17b11	-0.744	-0.169	0.913
Hsd17b7	-0.751	-0.188	0.939
Hsd3b7	-0.798	-0.041	0.839
Hsdl2	-0.670	-0.366	1.036
Hsf2	-0.551	-0.155	0.706
Hsp90aa1	-0.708	-0.231	0.939
Hsp90ab1	-0.667	-0.119	0.786
Hsp90b1	-0.744	-0.182	0.926
Hspa12b	-0.634	-0.072	0.706
Hspa4	-0.557	-0.178	0.735
Hspa9	-0.694	-0.225	0.919
Hspb2	-0.576	-0.240	0.816
Hspb8	-0.753	-0.167	0.920
Hspd1	-0.704	-0.230	0.934
Htatip2	-0.593	-0.206	0.799
Hus1	-0.468	-0.377	0.845
Hypk	-0.531	-0.398	0.929
I830077J02Rik	-0.810	0.005	0.806
Iars	-0.717	-0.127	0.844
Ica1l	-0.785	0.102	0.682
Icam1	-0.572	-0.211	0.783
Ick	-0.490	-0.296	0.785
Id1	-0.734	-0.123	0.857
Id2	-0.726	-0.223	0.949
Idh1	-0.518	-0.356	0.874
Idi1	-0.711	-0.261	0.972
Ids	-0.697	-0.092	0.790
Ifi207	-0.870	0.045	0.826
Ifi30	-0.713	-0.014	0.727
Ifi47	-0.594	-0.345	0.938
Ifih1	-0.586	-0.239	0.825
Ifitm10	-0.818	-0.018	0.836
Ifnar2	-0.592	-0.159	0.751
Ifngr1	-0.773	-0.188	0.961
Ifrd1	-0.582	-0.140	0.722
Ift20	-0.512	-0.301	0.814
Ift27	-0.671	-0.181	0.852
Igf2	-0.528	-0.253	0.781
Igf2bp2	-0.805	0.022	0.783
Igf2r	-0.582	-0.176	0.758
Igfbp3	-0.760	-0.118	0.878
Ighv3-6	-0.579	-0.457	1.036
Igip	-0.873	-0.028	0.901
Igkv6-32	-0.631	-0.263	0.895
Iglon5	-0.732	0.065	0.667

Iigp1	-0.619	-0.075	0.695
Ik	-0.630	-0.172	0.801
Ikbip	-0.562	-0.200	0.762
Il1r1	-0.695	-0.022	0.717
Il1rap	-0.801	-0.139	0.940
Il2rg	-0.919	0.067	0.852
Il4ra	-0.727	0.018	0.709
Il6ra	-0.703	-0.019	0.722
Il6st	-0.661	-0.165	0.826
Il7	-0.636	-0.090	0.726
Ilk	-0.644	-0.247	0.892
Immp1l	-0.681	-0.068	0.748
Immt	-0.620	-0.199	0.819
Imp4	-0.562	-0.171	0.733
Impdh2	-0.693	-0.198	0.890
Inafm2	-0.762	-0.167	0.929
Ing3	-0.573	-0.151	0.725
Inhba	-0.756	0.075	0.681
Inip	-0.686	-0.008	0.694
Ino80c	-0.635	-0.125	0.761
Ints12	-0.592	-0.133	0.725
Ints6	-0.651	-0.080	0.731
Ipo11	-0.545	-0.268	0.813
Ipo5	-0.657	-0.266	0.923
Ipo7	-0.801	-0.168	0.969
Iqgap1	-0.576	-0.246	0.822
Irak4	-0.615	-0.089	0.704
Ireb2	-0.602	-0.144	0.746
Irs2	-0.821	-0.134	0.955
Irs3	-0.707	-0.122	0.829
Irx1	-0.595	-0.321	0.916
Isca1	-0.518	-0.252	0.770
Isca2	-0.559	-0.271	0.830
Iscu	-0.690	-0.277	0.966
Islr	-0.591	-0.123	0.714
Itfg1	-0.663	-0.178	0.841
Itga1	-0.661	-0.185	0.845
Itga2	-0.663	-0.308	0.971
Itga3	-0.557	-0.172	0.729
Itga5	-0.785	-0.167	0.952
Itga6	-0.694	-0.167	0.861
Itga8	-0.649	-0.362	1.010
Itgav	-0.733	-0.079	0.812
Itgb1bp1	-0.661	-0.146	0.807
Itgb2	-0.652	-0.137	0.789
Itgb3	-0.693	0.011	0.683
Itgb3bp	-0.607	-0.426	1.033
Itih3	-0.547	-0.259	0.806
Itpa	-0.505	-0.287	0.792
Itpripl2	-0.637	-0.120	0.757
Jade2	-0.738	-0.026	0.765
Jak1	-0.615	-0.139	0.754
Jazf1	-0.622	-0.131	0.753
Jkamp	-0.701	-0.261	0.962
Jun	-0.505	-0.235	0.741
Jup	-0.662	-0.210	0.872

Kars	-0.620	-0.385	1.005
Kbtbd4	-0.697	-0.146	0.843
Kcnab1	-0.737	-0.135	0.872
Kcnh7	-0.784	-0.168	0.953
Kcnj15	-0.651	-0.300	0.951
Kcnn1	-0.792	0.087	0.705
Kdelc1	-0.709	-0.107	0.816
Kdelc2	-0.615	-0.284	0.899
Kdelr1	-0.684	-0.152	0.836
Kdsr	-0.748	-0.082	0.830
Khdrbs3	-0.519	-0.210	0.729
Kif18a	-0.679	0.028	0.651
Kif5b	-0.611	-0.188	0.799
Kirrel	-0.644	-0.091	0.735
Kitl	-0.693	-0.112	0.805
Klf10	-0.783	-0.028	0.811
Klf16	-0.664	0.013	0.651
Klf2	-0.813	-0.036	0.849
Klf3	-0.668	-0.039	0.706
Klf6	-0.717	-0.169	0.886
Klf7	-0.710	-0.077	0.787
Klhl11	-0.549	-0.176	0.725
Klhl2	-0.825	-0.104	0.929
Klhl29	-0.802	0.003	0.799
Klhl9	-0.730	-0.188	0.918
Klra2	-0.820	-0.046	0.865
Klrb1a	-0.862	0.000	0.862
Knop1	-0.572	-0.153	0.725
Kpna1	-0.829	-0.096	0.925
Kpna2	-0.695	0.027	0.669
Kpna4	-0.781	-0.105	0.886
Krt18	-0.564	-0.234	0.798
Krt7	-0.666	-0.274	0.940
Krt8	-0.685	-0.306	0.992
Lactb	-0.614	-0.151	0.765
Lactb2	-0.666	-0.264	0.930
Lair1	-0.710	-0.163	0.872
Lama2	-0.629	-0.295	0.924
Lamb1	-0.754	-0.162	0.916
Lamb3	-0.656	-0.150	0.806
Lamc1	-0.660	-0.176	0.836
Lamc2	-0.742	-0.217	0.959
Lamtor1	-0.658	-0.340	0.998
Lamtor2	-0.593	-0.347	0.940
Lamtor3	-0.697	-0.294	0.991
Lamtor4	-0.653	-0.080	0.733
Lap3	-0.583	-0.201	0.784
Laptm4b	-0.557	-0.267	0.824
Larp7	-0.579	-0.132	0.710
Layn	-0.547	-0.226	0.773
Lbp	-0.674	-0.284	0.958
Lbr	-0.546	-0.204	0.750
Lclat1	-0.713	-0.118	0.831
Lcp1	-0.720	-0.218	0.939
Ldb2	-0.650	-0.244	0.894
Ldha	-0.559	-0.212	0.771

Ldlr	-0.510	-0.293	0.803
Ldlrad3	-0.621	-0.173	0.794
Leo1	-0.612	-0.083	0.695
Lepr	-0.752	-0.082	0.834
Leprot	-0.672	-0.203	0.876
Lgals3bp	-0.604	-0.283	0.886
Lgals8	-0.649	-0.152	0.801
Lgr4	-0.526	-0.423	0.949
Lig4	-0.486	-0.269	0.755
Lilra5	-0.577	-0.436	1.014
Limch1	-0.559	-0.201	0.760
Limk1	-0.722	-0.041	0.763
Lims1	-0.804	-0.083	0.887
Lims2	-0.712	-0.028	0.741
Lin54	-0.618	-0.178	0.796
Lin7c	-0.612	-0.113	0.726
Lins1	-0.688	-0.024	0.712
Lipa	-0.805	-0.075	0.880
Lipe	-0.764	-0.010	0.774
Lix1l	-0.626	-0.095	0.722
Lman2	-0.598	-0.165	0.763
Lmbrd2	-0.651	-0.272	0.923
Lmcd1	-0.798	0.012	0.786
Lmna	-0.622	-0.210	0.833
Lmo4	-0.575	-0.192	0.767
Lnpep	-0.652	-0.168	0.820
Lnpk1	-0.692	0.047	0.645
Lnx2	-0.670	-0.069	0.740
Loxl1	-0.694	-0.262	0.956
Loxl2	-0.535	-0.185	0.720
Loxl4	-0.747	-0.173	0.920
Lpcat3	-0.662	-0.260	0.922
Lpgat1	-0.681	-0.191	0.872
Lpin2	-0.637	-0.178	0.815
Lpp	-0.610	-0.194	0.804
Lpxn	-0.892	0.091	0.801
Lrfn1	-0.686	-0.019	0.704
Lrif1	-0.730	0.003	0.727
Lrp1	-0.700	-0.205	0.905
Lrp11	-0.726	0.034	0.693
Lrpap1	-0.629	-0.268	0.897
Lrrc25	-0.703	-0.153	0.856
Lrrc28	-0.482	-0.290	0.772
Lrrc32	-0.581	-0.315	0.896
Lrrc40	-0.677	-0.191	0.868
Lrrc41	-0.707	-0.107	0.814
Lrrc57	-0.713	-0.245	0.958
Lrrc58	-0.679	-0.122	0.801
Lrrc8c	-0.734	-0.057	0.791
Lrtm2	-0.608	-0.275	0.883
Lsamp	-0.621	-0.287	0.908
Lsm1	-0.588	-0.289	0.877
Lsm10	-0.685	-0.237	0.922
Lsm12	-0.625	-0.083	0.708
Lsm2	-0.549	-0.195	0.744
Lsm3	-0.561	-0.383	0.943

Lsm6	-0.583	-0.130	0.712
Ltbp1	-0.700	-0.206	0.905
Ltc4s	-0.608	-0.356	0.965
Ltv1	-0.713	-0.238	0.951
Luzp1	-0.657	-0.123	0.779
Ly6g6c	-0.751	-0.162	0.914
Ly86	-0.736	-0.111	0.847
Ly9	-0.848	-0.030	0.878
Lyrm5	-0.845	-0.059	0.904
Lysmd2	-0.575	-0.128	0.703
Lyz2	-0.544	-0.202	0.746
M6pr	-0.572	-0.164	0.736
Macrod2	-0.638	-0.047	0.685
Mad2l2	-0.685	-0.062	0.747
Maea	-0.744	-0.163	0.907
Maf1	-0.572	-0.149	0.721
Mafk	-0.659	-0.171	0.830
Maged2	-0.825	-0.082	0.907
Magee1	-0.641	-0.292	0.933
Magt1	-0.650	-0.097	0.747
Malsu1	-0.668	-0.218	0.886
Maml1	-0.630	-0.163	0.792
Maml2	-0.552	-0.157	0.709
Man2a1	-0.757	-0.121	0.878
Manbal	-0.700	-0.246	0.946
Mansc1	-0.694	-0.248	0.942
Maoa	-0.522	-0.446	0.969
Map2k4	-0.581	-0.269	0.849
Map3k2	-0.614	-0.084	0.698
Map3k7	-0.624	-0.049	0.673
Mapk1	-0.704	-0.190	0.894
Mapk8	-0.624	-0.052	0.676
Mapk9	-0.535	-0.170	0.705
Mapre2	-0.644	-0.194	0.837
March1	-0.876	0.113	0.763
March5	-0.528	-0.222	0.749
Marcks	-0.734	0.005	0.729
Mars	-0.694	-0.060	0.754
Mastl	-0.753	-0.195	0.948
Mat2a	-0.615	-0.162	0.777
Matn2	-0.748	-0.171	0.919
Mbnl1	-0.792	-0.117	0.909
Mbnl2	-0.659	-0.213	0.872
Mbnl3	-0.687	-0.053	0.739
Mbtps2	-0.594	-0.128	0.722
Mcam	-0.760	-0.105	0.866
Mcm3	-0.589	-0.120	0.710
Mcm4	-0.623	-0.339	0.963
Mcmbp	-0.660	-0.071	0.731
Mcts1	-0.708	-0.190	0.898
Mdfic	-0.615	-0.083	0.698
Mdh1	-0.620	-0.283	0.903
Mdh2	-0.668	-0.238	0.906
Me2	-0.568	-0.184	0.753
Med10	-0.804	-0.027	0.831
Med14	-0.646	-0.087	0.733

Med17	-0.816	-0.018	0.834
Med19	-0.536	-0.204	0.740
Med30	-0.679	-0.120	0.799
Med31	-0.847	-0.057	0.904
Med4	-0.620	-0.144	0.764
Med6	-0.585	-0.439	1.024
Med8	-0.688	-0.115	0.803
Med9	-0.670	-0.184	0.854
Med9os	-0.489	-0.401	0.890
Mef2a	-0.725	-0.195	0.920
Memo1	-0.725	-0.099	0.823
Mesdc2	-0.665	-0.091	0.755
Metap2	-0.549	-0.188	0.737
Mettl15	-0.534	-0.212	0.746
Mettl23	-0.569	-0.184	0.753
Mettl4	-0.682	-0.058	0.740
Mettl9	-0.707	-0.275	0.982
Mfap1b	-0.685	-0.269	0.954
Mfap2	-0.851	0.066	0.785
Mfap3	-0.649	-0.051	0.700
Mfap4	-0.763	-0.171	0.934
Mff	-0.566	-0.211	0.778
Mfge8	-0.634	-0.143	0.777
Mfsd1	-0.741	-0.169	0.909
Mfsd10	-0.581	-0.211	0.792
Mfsd14a	-0.564	-0.229	0.792
Mfsd14b	-0.730	-0.112	0.842
Mfsd7b	-0.741	-0.106	0.847
Mfsd8	-0.558	-0.272	0.829
Mgll	-0.693	-0.120	0.813
Mia2_2	-0.581	-0.164	0.745
Mia3	-0.492	-0.253	0.745
Micu2	-0.532	-0.173	0.706
Micu3	-0.602	-0.219	0.821
Mid2	-0.662	-0.292	0.954
Milr1	-0.789	-0.174	0.963
Minos1	-0.546	-0.432	0.978
Mios	-0.592	-0.127	0.719
Mir22hg	-0.670	-0.070	0.741
Mitf	-0.744	-0.049	0.793
Mkks	-0.745	-0.099	0.844
Mkl2	-0.723	-0.135	0.858
Mllt1	-0.580	-0.165	0.745
Mlx	-0.630	-0.208	0.838
Mmadhc	-0.621	-0.237	0.858
Mmd	-0.702	-0.205	0.906
Mmp14	-0.743	-0.051	0.794
Mmrn2	-0.561	-0.240	0.801
Mob1b	-0.685	-0.075	0.760
Mob3a	-0.894	0.015	0.879
Mocs1	-0.575	-0.233	0.808
Mocs2	-0.535	-0.246	0.781
Morf4l2	-0.553	-0.164	0.718
Mospd1	-0.790	-0.073	0.863
Mpc2	-0.602	-0.285	0.887
Mpeg1	-0.737	-0.051	0.788

Mphosph6	-0.490	-0.272	0.762
Mpl	-0.585	-0.359	0.944
Mpp5	-0.583	-0.146	0.729
Mpp6	-0.686	-0.146	0.832
Mpv17	-0.707	-0.082	0.790
Mras	-0.751	0.025	0.726
Mrc2	-0.699	-0.077	0.776
Mrpl13	-0.672	-0.273	0.945
Mrpl2	-0.607	-0.400	1.007
Mrpl23	-0.835	-0.090	0.925
Mrpl3	-0.536	-0.169	0.705
Mrpl33	-0.631	-0.280	0.911
Mrpl34	-0.559	-0.173	0.732
Mrpl35	-0.797	-0.125	0.921
Mrpl40	-0.667	-0.260	0.926
Mrpl46	-0.534	-0.367	0.901
Mrpl47	-0.843	0.004	0.839
Mrpl49	-0.587	-0.145	0.732
Mrpl57	-0.684	-0.191	0.875
Mrps11	-0.797	-0.035	0.832
Mrps12	-0.589	-0.339	0.928
Mrps16	-0.496	-0.387	0.884
Mrps18b	-0.626	-0.287	0.914
Mrps2	-0.535	-0.202	0.737
Mrps21	-0.588	-0.236	0.824
Mrps22	-0.511	-0.261	0.772
Mrps23	-0.635	-0.155	0.790
Mrps24	-0.618	-0.120	0.737
Mrps28	-0.560	-0.207	0.767
Mrps30	-0.859	-0.039	0.898
Mrps31	-0.597	-0.213	0.810
Mrps34	-0.567	-0.237	0.805
Mrps5	-0.542	-0.248	0.791
Mrps7	-0.630	-0.188	0.817
Mrrf	-0.700	-0.225	0.924
Mrto4	-0.594	-0.166	0.760
Ms4a6b	-0.701	-0.100	0.801
Msmo1	-0.537	-0.314	0.851
Msn	-0.668	-0.245	0.913
Msra	-0.645	-0.283	0.928
Msrb1	-0.522	-0.237	0.759
Msrb3	-0.608	-0.155	0.763
Mtap	-0.762	-0.120	0.882
Mtap7d3	-0.534	-0.243	0.777
Mtch2	-0.714	-0.118	0.832
Mthfd1	-0.522	-0.271	0.793
Mthfd2l	-0.632	-0.045	0.677
Mtm1	-0.670	-0.134	0.804
Mtmr1	-0.588	-0.243	0.831
Mtmr10	-0.651	-0.179	0.830
Mtmr2	-0.623	-0.286	0.908
Mtmr9	-0.648	-0.188	0.837
Mtpap	-0.591	-0.211	0.802
Mtpn	-0.749	-0.199	0.948
Mtrr	-0.712	-0.117	0.829
Mum1l1	-0.527	-0.235	0.761

Mustn1	-0.497	-0.266	0.764
Mut	-0.547	-0.261	0.808
Mvd	-0.846	0.076	0.770
Mvp	-0.472	-0.308	0.780
Mxra8	-0.647	-0.153	0.800
Mybbp1a	-0.655	-0.220	0.876
Myc	-0.810	-0.029	0.839
Myd88	-0.557	-0.270	0.827
Mydgf	-0.652	-0.271	0.924
Myh10	-0.645	-0.243	0.889
Myl6	-0.630	-0.350	0.980
Mynn	-0.607	-0.120	0.726
Myo1b	-0.548	-0.163	0.712
Myo1c	-0.531	-0.213	0.745
Myo1d	-0.517	-0.260	0.777
Myof	-0.602	-0.183	0.785
Myzap	-0.654	-0.229	0.883
Mzt1	-0.935	0.160	0.775
N6amt1	-0.771	-0.058	0.829
Naa50	-0.794	-0.142	0.935
Naalad2	-0.968	0.126	0.842
Naca	-0.766	-0.166	0.933
Nacc1	-0.569	-0.172	0.741
Nadk	-0.507	-0.227	0.735
Naip2	-0.665	-0.046	0.711
Nalcn	-0.609	-0.081	0.689
Nans	-0.594	-0.203	0.797
Nap1l1	-0.663	-0.208	0.871
Nars	-0.565	-0.163	0.728
Nat2	-0.724	-0.036	0.760
Natd1	-0.732	-0.101	0.833
Naxd	-0.595	-0.336	0.931
Nbeal1	-0.554	-0.149	0.703
Nbn	-0.588	-0.214	0.803
Nceh1	-0.560	-0.200	0.760
Ncf2	-0.677	0.014	0.663
Nck1	-0.779	-0.133	0.912
Ncoa1	-0.548	-0.226	0.774
Ncoa4	-0.629	-0.345	0.974
Ncoa6	-0.637	-0.111	0.749
Ncstn	-0.584	-0.126	0.710
Ndc1	-0.791	-0.163	0.954
Nde1	-0.669	0.022	0.647
Ndel1	-0.674	-0.100	0.774
Ndfip1	-0.606	-0.189	0.795
Ndfip2	-0.614	-0.139	0.753
Ndrg3	-0.656	-0.092	0.748
Ndufa1	-0.632	-0.373	1.005
Ndufa10	-0.632	-0.240	0.871
Ndufa13	-0.613	-0.282	0.895
Ndufa2	-0.571	-0.326	0.896
Ndufa3	-0.691	-0.093	0.784
Ndufa5	-0.591	-0.196	0.787
Ndufa6	-0.787	-0.180	0.967
Ndufab1	-0.559	-0.171	0.731
Ndufaf1	-0.598	-0.258	0.855

Ndufb10	-0.514	-0.354	0.868
Ndufb2	-0.668	-0.159	0.827
Ndufs3	-0.523	-0.268	0.791
Ndufv2	-0.675	-0.342	1.017
Nebl	-0.541	-0.164	0.705
Necap1	-0.713	-0.055	0.768
Nedd1	-0.586	-0.317	0.903
Nedd9	-0.817	-0.050	0.866
Nek6	-0.685	-0.135	0.820
Nf1	-0.646	-0.109	0.756
Nfam1	-0.680	-0.030	0.710
Nfe2l2	-0.660	-0.276	0.937
Nfia	-0.556	-0.152	0.708
Nfkbia	-0.655	-0.143	0.798
Nfkbib	-0.539	-0.183	0.722
Nfu1	-0.591	-0.308	0.899
Nfyb	-0.794	-0.007	0.801
Nfyc	-0.594	-0.242	0.836
Nhlrc2	-0.535	-0.230	0.765
Nhp2	-0.640	-0.361	1.001
Nhp2l1	-0.540	-0.379	0.919
Nid1	-0.832	-0.106	0.938
Nid2	-0.759	0.031	0.727
Nif3l1	-0.592	-0.234	0.826
Nifk	-0.636	-0.037	0.674
Ninj1	-0.708	-0.226	0.934
Nip7	-0.587	-0.149	0.736
Nipa2	-0.669	-0.076	0.745
Nkain4	-0.617	-0.289	0.906
Nkd1	-0.526	-0.270	0.796
Nkiras2	-0.556	-0.213	0.769
Nkrf	-0.648	-0.155	0.803
Nmd3	-0.718	-0.051	0.769
Nme3	-0.531	-0.198	0.729
Nme6	-0.709	-0.237	0.946
Noc4l	-0.727	-0.004	0.731
Nol3	-0.460	-0.332	0.792
Nol8	-0.738	-0.049	0.787
Nolc1	-0.654	-0.247	0.901
Nop16	-0.854	-0.063	0.917
Nop58	-0.694	-0.049	0.742
Nos3	-0.665	-0.025	0.691
Notch1	-0.608	-0.085	0.693
Nox4	-0.835	-0.102	0.936
Npat	-0.582	-0.176	0.758
Npc1	-0.681	-0.201	0.882
Npl	-0.650	-0.297	0.947
Nptn	-0.741	-0.225	0.966
Nptxr	-0.553	-0.230	0.782
Npy1r	-0.682	-0.180	0.862
Nqo2	-0.592	-0.312	0.904
Nrcam	-0.674	0.018	0.656
Nrg1	-0.660	0.002	0.658
Nrip1	-0.650	-0.063	0.713
Nrip3	-0.684	-0.309	0.992
Nrm	-0.762	0.075	0.688

Nsa2	-0.713	-0.205	0.918
Nsf	-0.628	-0.168	0.796
Nsmce1	-0.580	-0.428	1.008
Nsmce4a	-0.693	-0.141	0.834
Nsun2	-0.576	-0.230	0.806
Nsun3	-0.783	-0.089	0.872
Nt5c3	-0.711	-0.139	0.850
Ntan1	-0.913	0.101	0.811
Ntn1	-0.640	-0.265	0.905
Ntn4	-0.537	-0.204	0.741
Ntrk2	-0.701	0.002	0.699
Nubp2	-0.630	-0.095	0.725
Nubpl	-0.534	-0.177	0.711
Nucb1	-0.695	-0.233	0.928
Nucb2	-0.728	-0.241	0.969
Nucks1	-0.598	-0.157	0.755
Nudcd1	-0.783	-0.067	0.849
Nudt19	-0.624	-0.051	0.674
Nudt21	-0.582	-0.163	0.745
Nudt5	-0.604	-0.110	0.714
Nudt9	-0.543	-0.316	0.859
Numb	-0.653	-0.110	0.763
Nup153	-0.659	-0.134	0.794
Nup155	-0.609	-0.150	0.758
Nup160	-0.682	-0.137	0.819
Nup205	-0.553	-0.235	0.788
Nup35	-0.752	-0.065	0.817
Nup54	-0.646	-0.111	0.757
Nupl1	-0.605	-0.130	0.735
Nus1	-0.687	-0.102	0.789
Nusap1	-0.515	-0.222	0.737
Nxpe5	-0.637	-0.041	0.678
Oas3	-0.836	-0.013	0.850
Oat	-0.529	-0.321	0.850
Ociad1	-0.529	-0.256	0.785
Ogdh	-0.516	-0.213	0.729
Ogfrl1	-0.694	-0.140	0.834
Oip5	-0.841	0.003	0.837
Ola1	-0.673	-0.231	0.904
Olfm2	-0.522	-0.387	0.909
Olfml2b	-0.605	-0.167	0.772
Olfml3	-0.657	-0.274	0.930
Orai1	-0.605	-0.091	0.696
Orc2	-0.571	-0.230	0.801
Orc3	-0.617	-0.052	0.670
Orc5	-0.600	-0.316	0.916
Osbpl2	-0.663	-0.138	0.801
Osbpl8	-0.597	-0.159	0.755
Osgep	-0.684	-0.012	0.696
Otub1	-0.604	-0.189	0.793
Otud6b	-0.772	-0.130	0.902
Oxnad1	-0.615	-0.196	0.812
Oxr1	-0.550	-0.176	0.726
Oxsm	-0.492	-0.330	0.822
Oxsr1	-0.521	-0.202	0.723
P2ry10	-0.730	0.067	0.663

P2ry2	-0.708	0.055	0.653
P3h4	-0.558	-0.251	0.810
P4ha1	-0.642	-0.176	0.818
P4hb	-0.669	-0.257	0.927
Pa2g4	-0.577	-0.242	0.819
Pacsin2	-0.581	-0.242	0.823
Pafah1b2	-0.704	-0.183	0.887
Paics	-0.582	-0.247	0.829
Paip1	-0.558	-0.190	0.748
Pak1ip1	-0.565	-0.263	0.828
Pak4	-0.533	-0.270	0.804
Pak6	-0.744	0.026	0.718
Palld	-0.668	-0.042	0.710
Papd4	-0.480	-0.293	0.773
Papln	-0.902	0.118	0.784
Papola	-0.618	-0.126	0.744
Papolg	-0.721	0.000	0.721
Papss2	-0.585	-0.308	0.893
Pard6g	-0.614	-0.165	0.779
Park7	-0.589	-0.318	0.907
Parp16	-0.756	-0.027	0.783
Parva	-0.534	-0.225	0.759
Parvb	-0.615	-0.134	0.748
Pbdc1	-0.750	0.049	0.701
Pcbp2	-0.761	-0.185	0.946
Pcgf3	-0.714	-0.076	0.790
Pcnp	-0.598	-0.138	0.736
Pcolce	-0.637	-0.217	0.854
Pdcd10	-0.710	-0.111	0.821
Pdcd2	-0.791	-0.148	0.939
Pdcd6ip	-0.565	-0.146	0.711
Pdcl	-0.527	-0.282	0.809
Pdcl3	-0.570	-0.188	0.758
Pde10a	-0.699	0.041	0.658
Pde12	-0.629	-0.150	0.779
Pde1a	-0.764	-0.084	0.848
Pde7b	-0.599	-0.323	0.922
Pde8a	-0.493	-0.276	0.769
Pde9a	-0.586	-0.172	0.758
Pdhx	-0.535	-0.204	0.739
Pdia5	-0.725	0.040	0.685
Pdia6	-0.641	-0.276	0.917
Pdk3	-0.529	-0.383	0.912
Pdlim1	-0.568	-0.186	0.754
Pdlim5	-0.703	-0.046	0.749
Pdzrn3	-0.690	-0.050	0.740
Pea15a	-0.664	-0.186	0.851
Peak1	-0.646	-0.126	0.772
Pebp1	-0.536	-0.225	0.761
Pecam1	-0.735	-0.243	0.977
Pef1	-0.596	-0.316	0.911
Peli2	-0.808	-0.073	0.880
Pex11a	-0.650	-0.119	0.769
Pex13	-0.719	-0.067	0.785
Pex2	-0.699	-0.177	0.876
Pex3	-0.754	-0.043	0.797

Pfdn1	-0.559	-0.148	0.707
Pfdn5	-0.595	-0.264	0.859
Pfdn6	-0.568	-0.309	0.876
Pgam1	-0.726	-0.246	0.972
Pgd	-0.577	-0.232	0.809
Pgf	-0.628	-0.427	1.055
Pgm3	-0.580	-0.139	0.719
Pgrmc1	-0.640	-0.307	0.947
Phax	-0.643	-0.234	0.877
Phc2	-0.617	-0.108	0.725
Phf6	-0.706	0.038	0.668
Phlda1	-0.684	-0.270	0.954
Pigh	-0.664	-0.112	0.776
Pigw	-0.956	0.140	0.815
Pigx	-0.771	-0.096	0.866
Pik3c2a	-0.609	-0.159	0.768
Pik3ca	-0.574	-0.136	0.711
Pik3cg	-0.702	-0.093	0.795
Pilra	-0.694	-0.161	0.855
Pin1	-0.740	-0.093	0.833
Pip4k2c	-0.534	-0.190	0.725
Pip5k1b	-0.497	-0.258	0.755
Pirb	-0.666	0.001	0.666
Pitpna	-0.612	-0.155	0.766
Pitpnb	-0.575	-0.190	0.766
Pitrm1	-0.587	-0.292	0.880
Piwil2	-0.767	-0.057	0.824
Pja2	-0.575	-0.190	0.765
Pkd2	-0.696	-0.121	0.818
Pkhd1l1	-0.762	-0.138	0.899
Pkib	-0.710	-0.029	0.739
Pkm	-0.589	-0.247	0.836
Pla2g12a	-0.582	-0.187	0.769
Plaa	-0.647	-0.170	0.818
Plac8	-0.651	-0.177	0.827
Plac9b	-0.606	-0.242	0.848
Plag1	-0.722	-0.161	0.883
Plbd1	-0.649	-0.156	0.806
Plbd2	-0.602	-0.219	0.821
Plcb4	-0.508	-0.224	0.732
Pld3	-0.575	-0.286	0.861
Plek	-0.721	-0.189	0.910
Plekha3	-0.822	-0.023	0.845
Plekhf2	-0.577	-0.175	0.752
Plet1	-0.648	-0.426	1.074
Plod1	-0.804	-0.082	0.886
Plod2	-0.792	-0.103	0.895
Plod3	-0.598	-0.134	0.733
Plp2	-0.659	-0.233	0.892
Plpp3	-0.537	-0.265	0.802
Plpp5	-0.601	-0.128	0.728
Plscr4	-0.631	-0.210	0.841
Plvap	-0.690	-0.179	0.869
Pmpcb	-0.630	-0.207	0.837
Pnp	-0.710	-0.300	1.010
Pnpla8	-0.669	-0.191	0.861

Pnpt1	-0.689	-0.095	0.784
Pnrc2	-0.720	-0.157	0.877
Poc1a	-0.619	-0.089	0.708
Podxl	-0.724	-0.142	0.867
Pold2	-0.606	-0.309	0.915
Pole2	-0.754	-0.095	0.849
Pole3	-0.619	-0.258	0.877
Polh	-0.560	-0.300	0.860
Polr1a	-0.618	-0.145	0.763
Polr2b	-0.556	-0.251	0.807
Polr2e	-0.603	-0.315	0.919
Polr2f	-0.520	-0.208	0.728
Polr2i	-0.554	-0.311	0.865
Polr2m	-0.567	-0.297	0.864
Polr3f	-0.515	-0.214	0.728
Polr3k	-0.730	-0.062	0.792
Pom121	-0.585	-0.129	0.714
Pomgnt2	-0.772	-0.113	0.884
Pomp	-0.556	-0.280	0.836
Pon2	-0.688	-0.202	0.890
Pon3	-0.546	-0.358	0.904
Pop4	-0.734	-0.227	0.960
Popdc2	-0.546	-0.218	0.764
Pou2af1	-0.896	0.088	0.809
Pou2f1	-0.735	-0.040	0.775
Pparg	-0.600	-0.269	0.869
Ppbp	-0.552	-0.257	0.809
Pphln1	-0.515	-0.239	0.754
Ppil3	-0.568	-0.285	0.854
Ppm1f	-0.657	-0.181	0.838
Ppm1g	-0.608	-0.143	0.750
Ppp1cb	-0.610	-0.159	0.769
Ppp1r14b	-0.795	-0.139	0.933
Ppp1r15a	-0.709	-0.180	0.889
Ppp1r3a	-0.599	-0.171	0.770
Ppp1r7	-0.761	-0.153	0.914
Ppp2cb	-0.660	-0.196	0.855
Ppp2r1a	-0.576	-0.284	0.860
Ppp2r2a	-0.673	-0.064	0.736
Ppp2r2d	-0.633	-0.083	0.716
Ppp2r3a	-0.531	-0.198	0.729
Ppp2r3c	-0.731	-0.079	0.810
Ppp2r5d	-0.661	-0.120	0.781
Ppp3r1	-0.564	-0.209	0.773
Ppp6c	-0.617	-0.242	0.859
Pqbp1	-0.691	-0.167	0.858
Pqlc3	-0.773	-0.161	0.934
Pradc1	-0.755	0.040	0.715
Prcp	-0.838	0.028	0.810
Prdm4	-0.686	-0.101	0.786
Prdx4	-0.717	-0.132	0.849
Prdx5	-0.503	-0.275	0.778
Prdx6	-0.523	-0.253	0.777
Prelid1	-0.595	-0.117	0.711
Prep	-0.552	-0.271	0.823
Prex2	-0.541	-0.186	0.727

Prim1	-0.826	0.112	0.714
Prkaa1	-0.517	-0.267	0.784
Prkab1	-0.558	-0.220	0.777
Prkaca	-0.543	-0.231	0.773
Prkag1	-0.685	-0.187	0.872
Prkar2a	-0.544	-0.187	0.731
Prkca	-0.612	-0.287	0.899
Prkci	-0.597	-0.188	0.785
Prkcsh	-0.580	-0.175	0.755
Prkd3	-0.593	-0.180	0.773
Prkx	-0.673	-0.129	0.803
Prmt5	-0.595	-0.253	0.848
Procr	-0.918	0.065	0.853
Prorsd1	-0.832	-0.037	0.870
Pros1	-0.812	-0.145	0.958
Prpf19	-0.566	-0.234	0.800
Prpf40a	-0.696	-0.201	0.897
Prpf8	-0.736	-0.239	0.975
Prps1	-0.745	-0.235	0.980
Prps2	-0.644	-0.168	0.813
Prr11	-0.495	-0.346	0.841
Prr13	-0.555	-0.470	1.025
Prr15l	-0.569	-0.356	0.925
Prr16	-0.725	0.065	0.659
Prr5	-0.772	0.005	0.767
Prrg1	-0.545	-0.215	0.760
Prrt4	-0.779	-0.195	0.974
Prune2	-0.722	0.019	0.704
Prx	-0.575	-0.150	0.725
Psen1	-0.695	-0.290	0.985
Psenen	-0.510	-0.266	0.777
Psip1	-0.497	-0.243	0.740
Psma3	-0.670	-0.252	0.922
Psma7	-0.620	-0.197	0.817
Psmb4	-0.701	-0.291	0.992
Psmb5	-0.571	-0.415	0.987
Psmb6	-0.600	-0.394	0.994
Psmb8	-0.565	-0.260	0.825
Psmc1	-0.670	-0.282	0.952
Psmc3	-0.604	-0.277	0.881
Psmc4	-0.643	-0.287	0.930
Psmc5	-0.590	-0.277	0.867
Psmc6	-0.707	-0.228	0.935
Psmd1	-0.564	-0.215	0.779
Psmd11	-0.653	-0.126	0.780
Psmd14	-0.696	-0.317	1.014
Psmd2	-0.518	-0.202	0.720
Psmd6	-0.577	-0.144	0.720
Psmd7	-0.536	-0.208	0.743
Psmd8	-0.537	-0.303	0.840
Psme2	-0.610	-0.389	0.999
Psme4	-0.579	-0.139	0.718
Psmg2	-0.583	-0.199	0.782
Psph	-0.602	-0.136	0.738
Pstk	-0.739	0.002	0.737
Pstpip2	-0.498	-0.279	0.776

Ptar1	-0.644	-0.204	0.848
Ptbp1	-0.666	-0.285	0.950
Ptbp3	-0.754	-0.113	0.867
Pten	-0.610	-0.143	0.752
Pter	-0.693	-0.256	0.949
Ptgdr2	-1.060	0.222	0.838
Ptges3	-0.656	-0.197	0.853
Ptgis	-0.654	-0.371	1.026
Ptk7	-0.708	-0.119	0.827
Ptp4a1	-0.769	-0.166	0.935
Ptp4a2	-0.583	-0.150	0.733
Ptpn12	-0.727	-0.129	0.856
Ptpn2	-0.589	-0.147	0.736
Ptpra	-0.649	-0.235	0.885
Ptprg	-0.598	-0.231	0.829
Ptprj	-0.760	-0.139	0.899
Ptprk	-0.542	-0.222	0.764
Pttg1ip	-0.609	-0.067	0.676
Puf60	-0.660	-0.198	0.858
Pum2	-0.719	-0.153	0.872
Purb	-0.689	-0.055	0.744
Pus1	-0.653	-0.034	0.686
Pus7l	-0.529	-0.279	0.808
Pvr	-0.696	-0.034	0.730
Pwp1	-0.515	-0.359	0.874
Pxdc1	-0.761	-0.076	0.837
Pxdn	-0.655	-0.227	0.882
Pycard	-0.650	-0.167	0.817
Pycr1	-0.592	-0.255	0.847
Pyroxd1	-0.537	-0.213	0.750
Pyurf	-0.877	0.052	0.825
Qk	-0.737	-0.157	0.893
Qser1	-0.646	-0.085	0.731
Qsox1	-0.682	-0.109	0.791
Qsox2	-0.663	-0.293	0.956
Rab12	-0.740	-0.207	0.947
Rab14	-0.541	-0.174	0.715
Rab18	-0.782	-0.173	0.955
Rab1a	-0.835	-0.065	0.901
Rab21	-0.643	-0.207	0.850
Rab27a	-0.631	-0.175	0.807
Rab29	-0.589	-0.151	0.740
Rab2a	-0.713	-0.175	0.888
Rab32	-0.861	-0.037	0.897
Rab33b	-0.593	-0.180	0.773
Rab35	-0.692	-0.150	0.841
Rab3c	-0.674	-0.295	0.969
Rab4a	-0.752	0.054	0.698
Rab5a	-0.761	-0.206	0.967
Rab5b	-0.551	-0.233	0.784
Rab8a	-0.631	-0.194	0.825
Rab8b	-0.759	-0.083	0.843
Rab9	-0.551	-0.239	0.789
Rabac1	-0.540	-0.344	0.884
Rabepk	-0.620	-0.314	0.935
Rabif	-0.728	-0.117	0.846

Rabl3	-0.664	-0.224	0.889
Rac2	-0.790	-0.030	0.820
Rack1	-0.631	-0.065	0.696
Rad1	-0.692	0.042	0.650
Rad17	-0.660	-0.162	0.822
Rad18	-0.521	-0.243	0.763
Rad23b	-0.677	-0.188	0.865
Rai2	-0.535	-0.295	0.829
Rala	-0.641	-0.159	0.800
Ralgapb	-0.573	-0.140	0.712
Ran	-0.631	-0.190	0.821
Ranbp2	-0.594	-0.118	0.712
Ranbp9	-0.657	0.008	0.649
Rap1gds1	-0.724	-0.125	0.850
Rap2b	-0.851	0.040	0.811
Rapgef5	-0.724	-0.086	0.810
Raph1	-0.737	-0.056	0.793
Rars	-0.687	-0.210	0.896
Rasl10b	-0.712	-0.176	0.889
Rassf1	-0.624	-0.078	0.702
Rbbp4	-0.622	-0.093	0.715
Rbbp9	-0.765	-0.201	0.966
Rbm18	-0.592	-0.156	0.748
Rbm3	-0.550	-0.205	0.755
Rbm42	-0.678	-0.191	0.869
Rbm48	-0.723	-0.029	0.752
Rbm4b	-0.562	-0.367	0.929
Rbm7	-0.629	-0.267	0.896
Rbms1	-0.608	-0.088	0.696
Rbms2	-0.578	-0.223	0.800
Rbmxl1	-0.621	-0.285	0.905
Rbp1	-0.693	-0.109	0.802
Rc3h1	-0.634	-0.052	0.686
Rc3h2	-0.709	-0.046	0.755
Rcn1	-0.740	-0.211	0.951
Rcn2	-0.661	-0.196	0.857
Rcn3	-0.699	-0.282	0.982
Rdh11	-0.720	-0.194	0.914
Rdh12	-0.649	-0.424	1.073
Rdx	-0.521	-0.212	0.733
Reck	-0.657	-0.113	0.770
Reep1	-0.684	-0.101	0.785
Reep5	-0.708	-0.156	0.864
Rell1	-0.640	-0.092	0.732
Rer1	-0.572	-0.168	0.740
Rerg	-0.713	-0.016	0.729
Rexo2	-0.590	-0.178	0.768
Rfc4	-0.548	-0.279	0.826
Rfesd	-0.464	-0.338	0.802
Rfk	-0.558	-0.255	0.813
Rgmb	-0.608	-0.213	0.821
Rhbdl1	-0.786	0.075	0.711
Rhoa	-0.578	-0.288	0.866
Rhob	-0.732	-0.110	0.841
Rictor	-0.582	-0.121	0.703
Rida	-0.562	-0.208	0.770

Rin2	-0.763	-0.112	0.875
Rin3	-0.717	0.041	0.676
Ripk1	-0.702	-0.096	0.798
Ripk2	-0.455	-0.352	0.807
Ripk3	-0.598	-0.129	0.728
Rmdn2	-0.550	-0.465	1.015
Rnd3	-0.655	-0.106	0.761
Rnf128	-0.563	-0.166	0.729
Rnf138	-0.603	-0.263	0.866
Rnf141	-0.609	-0.209	0.817
Rnf144b	-0.542	-0.244	0.785
Rnf2	-0.936	0.078	0.858
Rnf217	-0.609	-0.112	0.721
Rnf219	-0.681	0.009	0.673
Rnf34	-0.606	-0.102	0.708
Rnf7	-0.517	-0.206	0.723
Rnft1	-0.701	-0.106	0.807
Ror1	-0.811	-0.082	0.893
Rora	-0.585	-0.213	0.798
Rp2	-0.714	-0.021	0.735
RP23-386F15.2	-0.458	-0.366	0.824
Rpe	-0.706	-0.127	0.833
Rpf1	-0.564	-0.241	0.805
Rpf2	-0.554	-0.227	0.781
Rpl10a	-0.833	-0.024	0.857
Rpl11	-0.680	-0.264	0.944
Rpl12	-0.816	-0.119	0.934
Rpl13a	-0.661	-0.118	0.779
Rpl15	-0.771	-0.168	0.939
Rpl18	-0.716	-0.109	0.825
Rpl19	-0.784	-0.136	0.920
Rpl22	-0.632	-0.129	0.761
Rpl27	-0.587	-0.249	0.835
Rpl3	-0.558	-0.146	0.703
Rpl30	-0.636	-0.249	0.885
Rpl36a	-0.718	-0.176	0.894
Rpl37a	-0.555	-0.286	0.841
Rpl4	-0.590	-0.143	0.734
Rpl41	-0.648	-0.227	0.875
Rpl6	-0.733	-0.212	0.945
Rpl7	-0.714	-0.162	0.876
Rpl8	-0.649	-0.165	0.814
Rpl9	-0.577	-0.142	0.718
Rplp0	-0.697	-0.186	0.883
Rplp1	-0.573	-0.172	0.745
Rpn1	-0.675	-0.161	0.836
Rpn2	-0.715	-0.261	0.976
Rpp14	-0.649	-0.079	0.728
Rpp38	-0.687	-0.169	0.856
Rpp40	-0.709	-0.260	0.969
Rps10	-0.634	-0.233	0.867
Rps14	-0.748	-0.116	0.864
Rps19bp1	-0.621	-0.379	1.000
Rps20	-0.691	-0.257	0.948
Rps24	-0.606	-0.158	0.764
Rps27a	-0.646	-0.120	0.766

Rps4x	-0.692	-0.166	0.858
Rps5	-0.702	-0.106	0.808
Rps6	-0.638	-0.260	0.898
Rps6ka3	-0.702	-0.224	0.926
Rps7	-0.817	-0.139	0.957
Rps8	-0.686	-0.247	0.933
Rpsa	-0.694	-0.213	0.907
Rraga	-0.733	-0.235	0.967
Rragc	-0.614	-0.185	0.798
Rras	-0.628	-0.254	0.882
Rrbp1	-0.650	-0.130	0.780
Rrm1	-0.635	-0.203	0.838
Rrm2b	-0.702	-0.091	0.792
Rrn3	-0.694	-0.212	0.906
Rrp15	-0.512	-0.278	0.790
Rrp8	-0.634	-0.196	0.830
Rrs1	-0.601	-0.144	0.745
Rsbn1l	-0.601	-0.127	0.728
Rsl1d1	-0.784	-0.075	0.859
Rsu1	-0.554	-0.181	0.736
Rtca	-0.644	-0.233	0.877
Rtn1	-0.691	-0.066	0.758
Rtn4	-0.673	-0.110	0.783
Rtp4	-0.691	-0.057	0.749
Rundc3b	-0.668	-0.116	0.784
Ruvbl2	-0.598	-0.237	0.835
Rwdd1	-0.538	-0.177	0.715
Rybp	-0.672	-0.171	0.843
S100a1	-0.535	-0.302	0.837
S100a13	-0.534	-0.234	0.768
S1pr2	-0.558	-0.162	0.720
Samd4b	-0.712	-0.132	0.844
Samd8	-0.650	-0.148	0.798
Samsn1	-0.732	-0.073	0.805
Sar1a	-0.636	-0.161	0.796
Sar1b	-0.682	-0.306	0.988
Sars	-0.580	-0.136	0.715
Sav1	-0.553	-0.177	0.731
Sbds	-0.586	-0.178	0.764
Sbf2	-0.634	-0.194	0.828
Sc5d	-0.477	-0.350	0.827
Scamp1	-0.550	-0.155	0.704
Scamp3	-0.665	-0.033	0.697
Scarb2	-0.686	-0.209	0.895
Sccpdh	-0.498	-0.241	0.739
Scfd1	-0.681	-0.266	0.947
Scnn1a	-0.698	-0.311	1.009
Scnn1b	-0.559	-0.305	0.864
Sco1	-0.669	-0.066	0.736
Scoc	-0.798	0.017	0.781
Scp2	-0.526	-0.304	0.831
Scube1	-0.726	-0.037	0.763
Scube2	-0.593	-0.130	0.723
Sdc3	-0.741	-0.147	0.888
Sdc4	-0.615	-0.198	0.813
Sdf2	-0.657	-0.071	0.727

Sdha	-0.553	-0.286	0.839
Sdhaf1	-0.785	-0.173	0.958
Sdhaf2	-0.645	-0.086	0.731
Sdhb	-0.696	-0.276	0.972
Sdhd	-0.559	-0.175	0.733
Sdk2	-0.519	-0.239	0.758
Sec11c	-0.606	-0.096	0.701
Sec22b	-0.503	-0.236	0.738
Sec23a	-0.659	-0.228	0.887
Sec23b	-0.551	-0.319	0.870
Sec23ip	-0.608	-0.199	0.807
Sec24a	-0.716	-0.018	0.733
Sec24c	-0.616	-0.104	0.720
Sec31a	-0.658	-0.164	0.822
Sec61a1	-0.748	-0.182	0.930
Sec61a2	-0.678	0.037	0.641
Sec62	-0.706	-0.241	0.947
Sec63	-0.740	-0.165	0.904
Secisbp2l	-0.577	-0.207	0.784
Sectm1b	-0.687	-0.339	1.026
Seh1l	-0.676	-0.126	0.803
Selenoi	-0.689	-0.028	0.717
Selenok	-0.794	-0.179	0.973
Selenon	-0.644	-0.136	0.780
Sell	-0.680	-0.181	0.862
Selplg	-0.771	-0.058	0.829
Sema3a	-0.571	-0.266	0.837
Sema3d	-0.764	-0.085	0.849
Sema3f	-0.733	-0.143	0.876
Sema3g	-0.645	-0.217	0.862
Sema4c	-0.636	-0.031	0.667
Sephs1	-0.567	-0.246	0.814
Sept10	-0.759	-0.181	0.940
Sept11	-0.621	-0.110	0.731
Sept2	-0.707	-0.196	0.903
Serbp1	-0.655	-0.199	0.855
Serf2	-0.692	-0.293	0.984
Serinc2	-0.609	-0.134	0.743
Serp1	-0.652	-0.110	0.762
Serpina3h	-0.667	-0.043	0.710
Serpina3i	-0.658	-0.115	0.773
Serpinb6b	-0.681	-0.217	0.898
Serpinb9	-0.573	-0.247	0.820
Serpine1	-0.887	0.039	0.848
Serpine2	-0.701	0.052	0.649
Serpinh1	-0.769	-0.147	0.916
Sertad1	-0.577	-0.186	0.764
Sertad2	-0.607	-0.192	0.799
Sertad3	-0.718	-0.277	0.994
Set	-0.596	-0.134	0.731
Sf3a2	-0.792	-0.107	0.899
Sf3a3	-0.650	-0.253	0.903
Sfr1	-0.634	-0.163	0.797
Sftpb	-0.508	-0.387	0.895
Sgcb	-0.674	-0.202	0.876
Sgce	-0.693	-0.243	0.936

Sgf29	-0.714	-0.034	0.748
Sgms1	-0.702	-0.106	0.808
Sgms2	-0.558	-0.265	0.823
Sgpl1	-0.636	-0.082	0.718
Sh2d1a	-0.575	-0.249	0.825
Sh2d1b1	-0.921	0.122	0.799
Sh2d3c	-0.709	-0.059	0.768
Sh3pxd2a	-0.573	-0.176	0.749
Sh3pxd2b	-0.820	0.056	0.764
Shc4	-0.735	-0.051	0.786
Shmt2	-0.767	-0.170	0.937
Shoc2	-0.639	-0.135	0.774
Shroom2	-0.756	-0.059	0.815
Siae	-0.672	-0.115	0.788
Siah1a	-0.627	-0.086	0.713
Siah1b	-0.566	-0.357	0.923
Siglec1	-0.815	-0.097	0.912
Sigmar1	-0.603	-0.352	0.955
Sik1	-0.682	-0.127	0.809
Sirpb1a	-0.597	-0.337	0.933
Siva1	-0.486	-0.297	0.783
Ska2	-0.585	-0.232	0.817
Ska3	-0.826	0.104	0.722
Skil	-0.728	-0.059	0.788
Skiv2l2	-0.584	-0.197	0.781
Skp1a	-0.620	-0.415	1.035
Slain2	-0.613	-0.101	0.714
Slc11a1	-0.522	-0.380	0.903
Slc12a2	-0.554	-0.176	0.730
Slc12a8	-0.828	0.034	0.794
Slc15a2	-0.631	-0.074	0.705
Slc15a3	-0.641	-0.066	0.707
Slc16a1	-0.650	-0.257	0.907
Slc16a2	-0.698	-0.138	0.836
Slc16a3	-0.551	-0.254	0.806
Slc16a9	-0.529	-0.275	0.804
Slc19a2	-0.558	-0.158	0.716
Slc25a12	-0.467	-0.377	0.844
Slc25a17	-0.548	-0.183	0.731
Slc25a20	-0.793	-0.113	0.905
Slc25a24	-0.760	-0.166	0.926
Slc25a4	-0.605	-0.234	0.839
Slc25a43	-0.489	-0.281	0.770
Slc25a46	-0.695	-0.265	0.960
Slc25a51	-0.647	-0.162	0.810
Slc29a2	-0.829	0.009	0.820
Slc29a3	-0.607	-0.106	0.714
Slc2a1	-0.772	0.011	0.761
Slc2a10	-0.677	-0.208	0.886
Slc2a12	-0.540	-0.173	0.713
Slc2a13	-0.556	-0.216	0.772
Slc30a1	-0.695	-0.224	0.919
Slc30a5	-0.539	-0.197	0.736
Slc30a6	-0.685	-0.289	0.974
Slc35a3	-0.754	-0.073	0.827
Slc35a4	-0.555	-0.298	0.853

Slc35a5	-0.801	-0.105	0.907
Slc35b1	-0.673	-0.065	0.738
Slc35c1	-0.639	-0.065	0.704
Slc35e1	-0.560	-0.202	0.763
Slc35f5	-0.775	-0.149	0.923
Slc35f6	-0.692	-0.220	0.912
Slc36a4	-0.677	-0.110	0.787
Slc37a2	-0.659	-0.115	0.774
Slc38a2	-0.752	-0.120	0.872
Slc38a5	-0.586	-0.205	0.791
Slc39a1	-0.635	-0.156	0.791
Slc39a6	-0.660	-0.122	0.782
Slc39a7	-0.503	-0.231	0.734
Slc39a8	-0.526	-0.403	0.929
Slc3a2	-0.696	-0.243	0.939
Slc41a2	-0.748	-0.204	0.953
Slc43a3	-0.814	-0.150	0.964
Slc44a1	-0.584	-0.217	0.801
Slc4a11	-0.538	-0.238	0.775
Slc6a2	-0.712	-0.128	0.840
Slc6a9	-0.534	-0.245	0.779
Slc7a1	-0.785	-0.108	0.893
Slc7a11	-0.560	-0.341	0.901
Slc7a6	-0.745	0.053	0.692
Slc9a6	-0.629	-0.222	0.851
Slco2a1	-0.808	-0.149	0.957
Slco2b1	-0.640	-0.308	0.948
Slco4c1	-0.545	-0.223	0.769
Slfn5	-0.726	-0.045	0.770
Slit3	-0.696	-0.268	0.965
Slmo2	-0.658	-0.110	0.767
Smap1	-0.804	-0.065	0.869
Smarca5	-0.709	-0.106	0.815
Smarce1	-0.734	-0.059	0.793
Smg8	-0.625	-0.323	0.949
Smim10l1	-0.614	-0.146	0.761
Smim11	-0.496	-0.349	0.845
Smim13	-0.786	-0.039	0.825
Smim14	-0.608	-0.138	0.746
Smim15	-0.835	-0.082	0.917
Smim20	-0.607	-0.220	0.827
Smim7	-0.587	-0.131	0.718
Smn1	-0.559	-0.177	0.737
Smndc1	-0.545	-0.217	0.762
Smoc2	-0.690	-0.281	0.972
Smtnl2	-0.770	-0.153	0.923
Smu1	-0.564	-0.191	0.754
Smurf2	-0.625	-0.193	0.818
Smyd5	-0.555	-0.156	0.711
Snai1	-0.715	-0.250	0.965
Snap23	-0.702	-0.146	0.848
Snap29	-0.764	-0.128	0.892
Snap47	-0.660	-0.178	0.838
Snapin	-0.567	-0.188	0.755
Snd1	-0.608	-0.122	0.731
Snf8	-0.589	-0.151	0.740

Snrnp200	-0.557	-0.216	0.772
Snrnp27	-0.658	-0.186	0.844
Snrpb	-0.603	-0.220	0.823
Snrpc	-0.678	-0.199	0.876
Snrpd1	-0.798	-0.176	0.974
Snrpe	-0.719	-0.059	0.779
Snta1	-0.550	-0.159	0.709
Sntg2	-0.520	-0.201	0.720
Snx1	-0.667	-0.154	0.821
Snx13	-0.546	-0.201	0.747
Snx16	-0.806	0.112	0.694
Snx2	-0.690	-0.218	0.908
Snx3	-0.629	-0.177	0.807
Snx5	-0.638	-0.009	0.647
Snx6	-0.751	-0.132	0.883
Snx7	-0.528	-0.308	0.835
Snx9	-0.727	-0.084	0.811
Sod1	-0.507	-0.408	0.915
Sod2	-0.634	-0.406	1.040
Sod3	-0.652	-0.268	0.920
Sorbs3	-0.737	-0.136	0.873
Sos2	-0.644	-0.105	0.749
Sox18	-0.498	-0.241	0.739
Sp1	-0.700	-0.170	0.870
Sp4	-0.498	-0.263	0.761
Spag7	-0.562	-0.241	0.803
Spcs2	-0.627	-0.278	0.905
Spcs3	-0.625	-0.115	0.739
Speer4c	-0.806	0.073	0.732
Spg21	-0.639	-0.282	0.922
Sphkap	-0.547	-0.180	0.727
Spi1	-0.710	-0.110	0.820
Spidr	-0.573	-0.222	0.795
Spire1	-0.536	-0.180	0.715
Spon1	-0.789	-0.127	0.916
Sppl2a	-0.700	-0.235	0.935
Spred1	-0.670	-0.043	0.713
Spry2	-0.652	-0.170	0.821
Spry4	-0.619	-0.117	0.737
Spryd4	-0.726	-0.210	0.936
Spryd7	-0.619	-0.417	1.036
Sptlc2	-0.610	-0.135	0.744
Spty2d1	-0.584	-0.183	0.768
Sqstm1	-0.580	-0.262	0.841
Srek1ip1	-0.518	-0.210	0.728
Sri	-0.622	-0.219	0.841
Srp14	-0.523	-0.318	0.841
Srp19	-0.606	-0.137	0.744
Srp54b	-0.602	-0.115	0.718
Srp68	-0.561	-0.226	0.787
Srp72	-0.644	-0.232	0.877
Srpr	-0.620	-0.297	0.917
Srprb	-0.745	-0.116	0.861
Srxn1	-0.603	-0.324	0.927
Ss18	-0.687	-0.111	0.798
Ssb	-0.679	-0.126	0.805

Ssbp2	-0.567	-0.160	0.727
Ssh2	-0.682	-0.072	0.754
Ssr1	-0.658	-0.193	0.851
Ssr2	-0.671	-0.213	0.884
Ssr3	-0.732	-0.175	0.907
Ssrp1	-0.582	-0.175	0.757
Ssu72	-0.612	-0.103	0.715
St13	-0.784	-0.117	0.901
St7	-0.706	0.021	0.685
Stag2	-0.588	-0.123	0.712
Stat3	-0.692	-0.119	0.811
Stbd1	-0.610	-0.119	0.729
Steap1	-0.687	-0.150	0.838
Steap2	-0.863	-0.047	0.910
Steap4	-0.458	-0.380	0.838
Stip1	-0.726	-0.237	0.963
Stk17b	-0.681	0.045	0.636
Stk24	-0.601	-0.181	0.782
Stk32a	-0.484	-0.406	0.890
Stmn2	-0.694	-0.195	0.889
Stom	-0.698	-0.116	0.814
Stoml2	-0.788	-0.073	0.861
Strap	-0.617	-0.278	0.895
Strn	-0.596	-0.152	0.748
Strn3	-0.641	-0.144	0.785
Stx12	-0.705	-0.107	0.812
Stx18	-0.676	-0.126	0.802
Stx7	-0.769	-0.112	0.882
Sub1	-0.562	-0.304	0.866
Sucla2	-0.577	-0.209	0.786
Suclg1	-0.595	-0.297	0.892
Sugt1	-0.545	-0.285	0.829
Sulf1	-0.789	0.007	0.782
Sulf2	-0.673	-0.042	0.715
Sumf2	-0.700	-0.022	0.723
Sumo1	-0.616	-0.142	0.758
Sumo2	-0.567	-0.376	0.942
Suox	-0.674	-0.156	0.830
Surf4	-0.607	-0.203	0.810
Svbp	-0.672	-0.069	0.741
Svip	-0.543	-0.324	0.867
Swi5	-0.605	-0.261	0.867
Swt1	-0.626	-0.060	0.686
Syf2	-0.607	-0.162	0.770
Syn3	-0.552	-0.280	0.832
Syngr2	-0.795	-0.154	0.949
Synj2bp	-0.647	-0.233	0.880
Sypl	-0.619	-0.285	0.904
Tab3	-0.612	-0.114	0.727
Tacc1	-0.663	-0.139	0.802
Tada1	-0.659	0.017	0.642
Tada2b	-0.608	-0.189	0.797
Taf10	-0.510	-0.286	0.795
Taf5	-0.812	-0.046	0.857
Taf7	-0.734	-0.011	0.745
Taldo1	-0.682	-0.289	0.971

Tank	-0.681	-0.039	0.720
Tars	-0.619	-0.210	0.829
Tax1bp1	-0.619	-0.253	0.872
Tbc1d15	-0.707	-0.243	0.949
Tbcb	-0.584	-0.295	0.879
Tbl1x	-0.507	-0.273	0.780
Tbrg1	-0.678	-0.157	0.835
Tbx3	-0.557	-0.193	0.750
Tbx4	-0.646	-0.267	0.913
Tbx5	-0.617	-0.224	0.841
Tc2n	-0.474	-0.339	0.814
Tcea1	-0.687	-0.139	0.826
Tceal8	-0.603	-0.163	0.765
Tceb3	-0.757	-0.170	0.928
Tcf21	-0.593	-0.301	0.895
Tcp1	-0.638	-0.169	0.807
Tcta	-0.511	-0.271	0.782
Tek	-0.611	-0.332	0.943
Tex10	-0.626	-0.132	0.758
Tfb2m	-0.591	-0.114	0.705
Tfpi	-0.713	-0.119	0.832
Tgfa	-0.750	-0.123	0.873
Tgfb1	-0.800	-0.102	0.902
Tgfb1i1	-0.582	-0.145	0.728
Tgfbr1	-0.775	0.035	0.740
Tgfbr2	-0.664	-0.333	0.997
Tgfbr3	-0.613	-0.264	0.877
Tgm1	-0.590	-0.228	0.818
Thap7	-0.463	-0.390	0.852
Thbd	-0.755	-0.147	0.901
Thoc1	-0.542	-0.201	0.742
Thoc7	-0.500	-0.239	0.738
Thyn1	-0.593	-0.151	0.744
Timm23	-0.567	-0.226	0.793
Timm50	-0.677	-0.174	0.851
Timm9	-0.794	-0.038	0.832
Timp2	-0.587	-0.191	0.778
Tinag	-0.816	0.120	0.696
Tipin	-0.497	-0.377	0.874
Tln1	-0.674	-0.197	0.870
Tlr2	-0.485	-0.340	0.825
Tlr3	-0.622	-0.334	0.956
Tm2d2	-0.508	-0.259	0.766
Tm4sf19	-0.756	-0.177	0.934
Tm7sf3	-0.646	-0.190	0.836
Tm9sf3	-0.623	-0.210	0.833
Tm9sf4	-0.598	-0.106	0.704
Tma16	-0.585	-0.393	0.979
Tmbim4	-0.591	-0.268	0.859
Tmc3	-0.796	0.006	0.790
Tmc7	-0.990	0.179	0.812
Tmed2	-0.669	-0.227	0.896
Tmed3	-0.680	-0.343	1.022
Tmed9	-0.681	-0.188	0.869
Tmem104	-0.562	-0.202	0.764
Tmem119	-0.578	-0.283	0.861

Tmem120a	-0.652	-0.360	1.012
Tmem123	-0.825	-0.117	0.941
Tmem128	-0.696	-0.169	0.865
Tmem131	-0.592	-0.141	0.733
Tmem138	-0.783	0.068	0.715
Tmem144	-0.570	-0.152	0.722
Tmem147	-0.474	-0.384	0.858
Tmem14c	-0.630	-0.182	0.812
Tmem154	-0.666	-0.257	0.923
Tmem165	-0.570	-0.154	0.725
Tmem168	-0.732	-0.080	0.811
Tmem170b	-0.677	0.014	0.663
Tmem173	-0.713	-0.060	0.772
Tmem176a	-0.591	-0.200	0.791
Tmem176b	-0.622	-0.173	0.794
Tmem184b	-0.627	-0.181	0.808
Tmem185a	-0.554	-0.353	0.907
Tmem185b	-0.808	0.017	0.791
Tmem189	-0.827	-0.016	0.843
Tmem192	-0.723	-0.205	0.927
Tmem205	-0.483	-0.327	0.810
Tmem208	-0.662	-0.040	0.702
Tmem209	-0.777	-0.142	0.919
Tmem230	-0.515	-0.339	0.854
Tmem234	-0.652	-0.172	0.824
Tmem263	-0.748	-0.113	0.861
Tmem30a	-0.740	-0.148	0.888
Tmem38b	-0.644	-0.043	0.687
Tmem41b	-0.685	-0.198	0.883
Tmem47	-0.781	-0.152	0.933
Tmem5	-0.713	-0.112	0.826
Tmem50a	-0.679	-0.201	0.879
Tmem59	-0.606	-0.208	0.814
Tmem60	-0.565	-0.295	0.860
Tmem62	-0.537	-0.316	0.853
Tmem65	-0.630	-0.140	0.771
Tmem68	-0.836	-0.060	0.895
Tmem69	-0.830	0.038	0.792
Tmem87b	-0.623	-0.048	0.671
Tmem9b	-0.596	-0.179	0.775
Tmod2	-0.468	-0.388	0.856
Tmod3	-0.673	-0.172	0.845
Tmsb10	-0.612	-0.139	0.751
Tmsb4x	-0.623	-0.085	0.707
Tmtc3	-0.787	-0.168	0.955
Tmub1	-0.596	-0.127	0.723
Tmx1	-0.811	-0.072	0.883
Tmx3	-0.743	-0.127	0.870
Tnfaip6	-0.521	-0.425	0.946
Tnfaip8l2	-0.728	-0.105	0.834
Tnfrsf18	-0.641	-0.201	0.842
Tnfrsf1a	-0.647	-0.216	0.864
Tnfrsf9	-0.973	0.186	0.787
Tnfsf13b	-0.512	-0.340	0.852
Tnks2	-0.531	-0.197	0.729
Tnn	-0.613	-0.103	0.716

Tnnc1	-0.529	-0.226	0.755
Tnpo1	-0.728	-0.104	0.832
Tns1	-0.538	-0.229	0.766
Tollip	-0.615	-0.140	0.754
Tomm20	-0.604	-0.213	0.817
Tomm70a	-0.724	-0.131	0.855
Tor1aip1	-0.562	-0.201	0.763
Tox	-0.760	0.091	0.669
Tox2	-0.820	0.019	0.802
Tpbg	-0.578	-0.209	0.787
Tpbgl	-0.597	-0.411	1.009
Tpd52	-0.657	-0.144	0.801
Tpgs2	-0.669	-0.162	0.831
Tpi1	-0.710	-0.230	0.940
Tpp1	-0.672	-0.178	0.850
Tpp2	-0.654	-0.190	0.844
Tprkb	-0.558	-0.254	0.812
Tpt1	-0.740	-0.166	0.905
Trak2	-0.683	-0.143	0.825
Trappc13	-0.655	-0.163	0.818
Trappc2	-0.757	-0.197	0.955
Trappc6b	-0.678	-0.053	0.731
Trappc8	-0.807	-0.124	0.931
Trbc1	-0.869	0.078	0.791
Triap1	-0.497	-0.322	0.819
Trib2	-0.654	-0.100	0.754
Trim16	-0.677	-0.134	0.811
Trim23	-0.620	-0.142	0.762
Trim30c	-0.656	-0.138	0.794
Trim32	-0.895	0.017	0.879
Trip12	-0.610	-0.141	0.751
Trmt10a	-0.644	-0.118	0.762
Trmt10c	-0.544	-0.241	0.785
Trmt112	-0.542	-0.173	0.715
Trnt1	-0.618	-0.063	0.681
Tro	-0.766	-0.164	0.930
Trove2	-0.637	-0.129	0.766
Trp53	-0.640	-0.044	0.684
Trp53i11	-0.716	-0.147	0.864
Trp53inp1	-0.754	-0.095	0.849
Tsg101	-0.578	-0.140	0.718
Tshz3	-0.782	-0.045	0.827
Tsku	-0.691	-0.242	0.933
Tspan2	-0.688	-0.269	0.957
Tspan3	-0.521	-0.211	0.732
Tspan31	-0.551	-0.220	0.771
Tspan4	-0.675	-0.181	0.857
Tspan8	-0.675	-0.318	0.993
Tspan9	-0.564	-0.286	0.850
Tsr1	-0.629	-0.074	0.702
Tsta3	-0.682	-0.234	0.916
Ttc17	-0.677	-0.090	0.767
Ttc27	-0.715	-0.103	0.819
Ttc33	-0.630	-0.176	0.806
Ttc9c	-0.602	-0.179	0.782
Ttpal	-0.782	-0.012	0.794

Ttyh2	-0.587	-0.159	0.747
Tuba1c	-0.688	-0.283	0.971
Tuba4a	-0.688	-0.205	0.892
Tubb4a	-0.672	-0.278	0.949
Tubb4b	-0.595	-0.380	0.975
Tufm	-0.574	-0.217	0.791
Tug1	-0.660	-0.098	0.758
Tusc2	-0.672	-0.116	0.788
Tusc3	-0.494	-0.314	0.808
Twf1	-0.709	-0.280	0.989
Txn1	-0.490	-0.357	0.847
Txndc11	-0.560	-0.155	0.715
Txndc12	-0.600	-0.192	0.792
Txndc5	-0.539	-0.269	0.808
Txnl1	-0.729	-0.214	0.944
Txnrd1	-0.608	-0.214	0.822
Tyms	-0.614	-0.075	0.689
Tyrobp	-0.740	-0.089	0.829
U2af1l4_1	-0.484	-0.271	0.755
Uba1	-0.767	-0.179	0.946
Uba2	-0.540	-0.163	0.702
Uba5	-0.521	-0.220	0.741
Uba6	-0.682	-0.145	0.828
Ubac2	-0.588	-0.194	0.782
Ubald2	-0.735	-0.128	0.863
Ubap2	-0.543	-0.170	0.712
Ubap2l	-0.618	-0.147	0.766
Ube2a	-0.664	-0.172	0.836
Ube2b	-0.546	-0.207	0.753
Ube2cbp	-0.721	-0.170	0.891
Ube2d1	-0.674	0.011	0.663
Ube2d2a	-0.631	-0.063	0.694
Ube2d3	-0.765	-0.172	0.937
Ube2d-ps	-0.612	-0.191	0.803
Ube2e1	-0.624	-0.284	0.908
Ube2e3	-0.653	-0.149	0.802
Ube2g1	-0.672	-0.094	0.765
Ube2g2	-0.600	-0.103	0.703
Ube2k	-0.701	-0.211	0.912
Ube2n	-0.778	-0.028	0.805
Ube2q2	-0.601	-0.122	0.722
Ube2v1	-0.647	-0.323	0.970
Ube2v2	-0.687	-0.213	0.900
Ube2w	-0.667	0.006	0.660
Ube3a	-0.543	-0.187	0.730
Ubiad1	-0.572	-0.138	0.710
Ubl3	-0.710	-0.107	0.817
Ubl4a	-0.582	-0.252	0.834
Ubl7	-0.798	-0.084	0.882
Ublcp1	-0.535	-0.244	0.779
Ubr3	-0.592	-0.186	0.778
Ubxn7	-0.607	-0.132	0.739
Ubxn8	-0.626	-0.263	0.889
Ucp2	-0.832	-0.085	0.918
Uevld	-0.733	0.019	0.714
Ufl1	-0.493	-0.326	0.819

Ufsp2	-0.612	-0.345	0.958
Ugdh	-0.569	-0.284	0.853
Uhrf1bp1l	-0.602	-0.243	0.845
Ulbp1	-0.491	-0.364	0.854
Umps	-0.620	-0.178	0.797
Uprt	-0.611	-0.266	0.877
Uqcrc1	-0.534	-0.219	0.753
Uqcrfs1	-0.513	-0.320	0.833
Uqcrq	-0.594	-0.249	0.843
Usf2	-0.704	-0.136	0.839
Usf3	-0.546	-0.157	0.703
Usp1	-0.558	-0.161	0.719
Usp10	-0.619	-0.166	0.785
Usp14	-0.722	-0.201	0.923
Usp38	-0.694	-0.057	0.751
Usp9x	-0.666	-0.192	0.858
Utp15	-0.661	-0.163	0.824
Utp20	-0.647	-0.162	0.809
Utp23	-0.633	-0.080	0.713
Uxs1	-0.589	-0.159	0.748
Vapa	-0.623	-0.209	0.832
Vat1	-0.550	-0.233	0.783
Vcl	-0.566	-0.202	0.768
Vcpip1	-0.644	-0.078	0.722
Vcpkmt	-0.606	-0.222	0.828
Vdac2	-0.663	-0.188	0.851
Vdr	-0.593	-0.103	0.696
Vegfc	-0.771	-0.006	0.778
Vezf1	-0.649	-0.181	0.830
Vim	-0.644	-0.261	0.905
Vkorc1	-0.683	-0.025	0.708
Vkorc1l1	-0.610	-0.174	0.784
Vmp1	-0.701	-0.021	0.722
Vopp1	-0.602	-0.157	0.759
Vps26a	-0.698	-0.197	0.895
Vps37a	-0.616	-0.122	0.738
Vps4a	-0.510	-0.278	0.788
Vps4b	-0.643	-0.062	0.705
Vsir	-0.811	-0.143	0.954
Vta1	-0.613	-0.275	0.888
Vwa5a	-0.713	-0.131	0.845
Vwa9	-0.686	-0.170	0.855
Vwf	-0.691	-0.151	0.843
Wac	-0.649	-0.114	0.763
Wars	-0.596	-0.120	0.716
Was	-0.709	-0.099	0.807
Wasl	-0.589	-0.123	0.712
Wbp2	-0.526	-0.224	0.750
Wbscr17	-0.587	-0.121	0.708
Wdr1	-0.698	-0.187	0.886
Wdr12	-0.858	0.085	0.773
Wdr18	-0.707	-0.181	0.888
Wdr36	-0.797	-0.102	0.899
Wdr43	-0.582	-0.192	0.774
Wdr82	-0.601	-0.098	0.699
Wee1	-0.535	-0.207	0.743

Wfdc1	-0.693	-0.339	1.032
Wfdc8	-0.776	-0.183	0.959
Wfs1	-0.829	-0.064	0.893
Wipf3	-0.795	0.019	0.776
Wipi1	-0.599	-0.123	0.721
Wisp1	-0.712	-0.138	0.849
Wls	-0.617	-0.188	0.805
Wnk1	-0.646	-0.188	0.834
Wnk3	-0.617	-0.263	0.880
Wtap	-0.582	-0.213	0.796
Wwtr1	-0.583	-0.149	0.731
Xbp1	-0.667	-0.056	0.722
Xdh	-0.612	-0.079	0.691
Xiap	-0.615	-0.124	0.739
Xpnpep1	-0.645	-0.014	0.659
Xpo1	-0.598	-0.121	0.719
Xpo4	-0.588	-0.130	0.718
Xpo7	-0.621	-0.151	0.773
Yipf4	-0.789	-0.100	0.890
Yme1l1	-0.690	-0.234	0.924
Ythdf1	-0.673	-0.338	1.011
Ythdf2	-0.715	-0.138	0.854
Ythdf3	-0.652	-0.074	0.726
Ywhab	-0.559	-0.154	0.714
Ywhae	-0.640	-0.231	0.871
Ywhah	-0.695	-0.230	0.925
Ywhaz	-0.756	-0.194	0.950
Zbtb2	-0.742	-0.155	0.897
Zbtb20	-0.589	-0.142	0.732
Zbtb38	-0.691	-0.039	0.731
Zbtb41	-0.651	-0.129	0.780
Zbtb6	-0.605	-0.263	0.867
Zdhhc13	-0.780	-0.069	0.848
Zdhhc17	-0.753	0.012	0.741
Zdhhc20	-0.694	-0.116	0.810
Zdhhc21	-0.656	-0.051	0.706
Zdhhc5	-0.659	-0.251	0.911
Zdhhc9	-0.565	-0.199	0.764
Zeb1	-0.517	-0.214	0.731
Zfand2a	-0.673	-0.052	0.725
Zfand4	-0.620	-0.252	0.872
Zfand5	-0.602	-0.179	0.781
Zfas1	-0.612	-0.099	0.711
Zfp108	-0.681	0.042	0.640
Zfp111	-0.663	-0.018	0.681
Zfp119b	-0.719	-0.152	0.870
Zfp120	-0.626	-0.205	0.831
Zfp275	-0.560	-0.220	0.779
Zfp277	-0.718	-0.228	0.945
Zfp281	-0.791	-0.151	0.942
Zfp354c	-0.778	-0.124	0.902
Zfp36l1	-0.744	-0.199	0.943
Zfp37	-0.680	-0.203	0.883
Zfp40	-0.850	0.058	0.792
Zfp433	-0.645	-0.062	0.706
Zfp442	-0.814	0.094	0.720

Zfp449	-0.647	-0.025	0.671
Zfp503	-0.492	-0.347	0.839
Zfp518a	-0.525	-0.187	0.713
Zfp52	-0.838	0.105	0.734
Zfp58	-0.652	-0.241	0.893
Zfp593	-0.587	-0.120	0.707
Zfp65	-0.665	-0.071	0.736
Zfp655	-0.701	-0.104	0.805
Zfp706	-0.581	-0.170	0.750
Zfp719	-0.808	0.111	0.697
Zfp729a	-0.709	-0.170	0.879
Zfp729b	-0.536	-0.181	0.717
Zfp758	-0.737	-0.252	0.989
Zfp763	-0.608	-0.231	0.839
Zfp791	-0.530	-0.303	0.833
Zfp81	-0.535	-0.211	0.746
Zfp820	-0.582	-0.322	0.904
Zfp874a	-0.772	-0.098	0.870
Zfp9	-0.829	0.084	0.745
Zfp930	-0.707	-0.141	0.848
Zfp940	-0.689	-0.033	0.721
Zfp942	-0.833	-0.092	0.925
Zfp944	-0.650	-0.322	0.972
Zfp945	-0.607	-0.115	0.723
Zfp946	-0.680	-0.186	0.867
Zfp948	-0.806	-0.070	0.875
Zfp958	-0.611	-0.217	0.828
Zfp994	-0.721	-0.222	0.944
Zfr	-0.565	-0.181	0.746
Zfyve21	-0.749	-0.214	0.963
Zfyve9	-0.557	-0.211	0.768
Zhx1	-0.829	-0.048	0.877
Zhx3	-0.855	-0.070	0.925
Zkscan1	-0.541	-0.220	0.761
Zkscan14	-0.614	-0.134	0.748
Zmpste24	-0.745	-0.211	0.956
Zmynd19	-0.594	-0.108	0.702
Zpr1	-0.606	-0.106	0.712
Zscan22	-0.646	-0.203	0.849
Zswim1	-0.584	-0.170	0.754
