# Supplementary material for: Transcriptomic Evaluation of Pulmonary Fibrosis-Related Genes: Utilization of Transgenic Mice with Modifying p38 Signal in the Lungs
Source: Int J Mol Sci. 2020 Sep 14;21(18):6746. doi: 10.3390/ijms21186746 (PMC7555042; doi:10.3390/ijms21186746)
Supplement: Supplementary file 1 [file ijms-21-06746-s001.zip › Supplementary Table S2.pdf]

Supplemantary Table S2. DEGs altered by BLM in MKK6-CA group.

Name (symbol)	Fold change	FDR p-value correction	CA_PBS_Rep1 - RPKM	CA_PBS_Rep2 - RPKM	CA_PBS_Rep3 - RPKM	CA_BLM_Rep1 - RPKM	CA_BLM_Rep2 - RPKM	CA_BLM_Rep3 - RPKM
Ighv14-4	296.937	0.00566	0	0	0	0.78832	35.0771	0.63956
Gm21541	249.304	3.2E-06	0	0	0	23.3445	13.6134	19.0943
Stfa2l1	120.521	0.03546	0	0	0	0.26734	0.48488	13.0137
Mmp10	103.451	7.9E-07	0	0	0	0.89983	0.97145	0.65182
Vsig4	88.6544	0.00039	0	0	0	0.26104	0.76653	1.56314
Timp1	87.9385	5.7E-19	0.51429	0.86995	0.82196	47.7369	53.5759	68.4246
Sprr1a	82.8504	1.1E-13	0	0.19169	0	12.4961	5.31322	19.4035
Iglv1	78.8515	0.00072	7.7088	1.18545	0.26668	15.9934	16.6	74.2548
Ocstamp	76.449	4.2E-05	0	0	0	0.40858	0.20127	0.30693
Sectm1b	69.8381	8.3E-05	0	0	0	0.39813	0.3129	0.34992
Mmp8	68.0941	1.6E-14	0.07533	0	0.043	4.03924	3.42155	7.22061
Spp1	64.9655	8.3E-42	3.82421	2.76569	0.63272	229.654	291.243	239.107
Wfdc12	64.1368	0.0018	0	0	0	1.99938	0.69072	3.08971
Dio3	55.7593	0.00142	0	0	0	0.32713	0.49443	0.33175
Gm5483	54.4826	0.0222	0	0	0	0.66836	0.80813	4.51865
Ccl2	54.3728	1E-10	0.32105	0.20365	0	4.23073	6.4091	8.08784
Ifitm6	53.1986	0.00968	0	0	0	0.2446	0.5915	1.73636
Ptger2	49.258	0.00147	0.719	0.67102	0.22643	2.1481	1.99795	2.09212
Hist1h3i	47.5905	0.00136	0	0.9398	4.82034	16.4269	9.44275	5.09783
Hist1h3h	45.6583	0.003	6.00028	8.83414	2.79072	22.89	17.0946	14.019
Pcdhgb2	44.431	0.00111	2.42992	2.02204	0.82773	2.6358	2.16776	2.29576
Fgf23	44.2769	0.00222	0	0	0	0.39659	0.25821	0.37126
Wnt10a	42.7706	0.00179	0	0	0	0.2915	0.16267	0.27288
Arg1	42.2017	5.7E-13	0.18565	0.20935	0.14129	6.22386	22.9391	10.545
Sh2d1a	41.0833	0.00363	0	0	0	0.23238	0.14049	0.15711
Gm10718	39.1268	0.02904	0	0	1.07181	3.49429	2.16166	5.93357
Pdyn	37.0123	0.0054	0	0	0	0.17252	0.11589	0.25919
Igkv3-5	36.1997	0.03648	0.71731	0	0	2.75251	18.3924	2.54653
Mmp12	34.5296	2.1E-16	0.07573	0.26686	0.02882	11.2102	4.75244	6.40032
Igkv6-13	33.6049	0.02294	0.25442	0	0	16.3399	0.74555	3.33499
Pde2a_2	33.3386	0.02796	0	0	0	0.23616	0	0.40803
Igkv4-57-1	33.2412	0.0014	0.24487	0	0	4.59929	9.68718	2.20675
E330013P04Rik	33.0266	0.01084	0	0	0	0.06503	0.13105	0.07328
Gpr55	32.7229	0.00971	0.14728	0.29065	0.02802	0.52053	0.46755	0.22121
Igkv5-48	32.6213	6.5E-05	0.50743	0.21459	0	3.68932	27.1371	10.185
Hist1h3b	32.2151	1.6E-08	1.47319	0.2492	0.50454	27.4022	11.2242	29.5695
Tnc	32.1825	5.3E-36	1.1047	1.10331	0.59032	39.561	40.0429	38.021
Serpinb9b	31.98	2.1E-11	0.02808	0.02375	0	1.22486	0.84336	1.40319
Adamts4	31.9667	3.5E-34	0.03798	0.0803	0.08671	3.52058	3.47782	4.46484
S100a8	31.6914	6.9E-07	0.26322	0.7792	0.30049	10.0473	4.72443	72.1334
C430002N11Rik	31.6827	0.02895	0	0	0	0.1835	0.34867	0.07089
Cpa4	30.8189	0.01989	0	0	0	0.16164	0.04886	0.2732
Igkv6-20	30.7391	0.01679	0	0	0	1.91845	0.89218	1.79589
Serpinb2	30.437	0.03779	0.03888	0	0	0.23559	0.05697	0.25485
Gm37988	29.9505	0.0411	0	0	0	1.54281	0.24332	0.99772
Ivl	29.5541	0.03021	0	0	0	0.17276	0.06963	0.42826
Tfpi2	29.216	3.8E-12	0.10457	0	0	1.9323	1.53207	2.44147
Chl1	28.5008	1.8E-53	0.14117	0.03142	0.01696	3.07469	2.4059	3.84706
Naip1	27.9497	0.02586	0	0	0	0.10338	0.075	0.08387
Cemip	27.2995	7.1E-14	0.01493	0.02526	0.01705	1.19439	1.00654	0.6668

Cwh43	26.6806	0.04699	0	0	0	0.09492	0.11477	0.17969
Wfdc17	26.4592	1E-10	1.02819	0.28988	0	18.0659	13.1818	30.7458
Gjb4	26.3434	1.8E-06	0	0.05342	0	1.26294	1.01804	1.29372
Fn1	25.8381	1.1E-27	7.5587	6.82275	2.19519	271.475	196.628	237.141
Hist1h2ag	25.2052	1.1E-08	0.18832	0.63713	0	14.1487	9.24369	11.4172
S100a9	24.5417	2.9E-06	1.57479	0.88795	1.39828	18.6586	11.024	165.856
Has2	22.9647	1.1E-11	0.04311	0.01823	0.02461	1.37141	0.97914	1.04199
Gpnmb	22.5742	1.3E-21	0.37587	0.52455	0.08582	18.5263	9.98192	10.6854
Igkv4-70	21.9855	0.00027	0.241	0	0	9.34499	1.23587	4.73851
Ighv1-54	21.0593	4E-06	0.72682	0.40982	0	16.4405	14.5541	12.1076
Serpina3n	20.5441	7.6E-24	7.43445	7.11135	1.56609	158.11	154.413	231.23
Mt2	20.0997	2.3E-21	8.85415	4.20418	3.19198	131.479	146.8	305.806
Chodl	20.0359	4.1E-05	0.02391	0	0	0.37666	0.2102	0.45054
Fst	19.484	3.5E-20	0.58625	0.13222	0.04462	7.24604	11.052	6.14769
Ighg3	19.4479	2.9E-05	0.75974	0.49978	0.09637	2.04584	18.3053	23.5829
Clec4n	19.3979	2E-16	0.32161	0.65283	0	13.0555	9.04743	13.1214
A2m	18.8801	9.5E-08	0.01855	0.04706	0	0.94387	0.86953	0.63814
Fpr1	18.4694	1.5E-05	0	0.11669	0	1.04494	1.11185	2.82587
Sirpb1b	18.4108	1.8E-06	0.0635	0.10741	0.10873	1.07717	0.721	2.36685
Bcl2a1b	18.3104	2.5E-09	1.22976	0.56733	0.38288	16.7984	9.58244	14.3796
Igkv1-135	17.6881	2.3E-08	1.85457	0.39214	0	17.1357	25.4746	30.9571
Ms4a6d	16.7373	1.1E-11	0.21058	0.48978	0.0601	6.79388	6.82631	10.0922
Saa3	16.3511	1.1E-05	0.0701	0.7411	0.64019	11.5946	16.7668	21.0761
Hist1h2bh	16.2108	1.3E-05	0	0.79641	0	8.27245	3.79405	8.48575
Msr1	16.1741	2.7E-23	0.14397	0.2679	0.06574	3.53289	5.35813	5.01297
Ccr1	15.9976	2.5E-12	0.22568	0.32722	0.18403	5.82113	6.11738	10.0106
Hist1h2bk	15.9082	2E-06	0.76754	0.48688	1.09529	18.8342	9.41847	18.0784
Fgg	15.6653	0.00369	0	0.07382	0	0.89898	0.35167	0.17876
Igkv17-121	15.6076	0.00066	1.25809	0.42563	0	15.2451	4.05535	31.7456
Hist1h4n	15.4245	2.3E-09	12.9572	9.88104	7.51725	94.9786	53.9975	77.4392
Tnfsf18	15.2902	0.00013	0.04421	0.03739	0	0.77679	1.16596	0.54329
Prss22	14.8015	1.3E-06	0.06904	0.05839	0	1.12938	1.315	1.47056
Ereg	14.7482	1.8E-11	0.11063	0.01871	0.05052	1.60872	1.28056	1.75833
Mgp	14.7303	2.9E-13	47.8618	32.8011	7.02756	741.563	457.288	880.359
Ly6d	14.6711	1.3E-06	0.24553	0.20767	0	7.88444	2.24843	2.71557
4930558J22Rik	14.2476	0.00121	0	0.07029	0	1.20851	0.54797	0.74897
Ptger1	14.0205	0.00099	2.10818	2.10854	0.87864	1.94637	1.56895	1.72714
Cd68	13.89	1.4E-13	1.52593	1.7208	0.33181	36.409	16.3956	35.3607
Apoc2	13.8182	0.00041	0.11235	0	0	2.58661	1.9753	2.48509
Ccl7	13.7953	5.3E-10	0	0.48222	0	3.24716	5.09577	5.51174
C3ar1	13.661	2.2E-16	0.40312	0.46656	0.07266	8.52272	7.10322	6.7094
Pla1a	13.461	4.5E-15	0.18527	0.23505	0.10575	3.1711	4.64864	5.08472
Ebi3	13.1866	0.00272	0.2342	0.26411	0	1.08788	0.68629	1.471
Igkv14-111	12.9999	3E-05	1.7374	0.20992	0	23.3089	12.9099	5.49017
Cthrc1	12.8765	4.6E-05	0.06171	0.0522	0	1.12175	1.26593	0.55616
Ccl6	12.6826	5.6E-10	7.68355	7.78973	1.27798	63.9106	59.7187	81.624
Hist1h2ap	12.5276	2.2E-09	4.73743	5.12875	4.54295	47.4178	32.9014	47.195
Cxcl10	12.4958	1.1E-07	0.17041	0.67259	0.25938	4.99022	5.07676	3.58323
Igkv6-15	12.4729	0.00018	1.3161	0.22263	0	16.905	7.90618	10.1353
Mmp13	12.4566	2.6E-07	0.03417	0.1156	0	1.69767	1.17655	0.81184
Clec4a2	12.3939	3.6E-11	0.15608	0.07921	0.07127	1.98588	1.71514	1.86689
Igkv1-110	12.3905	0.00091	8.65543	1.92648	0.26003	96.4652	78.5939	29.8569
Il1rn	12.3443	3.4E-15	0.17576	0.27254	0.13377	6.08774	3.30489	3.83984
Lcn2	12.2066	2.2E-09	2.43753	4.85953	1.66962	64.3276	39.898	100.704
Cxcl13	12.1627	2.4E-10	1.58848	1.00763	0	16.4095	18.1539	16.9829
Tmem37	12.1241	1.2E-11	0.62703	0.58926	0.15907	10.0042	6.89039	13.3562

Cdh17	11.5256	0.01807	0	0	0.02331	0.19791	0.17947	0.11708
Serpina3m	11.4935	5.3E-11	0.42507	0.49434	0.18197	6.11668	7.55159	10.4909
Tarm1	11.3961	0.00013	0	0.07478	0	0.42858	0.64776	0.86926
Clec4d	11.391	3.1E-13	0.17137	0.28988	0.06521	2.97638	3.05483	5.38169
Wisp1	11.3551	3.7E-13	0.2814	0.196	0.07559	3.33965	4.19572	3.24105
Ccl17	11.0743	1.8E-08	1.0662	1.50295	0.20286	20.887	9.24289	27.0782
Hist1h2ad	11.0051	0.00276	0	0.3337	0	5.73718	6.93702	2.90912
Trem2	10.9238	1.3E-28	0.12927	0.21867	0.10541	3.65874	2.42165	2.96532
Arl11	10.8717	0.02186	0.24653	0.20851	0.14072	3.36072	2.21238	2.92853
Gm7932	10.8645	1.4E-05	0.08326	0.03521	0	0.83237	0.97595	0.71623
Il1r2	10.7937	9.3E-05	0.23935	0.08098	0.05465	0.92813	1.29759	5.17684
Gpr176	10.7624	4.1E-06	0.14056	0.21795	0.05349	1.61804	1.44158	1.26666
Hist1h2ab	10.5531	1.9E-09	1.15862	1.30658	0.44089	23.9845	12.1661	13.6053
Cenpk	10.4463	0.00878	0	0.04221	0	0.57457	0.25595	0.24534
Orm1	10.3774	0.00021	0.35356	0	0	1.42813	1.81313	1.93107
Fcna	10.2711	0.00034	0.13295	0.11245	0.07589	0.84581	2.87328	2.72303
Fcgr3	10.1991	8.3E-16	1.94671	1.91349	0.48051	24.7053	18.5014	28.104
Gm21738	10.1973	0.00025	0.13412	1.02095	0.76558	7.31364	4.02855	11.977
Lgals1	10.1724	9.7E-09	14.8424	5.31106	1.30339	119.188	81.3833	139.743
Pira2	10.0449	0.0014	0	0.29008	0.03012	2.126	1.21766	2.26951
Hist1h3c	10.0431	2.5E-11	5.04318	6.0032	1.49264	27.2738	21.4834	24.943
Igkv2-109	9.99512	0.01764	0.24422	0	0	1.77561	0.89456	3.20124
Osm	9.9392	1.4E-06	0.0804	0.034	0.04589	0.97429	1.03079	0.85632
Syt13	9.93152	0.00033	0.04857	0	0	0.29428	0.33804	0.27854
Cda	9.89657	0.00072	0.1765	0	0	1.22977	1.29301	0.72298
Megf10	9.86528	5.3E-05	0.01183	0.01001	0.01351	0.23662	0.25142	0.14543
S100a4	9.80668	4.2E-17	2.5024	3.04245	0.71419	38.4726	32.4255	44.4617
Hist1h2bb	9.79526	2.2E-08	2.22282	1.70911	0.23069	29.8737	15.0999	17.5484
Ly9	9.78073	5.5E-09	0.22096	0.4672	0.15765	3.11255	2.00315	1.94596
Chac2	9.71771	0.01185	0.48621	0.35248	0	1.09417	1.11946	1.93474
Cst7	9.71356	0.01721	0	0.07835	0	0.67351	0.61077	0.60713
Ighg2b	9.69735	1.6E-05	7.12829	3.34623	0.03894	42.7344	40.8526	65.0492
Fcgr1	9.67153	3.4E-08	0.14112	0.3879	0.08055	3.82612	4.08355	2.86136
Hist1h2bj	9.65292	4E-07	2.17001	1.0011	1.35125	28.2078	19.5104	23.9194
Tlr13	9.55904	4.9E-16	0.42744	0.53277	0.07705	5.1387	6.01561	6.17431
Lama1	9.49953	1.8E-15	0.15354	0.16233	0.06573	2.90712	1.87003	1.69816
Iglv3	9.46751	0.00997	0.47946	1.0138	0.27368	2.03349	6.67377	23.7646
C1qc	9.45556	7.2E-17	2.60154	2.36036	0.70198	33.2154	23.806	35.9613
Thbs4	9.4146	6E-07	0.16662	0.09395	0	1.71623	1.20033	1.45608
AU018091	9.40507	0.00064	0.025	0.02115	0	0.46962	0.20149	0.20484
Trem1	9.30364	9.5E-07	0.06097	0.12892	0.06961	0.9605	1.18371	2.44768
Cd53	9.26951	4.4E-11	2.04487	1.64082	0.35914	23.4448	16.4018	23.7545
Ccl9	9.26473	1.1E-17	1.34982	1.54757	0.27395	17.5379	16.4811	15.5556
Hist1h3d	9.18432	2.3E-06	0.48931	2.48309	0	17.4913	9.20058	18.0391
Dspp	9.13988	0.01064	0	0.01743	0	0.09991	0.16611	0.1351
Dhrs9	9.08051	3E-06	0.08534	0.12632	0	0.94366	0.64084	2.06256
Hist1h2an	8.85033	0.00185	1.39447	0.39314	0	5.77337	2.72421	3.61768
Kcnt1	8.85013	9.7E-05	0.03846	0.01627	0.02195	0.19225	0.64807	0.32298
Acod1	8.84465	0.0108	0.03482	0.02945	0	0.18988	0.63776	0.28528
Igkv17-127	8.83996	0.03939	0	4.73409	0	12.7945	21.0618	22.5112
Mmp19	8.82316	6.6E-21	1.82139	1.7897	0.2752	16.9752	14.8347	19.9471
A530064D06Rik	8.7684	0.02716	0	0.08077	0	0.26035	0.20987	0.46939
Kng2	8.74889	0.00432	0	0.06158	0	0.26468	0.32003	0.68596
Tshr	8.66398	0.0174	0.01874	0	0	0.14763	0.09612	0.09213
Cd52	8.64435	1.7E-08	1.61591	1.7572	0.61491	29.5581	8.96311	24.3336
Ube2c	8.57743	2.7E-05	0.21078	0.11885	0.0401	1.68148	1.59563	1.3239

Sfrp1	8.56911	4E-21	2.15182	1.23688	0.54855	18.8221	20.3403	17.338
Ackr1	8.54426	2.9E-05	0.13002	0.10997	0	0.74837	1.57163	1.70429
Foxs1	8.52343	0.00154	0.13934	0	0.07954	1.60405	0.7656	0.91324
Lgals3	8.49885	1.8E-18	4.47348	3.45097	1.90809	58.1396	35.0773	56.8668
Igkv10-96	8.4565	0.00309	7.12382	1.93667	0	24.9723	17.5205	66.4914
Igkv4-59	8.42108	0.00202	1.7374	0.41985	0	6.0152	19.0921	16.8772
Ighv1-53	8.38875	5E-05	1.30111	1.32054	0	20.3386	18.873	6.39563
Fcgr4	8.36618	1.9E-07	0.29088	0.30753	0.08302	2.68768	3.1965	4.64702
Tnfrsf12a	8.28885	3.2E-16	2.84541	1.76484	0.54139	23.2163	17.8574	33.4904
Ccl3	8.16002	0.01041	0	0.2017	0	2.16736	0.61148	0.87919
Svop	8.09065	0.02673	0.02005	0	0	0.10932	0.11749	0.11497
Ddias	8.08298	7.1E-06	0.04934	0.04174	0.11267	0.89692	0.8676	1.15215
Evi2a	8.07002	0.02729	1.15315	1.04258	0.54474	5.01118	3.3209	3.84406
Ano3	8.03863	0.03226	0.01265	0	0	0.07667	0.03708	0.10367
Lox	8.02196	6.3E-13	4.10165	3.4691	0.58531	29.5108	39.6472	34.1319
Ackr3	8.01964	2.8E-15	1.90969	0.96911	0.18687	15.5044	12.1115	12.2703
Cxcl9	8.00337	0.0003	0.03028	0.20491	0.13829	1.66974	2.46266	1.56307
RP23-110E20.5	7.92766	0.03767	0	0.02217	0	0.15879	0.2304	0.38649
Rgs16	7.92135	0.01	0.19525	0.16514	0.2229	1.67983	1.40178	1.72756
Cxcr2	7.90986	0.00026	0.29966	0.25345	0.17105	1.52511	0.76836	8.17521
Emilin2	7.86941	3.9E-08	1.168	1.02739	0.53336	5.46322	4.94577	7.25324
Mmp3	7.866	2E-10	1.57216	1.32971	0.17369	14.9027	13.0037	13.5864
Cks2	7.83749	0.01064	0.39597	0.44654	0.30136	3.03889	3.67443	3.67657
Gm14548	7.79952	0.00027	0.21461	0	0.049	1.56033	0.75466	3.30541
Apoe	7.79465	1E-10	37.0004	20.3355	4.84378	309.925	148.533	316.023
Bcat1	7.78169	0.0157	0.0098	0.00829	0.01119	0.07124	0.23687	0.10435
B2m	7.76882	2.3E-07	47.1557	46.0816	18.1869	466.85	228.128	411.124
Ighv1-15	7.71544	0.04929	1.82155	0.66027	0	7.56784	3.24082	23.2375
Pilra	7.7135	1.5E-07	0.11824	0.25001	0.10123	1.95212	1.60245	3.39031
Cd300lb	7.71091	1.2E-06	0.12525	0.1589	0.14298	2.9975	1.00932	2.15483
Ch25h	7.70492	1.9E-08	0.74423	0.68668	0.07724	7.41964	6.64177	8.14804
Gdf15	7.69486	6.2E-08	0.19727	0.25028	0.28151	4.39253	1.51747	4.5657
Clca3a1	7.68649	2.8E-14	0.35139	0.39626	0.24069	4.0593	5.62902	3.95352
Adamts12	7.64687	4.7E-11	0.26373	0.26023	0.06691	2.5211	3.22008	2.01656
Thbs1	7.61942	1.8E-15	19.7023	11.5375	3.58846	153.253	103.328	171.553
Ahsg	7.61478	0.00333	0.06197	0.05241	0	0.6007	0.18158	1.06608
Ctsd	7.5601	6E-11	36.2864	29.8689	6.96326	237.319	169.347	217.962
Hist1h3a	7.49664	5.5E-07	1.02627	2.16999	1.56212	20.0011	15.4126	21.8601
Clec7a	7.46542	3.9E-13	0.8656	1.37636	0.43479	13.8455	8.67489	13.9276
Tnf	7.45312	0.00488	0.05573	0	0.12724	1.0467	0.73486	0.50221
Itgam_1	7.41417	6.7E-20	1.39988	1.33776	0.72641	10.971	11.0146	12.8538
Lilrb4a	7.40012	5.4E-12	0.54489	1.95866	0.81644	15.1452	13.6721	18.2748
Pappa	7.38668	2.8E-13	0.28991	0.1191	0.03782	1.58085	2.34837	1.45898
A630033H20Rik	7.37543	0.00171	0.05272	0.04459	0.03009	0.55899	0.65658	0.4967
Gm13056	7.35035	0.01365	0.32389	0	0	1.27556	0.47456	2.91886
Hist1h4a	7.31031	3.7E-06	1.1862	4.1564	1.74108	25.6678	17.7525	37.4839
Clec5a	7.17901	1.8E-12	0.1665	0.20118	0.05431	1.55643	2.00391	1.92918
Hist1h2bp	7.17661	0.00437	0.12937	0.9848	0	5.87888	3.12768	3.28569
Hist1h1a	7.13194	3E-09	0.99589	1.44396	0.16242	10.2577	12.9241	10.1404
Ighg2c	7.08966	0.01577	45.0489	22.1968	0	161.971	256.148	246.947
Pilrb2	7.02173	0.00229	0.04667	0.03947	0	0.31106	0.54707	0.8412
Ccl12	6.98912	0.00097	0.34018	0.28772	0	2.8855	2.49212	3.06562
Wfdc21	6.94866	0.00089	0.63137	0.534	0	2.16769	3.70028	9.48293
Tnip3	6.92775	5.2E-07	0.10044	0.10194	0.04586	1.30226	0.79466	1.03678
Hist1h2ai	6.91356	0.00453	1.39447	0	0	6.33662	2.55394	7.23537
Vcan	6.88896	5.4E-22	0.79578	0.57156	0.20909	5.70159	5.31624	7.5129

Shcbp1	6.88873	2.3E-05	0.01451	0.06136	0.01656	0.29011	0.48896	0.43982
Folr2	6.88499	0.00058	0.47264	0.04997	0	1.21705	2.85658	2.08127
Marco	6.86808	0.00026	0.0949	0.40131	0.10833	1.35115	3.68458	2.60444
Plau	6.83179	3.7E-11	0.54576	0.52754	0.13351	7.18025	3.82688	3.64084
Emp3	6.757	6.4E-08	2.89961	1.87539	0.58415	22.0119	14.3698	25.7814
Clec4e	6.58427	1.8E-05	0.11038	0.09336	0.042	0.52386	0.91644	1.49206
Ly6c1	6.52027	2.3E-13	13.5618	10.7332	3.23322	107.941	74.5343	101.869
Fcer1g	6.51109	1.5E-09	3.23966	2.47488	1.07373	30.2026	19.2165	28.596
Retnlg	6.50433	0.00869	0.45068	1.52471	1.37199	5.27913	3.4117	39.0144
Egfem1	6.47114	0.00431	0.02187	0.01849	0.02496	0.21198	0.22427	0.35829
Chrna7	6.45608	4.7E-10	0.14192	0.17148	0.04629	1.89175	0.86148	1.52814
Wnt7a	6.35551	1.6E-09	0.20156	0.12177	0.09862	1.62251	1.28679	1.86365
Acp5	6.33418	1.5E-06	1.07985	1.06554	0.56502	7.0606	5.20803	5.7504
Itm2a	6.33219	4.3E-09	0.62847	0.75303	0.11958	6.31458	4.02861	6.82213
Hist1h2af	6.32058	0.00607	0	0.96807	1.30666	8.32178	5.19878	7.68919
Tgtp1	6.30473	0.03443	0	2.0729	2.66779	3.72961	3.02728	3.12857
Ear2	6.29131	7.6E-06	2.41365	2.4712	0.29005	21.8589	9.49257	18.7332
Lst1	6.27848	0.00544	0.44018	0.55845	0	4.93391	2.57979	4.50777
Igkv4-68	6.2648	0.02589	0	0.58821	0	6.461	2.03797	2.27905
Hist1h2bm	6.19542	0.00045	0	1.21656	0.82104	9.44118	6.67377	7.07045
S100g	6.16667	0.00392	1.14172	1.44847	0.43446	8.41627	3.90328	16.8365
Tmem141	6.15759	0.01918	0.73265	0.82622	0.09293	1.62764	1.31203	2.40093
Selp	6.10683	2.9E-13	0.28976	0.26258	0.09451	2.23215	2.16828	3.20477
Prnp	6.07811	7.2E-06	13.0204	9.26493	3.56027	25.4615	19.7913	31.6498
Ms4a1	6.07555	0.00755	0.17715	0.18729	0.03371	1.89624	0.44342	0.38703
Atg9b	6.0633	5E-05	0.11573	0.01958	0.05285	0.67317	0.64438	0.66371
Gjb5	6.03976	0.00094	0.10614	0.08978	0	0.64311	0.93313	0.69568
Tnfrsf23	6.03213	0.01782	0.10896	0.16128	0.15549	1.61745	1.45681	1.56219
Ccnb1	6.02675	6.7E-07	0.15162	0.12823	0.12363	1.49603	1.28527	1.1534
Tmem42	6.01228	0.01827	0.90077	0.76185	0.20566	0.94598	1.2758	1.57431
Gyg	5.99087	1E-07	4.55909	2.17518	0.53381	26.9144	16.7569	26.1774
Rwdd2a	5.98311	0.02734	0.13237	0	0	0.40101	0.72732	0.32534
Lamc2	5.97883	6.3E-13	2.56777	2.43123	0.46695	18.208	14.1584	21.5951
RP23-145I16.3	5.9733	0.03772	0.71427	0	0	1.67697	1.96227	1.02405
Fen1	5.9712	0.00025	0.75423	0.70171	0.51662	3.03889	3.45341	4.20179
Eda2r	5.96078	2.8E-10	0.15505	0.1967	0.1239	2.05728	1.13586	2.03236
Hist1h4j	5.92037	9.2E-06	2.12413	1.69674	0.53887	17.5171	8.12642	18.2722
Brinp1	5.9114	1.6E-12	0.28077	0.24997	0.03374	2.06819	1.39651	2.49389
Cdkn1a	5.90376	3.5E-20	6.61509	10.0431	3.84627	70.1021	55.0236	96.8097
Serpine1	5.90137	2E-10	3.27552	1.91336	0.56494	21.6305	19.4042	13.5912
Hist1h2ae	5.89784	0.00208	0	1.51145	0.22668	6.85733	4.21845	6.50686
Sectm1a	5.89279	0.02877	0	0.09131	0	0.58872	0.27683	0.30958
Rps27l	5.88503	5E-08	4.25347	2.27211	1.5334	28.4837	17.7123	37.6896
Etv4	5.86853	0.00239	0.05885	0.07466	0.03359	0.67749	0.6036	0.53036
Bmper	5.84083	2.7E-15	0.98273	0.47953	0.0863	5.53055	4.55503	4.97002
Rpl39	5.83802	5.9E-10	10.5732	4.74069	2.61769	54.761	34.1099	56.4087
Ighv1-80	5.82972	0.00298	3.90332	1.54063	0.29707	17.1853	20.7793	18.3342
Shc4	5.81787	4.9E-06	0.07738	0.06544	0.01262	0.52239	0.46968	0.49807
C1qb	5.80371	1.2E-11	5.12095	12.2126	3.83351	78.2286	60.8862	87.415
Tfec	5.799	8E-06	0.08164	0.19334	0.05592	0.84088	0.98085	1.77909
Cybb	5.78827	7.2E-11	1.52544	1.83553	0.50269	16.055	10.8296	11.7629
Ighv1-7	5.78166	0.00011	0.47081	0.99551	0	5.9904	4.13897	5.9786
Srpx2	5.77626	1E-06	0.46888	0.13219	0.03568	2.10223	2.24418	1.79262
Igkv6-25	5.76998	0.00019	1.20181	0.60988	0	8.44661	3.69784	5.31679
Ighv1-64	5.75733	0.00094	3.15735	1.14447	0	18.7199	7.26959	13.6724
Rspo3	5.75135	0.00256	0.11824	0.025	0	0.42982	0.64964	0.36325

Rpl29	5.73922	4.8E-09	7.13828	2.95011	1.85207	52.4402	27.1575	61.4714
Mmp14	5.72458	1.2E-30	3.95142	3.02948	1.36303	30.331	24.8868	28.0172
Tgfbi	5.72452	3.5E-19	7.12829	5.56743	1.98575	45.6068	43.5011	53.7885
S100a11	5.70015	8.5E-08	62.0238	32.8055	14.0797	309.188	166.301	392.01
Mt1	5.69318	3.7E-10	13.8546	7.00598	3.59845	57.1164	64.3356	115.006
D130017N08Rik_	5.68702	0.02663	0.15299	0.05176	0.06986	0.40786	0.4035	0.42616
C1qa	5.68063	2.7E-13	7.99204	10.5478	2.306	71.1968	57.3267	75.6204
AI506816	5.63235	4.7E-12	2.63782	1.93911	1.26646	14.4735	13.2021	14.0973
Serping1	5.63172	2.1E-06	18.2282	8.17373	2.51148	92.2807	58.1067	109.041
Tal2	5.60786	0.03343	0.03074	0.026	0	0.26077	0.18018	0.20149
Fgf2	5.60386	1.9E-06	2.80369	1.91086	0.57488	9.63149	8.505	9.62261
Hist1h2bf	5.60151	0.00025	0.39626	1.50817	0	12.0043	3.48356	10.713
Tlr8	5.58409	4.9E-07	0.38206	0.45239	0.11631	3.30249	2.94822	3.13004
Rps27	5.58131	2.7E-05	22.2433	8.88802	5.89839	121.928	50.8746	145.784
Serpina3f	5.5625	9.9E-06	0.35897	0.87709	0.50087	3.93904	4.90892	8.82262
Cers3	5.55792	0.00672	0.02805	0.04745	0.06405	0.39091	0.24661	0.82735
Snai1	5.54882	2.7E-15	1.52135	1.04845	0.38595	10.9929	6.60464	11.5405
Gda	5.5393	3.7E-19	1.78655	1.77547	0.8838	11.7445	14.6806	17.5516
Fbn1	5.53479	5.1E-21	5.69354	6.12765	1.82269	41.4912	45.9753	39.031
Ighm	5.52501	0.00021	34.2879	7.41387	1.65861	42.4292	164.409	125.971
Lgmn	5.52295	7.3E-26	7.3133	8.64634	2.91763	64.6545	53.836	64.8109
Col12a1	5.51859	3.1E-10	1.10363	1.06533	0.19173	7.41717	7.40405	6.20626
Igkv1-117	5.49789	0.00211	4.74775	1.72095	0	12.8761	16.5627	35.377
Litaf	5.47479	6E-16	3.83742	3.15791	1.42081	28.5497	19.8562	33.2227
Ighv1-18	5.47058	0.01964	0.78066	0.66027	0	2.20729	3.24082	8.7407
Ly86	5.45957	5.9E-06	1.09935	1.62717	0.41834	15.4308	7.71819	7.73057
Flrt3	5.4583	9.8E-05	1.07014	0.52401	0.10716	5.31217	2.13187	4.66047
Hbb-bs	5.45388	0.00863	15.8176	7.94647	1.22193	52.6737	24.5697	76.875
Ms4a4a	5.45164	7.8E-05	0.54628	0.73925	0.37418	4.56752	7.04351	4.56492
S100a6	5.40186	2.2E-12	14.7664	9.55051	3.89016	84.4064	62.5079	113.475
Cd302	5.38804	5.4E-05	2.36102	1.71163	0.53907	14.632	9.93322	18.1269
Ctss	5.35379	5E-12	15.1333	26.7366	8.36936	195.89	135.896	150.099
Lrrc25	5.35368	6.4E-09	0.50533	0.32055	0.21633	3.48266	2.54511	3.93292
Colec12	5.34765	2.7E-07	5.93887	3.09286	0.72193	30.5852	17.9872	33.45
Slc7a8	5.34714	4E-09	0.31707	0.30648	0.15513	2.26409	2.67122	2.44915
Pla2g7	5.33942	3.2E-10	1.00613	0.90415	0.35894	7.23895	4.58373	7.36698
Lhfpl2	5.32344	5.3E-11	0.26487	0.39205	0.08819	2.9355	2.11833	1.98013
Gm15922	5.32048	0.02534	0.34298	0	0	0.76728	0.90841	0.67005
Sirpb1c	5.31811	5.3E-05	0.19249	0.35275	0.25638	2.35199	1.85674	2.70719
Hist1h4k	5.29952	2.3E-05	4.09848	5.69484	3.67624	20.5751	12.0101	18.2276
Hist1h3e	5.29805	1.2E-05	2.07585	1.66332	1.12254	14.8279	8.72434	15.7535
AA467197	5.29585	6.7E-07	0.45555	0.64216	0.52006	4.41615	5.45096	5.66038
Hist1h2bn	5.29052	0.0316	1.67812	1.82484	1.09471	7.4077	7.90315	6.28484
Angptl4	5.28395	2.4E-07	3.41891	1.64565	0.63464	20.8156	12.0957	16.783
Tubb3	5.27446	0.03775	0.09779	0.12407	0.05582	1.36276	1.32537	1.60233
Nrcam	5.27309	3.8E-10	0.50603	0.56046	0.16505	4.21929	4.33379	2.56635
Cpxm1	5.2614	1E-11	2.70407	1.68353	0.47162	16.247	13.9494	12.2151
Ms4a7	5.26121	3.9E-07	0.14358	0.34696	0.11708	2.63464	1.45758	1.86526
Ptprt	5.25278	2.9E-07	0.06518	0.06738	0.01654	0.746	0.23346	0.45096
Sirpb1a	5.24233	0.0011	0.19934	0.13488	0	0.99037	0.67176	1.27382
Nup43	5.22123	0.00272	0.28543	0.18106	0.16292	1.01601	0.86255	1.05228
Iglc2	5.19375	0.00161	3.58187	1.38851	0.51113	9.85628	10.9335	27.3884
Rps26	5.19218	2.6E-05	31.8762	11.7519	8.63097	177.286	85.3257	222.311
Cilp	5.17832	0.0001	0.51555	0.41787	0.07357	2.52491	4.21749	1.65426
Plat	5.17598	2.5E-07	2.23917	1.16354	0.28403	10.5608	9.07045	10.671
Stra6l	5.14813	0.00146	0.00955	0.05652	0	0.24873	0.28676	0.1095

Hist1h2bg	5.14322	7.5E-05	4.97857	3.56297	2.62318	51.6273	24.1281	37.9635
Ifi211	5.12867	1.1E-05	1.94444	0.90884	0.58415	6.8516	8.32199	7.5877
Ccl5	5.0786	0.00822	0.666	0.80471	0.10862	7.09042	2.36985	4.91065
Itih3	5.06274	0.00039	0.10045	0.02124	0	0.38039	0.29437	0.45264
Bgn	5.05387	2.2E-11	42.7849	28.2078	7.84304	236.41	172.758	235.618
Eln	5.05095	4.5E-16	15.1343	20.1752	10.4327	112.242	183.902	121.916
Hist1h2ac	5.04975	0.00099	1.72697	0.81147	1.09529	9.88212	4.21723	5.65933
Ifi27l2a	5.04058	1.6E-06	4.93184	3.33701	2.7025	38.1283	33.2399	27.3134
Hexb	5.03832	7.5E-08	4.12554	3.12415	1.04052	26.9433	16.7986	25.7814
Sparcl1	5.03797	3.3E-10	50.3492	26.6186	9.86975	240.522	196.89	285.201
Dab2	5.03555	1.1E-10	1.97803	1.80977	0.31245	10.2735	10.5264	12.8826
Cstb	5.0247	1.4E-13	7.16672	5.41665	2.26299	47.8565	33.9593	49.4695
Hmox1	5.01564	1.9E-10	2.55599	1.8971	0.65505	14.0325	14.5979	15.1709
Lrg1	5.01512	5.9E-18	6.76074	9.37771	3.78187	59.2722	45.4674	78.0414
Slc7a5	5.01217	2.2E-06	1.15773	0.72918	0.39369	4.32809	4.74601	5.79178
Tlr6	5.00991	0.00033	0.07352	0.10364	0.08393	0.63848	0.5027	1.08419
1200007C13Rik	5.00901	3E-06	0.14882	0.1888	0.04247	1.19471	1.00847	1.34113
Ttk	5.00033	0.01515	0.01857	0.12564	0	0.52876	0.53052	0.28903
Fcrls	4.99676	9.7E-11	0.54787	0.62887	0.26805	4.93174	4.18567	3.78312
Ckap2	4.96871	6.9E-09	0.19887	0.24029	0.0973	1.63524	1.78991	1.34995
Glrx	4.96659	2.9E-12	2.241	1.2636	0.6202	11.0268	9.40269	15.5221
Rpl37	4.93567	2.8E-05	21.4912	8.19742	4.93095	95.3607	41.5989	130.152
Aprt	4.9337	3.6E-11	2.37534	1.67419	0.83934	8.66934	6.62918	8.01573
Ctsb	4.89816	2.9E-12	15.9585	11.9651	4.29055	101.069	73.6061	91.3769
Ulbp1	4.88955	0.002	0.05021	0.21235	0.17197	1.55158	1.36107	1.06957
Col28a1	4.88858	8.7E-08	0.24213	0.22054	0.10631	1.6137	2.19679	1.00708
Atox1	4.88819	2.1E-05	4.05194	2.85589	1.73465	30.7898	11.0079	35.6855
Slfn4	4.88438	0.00352	0.18433	0.60414	0.28935	1.56359	5.89121	1.9821
Il6	4.87513	0.00595	0.11286	0.06363	0	0.38747	0.68898	0.49311
Ly6c2	4.86991	2.6E-06	1.93342	1.07137	0.19028	5.10985	4.22482	4.7246
Pus3	4.86214	0.02501	0.92198	0.54586	0.35085	1.41515	0.65292	1.08265
Uba52	4.85855	2.4E-09	7.49968	4.17716	2.01861	29.9234	18.046	43.9582
Myadm	4.85562	2.5E-07	17.0035	11.8119	3.46796	46.5695	33.5761	48.572
P4ha3	4.83266	2E-08	0.20394	0.36963	0.09978	2.06531	2.34784	1.456
Serpina3i	4.83225	9.5E-05	0.20535	0.24315	0.18754	1.39345	2.13617	1.88418
Tmem252	4.82364	1E-08	2.41143	1.5375	0.25411	11.3961	8.94171	13.0996
C5ar1	4.82292	3.1E-16	1.74146	1.3226	0.40573	8.9362	9.29496	10.7439
Fkbp11	4.81882	0.00571	0.2499	0.21136	0.28528	1.89261	2.28842	2.25203
P2ry6	4.81422	1.3E-08	1.2538	0.89977	0.26024	7.84979	5.01015	7.28368
Igsf6	4.8128	2.2E-05	1.13385	1.31417	0.28765	4.02009	3.78406	5.19502
Hist1h2ak	4.80057	0.00046	1.62688	0.58971	1.06129	13.7998	11.7481	11.8051
Bub1	4.79811	0.00018	0.10489	0.10646	0.07185	0.85158	0.82988	0.75619
Cxcl14	4.79233	1.1E-07	9.92037	7.62771	0.97236	43.1062	42.9816	53.6076
Sec61g	4.76679	3.3E-05	1.00086	0.87786	0.16927	6.37846	4.26355	10.2039
Tnfaip8l2	4.76358	1.6E-05	0.81406	0.68852	0.18587	5.67209	2.98188	5.86894
1810055G02Rik	4.74396	2.4E-06	0.87358	0.41048	0.33243	4.67536	3.23545	5.68573
Ear1	4.74227	0.00578	1.00096	0.10582	0	2.8049	1.46659	2.76765
Fcgr2b	4.74058	5E-15	1.45131	1.80121	0.52226	10.3033	9.21069	10.2098
2810417H13Rik	4.72912	0.00104	0.55034	0.69018	0.38996	1.58672	1.52929	1.35261
Fgl2	4.7191	0.00487	6.17276	6.05696	2.94537	7.53837	6.85384	10.9636
Emp1	4.71705	7.5E-08	14.627	9.34719	1.97906	73.967	54.4712	64.9538
Ly6a	4.71491	3.5E-08	26.757	23.5281	8.39364	177.455	121.943	174.684
Lair1	4.71453	6.9E-07	0.30548	0.35138	0.0558	1.87304	1.84403	1.66175
Kdelr3	4.69059	1.1E-05	1.42109	0.76487	0.22123	6.10539	6.48311	6.45624
Bst1	4.68929	4.7E-07	0.82609	0.9083	0.06287	4.65475	3.97405	5.36909
Gm26703	4.67726	0.01934	0.13307	0.12662	0.09495	0.45352	0.68242	0.42245

Cxcl15	4.67501	3E-05	22.1151	16.4528	3.52964	92.155	62.572	167.459
Tubb6	4.66471	1.7E-09	3.7949	2.76998	0.83085	18.2366	16.9092	23.3814
Atp1a3	4.6554	2.8E-08	0.18396	0.44085	0.15751	2.3871	1.72955	2.68771
Alox5ap	4.64612	5.2E-09	2.70153	3.97139	0.88117	16.2902	12.723	21.2367
Foxf1	4.61323	6.5E-06	26.9285	15.8982	5.13801	37.7411	26.8762	43.2861
Ccr5	4.60913	1.7E-07	0.34655	0.64117	0.34617	4.06814	3.57812	3.50456
Slc39a14	4.60793	3.1E-22	1.23804	0.95192	0.63255	6.7352	8.46723	7.89427
Lipa	4.54372	8.2E-07	3.66089	3.30447	0.31608	22.2739	14.0182	16.5838
Rnasek	4.53528	0.00026	4.8507	1.65851	1.29603	23.9493	14.5168	25.2812
Cdh11	4.53257	1.3E-09	2.14393	1.24068	0.31619	10.0624	6.94383	10.5638
Igkv13-85	4.52826	0.00389	1.57932	0.22263	0	6.06027	2.69967	3.01903
Rtn4rl2	4.52134	7.4E-08	0.97039	0.41037	0.5539	4.88725	3.68779	6.95621
Ifi204	4.50754	9.2E-05	0.35909	0.54235	0.49779	3.55878	5.37411	2.29047
Tnfrsf26	4.50386	0.00015	0.11353	0.14404	0	0.75667	0.70698	0.6976
Ifitm2	4.5014	4.2E-07	18.2675	9.67121	5.25337	100.964	53.122	145.202
Glipr2	4.49873	2.3E-13	1.22046	1.7315	0.4045	8.51566	7.64312	10.9341
Dctd	4.49377	0.00447	0.05359	0.06798	0	0.3409	0.43183	0.2634
I830077J02Rik	4.48683	0.00061	0.12752	0.18875	0.07279	0.96579	0.88751	1.38427
S100a10	4.47019	1.1E-06	9.72158	5.2266	1.37652	40.409	31.8004	45.9346
Rpl31	4.45767	3.2E-07	15.113	6.50737	4.14074	68.2924	34.8255	85.8597
Myc	4.4559	1.7E-08	1.00851	0.90985	0.61404	6.86402	6.13228	7.46361
Col5a2	4.40779	6.8E-14	3.48551	5.00997	2.45703	29.173	35.1066	23.4996
Gapdh	4.3923	1.1E-05	4.6292	1.71671	1.01629	20.7983	11.4522	27.3352
Cldn4	4.38739	8E-10	1.91125	1.31872	1.14836	11.0314	10.2433	13.4742
Ms4a6c	4.37575	3.3E-11	1.60398	1.65809	0.55951	10.6091	9.04146	9.454
Ildr2	4.32582	2.7E-08	0.35424	0.59921	0.1011	2.68282	2.95194	1.9952
Rpl35a	4.32518	3.8E-07	5.02538	2.41001	1.09417	16.4168	10.8359	19.0998
Hsd11b1	4.32423	4.5E-06	5.90863	3.8373	0.76286	24.0367	13.7331	32.3577
Slfn9	4.31616	0.00014	0.21437	0.27197	0.26221	2.29152	2.37814	1.37991
Tlr7	4.29828	4.1E-06	0.71602	0.28387	0.10218	2.53517	2.80308	2.49308
Tceb2	4.29527	7.8E-08	11.6869	6.38696	2.87362	54.0326	36.7496	62.0137
Igkv8-30	4.28697	0.01459	7.9424	4.82823	0	31.4294	9.27331	46.9714
Arhgdib	4.2771	2.3E-07	2.46135	2.6701	1.64929	19.6137	11.015	18.1483
Ighv1-39	4.27634	0.00273	3.12265	1.54063	0	7.56784	13.9164	9.80664
Hist1h2be	4.27471	1.8E-05	1.34871	0.9506	0.51323	8.1262	3.62287	8.93159
Adgre1	4.27378	8.9E-08	1.23848	2.28541	0.41773	11.3749	7.93887	10.2847
Loxl1	4.27179	3E-12	5.42984	4.35815	1.3283	28.1986	23.441	28.3247
Scarb1	4.25941	8E-12	2.19792	2.35141	0.66468	12.2321	8.42415	18.1974
Cysltr1	4.24161	3.2E-05	0.17763	0.09014	0.02028	0.92556	0.65065	0.69852
Ifi205	4.23784	3.1E-06	1.15894	0.70279	0.22467	3.89512	4.42137	5.82222
Il2rg	4.23191	8.8E-07	2.02802	1.84472	0.61156	13.4466	7.9612	10.5331
Ifitm3	4.22827	4.3E-09	30.9847	25.1809	12.7648	189.363	120.404	213.229
Pgf	4.22464	1.1E-07	0.46188	0.53715	0.39547	3.673	3.38373	3.45292
Mest	4.22004	4.1E-08	0.57882	0.39164	0.07929	2.42682	2.95131	2.7314
Cdk1	4.20995	0.00021	0.15908	0.26909	0.15134	1.57425	1.70925	1.12949
Fxyd5	4.20801	2.6E-12	1.80845	2.00869	0.64672	12.0924	8.65148	12.406
Cyp7b1	4.20336	3.8E-12	1.01205	0.92731	0.33698	5.87635	4.07782	7.28943
Cd33	4.20122	1.8E-07	0.82276	0.88475	0.10734	3.68885	3.14288	5.97977
Cd300ld	4.19337	2.1E-05	0.48345	0.52572	0.07884	2.19686	1.82146	4.21532
Tyrobp	4.19084	2.4E-13	6.8469	9.49722	3.43924	56.5014	34.0084	60.8055
Nuf2	4.17904	4.3E-06	0.07202	0.14619	0	0.69818	0.56983	0.42483
Prc1	4.16907	5.7E-05	0.2221	0.42265	0.20284	1.70225	1.87113	1.03714
Hist1h3f	4.14473	6.8E-06	2.80044	2.45967	2.21331	19.9693	11.6783	22.3251
Hba-a2	4.14033	0.03816	33.5498	15.1724	1.71962	86.5359	42.4353	149.658
Gins2	4.13482	0.02249	0.17835	0.39597	0.02545	1.66141	1.22492	1.09586
Tm4sf19	4.12988	0.03897	0.14533	0.12291	0	1.23271	0.3194	0.53577

Atp6v0d2	4.12549	1.8E-06	1.54695	1.27994	0.19196	7.37593	5.86354	7.60412
Tubb2b	4.12242	1.1E-05	1.04549	0.52251	0.27126	6.16167	4.00366	4.16581
Rps29	4.11522	1E-06	32.6865	16.6097	7.97459	157.315	78.0172	189.826
Enc1	4.11362	2.7E-13	1.75279	1.28699	0.3958	7.55059	8.25481	8.0636
Anxa1	4.10885	1.5E-15	14.4291	9.06405	5.91725	70.3631	54.1105	91.9007
Mrc1	4.1087	7.5E-08	4.17191	3.5164	0.81833	14.063	20.1444	22.4804
Cdc20	4.10024	0.0004	0.44006	0.24813	0.2791	2.22189	2.86567	1.88274
Dcakd	4.08418	4.1E-05	1.0363	0.81604	0.48954	5.23953	3.74358	4.91833
Frzb	4.08162	0.00788	0.08465	0.04773	0	0.4445	0.35141	0.18493
Capn6	4.07908	6.8E-05	0.08202	0.03964	0.02675	0.38337	0.34337	0.33599
Tnfsf9	4.07022	0.02208	0.74258	0.12561	0.16955	1.34975	1.14242	2.12928
Lrrc8a	4.06793	0.0006	11.1522	7.10114	3.00131	23.2253	20.7357	26.7668
Ccna2	4.05908	1.6E-07	0.26844	0.54869	0.15323	2.35835	2.86795	2.47414
Milr1	4.04818	0.00029	0.18459	0.28103	0	1.11843	1.29824	1.14935
Slc11a1	4.04773	2.3E-10	0.64097	0.77147	0.197	4.39134	3.14249	4.12014
Ctla2a	4.03566	1.4E-06	11.176	7.31444	1.67077	40.5879	25.7321	67.6894
Gjb3	4.02903	0.00054	0.27875	0.39294	0.10607	1.40742	2.34843	1.86501
Cd200r4	4.02608	0.00175	0.22332	0.22666	0	1.1095	1.07977	1.13433
Ttyh2	4.02465	9.1E-14	1.14813	0.68182	0.11155	4.91384	3.65079	4.88319
Rpl24	4.02318	5.4E-05	17.3807	9.0002	4.35308	81.4517	46.1897	94.5169
Aldh1a3	4.01145	0.00126	0.13848	0.10039	0.06775	0.49143	0.59421	1.02106
B430306N03Rik	4.00845	0.00019	0.0395	0.08909	0.10522	0.59033	0.54981	0.57171
Tmem26	3.98536	0.02427	0.05424	0.03058	0.08256	0.49293	0.22516	0.48879
Cav2	3.98257	4.8E-05	8.92835	5.25821	1.71962	19.398	12.6403	23.9632
Plek	3.97904	5.7E-16	2.19894	2.22146	0.81353	12.6322	11.1572	12.7439
Hbegf	3.97669	4.2E-17	2.69432	1.92071	0.83487	12.9196	9.55904	15.7667
Serpina3g	3.9741	6.1E-05	1.17155	2.42215	1.38698	10.9365	10.7125	13.8282
Gtse1	3.96135	1.2E-05	0.16784	0.22713	0.26825	1.7084	1.4263	1.51253
Prr11	3.95953	0.00693	0.09554	0.18182	0.21814	1.09985	1.71483	0.84143
Mmp9	3.954	0.00425	0.8775	0.32743	0.20624	1.42298	1.68276	6.63924
Rpl26	3.9503	7.5E-06	19.988	10.0577	5.05471	81.7834	42.0757	97.1636
Il1b	3.94505	0.00749	0.47896	1.01274	0.60146	4.38192	1.85968	10.4768
Tmem173	3.94253	7E-09	1.5871	1.14099	0.90592	10.7458	6.74365	8.41907
Ccdc184	3.94143	0.01858	0.11765	0.13268	0.13431	1.1643	0.74699	0.83536
Dpep2	3.93847	1.9E-05	0.34201	0.52749	0.11484	2.14533	2.47609	2.20861
Serpine2	3.93198	1.3E-12	5.87402	4.65396	1.35177	29.1892	22.5709	26.4013
Sh3bgrl3	3.91306	1.4E-05	12.3489	9.12719	4.95321	66.4617	43.9297	69.816
Kif2c	3.91216	0.00011	0.08374	0.14165	0.0478	0.72298	0.72082	0.49737
Mpeg1	3.90754	1.5E-08	7.79583	10.3002	2.14075	56.0547	34.4677	43.0849
Ptafr	3.89153	1.6E-08	0.65295	0.57349	0.20069	4.4278	2.59411	2.79811
Spon2	3.89114	1.5E-05	0.27894	0.23592	0.10615	1.8215	1.02175	1.4981
Gm26870	3.88393	0.00407	0.95002	1.00439	1.43315	8.75735	4.64814	16.8448
Ap1s2	3.87656	2.5E-06	1.45331	1.08915	0.25202	4.48069	6.72502	6.16416
Cd200r1	3.86306	1E-05	0.17962	0.49374	0.20506	2.23101	1.87515	2.17055
Igf1	3.86264	4E-09	1.17123	1.31041	0.31585	6.30284	6.35082	5.31902
Vps25	3.86104	0.00657	4.72819	0.70571	0.76203	10.3804	7.37595	13.6715
Kcne4	3.8399	0.03396	1.99382	1.01762	0.51017	3.24916	4.23088	4.19628
Pdpn	3.83827	6.3E-08	9.80233	6.46245	2.00853	42.1216	32.4808	41.5945
Mfap4	3.83341	6.5E-05	24.1413	11.4551	4.01461	116.064	47.8698	82.8102
Slpi	3.83038	1.2E-06	4.48947	4.58273	0.97202	15.1012	27.6159	21.0536
Lyve1	3.82385	0.00138	24.751	15.9849	1.50031	61.5393	62.9246	121.305
Medag	3.8223	1.7E-07	2.07795	1.15214	0.39537	5.21784	7.39159	10.0819
Kif20a	3.81801	0.00592	0.16843	0.28491	0.27469	1.86604	2.34443	1.06448
Lilra6	3.8111	0.01405	0.11979	0.10131	0.06837	0.81649	0.35102	0.95683
Capg	3.8101	3.9E-15	2.45589	2.5048	1.23691	15.5363	11.43	15.5931
Fstl1	3.80857	5E-12	9.75829	7.16866	2.58875	39.9001	41.8583	33.8816

Cd79a	3.80024	0.01027	1.00302	1.11344	0.4294	10.673	3.03108	3.80051
Ccl8	3.79649	0.00156	1.78743	2.11649	0.20405	6.28124	10.7376	10.1041
Syt12	3.79227	0.00691	0.1649	0.13947	0.11295	0.64941	0.77315	0.64845
Ticam2	3.79211	0.00397	0.23253	0.118	0	1.18344	0.7325	0.5715
Hgf	3.79171	1.4E-06	0.87702	0.60202	0.11608	3.56559	2.51414	4.05072
Pi15	3.78792	0.00016	1.55682	0.63605	0.10543	4.94006	4.89067	6.73385
Isg15	3.7836	0.00012	1.4498	2.14588	1.1034	10.3213	12.7454	8.90821
Tdo2	3.78057	0.00525	0.11767	0.03981	0.02687	0.3137	0.34483	0.59771
Csf2rb	3.7785	3.4E-12	1.57214	2.35128	0.80983	12.8592	8.23073	11.3563
Tlcd2	3.76221	0.04481	0.09436	0.07981	0.10772	0.77179	0.553	0.54112
Fau	3.75589	0.00026	31.8281	16.493	7.93231	144.834	93.5144	138.089
Slfn1	3.75552	1.5E-06	0.78401	0.87033	0.11188	4.03767	3.62567	4.09472
Scube1	3.75152	1.8E-15	0.974	0.66644	0.23738	3.87896	3.78422	4.43809
Areg	3.74948	1.7E-05	0.52622	1.46238	0.60074	6.83208	3.35943	8.31433
Esd	3.7384	1.8E-10	2.79494	1.74425	0.74347	11.0318	8.76672	13.7609
Col4a1	3.73148	2E-14	29.7133	33.7009	8.29244	157.672	157.754	166.411
Fscn1	3.72533	1.2E-09	1.51623	2.41091	1.14242	15.0476	11.5299	7.87542
Mastl	3.7206	0.00169	0.17392	0.09361	0.0722	0.6035	0.97295	0.59583
Emilin1	3.71891	1.9E-21	4.34765	4.66296	1.68963	24.2343	19.4356	26.5243
Rpl36	3.7186	7.6E-05	34.8168	13.7676	12.3887	122.597	58.9632	170.032
Psat1	3.71591	2E-05	0.5153	0.41768	0.31865	2.4847	2.84704	3.04311
Cd109	3.7155	0.0006	0.35741	0.12315	0.06045	1.03461	1.39645	0.84589
Svep1	3.71434	1E-07	2.85058	1.73993	0.45935	11.9353	7.32779	10.8132
Myl12a	3.70916	3.7E-06	12.983	6.41955	2.60597	55.1151	30.1852	57.7873
Tnfrsf11b	3.70564	0.00102	0.16206	0.1919	0.074	1.27647	0.59362	1.0356
Nlrp12	3.70303	0.04051	0.12106	0.10239	0.04146	0.26405	0.1685	1.20996
Rap2b	3.70025	4.3E-10	1.16979	0.57999	0.16885	4.08145	2.96495	3.96561
Gamt	3.69214	0.00138	0.37952	0.21399	0.07221	1.34134	1.11213	2.021
Cystm1	3.68529	3.9E-06	2.28344	1.49607	0.69759	10.4639	6.4086	12.0148
Lgals9	3.68203	6.5E-08	4.53055	4.12106	1.85415	24.3164	18.5366	25.7717
Oas3	3.67653	9.2E-05	0.17474	0.31037	0.19949	1.45046	2.02263	1.13095
Rasl11a	3.676	1.8E-05	1.52103	0.70755	0.60774	6.72741	3.45431	8.47355
Tmem88	3.67321	4.6E-05	1.38565	0.53715	0.06591	3.81292	3.04536	5.91253
1500009L16Rik	3.67163	0.00453	0.22968	0.32377	0.26221	1.8091	1.34612	2.32076
Tgm1	3.66945	3.4E-07	0.30385	0.71958	0.20813	2.65101	2.24825	3.50994
Ighv1-69	3.66754	0.01575	2.08177	0	0	3.62626	5.52845	6.82201
Prrx2	3.66229	0.02201	0.13973	0.07879	0.10635	0.81839	0.64832	0.61054
Pld3	3.64628	3.4E-10	4.06084	3.40817	1.06982	20.0049	13.227	20.3707
Rhog	3.64274	8.4E-09	5.59604	4.78743	0.9546	24.9029	16.3514	29.0357
Fbxw10	3.63051	0.02523	0.09077	0.15354	0.05181	0.42622	0.71485	0.33464
Chil3	3.62884	0.00778	10.018	33.3243	6.96421	46.9643	103.527	165.614
Tuba1c	3.62529	1.9E-10	3.32641	1.95376	1.84598	16.5992	14.4183	20.9688
Cd300a	3.62173	4.3E-08	0.50122	0.39131	0.3081	3.78432	1.59586	2.52694
Cd276	3.61726	1.5E-07	0.34957	0.40125	0.28505	2.04234	2.28654	2.55703
Apex1	3.61133	5E-06	1.15617	0.70624	0.07333	4.67003	4.28208	4.68339
Grp	3.60456	0.01131	0.51749	0.07295	0.19692	1.46319	1.64282	1.7665
Hif1a	3.5971	3.6E-11	3.54754	2.46325	0.79583	14.2667	10.7361	13.4022
Olfml3	3.58928	6.9E-10	2.9502	1.84321	0.86315	11.4075	8.52572	15.1941
Cd248	3.58581	1.4E-10	1.71258	0.75441	0.36658	5.79339	6.01174	5.43679
Pcna	3.58488	2.1E-08	3.46322	2.38193	1.30339	14.9187	11.3474	14.4981
Hist1h4h	3.5784	1.8E-05	27.2275	16.3323	5.51118	90.3221	40.7421	89.2252
Hmox2	3.57839	0.00013	3.70789	2.33463	1.45801	9.26075	7.03239	10.3957
Fabp5	3.57705	0.00012	1.35713	0.88795	0.46772	7.57103	4.0707	6.35637
Fam57a	3.57608	0.01577	0.59373	0.39706	0.18915	1.11265	0.83958	1.09726
F2r	3.57038	3.6E-07	16.1298	11.7968	2.2702	58.1847	48.9052	64.4656
Egr2	3.56774	1.7E-05	0.672	0.79571	0.18412	4.51122	3.09164	2.68662

Clec12a	3.5599	5.4E-05	0.33045	0.20962	0.11317	1.76191	0.98046	1.36717
Lamtor5	3.55681	5.4E-05	3.09969	1.83517	0.82568	10.3919	7.87215	12.4433
Spry4	3.55163	1E-10	3.28552	2.22307	0.40918	13.7656	10.08	10.31
Ltbp2	3.5371	3.3E-15	5.88159	6.49428	2.06402	31.0644	29.107	24.495
Nxpe5	3.53611	0.00172	0.1021	0.12089	0.02331	0.47014	0.59838	0.5186
Col5a3	3.53561	0.00012	0.48291	0.7715	0.47473	2.99903	5.73908	2.42873
Atp6v0c	3.53318	3.1E-05	10.0946	6.55179	2.68074	30.0412	22.8581	31.3335
Usmg5	3.53161	0.04293	9.10697	2.26545	1.22313	25.4791	13.3434	34.2326
Dlgap5	3.51565	0.0026	0.0412	0.10455	0.14112	0.52427	0.81503	0.49511
Postn	3.51564	3.2E-08	4.9332	2.92069	1.07842	18.9893	13.864	19.1673
Sgk1	3.51321	1.9E-13	5.30924	3.35603	1.46741	15.9484	20.9079	23.1065
Ndnf	3.51133	5.9E-08	4.09096	4.39191	1.01034	18.7074	11.7035	26.287
2200002D01Rik	3.51126	0.00051	1.46	0.69151	0.6667	3.32605	3.16597	5.40644
Psma2	3.50763	9.3E-05	8.70803	5.81025	1.10457	30.3666	23.1078	35.9876
Vstm4	3.49354	1.1E-09	1.49952	1.26827	0.6467	7.10676	5.59035	8.65406
Csf2rb2	3.48272	5E-06	0.59413	1.07201	0.42957	5.42359	3.80123	4.62408
Olr1	3.47581	7.7E-06	0.51141	0.36766	0.05838	2.15348	1.87327	1.5921
Tnfrsf9	3.47163	0.04789	0.06711	0.28381	0.22984	1.13851	0.9833	0.82471
Psma6	3.46269	7.7E-06	6.3655	3.75616	2.19697	24.9343	18.1111	32.0782
Psrc1	3.46131	0.00069	0.1213	0.48731	0.45004	2.37013	2.15491	2.55889
Gm6377	3.461	0.00134	0.13072	0.16584	0	0.83158	0.59851	0.56222
Gcnt4	3.45605	0.02152	0.07174	0.03034	0.02047	0.23905	0.31532	0.191
Gprc6a	3.45445	0.01111	0.09594	0.1082	0	0.5813	0.21086	0.55021
Rps11	3.44914	2.5E-06	32.5225	16.7007	11.2107	145.035	77.1244	153.81
Hist1h4f	3.44639	3.5E-07	7.2416	8.10057	8.80041	50.3862	27.2103	37.3188
AB124611	3.44528	2.2E-05	0.69434	0.8542	0.43236	4.35988	2.40462	5.84359
Cdkn2b	3.43575	8.6E-09	0.98353	1.44188	0.59883	5.83988	6.19657	6.76845
Lxn	3.43518	0.01915	1.57479	0.87088	1.03718	8.11015	4.70346	6.35153
Dpt	3.43235	0.00044	12.2914	7.16954	1.57254	38.5838	24.1416	38.4996
Pcdhgc3	3.43044	0.00065	0.25334	0.1813	0	1.01541	0.51395	1.1016
C1qtnf5	3.42985	0.03159	1.09214	1.31265	0.26248	6.30366	3.36883	4.19117
Ccng1	3.42261	4.5E-10	9.70072	6.99489	2.85025	41.4261	24.902	49.5592
Cers6	3.42131	4.2E-11	2.35347	1.90314	0.55045	9.96062	8.17493	9.51821
Gpr141	3.4209	0.00055	0.15963	0.20252	0.12149	0.78988	0.99405	1.22063
Mrps18c	3.41258	0.00065	1.52795	1.07693	0.67835	6.53175	5.03715	8.27569
Tmem45a	3.41239	0.00027	0.71731	0.20223	0.03412	2.08249	1.40133	1.93439
Mxd3	3.41133	0.0297	0.13167	0.11137	0.05011	0.90416	0.41801	0.46746
Lsm5	3.41056	0.00034	1.03793	0.78032	0	3.28406	2.70357	5.4799
Capns1	3.40435	9.9E-06	11.3573	6.36228	2.31527	39.8795	27.5271	45.5559
Chsy3	3.40078	1.1E-07	0.46744	0.52714	0.24903	2.62444	2.14597	2.85938
Col8a1	3.40046	3.2E-06	0.75101	0.34784	0.28579	2.51348	2.96053	2.65153
Cd8b1	3.39896	0.0478	0.06159	0.26046	0.14062	1.41801	0.49632	1.05961
Tgfb1	3.39376	1.2E-13	4.56185	3.65974	1.72318	23.1887	15.6532	20.7202
Slfn2	3.38536	2.7E-05	5.10481	5.33891	2.3812	19.6882	18.5379	20.6409
Slc7a11	3.37665	9.7E-05	0.26941	0.13672	0.05272	0.92342	0.86278	0.83241
Fmod	3.3676	0.02028	0.6907	0.28041	0.06308	2.5611	2.024	0.95064
Rsad2	3.36477	9.3E-05	0.50649	0.49978	0.3132	2.455	3.89606	1.38316
Loxl2	3.36402	5.7E-08	2.95728	2.68702	1.04175	11.4878	12.3799	9.35881
Gm15832	3.36336	0.01259	0.44871	0.08434	0.05692	1.60099	1.42446	1.14367
Socs3	3.36014	0.00049	3.98082	5.15724	3.64283	27.0478	23.6546	33.0531
Ube2l3	3.35849	0.00526	3.94779	2.44214	0.98703	9.29085	6.44156	10.0235
Ptges	3.35587	2.2E-07	1.46382	1.72937	0.50398	6.46171	6.40026	7.69038
Nme2	3.35103	1E-06	10.374	6.46513	2.31517	34.168	27.0282	42.4947
Nit2	3.3506	0.00014	0.71357	0.24141	0.40731	2.8967	2.30015	2.6307
Cdca3	3.34843	0.00286	0.19122	0.2965	0.10915	1.33233	1.26075	1.01826
Kcnk13	3.34762	1.5E-06	0.4696	0.59577	0.26805	2.18728	2.77372	3.0778

Itga5	3.34742	1E-09	5.49013	4.05215	1.33802	18.3511	20.4265	21.8658
Grn	3.34202	4.2E-09	9.96458	8.36116	2.91144	44.0775	36.4614	39.6761
Hist1h4d	3.34136	3.9E-06	42.9961	29.4854	16.45	122.508	75.4265	126.048
Cyb561d2	3.33676	3.8E-05	0.69405	0.19567	0.10564	1.76617	1.66097	2.35025
Atp5l	3.33669	0.00301	20.4337	6.13859	2.54942	42.3505	23.4769	50.9532
Capza1	3.3314	5.8E-08	5.55611	4.2702	1.4892	20.3005	15.9629	21.7501
Ecscr	3.33106	1.8E-07	3.31642	3.31079	1.42753	13.1762	9.6387	13.2732
Eno1	3.32568	2.5E-08	5.34883	3.57607	1.53141	23.7862	17.8137	24.6787
C1qtnf6	3.32369	3.7E-10	1.78721	1.18997	0.56433	6.77349	6.74147	6.94707
Tpx2	3.32181	3.8E-06	0.19529	0.51204	0.28983	2.10616	2.26049	1.72793
Commd1	3.32094	0.00061	0.76935	0.46851	0.08783	1.52893	1.03707	1.37405
Mmrn1	3.31981	0.00012	1.15184	0.39495	0.15993	3.15931	2.63411	3.41754
Cpz	3.31512	0.00853	0.16274	0.27528	0	1.08461	0.71533	0.89995
Adssl1	3.31238	1.4E-08	1.12884	1.07928	0.67236	4.93628	4.71018	7.5995
Fgr	3.30131	7.6E-09	0.70933	1.07988	0.40489	4.44094	3.06592	5.83057
Sparc	3.30124	4.3E-10	52.0103	54.839	14.6718	243.098	205.209	222.808
Fkbp10	3.30024	5.3E-08	2.42737	1.86878	0.60396	9.05047	9.46134	9.3568
F13a1	3.30022	2E-07	2.51196	1.49507	0.84968	6.42602	10.82	10.0991
Cfb	3.29289	1.8E-05	4.4618	4.01983	2.47299	20.0399	19.5647	15.8134
Gpr182	3.28312	2E-08	1.8249	1.3481	0.39557	6.73204	4.73842	8.76221
Ckap2l	3.28189	9.4E-05	0.26205	0.39402	0.1662	1.7641	1.91973	1.45507
Ier3ip1	3.2799	0.00479	2.98993	2.08017	0.88086	6.34042	4.59278	7.54611
Rpl23	3.27829	1.2E-05	31.9424	15.3194	6.83361	97.9234	67.7072	110.045
Gm13889	3.27684	1.9E-08	5.90545	7.71408	3.8952	28.4253	34.1296	45.3167
Serpinb6b	3.27052	0.0002	4.77446	3.79236	0.75834	20.9788	10.9799	18.7412
Tubb2a	3.26834	1.4E-09	7.78445	5.80364	1.44821	31.0588	22.4734	32.6432
Clec4a3	3.2683	0.00138	0.41298	0.81501	0.31431	3.66986	2.52122	3.10142
Gpr85	3.26508	0.01797	0.13422	0.01892	0	0.20331	0.36054	0.31156
C1ra	3.2641	4E-05	5.36199	4.2241	1.67898	19.8822	13.1088	24.5497
Prkcdbp	3.26389	5.8E-05	3.73704	2.2311	0.94108	11.1878	8.57553	12.4265
Cnot9	3.26384	0.0097	2.38729	1.57492	0.89936	4.54174	3.79514	5.00685
Ano5	3.26363	0.01031	0.08176	0.01976	0	0.15569	0.20536	0.22009
Rpl36al	3.26229	0.00019	7.42582	3.4258	2.38907	24.091	14.4905	31.3032
Phlda1	3.25816	2.3E-05	4.5151	2.97796	1.74968	13.9793	9.1342	17.2734
Arpc3	3.25738	2.1E-06	4.39823	3.02071	1.17032	16.4096	11.0957	20.6714
Anxa2	3.256	2E-09	16.9407	12.5268	5.30999	66.39	48.8175	75.5509
Akr1b8	3.25591	0.00038	3.83874	0.8761	0.83473	11.1861	7.54393	11.5813
Cox7b	3.25528	0.01077	7.97511	1.97266	0.42945	17.1399	11.2441	23.1144
Ost4	3.2548	2.4E-07	5.38547	4.31201	1.88541	18.7947	11.6783	23.0605
Hist1h3g	3.24979	0.04925	0.21391	2.17101	0.73259	7.51689	3.13411	8.58693
Fzd1	3.24856	4.4E-14	4.41781	2.76096	1.41612	19.0268	12.7067	16.7772
C330027C09Rik	3.24176	0.00016	0.17664	0.16298	0.10999	0.97292	0.89406	0.7104
Aurka	3.23873	0.00026	0.14435	0.36627	0.0824	1.13697	1.11037	1.35999
Ccl21a	3.23268	0.01182	11.9684	5.68289	2.27719	31.3592	19.1511	30.5336
H2-Eb2	3.22537	0.00846	0.45483	0.13989	0.0472	1.55322	1.30252	0.60974
Cotl1	3.22399	2.3E-07	7.95484	10.0195	6.27197	56.8308	36.8526	55.5591
Atp5k	3.21815	0.00361	5.93101	2.38276	1.1849	15.9911	9.01592	27.5749
Ccne1	3.21781	0.004	0.17156	0.26118	0.11751	1.06023	1.20655	0.92763
Cst3	3.2152	0.00031	40.9793	29.4118	7.24794	171.702	81.3953	156.632
Gas2l3	3.21448	0.00011	0.21657	0.32055	0.04636	1.35315	1.11059	0.83168
Mcm4	3.21285	5.2E-07	1.32336	0.77489	0.58106	5.50469	4.53049	5.17068
Rps25	3.20445	3.6E-06	14.7319	7.11998	4.28094	43.7943	29.9106	55.5809
Asah1	3.20416	4.2E-06	6.81664	3.4638	1.13807	20.1607	16.4816	22.7356
Prelp	3.20292	0.00037	18.8992	13.0142	2.88441	44.9914	24.0629	37.1557
Rpl34	3.20157	4E-07	11.3548	6.36729	3.03888	36.8932	23.2795	46.4103
Siglec1	3.20099	3.4E-07	0.95903	0.94091	0.56931	4.36955	5.78922	3.92844

Clmp	3.19998	3.7E-06	1.07433	0.45432	0.22712	3.08582	2.88583	3.76508
Ctsk	3.19609	1.2E-06	3.68492	2.14588	0.96548	11.2363	14.2943	11.0363
Col4a2	3.19163	4.5E-14	27.4468	30.4385	10.0034	119.668	116.216	136.08
Tmem254a	3.18617	0.02622	3.42088	1.32266	0.66948	6.48444	4.86903	5.40498
Rps15a	3.18615	5.6E-07	3.75839	1.57707	0.94792	14.1306	7.88659	15.3238
Cdh5	3.17859	0.00025	25.904	19.0418	3.59728	89.0204	59.5899	97.1904
Arntl	3.1746	0.00056	0.32196	0.46083	0.08482	1.56056	1.8325	1.46087
Tnfrsf10b	3.17089	4.1E-08	1.03695	0.77958	0.39459	4.32806	2.86982	5.82868
Pou2af1	3.15981	1.3E-05	1.14665	0.93951	0.16363	4.55919	4.06888	3.46403
Itgb2	3.15867	4.9E-07	4.12994	7.82136	2.15239	29.5013	21.2886	29.3235
Lrp12	3.15601	1.5E-09	0.71888	0.7938	0.31915	3.85951	3.10136	3.35372
Bcl2a1a	3.15475	0.02283	0.66914	1.13189	0	5.13532	1.38892	4.56831
Sla	3.14886	0.00026	1.50141	2.03515	0.70377	4.96408	4.90231	6.26425
Vgll3	3.14853	7.1E-05	1.75966	0.88367	0.15694	5.54731	4.71334	3.89687
Cd300lf	3.14368	0.00211	0.67408	1.03522	0.68853	3.29954	5.17217	5.02832
Cyba	3.14012	1E-08	9.84582	7.80696	3.01073	44.0482	26.0183	43.2121
Gpx7	3.14012	0.00016	0.99203	0.59932	0.48536	4.12151	3.21848	4.17974
Ctsl	3.13739	1E-08	15.1998	8.19159	3.12127	44.6144	43.0472	48.8229
Fkbp7	3.1346	0.00058	1.8452	0.62426	0.21065	4.99363	3.64982	5.4421
Naa38	3.13383	9.9E-05	1.64202	1.0416	0.46864	7.15065	3.75917	7.90328
Rac2	3.12923	8.1E-05	1.5945	1.78117	0.78994	8.6947	5.20143	9.88353
Spi1	3.12861	4.6E-06	1.22009	1.5479	1.08334	8.06997	5.95888	8.66294
Arg2	3.12086	0.01391	0.8315	0.48688	0.21906	1.7439	2.15547	4.66371
Slc37a2	3.11619	8.2E-06	0.38498	0.34887	0.32963	2.4825	1.7728	1.85861
Epha3	3.11499	0.00081	0.54877	0.27302	0.0737	1.72112	1.47803	1.42808
Cmc1	3.11402	0.00428	2.34399	0.85909	0.26759	4.26055	3.66335	4.28874
Rab5c	3.11251	2.3E-06	10.9072	7.99277	2.56639	37.8097	30.0713	44.2016
Yif1b	3.10816	0.00015	2.27841	1.24691	0.38251	7.91729	4.95836	8.94873
Inhba	3.10445	0.00038	0.59969	0.46819	0.21065	2.5993	2.02768	2.41871
Atp5j2	3.09896	0.00491	6.60361	2.3847	1.35528	18.1169	10.1647	25.8345
Psmb3	3.0984	1.9E-07	3.09754	1.51145	0.99738	10.4424	7.15681	12.1353
Gla	3.09667	8.7E-08	0.64342	0.82812	0.19162	3.44073	2.82819	2.93357
Lcp1	3.09663	4.4E-07	4.99743	4.80385	1.48113	22.7322	14.8485	21.1401
Ctsz	3.08667	1.7E-09	12.8037	12.7055	5.64412	67.4754	45.5995	58.3676
Rpp30	3.08319	0.00535	0.7908	0.33442	0.27083	2.10818	1.85388	3.69286
Ppia	3.07866	3.7E-06	19.3454	12.3101	4.75583	71.5727	44.0048	79.6071
Cfp	3.07749	1.1E-07	2.42024	1.3795	0.8409	10.4878	5.93586	8.70705
Nop10	3.07446	0.00142	1.64721	1.46284	0.65816	8.18371	3.19784	9.78376
Ly96	3.07265	0.01588	0.26838	0.52964	0	1.73445	1.11413	1.83225
Bank1	3.07223	0.01846	0.41484	0.29927	0.22286	2.3508	1.09049	0.59975
Tubb5	3.07221	1.3E-09	18.0369	15.3482	6.98904	82.4692	57.4002	78.4725
Nfam1	3.07102	1.3E-05	1.38906	1.89413	0.38835	6.68132	4.50658	8.70914
Hist1h1b	3.06945	0.00266	2.21275	2.91124	2.52609	13.9281	15.4	15.2075
Clcn5	3.06676	1.3E-13	0.83745	0.55315	0.20031	2.66261	2.61179	3.10371
Rrm2	3.06654	0.00078	0.42851	0.94787	0.48918	3.13546	4.51562	2.83545
Rpl32	3.06604	1.7E-06	37.4645	18.3704	7.36289	116.714	70.318	134.195
Napsa	3.06401	5.6E-05	8.30966	11.0069	1.99919	32.3509	20.3759	41.2966
Tnfsf8	3.06292	0.01086	0.14395	0.20292	0.02739	0.77043	0.70306	0.58967
Cfl1	3.06046	1.9E-11	32.166	26.8445	9.92218	125.279	96.9021	139.645
Kctd12b	3.06	0.00061	0.50267	0.31493	0.06376	1.5228	1.22752	1.86081
Gpr35	3.05771	3.7E-06	0.60401	0.47893	0.17239	3.24789	1.5349	1.97936
Blvrb	3.05376	0.00034	1.67947	0.6556	0.22123	4.10939	3.54915	5.66244
Mrc2	3.05356	7.5E-12	4.62573	4.00496	1.07802	18.8308	13.8358	17.1319
Dusp6	3.0526	1E-08	10.4208	5.69165	2.57322	38.3579	21.0121	37.7356
Birc5	3.04691	0.00868	0.10739	0.24979	0.3065	1.18748	1.37682	1.18776
Ccdc80	3.04551	8.4E-05	2.93428	1.58609	0.60447	9.81146	8.84096	6.85028

Abcd2	3.04413	4.5E-06	1.02459	0.60102	0.1132	3.20404	2.27605	3.37116
Sec61b	3.0441	9.7E-05	4.59922	2.43121	1.80486	15.5875	10.1607	20.1938
Slc6a14	3.04372	2.1E-05	3.00558	2.3171	0.48583	10.1043	6.66405	12.8129
Pik3cg	3.03787	1.1E-07	1.1037	0.99041	0.23049	4.37924	3.43145	4.23435
Apoc1	3.03612	0.00591	1.81356	0.55777	0.37643	4.59501	3.86503	6.07816
P2ry14	3.03466	0.02731	0.84825	0.78687	0.46857	2.20497	1.68386	2.24173
Kif18b	3.02812	0.0105	0.07671	0.21627	0.11677	0.8366	0.86171	0.50277
Sh3pxd2b	3.02632	2.3E-14	2.13551	2.58593	1.13841	10.824	9.73134	11.3758
Tgfbr2	3.02372	2E-11	10.197	8.0677	3.06943	37.8396	30.2814	42.0621
Heyl	3.02304	1.4E-06	2.41161	2.10014	0.42826	8.46387	8.91218	7.01018
Srgn	3.02126	3.8E-06	6.03094	5.10087	0.94965	19.127	17.4291	23.5118
Actb	3.01941	1.9E-08	306.811	235.331	85.7836	1166.56	833.307	1216.26
Itga2	3.01485	2.1E-06	1.34384	0.91661	0.14846	4.26802	3.06461	4.91875
Adam12	3.01007	0.00016	0.29409	0.24071	0.22743	1.299	1.93206	0.86269
Mfsd2a	3.01003	0.00104	0.28416	0.44634	0.13903	1.37735	1.24905	2.22824
Ndufa11	3.00275	0.00059	1.75055	0.929	0.23511	5.61509	2.56488	6.18653
Ifi47	3.00225	0.02564	0.96028	1.46819	1.09626	4.65454	4.95153	4.44798
Piwil2	2.99915	0.00983	0.07436	0.07862	0	0.29286	0.28601	0.27415
Prdx1	2.99202	9.2E-05	12.8167	6.54977	3.11821	41.1003	28.564	42.099
Pfn1	2.9852	8.4E-08	35.6982	23.9435	18.903	157.767	108	168.62
S1pr3	2.98342	6.7E-09	3.70728	2.36028	0.90691	11.8726	11.2666	11.7984
Samsn1	2.98118	0.00073	0.30218	0.37484	0.13799	1.59892	1.28396	1.48536
Ctsc	2.98048	8.6E-07	5.61424	6.51745	1.99279	23.5919	19.6431	28.9112
Nid1	2.98011	1.5E-07	10.575	7.42363	2.51797	35.6057	29.0076	34.5678
Plin2	2.97817	1.4E-09	6.00768	6.313	1.98923	32.2553	21.0724	20.3905
Sdcbp	2.97812	1.5E-08	6.52222	4.47577	1.67644	22.6041	16.9125	22.5332
Cd84	2.9778	7.8E-07	0.99027	1.66278	0.69825	6.37926	5.89972	6.28744
Plac8	2.97676	3.4E-06	1.31958	2.3292	0.91696	8.96839	7.14527	8.78958
Ncapg2	2.97558	7.6E-08	0.4979	0.5381	0.18947	2.0279	2.4824	1.86959
Fam177a	2.97512	0.00379	5.66545	2.89921	1.49464	9.41801	8.21375	12.1107
Psmb7	2.97178	3.5E-06	2.56311	1.98718	0.73152	9.47944	6.68937	11.8116
Col6a3	2.97105	5.9E-13	7.62737	7.89759	3.3993	31.1463	35.68	31.4253
Pld4	2.97063	2.8E-07	2.21648	2.49955	1.15974	12.8138	8.22039	10.5169
Rab20	2.9648	2.6E-05	1.18109	1.33193	0.29963	4.5719	3.70138	5.10686
Rnf152	2.96363	0.0008	0.18199	0.10865	0.03666	0.64213	0.56467	0.41221
Rpl28	2.96159	8.1E-08	20.5013	12.8053	5.83694	71.9117	43.6027	79.0177
Nme1	2.96077	0.00012	1.58515	1.59496	0.65521	4.02381	3.94432	4.34375
Bak1	2.95831	0.00064	2.20091	1.7652	1.16964	9.1505	6.92207	9.07771
Icam1	2.95638	4.4E-09	11.6686	11.0562	3.27642	53.1186	35.8815	55.5038
Srm	2.95423	4.5E-06	1.82789	1.40106	0.71731	7.06024	5.56565	8.18953
Prcp	2.94978	2.2E-05	2.1753	2.19934	0.62797	9.81665	7.16209	8.23466
Zfp385a	2.94637	2.6E-06	1.47512	0.91043	0.40962	5.36248	3.62167	5.45457
Ntm	2.94544	0.04457	0.03392	0.02869	0	0.0959	0.09111	0.16672
Snrpf	2.93745	0.0002	3.92508	1.88419	0.72663	9.89818	6.91671	9.99457
Scel	2.9369	3.5E-05	3.14132	1.74595	0.61477	10.187	5.91765	11.2011
Ppp1r14b	2.93503	3.3E-11	6.36083	5.04364	3.51732	20.8352	17.9843	24.183
Spcs1	2.93375	0.00017	4.96272	2.99813	0.53957	13.6023	9.17569	16.6502
Ghrl	2.92978	0.01819	0.55694	0.47105	0.21193	2.64327	1.29203	2.6616
Rps28	2.92857	3E-05	20.1517	10.2369	5.34178	71.783	36.4275	81.4225
Ecm1	2.92468	3.4E-10	8.91275	6.52702	2.19214	28.7126	23.9678	34.8173
Tspan8	2.92461	4.9E-07	5.64109	5.68475	1.37019	20.8706	18.4209	24.2874
Pappa2	2.92388	0.00017	0.11535	0.15009	0.08103	0.66124	0.49402	0.69057
Mpp1	2.92353	5E-07	1.48725	0.95426	0.3708	4.81621	3.86978	5.41995
Ptgir	2.92051	0.00024	0.24656	0.48659	0.28148	2.30721	1.32462	1.84042
Sell	2.92012	5.4E-06	0.88077	0.73187	0.33516	3.77296	2.35459	4.01299
Dynlt1f	2.91765	0.02371	5.77226	3.06327	1.03367	15.2236	6.13579	16.3196

Naip2	2.91555	5.7E-09	0.81047	0.84489	0.32276	3.60862	2.74778	3.25813
Car2	2.91447	1.9E-06	2.14956	1.84996	0.77493	8.54546	5.99512	10.1955
Rrm1	2.91145	2.7E-09	1.21711	1.32133	0.49772	4.90883	5.00386	5.58092
Crip1	2.91142	5.4E-07	6.53656	4.53338	1.84067	22.099	15.6748	27.204
Ppic	2.90702	3.6E-12	8.16782	9.25099	2.91895	37.2662	31.6876	34.6797
Rps13	2.9045	5.7E-05	10.8614	7.14816	2.32489	32.7597	19.9671	36.0102
Nsmce1	2.90157	1.7E-05	1.78689	1.10443	0.47075	5.57979	4.32998	7.31961
Serpinb9	2.89893	6.6E-05	3.65978	2.4763	1.04451	11.8789	9.3265	14.606
Cd34	2.89696	2.5E-07	6.87045	5.59905	1.51148	20.7375	17.1052	19.9935
Disp2	2.89688	4.1E-07	1.28092	1.13189	0.20734	4.67392	3.16534	4.87112
Ndufa4	2.89632	0.02169	9.98695	2.40538	0.52853	18.5935	11.9678	26.1712
Fam111a	2.89482	0.03282	1.15141	1.51694	0.63195	5.16505	3.52005	4.73463
1110012L19Rik	2.89288	0.00117	0.75141	0.84737	0.38125	3.6927	2.69121	3.76197
Lamp1	2.89244	4.8E-05	37.2862	24.8072	7.71804	102.901	70.3403	104.537
Dscr3	2.88774	1.2E-05	1.56792	1.09458	0.59665	4.43322	2.84427	4.70994
Fignl1	2.88701	0.0086	0.0755	0.2235	0.06464	0.60612	0.81585	0.49484
Rnf125	2.88683	0.00051	1.23244	1.1582	0.23449	3.11131	3.61151	5.3289
March1	2.8835	2.1E-07	0.63908	0.8216	0.32101	2.73369	2.37839	2.11523
Cox7a2	2.88173	0.00075	8.1275	2.94604	1.65686	21.9249	12.7589	27.1096
Cks1b	2.87719	0.00073	0.89229	1.09772	0.27781	3.93178	4.27865	3.18987
Serinc3	2.8752	8.9E-05	55.8967	36.6745	8.14621	152.401	123.581	183.682
Slc15a3	2.8741	8.5E-08	0.76517	1.16771	0.39878	4.86781	3.63143	3.81572
Itgb6	2.87324	5.1E-10	1.78539	1.65128	0.45457	6.70833	5.40124	7.09249
Ndrg1	2.8715	2.6E-05	2.2166	1.66385	0.58517	4.98587	4.34383	5.92457
Gm2a	2.87056	0.00021	4.97964	4.324	1.08642	19.0545	12.1439	18.6755
Ccr2	2.86996	4.6E-05	1.27008	1.57703	0.63242	7.80161	4.385	5.81142
Ada	2.86941	0.00097	0.54748	0.46305	0.15625	1.35447	2.20593	2.39213
Ifi207	2.86555	3.2E-07	1.67929	1.79758	0.89863	7.23345	7.57364	7.24428
Maged2	2.86383	1E-08	1.29717	1.35948	0.67604	5.80906	4.31765	7.18487
Hist1h2bc	2.86275	1.5E-05	11.9136	7.97708	3.6835	44.2117	29.1837	42.5996
Mrps36	2.85659	0.01746	1.72065	0.4851	0.24554	3.34472	2.3635	5.57986
Ifi30	2.85383	7.3E-09	11.7434	14.1103	5.74557	66.0125	42.8791	53.2965
Mgst3	2.85129	0.00026	3.33045	1.83094	0.38021	9.98841	4.94073	12.1418
Ctbs	2.84792	0.00039	1.15805	0.82877	0.13559	3.09443	2.32761	3.62468
Dtx4	2.847	2.5E-11	1.25163	1.67502	0.57878	6.94988	5.01415	5.91882
Hist1h4c	2.83574	0.00013	26.3607	18.9707	10.0312	79.0169	44.0442	87.1425
Cd80	2.83497	0.00223	0.37587	0.54499	0.4904	2.76537	2.55695	1.80364
Rhoc	2.83454	7E-09	11.0079	8.21218	3.0289	37.3151	32.4228	37.6457
Cyp4f18	2.8332	5.3E-05	0.98477	1.16606	0.84317	5.07158	3.31861	5.56679
Sdk2	2.83253	1.1E-06	0.17714	0.18977	0.02022	0.64394	0.64885	0.61918
Sema5a	2.83139	9.2E-07	1.96888	1.25787	0.4534	6.20519	5.18146	5.99518
Cd44	2.83086	1.2E-10	5.70186	5.66619	2.61602	24.7543	18.3281	28.0388
Slc35f6	2.82908	6.5E-07	2.20538	1.38324	0.36775	6.87619	5.19186	6.76018
Rps16	2.8267	3.1E-06	39.7528	23.7369	9.63646	116.362	74.6829	127.966
Cd151	2.82285	1.3E-07	8.7972	5.89041	2.16836	30.4662	17.1861	32.9509
E2f8	2.82245	0.00402	0.18459	0.22709	0.15326	0.6507	1.09412	0.72863
Csf3r	2.82057	0.01181	1.26541	1.71242	1.06901	3.8488	2.87381	16.0274
C1galt1c1	2.82029	0.00751	2.01712	0.90634	0.50373	5.38504	4.10993	7.17821
Degs1	2.81996	2.3E-06	5.46404	3.60767	1.20731	17.2363	12.9385	18.6856
Tex30	2.81922	0.01239	0.42681	0.32088	0.21656	1.72398	0.97278	1.43752
Mki67	2.81751	3.6E-05	1.22558	2.05779	1.31622	8.61368	9.46403	6.6194
Lamb3	2.81467	1.1E-07	2.59548	2.30707	0.64167	9.16488	6.61261	10.9975
1110059G10Rik	2.81281	0.0002	0.59787	0.2988	0.15512	1.81118	1.21443	2.18186
C1s1	2.81272	0.00116	8.55286	7.28324	2.03278	25.4857	17.1934	25.4213
Klhdc8a	2.81021	1.8E-06	0.66326	0.48859	0.34195	2.33337	2.27276	2.85714
Hcn1	2.80681	7.2E-05	0.10585	0.03837	0.01151	0.26876	0.17356	0.32211

Col3a1	2.80166	1E-07	18.582	34.864	15.4134	120.14	131.27	104.506
Vsir	2.79388	8.4E-07	6.35756	8.02716	2.91145	14.9773	11.3035	14.3569
Mrpl30	2.79347	1.3E-05	0.96264	0.7184	0.32322	3.48233	2.53051	3.94325
Snrpd2	2.7922	0.00171	3.40908	1.52327	1.24832	10.7562	6.45572	13.8065
Gja1	2.78797	3.1E-05	6.53042	2.88606	1.00745	15.6128	11.594	20.1472
Vcam1	2.78017	1.5E-06	3.11439	2.45244	0.75603	10.9747	9.33613	9.04751
Rpl22l1	2.77666	0.00011	6.13803	3.56427	1.25502	17.5955	13.5572	16.7371
Slc29a3	2.77502	8.8E-05	1.12724	1.17851	0.42895	5.82425	3.3147	4.16856
Dpm2	2.77405	0.00111	1.66827	0.86227	0.21161	4.4081	3.1912	5.27713
Polh	2.77385	2E-05	0.81774	0.66156	0.24353	3.16659	2.47441	2.79625
Gpc4	2.77341	0.00032	2.51514	1.54445	0.39333	6.80528	5.60345	8.15751
Rab8b	2.77118	3.1E-05	4.59293	3.1991	0.66092	14.6505	10.7729	12.5532
Upp1	2.77091	0.0015	0.83839	0.43333	0.15952	3.10422	1.84259	1.90793
S100a14	2.7698	0.00087	1.00487	1.11829	1.26792	4.48615	5.0369	8.4058
Pdgfc	2.76928	3.5E-05	0.32306	0.28842	0.10245	1.00045	1.30171	1.19103
Srxn1	2.76884	2.4E-07	2.75308	1.58887	0.62859	7.39827	6.80997	9.31381
Dclre1b	2.76779	2.5E-05	0.57595	0.26654	0.08684	1.11272	1.09067	1.35324
Hsd17b12	2.76434	1.8E-05	4.66744	3.11318	0.77976	12.7601	10.8696	15.6373
Gm10521	2.76259	0.00123	0.31936	0.39016	0.04051	1.24697	1.06583	1.48262
Hsd17b10	2.75903	0.00063	2.81472	1.54742	0.80332	8.3564	5.05201	11.7028
Rps18	2.75785	1.6E-09	16.1702	10.7613	6.19517	54.6289	41.7973	63.564
Gm14636	2.75203	0.00119	0.40398	0.22044	0.01488	1.14485	1.0215	0.7153
Eif2s1	2.74969	0.00021	2.97752	1.54277	0.58184	7.26488	6.50464	9.33988
Slc39a1	2.74928	3.1E-16	10.4706	9.05192	3.30811	37.2444	30.6828	41.8776
Cmtm3	2.7486	6.8E-08	2.35532	1.73338	0.5238	8.43256	6.22971	7.09196
Nipal1	2.74661	0.01624	0.14554	0.17585	0.04747	0.37792	0.74636	0.69838
Ppa1	2.74043	5.4E-05	1.38486	0.69212	0.4671	4.06182	3.41253	4.69292
Cd300c2	2.7384	4.4E-06	1.28947	2.42358	0.57247	8.42034	5.82539	7.27748
Psmg4	2.73726	0.01581	1.31895	0.97611	0.75286	6.59283	3.26112	4.9976
Tnfrsf1a	2.73674	7E-09	10.364	9.18315	2.41462	33.127	26.2127	42.3674
Ctps	2.73589	6.6E-14	3.0299	3.09846	1.69803	13.4177	10.5939	14.8484
Was	2.73518	9.2E-05	0.84394	0.77868	0.26276	3.74201	2.22014	3.23703
Arpc1b	2.73515	2.1E-14	7.57129	6.22243	2.97627	27.1777	21.1925	29.7706
Eif3i	2.73255	2.5E-07	2.09716	1.31466	0.78866	7.11555	5.20557	7.96396
Uchl3	2.73153	0.00125	1.86805	0.91471	0.33672	5.53994	3.81744	5.31616
Hist1h4i	2.73036	0.0286	1.00509	1.16887	0.28685	3.6538	3.31345	2.47028
Tnfrsf1b	2.72881	2.2E-07	3.22959	3.96578	1.14704	15.77	10.8484	13.1705
Rab3il1	2.72522	2.2E-06	0.64336	0.76521	0.41314	3.49605	2.69539	2.76717
Tmem97	2.72257	0.00463	1.39497	0.84275	0.91001	5.23213	4.6231	5.27884
Ace	2.72031	2.7E-05	46.2821	36.646	13.7464	140.961	103.718	160.287
Slc39a10	2.72017	2.5E-05	1.37868	0.98862	0.32505	4.54885	3.42526	4.49342
Snhg6	2.71893	0.00015	1.11582	1.01115	0.36395	3.81488	3.91203	4.37482
Pirb	2.7123	2.1E-06	1.26659	1.60689	0.93287	6.84476	6.64494	7.48122
Extl2	2.71145	0.00096	1.03341	0.38099	0.15125	2.00681	2.11591	2.99576
Il4ra	2.70831	1.8E-13	6.78717	7.99289	3.37032	25.1436	26.1878	34.547
Pdgfra	2.70511	1.8E-06	11.8819	8.31551	2.09084	33.7096	29.4648	33.1072
Sdc3	2.70509	2.4E-09	7.06116	5.99801	2.66381	18.9148	16.8037	20.0785
Aqp1	2.70443	0.00077	40.6447	29.1422	4.76307	106.003	73.1538	139.945
Tmem43	2.70426	1.7E-08	4.16111	3.07218	1.12854	12.8007	10.1726	15.9339
Fermt3	2.70419	3.1E-07	2.11838	2.20975	1.45101	10.1182	7.94062	10.4998
Lpl	2.70218	4.5E-06	12.4466	4.5262	2.72545	22.8917	25.4487	35.2498
Btk	2.70206	0.00062	0.40607	0.63784	0.36424	2.91725	2.06116	1.97231
Mrps16	2.69983	0.00201	3.0661	0.95541	0.92113	9.28849	4.61063	8.99005
Gm20390	2.69879	0.00339	2.67285	2.80632	1.47306	13.5139	13.2341	15.4792
Cd36	2.69715	0.00331	35.5428	19.6034	3.92107	87.1872	57.8362	104.052
Serpina3c	2.69714	0.00409	0.45401	0.34908	0.23559	1.57542	1.2397	2.23168

Vsnl1	2.69556	0.02352	0.61395	0.31955	0.10783	1.63101	1.03795	2.28279
Apip	2.69502	0.03321	1.64408	0.77252	0.20854	4.09515	2.60962	5.46251
Hpgds	2.69392	0.00019	0.40753	0.36622	0.20354	1.51236	1.6607	1.48155
Ndufs5	2.6923	0.00097	5.20412	3.68293	1.3337	13.3845	8.16965	16.358
Crispld2	2.69224	0.0003	8.87181	5.88646	1.74623	15.6972	21.2772	24.5775
Snai2	2.69213	0.0043	1.16927	0.49447	0.10268	2.91549	2.04265	3.058
Calcrl	2.69003	0.00423	31.8615	26.7422	3.43869	107.948	67.9276	84.6232
Lypla1	2.68948	0.01792	1.75133	0.85415	0.48159	3.51635	2.80952	3.84356
Col5a1	2.68709	9.1E-06	5.33609	6.50589	3.78276	23.9285	33.1966	19.2933
Slc33a1	2.68484	0.00015	2.03373	1.17576	0.61716	5.00679	4.24338	5.23043
Serpinh1	2.68464	0.00189	24.7879	24.6333	5.63399	101.891	64.2017	78.382
Slfn8	2.68329	0.00026	0.60281	0.71757	0.40781	2.82719	3.63108	1.93886
S1pr2	2.68329	2.6E-06	1.08181	0.75283	0.29703	3.83314	2.53809	3.54512
S1pr1	2.683	0.00028	19.6409	11.8186	3.38899	53.3595	39.4318	58.4678
Snrpd3	2.6816	0.00038	1.25011	0.5624	0.45547	3.44867	2.689	4.53245
Igha	2.68046	0.00564	71.8582	36.9465	5.19794	174.731	128.76	155.632
Gusb	2.68026	3.3E-09	2.77146	2.68377	1.07756	11.3527	8.47451	11.1059
Tmem167	2.67985	2.5E-05	1.25085	0.82206	0.19297	3.10317	2.95962	3.83596
Zfp703	2.67756	4.1E-05	4.68536	2.0725	1.2911	11.0125	7.85127	12.1332
Rab32	2.67756	6.5E-05	1.21321	1.31929	0.49465	5.01419	5.01532	4.25971
Tes	2.67481	0.00011	2.39836	2.31828	0.81488	6.66016	4.89457	6.90045
Nabp2	2.67427	0.00011	1.53019	1.35583	0.49911	3.62016	2.88258	4.5369
Adgrg2	2.67215	0.00351	0.39802	0.13173	0.03951	0.92268	0.62121	0.86483
Calu	2.67038	4.2E-05	14.7104	9.59227	2.59393	39.5675	36.8073	38.5939
Dhfr	2.66962	0.0016	0.22446	0.13143	0.13798	0.60675	0.92338	0.77799
Cxcl16	2.66715	6E-07	5.17005	6.34175	1.96739	21.4142	14.2421	17.5864
Polr2j	2.66143	0.00431	1.98201	1.46681	0.47139	6.65479	3.08551	8.52481
Ckap4	2.66048	8.6E-12	6.15028	5.37686	2.53168	20.477	23.1781	21.6565
Plpp1	2.65978	0.00125	9.32928	5.76435	0.70152	21.6282	16.9432	27.689
Icam2	2.65528	2E-05	4.50931	3.36759	1.25958	17.3518	9.98077	14.6985
Mif	2.65501	0.00617	8.5732	4.04386	2.82323	27.6699	16.7887	29.5804
Slc27a6	2.65465	0.00646	0.47782	0.62174	0.04196	2.11561	1.61561	1.59595
Rgs4	2.6508	3.7E-06	1.17931	0.6234	0.37023	4.5015	2.54867	2.97094
Rpl12	2.64502	3.8E-10	11.0428	6.18358	3.27478	40.359	27.4869	39.827
Cap1	2.64398	4.2E-07	14.2993	11.1655	4.05865	48.9886	34.9696	48.9365
Lrrc8c	2.64042	5.4E-08	3.68739	3.21966	0.91275	12.3564	9.48536	13.0516
F7	2.63815	9.9E-05	0.73699	1.53755	0.67308	4.37599	4.78724	5.11204
Ctgf	2.63716	0.00936	33.5532	18.1181	5.53976	81.6074	68.4569	62.7454
Slc31a1	2.63597	0.00017	4.37481	3.4655	1.72974	7.20194	6.31612	8.91653
Plaur	2.63335	3.3E-06	3.76938	3.07	1.19532	13.2799	9.61383	14.0108
Cfh	2.63315	0.00989	11.2588	5.1572	1.39481	22.9689	17.843	30.6369
Nsdhl	2.63113	0.01753	0.92842	0.5957	0.18274	2.36644	1.5714	3.85554
Nos2	2.63054	0.02336	0.08281	0.26264	0.16544	0.97836	0.86447	0.64449
Batf	2.62978	0.02528	0.76513	0.48535	0.32755	3.01327	1.75166	2.58571
Arsg	2.62576	0.00159	0.35076	0.15822	0.10678	0.89258	0.92507	0.99619
Aoah	2.62356	0.0284	0.376	0.10601	0.32194	1.00618	1.30842	1.3862
Armcx6	2.61707	0.03724	0.29393	0.18645	0.08389	0.71234	0.59215	1.0234
Tmco1	2.6169	0.00052	1.74469	0.97294	0.30642	4.79751	3.28667	5.13624
Nnmt	2.61587	0.0001	1.21706	0.58821	0.19849	2.9847	2.92958	3.22866
Tmem184c	2.61544	1.9E-06	1.50943	1.07806	0.45951	4.63367	3.95631	5.41362
Espl1	2.6138	1.2E-05	0.22042	0.27964	0.11009	1.0684	0.9386	0.84648
Arpc2	2.60751	2.7E-08	19.1391	14.5916	5.94958	64.5133	48.0867	65.4431
Rps23	2.60682	0.00013	80.5849	50.8881	26.6196	180.294	122.851	202.216
Rpl17	2.6055	1.4E-05	76.4879	44.5568	17.7598	198.367	128.215	195.135
Gpm6b	2.60317	7.7E-05	2.96847	1.79334	0.42826	7.97487	6.4882	7.37599
Haus3	2.60245	0.00409	1.65167	1.31713	0.53873	2.41603	3.19787	2.60964

Arpc4	2.60138	1.9E-08	9.68076	7.78841	3.23462	26.7997	21.1032	29.822
Il33	2.60122	0.02309	4.2409	4.33638	1.30068	17.4687	9.04234	18.1238
Nckap1l	2.60045	2E-07	3.21434	3.65214	1.41475	15.322	11.0789	11.7233
Eif4ebp1	2.59952	0.00551	2.78671	1.02017	0.66475	6.45128	5.05811	8.2121
Rnf130	2.59829	0.00185	5.39349	3.86755	1.0039	16.4101	11.9396	16.0897
Peg10	2.59813	0.00257	0.2466	0.22016	0.03128	0.71385	0.73266	0.80811
Selplg	2.59813	0.00102	3.15865	3.11679	1.80296	13.7685	7.90618	17.3954
Rpl7l1	2.59788	0.0004	5.29239	2.8458	1.52046	10.7877	8.03679	11.4856
Cd63	2.5954	1.7E-09	28.2606	24.2021	8.89999	109.66	81.1916	104.877
H3f3a	2.59426	0.00055	8.39347	3.81408	1.45813	24.8113	14.1885	30.0042
Cd9	2.59346	0.00021	14.2994	8.88168	4.74425	44.104	27.5641	55.1332
Nt5e	2.59329	1.4E-05	3.27351	1.9552	1.05947	9.71233	8.18338	10.2574
Vps29	2.59196	1.7E-05	2.27679	1.30045	0.64136	6.64651	4.87391	6.73436
Tmbim1	2.59065	0.00242	4.47344	3.02685	0.93901	5.86229	4.73082	5.95841
Cox6a1	2.59022	9.2E-06	19.2564	10.1242	5.49579	58.099	33.8379	64.9157
Pros1	2.58743	5.1E-05	7.61891	5.12114	1.45388	20.7524	17.1539	22.24
Il21r	2.58512	0.01267	0.32974	0.53453	0.28232	2.46399	1.73121	1.21563
Rcn3	2.58467	0.00012	7.07507	3.95932	1.51822	19.7896	17.1473	16.343
Diaph3	2.58119	0.00012	0.27895	0.60669	0.27296	1.7505	2.07279	1.63239
Plrg1	2.57823	2.6E-05	2.23803	1.12476	0.40731	6.11175	4.1108	6.29775
Pdgfrb	2.57512	2.8E-07	7.03623	5.48575	1.50373	20.8004	17.5773	21.3641
Mrpl52	2.57134	8.1E-06	3.37395	3.87278	2.20098	16.5728	10.9462	16.6835
1110008P14Rik	2.57079	0.03872	1.01712	0.91762	0.69669	2.34178	1.96219	3.11093
Slfn10-ps	2.56951	0.01019	0.37837	0.21334	0.11519	0.96283	1.47834	0.66129
Hist2h2ac	2.569	0.00715	7.72857	5.49676	1.60418	15.9634	10.5517	27.0535
Gng11	2.5679	7.8E-08	4.27368	3.95085	1.475	17.162	10.9945	16.2849
Acp1	2.56721	3.1E-06	2.07005	1.22317	0.48559	5.27455	4.52875	6.27253
Apobec1	2.56672	0.0004	1.25074	1.97275	0.8104	6.5949	7.13214	4.81874
Adam10	2.56577	5.4E-06	7.00881	5.22035	1.31586	19.6556	15.8805	21.5211
Slamf9	2.56478	0.00031	0.9739	1.05905	0.71473	4.97342	3.51639	4.10334
Prmt1	2.55988	9E-08	1.89342	1.37587	0.60888	5.7198	4.53252	7.25349
Gprc5a	2.55913	6.4E-05	33.6322	22.0295	6.6077	84.9625	63.5403	113.986
Rpl21	2.5585	0.00084	12.8141	7.77062	2.69879	32.1785	20.8809	43.5989
Esco2	2.55679	0.04469	0.09111	0.13485	0.10401	0.45542	0.66746	0.37321
Rps6ka3	2.55574	8.7E-05	3.94018	2.33927	0.71419	9.97474	7.94405	11.2666
Ntrk2	2.55422	6.7E-05	0.56896	0.35811	0.13595	1.87994	1.6818	0.95935
Pdzrn3	2.55417	1.8E-08	2.20288	1.84186	1.00593	6.3531	6.8149	5.21822
Phlda3	2.55341	1.1E-09	7.382	6.14446	3.47795	25.1319	20.9453	31.9667
Ppp2ca	2.5517	0.00014	6.03633	4.10497	1.41999	10.4917	9.03196	12.678
Cox8a	2.55169	0.00208	21.2842	10.7727	7.07898	62.5493	33.0269	82.9297
D8Ertd82e	2.55128	2.3E-05	0.31378	0.21713	0.13026	1.00817	1.0588	0.87246
Efemp2	2.54894	2.7E-10	3.20785	2.82984	0.90568	10.4076	8.92009	11.6426
Trim59	2.54475	0.00954	0.46323	0.36874	0.09332	1.53538	1.25762	1.18315
Tmem126a	2.54414	0.00643	2.24306	0.37943	0.40971	4.72944	3.61516	4.63087
Tgds	2.5402	0.00561	1.20977	0.40928	0.36829	2.1745	1.59528	2.51082
Myl12b	2.53625	0.00039	19.9651	14.4029	6.09418	57.992	28.2749	67.5691
Cxx1b	2.53489	0.00959	12.8258	7.77534	3.38544	33.0153	17.8146	27.2105
Cdc42ep2	2.53475	0.00536	3.06296	1.91705	0.2098	8.72224	3.76976	9.43517
Enpp1	2.53031	1.9E-05	0.27605	0.25607	0.11182	1.00893	0.98507	0.87545
Timp3	2.5263	0.0192	54.7756	28.2485	5.23662	78.2192	64.6292	67.6989
Dynlrb1	2.52494	0.00099	7.5134	4.13438	2.58353	24.4066	13.2633	25.8824
Selenof	2.52451	0.00037	18.7103	9.91348	3.66734	45.2398	36.8914	51.2137
Psmb2	2.524	0.00202	5.1586	3.5931	1.73208	15.1679	9.50347	22.3119
Dck	2.52263	0.00017	0.74186	0.75294	0.44039	3.05646	3.10871	2.50402
Brix1	2.52219	2.4E-07	1.60535	0.8769	0.41999	4.13377	3.4302	5.26077
Nusap1	2.51793	0.0169	0.22502	0.28548	0.28258	1.43156	1.10451	1.14299

Tlr2	2.51627	0.00058	1.51522	1.43533	0.72651	6.11419	4.50677	6.00816
Thyn1	2.51427	1.1E-05	1.23699	1.01247	0.36443	4.7144	2.80632	4.15171
Serpinb6a	2.51377	0.00043	3.73186	2.3137	0.94459	9.7809	7.97912	10.2788
Psmb1	2.51298	1.8E-05	9.16902	5.66712	3.92528	29.9668	19.5056	35.5365
Racgap1	2.51054	0.00033	0.21934	0.4256	0.23568	1.35242	1.56909	1.12048
Mmp2	2.50758	2E-06	7.64082	10.45	3.12412	32.5538	23.6608	37.7585
Clu	2.50753	4.2E-07	13.0179	8.08919	3.21489	41.2393	23.7836	38.2198
Osmr	2.50555	3E-05	3.04635	2.32144	0.98134	9.77687	7.25866	10.4401
Ubb	2.50509	0.00011	85.9391	52.3467	31.0636	258.891	158.794	289.264
Ssr2	2.50505	8.9E-07	5.6841	4.14441	3.02074	19.5055	15.9386	23.0428
Fdps	2.50253	0.00306	3.5113	2.07886	1.85394	7.36624	5.59487	9.81658
Hsd17b7	2.50186	1.1E-06	0.82318	0.45407	0.26558	2.21186	1.8354	2.72692
Ly6e	2.50138	3.9E-05	30.3749	26.9764	11.0033	114.659	70.0763	115.282
Tnfrsf13b	2.50024	0.00136	0.64413	0.73547	0.1103	2.94652	2.00551	1.55674
Rpl10	2.50023	0.00011	16.0163	7.26234	4.06318	48.0888	26.4655	56.5682
Psmd14	2.49925	2.4E-07	2.65592	1.61626	0.73951	7.22165	6.12186	8.38509
Cdca2	2.49778	0.00697	0.11187	0.16221	0.16421	0.71656	0.67909	0.54992
Cenpq	2.497	0.01628	0.24553	0.29667	0.08009	1.08384	0.87368	0.80461
Glmp	2.49554	6E-05	1.69836	1.39691	0.7293	6.15517	4.01799	5.6677
Ccnb2	2.49554	0.00775	0.65625	1.00917	0.20432	2.53023	3.32163	2.44379
Ostc	2.49182	3.7E-06	6.07776	4.70606	2.34538	19.6049	16.9321	21.2495
Ddt	2.4896	0.00266	2.3632	1.599	0.13489	7.01592	4.15503	7.93787
Ap3s1	2.4887	2E-05	3.89065	2.03706	0.70501	8.64334	7.73643	9.66351
Armcx4	2.48618	5.4E-05	2.67296	2.995	1.05628	11.4334	10.427	6.87835
Tmed5	2.48509	1.9E-05	1.52071	1.02493	0.31195	3.82225	3.21163	4.58434
Pgam1	2.48423	1.4E-06	11.0244	7.76315	3.87245	35.4828	28.3587	35.4732
Ccdc86	2.48288	6.7E-06	0.84095	0.68831	0.43356	2.46539	2.74251	3.77812
Sirpa	2.48049	1.4E-08	5.2448	6.44944	2.12322	22.5484	17.4402	20.1433
Sgpl1	2.4791	1.3E-07	3.10753	2.10012	0.93357	9.05365	7.81	9.25769
Rps8	2.47785	1.3E-05	44.3458	26.5413	10.8742	108.66	78.8926	122.171
Rhoh	2.4778	0.00574	0.16821	0.29747	0.08729	1.00989	0.82899	0.62639
Jam2	2.47741	0.00435	3.54649	1.63134	0.31963	8.02013	5.86859	7.25096
Bard1	2.47631	0.0042	0.2421	0.15016	0.0737	0.75299	0.68581	0.56859
1110038B12Rik	2.47505	0.00026	0.90783	0.63985	0.17273	2.47517	2.17995	2.93366
Krtcap2	2.47435	0.00015	3.36065	2.38557	1.23317	10.5444	5.80326	13.4712
Taf9b	2.47406	0.00695	1.24367	0.75833	0.09905	2.97903	2.2036	3.43578
Ddah1	2.46935	7.6E-07	1.62816	1.45465	0.52358	6.30788	3.69594	5.74885
Rpl38	2.46694	2E-05	62.0152	31.9269	15.3906	163.569	95.5552	181.55
Sf3b4	2.46663	0.00234	2.99952	1.21487	0.96458	8.42128	5.50905	9.10269
Smc2	2.46571	6.5E-06	0.78366	0.91664	0.39973	2.99025	3.17588	3.05982
Gpx1	2.46379	3E-09	25.9017	19.2409	8.5596	84.352	58.0498	88.4405
Fam206a	2.46356	3.5E-06	0.73739	0.38806	0.16836	1.94593	1.52458	2.06739
Cd93	2.46328	0.00404	20.697	18.0489	2.52988	58.5308	45.0866	55.8779
Slc25a5	2.46131	2.8E-05	22.3627	12.1557	5.44796	52.314	37.5219	67.8279
Taldo1	2.46016	2.6E-05	8.03569	5.55102	2.16132	22.5336	14.0237	30.0901
Eif4a1	2.45986	4.6E-09	45.0576	33.5102	11.6445	61.8008	50.0229	84.4113
Fbln5	2.45847	0.02246	2.2094	1.3253	0.23255	6.42736	3.22571	5.13494
Zdhhc13	2.45793	0.00014	1.28858	0.67315	0.21633	3.44439	2.41554	3.41543
Il1r1	2.4552	3.3E-09	2.97138	2.22176	0.73742	7.31429	7.22449	10.3135
Prr5	2.45313	0.00271	0.66705	0.36268	0.10879	1.55887	1.64055	1.71751
Rps7	2.45114	2.2E-05	48.6627	31.3509	12.1523	134.347	94.7648	138.99
Polr2d	2.44923	0.00348	1.81529	0.56565	0.98164	5.15193	3.35966	5.87047
A430005L14Rik	2.44896	0.00097	1.23588	0.64708	0.13437	3.4944	1.94014	3.61609
Tpbg	2.44759	0.00432	0.33405	0.30271	0.10896	0.93968	0.75164	0.91875
Rps17	2.4432	5.9E-07	26.8134	15.0987	8.67565	79.7954	51.4718	84.9902
Rps20	2.44267	8.1E-07	11.0366	7.06248	3.4246	31.739	23.0917	32.4075

Rbmxl1	2.44191	0.0069	3.50676	2.19024	1.66292	9.70817	7.50947	10.2542
Sema7a	2.44119	4.5E-07	13.5428	12.7894	3.95207	39.5836	39.7868	45.6053
Ncapg	2.43832	0.01663	0.11634	0.25146	0.22135	0.79885	0.88069	0.55068
Adamts1	2.43527	0.00089	15.2631	7.15402	2.94997	35.9156	34.176	28.6219
Hn1l	2.43379	0.00012	1.65049	2.05443	0.85323	6.47172	6.27384	7.3987
C5ar2	2.43223	0.00789	0.35909	0.23863	0.08784	1.18108	0.7892	0.98763
Slc19a2	2.43212	5.2E-05	0.78534	0.76487	0.38035	2.56657	2.37108	3.50943
Pgm1	2.42907	2.2E-05	2.01013	1.1036	0.56363	5.38442	3.95279	6.24056
Fkbp5	2.42886	0.00732	6.11501	4.20071	2.27781	10.6975	20.7691	21.0504
Lclat1	2.42866	3E-05	3.35041	2.12529	0.86726	9.85472	8.1626	7.91537
Zfas1	2.42816	2.1E-06	1.21617	0.92312	0.60519	3.26862	3.63237	4.34309
Uqcrb	2.42691	0.01258	10.8437	4.40935	1.66644	22.8688	14.5132	32.7163
Asf1b	2.42614	0.03162	0.15627	0.56171	0.0892	1.2545	1.2879	1.08819
Nedd8	2.4261	0.00022	11.0391	6.13921	3.10743	30.327	18.5009	37.1667
Sec13	2.42443	0.00013	7.1734	3.79775	1.62533	17.8826	14.0004	19.6493
Plbd1	2.42391	0.01621	2.80985	2.8753	0.55442	11.2445	5.10808	9.2648
Cd48	2.42366	0.00317	1.92047	1.81922	0.70157	7.8658	5.06493	6.16756
Havcr2	2.42273	0.0006	0.80562	1.67507	0.30657	4.10829	3.61494	4.40008
Grrp1	2.42269	0.00154	2.22076	2.54476	1.79919	10.6339	8.50193	10.3293
Dse	2.42239	2.2E-05	2.34763	1.81495	0.67002	7.53422	6.61071	5.73989
Galns	2.4205	0.00489	1.30975	1.10776	0.43282	3.44569	3.25729	4.26384
Itgb1	2.41997	4E-05	44.8519	29.038	9.07042	118.423	95.8692	111.776
Rpl23a	2.41985	0.0011	61.0355	29.3684	12.0645	162.426	96.8544	175.897
Mthfd2	2.41964	0.0015	0.89059	0.288	0.23922	2.36468	1.6311	1.86697
Slco2a1	2.41832	3.8E-07	14.5745	12.6104	3.61571	40.6403	33.2143	50.6015
Cndp2	2.41748	3.6E-08	7.45701	5.90599	2.60802	22.8515	21.2203	22.9537
Gm14305	2.41664	0.04789	0.47496	0.1545	0	1.59379	0.82973	1.28706
Cryab	2.41624	0.00671	16.4889	5.74557	2.39705	36.9681	19.9066	39.1094
Prkcb	2.41566	0.00362	0.73085	0.92722	0.37326	3.9387	2.42348	2.45806
Snx7	2.41458	1.2E-05	1.66232	2.54593	1.12837	7.0774	7.33973	9.09136
Rsl1	2.41404	0.01175	0.66508	0.56251	0	2.48492	1.4211	1.5438
Timm10b	2.409	0.00638	1.73394	1.31557	0.75686	3.05899	2.33505	3.71847
Dbn1	2.40846	0.00052	0.95101	1.33488	0.97018	4.32764	4.93623	5.61963
Rpl14	2.40595	0.00011	28.9337	17.1723	6.18934	74.3786	47.5405	82.3073
Sh3bgrl	2.40494	0.00064	14.8721	8.33888	2.37124	34.4658	27.8163	36.3027
Natd1	2.40457	4.9E-05	1.07532	0.90949	0.39283	3.88304	2.77296	3.43575
Cox6b1	2.40435	0.00037	23.3504	10.4748	4.71283	57.9974	35.4414	61.5119
Lamtor1	2.40207	1.1E-05	4.94801	3.84562	1.29767	14.4224	12.5891	16.7626
Psmb6	2.40162	6.9E-07	5.85497	4.53937	1.44821	17.0276	11.9028	20.506
Ubl5	2.40101	0.00215	3.21314	1.53702	0.84187	7.56375	3.74313	8.13452
Cnr1	2.40096	0.01156	0.14174	0.10789	0.01618	0.4208	0.22844	0.51094
Tagln2	2.40047	3.1E-07	33.0781	27.0863	9.71598	103.078	71.477	112.854
Lgalsl	2.3998	0.00059	2.84112	1.52063	0.63348	6.97968	5.9352	7.25559
Malt1	2.39938	9.4E-06	1.65643	2.23852	0.63718	6.2725	5.8564	6.9917
Cnn2	2.39903	7.7E-06	18.119	12.5299	4.66255	55.0474	34.1095	54.6008
Rpl7a	2.39881	2.1E-05	19.0778	11.1475	6.67431	56.9556	38.6978	64.0731
Plppr3	2.39628	0.00901	0.8595	0.65603	0.47864	1.30824	1.45898	1.30526
Arhgdia	2.39495	5.8E-08	30.338	23.7777	10.2978	93.818	71.5658	102.368
Acat2	2.39113	0.03522	1.83106	0.94135	0.16395	3.95906	2.55133	5.55922
Mfap5	2.39111	0.00357	2.0105	1.74349	0.29053	5.7514	5.92879	3.98226
Cd180	2.39093	0.01672	0.36462	0.68531	0.34688	2.55283	1.46915	1.4438
Ccnf	2.39069	0.00479	0.17684	0.6232	0.06729	1.5893	0.95004	1.35218
Tspan4	2.3906	2.9E-05	7.21307	3.75973	1.57987	21.4194	11.4594	20.9576
1700017B05Rik	2.39053	7.5E-09	2.63457	1.87411	0.91623	6.82895	8.11651	7.40428
Rpl27a	2.38869	1.7E-05	18.1753	9.74698	4.47307	51.2103	34.0597	56.4049
Ogfrl1	2.38713	8.6E-05	1.08132	0.66687	0.12859	2.3954	2.23622	2.74991

Nek6	2.38662	8.9E-06	1.50041	1.03829	0.51581	3.71894	3.14144	5.14737
Tlr1	2.38643	0.02171	0.27298	0.73881	0.09349	2.06739	1.43986	1.25237
Mmgt1	2.38578	0.00262	1.70422	1.53263	0.34478	4.65304	3.7297	6.11498
Lhx6	2.38544	0.00347	0.32315	0.22508	0.1085	0.97896	0.94695	0.8098
Nsa2	2.384	0.00013	6.22293	4.02652	1.04815	15.6789	10.8118	18.0247
Uprt	2.38396	0.00042	1.11001	0.57	0.18103	3.17051	2.14913	2.3384
Mcam	2.38282	1E-10	17.0133	13.029	4.32649	50.7475	38.3503	49.0074
AI837181	2.38239	0.00083	2.50895	2.87989	0.81835	8.21592	5.77669	12.4796
Rnf149	2.38214	3.4E-08	2.96091	3.13036	1.28594	9.4573	10.2327	12.4188
Cdkn2aipnl	2.38122	0.01293	3.73187	2.09108	0.79881	7.12239	5.60459	7.75806
Ctla2b	2.38122	0.00222	2.62785	1.01027	0.63636	4.96949	5.13372	6.98055
Scn3b	2.38045	2.9E-05	3.14438	2.80092	1.12654	12.9407	8.29524	8.01592
Mrpl16	2.37994	0.00315	2.52359	0.82622	0.65054	4.88293	5.12883	6.26909
Atp6v1f	2.37491	0.00015	5.24139	3.12495	2.05993	16.2948	9.69393	18.1617
Ndufb4	2.37454	0.00488	5.62077	1.4526	1.42593	10.5004	7.03448	13.8146
Snhg18	2.37183	0.01002	1.22931	1.09171	0.21051	3.6496	2.88187	3.97812
Siglece	2.37156	0.00588	0.59278	0.66848	0.33836	1.70599	1.77326	3.48042
Bud31	2.37123	0.00012	2.78157	2.20856	1.03688	8.90803	6.32121	11.115
Selenon	2.36987	2.2E-07	3.0286	2.09192	0.89318	9.11372	6.50829	9.15977
Fhl3	2.369	0.0092	0.70552	0.78032	0.5576	3.68275	2.18671	2.80108
Mrpl17	2.36838	0.00877	0.99685	0.80248	0.2468	1.97201	1.53975	1.83997
Msn	2.36634	2.9E-07	32.7412	29.9508	8.63573	101.085	77.9122	103.899
Ninj1	2.36581	2.2E-07	3.19972	2.16502	0.99622	8.91782	7.79945	9.67533
Kctd12	2.36511	0.0007	6.71046	6.37706	1.96251	16.9574	11.3345	13.3425
Slit2	2.36505	4.8E-08	1.67645	1.30306	0.54255	6.22411	3.73802	4.46261
Cd38	2.36335	0.00025	2.68147	2.00807	0.44642	6.194	6.36393	7.64308
Crtap	2.36261	2.3E-05	3.86587	2.70294	1.41225	12.8356	9.78022	12.7108
Gatm	2.36147	0.02247	0.39566	1.00394	0.41695	2.85827	2.74253	2.34386
Rpl13	2.36055	5E-06	30.0235	20.2135	9.24179	70.3317	48.5025	75.0308
Grwd1	2.35983	0.00021	0.97687	0.56333	0.35484	2.74412	2.11442	3.01935
Qpct	2.35946	0.01126	0.80704	0.44167	0.27098	2.07093	1.46069	2.83914
Ndufc1	2.35743	0.01399	2.07724	0.7751	0.48823	4.59006	2.64073	6.45681
Rraga	2.35731	3.7E-05	5.19348	2.9602	1.28889	14.4335	8.80874	15.2155
Commd3	2.35685	8.2E-05	2.39124	2.05563	0.76078	7.86158	5.31286	8.47847
Gimap9	2.35348	0.00137	0.94564	0.8939	0.38102	3.87581	2.28206	3.19003
Wdr75	2.35135	0.00094	1.52851	0.48874	0.3192	3.08322	2.58094	3.54292
B3gnt3	2.3504	6.5E-07	2.6609	2.04825	0.71676	7.46318	6.52706	8.10749
Rpsa	2.34913	1.3E-07	32.4148	22.0545	10.3614	90.0276	64.1903	93.3119
Ptpn6	2.34849	0.002	1.92324	2.42618	1.48853	10.4873	6.23282	8.70601
Ugcg	2.34798	8.6E-06	3.41727	3.00885	0.76023	9.83206	8.02395	10.5668
Mcm3	2.34761	0.00013	1.18974	1.23687	0.93377	5.21111	4.39431	4.93444
Pls3	2.34642	0.00013	12.3062	7.30957	3.05089	30.7255	24.7254	33.5653
Zfp277	2.34452	0.00132	0.70718	0.72404	0.38241	2.70611	2.07229	2.86631
Rplp0	2.34341	3.3E-06	70.6917	42.8866	20.6783	188.369	147.137	207.843
Clns1a	2.34335	0.00463	3.67762	1.7337	0.68826	8.65713	6.62508	8.79178
Tma7	2.34301	0.0007	6.18327	4.03596	1.61126	17.2657	13.5402	20.7583
Id2	2.34164	0.00066	6.18507	4.44045	3.36624	21.1773	13.5407	25.5715
Lyz2	2.34035	0.01603	342.408	305.468	45.382	882.859	572.674	1034.78
Fstl3	2.33988	7.3E-05	2.35348	1.16904	0.59706	6.85807	7.88181	7.77364
Akap2	2.33779	0.00043	26.317	20.8863	4.63684	50.5265	40.4529	55.1844
Psmb8	2.3363	0.00109	2.437	4.57412	1.67688	11.0639	8.1196	10.1741
Nfil3	2.33566	0.0036	1.44265	2.09716	0.87493	5.59954	6.01097	7.09139
Igfbp2	2.33463	0.00877	10.83	9.49439	1.75009	42.0068	16.52	26.2934
Hvcn1	2.33434	0.00913	1.51247	1.7779	0.96575	8.68228	5.18329	4.93539
Cenpl	2.33411	0.00308	0.17592	0.26782	0.06025	0.73545	0.70883	0.69179
Entpd1	2.33404	2.7E-06	6.26923	5.51007	1.62574	17.1375	14.1501	21.7782

Kynu	2.33388	0.0141	0.10398	0.20101	0.06783	0.71099	0.43528	0.43809
Emcn	2.33328	0.00037	0.92874	0.58557	0.13494	2.58846	1.91746	1.97828
Serinc2	2.33094	0.00045	1.26037	1.03269	0.17986	4.0091	2.22179	3.9044
Spc25	2.32974	0.00157	0.34381	0.46041	0.22895	1.40606	1.42726	1.50221
Mb21d1	2.32928	0.00314	0.39847	0.22468	0.07582	1.04617	1.00548	0.76171
Ncf2	2.32805	1.8E-05	1.36259	1.69151	0.97849	6.47141	4.42761	6.62585
Nkain4	2.32768	0.01452	0.73719	0.8729	0.08416	2.14392	1.51217	3.62368
2410015M20Rik	2.32759	0.02079	5.44396	2.45568	1.93351	13.9266	6.46976	20.7142
Fbln2	2.32758	0.0195	2.58097	1.11572	0.34375	4.37854	6.88041	3.06599
Fam198b	2.32128	0.00375	2.2051	1.13429	0.23554	4.75815	3.47651	5.43019
Tmsb10	2.32043	3.9E-05	30.2073	26.7596	12.6662	117.922	91.3503	105.91
Col6a2	2.31969	1.1E-08	8.00822	8.81446	4.68383	29.85	28.7793	32.4714
Tmem123	2.3182	0.00029	11.2813	7.37979	2.58247	31.0252	23.9114	26.6438
Pf4	2.31776	0.00475	2.02691	1.50003	0.14462	4.91228	5.19715	4.77411
Nipsnap3b	2.3137	0.00188	1.57012	1.28219	0.43266	4.92056	3.21279	5.32275
Laptm5	2.31114	1.3E-06	8.65633	11.6245	4.54192	39.9125	26.0199	38.0071
Tirap	2.31074	0.00012	1.09331	0.5799	0.52887	2.76194	2.44358	3.7498
Asnsd1	2.30962	0.01148	2.35593	1.3795	0.67977	4.47048	3.67947	4.00869
Pnp	2.30948	2E-05	5.24044	4.28203	1.82517	13.0771	11.3872	12.8798
2410006H16Rik	2.30933	0.00117	4.74481	4.18029	2.03126	17.9675	12.0212	15.063
Lpxn	2.30859	0.0176	0.76342	1.10689	0.62252	4.0968	3.23581	3.17185
Ranbp1	2.30705	2.8E-05	4.49561	4.12689	2.37834	15.9775	11.0451	19.3136
Msln	2.30626	0.0146	2.8516	3.14269	1.52905	8.90045	10.6985	11.5394
Mrpl41	2.30604	0.01033	2.6743	1.47892	0.46969	6.23197	4.59652	5.98294
Sh2d1b1	2.30591	0.03922	0.75397	0.54659	0.08197	2.47985	1.31512	1.64718
Avpi1	2.3053	0.00657	3.84268	1.43753	0.92798	8.64125	4.70992	11.4423
Nt5dc2	2.30335	0.00043	5.64774	3.0747	0.66698	11.2489	8.6079	16.2209
Tmem119	2.30293	0.00014	3.51147	2.66961	0.76571	9.46634	7.02373	10.9253
Mrpl32	2.30269	0.042	2.49751	1.30765	1.49347	7.49394	5.14048	9.74335
Ggh	2.30254	0.02394	1.53391	0.82559	0.26532	3.21056	2.92852	4.11273
Lrp1	2.30084	3.8E-08	22.5908	18.418	7.03304	62.371	54.82	71.0826
Sp3	2.29883	5.8E-05	5.23992	3.03808	1.31053	12.0148	8.73549	13.6825
Slirp	2.29746	0.00725	1.20646	0.87107	0.53748	3.08434	1.9617	3.9777
Dctpp1	2.29686	0.00548	2.16184	1.14278	0.15425	4.42064	3.95937	5.64538
Sftpc	2.29583	0.04433	771.598	856.303	152.646	2119.16	1216.01	3481.5
Psmb4	2.29253	1.4E-05	10.8232	6.64231	3.39033	27.6299	19.7259	36.225
Fads2	2.29163	1.4E-06	4.11867	3.79087	1.65949	12.9542	9.76189	16.5735
Eif5a	2.29148	1.2E-08	16.254	12.2524	5.92202	46.6326	39.1852	54.8389
Ctsh	2.2914	6.3E-09	14.1519	16.0814	6.6492	43.7615	44.1328	63.0574
Gpx3	2.29118	1.4E-05	109.222	74.9934	24.1556	234.21	237.647	311.079
Dnttip2	2.29065	0.00049	3.47795	1.93771	1.44953	6.4722	5.25762	8.29925
Lpcat3	2.29059	1E-07	2.37567	1.89111	0.9253	7.16302	5.85594	8.22019
Adamts2	2.28873	4.4E-06	2.99371	2.7058	1.0722	8.103	8.93038	7.88685
Aimp2	2.28854	0.02273	1.10444	0.46706	0.18913	2.6432	2.26551	2.98592
Ifi209	2.28772	0.00984	0.95277	1.21965	0.47035	5.05501	4.24457	2.17293
Eif3l	2.28671	0.0006	7.86478	3.71363	2.97446	21.3408	13.6089	24.271
Lamc1	2.28513	5E-06	12.4993	8.5358	2.92444	31.6639	25.1168	34.1669
Fam105a	2.28404	0.00044	1.38781	1.47406	0.58952	5.51443	4.13775	4.33638
Yif1a	2.28224	0.00626	1.95767	1.65576	1.0476	6.37539	4.70589	8.26977
Praf2	2.27867	0.02946	1.56891	1.15387	0.70085	5.0835	3.99781	4.80604
Msrb1	2.27831	1.4E-06	7.11533	5.71098	2.07217	19.0038	16.0103	24.3283
Mea1	2.27723	0.00094	5.21929	4.78225	3.10332	12.4515	9.71836	16.2129
Podxl	2.27606	0.00014	19.9718	16.4695	3.72398	63.4674	38.5044	46.7408
Cmah	2.27594	2E-05	1.08081	1.20076	0.33461	4.20654	3.34164	3.20417
Mboat2	2.27446	0.03463	0.42376	0.35841	0	1.28376	0.81194	1.01481
Cxcl12	2.27378	0.00057	8.49028	5.87703	1.28809	17.8013	16.2127	22.899

Nlrc4	2.27373	0.03837	0.21418	0.18115	0.05434	0.74978	0.52303	0.54591
Vamp3	2.27324	0.00804	9.04207	5.93627	2.09337	14.6922	11.627	14.1939
Rap1b	2.27304	0.00019	21.9319	15.3053	4.48202	52.715	44.4326	58.7466
Mrps34	2.27271	0.04635	3.49596	2.17083	0.95986	4.2631	2.98257	5.76444
Tlr4	2.27161	0.00029	1.2494	0.84816	0.58179	3.48613	3.09516	4.3098
Cyr61	2.27122	0.0083	21.3528	15.3432	3.30529	60.7395	41.9244	42.214
Gnpnat1	2.27059	0.00688	1.42444	1.08723	0.23797	3.68371	3.48692	4.26943
Lrrc59	2.27019	3.4E-07	7.16315	5.65625	3.6646	22.4745	20.5552	24.6183
Tmem106a	2.26964	0.00032	1.10606	1.40323	0.9304	5.74914	4.71251	3.81537
Marcksl1	2.26899	1.6E-05	2.33323	2.4066	1.68913	9.68878	8.3798	9.04474
Vmp1	2.2682	4.9E-10	9.22363	7.74118	3.83391	30.1202	23.9273	29.4317
Siae	2.26808	0.00702	0.59356	0.38249	0.06453	1.44707	1.0871	1.38937
Plxdc2	2.26773	0.00106	6.95517	4.10334	1.14073	16.0754	9.41044	19.7548
Actg1	2.26636	3.4E-06	147.006	107.798	53.4303	376.657	265.841	346.415
Arf4	2.26541	4.2E-05	10.0656	6.72829	3.15076	26.2436	22.0983	29.3942
Lgals3bp	2.2648	0.00032	9.59253	10.4196	7.21513	45.271	33.5859	38.4264
Lamb1	2.26471	1.1E-05	5.8669	4.06629	1.3143	14.1126	12.5163	15.4759
Psma4	2.26431	0.00029	4.933	2.96621	1.40788	10.8345	9.93818	13.7028
Anln	2.26339	0.00215	0.68187	0.61891	0.56958	2.68034	3.11905	1.90751
Dpy19l1	2.26261	7.2E-05	1.43839	1.38688	0.3941	4.14831	3.54061	4.00659
Fth1	2.26255	1.4E-05	410.281	276.269	107.522	927.677	789.902	1309.51
Cpne1	2.26136	0.00045	2.43189	3.44522	1.41126	8.61961	7.2748	10.6922
Blm	2.25951	0.0238	0.12132	0.25082	0.1385	0.80037	0.66163	0.66259
Zfp143	2.25866	0.00252	0.94379	0.75389	0.29929	3.00208	1.74774	2.77065
C1qbp	2.25859	8.3E-06	4.27014	2.38734	1.7351	10.7435	9.22577	13.4597
F10	2.25414	0.00701	0.40134	0.79204	0.64907	2.45189	2.42564	2.73998
Plk1	2.25335	0.03787	0.29075	0.56209	0.61643	2.31526	1.43016	1.73546
Tor2a	2.25271	0.00266	1.58718	1.11445	0.88887	3.41111	2.89592	3.55744
Tcn2	2.25183	0.00615	10.0228	6.98742	2.44162	23.9349	17.5117	32.7419
Lims1	2.25168	0.00013	7.2831	4.48569	1.42838	16.3949	14.6112	17.6248
Mettl9	2.25072	2.5E-05	4.8834	3.74786	2.73584	9.5064	7.98322	9.52031
Icos	2.24838	0.02478	0.23966	0.38288	0.0304	1.24232	0.54623	0.89446
Cltc	2.24773	3.6E-05	16.9585	12.4526	3.96278	43.9906	34.4841	45.8271
Mcm5	2.24628	0.00204	0.59474	0.96734	0.62673	3.36779	3.04989	2.69856
Kif4	2.24598	0.00272	0.24847	0.30822	0.1891	0.94341	1.09217	0.74639
St3gal1	2.24589	2.2E-06	7.89699	7.3455	2.32179	23.8788	18.0404	25.6085
Rras2	2.24584	9.9E-06	3.98349	2.73881	1.05621	11.0711	8.90546	9.76945
Lactb	2.24522	0.00013	1.65987	0.91063	0.40971	4.56635	3.02359	4.07957
Med6	2.24439	0.00062	1.45181	0.90759	0.64854	3.8627	3.14451	4.96446
Lamtor2	2.24415	0.00448	4.79935	2.34673	0.77048	11.0407	6.31776	12.7172
Apbb1ip	2.24363	5E-05	1.02253	1.12533	0.49224	3.82169	3.26715	3.47197
Ptk7	2.24317	5.2E-08	6.78324	6.28181	2.74463	20.016	13.5714	22.1233
Tceb3	2.24208	0.00019	4.90833	3.47863	1.59464	9.13319	8.0728	11.3483
Ptger4	2.24153	8.2E-05	1.00295	0.6275	0.28232	2.59718	1.5903	2.02605
Tuba1b	2.24139	1.6E-06	35.6582	23.6493	13.0687	103.862	82.8298	118.09
Rpl18a	2.24125	1.6E-05	44.2244	33.184	15.0501	108.388	61.7188	115.707
Il7r	2.24052	0.00207	1.73678	1.13181	0.84509	6.76223	4.06736	4.03535
Gpr65	2.23976	0.00974	0.84696	0.98026	0.15267	2.78185	2.54721	2.44682
Loxl4	2.2397	0.00453	0.56368	0.29338	0.17325	1.47118	1.35002	1.15449
Triap1	2.23956	0.00714	1.25925	1.49107	0.86254	5.28984	4.05908	5.2958
Vim	2.23916	1.1E-08	72.9923	61.0453	26.8887	221.995	181.435	223.372
Rpl10a	2.23875	4.9E-09	19.9862	12.5629	6.74724	57.2717	37.5729	55.0147
Tmem263	2.23853	0.0004	1.72936	1.3087	0.38965	4.02572	3.96746	4.77235
Hist2h4	2.23758	0.00664	2.35348	1.84835	1.02352	8.79326	3.81775	8.58467
Maoa	2.23704	0.02158	1.75354	1.18649	0.27525	4.43569	2.82619	4.72282
Rnf13	2.23438	0.00294	2.60927	1.28642	0.50893	5.37033	4.51468	6.34854

Mdga1	2.23384	0.04767	0.09722	0.10689	0.06659	0.34164	0.4487	0.27877
Shmt2	2.23344	0.00042	1.50578	1.13951	1.08569	4.87375	4.4125	5.81101
Psma5	2.23343	0.00076	6.01542	3.97662	1.97334	13.9502	10.4853	16.5972
Eif1a	2.23213	3E-06	5.80576	3.8371	1.73846	13.4361	12.7133	17.96
2010107E04Rik	2.2321	0.00122	15.0144	6.9844	3.14243	33.5071	21.6322	41.4122
Col1a1	2.23186	0.01267	33.9437	65.2926	42.4839	202.826	270.844	140.149
Gas7	2.23181	0.00011	2.35149	2.98327	1.25949	9.52419	11.0972	10.5032
Eef1e1	2.23173	0.00178	1.77011	1.04799	0.50519	3.8073	3.95515	5.36564
Mrps14	2.23152	0.00612	1.06532	0.52165	0.35205	3.02348	1.68413	2.89394
Reln	2.23137	0.00578	0.53907	0.25287	0.07757	0.8179	0.98231	1.25439
Map1lc3b	2.2306	0.00777	6.08755	4.04911	2.08071	8.81227	6.56938	10.9401
Sash3	2.23052	0.00169	1.19418	1.24088	0.66216	4.94069	3.34941	4.19288
Sulf1	2.22977	4.5E-05	1.63363	1.04653	0.37853	4.12574	3.63775	3.61755
Atp6v0d1	2.22877	2.1E-05	7.74306	4.91171	2.61696	20.2162	15.1516	24.2891
Pde4b	2.22841	0.02062	3.21757	2.31902	0.35547	9.56918	3.53576	7.96475
Adam17	2.22803	5.3E-05	2.17045	2.21367	0.77249	7.60401	5.59326	6.85112
Lrrc40	2.2276	0.00027	0.74663	0.38861	0.17484	1.32231	1.3464	2.05462
Wdr1	2.22687	5.7E-07	8.49572	6.82189	3.10203	26.4664	19.919	25.2342
Rab11a	2.22683	0.00015	6.65494	3.82128	1.28945	16.5539	11.4728	15.456
Aqp5	2.2266	0.00047	16.6019	10.5196	2.81477	37.6457	24.5657	47.8061
Ifit2	2.22609	0.00142	1.72116	1.41638	0.5045	4.49539	5.65705	3.50612
Eif3e	2.22597	0.00022	10.7874	7.26898	2.02994	26.3951	20.7557	26.1731
Ppib	2.22527	7.5E-05	6.60505	5.46956	2.39777	21.6838	15.1846	23.0034
Ripk3	2.22397	0.00186	0.93012	0.78668	0.79637	3.07131	3.40699	3.46712
Abcg1	2.22335	0.00026	4.65	4.57887	1.2053	16.2916	9.49557	13.8027
Phex	2.22177	0.043	0.2323	0.36839	0.09945	1.07319	0.64882	1.1062
Churc1	2.22097	0.00331	0.9145	0.67848	0.32968	1.46782	1.2929	1.40641
Magoh	2.22082	0.0009	5.10067	2.6085	1.62501	12.0023	8.95074	15.4517
Aspm	2.2207	0.01119	0.1655	0.24662	0.21592	0.95973	0.98147	0.67146
Capza2	2.22019	1.1E-05	5.34999	4.11834	1.22152	12.731	12.6101	13.9748
Carhsp1	2.22003	0.00026	11.5735	9.08366	1.82997	28.9228	22.1948	30.5987
Ndufb6	2.21985	0.0005	5.95981	3.04787	0.94936	13.9399	8.32609	17.7137
Kif15	2.21858	0.04679	0.05691	0.30484	0.08662	0.61489	0.70179	0.52839
Kdelr2	2.21592	1.8E-05	10.9865	7.22255	3.19257	27.8363	22.6093	30.9707
Aif1l	2.21584	0.01098	0.29449	0.1916	0.07758	0.75488	0.63063	0.83514
Ndc1	2.21543	0.00484	0.63068	0.54911	0.16941	1.82069	1.56276	1.82361
Kntc1	2.21531	0.01726	0.14874	0.16451	0.1698	0.67242	0.70409	0.54367
Clstn2	2.21453	0.00159	1.15556	0.5294	0.21987	2.62551	1.5873	2.97159
Abhd12	2.21424	1E-07	3.52667	3.17435	1.29277	11.9972	7.56123	11.9016
Rpl27	2.21377	0.00901	1.48758	1.71111	0.27172	4.90307	2.96424	5.50858
Dazap2	2.21211	0.00327	19.9582	11.042	4.5683	45.8539	32.0642	53.7654
Itgax	2.21138	0.0019	2.45316	3.98885	1.71506	14.8633	7.14689	11.3108
B4galt1	2.21133	7.5E-09	9.88016	10.6189	4.82693	31.2904	30.2959	38.74
G6pdx	2.21039	1.3E-06	3.79585	2.78407	1.01563	10.0259	8.08174	12.2933
Trmt10a	2.21031	0.02036	0.19468	0.0678	0.05229	0.39548	0.34396	0.39403
Gars	2.21013	2.2E-07	5.769	3.98767	1.63812	13.8039	13.8804	15.3305
Tyms	2.20965	0.00049	0.39001	0.32986	0.137	1.03609	1.25277	1.05687
Jkamp	2.20924	0.00316	1.69646	0.8609	0.2324	3.90586	2.38621	4.16952
Tgm2	2.20891	4.3E-06	26.6005	22.3641	8.64403	74.2188	60.0739	77.8006
Oaf	2.20825	0.00237	0.86856	0.61707	0.39662	2.44177	2.46884	2.39088
Psma1	2.20821	0.00039	4.77752	3.08566	1.58663	12.0521	10.6272	13.5567
Hgsnat	2.20753	4.5E-06	2.68071	2.0803	0.78874	6.81492	6.41801	8.0376
Tubg1	2.20718	0.00291	0.88677	0.95457	0.55219	3.54116	2.27375	4.16083
Ercc6l	2.20717	0.02017	0.22395	0.16027	0.059	0.59494	0.70674	0.40929
1810011H11Rik	2.2058	0.03384	0.43406	0.36712	0	1.23979	0.99938	0.96521
Dpysl3	2.20486	3.3E-09	4.85482	3.77319	1.64771	13.2233	12.3467	13.5803

Cxx1a	2.20484	0.00505	13.6815	14.2221	3.66477	34.1301	23.9096	25.7361
Bcl3	2.20458	0.00283	1.8083	1.90398	1.30603	8.4519	3.64982	8.01199
Rpl30	2.20453	1.2E-06	9.95035	6.03689	2.96932	27.7432	17.3487	27.9246
Psmc2	2.20391	0.00063	2.61431	1.76584	0.49742	6.29187	4.53537	7.06495
Rhoj	2.20316	4.8E-06	3.46531	2.31136	0.86839	7.65574	7.96685	9.00163
Cops2	2.20302	0.00375	5.25017	2.37262	0.94018	10.0731	8.8803	11.8284
Tmed3	2.20283	0.00028	5.02153	3.96016	1.93669	14.0611	11.9808	16.4557
Gcnt1	2.20099	0.00044	0.73778	0.5824	0.2246	1.91718	1.95779	2.04164
Alg5	2.20099	0.00017	1.07802	0.74599	0.33564	2.67199	2.44104	3.23161
Atp5f1	2.20056	0.00432	6.4971	2.46528	0.88904	11.742	8.67183	15.2939
Btla	2.20056	0.0007	1.34609	1.28081	0.28813	4.26475	3.96508	2.68805
Tinagl1	2.19978	0.01131	8.90741	4.98112	1.36381	21.2826	14.74	17.5482
Psap	2.19894	1.6E-06	87.4431	90.8778	37.8098	351.783	226.783	255.22
Slc41a2	2.19637	0.00018	1.19529	0.72211	0.3119	2.45196	3.17741	2.67196
Ola1	2.19541	5.4E-05	1.79546	1.01961	0.49779	4.30473	3.75812	4.53891
Pdlim1	2.19533	8.4E-07	2.88252	2.29112	0.73347	8.26941	5.91534	8.77746
Hexa	2.19531	6.6E-05	8.96902	7.43103	3.13441	31.4683	17.6335	25.1554
Slc30a4	2.19481	0.00227	2.01602	1.67694	0.30433	4.45182	3.88151	4.34068
Yipf5	2.1946	0.0013	3.96097	2.81941	0.80588	9.31439	7.92961	10.5705
Hspa13	2.19365	0.00372	2.20053	1.10098	0.30075	4.42231	3.34907	5.09101
Ccl22	2.18989	0.00313	1.09998	1.76101	0.35878	5.35547	3.91407	3.15408
F2rl1	2.18913	0.0094	1.22712	0.86957	0.15145	2.4917	2.08952	3.85828
Rpl15	2.18895	6E-05	17.414	10.3454	4.30658	49.1756	33.8335	53.8194
Knl1	2.18872	0.02462	0.17108	0.36175	0.26855	1.15318	1.33169	0.82345
Foxm1	2.18866	0.01822	0.3016	0.5236	0.38055	1.6927	1.97693	1.09239
Ier3	2.18852	7E-05	15.1018	9.49425	3.1346	47.56	23.7243	30.8314
Nsg1	2.1882	0.00013	2.41484	2.42688	0.45406	7.62537	5.45297	7.28506
Gnai2	2.18676	4.4E-07	22.7921	18.5617	7.09123	65.1221	48.7286	67.9579
Acsl4	2.18526	0.00014	7.60442	7.26066	2.21854	22.1564	16.7995	24.7953
Ap3m1	2.18408	0.0027	1.83587	1.00213	0.49051	4.78061	3.47205	4.87481
F630028O10Rik	2.18289	0.0117	1.2474	0.70335	0.37974	2.77119	2.1526	3.99693
Psmd8	2.18283	2.2E-05	5.76649	4.06854	1.36436	13.2149	11.6885	16.5226
Acot7	2.18277	0.00201	4.04225	3.27338	1.62004	11.4379	6.4267	17.4389
Zyx	2.18093	6.3E-05	9.86243	6.97989	3.07591	20.8731	15.3441	28.5016
Syngr2	2.1806	0.00472	10.4401	7.3511	2.70073	26.6417	16.0125	23.5104
Slc25a24	2.17964	3.3E-05	3.30104	2.16152	0.91173	7.90339	7.17694	8.74567
Kif11	2.17956	0.00374	0.45489	0.7855	0.32456	2.30822	2.33273	1.50621
Fam49b	2.17915	5.3E-05	2.40797	2.61566	0.72766	7.68093	6.77953	7.29142
C1qtnf7	2.17895	0.00213	2.04094	1.00231	0.30064	3.7496	3.3119	4.3869
Leprot	2.17779	0.00033	9.96148	6.30876	1.70306	20.5848	19.6721	23.7339
Tsta3	2.17713	0.00118	1.3934	1.47315	1.1135	5.78304	4.38943	5.36531
Cept1	2.17678	0.00572	2.87262	2.25607	0.70273	7.63676	5.79801	8.59716
Ceacam1	2.17402	0.01242	3.22498	3.06027	1.50858	6.40516	4.82891	7.42361
Tifab	2.17222	0.00378	0.6789	1.10423	0.41732	3.49631	2.35287	2.86652
Ramp3	2.17188	0.03313	0.86043	1.28753	0.22668	2.08527	2.56986	4.71748
Hyal2	2.17166	0.00017	3.85543	2.1348	0.75995	8.77237	6.48204	9.38486
Nmt1	2.17062	0.0003	5.27963	3.94189	1.47564	9.78402	7.05544	9.87378
Arsk	2.16902	0.04039	0.54664	0.23117	0.03671	1.00335	0.75382	1.17229
Elovl7	2.16859	0.01801	0.35291	0.19899	0.09593	1.01821	0.55402	0.89492
Mcm2	2.16656	2.1E-05	0.89121	0.96637	0.57392	3.43365	3.01336	2.97668
Dchs1	2.16526	0.00027	1.5289	1.24419	0.33965	3.31979	1.87081	3.97436
Gnl3	2.16522	1.1E-05	3.34694	2.58463	0.71988	7.55302	7.60459	9.81558
Sigmar1	2.16451	0.02211	1.92228	1.50266	0.49875	5.57634	3.88336	5.86987
Angptl2	2.16352	0.00572	3.44369	2.19982	0.76304	5.62554	3.97052	5.47587
Rnd3	2.16232	5.5E-06	2.26695	1.77791	0.49407	5.75624	5.14834	5.84179
Mrpl33	2.16178	0.00078	1.96988	1.72936	1.13865	2.74206	2.42956	3.03361

Uck2	2.1593	1.4E-08	1.83879	1.52694	0.82058	5.43878	4.55559	6.12162
Rpl6	2.15908	4.1E-05	30.9339	17.3681	9.98572	81.8611	58.6901	84.2095
Kpna2	2.15815	0.00056	3.56067	2.60822	2.03244	10.5363	7.80228	11.408
Rps15	2.15524	0.0001	85.8283	64.0086	26.8127	233.394	134.358	282.45
Copb1	2.15524	0.00045	4.48343	2.97548	1.08344	10.7071	9.03845	11.0863
Ptprc	2.15501	0.00105	2.76804	3.47587	0.88023	10.726	6.63146	9.01409
Wnt2	2.155	0.01095	0.97211	0.58239	0.04624	1.84057	1.15726	3.05289
Txndc17	2.15461	0.00169	4.20815	3.79257	1.10257	11.7033	8.69267	13.7902
Arl6ip5	2.15382	0.00012	9.05775	7.12516	2.097	26.7105	18.1436	21.9505
Actr2	2.15228	9.1E-06	14.2167	10.6733	4.26784	37.5596	31.9394	37.3569
Stt3a	2.15196	0.00033	4.06262	2.89219	0.93224	9.90774	7.69488	10.7293
Sept10	2.14883	0.00202	1.6548	1.09534	0.65709	4.42457	3.68959	4.80391
Eif2s2	2.14865	6.4E-06	2.83951	1.9899	0.83355	6.66864	5.81467	8.31925
Ms4a6b	2.14853	0.00547	1.18118	1.4813	0.48823	4.02247	4.26694	3.55375
Cse1l	2.14786	0.00321	2.89273	1.6848	0.39549	5.8142	5.15206	6.2724
Fndc3b	2.14694	6.5E-07	4.58875	3.82727	1.44355	12.2196	11.4192	12.4509
Pole	2.14564	0.03126	0.17144	0.2175	0.26095	0.83096	1.00475	0.80369
Fli1	2.14513	0.0003	4.61142	3.32409	1.0469	11.6839	7.88909	13.1584
Itgav	2.14482	2.7E-08	3.77456	2.92642	1.11609	10.2243	7.88461	10.1824
Twf1	2.14371	0.00032	7.26753	4.93258	2.69726	14.895	13.2994	16.6858
Hist4h4	2.14332	0.04136	1.1804	0.56664	0.3642	2.41617	1.51916	3.13639
Adh1	2.14201	0.0056	7.68986	5.12013	1.72774	18.1905	9.8587	22.0164
Mybl1	2.14165	0.00583	0.28744	0.18591	0.07721	0.82979	0.49547	0.76186
Esyt1	2.14001	4.2E-08	10.6771	9.7848	3.81791	32.4805	23.9559	27.8046
Pdap1	2.1373	0.00025	3.62383	2.63057	1.10754	7.48583	6.4593	10.7299
Picalm	2.13682	2.6E-05	7.3464	5.09664	1.58239	16.6948	14.6921	17.8047
Rac1	2.13674	1.9E-05	15.0943	11.8902	4.17457	37.64	29.5871	38.5129
Adam15	2.13645	0.00038	6.09413	5.69488	2.76587	15.6519	13.84	13.0783
Ppp1ca	2.13561	1.5E-06	20.2016	14.6773	6.20033	57.1056	38.1877	61.5887
Clic1	2.13336	6.7E-05	10.3573	9.40419	8.63616	44.5118	28.6399	44.7143
Myo5a	2.13299	1.4E-06	1.43149	1.33598	0.66917	4.77023	4.39369	3.79124
Ednra	2.13286	0.00885	4.47014	2.77914	0.83783	9.85324	7.52714	11.2052
Mrpl46	2.13262	0.00372	1.03279	0.98271	0.36845	2.97232	2.26985	3.9662
Mtpn	2.13155	7.7E-05	10.3331	6.90584	3.05442	26.0349	21.2568	26.4547
Rfc3	2.13118	0.02625	0.22765	0.29951	0.14438	0.82757	0.68563	1.09829
Lrp2	2.13112	0.00143	1.63523	2.27578	0.54942	5.71921	4.0386	6.7118
Emc3	2.12986	0.0018	4.91183	3.58352	1.15572	11.904	9.28436	13.7002
0610009B22Rik	2.12961	0.04367	1.09825	0.69666	0.31344	3.16069	1.74323	3.07413
Csf1r	2.12603	2.1E-05	6.82461	5.96191	2.24125	21.207	15.3825	18.8872
Manea	2.12403	0.0027	1.6327	0.79363	0.34279	3.52483	2.70843	3.68994
Plk2	2.12378	3.7E-06	5.63933	5.07292	1.6746	19.7699	11.1283	14.7949
Rabl3	2.12377	0.00257	0.32984	0.19407	0.16372	0.79938	0.8615	0.89292
Brca1	2.12335	0.0192	0.14986	0.23539	0.14664	0.74582	0.78417	0.50862
Dbi	2.12242	0.00197	7.96474	3.36822	1.39322	14.3993	11.1993	19.4176
Bhlhe40	2.12213	0.00011	7.25788	4.48589	2.54942	21.0738	15.2764	17.2207
Cd86	2.12195	0.00645	0.69857	0.73854	0.28482	2.93251	1.80946	1.73736
Api5	2.12147	0.00145	5.90831	3.72236	1.28475	12.7117	10.4529	14.2743
Pxdn	2.11942	0.00206	4.71228	3.80698	1.07372	12.6589	9.59018	11.4166
Coro1a	2.11936	0.01259	4.85733	5.99582	3.82166	23.9813	14.354	21.3221
Abcb1b	2.11717	0.00041	0.51513	0.5709	0.30418	1.75426	1.57458	1.80451
Ier5l	2.11443	0.00033	1.29364	0.97896	0.46637	4.2696	2.1947	3.84882
Adgrl4	2.11418	0.0077	8.8449	6.23406	1.34177	20.2531	15.7177	19.6334
Pim1	2.11393	1.8E-06	4.74346	5.79827	3.04356	15.251	12.4036	16.0834
AI413582	2.11377	0.03152	0.78401	0.71836	0.67128	3.28556	2.24959	2.67628
Psmc1	2.11304	1.8E-05	11.0712	9.21118	3.29712	26.8314	24.6407	35.0976
Rpl37a	2.11292	9.1E-05	42.3651	24.4904	12.202	107.736	63.3066	108.051

Chchd2	2.11266	0.00175	12.8412	7.42216	3.67639	21.3408	14.3617	24.7341
Cd81	2.11177	0.00239	17.6076	12.5041	2.96987	43.1724	30.7025	47.642
Ufm1	2.1093	0.00019	1.96227	1.27067	0.43753	4.3005	3.95325	4.84793
Ctsa	2.10519	6.4E-07	6.10209	5.50511	3.71529	20.6336	14.9437	21.5203
Smpdl3a	2.10492	0.00877	6.05969	3.85323	0.9594	14.0159	9.59145	14.0599
Procr	2.10485	0.04284	0.6296	0.38036	0.56474	2.07082	1.58141	2.2106
Sh2b2	2.10464	0.01343	0.88063	0.9543	0.12567	3.06795	1.67334	2.41238
Snrpb2	2.10414	0.004	1.85425	1.05454	0.36497	4.22264	2.90418	4.60969
Map2	2.104	0.00068	0.26098	0.17377	0.09509	0.67623	0.50849	0.787
Ddb1	2.10339	9.5E-06	17.4761	13.1449	5.04418	44.8446	34.3021	48.6973
Tmem86a	2.10177	0.00065	1.69413	1.10535	0.71835	4.86826	3.8297	4.28274
Mrpl20	2.10144	0.00084	1.97605	1.76416	0.43864	4.85554	3.94081	6.79037
Mcemp1	2.10023	0.00188	1.10045	1.18928	0.34897	3.11148	2.77686	4.15716
Abce1	2.09982	0.00102	3.8032	2.44468	0.71205	8.23093	7.12152	8.57467
Rpl19	2.09944	2.6E-07	62.2025	40.8935	23.6702	167.103	105.673	182.899
Ywhaz	2.09934	0.00013	35.6762	23.9347	10.1429	83.6394	62.0943	85.7193
Naa20	2.09724	0.00104	1.52846	1.16764	0.36586	3.5997	3.50369	3.99895
Kif20b	2.09622	0.01333	0.29554	0.49992	0.24367	1.27332	1.61179	1.03574
Elovl1	2.09562	0.00153	9.01314	8.84173	3.13656	26.2083	14.8996	36.7831
Hmga1	2.0953	0.00116	3.85546	3.06678	1.57193	11.9579	9.58306	12.9729
Lipg	2.09451	0.00402	2.53247	2.73349	0.46808	6.45899	4.8767	9.03006
Casp3	2.09335	0.00077	1.08832	1.08157	0.5591	3.19807	2.85034	4.25747
Kif22	2.09293	0.01674	0.22355	0.42541	0.27647	1.02711	1.0645	0.91571
Ube2v1	2.09256	0.00155	9.53321	5.70658	2.83575	20.7041	14.1155	23.898
Ptprj	2.09167	7.9E-10	5.5883	4.32876	2.1099	12.9603	11.3605	14.1021
Pes1	2.09165	1.4E-05	3.77553	2.24807	1.55166	8.7292	6.63823	11.2095
Alpl	2.09001	0.01917	0.72472	0.3831	0.25855	1.49567	1.19458	2.37493
Plxnc1	2.09	6.6E-05	1.66683	1.91256	0.54558	6.44078	4.06467	4.47866
Txnl4a	2.08941	0.01269	1.25406	0.77782	0.42949	3.11524	2.20492	3.11644
C3	2.08892	0.00128	42.4809	40.7513	18.7944	125.152	99.6739	156.38
Mrpl54	2.08886	0.00485	3.6535	1.96641	1.01112	7.5128	5.7586	11.1563
Gng12	2.08876	7.5E-05	4.86928	3.38293	1.52205	11.356	10.3619	13.2815
Ddx21	2.08813	7.9E-08	5.70138	4.08903	1.979	13.0939	12.4175	17.3738
Atp5g1	2.08799	0.02452	2.41732	0.64481	0.29719	3.66153	2.84709	4.28073
Actr3	2.08644	3.7E-06	11.893	11.0264	3.78268	33.4118	29.0136	31.9449
Scfd1	2.08547	1.2E-05	3.71291	2.73392	1.496	8.99835	8.25616	12.1315
Uba1	2.08381	7.2E-06	11.4859	8.60846	3.33219	30.4317	22.0499	30.9316
Acadsb	2.08376	0.00075	4.49118	2.46422	0.75527	7.2846	7.05577	9.04743
Grb10	2.08365	0.00023	2.88413	2.47335	0.86042	7.36728	7.20741	7.39315
Krt18	2.08173	5.4E-05	17.0279	10.7049	9.01203	49.292	32.4052	59.97
Ifngr2	2.0813	6.9E-06	4.85222	3.96402	1.35336	13.313	10.3208	12.7613
Rps21	2.08114	0.00211	47.473	25.083	10.4271	104.736	61.9568	122.498
Krt7	2.07981	1E-07	8.03978	6.85466	3.29589	22.2693	18.5422	26.2087
Erap1	2.07873	0.00035	3.66352	2.92533	0.90919	9.30603	7.01806	10.2717
Ndufs8	2.07828	0.02651	5.12173	1.92528	1.08278	10.6887	6.87894	11.1117
Snap29	2.07694	0.01608	2.55713	2.16278	0.57777	6.92353	4.3321	6.67765
Psmb5	2.07665	0.00058	12.5838	7.62761	5.98573	27.4475	19.2827	28.4367
Fam217b	2.07636	0.0256	0.96166	0.47446	0.1601	1.73582	1.76126	2.03527
Tmtc3	2.07552	0.00031	1.74726	0.94199	0.44326	3.68355	3.08405	4.32784
Clptm1	2.07456	3.4E-05	3.45641	2.4009	1.02424	8.68862	6.4435	9.50725
Dcaf13	2.07381	0.0001	2.47336	2.34096	0.9412	7.56418	5.09076	8.7807
Asna1	2.07271	0.00281	4.02115	1.84627	1.63949	10.3719	6.90175	10.2596
Myd88	2.07264	0.00195	3.51026	3.35877	1.57864	9.25911	8.41611	11.6194
Steap3	2.07248	0.00038	0.73518	0.60828	0.18245	2.01412	1.33475	2.23898
Ndufb8	2.07218	0.00078	3.77428	1.64522	0.72917	6.57886	5.29606	8.61032
Mrpl13	2.07144	0.00041	0.58697	0.37964	0.18395	1.32492	1.20572	1.53693

Psen1	2.07099	0.00138	3.60775	2.87771	1.47333	11.1426	7.04803	11.2219
Cmklr1	2.07074	1.1E-05	10.372	9.45246	2.90663	27.1178	21.951	31.2665
Csmd1	2.07063	0.00066	0.17279	0.17862	0.04931	0.55543	0.43953	0.46793
C2	2.0692	0.02359	0.99075	1.17713	0.32316	2.84417	2.35026	2.80222
Calm2	2.06741	0.00418	35.9871	19.7474	6.32594	71.3722	54.8717	82.0211
Rap1gds1	2.06687	1.4E-05	2.46499	1.99287	0.62075	7.06482	4.84212	5.98909
B3galnt1	2.06674	0.01562	0.76565	0.87613	0.10283	2.04659	2.17765	2.14008
Tmem128	2.06561	0.00076	1.96324	1.18606	0.58699	4.81462	3.49292	5.24645
Klhl6	2.06525	0.0002	1.50711	1.74898	0.76023	5.139	4.98127	4.65168
Cln5	2.06344	0.00043	3.62148	2.16021	0.95742	7.99148	7.42861	8.33862
Nhp2	2.06286	0.00187	3.10835	2.15712	2.09271	8.40239	6.83146	12.4062
Zdhhc5	2.06214	8E-06	5.61019	3.82662	1.67347	13.3224	11.097	14.9747
Sardh	2.06061	0.00025	0.80711	0.95285	0.47989	2.54694	2.71004	2.86532
Sema3a	2.06015	0.00864	0.955	0.69074	0.23308	2.24264	1.90106	2.40653
Mtap	2.06009	0.00025	1.5289	0.74234	0.35554	3.53379	2.38532	3.45614
Cd3eap	2.05898	0.01367	0.91631	0.558	0.46027	1.77656	1.53052	2.43224
Mpv17l2	2.05797	0.00959	2.1418	0.93497	0.39437	4.06049	2.88507	5.49047
Rpl18	2.05765	7.6E-06	10.618	8.3691	4.28124	29.99	17.9241	30.8903
Atp6v0e	2.05671	0.00134	3.72521	2.41634	0.92728	9.45241	5.78643	9.70642
Rbm7	2.05579	0.00119	3.00343	1.77817	1.02862	7.2789	5.45183	7.13568
Amica1	2.05566	0.01878	0.99919	1.54106	0.50324	3.1694	2.13141	3.97258
Sec61a1	2.05505	8.9E-08	5.88837	5.6688	2.41627	17.1478	15.7344	20.3523
Tpgs2	2.0549	0.01571	0.45402	0.192	0.04573	0.75243	0.66522	0.83143
Scnn1g	2.05436	0.00479	6.38233	4.18415	2.02198	17.0035	11.2753	16.5369
Rcan1	2.05265	0.00032	4.15502	4.06913	1.53952	14.7956	9.05172	11.4662
Smdt1	2.05153	0.00166	7.46305	4.66236	2.03315	17.3162	11.6803	21.5385
Hells	2.0512	0.00485	0.25354	0.36455	0.18814	1.02154	1.0773	0.8724
Edf1	2.05108	0.00475	8.59974	5.86574	2.3752	21.43	12.4478	24.0339
Pycard	2.05014	0.01915	1.68253	0.99614	0.4116	3.52427	4.0148	3.6036
Snrpa	2.04914	0.00239	2.81199	2.42238	1.30785	7.50906	5.18826	8.66036
Tmsb4x	2.049	1.2E-08	163.022	152.588	65.4834	490.551	355.212	534.282
Ap1s3	2.04876	0.00624	0.85688	0.51	0.32607	1.90363	1.74375	2.73003
Sf3b6	2.04847	0.00187	3.44052	1.93995	1.21572	8.18931	6.24124	9.32845
C1qtnf1	2.04836	0.02716	2.42128	2.14657	1.1656	5.99176	5.89846	6.07044
Pdcd6	2.0471	0.00033	4.13536	2.73727	1.57365	9.76799	8.12268	12.717
Vps35	2.04678	0.00087	7.1959	4.18077	1.64797	15.7962	12.1617	16.6107
Ube2j1	2.04643	0.00024	5.09731	3.19511	1.21109	10.8798	9.62945	12.2736
St8sia4	2.04556	0.04948	0.81088	0.5761	0.11108	1.97174	1.37819	1.58108
Ubash3b	2.04339	6.6E-05	0.66078	0.75923	0.28466	2.26617	1.99114	1.88962
Irf5	2.04196	0.00241	2.13802	3.11134	0.82556	8.53434	6.127	7.03212
Mfge8	2.03968	4.3E-05	9.04814	6.84634	2.04367	24.0859	15.4097	21.8078
Cenpf	2.039	0.035	0.21736	0.50385	0.26651	1.35106	1.62772	0.76504
Lrpap1	2.03846	6.8E-05	5.01513	3.56604	1.39333	12.598	9.05508	13.2714
Sept2	2.03816	8.9E-05	13.2405	9.2296	2.86529	29.7416	24.2364	30.5455
Pcdh18	2.03738	0.01368	1.8213	1.29569	0.34977	5.0534	2.1947	4.75525
Nedd9	2.03699	5.2E-05	7.05579	6.13161	2.27928	20.7524	15.3367	18.1514
Plod2	2.03518	0.00725	3.803	2.88377	0.62378	8.36918	6.50079	7.66374
Mfsd1	2.0344	6.3E-05	5.28449	4.26637	1.9538	14.6081	12.362	13.8982
Fam20c	2.03388	0.00012	1.27609	0.8049	0.61728	4.00994	2.69364	2.97686
Rap2c	2.03228	0.0014	2.11275	1.35109	0.79417	4.8237	4.20922	6.22695
Tpp1	2.03202	0.00068	4.6164	3.99423	1.91824	14.6173	9.58866	13.8819
Ifngr1	2.0317	0.00187	10.4587	10.284	2.051	28.7487	19.155	28.9906
Tmem185a	2.03145	0.00648	1.78096	1.56778	0.20746	4.25014	3.22185	4.85361
Creg1	2.03132	0.00201	7.61791	4.2845	1.28022	16.3302	10.6234	17.3291
P3h1	2.03002	0.00012	1.8049	1.76759	0.81335	5.48218	5.20205	5.05864
Plk4	2.02998	0.0154	0.34273	0.35576	0.33791	1.19873	1.41519	1.07208

2700060E02Rik	2.02995	0.00045	7.07715	4.19513	2.07162	14.8795	12.3191	17.5427
Ltbp1	2.02865	3.4E-05	4.33156	3.11071	1.2238	9.87457	9.4049	10.3747
Ywhaq	2.0273	0.00265	15.4865	8.60596	3.91742	32.5965	23.412	36.62
Spon1	2.02687	0.005	7.2141	5.0896	1.386	14.6372	13.915	16.354
P2ry2	2.02658	0.00019	1.78061	1.16662	0.31493	4.1786	3.01312	4.17086
Tmx2	2.02652	0.00055	4.66488	3.38184	1.3873	8.1466	6.34671	9.15289
Mef2a	2.0263	0.00056	6.49423	3.95917	1.08471	11.9679	10.8499	12.7833
Rgs19	2.02626	0.00469	1.72026	1.21247	0.84165	5.03766	4.23084	4.26156
Rps4x	2.02582	4.3E-05	57.457	40.5915	16.5581	133.82	90.8725	147.96
Abhd4	2.02571	0.00039	3.6769	2.45019	1.43099	9.38364	6.42809	11.456
Rarres2	2.02534	0.00562	4.29864	3.08309	1.3348	9.66781	6.32351	12.5654
Pus1	2.0247	0.02622	1.80161	1.34659	0.81313	3.70625	2.94664	3.19224
Gnpda1	2.02417	0.01046	2.17652	1.22724	0.30514	5.25173	3.38483	3.37856
Csnk2b	2.02366	0.0004	4.46304	2.87779	1.16025	10.2808	6.73341	12.924
Jak1	2.02328	4.2E-07	8.89742	7.69631	2.81347	24.4347	18.2583	24.9016
Cdkn1c	2.02319	0.00213	5.59941	3.03097	1.17619	7.40941	6.13673	9.3949
Top2a	2.02318	0.0242	0.64857	1.43995	1.05278	4.51904	4.84793	3.23791
Mrto4	2.02314	0.01111	0.90953	0.94679	0.51916	2.64936	2.71651	3.00921
Rpl13a	2.02286	4.4E-06	47.3106	33.2906	11.5419	105.758	81.5051	119.995
Olfm1	2.02254	0.00025	2.62213	2.00033	0.52825	6.58845	4.74589	6.4446
Adam9	2.02158	6E-07	11.3201	10.2502	3.52217	29.0215	26.0173	31.8409
Rpl36a	2.02142	5.9E-05	15.0666	9.94237	4.59929	34.3074	27.6548	40.5568
Dpagt1	2.02128	0.00178	1.94676	1.48865	0.548	4.3464	4.08318	4.50066
Eefsec	2.02065	0.00359	0.68479	0.74466	0.29781	2.09426	1.81557	2.00363
B3gnt5	2.01992	0.01621	0.33393	0.32702	0.10032	1.13938	0.82402	0.74872
Rrn3	2.01924	5.9E-05	2.81859	1.86847	0.78269	6.72325	4.65063	7.88439
Manbal	2.01766	0.0002	3.87793	2.93096	1.69547	10.4981	8.64372	11.2886
Got2	2.01751	0.0188	8.00717	3.8849	1.1692	14.1593	10.4815	16.1201
Dda1	2.01745	3.4E-05	3.30561	1.95437	0.80603	7.0779	6.08944	7.96668
Atp5o	2.01639	0.03371	6.83314	2.66739	1.06164	13.0572	7.37557	16.1318
Lpcat2	2.01621	0.00815	0.67398	1.02607	0.36932	2.74412	1.87626	2.58412
Ntpcr	2.01597	0.00545	0.51256	0.83812	0.4291	2.09103	2.10278	2.32353
Stk17b	2.01522	6.6E-06	4.54885	3.84734	1.10826	11.6293	8.51402	12.8616
Necap2	2.01391	1.9E-05	3.86599	3.21986	1.34761	10.5673	7.22102	12.2579
Gucy1b3	2.01388	0.00077	5.14021	3.63931	1.30107	12.5561	8.12774	14.0245
Hspg2	2.01316	0.0002	12.5129	9.24948	3.12661	32.2013	23.469	27.7202
Cercam	2.01312	0.01724	0.78297	0.54805	0.30822	2.04478	2.03727	1.61469
Slc35a4	2.01274	0.00887	4.46229	2.12295	0.92332	8.22916	6.41551	9.48214
Gorasp2	2.01271	1.5E-05	5.46483	3.8952	1.55967	12.2562	10.3639	14.6047
Brox	2.01211	0.00206	2.88051	2.15873	0.68682	6.52811	5.00848	7.00494
H2afx	2.01122	0.00062	4.15771	3.40488	0.82875	11.1559	7.88069	10.1106
Ddx3x	2.00961	3.3E-05	22.5531	14.7125	5.39437	44.5907	39.3339	59.2023
Ndufa7	2.0087	0.00748	4.40311	2.90659	1.2873	9.69505	6.29402	14.649
Ptbp3	2.00868	0.00155	7.61961	5.78355	1.58916	17.4232	13.8299	17.4968
Nrep	2.00856	0.00035	5.59729	5.34891	1.41076	13.0587	15.3371	13.2506
Tgfbr1	2.00795	0.00035	4.10977	4.31541	0.94154	11.1459	8.89924	11.4408
Arf2	2.00786	1.8E-05	3.29035	3.0406	0.76517	9.02641	7.09754	8.43635
Bcap29	2.00767	0.00667	1.86496	1.15416	0.51928	3.80324	3.79886	4.73271
Ppp1r2	2.00767	0.00033	13.4744	7.53379	2.5833	25.6383	25.62	29.5439
Nxpe4	2.00745	0.00993	0.49447	0.58811	0.15876	1.71328	1.35842	1.16465
Rps10	2.00743	8E-07	22.7283	13.6796	6.02912	53.0257	36.3757	62.3896
Mrps7	2.00742	0.00031	2.51267	1.56591	0.80519	5.31498	5.23164	6.60893
2610001J05Rik	2.0068	0.00073	1.47241	0.97994	0.38578	3.4368	2.79395	3.65839
Chst14	2.00662	0.00129	1.17996	1.40458	0.69847	4.44837	3.39369	4.00997
Pkm	2.00639	1.4E-07	18.0264	15.1538	7.10989	47.7155	43.4181	50.7388
Col18a1	2.00552	5.8E-05	3.99382	2.65171	0.98019	9.89197	8.25337	8.14261

Gpihbp1	2.00486	0.00233	7.36709	5.20255	1.63307	12.4374	10.1654	26.0466
Tmem106b	2.00471	0.01478	4.04568	2.39635	0.90265	8.21594	7.07653	9.09046
Cmpk1	2.0036	0.00013	3.92591	2.82353	1.09759	8.70551	8.82395	9.29892
Tbx4	2.00262	1.7E-06	4.78039	3.52074	1.71691	12.0736	10.4866	12.4972
Col15a1	2.00094	0.00095	2.34494	2.21449	0.78832	5.95324	6.724	5.62189
Lamp2	2.00052	0.0003	7.97483	5.45097	2.15049	18.632	14.0454	20.0233
Atp2a3	-2.0019	4.1E-07	22.2247	17.7587	7.3845	13.8952	9.78168	17.5418
Abtb1	-2.00292	0.00094	2.4234	2.58989	1.22243	1.41138	1.80289	2.38554
Mum1	-2.00303	2.3E-05	2.53887	2.12496	1.16741	1.64508	1.76309	1.74777
Arhgap26	-2.00746	0.00594	1.77078	1.67154	1.04687	1.72109	1.80304	1.92135
Chd5	-2.00785	0.04337	0.34312	0.17735	0.10881	0.17902	0.15362	0.21083
Sgip1	-2.0096	0.00012	1.46883	1.17371	0.54522	1.04827	0.8648	1.28455
Ppp1r14c	-2.01118	0.0002	6.52661	6.13999	2.98829	3.84863	2.8637	6.26195
Tacc2	-2.01208	3.9E-05	4.83492	3.19957	1.86295	2.36149	2.20821	2.72356
Pxylp1	-2.0123	0.00084	1.59118	0.75154	0.75491	0.9265	0.86291	0.9142
Hspa2	-2.01267	2.2E-05	5.58174	4.16914	3.1447	4.21637	2.89427	4.60257
Hpn	-2.01315	0.01103	0.86861	0.77233	0.73735	0.75568	0.53844	0.91233
Vps9d1	-2.01441	0.01567	1.82569	2.21876	1.23434	1.93308	2.02571	1.85874
Dedd2	-2.01538	0.00019	3.22224	2.80281	1.60391	2.48895	1.79002	2.75244
Dvl1	-2.01544	0.00106	6.3439	5.58204	4.38291	5.39442	5.2502	4.47835
Fdxr	-2.0162	0.02118	0.87887	1.23889	0.85468	1.10443	0.90617	0.69335
Nhsl1	-2.01733	0.00057	1.15098	0.7284	0.66157	0.80464	0.82551	0.63962
Stx4a	-2.01827	0.00404	9.04103	10.2937	7.23372	7.9592	8.57645	8.38818
Scarf1	-2.019	0.00017	3.57504	4.38839	1.95035	3.04781	2.41046	3.44726
Pank1	-2.01912	0.00423	1.95441	1.00436	0.42364	1.21412	0.74308	0.87152
Usp20	-2.02104	0.00023	1.33977	1.5074	0.85595	1.12452	1.02653	1.19831
Pnmal2	-2.02126	0.00821	0.85897	0.55885	0.35201	0.50709	0.5002	0.52328
Ak8	-2.02178	0.00456	1.75555	1.15486	0.9464	1.27048	0.75023	1.35835
Adcy6	-2.0222	3.3E-06	9.33565	8.52504	4.10709	6.91633	5.70492	5.70824
Zfp7	-2.02235	0.0006	2.37498	2.2319	1.99581	1.77871	1.73989	2.48619
Cdk11b	-2.02411	0.00987	11.4415	10.6654	9.97693	8.83491	10.4864	9.51317
Casz1	-2.02436	7.5E-05	6.14197	6.61038	3.97501	5.21075	4.12187	5.98068
Chchd6	-2.02454	0.00146	2.34755	1.46623	1.19568	1.33481	1.11125	2.01201
Taz	-2.02461	0.00099	3.49072	3.29803	2.21606	2.97127	3.06873	2.05068
Agmo	-2.02717	0.00068	1.56952	0.90957	0.38158	0.75724	0.63879	1.03582
Tctex1d4	-2.02721	0.03545	4.7683	2.57274	3.47259	2.63997	2.65003	4.8494
Csad	-2.03008	0.00014	5.56494	5.18422	3.13045	4.22684	3.6337	4.55911
Coq4	-2.03011	0.004	0.96739	0.68183	0.58286	0.65125	0.62996	0.70448
Wnt2b	-2.03069	0.01311	1.43145	0.83817	0.43996	0.51704	0.68567	1.15017
Intu	-2.03204	0.00302	1.43868	0.90481	0.8563	1.03557	0.89182	1.01746
Atat1	-2.03263	0.03119	1.27466	0.84226	0.93221	1.26705	1.21103	1.02796
Caskin1	-2.03337	0.0275	0.28118	0.2253	0.16894	0.24209	0.21683	0.10912
Napb	-2.03654	0.01591	0.56997	0.46016	0.07394	0.26685	0.29419	0.38205
H2-Q7	-2.03774	0.04561	10.8791	24.5249	10.6105	18.6845	13.4768	12.4218
Ing4	-2.03837	0.00162	2.95302	2.78803	1.76399	2.36131	2.30172	2.1661
Dynlt1c	-2.0411	0.01915	3.28883	3.02707	1.79445	2.60802	2.49796	2.06042
Cadps2	-2.04397	1.7E-07	2.50161	2.22081	1.16632	1.71816	1.39199	2.06511
Prkcg	-2.04572	0.01837	1.01149	0.72718	0.49076	0.5822	0.87069	0.43505
4931414P19Rik	-2.04703	0.01923	0.70667	0.71723	0.3227	0.64224	0.38828	0.46316
Obsl1	-2.05014	0.00493	1.61812	1.51456	1.18224	1.62745	1.24075	1.0782
H2-Q6	-2.05148	0.01605	18.2237	35.0875	19.0039	23.6223	20.4752	19.1648
Slc2a4rg-ps	-2.053	0.0027	2.45531	1.60941	0.52556	1.58061	0.96683	1.25721
Ccdc134	-2.05306	0.00088	2.63775	2.57128	1.88842	2.43788	1.96515	1.86798
Barx2	-2.05325	0.03	1.2067	0.78508	0.84774	0.59052	0.71401	1.21673
Epha1	-2.05492	6.6E-06	2.74385	1.86836	1.14146	1.59201	1.48204	2.15267
Unc13b	-2.05497	1.1E-06	2.6864	2.0106	1.11395	1.69577	1.59149	1.68765

Ephb6	-2.05544	0.00713	0.60521	0.6552	0.38691	0.51336	0.30149	0.63465
Fgfr2	-2.05646	7.2E-07	7.30448	6.3594	2.33315	4.50974	3.14234	5.89123
Eef2k	-2.05696	3.5E-06	2.93444	2.15863	1.40755	1.88251	1.73425	2.04042
Jund	-2.05706	0.01897	63.3559	63.3575	36.57	58.6576	43.8467	67.4716
Tspyl5	-2.05798	0.04986	0.50135	0.32766	0.39024	0.31757	0.41737	0.31739
Usp35	-2.06058	0.03426	0.94999	1.28558	0.98593	1.23489	1.06292	0.67923
C2cd5	-2.06083	2.9E-05	2.11045	2.10091	1.37522	1.58987	1.75133	1.64484
Cep83	-2.06105	0.00072	9.11541	7.91662	7.54275	7.69124	7.77593	7.51798
Exoc3l	-2.06119	0.01056	1.45351	2.08551	0.50373	1.36816	1.38809	1.02068
Cfap45	-2.0612	0.00792	2.86758	2.96431	1.93993	2.40235	1.65987	2.90035
Dnajb2	-2.06193	1.4E-05	6.59935	6.05433	3.70558	5.47017	3.66929	5.45939
Tdrp	-2.06222	0.00462	1.22003	0.89244	0.45172	0.83909	0.5556	0.78341
Bckdhb	-2.06425	0.00094	2.68193	2.13994	1.55974	1.6556	1.81648	2.07282
Gm28043	-2.06498	0.02109	2.40915	2.1087	1.39114	2.16403	2.18564	2.00814
Fgfr3	-2.06503	0.00034	17.5836	11.5262	5.91425	9.40113	7.8991	11.2379
Brd9	-2.06503	0.00276	6.37769	6.61424	5.50394	5.8652	5.53439	5.81588
4933407K13Rik	-2.06527	0.01406	0.72423	1.12899	0.55119	0.69691	0.79064	0.81437
Fgfr4	-2.06662	0.00213	1.95981	1.39585	0.47102	1.31241	0.69898	1.18307
Cetn4	-2.06695	0.02156	1.26858	0.91966	0.72411	0.80978	0.49787	1.05785
Usp43	-2.06745	0.0004	1.84793	1.62807	1.69208	1.37621	1.48069	1.57701
Fxyd3	-2.06769	0.0493	0.67019	0.51825	0.3279	0.45246	0.21042	0.59612
Morn4	-2.06776	0.04516	0.85503	0.46793	0.68902	0.63994	0.33162	0.7417
Tatdn3	-2.06777	0.02717	1.14746	1.44188	0.93567	0.73495	1.24892	1.15493
Phlpp1	-2.06813	7.5E-06	7.79183	6.68173	3.27393	6.31097	4.57849	4.18918
Ablim2	-2.07005	0.0312	0.58325	0.27406	0.20715	0.17277	0.32284	0.4035
Arhgef15	-2.07028	0.00011	8.36874	6.6849	3.40977	6.44479	4.7375	4.43225
Tst	-2.07078	0.00284	11.6672	7.82385	5.51802	6.31157	4.57892	11.2653
Epm2a	-2.07212	0.00394	1.63946	0.89308	0.41239	0.80813	0.63107	1.02443
Gm12258	-2.07478	0.00186	1.01051	1.32561	0.824	0.93712	0.92159	0.96303
RP23-54G8.4	-2.07493	0.02021	0.88463	0.8729	0.39274	0.62531	0.68407	0.50329
D2hgdh	-2.07669	0.00011	2.45359	1.94889	0.80377	1.56415	1.32858	1.32843
Klrg2	-2.07699	0.00456	0.85412	0.9933	0.51801	0.54984	0.64528	0.87468
Fahd2a	-2.07732	0.02949	1.1862	1.16168	0.42763	1.02131	0.59458	0.71607
Cbs	-2.07754	0.02929	0.65573	0.41595	0.40548	0.31453	0.3803	0.58198
Odf2	-2.07779	4.5E-05	4.22663	3.55145	2.8173	3.01276	3.32576	3.16848
Spag1	-2.07834	0.00014	1.7961	1.05325	0.65614	1.08822	0.89475	0.94174
Cnn1	-2.07937	0.02664	15.056	4.15059	2.32477	6.5534	3.81523	6.18104
Arvcf	-2.07972	2.2E-05	2.85741	1.97187	1.08734	1.74848	1.32264	1.94497
Dock9	-2.08001	1.2E-09	16.3694	14.8767	7.48281	10.2729	10.2753	12.6202
Mrvi1	-2.08009	0.00215	2.86308	1.04104	0.88586	1.15916	1.29377	1.4797
Polr3gl	-2.08062	0.00068	6.7488	6.24012	3.46048	4.4355	3.64525	4.12332
Ccdc85a	-2.08165	1E-05	4.12729	3.67918	1.76503	2.63561	2.63007	2.83386
Rdm1	-2.08239	0.00046	2.66764	1.96195	1.32408	1.79196	1.44448	1.8054
Por	-2.08268	4.1E-05	41.1371	20.1535	14.3921	21.4285	15.6758	26.8722
Kctd13	-2.08277	0.00648	1.90975	1.98798	1.34165	2.00265	1.5605	1.47431
Pfkfb2	-2.08349	0.00015	6.17126	5.99746	3.2008	3.98174	3.78201	4.50894
Anks1	-2.08608	4.1E-07	8.70795	8.53306	4.53989	5.33798	5.58313	7.63689
Mapt	-2.08729	1.2E-05	9.49639	7.21126	2.53375	6.03293	4.47807	4.96806
Tle6	-2.0921	0.00584	0.70988	0.57539	0.4052	0.53763	0.43338	0.43618
Igfbp3	-2.09341	0.02361	3.56012	2.44343	0.4997	1.32603	1.62473	2.10382
Sec14l2	-2.09376	0.0007	1.82415	1.65303	0.59499	1.1368	0.99272	1.28095
Gm16432	-2.09685	0.00535	0.9726	0.54351	0.35689	0.45248	0.59801	0.54069
Cep290	-2.09714	1.5E-05	3.76485	2.93337	2.46157	2.73036	2.57423	2.7666
Sh3glb2	-2.09723	0.00359	3.09037	3.81418	3.37118	3.25936	2.88448	3.58204
Creld1	-2.09773	0.0033	3.23128	4.12981	2.81446	3.37898	2.61271	3.31396
Fam167a	-2.09804	0.00266	4.31684	3.45229	3.22036	3.21111	2.78675	4.07935

Rpgrip1	-2.09903	0.01509	0.21797	0.24157	0.11155	0.19127	0.21475	0.14163
Pnpla6	-2.09952	3.8E-06	3.02324	2.82784	1.89233	2.41377	2.2424	2.11416
Mettl24	-2.1003	0.00466	1.61289	1.56626	0.81835	1.15819	0.78773	1.46819
Cd300lg	-2.1015	0.02729	1.76869	1.2419	0.30478	0.82899	0.44006	1.47635
Map3k13	-2.10329	0.0003	0.5718	0.62179	0.41963	0.48667	0.39895	0.50191
Szt2	-2.10495	3.2E-05	4.06726	4.87552	2.31288	3.22858	3.39411	2.99391
Ppp1r26	-2.10516	4.1E-05	1.10535	0.90739	0.51959	0.80759	0.66687	0.63922
Cd24a	-2.10552	1.4E-05	57.1037	39.075	36.7375	37.2364	28.4252	48.4358
Hip1r	-2.10658	0.0003	5.71402	5.5352	4.45726	4.80628	4.93065	4.70156
Trim14	-2.10908	0.0066	1.53703	1.5315	1.3701	1.78598	1.31112	0.96598
St5	-2.10953	7.8E-08	12.4161	13.1401	6.77819	9.8084	9.36413	8.41141
Fam221a	-2.10955	0.0193	0.94221	0.41053	0.45633	0.39788	0.50201	0.60818
Hemk1	-2.11031	0.01643	0.90185	0.8899	0.57198	0.74373	0.80751	0.43099
Nlrc5	-2.11338	0.02319	2.51654	5.3575	3.78142	4.16368	4.37265	2.43174
Exd2	-2.11424	3.7E-06	3.16095	3.06868	1.66307	2.81446	1.83241	2.02666
Trim39	-2.11431	9.3E-06	3.21193	2.9305	2.07879	2.59729	2.39936	2.15484
Mecom	-2.11433	9.1E-07	3.65972	2.79601	1.95103	2.53637	2.1689	2.75782
Wdpcp	-2.11504	0.00133	1.43148	0.80715	0.91374	0.80203	0.89082	1.05926
Dzip1l	-2.1157	0.00079	4.68951	4.3646	3.29278	4.13711	3.82362	2.89349
Sclt1	-2.11678	1.4E-05	1.85431	1.56834	0.94084	1.23272	1.17921	1.27651
D430042O09Rik	-2.11761	3.6E-06	3.34396	3.37008	2.25246	2.75574	2.45915	2.6346
Cadm4	-2.11839	0.01216	2.40918	1.25119	1.01328	1.45968	0.86699	1.59284
Zc2hc1c	-2.11853	0.00016	1.2431	0.74115	0.58161	0.70379	0.65689	0.76799
BC022687	-2.12015	0.00663	2.71866	2.13804	1.68795	1.99397	1.60732	2.03191
Oscp1	-2.12361	0.00012	1.25168	0.94102	0.69461	0.7373	0.76414	0.93999
Ppp1r12b	-2.12609	1.2E-06	6.91642	4.11548	2.51402	3.69119	3.95114	3.51647
Zfp783	-2.1292	0.00389	0.4935	0.39483	0.35021	0.33941	0.41039	0.32781
Rsrp1	-2.13109	3.8E-08	51.3552	45.7882	29.8274	42.8475	34.9052	31.6163
Dus3l	-2.13136	0.01674	4.75923	5.2664	4.16543	4.3253	4.09673	4.38641
Krba1	-2.13178	0.00021	1.40944	1.27544	1.01267	1.14657	0.92424	1.18699
Rhpn2	-2.13304	8.2E-05	2.11756	1.393	1.34301	1.45406	1.36171	1.29148
Trmt13	-2.13378	0.01878	0.97283	0.8881	0.8109	0.84203	0.88237	0.56928
Ccdc61	-2.13621	0.01351	2.28129	2.43915	2.06381	2.39928	1.70279	1.93949
Ulk3	-2.13721	0.00064	1.86762	1.65393	1.17892	1.34456	1.36821	1.27805
Unc45b	-2.13768	6.8E-05	4.18775	3.21524	1.23	2.46338	2.2637	2.59811
Mlf1	-2.13815	0.00142	4.30387	2.29194	1.51646	2.15693	1.40133	2.96007
Sox11	-2.13969	0.00404	1.60453	1.48722	0.40148	0.95884	0.6763	1.15246
Pi16	-2.1407	0.00189	3.96323	2.51887	1.62148	1.72113	2.03073	2.96538
Tmc4	-2.14128	0.0001	5.97536	6.46171	5.23794	5.44348	3.86159	6.66117
Actr5	-2.14155	0.001	1.7674	1.82702	1.22057	1.441	1.53456	1.10831
Ddx55	-2.14183	0.00052	1.8906	1.79515	1.36422	1.49129	1.54184	1.3151
Dnal1	-2.142	1.8E-07	3.05424	2.54982	1.60811	2.32112	1.75529	1.98451
Chd1l	-2.14276	0.00026	2.24688	2.00794	1.30672	1.63104	1.50629	1.54555
Lamc3	-2.14282	0.00044	3.10407	2.54927	1.18121	1.36283	1.66981	2.54303
Plcg1	-2.14286	9.2E-05	13.9035	13.4505	7.47166	9.36065	8.99331	9.30481
Klf5	-2.14418	0.00042	10.1899	5.65585	6.05877	7.23502	3.88805	8.17422
Kcp	-2.14427	0.02169	0.29249	0.30675	0.17363	0.20557	0.21427	0.21087
Slc1a5	-2.14537	3.3E-09	18.7578	13.514	8.89543	11.7761	10.118	12.6956
Nek1	-2.14777	1E-06	2.93031	2.28248	1.41479	2.05612	1.62913	1.88828
Rhobtb1	-2.15157	8.4E-06	3.77378	2.14906	1.2873	1.831	1.68522	2.31569
Dhrs11	-2.15247	0.0135	1.3675	1.25299	0.65048	1.0702	0.87659	0.70021
Zdhhc14	-2.15321	2.2E-05	2.60965	2.18239	0.9038	1.51008	1.58959	1.51339
Anks6	-2.15394	0.00556	0.71507	0.87032	0.49776	0.66573	0.52386	0.57154
1700088E04Rik	-2.15455	0.00179	1.86403	1.40139	0.61081	0.73201	0.60692	1.01808
Wdr19	-2.15613	1.7E-06	1.61165	1.00692	0.78587	0.98138	0.88403	0.95543
Mapre3	-2.15641	7E-06	6.04692	5.47172	3.55711	4.19169	3.81091	4.82418

Scube3	-2.15755	0.00017	1.62425	0.82961	0.43346	0.88186	0.61814	0.76903
Clec9a	-2.15794	0.00011	6.43496	7.06127	2.18486	4.65507	3.90141	3.93119
Adcy9	-2.16053	5.8E-08	4.93859	4.17698	2.86912	3.82278	2.88408	3.44124
Smarca2	-2.16074	2.6E-08	12.3277	12.0254	6.39232	10.2103	7.16061	9.13067
Rnf31	-2.16211	0.00011	4.32334	5.95595	3.53463	4.59831	3.51302	3.99046
Armc9	-2.16212	0.00024	1.02532	0.82562	0.52112	0.67228	0.76658	0.55233
Tmem8	-2.16316	1.7E-05	2.7686	3.07758	1.62548	2.10879	2.27938	1.79296
Rsph9	-2.16328	0.00017	3.53491	2.55577	1.6923	2.31446	1.29483	2.80258
Plekha5	-2.16733	1.3E-05	2.98731	2.73304	1.8389	2.42511	2.05975	1.9435
1110038F14Rik	-2.1675	0.00175	7.05379	6.88382	5.16195	5.86274	4.77006	5.77887
Gnb5	-2.17028	0.00257	1.29016	1.2896	1.23854	1.26136	1.07405	0.81675
Trim45	-2.17154	0.01512	0.31968	0.30901	0.43446	0.37816	0.2342	0.31179
Eva1c	-2.17158	9.6E-05	2.35183	1.23814	1.15066	1.06143	1.21309	1.63184
4933427D14Rik	-2.1719	8.8E-06	1.97201	1.73569	1.06157	1.44736	1.17454	1.32661
Rnf215	-2.17308	2.2E-06	7.52962	7.06064	4.48479	5.47944	4.76667	5.79991
Bbs1	-2.17317	6E-06	1.75114	1.43956	0.74733	1.00149	0.97115	1.19329
Stx1b	-2.17429	0.04653	0.36139	0.28957	0.32571	0.19591	0.3623	0.24933
Fam214a	-2.17443	0.00017	3.12395	3.27072	2.18378	2.40137	2.84308	2.00686
AU020206	-2.17766	0.00061	13.2272	17.5465	5.88117	11.3042	9.89419	8.327
Slain1	-2.17773	0.01496	0.77876	0.98799	0.85729	0.89312	0.73352	0.66079
Dapk2	-2.17868	0.0002	3.97911	3.60743	1.39543	2.28482	2.0958	2.81248
Fuom	-2.18292	0.00578	0.58621	0.66108	0.44615	0.49048	0.34765	0.526
Zfp704	-2.18566	0.00074	4.70609	3.5904	2.08475	4.5781	3.66258	4.71881
Col27a1	-2.18605	0.00846	1.51505	2.41449	1.42531	1.76363	1.71111	1.16651
Frs3	-2.18649	0.01323	0.6132	0.54333	0.36668	0.51306	0.40644	0.33491
Polm	-2.18749	0.00026	1.77282	1.79508	1.22572	1.48259	1.29875	1.20692
Dgka	-2.18867	0.0007	9.17279	11.0871	7.53814	9.24302	7.31566	7.70141
1810019D21Rik	-2.19183	0.01001	3.59226	2.73942	1.81517	1.89105	1.59626	3.18421
Tbc1d17	-2.19205	0.00013	2.13177	2.22399	1.49021	1.92318	1.6718	1.62337
Syde2	-2.19635	3.3E-06	3.55758	1.92088	1.16038	1.49151	2.01287	1.65246
Dtx1	-2.19779	0.00967	0.41134	0.69581	0.42487	0.629	0.2583	0.40118
Dnaaf2	-2.20006	0.00093	2.23629	3.05361	1.75324	1.66509	1.38169	1.83209
Dynlrb2	-2.20084	0.0176	17.5776	9.30911	5.62616	8.46023	3.36973	14.5351
Ift88	-2.20319	2.7E-05	3.47351	2.57061	2.16293	2.46327	1.735	2.74869
Gpd1	-2.20392	0.00546	6.01514	2.2959	1.69032	2.5044	1.37849	4.06871
Tspan1	-2.20534	1.7E-05	10.0005	7.33046	5.95453	6.51057	4.19465	9.18895
Pan2	-2.20685	2.5E-05	4.16664	4.39207	2.43691	3.17116	3.08252	2.75774
Mmp28	-2.20829	0.00014	4.07883	3.2504	0.96897	2.42844	1.77906	1.7191
9230110C19Rik	-2.20905	0.00878	1.54907	1.31017	0.91696	1.21664	0.5464	1.31609
Fbf1	-2.20959	0.00038	3.382	3.0797	2.32731	2.4989	2.37404	2.59695
Stk11ip	-2.20979	0.00021	2.57647	2.7979	2.28769	2.42829	2.15549	2.05867
Cep250	-2.21001	0.00017	3.8039	4.47588	2.64373	3.30769	3.52209	3.12358
Aldh5a1	-2.21172	0.00649	1.37484	0.88921	0.59088	0.65659	0.73466	0.92758
Gramd2	-2.21297	3.3E-09	11.8897	8.81007	4.77359	6.86113	6.32286	7.02207
Sidt1	-2.21461	0.00636	0.69847	0.52206	0.51922	0.66924	0.4284	0.33269
Whrn	-2.21641	0.00226	0.57265	0.53698	0.56847	0.52044	0.48336	0.41815
Acad12	-2.21769	0.00015	1.07934	0.66392	0.38406	0.56053	0.52372	0.49381
Scgb3a2	-2.21903	0.01166	236.855	97.4425	111.618	146.399	59.4616	173.807
Zfp94	-2.21908	0.01352	0.7026	0.37415	0.4456	0.47299	0.45753	0.25583
Trim65	-2.22092	7.6E-05	2.42201	1.98283	0.92166	2.3705	1.95633	2.27679
Il17rc	-2.22149	0.0002	4.03513	4.62759	2.88884	3.16997	3.08137	3.38985
Map3k12	-2.22468	0.00043	1.60598	1.27234	0.85868	1.15779	1.39248	0.89935
Epb41l4b	-2.22533	7.8E-07	3.11402	1.79655	1.04766	1.71805	1.2313	1.72331
Ccp110	-2.22588	6.3E-09	3.51809	2.76968	1.74291	2.27231	1.856	2.37466
Slc8a2	-2.22683	0.0416	0.46875	0.17621	0.11892	0.15778	0.21622	0.21335
Ppp2r3d	-2.22893	0.01762	2.09324	1.95768	1.74628	1.71946	1.75467	1.48405

A330076H08Rik	-2.23196	0.00669	0.82138	0.67734	0.37508	0.49766	0.4513	0.53834
Ogfod2	-2.232	0.001	2.38448	2.93967	1.89165	1.71407	1.89489	2.41704
Cyb5rl	-2.23229	0.00035	0.62695	0.45748	0.39295	0.36496	0.43228	0.38271
Pon1	-2.2325	0.01615	5.57859	1.67939	1.25932	2.53975	1.47773	2.3497
Spata1	-2.23275	0.04866	0.81967	0.43095	0.15174	0.26844	0.48688	0.29039
Rsad1	-2.23365	0.00154	1.60168	0.59817	0.52243	0.64275	0.65527	0.80095
Otud3	-2.23436	0.00018	5.14732	4.86297	3.68826	3.78222	3.24939	4.39642
Fam81b	-2.23476	0.00542	0.80007	0.74218	0.67766	0.60985	0.41596	0.82462
Rsph3b	-2.23646	0.00628	7.35681	6.27012	4.4577	5.04025	4.68476	7.18617
Smyd3	-2.237	4E-05	1.15365	0.83274	0.7834	0.68693	0.69944	0.91255
Car8	-2.23763	4.6E-06	8.79477	7.97981	5.46661	6.23345	4.23744	8.27333
Mall	-2.2396	0.00377	1.82129	1.07829	1.19554	1.43453	0.7672	1.08177
Hist2h2be	-2.24111	0.00475	1.1853	0.91405	1.03476	1.03498	0.61295	0.9425
Cc2d2a	-2.24279	2.7E-07	3.31332	2.33718	1.68451	1.99123	1.87699	2.01112
Olfml2a	-2.24319	1.6E-06	4.09292	2.28474	1.45782	2.24176	1.81106	2.01189
Paxbp1	-2.24358	0.00029	6.10244	6.15714	3.82404	5.10397	4.34912	3.79035
Hdac7	-2.24435	1.3E-05	14.9807	16.2326	10.3894	12.6391	10.8901	11.2311
Muc4	-2.24572	0.00266	6.26164	6.91238	8.02634	7.78062	4.2799	7.86668
Ciz1	-2.24581	0.00187	3.58811	3.62735	3.0055	3.07444	3.14191	2.66128
Slc16a7	-2.24663	0.0001	3.24029	1.50079	0.96882	1.61733	1.10778	1.64334
Nr3c2	-2.24712	4.1E-06	2.22912	1.14553	1.01469	1.17963	0.8682	1.42168
Proca1	-2.24845	0.0449	0.6999	0.92495	0.24969	0.55658	0.54479	0.35838
Mzf1	-2.24901	0.00516	0.30895	0.27997	0.26452	0.27409	0.2425	0.16271
3110062M04Rik	-2.24915	0.00384	1.60467	1.17624	0.51293	1.1278	1.41068	1.59509
Sec14l3	-2.24962	8.3E-06	177.688	111.965	72.0986	100.284	63.5725	125.924
Sdccag8	-2.25049	5.5E-05	3.38451	4.02255	2.29806	3.23006	2.52809	2.75466
Cables1	-2.25159	0.0002	3.71051	2.79716	1.41198	2.4762	1.7334	1.93846
Bspry	-2.25187	2E-05	2.79605	2.16778	1.72899	1.69408	1.47938	2.22705
Cfap53	-2.25203	0.00085	2.31857	2.94151	2.44637	2.25616	1.75004	2.64779
Ccdc181	-2.25246	0.00019	8.03809	7.00081	6.22679	6.58049	4.59175	6.97724
Sobp	-2.25269	0.01942	0.52863	0.36327	0.60349	0.42038	0.45989	0.39248
Ptms	-2.25346	0.0195	92.6352	117.858	90.682	101.553	76.0472	97.5688
Zfp276	-2.25351	0.00296	2.98953	3.19903	1.81014	2.30166	2.66201	2.368
Scrib	-2.25418	0.0002	7.13376	7.25919	5.19903	5.68256	5.41279	5.29647
Gramd1c	-2.25613	0.00053	1.01931	0.67738	0.74806	0.59553	0.52005	0.89473
Cpe	-2.25623	0.02168	8.19203	2.30956	1.07908	2.92714	2.12871	3.04019
Rfx5	-2.25656	0.00063	2.30961	2.06586	1.65029	2.58729	2.27629	2.28693
Foxq1	-2.25782	0.00077	2.34111	1.22298	1.45422	1.4393	1.28631	1.94617
Zfp839	-2.2589	0.00019	2.84434	2.34348	1.37162	1.97589	1.63466	1.5468
Bbox1	-2.25918	0.02839	0.49787	0.24563	0.14209	0.22624	0.15197	0.30591
Magi1	-2.26035	2.7E-06	3.3413	3.16923	2.06986	2.20753	2.20746	2.60299
Calcoco1	-2.26079	1.3E-09	10.4175	8.14818	4.65709	6.67485	4.76143	6.9863
Macrod1	-2.26335	0.00071	3.44129	2.06166	1.71876	1.82439	1.20801	2.70183
Irx3	-2.26428	0.00018	6.31625	4.97857	2.71816	3.22582	1.96234	5.44555
E4f1	-2.2649	0.00024	4.52943	4.34371	3.05364	3.34935	3.16146	3.21405
Fos	-2.26568	0.0269	18.2132	15.6014	12.7679	10.0233	14.9929	13.585
Med12l	-2.26718	0.0005	0.45086	0.25422	0.34313	0.30785	0.24641	0.26383
Neurl4	-2.26743	1.3E-05	5.42845	5.33902	3.09857	4.00319	3.54508	3.58325
Zbtb32	-2.26846	0.03871	0.62003	0.42608	0.30967	0.37566	0.36906	0.28573
Aqp4	-2.26974	0.00019	1.29404	1.17048	1.12848	1.00182	0.869	1.11905
Nr1d2	-2.27053	7.7E-06	16.401	10.9058	3.98237	8.37678	6.48929	8.07991
Lmbr1l	-2.27264	0.00015	3.09729	3.24249	1.63195	2.03407	2.23732	1.98739
Calml4	-2.27337	0.00075	4.01941	2.05145	2.3734	1.80547	1.87845	3.06583
Plxna3	-2.27504	6.6E-05	0.78819	0.53919	0.50283	0.59694	0.45005	0.38934
Upf3b	-2.27563	0.00024	3.38034	3.41924	2.81603	2.65698	2.7776	2.58224
Srrt	-2.27604	0.0006	11.2343	12.124	8.94778	8.70621	9.73586	8.54739

Grin2d	-2.27651	0.00125	0.5213	0.48732	0.2819	0.34078	0.39195	0.39336
Amt	-2.27994	0.00048	1.42422	1.64838	0.47065	1.13541	0.68643	0.92116
Dnhd1_2	-2.28037	0.00896	0.421	0.3667	0.40888	0.30076	0.31763	0.33976
Dennd3	-2.28054	5.7E-05	12.1239	13.7761	8.4836	7.82113	9.51546	10.8642
Fuz	-2.28077	4.4E-05	1.98997	1.77572	1.08377	1.43797	1.36421	1.2115
Txlng	-2.28126	6.3E-09	3.65786	3.08351	1.61779	2.12083	2.0231	2.26243
Ano8	-2.28222	0.00518	1.61412	2.09856	1.37059	1.55181	1.38772	1.27866
Sh3gl2	-2.28279	0.03032	0.18125	0.16509	0.14325	0.14361	0.15321	0.12565
Gm28729	-2.28363	0.00059	1.5812	1.69136	0.79637	1.21162	0.88582	1.06681
Coq8b	-2.28558	0.02025	4.62007	5.27009	5.3784	4.84339	4.76521	4.45736
Gnao1	-2.28598	8.8E-05	3.04733	2.90246	2.22521	2.40632	1.9844	2.3691
Fbxl12	-2.28619	2.1E-05	2.40457	2.48905	1.51593	1.84829	1.9719	1.2643
Has3	-2.29013	6.7E-05	1.2312	1.1503	0.44127	0.82403	0.71318	0.59816
Sntb1	-2.29017	2.2E-08	5.41213	4.4967	2.32603	3.1248	2.58884	3.80796
Nr4a2	-2.29043	0.00566	2.88032	1.35129	0.6936	1.09071	1.18693	1.30891
Hsf4	-2.29197	0.01211	1.92783	1.8068	1.07067	1.19961	0.9161	1.70745
Pde4dip	-2.29239	2.3E-06	5.12579	2.63051	1.38559	2.59021	1.97679	2.11742
Ldhb	-2.29273	0.00017	23.5034	14.5647	11.357	15.1215	7.33063	17.5395
Hlf	-2.2982	0.0003	4.36113	2.09289	0.73354	1.90513	1.21187	1.99365
Ppargc1a	-2.29948	0.002	1.28859	0.42188	0.4429	0.47012	0.45678	0.70379
Mill2	-2.30005	1.4E-06	4.16826	3.69658	1.15498	2.74615	1.66023	2.42025
Myh14	-2.30095	2E-06	44.8462	40.0545	20.2005	27.202	21.0777	33.8803
Bik	-2.30224	0.00404	3.68062	2.45764	1.87976	1.58449	2.62544	1.90445
Fbxl2	-2.3032	0.0002	1.15097	0.92132	0.77429	0.97131	0.70768	0.67353
Cry2	-2.30434	2.2E-06	8.41115	7.08956	4.47113	4.35188	4.53147	5.68321
Map9	-2.30954	0.00067	1.12272	0.9959	1.00036	0.89592	0.65198	1.06562
Wnt11	-2.31435	0.00045	2.20782	1.73662	1.56269	1.29755	1.35865	1.79068
Rassf7	-2.31453	1.1E-05	4.24183	3.39268	2.26334	2.45831	2.7022	2.45527
Tbx3os1	-2.31585	0.0014	1.24014	0.78057	0.36217	0.59411	0.63385	0.54344
Arhgef1	-2.31647	0.00058	12.1437	15.9359	10.5458	11.3872	11.1291	9.36813
Cyp27a1	-2.31939	1.9E-05	2.90027	2.98626	1.48754	2.0119	1.90917	1.72179
Trp53bp1	-2.32127	0.00014	2.58588	2.19254	1.84043	2.27001	2.0314	2.0868
Grhl1	-2.32282	2.6E-05	2.27039	0.99324	0.715	0.98031	0.93679	1.02623
Usp13	-2.32462	0.00056	0.76926	0.35581	0.23327	0.30587	0.33461	0.36435
Myom3	-2.32537	0.04116	0.19665	0.2175	0.08635	0.17414	0.07757	0.12393
Nav2	-2.32564	7.4E-11	3.45251	2.85573	2.04308	2.08523	2.05687	2.50498
Capsl	-2.33144	0.00034	5.02067	4.40972	3.52715	3.74392	2.05125	4.50872
Dgkq	-2.33174	0.00021	2.23611	2.05245	0.98629	1.57036	1.75916	1.59255
Rccd1	-2.33357	0.00201	0.59398	0.78782	0.53939	0.56436	0.49448	0.54192
Rims2	-2.33562	0.04331	0.39822	0.13779	0.12399	0.12064	0.18565	0.17795
Hid1	-2.33582	7.9E-06	2.92201	2.94212	2.40539	2.11965	1.93677	2.70329
Ehf	-2.33602	4.2E-08	2.6045	2.14742	1.3838	1.56809	1.20002	2.01297
Capn10	-2.33744	0.00101	1.80742	2.04417	1.43955	1.57896	1.29331	1.25691
B3gntl1	-2.34768	0.00973	1.11641	1.09795	0.77062	0.86518	1.06514	0.61684
Eml5	-2.34864	3.4E-07	1.34267	0.7774	0.4012	0.58965	0.69976	0.58322
Clk2	-2.34928	0.00181	4.29195	4.8255	4.30939	3.55077	4.07869	3.58682
Rabl2	-2.34953	8.1E-05	3.42516	1.95739	1.90225	1.94437	1.5598	2.1488
2610307P16Rik	-2.35165	0.00037	0.88758	1.00875	0.79161	0.80665	0.6096	0.65899
Hipk4	-2.35375	0.03149	0.37577	0.33901	0.20019	0.15178	0.29364	0.24628
Mturn	-2.35382	1.8E-05	3.68879	2.71297	0.7873	1.92402	1.1867	1.94463
Mlph	-2.35411	0.01197	2.48932	2.40826	3.99023	2.52061	2.49816	3.1429
Zscan20	-2.35514	0.00118	0.74161	0.81172	0.51046	0.71364	0.35953	0.56289
Iyd	-2.35546	0.0207	1.06926	0.66637	0.38548	0.37507	0.41228	0.8299
Podnl1	-2.3562	0.01059	0.48899	0.91422	0.44071	0.51457	0.50906	0.40061
Colgalt2	-2.35689	0.00065	0.98976	0.87352	0.29476	0.5345	0.4256	0.65222
Rbm12b2	-2.35694	0.00813	5.95222	5.36842	4.02894	5.36165	4.68856	4.14278

Galnt12	-2.35818	0.03057	0.87825	1.11421	1.50392	1.33028	0.70189	0.98115
Trim29	-2.35955	0.00251	0.57859	0.64506	0.36028	0.36649	0.38533	0.45246
Zdbf2	-2.36178	0.00765	0.20924	0.07933	0.09061	0.08306	0.05814	0.14187
Noxo1	-2.36186	0.0111	0.69723	0.66663	0.69214	0.33061	0.48858	0.4222
Spire2	-2.3627	0.00569	1.67392	1.20033	1.74478	1.14648	0.79976	1.10306
Srsf5	-2.36293	0.00047	58.3342	53.9484	39.0364	34.5388	44.2621	39.7599
Etfbkmt	-2.3663	4.2E-05	4.35464	4.54968	2.5483	2.21714	2.57359	4.1072
Gm21992	-2.36646	0.00614	1.72246	1.25634	1.28084	0.95744	1.31974	1.04864
Faim2	-2.36678	0.00157	1.07099	0.85455	0.20762	0.6489	0.26647	0.64565
D2Bwg1423e	-2.3694	0.01182	1.2512	0.48842	0.60431	0.52482	0.35255	0.86735
Mettl7a1	-2.36961	2.3E-05	80.3266	50.9131	26.2478	43.7017	20.8593	51.502
Hdac5	-2.37333	2.1E-09	5.3575	5.00641	3.33716	4.02758	3.13227	3.5369
Sytl3	-2.37526	0.00676	0.40088	0.51751	0.52991	0.34515	0.37097	0.39757
Coq8a	-2.37704	0.0036	1.70837	0.68597	0.2364	0.73187	0.46775	0.65032
Ankzf1	-2.3787	3.9E-05	2.68222	2.88455	1.82505	1.75426	1.95196	1.58622
Otud1	-2.37931	9.3E-07	5.99887	3.98261	1.84095	3.41966	2.19737	2.95934
Srpk3	-2.37973	0.004	0.95143	0.44398	0.4869	0.61621	0.40859	0.32254
Arl10	-2.38222	0.00026	3.80353	3.75312	1.74891	2.59256	1.93503	2.42361
Cchcr1	-2.38408	0.03017	0.89547	1.26228	1.19265	0.91429	1.1541	0.70645
Usp11	-2.38812	0.00021	1.60208	1.79579	1.8069	1.68405	1.31508	1.26507
Nr6a1	-2.38872	3.4E-05	0.61601	0.75653	0.53947	0.37834	0.56874	0.54615
Abcc10	-2.38884	0.00011	1.03914	1.09297	0.83648	0.75874	0.8979	0.63303
Dsg2	-2.39177	7.1E-05	1.84487	0.79419	0.70624	1.09769	0.4694	1.15845
Setd4	-2.39291	0.00542	0.56333	0.54218	0.42134	0.40016	0.45539	0.31829
Pigv	-2.39294	1.2E-06	2.62213	1.92205	1.07265	1.5887	1.31265	1.18151
Atp8a2	-2.39365	0.0497	0.22767	0.12035	0.11371	0.11208	0.12509	0.09326
Nos1ap	-2.39375	0.00024	1.0458	0.99109	0.84867	0.73288	0.75692	0.7742
Zscan26	-2.39393	2.9E-05	8.00327	5.19607	2.76721	8.8245	6.42956	7.97764
Safb	-2.39553	0.00025	12.3152	12.9805	9.49698	9.23273	8.82836	9.74534
1700007K13Rik	-2.39671	0.00179	6.81483	5.28354	3.56576	4.18633	2.70427	5.04029
Mapk8ip3	-2.39733	7.1E-08	4.81219	4.73338	3.03689	3.55425	3.29557	2.87843
Elf3	-2.40011	0.00159	5.56183	6.58574	7.43116	4.36828	2.77674	4.3878
Dzip1	-2.40099	5.4E-09	2.86874	2.10065	1.56056	1.73812	1.21302	1.98745
4930516B21Rik	-2.40212	0.03429	1.52866	0.90504	0.34903	0.69464	0.78392	0.50095
Tmem44	-2.4024	0.00011	2.754	3.17869	1.09921	1.53687	1.78243	1.74731
Speg	-2.40315	0.00097	1.73485	1.21871	0.9657	1.07283	1.11338	0.7635
1600020E01Rik	-2.40414	0.00055	1.75162	1.60644	1.03597	1.36815	1.19046	0.70887
Ptpn13	-2.40637	5.7E-09	6.43786	4.83861	3.38611	3.9326	2.94702	4.22312
Gtpbp2	-2.40756	4E-05	6.88691	8.41687	4.81555	5.3097	5.41109	4.7394
Traf1	-2.40801	6.5E-06	2.30974	2.95588	1.46336	2.24202	1.62123	1.20868
Cyp39a1	-2.41033	9.2E-05	3.64991	1.79655	1.36615	1.74013	1.51229	1.66668
Kcnq1	-2.41281	6E-07	2.32082	2.35926	1.73234	2.01457	1.19342	1.73681
Snx29	-2.41311	0.00012	3.17943	3.43341	2.20372	2.47675	2.38122	1.89543
Fam149a	-2.41394	1.9E-10	4.21865	3.42534	2.34672	2.63036	2.18025	2.72722
Cspp1	-2.41536	9.7E-07	3.43866	2.85045	2.00622	2.17561	2.22933	1.85732
Wdyhv1	-2.41559	2.8E-05	4.14543	4.49953	3.62821	3.05583	2.68261	3.79239
2210011C24Rik	-2.4156	0.01347	2.00387	1.80418	0.29518	1.05745	0.80504	1.05915
Card10	-2.4157	4.2E-05	7.96806	7.92486	5.76946	5.92288	4.64826	6.55811
D930048N14Rik	-2.41595	0.00095	0.77247	1.05886	0.45613	0.581	0.64396	0.45827
Ccdc62	-2.41673	0.00063	0.95143	0.56142	0.68201	0.49602	0.48629	0.47131
Spef1	-2.41961	1.2E-05	5.35324	5.14637	4.02358	4.10969	2.70381	3.73191
Six5	-2.42327	7.6E-08	2.39962	2.20939	1.00561	1.43549	1.17938	1.49309
Zfp474	-2.42499	0.00017	2.64247	1.87866	2.27342	1.85626	1.37474	2.10211
Aasdh	-2.42579	1.7E-06	0.95885	1.06902	0.63024	0.83622	0.55344	0.58321
Oas1b	-2.42683	0.00645	1.00875	1.82271	1.57035	1.19459	1.37723	0.90155
Cacna1h	-2.4271	0.01696	1.1133	0.49059	0.40585	0.37978	0.68538	0.32958

Esrp1	-2.42846	3.6E-05	2.83936	1.97265	1.76956	1.58866	1.32659	2.32616
Uba7	-2.42877	0.00443	3.39854	5.391	4.58096	3.80399	4.68953	2.62773
Adamts10	-2.43266	5.2E-07	16.3312	16.6744	6.42494	10.2399	9.41179	8.35702
Nrg2	-2.4339	0.00233	0.77078	0.72713	0.27075	0.467	0.49952	0.29145
Pnisr	-2.43683	6.8E-07	10.8848	10.9807	6.69417	7.42609	7.42897	6.6624
Efcab11	-2.43866	0.0361	0.4856	0.33604	0.70556	0.37446	0.35575	0.39783
1810010H24Rik	-2.43908	0.00028	9.16086	5.96008	2.90846	4.86071	2.8592	5.15143
Exosc7	-2.43979	2.7E-05	4.87187	4.65655	2.66783	2.99978	2.26333	3.89396
Taf1a	-2.44037	1.9E-05	2.9953	2.72578	1.68808	1.88371	1.61103	1.98798
Rps6kb2	-2.44298	0.00374	3.32476	3.60192	3.66763	2.82924	2.98305	2.75444
Cdkl4	-2.44363	0.00013	2.11906	1.39691	1.20956	1.13285	1.09582	1.25098
Strbp	-2.44554	1.9E-08	2.69361	1.97528	1.27707	1.525	1.32633	1.54353
Cap2	-2.4469	0.00021	1.97962	0.69764	0.7847	0.86625	0.54385	1.08121
Tmie	-2.44693	0.00831	0.94467	0.5493	0.1348	0.42927	0.25952	0.45952
Sfswap	-2.44997	0.00011	6.6416	7.26145	5.49097	5.0055	5.33116	4.80824
Spaca6	-2.4531	0.03074	2.18944	2.01281	1.30407	1.24003	1.74344	1.01384
Gpatch2	-2.45672	0.00193	2.79338	2.75468	1.62839	2.29022	2.5689	1.74315
Gli1	-2.457	0.00017	1.42053	1.1593	0.65436	0.60398	0.76681	0.98002
Igsf9	-2.45755	0.00043	0.44205	0.66596	0.34694	0.43522	0.31372	0.3282
Lat	-2.45851	0.0224	1.4901	3.40748	2.33114	2.77535	1.53638	1.49206
Myh11	-2.45854	1.2E-05	49.2634	17.4026	13.8672	17.2061	15.9918	22.3065
Cep112	-2.45862	5.6E-06	1.3904	1.21351	0.87808	0.80657	0.93191	0.82403
Snapc4	-2.4592	0.0003	1.93103	2.11153	1.40141	1.57111	1.61675	1.15261
Nme5	-2.45982	1.8E-05	5.38211	4.15044	3.97569	3.64457	2.78322	3.69606
Ggt6	-2.46034	0.03902	0.70337	0.33994	0.40149	0.21308	0.29445	0.53508
Als2cl	-2.46084	2.3E-06	9.75013	9.0869	5.03367	6.34014	6.10784	4.84614
Neurl1a	-2.46368	2.8E-06	1.59442	1.1734	0.75645	0.7653	0.68263	1.11963
Sync	-2.46407	0.02193	1.21273	0.37789	0.47363	0.73477	0.25718	0.36604
Rgl2	-2.46512	3.7E-05	8.70916	9.2034	6.44792	6.68864	6.26453	5.66273
Efcab1	-2.46563	0.00025	3.29606	1.51778	1.92321	1.59762	1.23417	2.22026
Klc1	-2.46702	2.4E-05	17.123	17.6901	12.2718	13.733	11.5578	12.8882
Trim46	-2.46961	0.00025	1.01165	1.34457	0.76994	0.74429	0.847	0.7104
Tcea3	-2.46966	2.9E-05	6.19791	4.30403	3.79846	3.71568	2.19858	4.86388
Ift122	-2.47097	1.5E-05	1.79228	1.34097	1.36405	1.06503	1.1026	1.2895
Sgsm3	-2.47129	3.3E-05	4.27555	3.29711	3.32578	2.70477	2.59485	3.17654
Rab44	-2.47242	0.01366	0.68909	1.10924	0.65979	0.65993	0.6351	0.56454
Foxo6	-2.47244	0.01917	0.62871	0.32496	0.31899	0.31744	0.28147	0.28615
C030037D09Rik	-2.47251	0.02161	0.4568	0.31276	0.09933	0.11861	0.2709	0.23167
Cckar	-2.47275	0.00042	5.75511	2.97929	1.64252	2.40477	1.30846	3.59715
Nrxn2	-2.47285	6.8E-05	0.53565	0.34118	0.15854	0.24441	0.18894	0.2763
5430405H02Rik	-2.47514	0.041	0.71672	0.68197	1.17619	0.62424	0.52507	0.84407
2900052L18Rik	-2.47611	0.00095	2.47325	2.33507	1.9042	1.21971	1.72761	2.07335
Mapk12	-2.47664	3.3E-09	7.68802	5.60269	2.64957	3.72057	3.22346	4.31782
Aoc3	-2.47752	0.03541	2.41142	0.5684	0.36104	0.70657	0.59369	0.84205
Dgat2	-2.48143	0.0309	5.27346	1.05241	0.50732	1.23855	0.97668	2.06307
Synpo2	-2.48237	6.2E-06	5.13765	2.18149	1.61907	2.2788	1.95576	1.90803
Foxa1	-2.48241	9.8E-06	7.01936	3.00477	3.30346	3.02043	2.14089	4.71788
Kcnmb2	-2.48541	0.00034	1.7228	0.815	0.83337	0.81382	0.62036	0.98081
Palmd	-2.48569	4.8E-07	4.62681	3.44693	1.39576	2.57091	1.80894	1.76761
Luc7l3	-2.48789	1.9E-05	11.0187	11.3787	7.96578	8.16639	7.92515	6.85778
Ppip5k1	-2.48944	2.9E-07	1.78491	1.50964	1.09399	1.08588	1.07181	1.08474
Me3	-2.48959	0.01927	0.23734	0.10037	0.04278	0.06055	0.08236	0.1228
Gm12992	-2.49068	0.01464	0.48212	0.72345	0.1953	0.32037	0.41016	0.4332
Dusp18	-2.49152	4.7E-06	3.43108	2.90194	2.65631	2.16246	2.09465	2.61707
Ppp1r16a	-2.49249	1.1E-05	5.92064	6.4142	4.73386	4.66204	3.94754	4.46903
Dmpk	-2.49356	4E-05	8.94391	6.4763	4.79215	5.0611	5.22326	4.3895

Rpgrip1l	-2.49414	2.7E-07	2.20101	1.64708	1.31322	1.25673	1.10814	1.24665
Dyrk1b	-2.49438	2E-08	5.11762	4.70103	3.55955	3.73724	3.15324	2.97094
Pm20d2	-2.50113	0.02939	0.94625	0.46334	0.39798	0.33192	0.40133	0.53041
Ypel4	-2.50434	0.00793	0.62895	0.4433	0.35901	0.27522	0.38397	0.34351
Tmem147os	-2.50487	0.00432	2.12863	2.45503	1.10457	1.32878	0.99236	1.6382
Igsf11	-2.50492	0.00559	0.56921	0.48143	0.13925	0.32024	0.08936	0.28313
Bdh1	-2.50514	0.0028	0.79319	0.28461	0.24696	0.29126	0.21131	0.43322
Pcp4l1	-2.50653	1.6E-05	27.6272	11.7078	7.80218	10.8083	7.04351	14.5672
Fbxl19	-2.50668	7.8E-05	3.03654	3.96294	2.18093	2.44899	2.28348	2.02641
A230050P20Rik	-2.50814	0.00565	3.12745	4.58944	4.05856	2.91518	3.85775	2.93447
Enkur	-2.50834	0.00081	4.38842	2.1775	2.40472	1.94983	1.28597	3.40349
Slc23a2	-2.5088	2.5E-09	20.8155	14.8654	11.1209	11.9644	9.13105	13.4675
Prkcz	-2.51295	4.6E-08	3.5419	2.81227	1.88144	2.20729	1.46154	2.41613
Map7	-2.51376	0.00043	3.95056	3.40035	3.04384	2.4697	2.17829	2.97349
Hr	-2.51616	3.3E-06	1.78213	1.39565	0.61788	0.96779	0.78335	0.8652
Mks1	-2.5165	0.00127	1.06154	1.22258	1.16029	1.01265	0.77768	0.85117
Gpt	-2.51711	4.8E-06	3.55115	2.82682	1.43083	1.89845	1.45381	2.13919
Ubxn10	-2.51796	3.5E-09	4.42279	2.8944	1.94195	2.38868	1.34882	2.902
Zfp446	-2.52156	0.00012	0.67469	0.67831	0.52317	0.4782	0.48495	0.39631
Nek3	-2.52622	0.0012	0.97574	0.71762	0.58117	0.38555	0.55942	0.66036
Sytl2	-2.52956	3.4E-06	1.39258	1.28964	0.89551	0.89714	0.76192	0.96036
Rbm12b1	-2.53306	0.00919	2.54528	3.76114	2.50492	2.46388	2.42191	2.4927
Sec31b	-2.53346	0.0018	0.62433	0.50849	0.23758	0.35025	0.35574	0.22733
Ift172	-2.53497	1.8E-07	5.9493	4.71659	4.19166	4.374	3.05392	3.75445
Inafm1	-2.5353	7.9E-06	6.43062	6.69034	4.41773	5.10299	3.62708	4.05615
Gal3st1	-2.54049	0.00043	1.96906	1.514	0.81741	1.00322	0.78683	1.20987
Mok	-2.5416	0.00021	2.36056	1.42933	1.50053	1.05641	1.39526	1.36252
Sirt7	-2.54629	4.5E-05	4.09848	4.54687	3.74713	3.18193	3.22348	2.99432
Thap3	-2.54644	7.4E-05	3.9712	3.47459	2.73576	2.48905	2.70863	2.01937
1810041L15Rik	-2.55033	0.00052	0.82261	0.31839	0.30242	0.3379	0.30643	0.33125
Hdgfrp2	-2.55285	0.00159	9.57604	10.9477	9.91297	7.54746	8.20439	7.84524
Ccdc136	-2.55323	0.00155	0.42149	0.37432	0.19247	0.29368	0.13895	0.24172
Pnck	-2.55423	0.04497	0.69511	0.27436	0.26451	0.28077	0.27159	0.26575
Irx2	-2.55578	1.4E-10	5.56597	4.5193	2.66874	2.93392	2.38539	3.67077
1700001L19Rik	-2.55584	0.00388	1.31348	0.81183	0.86508	0.61217	0.48112	1.03469
Shh	-2.55707	0.00062	1.82868	1.49239	1.83125	0.73864	1.01063	1.49816
Rpgr	-2.55785	1.2E-05	1.26035	0.95938	0.61869	0.64144	0.74789	0.5679
Lhfpl4	-2.55841	0.00755	0.34568	0.23921	0.21525	0.1904	0.17266	0.19309
Adamts17	-2.55843	5E-05	0.45881	0.5771	0.17459	0.29224	0.27579	0.26986
Pcgf2	-2.55843	6.8E-05	2.5904	3.06728	2.21791	2.13449	1.63203	2.1222
Alas1	-2.559	8.7E-09	33.1732	25.9512	19.2907	20.2284	13.9951	23.4323
Ascc2	-2.55938	1E-07	10.3776	9.15069	8.31819	7.16832	6.34895	7.58238
Slc12a5	-2.55977	0.00136	0.26983	0.21784	0.15402	0.17092	0.09884	0.17082
Phyhd1	-2.56584	0.01267	2.40633	2.52951	3.13952	2.20776	2.16581	1.97141
Engase	-2.56719	5E-05	1.07477	0.90902	0.92525	0.86469	0.52921	0.7939
Lrrc46	-2.56749	0.00252	1.57243	1.21429	1.24876	0.7456	0.50085	1.68029
Ltbp4	-2.57195	2.9E-05	59.5963	41.4647	12.8101	29.224	19.4228	23.9202
Chkb	-2.5742	0.00055	3.16259	3.44672	2.99971	2.34515	2.70881	1.89489
Fam84a	-2.57716	0.00092	2.73013	2.62917	2.53041	1.91618	1.38619	2.76816
Grk3	-2.57718	5.2E-10	5.60025	5.62526	3.49051	3.9915	3.12514	3.44318
Wscd1	-2.57799	0.0002	2.00274	2.5228	0.58375	1.30378	0.93651	1.16947
Inha	-2.58252	0.04358	0.68116	0.52374	0.56554	0.33767	0.68047	0.15219
Dmd	-2.58316	2.9E-07	2.87816	1.334	0.80172	1.0556	1.00085	1.21985
Gm11992	-2.58454	0.00142	1.44618	0.5472	0.43446	0.43811	0.55761	0.68593
Gmpr	-2.58898	1.2E-05	2.53851	1.56898	0.89169	1.0648	0.96561	1.3598
Nthl1	-2.59179	0.00809	2.02972	1.00141	1.06202	0.56365	1.11522	1.10858

Pdlim3	-2.59267	9.1E-06	1.95204	0.8255	0.76983	0.92466	0.68902	0.77053
Tyro3	-2.59356	1.1E-05	1.72418	1.14669	1.24493	0.88394	0.96084	1.13487
Pacrg	-2.59467	0.00055	2.49842	1.47427	1.72459	1.76017	0.80875	1.42803
Thnsl1	-2.59485	2E-06	1.53622	1.71968	0.79951	1.08134	0.67857	1.05497
Trmu	-2.59514	6.7E-08	2.20644	1.6329	0.94458	1.04441	1.26283	0.90382
Wnk4	-2.59525	0.00019	1.2331	1.32229	1.18148	0.73377	0.64526	1.19062
Arhgef4	-2.59536	0.00137	0.72604	0.65447	0.69798	0.54408	0.5109	0.50089
Gm12473	-2.59549	0.02165	2.68176	2.40161	0.54027	1.43367	1.15567	1.29238
Xrra1	-2.59569	0.02327	0.33229	0.19322	0.23709	0.10066	0.213	0.20417
Lrig1	-2.59792	6.8E-06	3.48342	3.1537	2.8565	2.45735	1.86902	2.77343
Zscan18	-2.60049	0.0043	0.3001	0.32634	0.36707	0.25975	0.20415	0.2283
Dgkb	-2.60166	0.00339	0.54368	0.20901	0.16927	0.22958	0.20518	0.16197
Dnase1l2	-2.607	0.00606	0.78469	0.63049	0.22395	0.4992	0.25869	0.32143
Ankrd24	-2.61033	1.6E-05	0.61699	0.56928	0.26894	0.37383	0.27942	0.33086
U2af1l4_2	-2.61127	0.01043	2.51362	2.22153	0.8383	1.40317	1.59317	0.55532
Tmem212	-2.6118	1.2E-06	16.2009	11.8683	7.57279	6.80155	5.60726	12.4367
Tekt1	-2.61343	2.2E-07	4.73018	2.9529	2.57143	2.49063	2.02143	2.49122
Smim22	-2.61351	0.00101	3.25624	6.74748	2.60214	3.25529	2.26623	3.33462
Snrnp70	-2.61564	4.4E-05	25.6466	27.303	18.8973	15.5592	19.9605	15.9067
Cbx7	-2.61614	1.6E-12	6.25156	5.10883	3.0219	3.60856	2.93977	3.31061
Lrrc4b	-2.61654	0.04562	0.56131	0.211	0.2492	0.15115	0.13707	0.40876
Pcyt2	-2.61685	3.7E-06	3.36264	3.78615	2.66317	2.3939	2.44805	2.27276
Spice1	-2.62444	1.9E-06	1.82136	1.48342	1.30917	1.46115	0.70422	1.13293
Banp	-2.62475	0.00044	1.94189	2.10835	0.86474	1.46026	0.82734	0.74468
Gm14443	-2.63004	0.00526	0.37595	0.28618	0.27897	0.27334	0.15148	0.2464
A530020G20Rik	-2.63354	0.00016	2.49345	2.21129	1.38181	1.11472	1.48972	0.93214
Rfx2	-2.63419	2E-08	3.40507	2.78779	1.80368	1.93104	1.49672	2.05316
Enah	-2.63651	7.8E-09	5.31249	3.22633	2.75119	2.66212	2.15566	3.08697
Camkk1	-2.64118	5E-06	1.86203	1.47126	0.923	0.92035	0.82565	1.14411
Wnt3a	-2.64285	0.00091	1.12477	1.34302	0.33989	1.10239	0.38776	0.32523
Ppox	-2.64293	0.0002	3.15585	3.31934	2.74824	2.50041	2.5639	1.78993
Krtcap3	-2.64523	6.5E-05	3.68627	4.67668	3.35103	2.60569	2.60052	3.13185
Kcnmb4	-2.6461	0.01505	0.74191	0.32537	0.21958	0.24973	0.32209	0.31516
Fam166b	-2.6483	0.00111	1.7604	2.11884	1.7005	0.94353	1.19045	1.83051
Dennd1c	-2.64957	0.01987	1.66727	3.73997	3.02056	2.63523	2.23044	1.60348
Pgr	-2.651	0.00034	0.32063	0.27119	0.12201	0.19427	0.13311	0.1401
Hspa4l	-2.6559	1.2E-09	7.62179	6.42673	3.02416	4.60383	2.89398	3.84552
BC039771	-2.65677	1.8E-05	2.26745	1.40964	0.68585	0.70452	1.27778	0.77798
Hgfac	-2.66318	0.00599	0.66963	1.06476	0.36694	0.50309	0.66717	0.1975
Sh2d4b	-2.66395	0.00157	0.45363	0.67142	0.3884	0.32981	0.4154	0.27873
Wdr90	-2.6653	9.2E-05	1.90961	1.73374	1.68736	1.17661	1.27251	0.96342
Grhl2	-2.66626	1.5E-07	2.53808	2.51145	2.04528	1.86945	1.30035	1.82112
Rhov	-2.66657	0.02033	0.69723	0.45362	0.4286	0.35745	0.27504	0.39546
Odf2l	-2.66877	1.3E-05	1.47282	1.46608	1.40977	1.04337	1.10388	1.00242
Ptprz1	-2.66893	9.2E-07	1.82426	0.82056	0.64371	0.70337	0.71682	0.70651
Ccm2l	-2.67025	0.00042	9.25898	10.5912	8.49074	6.48352	7.06106	7.05699
1700020L24Rik	-2.67295	0.00088	2.69663	2.50149	1.09237	1.68655	1.52945	1.14025
Adrb1	-2.67359	5E-06	9.4601	9.02459	5.52543	6.53148	4.67399	5.28699
Nat9	-2.67441	7E-06	2.82943	2.95396	2.57398	2.22324	1.78133	1.99206
Gm17018	-2.67571	0.00021	4.99037	3.87227	4.44264	3.52289	2.28075	4.01338
Fsd1l	-2.67686	4E-08	2.70102	1.33261	0.95289	1.17055	0.9138	1.1602
Cntrob	-2.67962	0.00532	0.64457	0.74236	0.93938	0.53179	0.79372	0.40448
Wdr34	-2.68117	0.00019	2.86519	2.22684	2.07747	1.73597	1.24807	2.06184
Shroom1	-2.68222	0.00738	0.37726	0.35267	0.31735	0.25264	0.17456	0.21147
Efcc1	-2.68284	7.9E-06	1.28454	1.18174	0.3859	0.81924	0.46227	0.60927
2210408F21Rik	-2.68696	0.00044	2.10398	1.30846	0.98902	0.78736	0.77068	1.36882

Adam22	-2.69329	2.9E-09	1.18737	0.89056	0.65644	0.58367	0.53067	0.73416
Stk33	-2.69413	1.4E-06	2.85645	2.24129	1.37509	1.52216	1.2354	1.55071
Gas8	-2.6958	0.00103	3.98407	3.61997	3.63859	2.04145	2.3016	2.33141
Leng8	-2.69589	5.7E-06	27.1799	27.0713	16.7617	14.2871	19.9537	13.9829
Mns1	-2.69799	3.2E-06	2.63981	1.91581	1.32211	1.32081	1.04805	1.54876
Naaladl2	-2.70187	9.1E-07	0.851	0.39327	0.33051	0.3774	0.23138	0.41687
Zfp467	-2.70231	3.2E-11	3.75654	2.94119	2.08298	2.4581	1.43105	2.00482
Celf3	-2.70256	0.0002	0.61748	0.35288	0.30483	0.23256	0.26897	0.32814
Sstr4	-2.70503	0.00043	2.6298	2.22424	0.51257	1.12701	1.08076	1.20861
Sema4f	-2.70701	0.00011	1.57121	2.23431	0.80815	1.01474	0.86013	1.18821
Fhod3	-2.70952	0.03961	0.96269	0.24284	0.07712	0.27629	0.30933	0.19371
Il22ra1	-2.71253	0.00657	0.53331	0.54127	0.64941	0.43083	0.23442	0.5243
Ankrd63	-2.71346	0.03101	1.14618	1.2237	0.23596	0.38707	0.19271	1.09295
Casq1	-2.71411	0.03422	0.84268	0.27717	0.26723	0.31201	0.34297	0.19177
Slc22a17	-2.71495	0.00016	1.91116	1.88583	1.90907	1.20619	1.51678	1.23954
Cntnap1	-2.71543	0.0001	0.65297	0.47337	0.40821	0.35794	0.27335	0.36937
Lmod1	-2.71735	7.7E-06	4.44195	1.9176	1.79596	1.90634	1.49148	1.91432
Gria1	-2.71845	0.01442	1.63238	0.65197	0.22431	0.40294	0.31004	0.7925
Tnxb	-2.72072	5E-08	15.9873	11.4717	4.85103	6.74014	5.12441	8.65414
Klf15	-2.72639	0.00764	12.0431	6.034	2.98879	3.39058	3.71607	5.81794
Spata24	-2.72656	0.00361	2.45582	3.19552	4.42102	2.63251	2.1451	2.78577
Sorbs2	-2.72681	4.1E-05	1.3018	0.48494	0.5687	0.41857	0.42004	0.45433
Fam81a	-2.72848	0.00015	1.39177	0.9136	0.90114	0.71739	0.51741	0.95302
Rspo1	-2.72927	0.00567	0.98973	1.39516	0.53804	0.8281	0.65601	0.3475
Cep89	-2.73025	1.5E-05	1.9212	2.32348	1.84479	1.46862	1.40746	1.39743
Lca5	-2.73136	1.8E-07	1.88295	1.83758	1.35589	1.23738	1.09294	1.0917
B230369F24Rik	-2.7332	0.02148	0.74925	0.33194	0.12219	0.36749	0.18297	0.17538
Gm16136	-2.73654	0.00206	2.11502	1.52383	1.60968	1.13907	0.63126	1.79692
Fsip1	-2.73858	0.00373	1.43365	0.7823	1.10871	0.98071	0.74536	0.45466
Tctn2	-2.75009	3.9E-06	1.10905	1.06804	0.94017	0.7651	0.57115	0.82763
Tox3	-2.75384	0.00023	1.82349	0.80566	0.37284	0.84098	0.45858	0.51283
Klhl3	-2.75478	0.02623	0.93286	0.48732	0.90834	0.46546	0.7035	0.44956
Rnf43	-2.75814	9.9E-08	1.04457	1.08374	0.79499	0.81854	0.41834	0.73026
Vars2	-2.7626	9.4E-05	2.6431	3.1976	2.08161	1.87507	1.78926	2.11056
Dync2h1	-2.76371	7.7E-10	4.21266	3.23221	2.63421	2.28834	2.03425	2.52207
Bbs9	-2.76612	4.6E-08	3.24795	2.68519	1.93745	2.1097	1.72562	1.34244
Hsph1	-2.76852	0.00236	8.77424	10.9403	2.3822	6.72517	3.02979	3.69716
Stox1	-2.7705	0.00074	0.59568	0.62977	0.62336	0.37595	0.34548	0.52868
Crebzf	-2.77505	0.0003	10.3942	9.86468	10.567	8.24662	7.34219	7.17096
Gm16576	-2.77708	0.0006	0.55776	0.51463	0.15436	0.24577	0.28479	0.2354
4833427G06Rik	-2.78062	0.00295	4.13037	3.57279	2.89345	1.53564	1.51294	1.92263
Ppp1r36	-2.78124	0.0003	2.99154	1.56119	1.81658	1.65827	0.88596	1.61651
Tcea2	-2.78214	0.00066	1.18225	1.08564	0.77124	0.61398	0.66814	0.69184
Pifo	-2.78261	0.00046	5.26792	3.35877	2.5906	2.25877	1.18746	3.9174
Prodh	-2.78283	1.1E-08	2.69183	1.87493	1.47625	1.18323	1.17934	1.55668
1700010I14Rik	-2.78383	0.02264	0.38666	0.47238	0.34332	0.20824	0.25179	0.31677
Apob	-2.78546	0.01333	0.10488	0.09425	0.02993	0.04766	0.04322	0.0537
Gm12185	-2.78731	0.01736	0.21253	0.73283	0.59722	0.45564	0.26349	0.28126
Rph3al	-2.79016	3.6E-08	1.84399	2.16726	1.03889	1.08822	0.96493	1.21641
Sirt3	-2.79189	6.2E-05	1.56072	1.50003	1.49827	1.09605	0.93549	1.36581
Gm26615	-2.79264	0.00176	0.62775	0.50681	0.29317	0.25932	0.37627	0.25714
Clcn2	-2.79474	7.7E-06	0.53086	0.64141	0.38093	0.32164	0.35557	0.32307
Ciart	-2.79601	0.0284	5.69598	4.30505	1.56799	1.83569	1.71648	1.68787
Fads6	-2.7977	0.00011	0.45305	0.37157	0.3448	0.2329	0.28161	0.24744
Dennd6b	-2.7983	0.00027	1.03094	0.72104	0.679	0.68469	0.49383	0.38982
Gm5148	-2.80373	0.01706	0.35121	0.31685	0.21384	0.1986	0.12007	0.19182

Acacb	-2.80906	6.1E-06	2.40861	1.37148	0.90934	0.92501	1.02815	0.94002
Sgsm1	-2.81041	0.00597	0.64839	0.55875	0.51675	0.37802	0.33161	0.46104
Hcar2	-2.81363	0.00014	1.27778	0.92062	0.54027	0.65949	0.41604	0.62034
Rsph1	-2.81568	2.5E-06	13.8105	7.43316	7.70391	6.13302	3.96651	9.1929
Vsig2	-2.81731	2.7E-06	4.70765	4.9185	2.42369	2.18116	3.07686	2.3065
Panct2	-2.82581	0.00454	0.5764	0.33751	0.40494	0.25521	0.22738	0.34508
Abhd3	-2.82713	0.00013	1.3966	0.86623	0.74404	0.81797	0.47746	0.53395
Syne1	-2.8284	1.9E-13	18.4061	14.033	7.88982	8.6607	7.90943	8.67073
Ccdc9	-2.8287	0.00203	5.53963	6.18631	6.55129	3.86887	4.70228	4.57914
Kif21a	-2.83128	2.8E-11	7.50449	4.52176	3.68775	3.33321	2.70753	4.10259
Fam193b	-2.83379	2.3E-09	10.1907	10.5068	4.9035	5.82995	5.52212	4.6229
Rab11fip4	-2.8358	4.6E-07	2.30459	1.67961	1.66532	1.29233	0.94295	1.58676
Fam129c	-2.83588	0.04862	0.25657	0.7595	0.35148	0.32644	0.35712	0.18917
Nr4a1	-2.837	0.00235	20.1947	12.8024	3.83537	8.11979	7.546	4.96037
Sord	-2.83838	6.9E-09	10.99	9.0981	6.6943	5.83515	4.69417	6.61752
Fam83f	-2.83862	0.00238	0.45852	0.43952	0.27917	0.33337	0.22394	0.15026
Gpr179	-2.8391	0.00055	0.21059	0.22669	0.15299	0.08699	0.15428	0.13332
Tmod1	-2.84387	1.2E-10	6.52742	4.34614	2.61604	3.18351	3.20492	3.48924
5330417C22Rik	-2.8506	3.2E-07	9.72201	5.40419	3.44043	4.07324	2.48377	5.08039
4930481A15Rik	-2.85156	0.00067	1.5087	0.68975	0.5586	0.41999	0.7468	0.46768
Agbl5	-2.85231	6.9E-09	2.17563	2.09203	1.4784	1.43587	1.15744	1.13522
Cuedc1	-2.85246	3.6E-05	4.72572	4.10387	3.48233	2.66214	2.85994	2.65443
Pih1d2	-2.85253	6.1E-06	1.52608	1.23581	1.1491	0.88528	0.66604	0.95764
Arhgef28	-2.85516	1.3E-08	5.28267	4.45564	3.63176	3.18299	2.5337	3.14427
Hepacam	-2.85625	0.00705	0.35476	0.36929	0.18692	0.19841	0.21991	0.13414
Pir	-2.85655	4.3E-08	3.01004	1.57202	1.35198	1.30551	1.00014	1.41492
Acvr2b	-2.85751	2.2E-10	2.74385	2.4977	1.2211	1.39477	1.14134	1.48591
Magi2	-2.86035	0.00061	0.46667	0.45896	0.29735	0.37481	0.24647	0.33787
Nov	-2.86224	0.00017	3.85216	1.13091	0.9086	1.40809	0.76965	1.27801
Dhx16	-2.86227	6.4E-06	8.41091	10.0952	7.02954	5.48803	5.86758	5.66609
Acbd4	-2.86454	9.9E-08	3.48308	3.39308	2.13016	2.22339	1.70872	1.78346
Cep128	-2.86625	0.00016	1.56025	1.8928	1.7542	1.22702	1.42013	0.93609
Rasgrp2	-2.86694	7.1E-10	3.56234	3.71163	1.66713	2.19858	1.71282	2.10275
Mdm1	-2.86707	1.7E-06	2.2044	1.54553	1.49007	1.08957	0.89246	1.402
Phf21b	-2.86905	0.00423	0.5141	0.16305	0.26899	0.14276	0.17262	0.26323
Tlr12	-2.87106	0.0067	0.37375	0.77811	0.26257	0.31354	0.23168	0.3533
Ttc12	-2.87547	6.4E-08	2.85367	2.37959	1.97301	1.522	1.36918	1.79458
Phf1	-2.87756	4E-07	11.5065	10.9724	6.43916	6.26532	5.39934	6.71581
Platr21	-2.87889	0.02934	0.31644	0.30587	0.20643	0.08217	0.23182	0.20369
2810029C07Rik	-2.88329	0.04512	0.42156	0.27731	0.42778	0.1419	0.34315	0.19187
Meg3	-2.88462	0.00072	1.13105	0.70039	0.51879	0.4589	0.73612	0.2813
9330162012Rik	-2.8856	0.03174	0.09524	0.22152	0.32619	0.10099	0.10466	0.17556
Pbx4	-2.89065	0.00469	0.53558	0.55752	0.51736	0.3245	0.36218	0.30377
Ccdc96	-2.89713	7.2E-05	1.47771	0.96969	1.33793	0.87988	0.59727	1.04364
Gm281	-2.90099	3E-05	2.93939	1.2823	1.94273	1.42474	1.29203	1.21673
Homer2	-2.90308	5.9E-05	0.45593	0.30604	0.19828	0.17539	0.17496	0.24309
Mcf2l	-2.90309	1.4E-09	7.28696	6.38719	4.0777	3.07925	3.51872	4.58591
Erich2	-2.90619	0.00084	2.29273	2.25361	2.44055	1.44544	0.81712	1.77681
2410004P03Rik	-2.90716	8.8E-06	4.13175	3.0359	3.09542	2.28431	1.58912	2.96185
Slc9a2	-2.90784	0.00097	0.37927	0.12167	0.13437	0.11886	0.11497	0.13929
Saxo2	-2.91011	1.1E-06	2.76838	1.5825	1.37314	1.28401	0.90915	1.37645
Col8a2	-2.91141	5.8E-06	3.17413	2.32323	2.20668	1.61495	1.44589	1.95032
4930547M16Rik	-2.91725	0.0449	0.19029	0.32188	0.26068	0.06917	0.22304	0.18707
Mapk1ip1	-2.91815	1.9E-06	4.26856	4.88208	3.76551	3.11525	2.52302	2.66253
Fam198a	-2.91822	3.3E-05	0.89662	1.27722	0.5926	0.65762	0.29386	0.71523
Safb2	-2.92065	7.3E-05	10.1349	11.4955	9.29695	6.50658	7.12158	6.0578

2610028H24Rik	-2.92215	1.3E-05	8.62484	7.1135	7.46107	5.09581	3.13963	6.45153
Kif9	-2.92522	2.2E-09	2.29513	1.68954	1.35859	1.17169	0.80956	1.35799
Tlr11	-2.93081	0.04131	0.16739	0.40112	0.25479	0.25354	0.12263	0.13713
Gbp8	-2.93108	0.01754	0.61016	1.54818	1.84739	1.02868	1.10777	0.58681
Grtp1	-2.93288	1.9E-08	2.23156	2.45363	0.81522	1.02757	1.17707	1.27975
Ift81	-2.93697	1.7E-07	5.13692	4.36689	4.30849	2.84242	2.59205	3.71461
2810001G20Rik	-2.93701	0.00277	1.64183	1.90023	1.47973	0.99476	1.01288	1.1327
2810410L24Rik	-2.93932	0.01927	0.60329	0.43371	0.41323	0.34724	0.33147	0.1977
Ptpru	-2.9408	5.1E-09	5.14922	5.53893	3.50357	2.51297	2.98208	3.50317
Klhl17	-2.94091	1.1E-06	4.84871	4.22911	2.94064	2.60725	2.44209	2.43306
Zap70	-2.94345	0.0014	0.79371	2.14301	1.35915	1.18374	0.69329	1.0254
Col11a2	-2.94459	0.00236	0.36102	0.55414	0.29002	0.25114	0.30366	0.18622
Plin5	-2.94737	0.0001	1.92566	1.14749	0.84936	0.74246	0.70536	0.89637
Fam89a	-2.94835	0.00022	1.57008	1.50116	1.40276	0.78584	1.20024	0.72704
Sphkap	-2.94985	0.01121	0.17122	0.08045	0.07602	0.04611	0.06272	0.08572
Pcnt	-2.95055	3.4E-08	11.8557	13.6184	8.78594	7.36316	7.57602	7.80482
Fcamr	-2.95125	0.01401	0.66914	1.21274	0.87302	0.57917	0.49021	0.46988
Celsr1	-2.95257	1.3E-08	5.94314	4.69802	4.57662	3.5708	2.59781	3.92767
1700016K19Rik	-2.95379	2.2E-05	11.2065	5.11657	6.34007	4.80694	2.76081	6.82479
Ttll8	-2.95791	0.00556	0.45296	0.42817	0.21292	0.19372	0.37087	0.06549
Ovol2	-2.95879	0.004	0.6484	0.69464	0.49348	0.2619	0.38001	0.28331
Sfi1	-2.9608	2.4E-10	6.50081	5.87641	3.87621	3.79231	3.60282	2.62332
Syt5	-2.96155	7.7E-06	3.07017	1.39572	1.05148	1.20911	0.9559	1.03754
Mblac2	-2.96308	3.6E-07	1.20026	0.93554	0.45674	0.55611	0.60344	0.34706
Thbs3	-2.96696	6.3E-12	4.6966	5.38183	2.85379	2.46694	2.46954	2.24968
Ppara	-2.96811	0.00033	1.07148	0.48333	0.24464	0.36066	0.29654	0.37063
Osbpl10	-2.97011	1.2E-05	1.01308	1.02221	0.99422	0.5815	0.63801	0.72805
Adcy5	-2.97203	2.7E-10	5.05765	2.57984	2.09823	1.8007	1.74756	2.27466
Slc25a27	-2.97256	0.00012	1.05376	1.03501	0.42686	0.55608	0.57276	0.33418
Plch1	-2.97275	8.6E-06	1.14212	0.84076	0.70021	0.53821	0.48808	0.64112
Gm45151	-2.97457	0.01391	1.15455	1.085	1.17159	0.6218	0.56388	0.84077
Fhl2	-2.976	0.01276	4.04447	1.0588	0.9894	1.25441	1.12874	0.98615
Gm42742	-2.97718	0.00102	3.3431	3.99283	2.38242	1.66953	2.33039	2.30728
Ccdc65	-2.98656	1.1E-06	2.46467	1.92108	1.71027	1.37618	0.77889	1.58368
Ebf4	-2.98689	0.00059	0.53628	0.5252	0.48333	0.35913	0.26881	0.30061
Tchp	-2.9883	0.00052	1.9923	2.4674	2.38273	1.32206	1.54643	1.53505
Reep6	-2.98994	3.6E-06	4.41015	1.97291	1.33148	1.65622	0.96124	1.85131
Dnah8	-2.99876	0.00122	0.3069	0.55093	0.37896	0.34153	0.29366	0.15394
Gm9899	-2.99954	0.001	0.56814	0.2723	0.12972	0.19506	0.18036	0.15515
Ctxn1	-2.99961	1.6E-05	7.5361	4.52547	5.07592	4.01807	2.20836	4.6305
Lrrc9	-3.00204	0.00255	0.41353	0.15796	0.2132	0.17781	0.1075	0.17486
Armc4	-3.00394	2.9E-06	1.89137	1.17755	1.01962	0.98679	0.63507	0.8178
Dnaja4	-3.00738	3.7E-07	7.5839	7.64123	3.74785	4.34824	3.03897	4.96219
Cep164	-3.00744	1.1E-05	3.58366	4.15072	3.62208	2.43404	2.43306	2.60868
Clic6	-3.00988	9.7E-08	8.28432	5.25012	5.94086	4.15685	3.38499	4.98987
2310034G01Rik	-3.01182	0.04522	2.86054	1.85674	1.367	1.28979	1.02344	1.41701
Cxxc4	-3.01473	0.01243	0.22838	0.20389	0.11587	0.10762	0.07436	0.09355
Per2	-3.01565	3.2E-09	14.677	12.6246	8.38659	8.02314	6.12458	7.75635
Cacna1a	-3.01618	0.00026	0.44797	0.2774	0.21004	0.17933	0.24027	0.14418
Tjp3	-3.018	0.00017	2.32573	4.13849	3.10332	2.14113	1.74807	2.4745
Prom1	-3.02203	3.8E-08	4.39184	3.92988	3.3425	2.52982	1.8372	3.12874
Arhgap39	-3.02216	2.2E-09	2.60362	2.66349	2.06646	1.82538	1.38968	1.38139
Neat1	-3.02385	2.7E-07	275.199	259.169	112.706	104.637	165.434	90.8708
Gm10643	-3.02482	0.00392	0.81086	0.58784	0.59508	0.4211	0.42431	0.2847
Nckap5	-3.02563	4.3E-09	8.3789	8.31893	4.6362	3.4393	3.27378	6.35737
Hs6st2	-3.03243	4.1E-08	0.65517	0.4156	0.38733	0.34025	0.19713	0.3163

Rbpjl	-3.04254	3.1E-07	3.23474	4.5688	1.76454	1.73782	1.82109	2.10179
Mmp15	-3.04269	4.5E-12	12.9298	11.1075	6.53025	6.87006	3.68228	7.98619
Bcl7a	-3.04341	3.6E-14	2.53127	1.59551	1.24159	1.22459	1.02238	0.87524
Kank3	-3.0452	1.5E-06	27.8583	28.7402	13.8783	14.8023	14.2926	13.3914
Rtkn2	-3.04696	5.8E-11	17.6846	14.0103	5.05661	8.23064	4.83725	7.45996
Chrd	-3.04704	0.001	0.31291	0.23819	0.26791	0.10427	0.18339	0.19226
Ccdc157	-3.04721	1.1E-06	4.65758	4.4369	3.88056	2.5447	2.84941	2.79822
Gck	-3.04761	0.01401	0.69264	0.56035	0.75634	0.36492	0.44124	0.46876
Tppp3	-3.04861	5.1E-12	45.7182	25.876	20.9333	18.4167	12.4557	24.7405
5031434O11Rik	-3.04918	0.00052	0.68546	0.55905	0.36332	0.20765	0.46629	0.24067
Ccdc121	-3.05642	0.0005	2.3098	1.14667	1.66238	1.06481	0.88286	1.11071
Cfap161	-3.05679	4.9E-07	2.43529	1.33276	0.91581	1.0936	0.48275	1.12666
Echdc2	-3.05778	1.1E-07	4.79253	5.03302	3.69304	3.0005	2.48694	2.81386
Akap6	-3.0632	0.00052	1.84224	0.46669	0.48768	0.50686	0.46943	0.55413
Zdhhc23	-3.06325	0.00324	0.59182	0.94549	0.9759	0.67731	0.14452	0.70034
Rarb	-3.07027	5.8E-08	2.28796	1.32402	1.13413	1.09439	0.66163	0.98654
5031425E22Rik	-3.07079	3.9E-06	1.20309	0.96255	0.51969	0.59103	0.47642	0.45286
Efcab2	-3.0712	7.5E-06	2.13436	0.91191	0.80383	0.67992	0.69315	0.8112
Adamdec1	-3.07129	0.00011	1.54426	1.40168	1.33295	0.63898	0.71742	1.29601
Sec14l4	-3.07215	3.2E-12	8.82729	6.88996	4.21631	3.36451	3.14706	5.25755
Gfra1	-3.07403	4.5E-06	3.08573	1.60824	1.48524	1.2026	0.85536	1.62612
Nr1d1	-3.07503	0.00068	15.4654	7.83313	3.89705	3.7229	3.17496	7.39291
Nek2	-3.0803	0.00415	1.96186	2.03564	3.09397	1.61755	1.88175	1.14331
Ltk	-3.08144	0.01032	0.1779	0.4084	0.31914	0.15398	0.2048	0.18738
Ccdc40	-3.08209	3E-06	4.48283	2.72834	3.52701	2.41326	1.69752	2.79166
Irx5	-3.09347	1.3E-09	3.1254	2.44577	1.56724	1.30961	1.04853	1.81868
Cfap77	-3.09462	6E-07	2.15813	1.82531	1.5895	1.34975	0.61201	1.39734
Klhdc7a	-3.09537	9.5E-08	2.1059	1.99883	1.77637	1.26884	1.11422	1.31312
Foxp2	-3.09925	1.7E-07	0.70707	0.34969	0.31467	0.28681	0.21949	0.27982
Esr2	-3.10094	0.00112	0.65086	0.52755	0.21672	0.32862	0.29801	0.13331
Nat14	-3.10161	4.2E-05	1.53693	1.22563	0.6517	0.55872	0.38604	1.04329
Ace2	-3.1073	0.00035	1.94232	1.12941	0.80379	0.83848	0.40909	0.99454
St8sia6	-3.10919	1.4E-05	1.44205	1.17934	1.45577	1.20587	0.61989	0.80062
Gabbr1	-3.11165	1.1E-07	4.47041	4.7561	2.98149	2.35216	2.62861	2.25528
Ccdc85c	-3.11178	6E-07	3.18825	2.94625	2.56129	1.86016	1.38411	1.93481
Fermt1	-3.11336	6.5E-06	0.70519	0.7591	0.5123	0.31073	0.32875	0.53834
Rnf32	-3.11345	4.4E-05	1.00041	0.53588	0.34262	0.34347	0.36645	0.32784
Dlgap1	-3.12216	0.03521	0.08063	0.03182	0.02455	0.01303	0.03938	0.02202
Tnk1	-3.12235	1.7E-07	1.75596	1.67081	1.47214	1.04728	0.6633	1.34867
Lurap1	-3.1237	0.03713	1.86213	1.14185	0.72632	1.03421	0.93219	1.08061
Catsperg1	-3.12649	0.00695	0.4279	0.46062	0.19244	0.15713	0.24698	0.15935
Ppp1r9a	-3.12974	3E-14	3.90971	3.5141	2.10251	1.82422	1.57947	2.16283
Dock6	-3.12975	8E-08	14.1863	16.7927	9.01185	8.3369	7.90129	7.21705
Cep70	-3.13025	2.6E-14	2.47064	2.26644	1.25837	1.11693	1.22521	1.19888
Coro6	-3.13192	0.03082	1.91108	0.69273	0.51945	0.533	0.62224	0.49704
Foxc1	-3.13686	0.00015	1.38635	0.68399	0.7122	0.55998	0.54167	0.47324
Sptbn2	-3.13719	2E-08	1.08485	0.80519	0.72033	0.50974	0.57579	0.56228
Gm20605	-3.13802	5.3E-09	10.5988	9.36227	4.97532	4.77281	5.57611	4.64328
Slc23a1	-3.14056	1.2E-05	2.76439	1.45533	1.70673	1.16217	1.03325	1.52523
1700013F07Rik	-3.14144	0.00042	1.06938	0.90446	0.64631	0.60981	0.2765	0.56689
Eno3	-3.14358	6.6E-05	7.19566	2.44327	1.73886	2.16395	2.11629	1.67816
Dtx3	-3.14377	1E-05	9.94344	11.8341	9.72984	7.46615	6.50402	5.64417
Pih1h3b	-3.15179	0.01721	1.2711	0.25597	0.3455	0.29339	0.13303	0.59506
4930451G09Rik	-3.15538	0.03601	0.12421	0.42023	0.23634	0.15052	0.24266	0.03392
Gm20219	-3.15693	0.00215	1.29557	0.73051	1.47903	0.65414	0.58002	0.82554
4933408B17Rik	-3.16269	0.0005	0.75051	0.92042	0.29988	0.27283	0.41237	0.4304

Zgpat	-3.16615	2.2E-09	6.59372	6.49717	3.55977	3.7687	3.2838	2.87394
Efhc1	-3.16706	8E-06	3.9712	2.90394	3.91963	2.23064	1.66677	2.91453
E030030I06Rik	-3.16936	0.00271	0.65498	0.44318	0.52341	0.29763	0.23992	0.45611
Ccdc113	-3.17277	7.8E-11	7.37508	4.67829	3.40016	2.8787	1.87025	4.00869
Cdkl1	-3.1741	0.0042	1.83865	0.36591	0.43215	0.52424	0.51502	0.31013
Tmem132d	-3.17801	1E-06	0.606	0.64367	0.32178	0.25616	0.22714	0.41565
Tbx6	-3.17964	0.00283	0.86072	1.07056	0.4046	0.6442	0.37092	0.24888
D630039A03Rik	-3.18782	1.2E-06	1.48464	1.39217	1.25273	0.95818	0.61475	0.81968
Adrb3	-3.18986	1.9E-05	1.32569	0.61783	0.27797	0.44259	0.31712	0.35464
Eno2	-3.19226	0.00125	0.66004	0.73886	0.86431	0.54105	0.5262	0.31808
Ankrd45	-3.19301	8.8E-06	1.46727	1.05484	0.8096	0.57785	0.41205	0.90155
Shank1	-3.19368	0.00013	0.37355	0.55106	0.24793	0.28423	0.23548	0.13522
Gm26908	-3.19636	6.6E-06	2.45501	1.6045	1.14654	0.64231	1.14452	1.09706
Lgals4	-3.20324	1.8E-06	1.97273	1.73524	1.081	0.90839	1.12727	0.58182
Clmn	-3.20391	4.4E-10	1.90891	1.50474	1.45572	0.8975	0.92857	1.07596
Ccdc138	-3.20421	0.00039	1.01444	1.08044	1.32966	0.61463	0.74318	0.70797
Aox1	-3.20584	1.7E-08	5.38146	4.61377	2.85577	2.19704	2.70271	2.29912
Pitpnm3	-3.2068	1.2E-07	2.71733	2.62097	1.89102	1.37011	1.4044	1.44855
Grk4	-3.20788	0.00058	1.07812	1.43977	1.12282	0.85949	0.80368	0.75929
Ppef2	-3.20998	0.02266	0.36743	0.41436	0.20973	0.29683	0.13459	0.10034
Gm9987	-3.21142	0.00524	1.28871	1.7712	1.1034	0.87841	0.70808	0.65987
Bnipl	-3.21699	0.0073	0.8748	0.47084	0.63552	0.3132	0.17478	0.65154
Synm	-3.21857	5.5E-08	8.56614	5.34618	2.58937	3.96929	2.70757	3.65985
Tmem181b-ps	-3.22234	3.6E-05	4.55924	4.05908	3.11295	2.96062	2.70082	2.01951
Ccdc189	-3.22286	5.9E-05	1.83955	1.55586	1.61307	0.79149	0.95702	1.63811
Prpf40b	-3.22479	1.3E-07	4.37137	3.77523	3.17952	2.31328	2.04037	2.31196
Ifi203-ps	-3.22481	0.01898	1.13961	1.14235	0.43366	0.40917	0.89671	0.17289
C530008M17Rik	-3.22697	1E-05	0.30836	0.21399	0.11734	0.11018	0.08109	0.17489
Spata5l1	-3.23315	0.00058	2.95209	3.49555	3.63973	2.39675	1.64364	2.22504
Fam184a	-3.23317	5.5E-09	1.32191	1.59721	0.67071	0.7755	0.50727	0.72199
Cep131	-3.23562	2.1E-05	2.40676	2.25729	2.17629	1.57371	1.32674	1.17134
Clasrp	-3.23624	2.3E-07	3.72283	3.61558	2.21293	1.8667	2.04079	1.43032
Iqce	-3.23723	9.3E-09	2.17019	1.93178	1.8278	1.21373	1.13959	1.19358
Grik5	-3.24114	1.7E-05	2.40362	2.42159	1.99746	1.18861	1.52782	1.27418
Gigyf1	-3.24798	5.1E-05	8.47838	9.41615	6.93249	5.77022	6.0775	4.7847
Iqcc	-3.25294	0.00118	2.57854	2.18088	1.34101	1.59701	1.36429	1.07971
Plxnb1	-3.25361	2.8E-07	10.9601	10.9451	8.06604	5.88476	5.28079	6.42037
P4htm	-3.25683	4.9E-05	0.96268	0.73479	0.85776	0.52637	0.37843	0.57709
Nsun7	-3.25693	4.1E-08	0.67808	0.60219	0.44511	0.28759	0.36015	0.29165
Slc38a11	-3.25751	0.00745	0.42296	0.14309	0.169	0.16657	0.06197	0.15593
Carmn	-3.26145	2.7E-05	6.93753	6.20294	8.68929	5.35624	4.4725	4.02725
Hook2	-3.26179	3.6E-06	2.66344	2.67992	2.14939	1.50244	1.32885	1.56129
Gabrp	-3.26186	8E-06	12.6309	6.35949	4.8168	4.6376	2.49662	6.00493
Klhl32	-3.2637	0.0008	0.39682	0.29613	0.26647	0.16264	0.2052	0.16255
Kmt5c	-3.26512	1.7E-07	4.20048	4.86564	3.07524	2.66948	2.57552	1.60842
Lrguk	-3.2684	2.5E-09	2.17407	1.52485	1.08963	0.80319	0.77693	1.04261
Traf3ip1	-3.27168	1.7E-06	12.3768	11.7376	10.7586	6.75349	6.39931	7.8014
Trpm4	-3.27189	4.7E-10	2.55637	2.37621	1.69516	1.31883	1.10018	1.37548
Smim5	-3.27209	0.00017	3.15721	2.76401	1.5176	1.87934	1.17677	1.679
Peli3	-3.27274	5.9E-05	0.62725	0.38769	0.30295	0.27772	0.28278	0.11859
Grip2	-3.27372	0.00181	0.24985	0.30378	0.08914	0.07569	0.1144	0.16631
Proz	-3.27944	9.2E-05	0.62549	1.03021	0.33824	0.30916	0.39793	0.37758
Prom2	-3.28104	0.00422	0.31696	0.07447	0.14072	0.11736	0.0516	0.10098
Esrrg	-3.28239	3.7E-05	0.63265	0.34843	0.16796	0.16046	0.17246	0.27723
Zfp69	-3.28504	0.0001	0.95212	1.47173	0.93702	0.59676	0.5532	0.78003
Ino80dos	-3.28523	0.00013	1.48413	1.39871	0.96817	1.20751	0.90088	1.21588

Nphp4	-3.28796	2.4E-06	0.71027	0.71577	0.72459	0.47612	0.37642	0.3838
Cntfr	-3.28885	2.7E-06	1.67441	1.22181	1.01198	0.59676	0.64941	0.91452
Gm26583	-3.28916	0.01512	0.57024	0.25163	0.11322	0.18026	0.18163	0.10156
Rd3	-3.29375	0.00031	0.62018	0.32558	0.48828	0.20731	0.235	0.36792
Lncpint	-3.29465	1.3E-12	13.1562	10.5731	4.14324	5.65004	4.94834	3.75796
Pxmp2	-3.29568	0.0027	2.35955	0.70814	0.60825	0.87621	0.55761	0.99772
Sptb	-3.29704	1.1E-06	0.80457	0.37522	0.19744	0.19135	0.23687	0.27721
Gm35339	-3.29754	2.3E-06	0.55628	0.59191	0.32777	0.29355	0.24977	0.24992
mt-Nd1	-3.30126	0.01182	827.097	651.837	1020.05	495.225	415.674	724.676
Pabpn1	-3.30173	8.2E-05	20.1334	23.1628	19.7205	10.3751	12.0157	10.0347
Ccdc153	-3.30174	7.1E-06	17.2716	11.5726	11.1646	6.25155	5.48847	11.6507
Lrrc51	-3.30619	3.8E-06	6.49176	7.4698	6.98016	3.79604	2.98621	3.15395
Iqcg	-3.30788	1.6E-08	3.98382	2.65539	2.46975	1.96615	1.41094	2.03175
Wdr78	-3.30906	2.5E-11	3.35	2.59021	2.19759	1.75706	1.30951	1.53611
Shisa9	-3.31345	0.03631	0.44782	0.0909	0.0818	0.08682	0.02625	0.20545
Ccdc148	-3.31519	0.0002	0.34471	0.19159	0.21362	0.11338	0.06494	0.24206
Gm1673	-3.31537	0.00269	1.09223	2.07852	1.40276	0.99264	0.5001	1.00667
Mak	-3.31647	8.1E-08	2.43396	2.16695	1.75492	1.31946	0.78832	1.51127
Tsga10	-3.31667	4.3E-09	1.799	1.15928	1.10511	0.85641	0.72172	0.70885
Plekhg5	-3.32661	2.7E-08	5.15332	5.95206	3.79552	3.04397	2.82809	2.49682
Pcyt1b	-3.32696	8.4E-05	0.4433	0.51915	0.6034	0.30991	0.29978	0.29334
Lonrf2	-3.32883	0.01095	0.11859	0.11144	0.12034	0.07983	0.04826	0.05397
Murc	-3.33284	0.00448	0.75596	0.67699	0.30459	0.10777	0.39092	0.43717
Fam131a	-3.3376	1.7E-06	3.00937	2.22034	1.7543	1.37719	0.89124	1.59992
Slco1a5	-3.3382	0.0005	0.51534	0.39435	0.30816	0.23789	0.23371	0.16084
Gm45396	-3.34362	0.00013	10.5425	12.7043	13.42	8.98778	5.74128	7.87271
Aldh3a1	-3.34493	9.2E-05	4.51514	1.31272	1.71817	1.73828	0.62021	1.65687
Cux2	-3.3469	3.5E-07	0.60698	0.48273	0.43438	0.1976	0.27875	0.3711
Nrbp2	-3.35301	7.9E-07	18.4506	19.4445	13.9586	8.87212	11.6394	8.42763
Syt17	-3.35737	0.00209	0.44994	0.23256	0.11415	0.1666	0.07325	0.16383
4932411E22Rik	-3.35763	3E-08	1.06533	1.42665	1.03038	0.68133	0.61786	0.69095
Col4a6	-3.35844	5.2E-14	2.94416	1.67623	1.41243	1.16607	0.86443	1.2107
Agbl4	-3.35869	0.00402	0.14273	0.08048	0.13035	0.06918	0.06971	0.04677
Ankrd42	-3.36082	3.6E-05	2.5078	1.84835	2.39258	1.37834	1.29916	1.32077
Proser3	-3.36273	3.3E-05	0.96172	1.03325	0.80118	0.62994	0.47605	0.42589
Cdhr4	-3.3707	4.5E-06	8.41111	8.36643	10.0755	5.58064	3.44981	7.27908
Fanca	-3.37171	8.8E-06	0.86531	0.82886	1.01165	0.60008	0.58046	0.35873
Ushbp1	-3.37229	3.3E-07	6.50291	7.11419	2.61445	2.98926	2.76518	2.5827
Gstm6	-3.37716	0.00537	0.68882	0.23304	0.31454	0.25041	0.06728	0.30097
Grm4	-3.38156	0.00011	0.60113	0.40674	0.19607	0.18731	0.27681	0.14071
Arhgef19	-3.38256	2.3E-06	1.7656	1.43868	1.47484	0.9132	0.80447	0.91728
Akap14	-3.38306	0.00016	1.88588	1.4226	1.51287	0.80292	0.70946	1.1692
Rilp	-3.38526	7.1E-08	2.71186	3.02565	2.23956	1.67802	1.22583	1.41811
Dnali1	-3.38718	3.9E-10	6.50882	4.20122	4.30187	3.21713	1.97634	3.12226
Spata18	-3.38916	3E-10	15.5192	11.1826	9.45223	6.61872	5.24001	8.20384
Klhdc8b	-3.39203	1.9E-07	2.80326	3.11316	1.65577	1.24799	1.51792	1.23816
Ltf	-3.39824	0.0006	17.4733	5.86665	5.56115	6.84931	3.97601	3.81736
Cep126	-3.39897	4E-12	5.32275	3.54796	2.55257	2.09802	1.69603	2.26951
Asb2	-3.40003	4.2E-05	2.3724	0.80698	0.58877	0.73432	0.58563	0.42252
Rsph4a	-3.40085	4.3E-10	5.12992	4.16948	3.37097	2.77457	1.52159	2.74714
Mst1	-3.40383	0.00631	0.88834	0.75134	0.64536	0.36698	0.38456	0.46313
Stx1a	-3.405	0.00024	0.99354	1.44883	1.13424	0.66424	0.50198	0.8701
Stmnd1	-3.40614	0.00014	4.4228	1.64365	2.21854	1.13684	1.57097	1.647
Hcn4	-3.40852	0.00398	0.2538	0.12627	0.06817	0.05427	0.0875	0.08562
Wif1	-3.40892	0.00084	0.86558	0.47745	0.25778	0.18241	0.16542	0.49331
Tcte1	-3.41263	6.1E-08	3.06043	2.06072	2.13699	1.22417	1.02307	1.70398

Ccser1	-3.41297	2.8E-08	1.73359	1.3454	1.31576	0.81951	0.75364	0.85059
Drc7	-3.41535	2.3E-08	4.00443	3.95566	2.93133	2.00024	1.97067	1.97841
Nap1l3	-3.41826	0.00342	0.25756	0.57183	0.4043	0.33161	0.14152	0.18463
2010007H06Rik	-3.43123	8.9E-05	1.75863	1.58136	1.45819	1.02066	0.94932	0.68247
Rgl3	-3.4322	2.9E-09	2.35094	1.90964	1.43493	1.01541	0.98903	1.01069
Dalrd3	-3.43391	2.6E-08	8.88108	9.08289	7.76309	4.75049	5.19953	4.74911
4933413J09Rik	-3.43405	0.00893	0.16995	0.22359	0.10779	0.0572	0.11067	0.09282
Mib2	-3.44414	1.4E-11	5.80044	5.93615	3.73029	3.1278	2.60628	2.55027
Nhlrc1	-3.44685	0.0003	1.23483	1.21285	1.40969	0.89297	0.58363	0.68531
Gm37240	-3.44725	0.00022	1.97892	1.75498	0.96507	0.90797	0.68968	0.62961
Chadl	-3.45005	0.02195	0.4783	0.24895	0.10501	0.11146	0.22911	0.19593
Ptgfr	-3.45029	0.00041	1.80573	0.67245	0.27691	0.44089	0.25668	0.59616
KIAA1683	-3.45359	0.00713	0.43836	0.28649	0.04549	0.07243	0.17517	0.13059
Lca5l	-3.45886	8.8E-05	0.56027	0.59233	0.52767	0.20367	0.3694	0.34425
Muc6	-3.46099	3.7E-06	2.31247	2.40148	0.94681	0.67985	1.3224	0.77539
Mtcl1	-3.46702	3.3E-11	6.67194	7.10169	4.14026	3.28409	2.5847	3.76244
Tekt2	-3.46744	8.5E-05	2.21133	1.82965	2.14031	0.93204	0.88044	1.26028
Arhgef38	-3.46767	1.1E-15	2.49812	2.04684	1.19677	1.24329	0.66178	1.07812
Grb7	-3.47195	5E-15	2.79577	2.98447	1.39442	1.43078	0.89483	1.37872
Adamts20	-3.47496	0.0158	0.18274	0.13738	0.27815	0.09226	0.20081	0.04159
Fank1	-3.47529	6.6E-08	2.14538	1.61291	1.40601	0.91471	1.01869	0.71607
mt-Co3	-3.47777	0.03226	1291.66	1171.88	1762.77	874.779	579.176	999.405
Sult5a1	-3.47913	4.6E-07	1.38484	0.83912	0.77867	0.5385	0.87825	0.49107
Amy1	-3.47927	0.00014	0.82167	0.40352	0.33285	0.27301	0.31068	0.152
Eps8l1	-3.48289	2.8E-06	3.91922	5.02981	3.23041	1.96202	2.30583	2.18189
Kcnma1	-3.48479	6.8E-05	0.53921	0.21134	0.15014	0.1514	0.14452	0.14007
Gm13307	-3.49104	0.00912	1.53693	0.46425	0.87728	0.39909	0.24127	0.62957
Cfap206	-3.49351	7.8E-12	5.84649	3.76307	3.73597	2.33924	1.61626	3.22331
Megf6	-3.49413	6.9E-11	9.10245	8.14002	3.82563	3.64623	3.10909	3.89487
Rap1gap	-3.4962	1.4E-09	3.1966	4.12154	2.83831	1.95398	1.80059	1.80409
1810064F22Rik	-3.49621	3.8E-05	0.7299	1.06632	0.53026	0.38193	0.34028	0.43489
mt-Atp6	-3.4963	0.04304	1592.31	1489.9	2307.14	1083.07	696.94	1215.17
Osbpl6	-3.49688	1.8E-15	6.20838	4.51396	3.24326	2.73317	1.94829	2.64723
Kcnd3	-3.51383	5.8E-06	0.46683	0.21757	0.23928	0.1674	0.13959	0.1483
Dnaic2	-3.51591	1.1E-06	2.4594	2.01247	2.12286	1.19935	0.80565	1.70364
Ccdc85b	-3.51612	4.1E-07	15.8978	15.7294	10.1018	7.2696	6.81219	7.74092
BC051019	-3.52094	4.2E-09	4.98606	4.14249	4.38242	2.29914	1.93952	3.21728
Elmod1	-3.52769	1.9E-12	2.30523	1.40285	1.12327	0.93681	0.65904	0.8061
Dock3	-3.52809	0.04612	0.08218	0.02139	0.02887	0.05362	0.01853	0.01036
Ccdc159	-3.53171	0.00022	0.96233	0.99086	0.42989	0.40561	0.42913	0.34278
D830026I12Rik	-3.53177	1.9E-05	0.86494	0.84128	0.74056	0.52405	0.34851	0.31887
Ppp1r32	-3.53584	0.00022	1.69144	0.74179	1.35882	0.45547	0.64251	0.87249
1700030J22Rik	-3.54146	2.8E-07	1.98162	1.31503	1.25293	0.81273	0.82637	0.67436
Slc44a4	-3.54601	1E-09	3.05241	2.90439	2.41245	1.61823	0.90307	1.99576
4930579K19Rik	-3.5527	0.03347	0.72261	0.97787	1.31989	0.52538	0.52938	0.4736
RP23-163N19.1	-3.5542	4.5E-06	0.59023	0.46355	0.3369	0.21712	0.20076	0.27631
Klk11	-3.55518	0.02036	0.99618	0.42128	0.42647	0.41495	0.22806	0.40807
Myo5c	-3.55567	3E-09	9.28729	9.28104	8.84969	4.85658	4.64398	5.911
Hap1	-3.55643	0.00054	1.45519	2.52309	2.65801	1.32251	1.45695	1.09283
Faah	-3.55649	1.6E-10	2.05201	2.13986	1.46411	1.05255	0.81143	1.12711
Spag16	-3.56598	6.7E-12	2.63318	1.59894	1.52615	1.0636	0.78151	1.13947
Map3k19	-3.56944	3.9E-07	2.63744	1.59788	1.98592	1.57531	0.8222	0.95011
Spag17	-3.57049	4.8E-08	2.7652	1.86554	2.21096	1.27773	1.06412	1.48089
Maats1	-3.57758	5.2E-11	2.15552	1.85718	1.77082	1.24496	0.78218	0.95723
Wnt10b	-3.57889	0.00016	1.29141	0.99295	0.13403	0.56905	0.31536	0.22442
Neurl2	-3.57907	0.00121	3.0626	2.97122	1.74814	0.92779	1.64974	1.10694

Morn3	-3.58147	0.00027	1.62018	1.99838	2.46614	1.02254	0.69238	1.65918
mt-Co1	-3.5817	0.00958	2025.27	1788.54	2818.16	1428.66	1075.25	1527.87
Gm21988	-3.5824	0.00036	2.86105	2.87639	2.64993	1.66805	1.34459	2.74196
Katnal2	-3.58689	9.4E-05	0.42734	0.32339	0.46217	0.27254	0.16477	0.16584
Trp63	-3.58979	0.00318	0.26124	0.10127	0.06213	0.08574	0.04785	0.06242
Ttc39a	-3.59059	1.6E-08	3.5574	2.8355	3.01928	1.96353	1.21437	1.98364
Trpv3	-3.59454	0.04554	0.12523	0.11915	0.03574	0.03794	0.08027	0.01282
Lrrc48	-3.59459	2E-08	3.98174	2.56423	3.39188	2.0206	1.4511	1.98705
Ccdc24	-3.59827	0.00015	1.74906	1.82335	1.81099	0.99811	0.68537	0.88308
Col19a1	-3.60027	0.0382	0.06631	0.04487	0.0757	0.02411	0.04858	0.01087
1700024G13Rik	-3.60117	0.00129	3.44237	2.9115	2.61989	1.2514	0.84062	2.44416
Dpp6	-3.60174	0.0009	0.27319	0.10013	0.14554	0.11587	0.08006	0.05222
Wdr60	-3.60445	1.1E-05	3.75158	4.35513	4.17086	2.4513	1.99393	2.4106
Rbm24	-3.60804	4.1E-06	2.67052	1.3456	0.71352	0.80901	0.56195	0.86117
Spag6l	-3.60816	4.9E-11	3.3057	2.50784	2.26429	1.44449	1.10079	1.67417
Lmntd1	-3.61413	0.00042	0.51863	0.33895	0.12111	0.19282	0.13816	0.13519
Kctd8	-3.6151	0.0002	0.70405	0.32064	0.49462	0.22969	0.09919	0.44369
Pgam2	-3.61708	0.04248	6.85032	0.73573	0.24827	0.98821	0.63727	1.60348
Zfp692	-3.6196	1.6E-05	2.5126	3.38444	3.24022	1.6633	1.97706	1.29607
Rac3	-3.62108	2.2E-09	5.72184	2.98731	2.25801	1.62638	1.55251	2.14128
9530026P05Rik	-3.62464	8.7E-12	1.30255	0.90015	0.43522	0.42347	0.36075	0.48151
Wdr54	-3.62606	1.2E-05	0.61244	0.57686	0.4926	0.24457	0.40788	0.30789
Klhl41	-3.63431	0.01622	0.9029	0.18328	0.12369	0.2407	0.18521	0.11835
Catsper2	-3.63604	0.01278	0.24719	0.45996	0.45151	0.16475	0.23542	0.22277
Pcsk1	-3.64166	0.00193	0.33818	0.16685	0.25738	0.18782	0.06194	0.09235
Ugt8a	-3.64208	0.00078	0.38259	0.20437	0.16091	0.1708	0.08851	0.08248
Kctd14	-3.6452	0.00018	1.316	1.2033	1.13692	0.56029	0.33874	1.049
Ngef	-3.64723	4.9E-12	3.32137	2.43328	1.92255	1.20458	1.25088	1.26471
Rec8	-3.65103	0.0054	0.86418	1.96784	2.27667	0.96663	0.92529	1.14367
Akap8l	-3.65243	1E-06	11.3127	12.5837	11.0553	6.74746	6.1915	5.87813
Map4k2	-3.67395	8.7E-17	12.0941	11.6587	6.69936	5.53732	4.64144	4.92029
Drc1	-3.67476	1.7E-09	4.97029	3.45517	2.75933	2.22762	1.34675	2.00808
Appl2	-3.67541	2E-08	10.2714	9.02504	9.18444	5.58143	4.92655	4.79237
Lnx1	-3.67731	1.1E-05	0.8826	0.40351	0.43571	0.31796	0.34951	0.2736
Gsg1l	-3.67828	0.00053	0.30267	0.27569	0.18606	0.15517	0.05117	0.1526
Zfp579	-3.6787	3.8E-06	3.73344	4.17928	2.7077	1.82293	1.88239	1.67326
Ccdc81	-3.67995	2.7E-06	1.76782	1.4952	1.23332	0.81324	0.45568	1.01918
Hpse2	-3.68043	0.00215	0.77013	0.43424	0.29306	0.21775	0.26329	0.21031
Gm42984	-3.68329	0.00794	1.01795	0.70442	0.52822	0.50462	0.40677	0.07581
Tbc1d30	-3.68337	1.8E-21	4.25311	3.29857	1.97879	1.69199	1.17577	1.77507
Pkn3	-3.68646	4.2E-06	5.0445	4.52073	1.98496	1.92486	2.39032	0.98482
Ankrd13d	-3.68973	0.00015	1.18813	1.44454	1.10206	0.76486	0.68001	0.42586
Arhgdig	-3.69241	9.3E-09	2.75249	3.2307	2.46891	1.54856	1.0288	1.67974
Cracr2b	-3.69432	1.9E-07	10.8719	10.9002	8.11084	4.64502	3.94605	5.5499
Cdc14a	-3.69673	1.5E-09	3.94843	2.92378	2.9805	1.58672	1.50533	2.0927
Cyp2f2	-3.70198	2.4E-05	505.492	179.834	116.321	130.493	61.5272	166.139
Rgs9	-3.72368	1.4E-11	3.07317	2.23655	1.19121	0.8747	0.82725	1.39353
Baiap3	-3.7261	5E-08	1.39365	1.13339	0.48954	0.40054	0.34033	0.70262
Jhy	-3.72664	0.00067	0.65573	0.69326	0.62382	0.38074	0.22018	0.40291
Vipr1	-3.72688	9.9E-06	0.70865	0.42622	0.34158	0.26716	0.23074	0.19352
Acsl1	-3.73091	1.3E-14	8.78525	3.67438	2.78836	2.31631	2.14298	2.54676
AA415398	-3.73174	0.0003	0.4964	0.58779	0.3778	0.30076	0.29093	0.18978
Agbl2	-3.75614	4.9E-12	1.53832	1.11521	1.01919	0.68239	0.42261	0.70891
Synpo2l	-3.76248	0.0021	0.82873	0.16689	0.0901	0.17933	0.14455	0.12932
Pnma1	-3.76706	1.2E-05	1.81978	0.98243	0.79563	0.60993	0.39711	0.53925
Cdhr3	-3.77368	3.7E-15	13.7331	9.6532	7.87421	5.15356	3.73881	6.61373

A830018L16Rik	-3.77949	7.7E-08	0.41775	0.24396	0.20439	0.15066	0.12387	0.12223
AU022754	-3.78151	0.00946	0.21484	0.23362	0.45549	0.20455	0.11242	0.12572
Slc6a17	-3.78802	0.00013	0.48853	0.15025	0.09126	0.09149	0.08459	0.14554
Tmprss6	-3.78925	0.02283	0.20036	0.16946	0.13071	0.06937	0.08388	0.07035
Efcab5	-3.78971	6.9E-05	0.45987	0.28423	0.22211	0.10716	0.15549	0.18838
Chrm3	-3.79109	3.4E-05	0.68903	0.39423	0.43957	0.30697	0.11877	0.18263
4430402I18Rik	-3.79328	1.1E-08	1.04482	0.86944	0.73105	0.46967	0.2963	0.56605
Tef	-3.79419	0	24.1206	16.1772	9.33339	7.99314	6.72111	8.27733
Gpr155	-3.79644	4.1E-12	6.81032	2.63713	1.92026	1.69027	1.49275	1.96063
Vit	-3.80364	0.00347	0.53728	0.25561	0.11501	0.14242	0.0492	0.22009
Rassf10	-3.80495	2.7E-06	0.91442	0.88389	0.62634	0.33242	0.26796	0.57791
Adam28	-3.80743	2.5E-12	4.31868	2.81297	2.40844	1.44365	1.23644	1.93172
Itga10	-3.80764	5E-06	0.63404	0.84908	0.28149	0.35214	0.27096	0.15872
Lpar3	-3.81972	5.2E-10	4.26292	2.25736	1.94663	1.25772	0.869	1.88287
Galnt5	-3.82166	7.2E-06	0.53222	0.36358	0.42064	0.18604	0.22494	0.20124
Fmo3	-3.82391	2.6E-06	14.1568	5.60259	4.62372	3.27957	3.85452	3.7523
4930562C15Rik	-3.83535	1.2E-06	4.9302	4.66387	5.68718	3.02876	2.44145	2.75841
BC021891	-3.83613	1.3E-05	0.33692	0.32567	0.40295	0.15553	0.1528	0.2103
Dzank1	-3.8422	2.9E-08	1.81304	1.13138	1.02227	0.56934	0.42924	0.48002
Nrn1	-3.8424	1.3E-05	1.91364	1.0936	0.35426	0.62673	0.26523	0.59321
Cfap43	-3.84304	5.5E-08	7.25746	6.55728	7.55196	4.29711	3.52883	3.47272
Gm29811	-3.84513	0.02604	0.81991	0.64723	0.312	0.16559	0.36039	0.31347
Atp6v1c2	-3.84875	2.3E-08	6.35392	3.80661	3.07273	1.79119	1.26069	3.28958
Lypd6b	-3.84901	1.7E-06	0.8769	1.09766	0.76081	0.42504	0.33405	0.54599
mt-Cytb	-3.85015	0.00302	1318.09	1198.75	1728.77	836.677	559.054	999.594
Sox6	-3.85297	2E-10	0.73273	0.3337	0.30564	0.20917	0.18065	0.23665
Bmpr1b	-3.85445	5.2E-10	1.77712	0.91997	0.47221	0.43626	0.41526	0.55224
Lekr1	-3.85659	1.2E-06	0.50932	0.60308	0.40701	0.30859	0.17412	0.25036
Rhbg	-3.88378	0.00712	0.80162	0.79764	0.32299	0.19999	0.27636	0.50221
Ccdc171	-3.88838	1.2E-10	1.87273	1.73845	1.18194	0.89481	0.72502	0.62368
Lrrc56	-3.89456	1.1E-06	1.32461	0.99302	1.11695	0.5472	0.57342	0.56726
6820408C15Rik	-3.89557	1.1E-05	3.98676	2.85317	3.55935	1.4555	1.79734	1.92621
Klkb1_1	-3.90131	0.00017	0.33062	0.52983	0.17879	0.10543	0.25496	0.07128
Kif19a	-3.90407	8.2E-06	2.73923	1.93699	3.10149	1.30596	1.11851	1.71069
Ccdc191	-3.91278	3.1E-09	2.56954	2.86726	2.7362	1.12511	1.07567	1.45058
Tex9	-3.91662	0.00041	0.68519	0.64098	0.90073	0.38998	0.50196	0.29768
Drp2	-3.92524	7.6E-05	0.13883	0.27096	0.08534	0.09058	0.09388	0.035
Izumo4	-3.92541	3.4E-05	2.337	3.95318	2.77909	1.59294	1.21271	1.5955
Syne4	-3.92588	0.00013	0.86811	1.33735	1.55735	0.82653	0.59055	0.5334
Espn	-3.9279	2.3E-11	0.77292	0.69108	0.40337	0.36795	0.24267	0.22615
Lrrc10b	-3.93308	1E-06	5.76082	4.72363	5.1709	2.55784	1.93298	3.4226
Cpne7	-3.93536	0.01389	0.17338	0.17597	0.09897	0.10505	0.05081	0.07102
Gm8066	-3.94033	0.00137	3.7665	2.54851	3.0099	1.02693	1.10372	1.23429
Eno4	-3.94911	8.2E-13	3.87385	2.82555	2.43435	1.65804	0.98938	1.6014
Cfap54	-3.9524	6.8E-09	3.71701	2.61626	3.06385	1.49629	1.30153	1.9355
Bves	-3.96353	0.00782	0.63401	0.1173	0.11309	0.10804	0.07257	0.17855
A730020E08Rik	-3.9741	0.00022	0.53935	0.21817	0.21416	0.12787	0.10308	0.19212
Firre	-3.97534	2E-09	8.73837	9.00193	6.86546	3.43567	4.61667	3.65661
Cfap69	-3.98594	4.4E-13	3.53565	2.50176	2.20283	1.40012	1.21045	1.23058
C230062I16Rik	-3.98726	0.00233	0.43347	0.83799	0.919	0.26263	0.31755	0.35512
Myo15b	-3.98749	0.0004	0.30579	0.18809	0.26446	0.17404	0.13577	0.03796
Ly6i	-3.98872	0.00092	1.10616	0.93557	0.5412	0.3351	0.28942	0.51785
Wdr66	-3.99057	4E-11	3.61447	3.0061	2.81963	1.66436	1.26218	1.678
Scn4b	-3.99482	0.00812	0.40334	0.18607	0.02093	0.08886	0.09402	0.06008
Ccdc88c	-3.99976	5E-09	8.49282	9.53257	8.58881	4.68982	3.78395	3.92339
Gm867	-4.00543	1.6E-05	6.28367	5.63096	8.96683	4.30572	3.12372	3.55453

Snhg20	-4.00904	0.0004	17.5566	18.6752	11.6814	7.57007	9.46886	7.58878
Cryaa	-4.01057	0.02492	0.46208	0.26055	0.61543	0	0.16926	0.44165
Cyp2s1	-4.01753	1.1E-14	19.8287	15.441	11.7828	8.18106	5.35252	8.51637
Fabp4	-4.01926	0.03191	24.6693	1.98302	0.81462	3.0264	3.88336	2.42192
Sh3bgr	-4.03008	8.7E-06	2.07316	0.65128	0.81145	0.53833	0.26036	0.67938
Sned1	-4.031	3E-14	6.99636	5.82786	4.64723	3.05532	2.72137	3.0433
Irx3os	-4.03226	3.1E-05	0.613	0.70512	0.39189	0.2377	0.10778	0.44194
Espnl	-4.03365	0.00623	0.1342	0.12769	0.09575	0.07114	0.03687	0.04123
Cacnb1	-4.04127	7.3E-11	1.67388	1.41574	1.17418	0.81867	0.59098	0.51219
Erich3	-4.04443	5.2E-06	3.41382	3.17178	3.25739	1.68557	1.22434	2.01202
Mtus2	-4.05144	4.3E-10	1.34632	0.63174	0.63163	0.38551	0.35804	0.40795
Fam161b	-4.0538	9.4E-09	1.43205	1.29417	1.45568	0.73691	0.56049	0.70715
mt-Atp8	-4.05942	0.00103	723.537	675.953	860.24	419.389	257.485	511.698
Guca1b	-4.06259	0.04594	0.22451	0.28483	0.12815	0.04534	0.13706	0.09197
Acaa1b	-4.0667	0.00303	1.07073	0.39374	0.15944	0.22565	0.23873	0.07628
Ccdc57	-4.0812	8.1E-07	1.78136	1.73368	1.56003	0.76881	0.94748	0.61974
Gbp4	-4.08294	0.0004	1.99885	4.35018	4.49422	2.2449	3.06262	1.64755
Rwdd3	-4.08566	0.00024	0.68526	0.45756	0.67936	0.20759	0.30385	0.2807
Ankrd66	-4.08982	1.9E-06	1.79771	1.92983	1.89441	0.83785	0.70915	1.01962
Foxj1	-4.09359	7E-09	29.7726	26.6832	27.2704	14.1145	10.102	17.1168
Avil	-4.09647	0.00016	0.77951	0.43196	0.15343	0.228	0.39384	0.24224
Iqca	-4.11299	3.1E-11	2.75422	1.89608	1.29182	0.78909	0.57873	1.25939
Olig3	-4.11331	0.03214	0.22041	0.11185	0.30194	0.08013	0.12918	0.03611
Gm16201	-4.11572	0.00896	0.31596	0.33404	0.51099	0.23929	0.28933	0.21571
Ccno	-4.11618	0.00011	0.92506	0.61768	0.44465	0.20649	0.24968	0.39887
Egfl8	-4.11658	0.00082	0.88174	0.70189	0.35527	0.40853	0.34198	0.29745
Kcnh3	-4.11801	7E-05	1.22361	0.51746	0.87305	0.37068	0.41085	0.3968
Iqub	-4.1199	9E-08	2.17649	1.22723	1.57078	0.65177	0.62313	1.02477
Ano9	-4.1223	0.00492	0.10356	0.47297	0.26009	0.11294	0.18208	0.08484
Nek5	-4.12325	2E-08	4.06716	2.74661	3.31135	1.5855	1.43204	1.7306
Ucp3	-4.12942	0.00024	0.61183	0.39427	0.13304	0.22948	0.17075	0.04774
Slc7a3	-4.1299	0.00885	0.21724	0.20415	0.02756	0.10237	0.01768	0.05933
Cfap70	-4.15171	4.6E-09	2.37076	1.79031	1.64321	0.94905	0.63579	1.05784
Rapgefl1	-4.15582	0.0001	0.37005	0.36741	0.47755	0.16572	0.22395	0.17135
Mgst2	-4.16394	0.00017	0.9373	0.66758	0.95739	0.50812	0.34333	0.34353
Gm7173	-4.16653	8.5E-12	1.41105	0.80086	0.75244	0.48933	0.36724	0.4335
Fam92b	-4.17602	0.00014	2.55352	1.77208	2.91512	0.47604	0.8634	1.28738
Ttll9	-4.17616	2.8E-06	0.99422	0.53109	0.65711	0.44386	0.19167	0.30009
Vwc2	-4.17628	0.00155	0.22679	0.12788	0.06904	0.03664	0.02215	0.12387
Zcwpw2	-4.19276	0.00928	0.21919	0.23835	0.14298	0.07589	0.09176	0.10261
Pde6h	-4.20113	0.0079	0.24447	0.07877	0.19935	0.04937	0.13645	0.03815
Pdzd7	-4.20249	1E-06	0.44675	0.69903	0.35701	0.37895	0.11455	0.1464
Zkscan7	-4.20634	2.6E-06	1.32227	1.27812	0.71882	0.80114	0.7565	0.64997
Tekt4	-4.20853	1.7E-08	3.39833	2.31957	2.72249	1.37266	0.9609	1.41647
Acsm1	-4.20863	0.0001	1.34012	1.09803	1.86455	0.73583	0.39884	0.65188
Rbm20	-4.21279	0.0417	1.31827	0.1479	0.23035	0.1793	0.26608	0.17633
9330158H04Rik	-4.21778	0.00152	0.39799	0.22721	0.15902	0.10248	0.17494	0.05706
Trpm5	-4.22421	0.03559	0.12848	0.07245	0.0326	0.02595	0.03138	0.03509
Hdac11	-4.23112	5.6E-13	2.06453	1.32466	1.02944	0.60386	0.53892	0.73876
Gfra4	-4.2315	0.00324	0.42188	0.28546	0.19265	0.12781	0.06181	0.17282
Rnf186	-4.23167	9.2E-09	3.94894	3.4018	4.09071	1.77229	1.55363	1.85724
B3gat1	-4.23288	0.02839	0.16573	0.09345	0.04204	0.02231	0.02698	0.07543
Zcchc12	-4.23525	0.03766	0.2003	0.23718	0.1372	0.04854	0.0587	0.13128
Dydc2	-4.23562	0.00502	1.08001	1.30493	1.40907	0.28044	1.01726	0.2528
Dpf3	-4.23935	0.00077	0.21618	0.31084	0.17276	0.07859	0.06335	0.1594
1810020O05Rik	-4.24413	0.00881	0.61066	1.47568	0.49795	0.31713	0.31955	0.28588

Slc28a3	-4.24476	4.9E-05	0.30141	0.16668	0.19851	0.09833	0.1104	0.07598
Cbr2	-4.24942	5.5E-08	779.777	404.014	285.155	199.507	85.4695	307.379
Fhdc1	-4.24996	3.7E-10	5.92893	4.44783	3.29278	2.03001	1.43005	2.54204
Ccdc146	-4.25075	1.5E-07	2.821	2.49199	2.76721	1.25341	1.13283	1.36955
Ttc6	-4.25696	1.9E-09	1.18922	0.80928	0.67101	0.30643	0.41057	0.40315
A930001A20Rik	-4.26001	0.02434	0.53492	0.45243	0.07633	0.20256	0	0.21912
Armc3	-4.27075	1.9E-08	1.32327	1.0919	1.17904	0.62575	0.35466	0.58171
Atp7b	-4.27173	1.9E-14	9.29523	5.42722	4.27986	2.62024	2.44817	2.94484
Gm10044	-4.28166	4E-07	1.179	0.76255	0.63339	0.33616	0.33025	0.25568
Dqx1	-4.28483	2.3E-06	1.01516	1.03936	1.09792	0.46939	0.45013	0.4815
Ak7	-4.28602	5.8E-14	8.00274	5.14512	5.12424	2.84482	1.77397	3.50798
Gpr156	-4.30449	0.01916	0.11963	0.21923	0.04552	0.0604	0.05843	0.03267
1700040L02Rik	-4.30598	2.1E-06	2.38838	0.97942	1.32198	0.65776	0.58324	0.65223
Efnb3	-4.30616	0.00148	0.25826	0.41259	0.13104	0.08693	0.16818	0.07053
Ppp2r2b	-4.30992	4.2E-05	0.61885	0.32956	0.20933	0.1111	0.15112	0.20655
Slc6a15	-4.31535	1E-06	0.97781	0.44532	0.22898	0.31901	0.18368	0.12324
1700007G11Rik	-4.31607	6.1E-05	1.70312	2.18738	2.52037	0.99367	0.50832	1.49865
mt-Nd4l	-4.31684	0.00265	676.267	586.541	854.886	347.318	229.578	420.503
Ttc29	-4.32061	2.2E-08	2.86317	2.02892	2.32631	1.17212	0.69918	1.39472
BC049715	-4.32669	0.0038	0.50146	0.36354	1.02227	0.41234	0.23616	0.23476
4930412C18Rik	-4.33151	2.9E-06	0.31199	0.22618	0.3392	0.14402	0.08707	0.13388
Sgcg	-4.34289	0.00611	1.65361	0.20568	0.27761	0.26521	0.3563	0.31876
Ankmy1	-4.34621	0.00332	0.32042	0.33477	0.62399	0.23981	0.15189	0.1853
Cys1	-4.34672	3.7E-12	3.65903	3.73705	1.57629	1.75682	0.70808	1.18776
Fam47e	-4.35786	7E-07	2.98687	2.48963	3.85459	1.54742	1.07822	1.80866
Kcng2	-4.36029	0.03814	0.73339	0.18092	0.06977	0.09257	0.11193	0.15021
Cfap58	-4.36104	2.5E-09	2.62135	0.96998	1.05986	0.59559	0.54011	0.73822
Zfr2	-4.36688	9.3E-11	0.68354	0.5678	0.52951	0.29582	0.25038	0.19
BC030500	-4.36876	0.03508	0.20653	0.17468	0.35366	0.09385	0.11348	0.0423
Myom1	-4.37126	1E-06	7.81007	2.81931	2.18764	1.66839	1.84035	1.76782
Slc4a3	-4.37426	9.9E-06	1.75261	1.5003	1.28536	0.65643	0.77815	0.50472
Cfap57	-4.376	4.8E-13	2.68105	1.97934	1.50441	0.9361	0.59922	1.04239
Zdhhc1	-4.37701	4.1E-12	9.87199	9.24677	7.26613	3.92193	4.01258	3.44082
Morn1	-4.38217	4E-10	2.2623	1.43059	1.13443	0.51241	0.77446	0.67554
Gm20878	-4.38497	0.00853	1.94085	1.64154	0.67142	0.35634	1.33568	0.81912
mt-Nd5	-4.39267	0.00138	755.939	633.854	966.055	374.758	312.079	467.886
March4	-4.39441	6.1E-10	2.06628	1.57795	1.92374	0.88729	0.60256	1.00254
4933439C10Rik	-4.39602	0.0003	1.10594	1.16923	0.83551	0.73905	0.84595	0.4397
Cfap99	-4.39754	5.4E-07	1.59185	1.12803	1.81726	0.5474	0.59885	0.5992
Mycbpap	-4.40139	1.5E-09	2.85504	2.49457	2.18186	1.12938	0.82972	1.31448
Gabre	-4.41038	4E-10	1.65858	1.10919	0.9247	0.45571	0.63579	0.395
Acss1	-4.41242	1.1E-14	8.5629	6.41893	4.8751	3.12359	2.20451	3.136
Spata17	-4.41822	3.1E-09	1.55942	1.4266	1.34427	0.63631	0.53624	0.65182
mt-Nd4	-4.42182	0.00111	1161.41	939.915	1316.26	529.504	414.154	677.859
Asb11	-4.42429	0.00975	0.3529	0.13567	0.18313	0.05831	0.07051	0.13142
2010300C02Rik	-4.42473	1.7E-12	5.1293	4.23172	4.21195	1.78832	1.8327	2.28542
Sult1d1	-4.42967	2.8E-06	6.50024	1.5708	2.03539	1.17026	1.14289	1.61282
Dnah11	-4.42995	3.3E-11	3.97367	2.96547	3.02184	1.64971	1.1848	1.54233
Cdr1	-4.43749	9.8E-05	0.67131	0.26719	0.31556	0.1914	0.14465	0.12941
Grip1	-4.44279	1.7E-08	0.5113	0.37348	0.35818	0.11969	0.20432	0.1904
Nek11	-4.44542	1.6E-06	1.3122	0.96428	1.05598	0.45617	0.40974	0.56395
Calml3	-4.44542	0.00138	3.8431	1.35434	1.82804	1.12542	0.46924	1.31187
Fndc5	-4.45024	0.01316	3.90077	0.83878	0.37738	0.50072	0.31483	0.97497
mt-Nd6	-4.45246	0.00132	675.617	569.49	826.738	285.336	292.021	413.794
Ccl28	-4.4532	0.00037	0.34236	0.22752	0.19542	0.11853	0.10749	0.0601
Acrbp	-4.4603	1.6E-05	1.03128	1.29657	0.98639	0.38841	0.46964	0.27401

Dnaaf1	-4.46311	3.2E-09	2.26644	1.56092	1.62635	0.66698	0.71159	0.84882
Cyp4f15	-4.46405	1.1E-09	1.96124	1.15099	0.95955	0.72752	0.55712	0.26233
Frem2	-4.4657	4.5E-06	0.33076	0.1865	0.236	0.09603	0.06563	0.15807
Ubap1l	-4.46804	0.02158	0.27069	0.13082	0.13244	0.04686	0.11332	0.03168
Syt10	-4.47125	2.5E-07	1.33665	1.17238	0.7347	0.29994	0.47148	0.52725
Alpk3	-4.4755	0.00117	0.73694	0.35959	0.21032	0.08586	0.19726	0.20899
Shank2	-4.47976	2.5E-11	2.62104	2.10204	2.31435	1.06897	0.94454	1.09797
Gm16194	-4.48181	1.7E-05	1.86912	1.79165	1.28028	0.90597	0.18257	0.81669
Plekhd1	-4.48646	6.3E-09	2.713	3.05948	2.25008	0.78676	1.05322	1.59574
1700019D03Rik	-4.50554	0.01368	0.40079	0.23193	0.04816	0.25561	0.10817	0.06913
C430049B03Rik	-4.50973	0.01033	0.45385	0.52781	0.58288	0.20624	0.12468	0.32534
Lrrc6	-4.51313	2.7E-08	2.93648	2.18707	2.40165	1.06219	0.70638	1.43626
Gm31105	-4.51777	0.02211	0.79771	0.67469	0.75889	0.32221	0	0.32676
Mfsd4a	-4.52062	0.00425	5.88414	4.579	1.61043	5.79809	5.68861	6.33032
Capn13	-4.53558	0.0055	0.17471	0.11821	0.35901	0.08468	0.10239	0.05725
mt-Co2	-4.53691	0.01657	1925.97	1798.59	2727.52	1282.61	930.134	1488.48
Gm13421	-4.54949	0.01546	0.34124	0.67343	0.51941	0.20675	0.16666	0.18637
Vtcn1	-4.55978	1.4E-05	1.67208	0.70711	1.19304	0.48544	0.45936	0.5137
Gm45193	-4.56085	0.01947	0.76507	1.49327	0.67185	0	0.6036	0.3375
Plp1	-4.56313	3.4E-07	0.62526	0.32008	0.28176	0.1296	0.24109	0.08088
Atp10b	-4.58358	0.00086	0.29776	0.16527	0.26557	0.14658	0.05453	0.06861
Hydin	-4.58373	9.3E-16	5.65131	3.20365	2.94252	1.57702	1.25391	1.94571
2810030D12Rik	-4.58391	0.02379	0.75878	0.8022	0.10828	0.11493	0.13897	0.38852
Ccdc151	-4.59471	1.2E-08	1.85291	1.81299	2.19824	0.72642	0.79849	0.98224
Cerkl	-4.60322	0.00455	0.22471	0.33626	0.31573	0.14662	0.12663	0.09913
Nwd1	-4.60868	0.00016	1.27383	1.27327	1.83319	0.72502	0.67304	0.61983
Gm26704	-4.61361	0.02814	0.23506	0.11361	0.23001	0.04069	0.123	0.02751
Cyp1a1	-4.62862	0.00169	0.85209	1.16418	0.14965	0.17871	0.31212	0.32219
Ddo	-4.63004	2.4E-12	2.21052	1.6792	1.60058	0.79367	0.75723	0.6875
Trim66	-4.63552	3.1E-05	0.11727	0.12971	0.09269	0.04373	0.03965	0.04434
Ttc41	-4.63916	1.6E-05	0.82569	0.74824	0.76307	0.26205	0.51848	0.14495
6430531B16Rik	-4.64268	0.00435	0.68847	1.24224	0.73357	0.3059	0.23537	0.48883
Dsc2	-4.64707	1.3E-05	0.51926	0.19031	0.25687	0.09438	0.11412	0.15598
Kcnk2	-4.65951	1.8E-09	4.58259	3.2351	1.35007	1.28749	0.71746	1.43814
Gm4258	-4.66369	0.00037	2.70764	2.59541	1.23642	0.87494	0.92568	0.73942
Lrrc45	-4.66611	4.1E-08	9.65244	10.2752	9.24606	3.94925	4.36877	3.95391
Hs6st3	-4.67764	0.0001	0.90497	0.9841	0.95933	0.31332	0.28413	0.52957
Atp2c2	-4.68178	2.8E-06	2.80646	2.39763	2.78316	1.28817	0.91378	1.20767
Aim1l	-4.68337	8.3E-07	0.88154	0.80123	1.00637	0.43845	0.38556	0.45272
E230025N22Rik	-4.68867	0.01694	0.18123	0.22992	0.31033	0.0549	0.09957	0.11135
Adgb	-4.68926	4.4E-12	3.59922	2.23321	2.94695	1.29591	1.09145	1.28099
Diras2	-4.69468	1.1E-16	2.14269	1.88332	1.00722	0.80184	0.41552	0.6884
Six4	-4.69477	2.3E-16	1.91005	0.99634	0.88374	0.49452	0.35136	0.61352
Rab36	-4.69715	2.5E-08	1.3904	1.47623	1.58729	0.61837	0.7477	0.49684
Nkx6-2	-4.69842	0.00016	0.76634	0.68866	0.21871	0.17412	0.17544	0.27467
4732471J01Rik	-4.69913	0.00211	0.6747	0.67765	0.74617	0.29382	0.4016	0.15546
Bpifb5	-4.70277	0.01451	0.27898	0.47191	0.44588	0.20283	0.04088	0.22856
mt-Nd2	-4.71015	0.00046	855.3	687.154	976.616	353.471	261.4	528.488
Gm13111	-4.71721	4.9E-09	0.90255	0.95965	0.47102	0.28123	0.2267	0.38027
Zmynd10	-4.72	3.9E-11	4.16146	2.7577	3.13451	1.45563	1.25718	1.15987
Gm14317	-4.72047	0.01657	0.24956	0.68598	0.14245	0.1512	0.18282	0.05111
Cacna2d3	-4.74092	0.01526	0.24719	0.12544	0.08466	0.02995	0.01811	0.12151
Hpcal4	-4.74968	5.1E-05	0.30794	0.23151	0.05859	0.11402	0.02507	0.07008
Gm28042	-4.75728	0.00268	0.59859	0.49139	0.60296	0.35201	0.21926	0.12981
Endog	-4.76407	0.00024	7.56134	6.90397	9.22062	4.37305	3.33622	3.5901
Rhpn1	-4.76882	4.6E-05	0.74681	0.38447	0.88963	0.37378	0.1903	0.23941

C230034O21Rik	-4.77464	8.5E-07	1.32047	1.17131	0.77211	0.72199	0.23594	0.44855
Upp2	-4.78344	2.3E-08	0.38851	0.3505	0.44352	0.18831	0.12333	0.14853
Efhb	-4.78438	1.3E-07	1.57893	1.88702	1.52822	0.6239	0.52806	0.84362
Tsix	-4.78637	0.01259	0.43587	0.17797	0.29169	0.09106	0.14314	0.08619
Cracr2a	-4.78638	1.2E-08	3.5489	4.18644	4.19805	1.83193	1.55652	1.69283
Necab2	-4.79007	0.04377	0.30504	0.07371	0.0995	0.07921	0.03192	0.0357
Rn18s-rs5	-4.8034	0.00239	3926.49	5207.57	4574.95	3844.57	3965.5	3654.36
Plin4	-4.80405	5.6E-06	8.96459	2.25992	1.66055	1.12078	1.63316	1.47663
Spag8	-4.80455	3.7E-06	2.36313	1.76629	3.19967	1.03221	0.80521	1.30568
Tnfrsf25	-4.81492	8.5E-07	1.04294	1.18744	0.36635	0.29165	0.35264	0.29577
Gm32742	-4.83053	0.0047	0.13943	0.127	0.31835	0.09748	0.07857	0.07908
Gm44276	-4.83662	0.01371	0.13853	0.05207	0.07029	0.0373	0.05638	0
Dnah7a	-4.83821	1.2E-06	1.47376	0.80477	1.30676	0.63719	0.47694	0.35753
Kcnip2	-4.83847	0.00032	0.3416	0.33336	0.06	0.0796	0.05775	0.12916
Lhb	-4.85345	0.00018	1.07773	1.82305	1.07655	0.32649	0.29608	0.88294
Gm42902	-4.85346	0.02942	0.96581	0.70017	0.94506	0.25078	0.20216	0.11303
Actc1	-4.85703	0.0006	81.4587	13.0822	7.34669	12.8944	6.07927	9.52704
Colq	-4.86686	7.2E-08	1.03274	0.79222	0.46611	0.422	0.15835	0.25579
Got1l1	-4.86796	0.02763	0.45117	0.1272	0.17169	0.12149	0.03673	0.08214
Fam13a	-4.86871	2.2E-15	5.91789	3.45484	2.90009	1.53916	1.08914	1.91566
Dnah7c	-4.86981	2.2E-07	1.56541	1.36691	1.53061	0.67621	0.49908	0.67686
Itpka	-4.87023	6.1E-12	2.21059	2.12464	1.43388	0.48704	0.51528	1.19364
Myo18b	-4.88776	0.01765	1.11845	0.32495	0.17544	0.12932	0.20641	0.18886
Itgb1bp2	-4.89599	0.0007	0.91388	0.5153	0.17388	0.12305	0.22317	0.20797
Col20a1	-4.90024	7.1E-07	0.67528	1.01332	0.5471	0.30356	0.39896	0.10708
Dnah7b	-4.90617	1.9E-08	1.61829	1.10581	1.28382	0.57057	0.59278	0.47564
Nptx2	-4.91241	0.0035	0.4322	0.2437	0.08223	0.04364	0.07916	0.14753
Cfap61	-4.91485	6.1E-11	1.30039	0.95417	1.06179	0.51134	0.33438	0.46565
Pla2g4b	-4.93337	0.03591	0.59524	0.39745	0.39341	0.3037	0.30984	0.24383
Snx32	-4.95022	9.5E-06	2.21424	2.06005	2.52779	1.14034	0.93273	0.58956
Vwa3b	-4.97127	6.3E-08	1.67888	0.77453	1.37454	0.44181	0.53421	0.51405
Gm973	-4.98325	1E-08	2.29843	1.83128	1.99645	0.7972	0.59788	0.92786
Ccdc170	-4.98411	1.4E-08	3.80573	4.42079	4.48213	1.82423	1.53519	1.81547
Vpreb3	-4.98641	3.5E-11	9.67604	6.11539	4.61271	2.89906	1.63583	2.78757
Ccdc67	-5.00241	4E-08	0.82758	0.74662	0.91328	0.35099	0.28293	0.2938
Ccdc173	-5.0029	1.1E-06	1.14261	1.4798	1.34518	0.56248	0.39238	0.61431
Zbbx	-5.00453	9.7E-10	2.61911	2.16427	2.88688	1.11263	0.92629	1.0112
Lrrc26	-5.00731	3E-06	5.52631	1.68785	3.59255	0.93008	0.9559	2.26373
Spef2	-5.01479	5.3E-13	3.29759	2.22812	2.55526	1.08269	1.0122	1.02629
Crocc	-5.01731	1.1E-05	1.73482	2.22428	2.30845	0.83686	0.98759	0.71516
Unc79	-5.01988	7.6E-11	1.20947	0.40764	0.4464	0.25345	0.22651	0.23095
Casc1	-5.03069	2E-10	1.6848	1.16727	1.14584	0.48325	0.4924	0.52128
Kif27	-5.031	9.6E-10	3.1524	3.09907	2.59393	1.42628	0.98975	1.27454
Wdr63	-5.0318	3.6E-13	4.24826	3.26646	3.40118	1.68811	0.97005	1.53681
Lrrc31	-5.04573	0.00148	0.14627	0.19441	0.09542	0.07596	0.04593	0.03424
Slc2a4	-5.05107	5.1E-05	2.52111	0.42202	0.4497	0.30232	0.32706	0.45181
Dnah9	-5.05891	2.1E-10	5.74976	4.28181	5.0668	2.20679	1.63623	2.13475
Lrrc74b	-5.06934	2E-12	3.59232	2.19543	2.32831	0.98295	0.86592	1.13924
Trpm3	-5.07829	7.1E-06	0.48722	0.24917	0.19403	0.09611	0.14111	0.07426
Efcab6	-5.08173	6.3E-09	1.5394	1.07294	1.12277	0.56998	0.40724	0.40871
Ulk4	-5.08198	6.7E-09	2.96474	1.99875	2.26864	0.85909	0.78694	1.12644
Sptbn5	-5.09102	5.6E-05	0.54135	0.6305	0.4863	0.17206	0.26656	0.17449
D130043K22Rik	-5.09117	3.1E-11	1.21183	0.61497	0.47432	0.33565	0.15219	0.29784
1700029J07Rik	-5.09437	5.8E-08	2.56679	2.28372	2.51165	0.86847	0.83031	1.14701
Stpg1	-5.10366	3.5E-06	0.53834	0.65768	0.17071	0.19932	0.13146	0.09801
Adcy1	-5.1038	5.8E-08	0.43765	0.15423	0.19152	0.07955	0.13359	0.04781

Rp1	-5.10553	1.7E-11	2.27027	1.49345	1.54997	0.69225	0.65223	0.62606
Dixdc1	-5.10837	3.5E-20	2.05191	1.89042	1.06666	0.66601	0.47647	0.63039
Rsph10b	-5.10902	9.9E-08	4.5065	3.61222	4.57302	1.62397	1.6615	1.54436
Cmya5	-5.12127	1.6E-05	4.4397	0.70407	0.48396	0.52304	0.63808	0.46729
Esyt3	-5.13618	1.8E-11	1.43483	1.26342	0.96485	0.41681	0.48957	0.45087
BC048546	-5.16388	0.00041	8.77472	4.11517	5.2289	2.1957	1.63473	2.92773
H2-Q5	-5.16392	0.00038	5.10939	14.6152	12.0591	6.53922	5.08894	2.82196
Tle2	-5.1668	1.1E-13	3.2888	4.47044	2.43038	1.24539	1.33375	1.07053
Cadm2	-5.16889	0.00041	0.2421	0.09451	0.09567	0.02821	0.06139	0.0534
Mfsd4b1	-5.1694	5.2E-06	0.97061	0.82092	1.41372	0.36501	0.41683	0.4113
Wdr31	-5.20007	1.2E-09	1.34944	0.94567	0.96833	0.45552	0.3107	0.50539
Gm26660	-5.21506	0.00252	0.39509	0.57719	0.12301	0.10881	0.15788	0.08828
Defb23	-5.2244	0.0268	0.43912	0.3714	1.16971	0.17737	0.42893	0
Tmem191c	-5.22485	3.1E-05	0.78578	1.20261	1.06792	0.24938	0.52083	0.39852
Trp73	-5.22707	1.1E-16	1.68349	1.49366	1.43199	0.56	0.5562	0.54087
Catip	-5.23483	8.8E-07	4.17236	3.77349	4.05579	1.47673	1.51319	1.52298
Gm1661	-5.23609	0.00025	0.77876	0.50184	1.60874	0.31456	0.43468	0.42534
Gm10069	-5.2446	1.9E-05	0.29261	0.45962	0.14316	0.15196	0.06125	0.08562
Syngap1	-5.25673	9.9E-09	4.07769	4.01796	4.76266	1.74491	1.67604	1.58765
Tas1r1	-5.25948	4.2E-05	0.38283	0.48569	0.61915	0.11598	0.21035	0.20909
1700003E16Rik	-5.27403	1.7E-06	1.97398	1.70434	2.86383	0.897	0.93395	0.70752
Srl	-5.27461	0.00697	3.85933	0.26113	0.2154	0.45727	0.30158	0.42157
Dnah5	-5.27464	6.3E-13	10.5304	6.7169	7.61856	3.33526	2.6243	3.24594
Kif6	-5.28842	5.9E-11	1.62479	1.15589	1.33481	0.51522	0.44498	0.53494
Corin	-5.29596	0.00152	1.77601	0.41487	0.15448	0.16397	0.19826	0.30486
Uox	-5.31552	0.00298	0.19899	0.1683	0.19472	0.08612	0.02083	0.06987
4921536K21Rik	-5.32531	1.9E-05	0.42319	0.53688	0.32207	0.17093	0.10334	0.14446
Ankub1	-5.327	0.00029	0.64763	0.67556	0.86256	0.34007	0.25304	0.22992
Dnaic1	-5.32737	1.6E-07	2.65936	1.41619	2.32381	0.93493	0.48104	0.96831
Lrrc2	-5.32785	0.00454	0.77796	0.10281	0.02775	0.10311	0.03562	0.1195
Adgrb1	-5.33113	0.01917	0.0664	0.04493	0.0758	0.01609	0	0.04352
Prob1	-5.34977	5.1E-10	6.25984	4.21977	5.2477	1.8907	1.71116	2.23507
1700095J03Rik	-5.35513	0.00303	1.21245	0.34182	0.80741	0.42852	0.25907	0.12416
D630024D03Rik	-5.36271	5.5E-07	1.22405	0.73949	0.86505	0.28253	0.42702	0.19101
Slc27a2	-5.36644	6.4E-06	0.75425	0.2881	0.38887	0.11793	0.21389	0.13953
Cnksr2	-5.37279	3.5E-12	0.52238	0.33345	0.27005	0.13138	0.09387	0.14535
mt-Nd3	-5.37928	0.0052	566.923	456.851	446.45	201.8	195.548	335.87
Trim55	-5.38388	0.01725	0.68701	0.11621	0.15686	0.14569	0.05033	0.08443
Apol7a	-5.40164	0.04637	0.24139	0.07656	0.34447	0.09141	0.02211	0.1236
Caps2	-5.41099	5.5E-06	0.50713	0.31605	0.51801	0.16172	0.15643	0.10934
Myo1h	-5.43447	2.2E-09	1.38694	1.26429	1.17871	0.45751	0.53061	0.42925
Txndc2	-5.43708	0.00269	0.28859	0.36612	0.32945	0.08742	0.14094	0.07881
Rsph14	-5.43875	2.3E-10	1.06136	0.76944	0.86547	0.38584	0.22216	0.27328
Nek10	-5.44066	2.6E-08	1.15031	0.77022	1.14905	0.37752	0.39795	0.32722
Myb	-5.44733	1.3E-12	3.52954	2.42094	2.99744	1.14748	0.88293	0.93448
Dcdc2a	-5.4492	1.2E-12	1.21284	0.63413	0.44055	0.22045	0.16155	0.36132
Ttc21a	-5.46201	9.2E-09	9.40838	8.58831	11.7857	3.31752	2.91845	3.74978
Crb1	-5.48176	0.00013	0.10861	0.05653	0.08584	0.01519	0.03672	0.02738
Fbxl13	-5.50319	8.4E-07	1.07179	0.55275	1.04451	0.34845	0.19151	0.40691
Tmem88b	-5.50358	1.4E-05	0.3684	0.46738	0.1502	0.17538	0.03856	0.08623
Ubxn11	-5.50385	1.8E-05	2.42694	2.42735	3.69414	1.12033	1.26997	0.88368
2010001K21Rik	-5.5165	0.00142	2.8158	2.08386	2.20999	0.85302	0.25785	1.44179
Dnah6	-5.52474	1E-13	12.1489	10.0712	10.1855	4.43237	3.24792	3.90893
Sema6c	-5.53137	6.3E-09	0.55403	0.54668	0.40408	0.22378	0.14657	0.11347
Mgat3	-5.54666	5.3E-13	7.16604	4.75268	5.78913	2.25393	1.90771	2.10131
H2-M5	-5.55716	0.00033	0.20365	0.3732	0.38748	0.10282	0.09946	0.11123

RP23-478P10.3	-5.56714	0.00047	2.38084	2.37979	1.23544	0.65569	0.63425	0.53196
Als2cr12	-5.56894	0.00379	0.18827	0.09099	0.12282	0.06518	0.0197	0.04407
Erbb4	-5.56894	0.02404	0.23657	0.01876	0.03376	0.0224	0.02166	0.03634
Gm44246	-5.57777	2.4E-06	2.56339	3.8797	1.5402	0.40871	1.08722	1.1053
Kcnj11	-5.58189	0.0292	0.79489	0.2311	0.11343	0.10535	0.09099	0.1221
Panx2	-5.58207	0.01961	0.22841	0.06439	0.02897	0.06151	0.01859	0
Fsd2	-5.60402	4.5E-09	2.63584	0.83358	0.86348	0.45827	0.30224	0.58211
Adra2a	-5.6043	0.00228	0.64881	0.44713	1.53623	0.48046	0.17604	0.45279
Pclo	-5.61279	2.7E-07	0.52874	0.28208	0.29716	0.15771	0.12812	0.08997
Pisd-ps1	-5.61538	2.7E-07	20.638	25.7213	21.0086	8.94509	8.91162	5.49398
Prr36	-5.61815	1.1E-05	0.65361	0.64758	0.46902	0.27155	0.24626	0.22949
Slc4a5	-5.62547	2.3E-14	4.61094	2.94153	1.86841	1.16324	0.73784	1.07009
Dnah1	-5.63744	1.4E-08	2.42678	2.12454	2.44639	0.88134	0.57182	1.10453
Gm26671	-5.65075	9.9E-08	1.3624	1.34434	0.90727	0.34394	0.45746	0.32555
Fendrr	-5.65304	4.3E-08	42.9468	40.0909	18.2679	14.1431	14.9457	14.1909
Ccdc162	-5.67694	3.4E-08	3.64524	3.01474	3.78219	1.38717	1.19053	1.1769
Kifc2	-5.69906	2.7E-07	1.68552	1.74511	1.52609	0.54583	0.70256	0.38093
Prph	-5.70038	0.01915	0.44077	0.04142	0.16773	0.05935	0.03588	0.08025
Lgr6	-5.70725	3.3E-09	3.59964	1.15015	1.05017	0.52505	0.60555	0.57888
Spta1	-5.71222	0.00821	0.12252	0.01594	0.03228	0.01713	0.0069	0.02316
Gm12216	-5.71552	3.8E-06	1.89783	2.07211	2.24536	0.54357	1.03644	0.53712
Tex26	-5.71764	3.1E-05	1.01732	0.56115	1.31286	0.24119	0.35644	0.3986
Dnah12	-5.72341	3.9E-08	7.38306	6.4185	8.22182	2.62811	2.9486	2.27919
Nme9	-5.72772	9.3E-09	4.39651	4.60164	5.58373	1.83134	1.3286	2.07108
4930444P10Rik	-5.74474	0.00192	0.61984	0.14979	0.60652	0.16095	0.0973	0.18136
Tcp11	-5.75256	6.8E-13	3.05915	2.14383	1.79606	1.08561	0.28814	0.82348
Apobec2	-5.75739	5.5E-05	1.64978	0.44398	0.51365	0.22718	0.16481	0.36862
Efcab12	-5.77396	3.5E-08	1.40625	1.16037	1.64454	0.56109	0.47741	0.39339
Mlip	-5.78395	0.00292	0.32996	0.12685	0.01712	0.04544	0.04395	0.03686
Insm1	-5.78524	0.03951	0.14732	0.09968	0.16818	0.08926	0.02158	0
Iqcd	-5.78535	0.00397	1.63103	1.1036	1.73786	0.46117	0.39829	0.80174
Gm45123	-5.80838	0.00222	1.37868	0.6802	1.44275	0.6961	0.2525	0.09412
Lrriq1	-5.81691	3.4E-10	3.09452	2.71474	2.87503	1.02721	0.97675	0.93047
Sox2ot	-5.82201	0.00915	0.091	0.03421	0.03463	0.00613	0.03704	0
Bzrap1	-5.8648	5.9E-06	1.02886	0.85392	1.40507	0.36703	0.52832	0.25996
Tmem232	-5.86886	7.7E-09	3.48538	1.47394	1.83343	0.93165	0.52569	0.64387
Gm12059	-5.87609	0.0001	0.20591	0.29856	0.20149	0.12476	0.02155	0.0482
Zfp354b	-5.87818	0.00029	0.44277	0.45474	0.39716	0.24911	0.04634	0.07773
Kif17	-5.88195	0.00027	0.15947	0.15174	0.36411	0.1087	0.04381	0.06532
Clcn1	-5.88357	0.00279	0.13462	0.07116	0.05763	0.03059	0.02465	0.01379
Ankrd65	-5.88666	2.1E-08	2.83271	1.98681	2.76059	0.79535	0.658	1.01885
Adprhl1	-5.89549	0.02905	0.42113	0.09234	0.03561	0.0756	0.04571	0.01278
Stk36	-5.90125	1.3E-11	2.51154	1.86019	2.12204	0.86831	0.53015	0.76724
Slc1a7	-5.90457	0.00276	0.13592	0.20692	0.0931	0.06588	0.01991	0.04454
Ldb3	-5.91021	1.1E-05	4.03853	0.83467	0.5893	0.46914	0.47827	0.48509
Ccdc110	-5.9154	0.00269	0.17195	0.23269	0.11778	0.08334	0.05039	0
Myoz2	-5.92249	0.04328	3.14865	0.22568	0.09139	0.12934	0.23458	0.45907
Daw1	-5.94405	8.6E-07	1.06703	1.37176	1.51047	0.54305	0.21888	0.55947
Ccdc33	-5.9463	1.6E-05	1.79529	1.47504	2.38134	0.59049	0.71398	0.53229
Vwa3a	-5.95347	1.8E-10	4.2514	3.59576	4.23603	1.4199	1.45272	1.15689
Cfap157	-5.95944	3.8E-06	3.02918	2.99627	2.8134	0.93322	0.45135	1.51424
Ankef1	-5.97848	3.7E-08	0.48307	0.32255	0.36281	0.13864	0.10244	0.11456
Tsnaxip1	-6.0013	4.8E-08	1.88663	2.25477	2.99658	0.86973	0.63097	0.97442
Tnnt3	-6.00656	0.00014	3.11575	0.97602	0.8563	0.34959	0.4227	0.61451
Gm15411	-6.01011	6.2E-06	0.47135	0.48725	0.26905	0.12693	0.1151	0.12872
Tbata	-6.02609	0.01104	0.67769	0.15922	0.47279	0.02281	0.16549	0.21591

Ppil6	-6.02616	2E-08	5.46932	4.49369	5.88701	1.5622	1.43099	2.3044
Mdh1b	-6.04362	5.4E-10	2.28477	1.43808	1.03119	0.35412	0.50603	0.52237
Ccdc39	-6.04994	6.3E-11	5.28906	3.79876	4.3569	1.53908	1.59125	1.31702
Wdr38	-6.0625	8.5E-05	0.66809	0.48434	0.61742	0.28913	0.16315	0.18245
Asb14	-6.08147	1.6E-06	2.14996	1.42441	1.88171	0.71644	0.70877	0.26421
Clstn3	-6.08995	9E-05	0.31255	0.1244	0.12593	0.08911	0.01347	0.04519
C2cd4b	-6.10591	1.7E-08	2.27139	2.44504	2.043	0.83406	0.55467	0.84584
Ryr3	-6.11073	5.6E-07	0.39216	0.15711	0.15316	0.08754	0.0567	0.05496
Dlec1	-6.12471	2.7E-09	2.76905	2.33047	2.89643	1.00828	0.8594	0.81579
Xntrpc	-6.1252	0.01118	0.66012	0.61278	0.42275	0.14632	0.05898	0.19786
Kcnc4	-6.13119	0.00738	0.12454	0.1264	0.08531	0.04527	0	0.04081
4930505A04Rik	-6.14932	0.02739	0.36904	0.15606	0.52662	0.0559	0.13518	0.07558
Aox3	-6.16522	3.5E-20	12.7792	9.14982	5.24841	2.38419	1.8077	3.73453
Wdr49	-6.16866	2.3E-09	1.29228	0.92483	1.13482	0.37141	0.43695	0.21717
Crnde	-6.17796	6.5E-13	1.53965	1.41711	1.13732	0.46642	0.19905	0.51939
Frmpd2	-6.17946	3.4E-09	2.48277	2.24388	2.78576	0.94554	0.82109	0.69738
Car11	-6.18501	6.6E-05	0.37939	0.52143	0.43311	0.0862	0.17371	0.11656
Kremen2	-6.18594	0.02989	0.04317	0.18254	0.34494	0	0.06325	0.10609
Galntl6	-6.19425	0.0044	0.19711	0.1042	0.16877	0.05225	0.02708	0.04037
Tbx1	-6.21659	5.2E-07	3.75219	3.26048	3.87277	1.58826	1.092	0.75797
Phkg1	-6.22014	6.9E-05	0.76303	0.19122	0.22584	0.11986	0.08281	0.11576
Mycl	-6.24288	2.4E-15	2.556	2.29574	2.09163	0.74006	0.66284	0.70419
Mapk10	-6.24405	0.00026	0.45135	0.06247	0.06558	0.02486	0.06012	0.06051
Magix	-6.25572	3.4E-10	1.03579	0.79641	0.57331	0.17115	0.27593	0.23143
Tnnc1	-6.27363	0.01811	14.0384	2.4489	1.80296	1.43533	1.28556	1.79704
Ttc25	-6.28349	2.2E-10	1.81278	1.42012	1.22133	0.48615	0.32657	0.53562
F11	-6.28668	0.03999	0.1631	0.20692	0.0931	0.02471	0.05974	0.03341
Ttll13	-6.2937	3.7E-06	0.30592	0.2957	0.19956	0.02648	0.04802	0.16111
Iqch	-6.30019	3.6E-05	0.18495	0.25698	0.15081	0.08004	0.01936	0.04329
Ect2l	-6.30449	7.1E-14	1.459	1.25214	1.20021	0.38999	0.4244	0.33398
Rbbp8nl	-6.33585	0.0279	0.15755	0.19988	0.08993	0.04773	0	0.06454
Gm12576	-6.34644	0.03814	0.12034	0.15267	0.06869	0.03646	0.02204	0.02465
Wnk2	-6.36768	1.1E-05	0.63541	0.18218	0.27048	0.09788	0.11046	0.08823
Bcan	-6.37647	8.8E-05	0.62929	0.57026	0.07697	0.06809	0.16465	0.09206
Klhl38	-6.41265	0.00112	1.48651	0.94295	0.56567	0.57541	0.33275	0.30446
Ccdc190	-6.42656	0.0026	0.2107	0.26731	0.48107	0.15957	0.07718	0.08631
Mypn	-6.44366	0.01326	1.20135	0.11781	0.17889	0.09494	0.17857	0.07132
RP23-35O24.3	-6.44752	0.03848	0.32621	0.34487	0.2793	0.04941	0.11949	0.06681
Wdr93	-6.46181	2.5E-11	1.43902	1.08186	1.04956	0.33906	0.23427	0.42572
Tnnt2	-6.5093	0.00107	27.6604	4.58397	3.47572	2.10632	3.11629	3.025
Myom2	-6.51042	0.02258	2.5896	0.45375	0.22378	0.19378	0.30233	0.22821
Kndc1	-6.51325	3.5E-10	2.47342	1.94049	2.42447	0.68212	0.61234	0.8385
Ccdc30	-6.5336	8.6E-09	2.32249	1.91756	2.58824	0.77059	0.72919	0.71352
Cyp2b19	-6.53517	0.00175	0.19718	0.1853	0.05002	0.01327	0.04815	0.05385
Sptbn4	-6.53943	0.01953	0.0382	0.01616	0.07633	0.04051	0.03499	0.03912
Ccdc150	-6.5395	0.00023	0.58212	0.49234	0.76838	0.08817	0.33317	0.13413
Snhg11	-6.54311	2.1E-36	3.37652	2.90113	1.37054	0.58451	0.6989	0.77279
Gm14963	-6.54319	0.00029	0.21424	0.3624	0.24458	0.02163	0.10463	0.08776
Gm20683	-6.54442	0.03505	0.85117	0.58146	0	0.07934	0.11992	0.21456
Fn3k	-6.55595	8.9E-07	0.29069	0.29854	0.14222	0.07548	0.06084	0.05103
Fam227a	-6.56186	6.2E-13	1.16885	1.14159	1.1914	0.33723	0.35679	0.342
Gm29455	-6.56273	0.03064	0.04698	0.09935	0.21455	0	0.03442	0.07698
Paqr9	-6.592	0.0362	0.29474	0.01847	0.01246	0.02646	0.02399	0.01789
Srcin1	-6.59324	8.1E-11	1.74877	2.06102	1.64731	0.54425	0.53906	0.52454
Kcnk12	-6.61562	7E-09	2.02868	1.59611	2.15437	0.60028	0.38019	0.73437
Rgs22	-6.61669	1.9E-09	6.92784	4.9963	6.07167	1.71228	1.96973	1.67215

Gm16008	-6.64125	0.01466	0.62087	0.9919	0.70879	0.37618	0.55593	0.28259
Gm9806	-6.68498	0.00027	5.12947	8.11703	4.15575	1.20304	1.33342	2.03339
Aqp3	-6.68644	0.01302	1.59803	0.59132	0.62711	0.45385	0.03658	0.24547
Fam179a	-6.69698	6.7E-09	4.56363	5.19224	5.52505	1.65311	1.59431	1.38375
Slc7a10	-6.71391	3.9E-15	2.50951	1.35401	0.98789	0.68159	0.12679	0.49626
Vat1l	-6.71539	0.01685	0.05017	0.08486	0.01909	0.02026	0	0.0137
Tex11	-6.72093	0.00058	0.27724	0.34107	0.02877	0.07635	0.01846	0.06194
Wdr27	-6.75488	3.6E-05	0.35485	0.66027	0.4051	0.1075	0.12998	0.14536
4932443I19Rik	-6.78422	4.5E-08	0.83744	0.66	0.97775	0.26523	0.20915	0.24948
Ak9	-6.78507	2.3E-11	2.98866	2.66079	2.7684	0.92127	0.80665	0.71951
A430108G06Rik	-6.80701	4E-07	1.51824	1.23274	0.62396	0.36795	0.22245	0.24877
Plk5	-6.80776	7.1E-07	0.85267	0.46155	0.62298	0.18598	0.14992	0.16765
Thrsp	-6.81986	0.00025	3.93388	1.20989	0.16331	0.65004	0.1572	0.35158
BC024139	-6.82203	0.0125	0.10151	0.05151	0.18541	0.0369	0.01487	0.03326
Ccdc78	-6.83102	2.3E-07	6.3621	8.04803	10.2945	2.41337	2.18856	3.17264
Per3	-6.83347	1.4E-23	9.41135	8.31373	4.78598	2.66101	1.88037	2.05608
Wdr72	-6.86623	1.8E-08	0.61006	0.28895	0.41787	0.12567	0.08938	0.13994
Dmrt2	-6.86983	2.5E-06	0.54483	0.52664	0.53313	0.14147	0.11404	0.15941
Pla2g4c	-6.88038	0.00843	0.0975	0.26802	0.58439	0.13292	0.14286	0.01997
Ldlrad1	-6.88368	1.7E-06	7.77163	5.3009	11.2571	2.88598	1.8366	3.01233
D030018L15Rik	-6.88849	5.7E-07	0.76362	0.60998	0.84754	0.21848	0.20201	0.20853
Gm28539	-6.89845	0.00017	0.70448	1.11396	0.59444	0.05567	0.4039	0
Serpina9	-6.914	1.4E-05	0.8204	0.46259	0.62438	0.16569	0.12749	0.14257
Gm3336	-6.91677	0.00041	1.03161	0.67117	0.18118	0.2404	0	0.19504
H2-K2	-6.92634	8.3E-05	2.78628	4.05038	3.57843	1.31887	1.14818	0.35667
Ces1f	-6.93977	1.7E-08	1.58485	0.58791	0.22219	0.28639	0.08148	0.18223
Lrrc71	-6.98437	1.1E-07	4.39432	4.39548	6.25354	1.32514	2.07267	1.41373
P2rx6	-7.00077	1.9E-07	2.42348	2.06907	2.74055	0.74802	0.61972	0.59938
Dcdc5	-7.00434	4.8E-09	1.62244	1.23027	1.41788	0.38642	0.50822	0.27501
Adam33	-7.00741	7.6E-09	1.65945	1.84751	1.60447	0.42064	0.52102	0.47167
Myl3	-7.05509	0.0006	0.6256	0.37794	0.30608	0.13537	0.03274	0.18305
Txlnb	-7.08538	0.00136	2.68835	0.32282	0.09472	0.19104	0.19452	0.24472
Rnf207	-7.10475	6E-05	0.27411	0.14643	0.14823	0.01748	0.08455	0.03546
Noxa1	-7.10929	0.00031	0.3954	0.66885	0.77381	0.10267	0.08276	0.32393
Ugt1a5	-7.11533	0.01594	1.45112	1.1572	1.27795	0.55264	0.5771	0.37364
Cecr6	-7.14179	7.8E-10	0.52525	0.41252	0.17132	0.09093	0.0962	0.07684
Dthd1	-7.16215	0.0073	0.45161	0.19098	0.55852	0.11401	0.16542	0.03083
Lmod3	-7.17257	0.00455	0.53607	0.06975	0.09415	0.04997	0.06042	0.03378
Ttll3	-7.20478	2.3E-07	4.16736	4.67403	5.81239	1.53691	1.71232	0.93518
Krt79	-7.2309	1.3E-11	2.17782	2.17351	0.94476	0.31668	0.44673	0.53526
AI480526	-7.2461	3.9E-06	3.94346	3.9893	1.80958	1.42888	2.32249	1.33029
Lmod2	-7.25388	0.01172	5.87038	0.89919	0.31661	0.25205	0.44022	0.71951
Rgs11	-7.2875	2.2E-10	2.75793	2.81016	2.28078	0.72365	0.79545	0.3914
Cacna2d2	-7.28854	0.01094	0.24151	0.0383	0.03446	0.01829	0.02212	0.02473
Fam83e	-7.30497	1.1E-05	0.83185	1.0855	2.00783	0.4176	0.24376	0.56467
C130021I20Rik	-7.33036	0.00126	0.15444	0.13062	0.27705	0.0401	0	0.10845
Ccdc60	-7.34515	2.1E-06	1.08758	1.31408	1.35245	0.48244	0.29878	0.55688
Asgr1	-7.35258	0.01048	0.45927	0.19422	0.13108	0.10435	0	0.04703
Pla2g2c	-7.36723	0.00057	0.15172	0.23526	0.23094	0.04596	0.05558	0.02072
Ccdc155	-7.37223	1.5E-05	0.69517	0.81661	1.2345	0.32759	0.25464	0.1582
Lrrc10	-7.40414	0.01781	1.66301	0.16229	0.07302	0.11626	0.09372	0.1572
Cfap44	-7.41818	7.5E-09	9.05525	7.42228	10.7058	2.90611	2.8757	1.78856
Gm45336	-7.43078	0.00674	0.11311	0.1435	0.12913	0.01713	0.06215	0
Gas2l2	-7.4392	1.9E-07	2.2047	2.10148	3.23601	0.59368	0.87167	0.63074
Armc2	-7.43969	1.3E-11	1.19445	0.9995	1.40711	0.38495	0.31651	0.27067
Fam161a	-7.45441	2.3E-11	6.815	5.69371	7.06845	1.91348	1.7809	1.61709

Aqp7	-7.47248	0.04561	0.45217	0.05463	0.03687	0	0.09464	0.02646
Erich5	-7.47495	3E-09	1.3839	0.65027	0.96548	0.18633	0.30978	0.22046
5330413P13Rik	-7.48361	1.3E-12	0.92613	0.55217	0.48531	0.17478	0.16684	0.12438
Cfap221	-7.52477	2.1E-13	1.56668	1.06006	1.00158	0.30375	0.22955	0.33371
C530043A13Rik	-7.52878	0.01213	1.45278	0.436	1.3375	0.39752	0.37765	0.07679
Hmcn2	-7.5335	2.2E-16	1.22177	0.47102	0.42168	0.16527	0.17069	0.14432
Zfp683	-7.53963	0.00888	0.24837	0.36762	0.21265	0.15048	0	0
Pax9	-7.59683	0.00021	0.4155	0.18335	0.24748	0.10945	0.02647	0.0592
Adgrv1	-7.63033	1.9E-10	0.49776	0.3711	0.47985	0.14521	0.11615	0.09364
Dytn	-7.63854	0.00035	0.14869	0.12576	0.0679	0.03603	0.03268	0
Ccdc114	-7.68732	1.6E-08	2.34122	2.53867	3.03254	0.81837	0.68167	0.62706
Cacna1b	-7.72232	9.1E-06	0.12862	0.11655	0.12586	0.02227	0.03365	0.03011
4930426I24Rik	-7.74025	0.03486	0.43016	0.24255	0.49107	0.08688	0	0.11747
Ttc16	-7.76229	1.6E-11	2.00176	1.86236	2.28522	0.63241	0.50279	0.46856
Dnah3	-7.82164	9.5E-10	7.82802	7.02583	9.81121	2.41785	2.21719	2.02438
Ccdc187	-7.83963	6.7E-11	6.38536	4.77959	5.95261	1.59932	1.43673	1.34779
Mybpc3	-7.87589	0.00538	5.88001	0.8165	0.60113	0.3988	0.49827	0.46734
Pisd-ps2	-7.90354	4.5E-07	4.59288	6.77968	6.33147	1.78516	1.96804	1.38441
D830044I16Rik	-7.90742	0.00011	0.74029	0.56649	1.32804	0.21358	0.23243	0.25992
Alox15	-7.91697	0.00368	0.85606	0.4722	0.63736	0.0902	0.13634	0.24394
Mesp1	-7.92164	3.4E-05	0.78801	0.6059	0.32713	0.13021	0.05248	0.17607
Gm14062	-7.94363	0.02716	0.11047	0.11212	0.02522	0	0	0.0543
Agr2	-7.95976	0.00038	1.27349	0.95742	2.09997	0.72872	0.20732	0.17389
Gm2568	-8.05901	0.00611	0.31334	0.46378	0.35771	0.09492	0.11477	0
Adh7	-8.06186	1.3E-07	2.50835	1.04036	1.51437	0.42378	0.31805	0.41495
Casq2	-8.08617	0.00283	4.87401	0.73412	0.49544	0.24272	0.36685	0.51965
Scn5a	-8.1124	1.9E-11	2.4283	1.37509	1.51101	0.53041	0.38175	0.30738
Ccdc180	-8.11508	1.9E-08	4.97075	4.87568	6.32486	1.42219	1.39087	1.3433
Myl2	-8.1552	0.04623	0.09368	0.07923	0.21389	0.02838	0.03431	0
Ttc34	-8.15772	7.3E-10	1.88249	1.23316	1.70661	0.49201	0.50026	0.51408
Ccna1	-8.20508	0.00343	0.1991	0.1684	0.22729	0.03016	0.03647	0.04078
Slc16a11	-8.21746	7.6E-11	10.5857	6.04947	7.71625	2.16679	1.51957	2.10951
Cntn2	-8.24151	0.02945	0.30433	0.01661	0.01121	0.02379	0.02158	0.00804
Eef1a2	-8.24594	0.01366	4.77725	0.37069	0.40027	0.10622	0.54584	0.28725
Ggnbp1	-8.24657	8.3E-12	0.71039	0.73435	0.64578	0.15941	0.28913	0.11855
Mcidas	-8.24679	0.00389	0.19401	0.13127	0.26577	0.02351	0.02843	0.06358
Ryr2	-8.26214	1.4E-06	6.85208	1.72394	1.42956	0.66346	0.7069	0.56403
Atcayos	-8.34495	0.00052	0.41754	0.1236	0.02383	0.0506	0.04588	0.06842
Gm45055	-8.3714	0.00034	0.888	0.64376	1.15857	0.23058	0.27881	0
Fhad1	-8.38019	9.3E-10	7.03512	6.8685	8.86333	1.94649	1.93952	1.98618
Gm12695	-8.38549	1.1E-06	2.25466	1.8258	4.27163	0.75569	0.98402	0.39301
C330002G04Rik	-8.40172	4.5E-10	1.24511	0.7146	0.71071	0.08083	0.32577	0.14572
Insrr	-8.43054	0.02895	0.11189	0.14872	0.18248	0.04842	0.02342	0.02619
Pklr	-8.46252	0.0013	0.16935	0.21485	0.12889	0.06841	0.02068	0
Fer1l4	-8.52254	1.3E-06	0.23841	0.24645	0.30241	0.07222	0.01941	0.08681
Mapk15	-8.54924	1.1E-07	7.0973	6.45524	9.24234	1.7503	1.77669	2.10374
Pcsk4	-8.55422	2.7E-08	0.74108	0.49621	0.74026	0.07483	0.18097	0.17708
Ankrd61	-8.56195	0.01843	0.50939	1.06051	0.44733	0.641	0.976	0.32102
Gm12963	-8.57529	0.00398	0.87748	0.20241	0.45533	0.09666	0.11688	0.06535
Ppp1r1b	-8.71533	1.1E-05	1.43495	0.65959	0.81907	0.2268	0.04571	0.53668
Gm15889	-8.73544	1.7E-05	2.19278	1.92594	0.28884	1.02197	0.67964	0.48366
Gm20661	-8.78735	4.1E-07	3.72116	4.18447	3.86191	1.25539	1.33207	0.41572
Gm20662	-8.8421	0.00057	0.83796	1.54253	3.26376	0.56744	0.79444	0.56536
Lrrc36	-8.88874	3.9E-07	4.80158	4.60662	7.85411	1.17236	1.73255	1.26232
Ttll10	-8.9114	1.9E-06	1.21478	1.55085	2.06711	0.36106	0.52053	0.30044
Dnah2	-8.91575	2.3E-11	2.64835	2.79217	3.1209	0.76163	0.62139	0.61991

Six1	-8.95597	3E-10	1.77132	0.91652	1.57014	0.35353	0.229	0.35852
Bbof1	-9.06414	1.2E-13	5.56039	4.39166	4.94753	1.18904	1.19809	1.07186
Cfap73	-9.10065	2E-05	2.08262	1.84532	3.17003	0.66095	0.21796	0.73123
Flt3l_1	-9.11161	7.7E-08	2.57992	4.06496	3.49155	0.92023	1.14317	0.57954
Pln	-9.11387	0.02554	11.0857	1.52635	0.29431	1.42813	1.75378	2.02159
Xirp2	-9.12057	0.03156	1.82233	0.07739	0.04352	0.11087	0.08379	0.11869
D430001F17Rik	-9.13352	0.00073	0.58991	0.16631	0.2806	0.11914	0.10804	0.04027
Ces1e	-9.17998	2E-14	2.33664	1.05873	1.5243	0.42977	0.21398	0.30766
Gm1667	-9.1928	0.0297	0.16369	1.10755	0.18687	0.09918	0	0.1341
Smyd1	-9.23467	0.01122	1.34615	0.11895	0.11468	0.06087	0.0736	0.11522
Npm2	-9.2662	0.01416	0.36645	0.30994	0.20917	0.05551	0.06711	0
Doc2g	-9.2687	1.4E-07	1.56906	0.66354	0.51671	0.2011	0.22105	0.19776
Lrrc23	-9.28794	1.3E-08	11.6411	12.6525	15.5746	2.71274	2.43111	3.97016
Hcar1	-9.29561	0.00257	0.16683	0.04703	0.15871	0.03369	0.02037	0
Myl4	-9.32225	0.0045	14.0641	0.84965	0.31482	0.75187	0.66379	0.80687
Abcc8	-9.32732	3.6E-05	0.32742	0.16233	0.07733	0.02052	0.04136	0.037
Reg3g	-9.32851	0.00022	16.5047	15.5307	9.7662	3.534	2.31063	2.44238
Obscn	-9.33312	0.00023	2.15875	0.85277	0.61701	0.2164	0.28249	0.13724
9130230L23Rik	-9.33413	2.9E-14	1.34903	1.11414	1.19582	0.21155	0.29067	0.24705
Actn2	-9.36549	0.00191	7.76394	1.33842	0.64923	0.50935	0.47097	0.54694
Ccdc17	-9.38573	4.5E-09	12.316	15.625	22.0944	3.45295	3.81081	3.22755
Plekhd1os	-9.45685	0.00488	0.77405	0.32734	0.22091	0.17587	0	0
Gp2	-9.48103	1E-06	2.10287	2.47453	2.24408	0.66474	0.26792	0.48687
Ribc2	-9.49289	3.2E-06	0.4976	0.7482	0.31559	0.1005	0.16202	0
Otx1	-9.49578	8.5E-05	0.25269	0.15673	0.38462	0.07145	0.06171	0.0276
Gm45623	-9.49702	0.00137	0.25076	0.53021	0.64409	0.07596	0.13778	0.05136
Pigr	-9.52346	2.8E-13	11.0059	14.1644	17.072	3.38226	2.59341	3.5323
2310002F09Rik	-9.52363	0.01397	0.11324	0.05746	0.07756	0.01372	0.01659	0
Cfap100	-9.53463	2.4E-10	4.09933	4.01064	5.23634	0.81895	0.97399	1.12552
Plch2	-9.5909	4.4E-07	0.75439	0.60948	0.89978	0.14326	0.15672	0.18449
Cdh26	-9.60561	0.01129	0.90974	0.25648	0.20771	0.12861	0.02222	0.07453
Scgb3a1	-9.69857	7.2E-08	162.819	85.5195	107.061	31.8343	16.8962	22.6489
Dnaaf3	-9.71613	1.1E-07	4.23011	4.16826	7.31401	1.66716	1.384	0.97574
Mylk3	-9.72818	0.0035	2.6657	0.41485	0.09738	0.15505	0.18748	0.10483
Gm26917	-9.7596	0.00078	7364.29	15908.3	35842.1	4858	6198.53	3689.09
Ttn	-9.75987	0.00025	11.0543	1.83897	0.78098	0.65039	0.77323	0.55853
Cfap74	-9.76367	2.3E-11	2.05604	2.00291	2.7768	0.6451	0.55812	0.34591
Slc5a8	-9.83435	0.00676	0.80678	0.29037	0.47031	0.1456	0.08803	0.05625
Cfap46	-9.88131	2.3E-11	4.39656	4.43879	5.67479	1.12192	1.04461	0.88425
Dnah10	-9.91971	6E-10	5.47367	5.23944	7.20812	1.34858	1.21464	1.26994
Ccdc13	-9.93951	2E-08	1.54991	1.74031	2.01343	0.42096	0.29365	0.39407
Ybx2	-10.0031	0.01279	0.09691	0	0.60848	0.02936	0.0355	0.07939
Ccdc108	-10.0514	7.4E-10	10.7848	9.69476	13.4991	2.52735	2.49095	1.69435
Gm12474	-10.062	1.4E-06	1.55468	0.38352	1.03532	0.15699	0.28474	0.05307
Odf3b	-10.0662	1.6E-08	10.4612	9.44271	17.964	2.87618	2.25406	3.24091
Hhatl	-10.1903	9.8E-10	0.90468	0.41201	0.59584	0.12649	0.12745	0.05701
Pde4c	-10.2569	1.2E-12	0.42827	0.37616	0.20685	0.05988	0.04827	0.05398
Kcna1	-10.2663	8.2E-08	0.36306	0.19394	0.13088	0.02315	0.028	0.06262
Gm12847	-10.2812	0.01072	0.44231	0.56115	1.26237	0.26799	0	0
Acvr1c	-10.3429	0.00018	0.13762	0.07482	0.02244	0.00596	0	0.03221
RP24-170O17.2	-10.4049	0.00011	0.13632	0.26904	0.41501	0.05506	0	0.07446
Cilp2	-10.437	0.00056	0.40227	0.08954	0.04834	0.01283	0.01551	0.05204
Capn8	-10.4755	0.00134	0.04845	0.20491	0.24892	0.01468	0.05325	0.01985
Crocc2	-10.4888	5.1E-06	1.53681	1.38646	3.33341	0.48108	0.56293	0.30427
Mettl7a3	-10.4912	3.4E-07	1.01612	0.66843	0.32222	0.13681	0.12407	0.18499
Mylk4	-10.5138	0.00228	1.31164	0.12606	0.10209	0.04515	0.08735	0.06105

Mlxipl	-10.5208	6.7E-12	0.87412	0.36268	0.15063	0.06995	0.06041	0.08107
D930020B18Rik	-10.7529	2.4E-10	0.73801	0.53503	0.6018	0.06388	0.10298	0.14396
Myh6	-10.7658	0.00144	103.929	14.0945	8.03352	4.53391	5.22908	7.16811
Spdef	-10.8299	8.7E-06	0.50094	0.74145	1.33437	0.17705	0.12233	0.2052
Cyp4a12b	-10.9273	0.00012	0.99156	0.51609	0.56598	0.11553	0.08382	0.03124
Csrp3	-11.1281	0.02129	5.60815	0.31815	0.11712	0.20719	0.15031	0.39221
Ccdc129	-11.2317	2.3E-15	2.0105	0.79641	1.4236	0.24671	0.20508	0.25019
Wdr95	-11.3071	6.1E-06	1.18211	0.91094	1.6194	0.25466	0.23093	0.19369
Cyp2a5	-11.3103	8.9E-06	47.4532	6.96868	10.8145	4.88528	1.25886	3.93456
Ky	-11.3922	2.7E-09	1.11861	0.73586	0.86908	0.22592	0.06829	0.16547
Entpd8	-11.6576	5.8E-05	0.13457	0.39835	0.15362	0.04077	0.02465	0.02756
Trim54	-11.7944	0.00129	1.78096	0.21519	0.07261	0.07708	0.09319	0.10422
Mybphl	-11.8073	0.01773	8.87607	0.48434	0.16343	0.13011	0.41952	0.52779
Aspg	-11.8303	1.2E-05	0.40119	0.50898	1.10683	0.10128	0.17145	0.08217
Slc35d3	-11.8682	0.00013	0.48621	0.11749	0.31718	0.02104	0.02544	0.08536
Bpifb1	-11.9348	3.7E-06	31.4644	16.168	18.7095	5.58447	2.21996	2.87565
Slc22a13b-ps	-11.9785	0.04588	0.12529	0.21194	0.21455	0.11387	0.04589	0
Trim63	-12.0346	0.00825	0.80346	0.16309	0.07338	0	0.07063	0.05266
Sec14l5	-12.0509	9.3E-05	0.54717	0.39667	0.89235	0.09472	0.02863	0.19212
Ttll6	-12.1134	3.7E-12	2.8654	2.50362	3.13598	0.54522	0.5378	0.31041
Gm29266	-12.2429	2.1E-05	1.52229	1.41628	1.73786	0.09223	0.22304	0.37414
Oit1	-12.3855	5.7E-05	1.16819	0.67927	0.5001	0.17695	0.05349	0.05982
Ascl1	-12.5819	6.4E-07	0.52563	0.20518	0.41542	0.04899	0.02962	0.06625
Acp7	-12.6294	0.00896	0.24553	0.10383	0.23358	0	0.02998	0.03353
Klhl34	-12.775	0.0001	0.42483	0.23954	0.21555	0.0572	0	0.03867
Cpt1b	-12.9492	0.00762	3.25045	1.07676	0.12369	0.18053	0.53578	0.06657
Slc5a9	-12.9851	0.00075	0.15857	0.11922	0.26148	0	0.02582	0.05774
Gm37381	-13.0196	0.00011	0.5357	0.33982	0.38223	0.08114	0.04906	0
Gmnc	-13.0931	1.7E-14	0.73822	0.41625	0.33199	0.08132	0.06555	0.03665
Slc17a7	-13.0936	0.01066	0.95483	0.11818	0.05317	0.04233	0.06824	0
Atp6v1b1	-13.2606	2.6E-13	0.91734	0.35267	0.52362	0.10105	0.07637	0.05124
Atp2b2	-13.2964	7.2E-25	1.12253	0.88816	0.85157	0.14919	0.11142	0.14833
E030044B06Rik	-13.3051	7.3E-06	0.64932	0.42714	0.6589	0.08742	0.05285	0.05911
9030622O22Rik	-13.4267	3.8E-12	1.68339	1.83714	2.35572	0.23031	0.27847	0.3559
RP23-199N18.2	-13.468	0.02757	0.58456	0.37081	0.33367	0.08854	0	0
Gm45218	-13.4986	0.0295	0.21773	0.13811	0.12428	0.03298	0	0
Sox21	-13.5558	3.9E-05	0.45681	0.24402	0.43915	0.05827	0.03523	0.07879
Cidec	-13.5944	8E-06	4.4783	0.99895	0.16854	0.38762	0.0721	0.12095
Flg	-13.6471	0.00141	0.0504	0.09592	0.32367	0.03054	0.02769	0.02581
Dbp	-13.8406	5E-41	20.2939	11.8988	8.84897	2.03387	1.0284	3.10016
Art1	-13.9724	0.01305	0.97548	0.16501	0.11136	0	0.07146	0
Trdn	-14.3637	0.00695	1.13312	0.17114	0.0231	0.07356	0.02965	0.01658
Dydc1	-14.4485	4.6E-06	0.82617	0.85404	2.2007	0.05562	0.269	0.22561
Gm15751	-14.6299	0.0009	1.27539	0.70531	0.896	0.56469	0.86247	0.48225
Tymp	-14.7468	2E-08	4.34941	5.34732	7.98544	0.97802	1.14971	0.95511
Lrrc43	-14.7536	5E-08	3.581	4.41225	6.91442	1.01787	0.90686	0.25353
Cyp2e1	-15.0001	5.2E-07	5.20599	0.57277	0.24159	0.2308	0.15504	0.17338
Mhrt	-15.3014	0.0006	1.27903	0.82724	0.25767	0	0.16535	0.06164
T2	-15.4895	4.9E-09	1.49649	2.26954	2.76879	0.37519	0.18902	0.33821
Gm21887	-15.743	0.00373	9.26613	49.262	141.639	11.0826	24.1558	8.873
Tcap	-15.7573	0.00025	26.7847	5.2968	3.29973	0.75888	1.19992	1.4208
Hamp	-15.8778	0.01024	8.91098	0.37684	0.50864	0.26995	0	0.73004
Hrc	-15.9392	0.00049	4.62739	0.33116	0.04064	0.08627	0.13038	0.20413
Fut9	-16.2875	3.7E-06	0.14536	0.11735	0.14331	0.02402	0.01936	0
Tnni3	-16.3738	2.9E-07	22.8897	5.13015	4.19512	0.9657	1.16766	0.94307
Foxa3	-17.1316	7.4E-06	0.35871	0.41717	0.87021	0.13584	0	0.03673

Clca3b	-17.6253	0.00931	0.16305	0.09194	0.06205	0	0	0.02226
Gm44868	-17.7313	0.00565	0.70695	0.59792	0.32282	0.08567	0	0
Gm44963	-17.8277	0.00044	0.98081	0.10369	1.11969	0.07428	0	0.10044
Tmem182	-18.1176	0.00731	1.37881	0.02844	0.03839	0.02038	0.04927	0.02755
Lypd2	-18.4841	3.6E-25	17.6218	10.4671	14.2817	1.30403	0.98547	2.20409
Cfd	-18.8272	2.6E-05	20.4842	2.59459	1.80752	0.35974	0.94244	0.5675
Sspo	-19.0418	2E-08	0.40881	0.50647	0.89396	0.1221	0.05905	0.0283
Tacr1	-19.647	1.5E-07	0.70357	0.40465	0.72288	0.09378	0.06185	0.02306
Foxn4	-19.7579	2.9E-05	0.08129	0.18333	0.27839	0	0.01985	0.0222
Mpz	-20.1508	2.6E-05	0.90862	2.67142	0.04939	0.10486	0.09509	0.07089
Gm43634	-20.2451	0.00033	1.62378	4.00565	9.88648	0	1.38783	0
Chad	-23.1462	4E-21	11.3085	7.26534	9.93062	1.5482	0.87625	1.06898
Fitm1	-24.5417	0.00894	1.50815	0.15945	0.10761	0.05711	0	0
Kcnj3	-24.6878	0.00594	2.90739	0.19257	0.11997	0.02122	0.06415	0.07174
Krt15	-24.724	0.01191	2.63623	0.43719	0.29505	0.06264	0.07574	0.04235
Cdc20b	-25.2305	0.0066	0.09993	0.04226	0.51337	0.03027	0	0
Alox12e	-25.8287	0.00195	0.12928	0.19135	0.18449	0.01958	0	0
Ckmt2	-25.9146	1.4E-07	7.9905	1.40363	0.28068	0.22344	0.09006	0.20142
Krt5	-25.9599	0.00161	0.66731	0.21165	0.23806	0.05054	0	0
Nppa	-26.9967	0.00606	33.8952	4.10819	1.80818	0.89567	0.61885	0.51904
Mb	-27.4033	0.00101	37.0603	1.85108	0.66627	0.57462	0.48101	0.89651
BC027072	-30.0285	0.04611	0.03651	0.06175	0.04168	0	0	0
Lyzl4	-30.5786	0.02994	0.1303	0.1653	0.22312	0	0	0
Myl1	-31.7914	0.00477	2.4662	0.11174	0	0.05337	0.03226	0
Car3	-34.8932	3.4E-05	13.1666	0.94374	0.36395	0.05795	0.25691	0.23506
Gm11771	-35.3639	0.04043	0.87345	0.31661	0	0	0	0
Muc5b	-35.8005	3.1E-16	39.0217	30.975	38.4712	2.92158	1.87361	1.36016
Lgals12	-36.2555	1.5E-17	1.51516	0.55717	0.63924	0.07983	0	0.02698
Tmem45b	-38.0407	1.6E-05	0.84965	0.23954	0.16166	0	0	0.03867
Tff2	-42.6193	9E-12	10.6352	15.4768	17.6762	1.23188	0.45831	0.3844
Gm26887	-47.6536	0.00363	1.16885	0.56491	0.38125	0	0	0
Gm5737	-48.2543	0.00209	0.35903	0.30366	0.24592	0	0	0
Dusp15	-48.3652	0.00214	0.07919	0.15629	0.09041	0	0	0
Ctcflos	-49.5563	0.00482	0.19708	0.04546	0.02045	0	0.01313	0
Acnat1	-50.1095	0.00177	0.21461	0.14521	0.343	0	0	0
Ckm	-52.0233	3.4E-06	3.76262	0.53039	0.17898	0.05699	0.02297	0
A530016L24Rik	-52.5525	0.01527	0.4158	0.11723	0	0	0	0
Gm15883	-53.0215	0.00077	11.3496	7.45091	7.77409	0.75315	0.6335	0.44277
Adipoq	-53.6459	0.00014	2.38099	0.27969	0.15101	0	0.04845	0
Plin1	-54.6625	1.1E-05	2.29867	0.13293	0.06729	0.02381	0.01439	0.0161
Gm13304	-57.0775	0.0231	7.63875	3.32266	0	0	0	0
Ern2	-71.6354	1.6E-06	0.4006	0.31276	1.0202	0.03734	0	0
Gm18595	-83.8866	1.1E-05	0.94074	0.57866	0.39053	0	0	0
Fcgbp	-91.0291	2.1E-08	0.99195	5.28406	7.87723	0.15289	0.05737	0.17109
Muc5ac	-111.863	1.5E-09	0.95143	5.11563	7.64192	0.14411	0.07468	0.07655
Fer1l6	-118.392	1.9E-06	0.28355	1.33342	4.15636	0.08246	0.00831	0.03717
Pck1	-125.604	4.6E-09	7.48494	0.54069	0.18245	0.01614	0.05854	0
Capn9	-128.076	0.01305	0.03784	0.19201	0.64792	0	0	0
Gm28635	-171.921	0.00303	0.15435	1.74608	1.05725	0	0	0
Smpx	-174.424	0.00063	2.24831	0.13583	0	0	0	0
Gcom1	-200.665	1.2E-05	7.6732	10.339	7.914	0	0.23261	0
RP23-325D10.3	-232.8	2E-09	1.30731	1.68067	0.73627	0	0	0
Chil4	-301.531	0.00082	0.05935	1.90745	6.1655	0	0.04348	0
Clca1	-1925.78	3.4E-10	3.86921	59.1916	83.4558	0.10256	0.01772	0.05944
