# Supplementary material for: Transcriptomic Evaluation of Pulmonary Fibrosis-Related Genes: Utilization of Transgenic Mice with Modifying p38 Signal in the Lungs
Source: Int J Mol Sci. 2020 Sep 14;21(18):6746. doi: 10.3390/ijms21186746 (PMC7555042; doi:10.3390/ijms21186746)
Supplement: Supplementary file 1 [file ijms-21-06746-s001.zip › Supplementary Table S3.pdf]

Supplemantary Table S3. DEGs altered by BLM in WT group.

Name (symbol)	Fold change	FDR p-value correction	WT_PBS_Rep1 - RPKM	WT_PBS_Rep2 - RPKM	WT_PBS_Rep3 - RPKM	WT_BLM_Rep1 - RPKM	WT_BLM_Rep2 - RPKM	WT_BLM_Rep3 - RPKM
Srp54a	240.446	0.00227	0	0	0	7.0086	3.34187	0
Ighv8-12	190.667	0.00209	0	0	0	8.73527	9.20892	1.23772
Bcl2a1b	168.924	1.1E-06	0	0	0	10.6819	7.06168	1.844
Serpinb9b	166.107	1E-06	0	0	0	0.69306	0.81439	0.13621
Celf5	156.579	3.3E-07	0	0	0	0.19632	0.30018	0.31897
Tfpi2	152.853	5.4E-07	0	0	0	1.24888	1.21315	0.30436
Flrt3	146.185	9.9E-06	0.04761	0.09559	0.17947	2.92998	3.23097	0.63756
Fcgr4	140.201	5.8E-06	0	0	0	2.25822	1.12493	0.28223
Clec4e	139.723	2E-05	0	0	0	0.36619	0.36758	0.16064
Ms4a1	136.286	0.00014	0.03744	0.0451	0.01283	1.11669	1.41778	0.22918
Igkv5-43	132.266	0.00382	0	0	0.20943	4.22158	9.32	0.70148
Trbc1	126.359	2.2E-05	0	0.12418	0	3.39842	3.6942	0.47327
Tlr6	110.488	6E-05	0	0	0	0.46832	0.44229	0.09511
Gpr176	105.837	6.5E-05	0	0.02385	0	1.41759	1.08713	0.22729
Ighv1-80	102.058	0.00328	0	0	0	0.62159	8.55395	0.75744
Rgs16	101.743	7.6E-05	0.09903	0.11929	0.06787	1.86557	3.75025	1.40186
Gm6377	100.589	7.6E-05	0	0	0	0.54642	0.42126	0.19024
Mmp12	98.3318	4.1E-15	0.04801	0	0	3.73831	5.45843	0.95515
Mmp13	92.3228	0.00059	0	0	0	0.43532	0.59466	0.06631
Ighv1-47	90.4888	0.01496	0	0	0	6.77801	0.74145	0.44645
C430002N11Rik	86.9772	0.00217	0	0	0	0.41341	0.66931	0.2099
Gjb4	86.9211	0.00125	0	0	0	0.75442	0.9364	0.18386
Cemip	86.7579	2.4E-19	0.00947	0.0076	0.00649	2.30688	2.26687	0.38398
Tarm1	86.6995	0.00069	0	0	0	0.56322	0.45593	0.30026
Spp1	86.3002	2E-48	0.28111	0.84656	0.81877	184.419	177.9	33.3403
Igkv6-15	81.1694	0.0167	0	0	0	0.83834	6.27735	0.51078
Sirpb1b	74.2333	0.00198	0	0	0.02759	0.58141	0.53203	0.43124
Ano5	71.4373	0.00235	0	0	0	0.0837	0.15057	0.04533
Ighv1-64	71.2451	0.02034	0	0	0	3.41185	3.19795	0.21882
Depdc1a	70.4853	0.00227	0.03485	0	0	0.52526	0.30118	0.18668
Hist1h2bn	69.8179	0.00217	1.21595	0	0.62498	8.58971	10.8163	3.489
Klrb1b	67.8962	0.00661	0.15595	0.25047	0.24938	1.99116	1.50613	0.75574
Igkv14-111	66.5494	0.01294	0	0	0	1.97626	3.03955	0.72245
Clec4n	64.6518	2.2E-16	0	0	0.11179	9.4749	9.3282	1.99708
Igkv6-32	63.8972	0.04308	0	0	0	1.78835	3.37786	0.48427
Gm26682	63.4825	0.00508	0	0	0	0.3365	0.44264	0.35879
Ankrd34b	63.2261	0.02586	0	0	0	0.07659	0.30998	0.05599
Olfr164	62.3982	0.01055	0	0	0	0.37839	0.24504	0.06148
Mir155hg	62.3258	0.00414	0	0	0	0.50751	0.53407	0.30921
4930554G24Rik	61.4312	0.00508	0	0	0	0.36295	0.44071	0.38699
Ighv1-53	60.3859	0.00956	0	0	0	2.69356	2.51587	1.2624
Mmp10	58.7998	0.00951	0	0	0	0.5068	0.44444	0.10293
Olfr111	57.2202	0.01883	0	0	0	0.22013	0.42321	0.10059
Ccl2	57.1894	0.02292	0	0.08173	0	2.10893	1.81063	0.38937
Izumo1r	54.5845	0.02064	0	0	0	0.28213	0.35526	0.0764
Fam167b	54.1818	0.01371	0	0	0	0.45501	0.73666	0.55446
Gm10561	53.4301	0.01845	0	0	0	0.33687	0.39389	0.18244
Ighg2c	52.9584	2.7E-05	0.17469	0.07014	0.05986	16.8927	23.1312	2.27232
Fpr1	52.5255	0.02162	0	0	0	0.60422	0.57804	0.06693
Gm10797	51.1321	0.02154	0	0	0	0.19638	0.34443	0.15953

Gdf3	50.1786	0.03787	0	0	0	0.27013	0.29156	0.07315
Svop	48.6404	0.04391	0	0	0	0.15963	0.12922	0.0389
Zeb2os	48.398	0.02154	0	0.13402	0.11437	1.30991	1.23002	1.08541
Dspp	47.536	0.02984	0	0	0	0.1313	0.10629	0.1
Ccr8	47.0662	0.02808	0	0	0	0.43326	0.50103	0.30169
Gm15922	46.4632	0.0424	0.03345	0	0	0.56719	0.54416	0.33278
Pcdhgb6	46.372	0.04751	0	0	0	0.13974	0.12569	0.03784
Ano3	45.2717	0.01709	0	0	0	0.0403	0.08972	0.06139
Ighv10-3	45.1723	0.01993	0	0	0	1.92908	1.40542	0.94027
Gm20707	44.609	0.02521	0	0.1109	0.12619	1.0406	1.28693	0.52834
Mmp8	41.7948	7.6E-08	0	0	0.03273	2.39922	1.6751	0.21927
Gm13749	41.3004	0.02681	0	0	0	0.30472	0.28778	0.30943
Tm4sf19	40.6864	0.04923	0	0	0	0.57857	0.23417	0.2115
Tnfsf9	40.2234	0.03388	0.18832	0.22685	0.12906	1.06429	0.67008	0.4323
Hoxa5	39.7012	0.04589	0.30852	0.94148	1.09947	4.14582	4.64196	2.73841
Gm10521	34.7905	2.2E-09	0	0.03613	0	1.38445	1.32655	0.44758
Cxcl10	30.9709	4.2E-07	0.14405	0.11568	0.04936	4.61323	3.00215	1.26758
Sprr1a	30.4095	1.1E-05	0	0.1154	0	5.14317	3.3599	0.76966
Ighv1-81	28.6761	1.4E-05	0.65994	0.52996	0.22613	60.2944	27.5068	13.129
A2m	27.99	9.9E-06	0	0	0.01612	0.85647	0.50205	0.05398
Il1rn	25.9207	4.2E-11	0.03715	0.02983	0.02546	2.49574	2.34126	0.68213
Atg9b	25.9026	2.7E-11	0	0.02357	0.02011	0.77408	1.05927	0.94325
Cthrc1	25.4107	1.6E-06	0	0	0.05363	2.11299	1.47178	0.47903
Igkv1-117	24.5911	1.2E-05	0.57336	0.23022	0.19647	24.6621	25.2098	6.36138
Timp1	24.5591	3.5E-10	0.13043	0.5237	0.98322	37.6735	26.2534	4.9899
Ighv1-26	24.4951	0.00906	0	0.26498	0.6784	23.8277	14.0889	0.25248
Atp6v0d2	24.1946	4.8E-16	0	0.03424	0.17535	6.29259	5.05045	1.72934
Fn1	23.8278	1.6E-26	1.44712	2.45158	2.67633	208.196	150.803	26.6355
Sh2d1b1	23.4182	3.4E-07	0.04553	0.07312	0	1.74383	2.08271	0.45286
Ighv5-9	22.2201	0.00701	0	0.22251	0	7.65539	6.05614	0
Igkv12-46	21.4509	0.02432	0	0.49341	0	10.6099	6.87092	0
Dcstamp	20.9814	1.8E-06	0	0.09334	0	2.18945	1.15202	1.15611
Igkv1-110	19.3575	0.00032	0	1.85553	0.19794	83.0639	13.5066	4.86198
Shcbp1	18.9567	2.1E-05	0	0	0.01261	0.3697	0.17769	0.15486
Ighv1-19	18.9398	0.00225	0	0	0.19598	5.02799	5.81445	0.87526
Ackr1	18.7463	3.3E-05	0	0	0.05649	0.93172	1.50845	0.63075
Thbs4	18.2603	2E-08	0.03521	0.02828	0.0724	3.80326	2.09424	0.40417
Frzb	18.2266	5.5E-05	0	0.02873	0	0.51674	0.58198	0.19164
Fst	18.181	2.6E-14	0.14868	0.19899	0.06793	7.31308	7.255	2.42692
Cxcl13	18.0738	8.2E-08	0	0.08088	0.20706	7.65206	5.4264	0.84767
Msr1	17.9226	2.1E-15	0.03651	0.02932	0.06256	2.22397	2.50556	0.71243
Gm26703	17.852	0.00019	0	0.10163	0.07228	0.51654	0.78266	0.29051
Gpnmb	17.8391	4.6E-17	0.11916	0.22965	0.21231	11.0137	9.61807	3.04519
Pilra	17.5322	1.4E-08	0	0	0.07706	1.64752	2.28627	0.48756
Tnfsf8	17.43	1.3E-06	0	0.02443	0.02085	0.99337	0.89691	0.16295
Phex	17.4232	4.4E-11	0	0.01478	0.05047	0.98263	1.39435	0.47895
Ereg	17.2394	1.2E-05	0.02806	0	0.01923	0.73995	0.95552	0.19321
Chl1	16.8075	9.9E-28	0.01884	0.05296	0.05811	2.34276	2.03054	0.76416
Ighg2b	16.7679	1.1E-05	0.12975	0.17365	0.02964	6.05599	6.81483	1.02585
Ighv9-3	16.7313	0.01122	0	0	0.22613	2.27917	8.38623	1.00992
Ccr1	16.6707	1E-08	0.04088	0.09849	0.05603	3.69664	3.5327	0.62563
Chodl	16.4126	0.0012	0.03032	0	0	0.38077	0.29282	0.32479
Gm10718	16.286	0.00623	0	0.95604	0.46621	2.13587	4.06307	8.58878
Adamts4	16.276	4.7E-11	0.04816	0.07735	0.0165	1.95045	1.48096	0.73697
Wfdc17	16.0206	7.7E-06	0	0.1745	0.29783	10.37	8.39441	1.33014
Saa3	15.9735	0.00014	0.17777	0.03569	0.12183	9.37683	3.75005	1.12221

C3ar1	15.6793	2.7E-13	0.10761	0.10802	0.11062	5.45658	5.62047	1.17337
Sirpb1c	15.53	3.2E-06	0	0.03267	0.05576	1.9925	1.94377	0.40466
Clec7a	15.5124	2.7E-17	0.30733	0.17629	0.33097	11.441	14.4166	4.03126
Pla1a	15.3929	1.1E-08	0	0.09433	0.04025	2.54468	1.6121	0.85385
Chrna7	15.353	3.4E-09	0	0.04129	0.01762	1.06547	0.81022	0.31475
Lama1	15.3156	3E-19	0.08518	0.08795	0.07505	2.10125	3.06171	2.72808
Gdf6	15.1452	0.00089	0	0.02633	0	0.4324	0.38336	0.17564
Ccl9	15.0773	4.8E-19	0.07607	0.2749	0.3128	10.891	10.4209	2.70664
Ccl6	15.0581	5E-09	1.29046	1.45083	0.9286	45.0133	32.9286	9.23234
Cd53	15.051	1.5E-12	0.13297	0.32035	0.50121	17.7849	14.4306	3.0015
Igkv10-96	14.7635	0.01736	0.32262	0	0.22109	6.48256	17.3826	0.24685
Ch25h	14.7247	4.8E-07	0.08579	0.20669	0.05879	5.17164	5.1894	1.18161
Syt13	14.4852	0.00036	0	0	0.0211	0.23204	0.42263	0.21207
Megf10	14.3789	7.8E-07	0	0.01205	0.01028	0.3298	0.32037	0.14927
Frmpd3	14.1909	0.00282	0	0.00907	0	0.05673	0.18369	0.06913
Tfec	13.7505	2.3E-06	0.0207	0.03325	0.01419	1.14407	0.77878	0.25347
Ms4a6d	13.5387	1.9E-06	0.06675	0.05361	0.13724	4.9043	3.46102	0.4597
Trac	13.472	0.00029	0.37161	0	0	4.58914	4.7223	1.04259
Selp	13.4249	3E-09	0.05249	0.02108	0.03597	1.648	1.37406	0.52212
Cers3	13.4185	0.00144	0	0	0.02438	0.42439	0.36162	0.16331
Cysltr1	13.1658	3.2E-09	0	0	0.06174	1.00419	0.98462	0.32746
Hist1h2bb	13.0861	7.6E-06	0.51247	0	0.87802	17.538	11.5919	2.94095
Emilin2	12.5727	3E-05	0.23697	0.45196	0.5075	3.58981	3.35762	1.67722
Wnt10a	12.5304	0.00207	0	0.03769	0	0.50095	0.47708	0.17954
Clec5a	12.4657	1.1E-09	0.03016	0.04844	0.04134	1.09847	1.54844	0.69235
A630033H20Rik	12.4506	0.01061	0	0	0.02291	0.25187	0.54369	0.05115
Igkv8-30	12.4289	0.0014	0	0.50548	0.43137	11.6599	8.7987	2.16735
Hist1h2bh	12.2817	0.00483	0	0.23971	0	6.93523	4.70364	0.91361
Tmem37	12.2085	3E-08	0	0.21283	0.18163	6.71234	4.53547	0.87877
Mest	12.1942	2.9E-12	0.02936	0.09431	0.14084	2.50717	2.55184	1.32538
Olr1	12.0981	5.3E-09	0.06485	0.07811	0.02222	1.28269	2.04369	0.89315
Sfrp1	12.0261	1.1E-24	0.26491	0.91477	0.41756	19.4291	19.9155	5.22967
Ighg1	12.0122	0.01975	0.04468	0.07177	0	0.89785	2.45297	0.06838
Tnc	11.992	9.3E-20	0.62589	1.28048	0.87829	32.9851	23.6334	7.76513
Trem2	11.8739	1.4E-23	0.07025	0.13163	0.08024	3.10255	2.89238	1.41549
Ctse	11.8587	0.01181	0	0	0.02504	0.34413	0.46428	0.05591
Clca3a1	11.8015	6.8E-15	0.05941	0.14313	0.1425	3.15228	4.59012	1.77287
Ephb1	11.692	0.00357	0.01654	0.01328	0.01134	0.21812	0.27746	0.12656
Cd200r4	11.6273	0.00147	0.05664	0	0.03881	1.2447	1.00758	0.30335
Slfn1	11.5486	4.6E-09	0.06213	0.14969	0.08516	4.01867	2.81092	1.09349
Ear2	11.4279	4E-05	0.48325	0.12936	0.22079	12.0367	12.7732	1.72557
Fam180a	11.4087	0.00851	0	0.05791	0	0.99625	0.5132	0.16554
Cd68	11.2752	8.7E-10	0.09214	0.51795	0.5683	20.1921	10.2568	2.32655
Iglc2	11.1793	0.00397	0.18925	0.30395	0	4.15918	8.46516	0.43441
Ccr5	11.1758	1.7E-14	0.09613	0.11028	0.24469	4.18215	5.2631	1.13481
Cybb	11.1348	1.2E-16	0.19941	0.56049	0.27332	12.3214	12.0317	2.8228
Tnfsf18	11.0591	0.00078	0	0	0.07684	0.80963	1.25379	0.25737
Prss22	10.9309	0.0028	0	0.0703	0	0.71462	0.53398	0.66985
Tnfrsf11b	10.8342	1.9E-06	0.0411	0.03301	0.05633	1.13554	1.58773	0.50317
Plau	10.6427	1.4E-12	0	0.15879	0.30489	2.91774	5.05042	1.929
Hist1h2bk	10.337	0.00169	0.72995	0.78159	0.50025	10.695	11.9969	1.30324
Fgf23	10.2358	0.01622	0	0	0.04376	0.52119	0.64908	0.09771
Arg1	10.2209	0.0002	0.31387	0.06301	0.10755	4.18816	5.94297	1.44098
Fcgr2b	10.2011	5.5E-26	0.24004	0.40159	0.4798	11.7191	11.6421	3.64278
Ticam2	10.0942	0.00058	0	0.07104	0	0.75911	0.92923	0.47379
Fam162b	10.0404	0.00969	0	0	0.13119	1.68292	0.97308	1.17184

H2-Eb2	9.98554	6.2E-05	0.05243	0.0421	0.07186	1.54737	1.65234	0.64189
Gm14548	9.93778	0.02314	0.10885	0.13112	0	0.88857	0.38731	0.41645
Tlr1	9.91766	1.1E-07	0.03461	0.08339	0.11861	2.02138	3.02626	0.82105
Ctss	9.81921	2.6E-19	5.03191	5.54764	4.67587	176.567	157.062	47.2469
Lox	9.7991	1.3E-14	0.28895	1.12152	1.28713	30.1489	25.2988	5.56412
Clec4a2	9.75909	6.8E-06	0.11875	0.03179	0.02713	1.6653	1.69009	0.39373
Lilrb4a	9.71934	8.9E-12	0.47502	0.27743	0.35514	11.2262	10.6899	3.83295
Rdh12	9.71538	0.04487	0	0.03135	0	0.29414	0.33731	0.11947
Ddias	9.65983	0.00023	0.03129	0	0.04288	0.7858	0.71562	0.19151
Clec4a1	9.64593	0.0002	0.07434	0.0597	0.10189	2.33396	2.87177	0.2844
Trem1	9.62663	0.00155	0	0.03104	0.02649	0.77678	0.49125	0.1479
Tdo2	9.61753	0.01438	0	0	0.02045	0.24361	0.22754	0.09134
Kynu	9.5854	3.1E-07	0.01884	0.04538	0.01291	0.55588	0.67019	0.37472
Fcer1g	9.50448	2.4E-09	0.92761	0.53209	0.63571	24.7968	20.4096	3.54888
Gm16638	9.47265	0.03903	0.03738	0.06003	0.01708	0.34423	0.18999	0.27646
Itm2a	9.44743	2.3E-07	0.13282	0.15999	0.13654	3.21096	4.45586	1.47362
Olfr99	9.27373	0.02073	0	0	0.04464	0.57265	0.46355	0.14953
Nr5a2	9.2472	0.00124	0	0.03153	0	0.09863	0.36926	0.18028
Wisp1	9.23316	5.4E-09	0.14693	0.10113	0.10069	3.2027	3.97952	0.62635
RP23-181A8.7	9.19267	0.00377	0.03937	0	0.02698	0.59328	0.52028	0.24098
Stra6l	9.16262	0.00071	0	0.01944	0.0083	0.25846	0.28306	0.07411
Susd3	9.11501	0.00072	0.04082	0.06557	0	0.61524	1.18282	0.2499
Sgol2a	8.88151	0.00576	0.0469	0.07533	0.03214	0.75097	0.88206	0.41269
Has2	8.80555	1.4E-06	0	0.08781	0.01873	0.75524	1.18104	0.35557
Gpr141	8.75873	0.00054	0.03374	0.05419	0	0.74146	0.68595	0.20651
Pou2af1	8.71084	5.1E-10	0.09087	0.25542	0.12456	4.96444	3.3489	1.18207
Ccr6	8.70237	0.02061	0.17133	0	0	1.18341	1.48049	0.43699
Pif1	8.61827	0.00614	0.02639	0	0.01809	0.28176	0.37566	0.18177
Gdf15	8.57064	5.3E-07	0	0.15066	0.12857	2.55249	2.03443	1.10058
Dusp19	8.56377	0.02872	0.19355	0.24869	0.31834	1.4098	0.72802	0.50353
Eda2r	8.42777	1.8E-10	0.01966	0.06315	0.09432	1.40737	1.71887	0.43626
Hist1h2ag	8.41351	0.00618	0.4776	0.19177	0.49097	9.14701	8.98243	1.09634
Shc4	8.36245	0.00037	0.04205	0.02251	0.00961	0.39606	0.62697	0.15015
Nuf2	8.21482	9E-05	0.01827	0.02934	0.01252	0.4473	0.43636	0.20964
Mis18bp1	8.16594	2.9E-06	0	0.01164	0.03974	0.49152	0.42735	0.14419
Mgp	8.14413	8.5E-09	7.31798	17.16	12.5713	442.117	266.731	40.9879
Bmper	8.12732	1.1E-12	0.21568	0.07698	0.18065	4.01777	3.08182	1.63195
Serpina3n	8.12265	1.5E-12	1.6273	2.97411	3.73022	100.104	53.0241	13.6967
Tlr8	8.07745	5.8E-07	0.0323	0.07781	0.15494	2.73788	2.18346	0.39541
Col24a1	8.02959	1.3E-06	0.0152	0.03661	0.04166	0.50577	0.64889	0.50002
Ildr2	8.02573	4.8E-12	0.08422	0.2029	0.13468	2.25644	3.34634	1.00961
Ccr2	8.01238	2.3E-15	0.27413	0.37148	0.58708	7.12201	12.2184	3.17251
Ighv3-6	8.00599	0.01549	0	0.53148	0.22678	9.97388	4.87791	0.7596
E230029C05Rik	7.96529	0.0002	0.10339	0.02076	0.01771	1.02248	0.6569	0.33621
Col12a1	7.93775	9.4E-14	0.07606	0.42754	0.31274	7.48371	5.84538	1.43744
Ifi206	7.93367	0.0001	0.03792	0.09136	0.10396	2.00033	1.85057	0.46429
Apoe	7.93123	8.3E-11	3.4711	11.3822	10.3478	280.152	149.59	37.6261
5430427O19Rik	7.84648	1.2E-05	0	0.02478	0.06343	0.79429	0.65866	0.28328
Lhfpl2	7.84083	1.3E-12	0.05598	0.10115	0.14386	2.1969	2.58931	0.74957
Chst5	7.78198	0.02036	0.10616	0	0	0.43329	0.78243	0.32491
Lipa	7.77861	5.6E-10	0.39009	0.75184	0.72182	14.3297	15.4664	4.38775
Cd52	7.71405	5E-06	0.68301	0.47014	0.53495	16.7877	11.9032	2.09046
Asxl3	7.71334	0.04361	0.00746	0.00599	0	0.07023	0.12507	0.02282
Abcg3	7.696	0.00106	0	0.0524	0.04472	0.71702	0.84576	0.24964
I830077J02Rik	7.68578	0.00187	0.08085	0.03246	0.0277	0.86307	1.15071	0.30932
Zwilch	7.68472	0.00117	0.05408	0.08686	0.11119	0.57733	0.61854	0.39313

Btla	7.67915	7.3E-13	0.21336	0.22845	0.21933	4.6668	4.89841	1.90465
Cd300lf	7.56601	2.2E-05	0.26992	0.23482	0.12332	3.98306	4.07034	2.09977
Ms4a14	7.54719	2.7E-05	0	0.06864	0.05858	0.93032	1.12963	0.26161
Edn1	7.52939	1.8E-06	0.19764	0.39679	0.77883	17.9644	4.84734	2.38187
Pappa	7.52693	2.1E-10	0.02101	0.09278	0.06478	1.60925	1.05175	0.45809
Dhrs9	7.46868	0.0149	0.02705	0.02173	0.01854	0.52663	0.53632	0.1035
Jchain	7.39094	0.00026	0.64421	2.18018	1.00911	28.3161	30.4765	7.00655
Cd79b	7.38438	0.00304	0.24281	0	0.27733	4.6248	4.85452	0.37157
Mettl21e	7.37766	0.00458	0	0.04696	0.02004	0.25705	0.54992	0.31323
Cdc25c	7.31391	0.04885	0	0	0.04089	0.29975	0.36396	0.18263
A530099J19Rik	7.30361	0.00058	0	0.03354	0.05725	0.73434	0.7006	0.22371
Tlr13	7.27354	1.2E-09	0.05705	0.13745	0.2346	3.67214	3.01607	0.82945
Tmem200a	7.25159	7.9E-06	0.02815	0.06781	0.05787	0.84833	1.11591	0.45226
Nxpe4	7.22195	2.2E-09	0.05878	0.11014	0.08057	1.21805	1.94213	0.62968
1810011H11Rik	7.21541	0.00025	0	0.06314	0.10777	1.38244	1.03914	0.6618
Fmod	7.21384	4.9E-05	0	0.22507	0.28811	3.56372	4.62993	0.56293
Nsg2	7.20294	0.00371	0.02786	0.02237	0.01909	0.41988	0.43902	0.2345
Fcgr3	7.20035	1.3E-09	0.80059	0.48219	0.91444	19.9411	13.2596	3.9818
F730311O21Rik	7.1958	2.5E-05	0.04253	0.03415	0.05829	0.8278	1.14565	0.27658
Fcgr1	7.17533	0.00013	0.13421	0.03592	0.18395	3.48322	2.36486	0.44499
Cd86	7.12845	3.4E-09	0.09491	0.02541	0.21681	2.72152	2.07441	0.89565
Trbc2	7.10069	0.00022	0.16737	0	0.91761	5.88536	9.01786	1.92097
Srpx2	7.07272	5.9E-06	0	0.15915	0.08149	2.16536	1.57151	0.36394
Igf1	7.06102	5.5E-16	0.2105	0.4226	0.43277	6.38117	8.54181	2.50543
Wnt7a	7.04466	3E-07	0	0.05864	0.10009	1.23809	1.07646	0.41908
Mxd3	7.04257	0.02101	0.05566	0.04469	0	0.7339	0.48093	0.25551
Ackr3	6.9236	3E-10	0.27675	0.52783	0.26078	8.99303	6.43576	2.0117
Ly6d	6.90516	0.02279	0.15567	0.12501	0.10668	3.22576	2.37384	0.59557
Hrh2	6.90369	0.00018	0	0	0.05349	0.3186	0.55548	0.23891
Olfr550	6.87984	0.00584	0	0	0.03202	0.2347	0.26123	0.08937
Cd180	6.84494	3.3E-06	0.1798	0.12376	0.10562	1.7742	2.29793	0.74686
Ntm	6.83198	0.02924	0.01434	0	0.00982	0.17104	0.1093	0.08775
Cmah	6.81239	1.4E-20	0.20906	0.27981	0.32634	3.63194	5.71475	2.9238
Hist1h2ak	6.80881	0.00265	1.47353	0.23666	0.20197	12.9538	11.235	2.48047
Cxcl9	6.79593	0.00383	0.0384	0.09252	0.07895	2.36312	0.97598	0.20568
Gcnt4	6.77274	0.00044	0	0.05479	0	0.29987	0.3699	0.1566
Slc27a6	6.75714	2.8E-06	0	0.14971	0.1597	1.60963	2.5349	0.78457
Ulbp1	6.75652	0.01706	0.06367	0	0.08727	0.95955	1.22986	0.19488
Ly6c2	6.74214	0.00111	0.12681	0.16972	0.26071	3.07892	1.86927	0.67921
Clec4a3	6.73334	0.00013	0.08728	0.07009	0.23926	2.84986	2.9724	0.60104
Zfp991	6.68507	0.03503	0.15882	0.06377	0	2.06934	2.28058	0.91142
S100a4	6.65803	4.5E-09	1.18992	2.22966	0.95139	29.5139	26.7139	7.13213
Nrcam	6.64436	5.8E-11	0.12222	0.28218	0.18846	4.27858	4.06143	0.98195
Havcr2	6.61398	1.2E-08	0.12769	0.06836	0.32088	3.31424	3.24539	1.43304
Mpeg1	6.6092	2E-14	1.25571	2.14553	2.65492	44.5081	40.171	11.1415
Tnip3	6.55026	7.2E-05	0.07641	0.02045	0.06982	0.71974	1.29473	0.40929
Rasgrp1	6.5223	2.8E-09	0.14477	0.09965	0.15591	2.28568	2.90152	0.85456
Ptger3	6.4755	0.00062	0	0.01635	0.05582	0.33243	0.58996	0.21812
Kcnj10	6.47493	0.02249	0	0.0172	0.01468	0.20176	0.25042	0.04917
Ms4a6c	6.47227	5.2E-09	0.16949	0.40833	0.46462	9.04643	6.86354	1.77241
Gm4070	6.47179	0.00069	0.03746	1.77494	0.53058	13.5181	5.82683	2.39825
Ms4a7	6.45773	8.1E-07	0.10404	0.10443	0.12477	2.23748	2.31361	0.51743
Cd8a	6.43705	4.8E-05	0.12738	0.07672	0.10912	2.01962	1.52156	0.73099
Ctsd	6.42998	3.2E-09	5.41902	16.0619	13.5721	196.432	130.463	45.0076
Slc28a2	6.41782	1.2E-05	0.08257	0.08841	0.03772	1.1061	1.52495	0.5265
Tgfbi	6.41458	3.4E-19	1.21095	1.7018	2.34147	35.4126	35.0854	10.0931

Gtse1	6.41101	9.2E-06	0.08513	0.06836	0.08751	1.36312	1.34143	0.68395
Mmp19	6.38787	5.3E-13	0.03396	0.79098	0.48881	9.25607	7.56177	2.80675
Mt2	6.36495	2.1E-08	1.57577	3.63809	2.69976	80.3946	30.2366	12.2079
Fscn1	6.36429	1.3E-15	0.42298	0.64847	0.94868	12.6764	11.8251	5.14894
Scarb1	6.33923	4.4E-16	0.3876	0.77075	0.59451	11.4276	10.8548	3.7143
Prr11	6.32145	0.00064	0	0.09729	0.04151	0.98895	0.89292	0.20857
Kif20a	6.31613	0.00285	0.12204	0.07351	0.08364	1.51353	1.14765	0.35019
Calcrl	6.28613	1.6E-08	1.3997	2.97201	3.64186	52.9284	49.4172	14.7398
C1qc	6.27537	2.1E-10	0.8397	1.20415	1.10982	27.6443	17.8047	3.71738
Igsf6	6.25661	0.00994	0.3195	0.21381	0.18247	3.67811	3.43754	1.34459
Efna2	6.24653	0.04382	0	0.04365	0.03725	0.64843	0.49727	0.12476
Atp1a3	6.244	8.5E-08	0.0972	0.10927	0.09325	1.84318	1.80823	0.62472
Ltbp2	6.20155	1.5E-29	1.13215	2.898	2.79318	38.1137	38.4994	16.0479
Icos	6.17758	3.7E-05	0	0.10846	0.04628	0.99654	0.92684	0.31004
Adamts12	6.17536	3.5E-09	0.01858	0.32823	0.16552	3.03318	3.22971	0.9098
Nipal1	6.11603	0.0049	0	0.04234	0.01807	0.41388	0.28142	0.18156
Ms4a4b	6.1032	0.00016	0.08784	0.05291	0.1204	1.8478	1.87531	0.33607
Milr1	6.09884	0.00809	0.14044	0.07519	0.16041	0.97007	1.07082	0.32239
Rnf152	6.09721	2.3E-07	0	0.06541	0.06512	0.65634	0.80731	0.20774
Cr2	6.07596	0.00099	0.07139	0.0344	0.02935	0.80687	0.78379	0.29496
Hist1h2bj	6.07509	0.01549	0.75045	0.60265	0.34286	16.3359	8.51918	2.29686
Ndnf	6.04449	2E-14	0.48096	1.28744	1.25565	15.7188	18.8302	6.52781
Emp3	6.03983	1.9E-05	0.21628	1.04211	0.37056	13.5131	8.52013	1.48942
Brinp1	6.03682	2.6E-08	0.07495	0.06019	0.12841	1.80021	1.30487	0.58784
B2m	6.02054	1.4E-05	11.5819	14.0594	8.30647	277.882	140.675	26.277
Fbln5	5.99337	1.2E-06	0.05961	0.38295	0.36766	5.2277	5.09026	0.92741
Pik3cg	5.92708	3.5E-12	0.18774	0.20559	0.19884	3.71883	3.40944	1.08392
Ly86	5.92603	6.6E-05	0.46467	0.65302	0.47767	10.7229	10.6287	1.51108
Ccl7	5.91333	0.0379	0.14459	0.11612	0.09909	1.6343	2.35191	0.66382
Scube1	5.8788	1.4E-19	0.11102	0.24517	0.26629	3.62498	3.407	1.36976
H19	5.8355	0.0066	0.04883	0.11763	0.03346	1.16509	0.94313	0.48569
Hist1h2bm	5.80251	0.03926	0.30399	0.24412	0.20833	6.29912	4.63554	0.6978
Epha3	5.78047	1E-05	0.02047	0.09861	0.11221	1.3494	1.55007	0.29754
Svep1	5.77328	3.5E-12	0.17965	0.54823	0.62545	8.58708	6.56764	2.35889
Lamc2	5.74524	4.3E-12	0.24492	1.11069	1.0071	13.9138	12.7937	3.71503
Angptl4	5.72635	6.8E-07	0.17623	0.36796	0.72465	10.7562	5.60765	1.34846
Gm32200	5.71166	0.02535	0	0.039	0.03328	0.24395	0.49368	0.2601
B430203G13Rik	5.70092	0.02432	0	0.06764	0.17318	0.89916	0.81349	0.38671
Csmd1	5.69422	2.3E-11	0.04869	0.02932	0.05005	0.53503	0.60944	0.43309
Samsn1	5.69341	3.9E-05	0.10218	0.10257	0.05252	1.70026	1.35038	0.29319
Slc29a3	5.68193	2.4E-10	0.07941	0.30291	0.36734	4.69965	4.20798	1.00256
Pla2g7	5.66806	9.6E-08	0.11961	0.25613	0.40984	4.48124	3.62753	1.03721
Col28a1	5.66035	1.4E-08	0.04723	0.15173	0.19422	1.69062	2.04081	1.22883
Cd300a	5.64736	9.2E-10	0.12222	0.15704	0.18428	2.50198	2.13716	1.04744
Espl1	5.62755	2E-11	0.01747	0.07014	0.13169	1.12983	1.0833	0.66833
Cd84	5.59801	3.6E-13	0.29546	0.26693	0.55683	5.49621	6.99285	2.37377
Cd200r1	5.58964	6.9E-06	0.11388	0.22863	0.11707	2.28834	2.54704	0.82782
P4ha3	5.57688	3.5E-08	0.07389	0.14834	0.22786	2.29662	2.36612	1.10246
Bcl11a	5.5706	4.1E-05	0.15823	0.11295	0.07229	1.24752	1.31371	0.41031
Gcsam	5.53857	0.03675	0	0.02716	0.04636	0.531	0.46423	0.05176
Kcnt1	5.50859	0.03381	0.02439	0.00979	0.01671	0.18375	0.24171	0.17726
Slco5a1	5.50707	1.9E-05	0.00882	0.0354	0.01208	0.19376	0.29577	0.18214
Thbs2	5.49474	0.00028	0.01956	0.14135	0.08042	1.52281	1.29235	0.29929
Nlrc4	5.48568	0.00065	0.06035	0.09693	0	0.54952	0.76695	0.43872
Clec2i	5.45768	0.00117	0.14896	0.05981	0.05104	1.00554	1.49545	0.54141
Mctp1	5.41662	3E-13	0.18377	0.2384	0.21313	2.87603	3.39159	1.50351

Tmem88	5.41448	0.00047	0.07321	0.23517	0.15052	2.02273	2.90263	0.78425
Rad54b	5.40532	0.04635	0	0.01609	0.04118	0.16351	0.19345	0.09196
Nlrp1a	5.40431	0.00711	0.0278	0.04465	0.01905	0.3666	0.46634	0.234
Kcnk13	5.39769	5.2E-06	0.11165	0.11955	0.12753	2.0799	1.58907	0.68345
Mfap4	5.39363	5.9E-07	2.04883	5.08112	4.95565	90.2834	52.1939	13.4176
Tlr7	5.39113	1.5E-05	0.08512	0.13671	0.175	2.7259	1.87488	0.52104
Lcn2	5.36518	0.00016	1.98699	0.85101	1.05911	38.7337	17.3494	5.43954
Ctla4	5.35566	0.00613	0	0.07984	0.06813	0.37456	1.08647	0.38035
Cnrip1	5.3447	0.00047	0.05787	0.11617	0.07931	0.79939	0.79417	0.26566
Txk	5.34245	0.0028	0.03388	0.0136	0.02322	0.2872	0.33582	0.18147
Kntc1	5.33146	5.6E-06	0.04352	0.05825	0.03977	0.51928	0.58259	0.49955
Lair1	5.33145	7.5E-06	0.06198	0.06221	0.14866	1.37183	1.77992	0.39123
C1qa	5.31258	5E-11	3.00684	3.66669	3.89233	67.8312	52.588	16.9572
Hgf	5.31116	2.8E-07	0.08059	0.11649	0.20986	2.33785	2.03993	0.72761
Serpine1	5.3098	6.6E-09	0.56774	1.91968	0.8396	23.2664	13.3964	4.66424
Hbegf	5.30452	3.8E-17	0.39046	0.90147	0.66897	8.45868	9.82429	4.78021
Tnfaip8l2	5.29745	0.0002	0.10323	0.58027	0.14148	5.31511	3.83031	0.94781
Gpm6a	5.29455	0.00013	0.03602	0.11572	0.19751	1.74181	2.17906	0.49616
Slamf1	5.29072	0.00965	0	0.01511	0.02579	0.20084	0.22952	0.10077
Ptafr	5.28977	4.5E-09	0.12738	0.23016	0.28371	3.25938	3.3021	1.24269
Birc5	5.28142	0.00054	0.10213	0.02734	0.11666	1.3254	1.12482	0.54704
Mex3b	5.27989	0.00014	0.10356	0.05544	0.07097	1.21391	0.94756	0.44905
Esm1	5.27666	0.02846	0.11046	0.6653	0.60561	0.86703	1.23526	0.97199
Rbm46	5.25739	0.00585	0	0.03722	0.06352	0.43653	0.51828	0.35462
Vps25	5.2569	0.01136	0.14107	0.56644	0.43505	9.38974	6.20264	1.40325
Cd3e	5.21733	5.8E-06	0.17032	0.20517	0.13618	2.06771	2.02009	0.58646
C1qtnf6	5.21341	3.5E-14	0.28931	0.69698	0.62785	7.32712	8.60276	3.17293
Cers6	5.20824	1E-16	0.17469	0.6079	0.64847	8.05327	6.37107	2.56193
Slamf6	5.20746	0.00072	0.07861	0.01263	0.05387	0.76014	0.7272	0.26465
Hist1h2ab	5.18261	0.0038	0.97944	0	1.34246	10.7629	8.71246	1.87358
Cdkn2b	5.1666	4.6E-08	0.49886	0.20031	0.45584	4.95979	5.53636	2.48112
Scel	5.14572	2E-07	0.34143	0.33511	0.38998	5.62181	5.18716	2.00289
Ly9	5.13629	0.00071	0.17511	0.28125	0.16801	2.74895	2.36767	0.53596
Galns	5.12962	3.4E-05	0.08741	0.56156	0.20966	3.54026	3.11018	1.03669
Ly6c1	5.11401	1.5E-09	1.51939	2.99491	3.21846	54.2086	32.051	9.19498
Robo2	5.10329	2.1E-12	0.03506	0.21399	0.20665	2.17087	2.14583	0.86925
St8sia4	5.09083	2.3E-06	0.08226	0.07707	0.14563	1.43335	1.6899	0.535
Cd33	5.08303	1E-07	0.05962	0.21544	0.26556	3.13515	2.43182	0.82109
Colec12	5.07836	1.6E-06	0.41837	1.45588	1.33801	19.5717	13.3621	2.93446
Cd300c2	5.06424	6.5E-11	0.18168	0.87538	0.74704	8.78419	8.49594	3.68384
Haao	5.06289	0.00268	0	0.22834	0.06495	1.42834	1.39711	0.58017
Kif15	5.05763	4E-05	0.04811	0.07727	0.04945	0.7552	0.63579	0.52455
Parpbp	5.04361	0.03519	0	0.07764	0.02208	0.50589	0.39313	0.17261
Slfn10-ps	5.04151	0.00055	0.09596	0.10274	0.08768	1.00423	0.95924	0.48948
Lpar5	5.03378	0.04573	0	0.06139	0.10478	0.67206	0.89376	0.46796
Itgam_1	5.01565	7.7E-09	0.66854	0.70349	0.55296	6.54304	5.27311	2.593
F2r	4.99654	2.7E-10	1.15574	3.37497	3.12019	39.8268	37.046	10.7995
Nusap1	4.99163	0.0004	0.05707	0.06874	0.09777	1.30796	1.08779	0.24016
Gpr65	4.98897	0.00046	0.28262	0.13618	0.11621	2.52004	2.58586	0.73526
Mmp14	4.98776	1.9E-23	0.97327	1.8237	2.12452	26.234	22.3172	8.5228
Cd302	4.97025	0.00049	0	0.61823	0.64483	8.91621	5.7393	0.58906
Fbxo5	4.96171	0.00206	0	0.09287	0.11888	1.19819	0.99931	0.35395
Etv4	4.94253	0.00093	0	0.05993	0.15343	0.49203	0.85348	0.97071
Pcdh18	4.94126	5.6E-07	0.21584	0.24266	0.19229	2.92748	3.51078	1.07349
Hist1h2ap	4.93161	0.02413	1.92231	1.15778	0.82337	28.8189	19.5424	4.78036
Ccdc80	4.91425	1.8E-08	0.27976	1.30302	0.76689	13.948	10.5229	2.35465

Gyg	4.89009	1.4E-05	0.69175	1.07135	1.11745	16.8784	15.195	2.75994
Xlr4b	4.88255	0.03661	0	0.07266	0.06201	0.56817	0.73589	0.48464
Csf2rb	4.88196	1.7E-14	0.63209	0.99568	0.6831	11.9226	11.2083	4.31569
Syt15	4.84097	0.00021	0	0.28812	0.18441	0.9434	2.35941	1.33832
Cxcr5	4.83989	0.00311	0.06952	0.16748	0.04764	1.24412	1.50182	0.37236
Gda	4.83176	3.3E-14	0.4342	0.66705	0.72451	9.06855	8.93386	2.83124
Hexb	4.83146	1E-06	0.60829	1.90511	1.08387	21.5429	15.3671	2.79266
Aif1l	4.82581	0.00073	0	0.0692	0.03937	0.54112	0.48183	0.19781
Ap1s2	4.82294	3.2E-06	0.20994	0.14986	0.43164	4.39433	3.50975	1.12449
Fam111a	4.80844	0.00615	0.2527	0.40586	0.3656	4.65448	3.82485	1.89057
Nlrc3	4.80596	1.1E-10	0.1147	0.15791	0.13476	1.58458	1.59089	1.07829
Abcg1	4.80527	5.5E-12	0.65515	1.09799	0.89797	14.0945	12.2492	3.94499
Cxcl14	4.79539	4.2E-07	0.88945	2.44894	2.83008	33.6304	29.5164	6.36824
Ckap2	4.78537	5.8E-06	0.07205	0.05786	0.19751	1.42512	1.50154	0.68912
Lgals1	4.78108	0.00035	1.88206	5.11547	2.38119	70.999	32.4528	7.08964
Bst1	4.77631	0.0001	0.10475	0.2804	0.14357	3.22302	2.21855	0.61449
Slc15a1	4.7742	0.0127	0	0.02978	0.05083	0.51232	0.28276	0.17026
Tnfrsf13b	4.7708	1.7E-05	0.12252	0.09839	0.27988	2.20538	2.55331	0.81246
Ms4a6b	4.76396	5.1E-07	0.23241	0.20738	0.28316	4.08629	3.18968	0.96821
Prcp	4.75307	4.8E-09	0.19023	0.48376	0.9995	8.26208	7.42942	2.57152
Adgrl4	4.73006	7.1E-09	0.2526	0.93313	1.03869	12.2453	8.97515	2.91858
Tk1	4.72586	0.01342	0.2451	0.76941	0.53445	2.40649	2.43504	1.31277
Acp5	4.67989	0.00325	0.62759	0.50399	0.2346	6.44863	5.10412	1.1787
Tlr9	4.67558	1.7E-05	0.0999	0.16045	0.3195	3.26202	2.45438	0.76441
Pglyrp2	4.67035	0.00995	0.05175	0.08312	0.07093	0.61743	1.10482	0.39598
Plbd1	4.66958	2.1E-05	0.21994	0.52986	0.87422	9.4464	7.37848	1.41362
Ube2l3	4.6284	0.03208	1.03428	1.46183	1.46016	6.61148	6.08796	1.96266
Pld4	4.6259	4.2E-11	0.52698	1.0815	0.96307	13.1996	11.0123	3.53945
Igfbp2	4.61676	3.2E-07	0.31353	2.92068	3.309	31.894	23.1087	10.076
Gjb3	4.59274	0.02769	0.05891	0.04731	0.12112	1.14675	1.19779	0.13523
Cldn4	4.59248	3.3E-10	0.89288	1.12676	1.61718	9.25097	16.8574	10.1992
Myct1	4.57076	0.00075	0.12213	0.16346	0.22319	2.40291	2.3383	0.71644
Neil3	4.56865	0.00334	0.02085	0.05023	0.04287	0.47131	0.4875	0.2393
Eln	4.56417	1.9E-14	9.5443	16.3959	17.8299	154.256	181.86	108.543
Hbb-bs	4.56256	0.04329	5.12741	5.44972	2.2737	12.2157	12.0349	58.965
Phf11c	4.55951	0.00675	0.06697	0.05378	0.04589	0.77793	0.71483	0.23058
Thbs1	4.55573	2.5E-09	4.34823	11.9917	7.56742	138.591	86.4894	29.4332
Lrg1	4.55315	4.4E-14	1.97176	2.82262	4.05384	44.8416	35.3838	14.3656
Gpr35	4.55223	4.9E-08	0.14361	0.26909	0.21324	2.94572	2.48187	1.0622
Smarca1	4.54305	0.00178	0	0.0304	0.03892	0.33284	0.30792	0.11588
Spry4	4.53775	3.7E-12	0.45449	0.75023	0.91711	8.38722	8.98407	4.40495
Dpep2	4.53569	4.1E-06	0	0.08195	0.36714	1.71403	1.99696	0.93696
Enox1	4.53555	0.00376	0	0	0.11355	0.33294	0.47164	0.45641
Slfn8	4.53285	6.2E-08	0.14155	0.40923	0.34923	3.7332	3.29541	1.68966
Ccl17	4.50454	0.00628	1.12665	0.18095	1.23538	17.1204	8.0175	3.10344
Mthfsl	4.50311	0.02234	0.10963	0.13205	0.03756	1.03255	0.80798	0.54523
Lrrc8c	4.49716	7.5E-16	0.36316	0.99643	0.95405	11.3071	8.97607	3.54297
Igha	4.48119	7.6E-06	7.59684	22.9733	11.9595	196.644	183.278	58.6264
Cd93	4.48092	3.6E-07	1.00607	2.52128	3.28096	35.5517	24.5731	9.35712
Tifab	4.47461	1.9E-06	0.36421	0.18612	0.22691	3.26416	3.39966	1.2414
Tmem26	4.4694	0.00408	0	0.09205	0.03142	0.3311	0.69918	0.2105
C1qb	4.46694	4.4E-08	4.79032	3.67589	4.96081	71.8573	60.6028	20.4446
Sh2b2	4.4642	6.9E-06	0.10469	0.30825	0.23915	2.43224	2.19947	1.70886
AI506816	4.46313	3.7E-08	1.34419	1.50621	1.00689	11.6401	7.21392	2.96598
Fbn1	4.46204	2.7E-16	1.48021	3.68875	3.65843	39.1035	36.547	12.634
Vstm4	4.46191	4.8E-09	0.12676	0.40719	0.46332	4.13911	4.91847	1.29326

Ticrr	4.45817	0.00012	0	0.00996	0.06801	0.31159	0.30268	0.15188
Cd79a	4.44765	0.0228	0.87441	0.19151	0.21791	7.73683	6.70737	1.09483
Il4i1	4.44384	0.00394	0.28387	0.09118	0.29181	1.2121	1.64493	1.02087
Gria3	4.44224	7.7E-10	0.08223	0.33019	0.24152	2.87689	2.59156	1.07866
Fkbp7	4.43532	0.00382	0.15599	0.37579	0.1069	3.72202	2.10113	0.65645
Hsd11b2	4.42424	0.00403	0.06295	0.1011	0.12941	1.18575	1.31181	0.43347
Dab2	4.41276	2.2E-08	0.36282	0.78542	0.63784	9.74702	7.12839	1.99161
Foxred2	4.40945	0.00836	0.039	0.04698	0.04009	0.62452	0.36677	0.25367
Skap1	4.40867	6.2E-05	0.03104	0.06232	0.07446	0.61401	0.8284	0.30878
Bgn	4.40376	1.1E-09	5.13596	15.0098	13.6558	175.702	126.021	39.9203
Cdh5	4.39974	2.1E-06	1.7636	4.11601	4.40017	60.164	37.4266	10.0365
Cdh11	4.38775	1.4E-08	0.23414	0.63719	0.64183	6.80379	6.69774	1.69199
Aspm	4.38728	2.1E-06	0.02998	0.05617	0.11642	0.86594	0.78224	0.39761
Sparc	4.36424	9.9E-15	7.42979	22.8102	22.9025	235.343	209.966	72.5481
Pkib	4.35628	0.00088	0.02945	0.04729	0.02691	0.30816	0.50889	0.12016
Slc40a1	4.35594	2.6E-05	0.01713	0.13757	0.10566	0.83902	1.34966	0.39323
Itgax	4.35241	6.6E-09	1.15747	0.81332	0.97502	13.0371	13.8874	5.81209
Slamf7	4.34534	2.4E-06	0.17713	0.42674	0.29481	4.60795	2.82973	1.49089
Lgmn	4.33565	6.5E-18	2.04416	3.84366	4.27104	48.6511	39.3067	14.9545
Il9r	4.3265	0.03315	0.09365	0.0376	0.04813	0.57334	0.69022	0.42994
Lgals3	4.32641	5.8E-09	0.93503	2.95345	2.13598	33.5449	21.7044	6.10518
Psrc1	4.32467	4.3E-06	0.11536	0.40143	0.47434	2.63186	3.1273	3.17763
Ctsw	4.32419	0.00837	0.09401	0.45296	0.12885	2.95155	2.2459	1.29478
Ajap1	4.3193	0.03347	0	0.06103	0.02604	0.3579	0.27041	0.26167
Tmem252	4.31492	3.4E-06	0.51747	0.75555	0.70926	11.0478	6.16929	3.02361
Bank1	4.3105	0.00417	0.29396	0.17394	0.13784	1.90413	2.36712	0.50904
Lhx6	4.30056	4.8E-06	0.0241	0.15485	0.11563	0.75678	1.10269	0.90373
Dsn1	4.29592	0.00213	0.06906	0.08319	0.071	0.60714	0.87763	0.58129
Ifi27l2a	4.29386	0.00025	1.0006	1.2053	2.22861	28.1166	12.4609	5.35934
Pgap1	4.29008	3.8E-08	0.04364	0.11388	0.14952	1.3015	1.33635	0.4257
Fgr	4.28663	7.8E-10	0.17989	0.33708	0.45203	3.61466	4.11474	1.72056
Gins3	4.28104	0.00507	0.04275	0.03433	0.1172	0.61746	0.86927	0.42527
Palb2	4.28104	0.00051	0.02135	0.0343	0.07317	0.34861	0.55355	0.35945
Il20rb	4.27627	0.00369	0.02754	0.06634	0.07549	0.44957	0.64386	0.50568
Cpxm1	4.27464	1.6E-08	0.14287	0.95609	1.27284	12.5895	7.72202	3.06091
F7	4.27317	1.5E-06	0.43611	0.30019	0.46966	5.28136	4.40189	1.95451
Slc11a1	4.26992	6E-07	0.12504	0.22593	0.25708	2.94438	2.25634	0.81325
Ptprc	4.25704	2.5E-10	0.68439	0.82441	0.74374	9.90884	10.4165	3.38144
Syn2	4.25308	1E-05	0.08236	0.07937	0.09031	0.83784	1.02989	0.45375
Ckap2l	4.25143	0.00011	0.14768	0.08895	0.22772	2.20242	1.21984	0.98875
Gas2l3	4.23619	7.1E-05	0.05149	0.11027	0.11762	1.23941	1.10798	0.39399
Fam78a	4.23026	2.2E-07	0.1939	0.109	0.21262	1.82637	1.85297	0.78635
Ighd	4.22434	0.0028	0.91436	0.34266	0.54308	6.54536	10.318	2.65862
Scn7a	4.21678	1.9E-06	0.74182	2.04066	2.30396	17.1657	20.3859	9.0577
Hist1h3d	4.2094	0.02508	0.20682	1.16261	1.27564	12.5972	6.72815	1.26601
Tlr2	4.20671	2.5E-06	0.23056	0.33945	0.44769	5.21197	4.10185	0.9409
Grrp1	4.19976	1.1E-07	0.72671	1.45896	1.43183	9.12643	12.513	10.4259
Paqr3	4.1952	0.01684	0	0.06146	0.02098	0.18263	0.49799	0.16398
Disp2	4.17954	5.9E-10	0.09697	0.37963	0.28244	3.25765	2.15646	1.54889
Snai1	4.1758	1.6E-08	0.07145	0.74591	0.68552	6.86435	4.61237	2.89753
Slamf9	4.1755	2.6E-06	0.26463	0.49586	0.42316	3.48953	5.06661	2.29482
Ndc1	4.1627	6.3E-06	0	0.09444	0.19344	1.13727	1.17169	0.52193
4930578C19Rik	4.16049	0.0042	0.0331	0.05316	0.18148	0.78982	1.36284	0.45589
Ackr2	4.15974	3.4E-11	0.22223	0.44616	0.91379	7.4424	5.17736	2.664
Egr2	4.13663	6E-05	0.23859	0.35583	0.21023	2.58972	3.32646	1.51265
Stap1	4.13209	0.00061	0.03727	0.05986	0.07663	0.73721	0.72938	0.19963

Slfn2	4.12482	0.00361	2.71451	2.85063	1.908	19.2305	18.1496	8.62772
Cdca3	4.11069	0.02861	0.16165	0.03245	0.08308	1.09115	1.10923	0.30921
Mb21d1	4.10593	0.00015	0.02807	0.13525	0.07695	0.75793	0.97025	0.53696
Adora2a	4.10342	2.6E-05	0.13712	0.18352	0.25059	1.95161	2.36972	0.94426
Foxm1	4.09825	3.3E-05	0.08051	0.09699	0.19312	1.71894	1.28915	0.67767
Postn	4.09567	2E-09	0.62555	1.18639	1.1584	14.9514	11.2845	3.1061
Trim59	4.08973	0.00347	0.13821	0.08324	0.14208	1.43196	1.52799	0.44945
Mrc1	4.07743	2.3E-07	0.39994	1.27009	1.19601	13.1046	11.2734	2.71245
Cenpa	4.06999	0.00011	0.23108	0.22269	0.09502	2.06048	1.73842	0.91945
Siglec1	4.06983	1.3E-08	0.17836	0.37761	0.3667	4.30676	3.28849	1.17863
Osm	4.06786	0.00405	0	0.04094	0.13974	0.80024	0.6737	0.35105
Gm8369	4.06628	0.03697	0.06428	0.06883	0.02937	0.47094	0.87136	0.14756
Armcx4	4.06479	2.5E-10	0.60835	1.23298	1.40957	6.55773	11.6918	9.07707
Spc25	4.06446	0.00044	0.07266	0.08752	0.12449	1.2775	0.81253	0.52817
Rap2b	4.06005	8.5E-09	0.08525	0.39706	0.43233	3.16899	3.54459	1.63073
Cxcl16	4.05732	6E-11	1.57186	2.4322	1.91798	18.8496	18.5325	7.8324
Cd300ld	4.04312	0.00523	0.13136	0.10549	0.24007	1.2648	2.15899	0.53608
Rnf125	4.03932	0.00386	0.08682	0.27889	0.238	3.16201	1.80939	0.93005
Lyz2	4.03885	1.4E-05	45.1018	55.2468	63.072	809.697	662.744	197.961
Ccne1	4.03845	0.02564	0.13053	0.13976	0	1.01085	0.9952	0.26633
Lamb3	4.03245	7.5E-11	0.37733	0.70703	0.71831	7.59512	7.08587	2.32579
Clec4d	4.02754	0.02556	0.14486	0.05817	0.14892	1.63736	1.03089	0.4988
Litaf	4.0275	5.4E-10	0.92059	2.04182	1.44205	20.1222	15.9991	5.46751
Il7r	4.01488	6.8E-07	0.50545	0.37691	0.56907	6.50637	5.57883	1.82324
Fam198b	4.00979	3.5E-06	0.13081	0.39394	0.44824	3.48076	3.87322	1.55145
Cenpl	4.00791	0.00311	0.04461	0.10748	0	0.53228	0.46489	0.2731
Cd28	4.00306	0.00516	0.09459	0.07596	0.02593	0.76029	0.74046	0.21714
Aoah	3.9979	0.00459	0.19866	0.12763	0.08169	1.59673	1.5349	0.45602
Kctd12	3.99547	1.2E-06	1.30026	2.3494	1.97875	12.139	13.3061	5.15015
Cd48	3.99438	0.00014	0.48704	0.70402	0.46729	6.36125	7.0804	1.71427
P2ry10	3.99039	0.00156	0.11652	0.34309	0.13309	2.53639	2.25061	0.74296
Scd1	3.97474	1.9E-06	5.5802	12.9627	16.019	158.16	103.232	49.0748
Tnfrsf12a	3.96539	8.2E-06	0.72161	1.93164	0.65938	16.9166	9.35339	3.9571
B430306N03Rik	3.95455	0.00273	0.06678	0.05363	0.03433	0.41935	0.63649	0.28105
Prc1	3.9529	0.00133	0.18306	0.12439	0.193	1.73305	1.34563	0.5495
Fam124b	3.95201	0.04795	0.1035	0	0.09458	0.36829	0.87684	0.44878
Cilp	3.94692	0.00359	0.10896	0.19687	0.41068	3.79709	2.68604	0.43768
Foxf1	3.94444	0.00141	3.11729	5.69991	5.75166	22.8267	15.6746	8.8804
Tpx2	3.94366	1E-06	0.09905	0.23864	0.22062	2.17697	1.74965	1.0232
Slit2	3.93171	1.7E-17	0.18543	0.52649	0.63084	4.79879	4.81702	2.1485
Pbk	3.92769	0.03248	0	0.05193	0.26591	1.66487	0.98612	0.44533
Ifi209	3.92477	5.7E-05	0.29387	0.49821	0.26853	4.79784	4.36514	1.42413
Neb	3.91153	7.5E-06	0.03486	0.03149	0.02389	0.27086	0.33885	0.15669
Ncapg	3.90843	0.00015	0.01639	0.14479	0.0674	0.85427	0.77484	0.3888
Fabp5	3.90049	0.00094	0.58445	0.28682	0.31153	6.17775	3.68049	1.04347
1110038B12Rik	3.89886	2E-05	0.06395	0.20543	0.39445	2.52996	2.50309	1.02763
Ddah1	3.88723	1E-08	0.26171	0.30357	0.39856	3.30491	3.62126	1.53523
Cish	3.88546	7.1E-07	0.27065	0.40571	0.54407	3.18368	3.20082	1.60149
Zfp984	3.87982	0.01709	0.15909	0.19164	0.08177	2.57239	2.18341	0.36519
Plcl2	3.87624	1.3E-10	0.20018	0.29855	0.41156	3.41185	3.1398	1.44419
Selplg	3.86395	2.8E-05	1.16821	0.71476	0.87684	14.0772	10.0664	2.89443
Fxyd5	3.85691	2.8E-09	0.46969	0.84311	0.79524	9.83678	6.89547	2.47343
Gm14636	3.85277	0.00144	0.0661	0.09289	0.07927	0.78859	0.99953	0.30345
Itgb2	3.85117	2.5E-08	1.66974	1.88943	1.92451	23.6384	21.1026	7.31729
Cldn18	3.84002	6.8E-06	0.98769	3.67359	5.25472	35.6776	34.1522	11.0975
Lyve1	3.83936	0.00207	0.91259	2.99514	3.3446	39.5397	18.8977	5.95054

Pdpn	3.83559	3.4E-07	1.01987	3.37841	2.27154	29.0985	24.851	5.85276
Vgll3	3.83264	5.2E-05	0.24405	0.61595	0.3584	5.91092	3.77471	1.28049
Sell	3.82466	5.6E-06	0.27431	0.25176	0.16114	2.55914	2.79865	0.77961
C330027C09Rik	3.82428	0.00115	0.06109	0.08176	0.06977	0.76715	0.67276	0.35835
Emcn	3.8183	2.1E-06	0.06424	0.25793	0.20544	1.72101	2.19856	0.67174
Kcnb2	3.81498	0.02483	0	0.01227	0.01047	0.06234	0.08929	0.05259
Arg2	3.80787	0.01579	0	0.32566	0.16675	2.85201	1.15434	0.49647
Il2ra	3.80131	0.00099	0.02223	0.07142	0.1219	0.79581	0.98326	0.17013
Cdc20	3.79819	0.00242	0.124	0.34853	0.21245	2.76422	1.73336	1.3758
2410006H16Rik	3.7944	4E-05	0.25069	1.40922	1.54623	13.5378	11.0862	3.83639
Il2rg	3.7901	0.00013	0.63077	0.58447	1.03082	10.7246	8.01558	2.45034
C5ar1	3.7894	3.3E-08	0.1352	0.7238	0.43238	5.26345	4.14619	1.72414
1200007C13Rik	3.78492	0.00883	0	0.07577	0.09699	0.5036	0.71941	0.32488
Slc15a3	3.78152	2E-08	0.31639	0.28795	0.33247	3.60254	3.14138	1.61392
Rgs1	3.77277	0.00455	0.06307	0.05065	0.12967	1.14852	0.56638	0.41825
Mdga1	3.76063	0.00046	0.01233	0.0495	0.07604	0.4722	0.5013	0.22639
Srgn	3.75953	7E-07	0.42193	1.14355	1.33734	12.8496	9.11487	3.38986
Tubb2b	3.75459	0.00075	0.06026	0.67748	0.20649	5.67582	2.17475	1.15271
Pdgfrb	3.75452	1.3E-12	0.69771	2.52981	2.14443	18.7325	18.2482	7.55491
Ripk3	3.7498	3E-05	0.11794	0.3315	0.32331	2.77722	2.66779	1.12806
Slfn9	3.74945	0.00087	0.09708	0.15592	0.33266	1.96294	2.01337	0.63884
Prrx2	3.74152	0.00399	0	0.14229	0.12143	0.85298	0.87061	0.58749
Ace	3.74105	1.4E-08	3.26406	10.5024	12.1454	88.1876	65.0847	25.2771
Fcrls	3.7368	3.2E-08	0.04962	0.47819	0.88419	5.39059	5.01945	1.93644
Fstl1	3.73615	1.7E-11	1.74412	5.22393	4.49037	37.9911	39.2595	13.0027
Cxcl15	3.72767	0.00084	4.90067	2.92975	6.04533	68.8283	46.859	19.999
Cd276	3.72422	5E-06	0.2533	0.33054	0.15189	2.20684	2.71986	1.11442
Hpgds	3.72383	0.00044	0.16149	0.18156	0.08854	1.31824	1.55961	0.5684
Mybl1	3.71844	0.00061	0.02144	0.05165	0.08816	0.49812	0.51221	0.37732
Serpinb9	3.71825	3.8E-06	0.56352	0.66549	1.24943	9.61635	10.1433	2.46027
Cd72	3.70257	0.01286	0.23692	0.10872	0.34792	2.78408	3.30313	1.45025
Mfap5	3.69497	2.8E-05	0.25816	0.85519	0.50866	7.1328	5.28185	1.55561
Serping1	3.69207	0.00072	1.44463	4.96046	4.33564	56.7108	29.423	6.93718
Col8a1	3.68874	6.5E-06	0.06802	0.32775	0.24862	2.47736	2.43184	0.60723
Tbx21	3.68829	0.01655	0	0.03738	0.12761	0.55539	0.54423	0.39181
Cyp4f18	3.68601	9E-06	0.6868	0.95265	0.47068	5.13592	5.04611	2.53202
D8Ertd82e	3.68173	1.4E-06	0.07234	0.10651	0.10742	0.93122	1.10928	0.34135
Sash3	3.68094	9.4E-06	0.56244	0.27795	0.50405	4.5369	5.10204	1.78764
Rac2	3.6801	9.5E-05	0.80112	0.49016	0.52288	8.00084	7.00018	1.86815
Ccbe1	3.66941	2E-11	0.1096	0.19558	0.23367	1.57519	1.81981	0.87586
Nckap1l	3.66795	3.4E-11	1.17857	1.34081	1.21154	13.0281	14.3902	5.33565
Ccna2	3.66459	0.00031	0.14183	0.15946	0.15552	2.42243	1.98977	0.71626
Glrx	3.66054	3.4E-05	0.60278	0.55321	0.70816	7.67816	5.90902	2.10844
Hist4h4	3.65484	0.00584	0.04045	0.16243	0.33268	1.52413	1.52165	0.37144
Abcg2	3.64447	9.9E-07	0.07103	0.12549	0.10709	0.96338	1.01091	0.47827
Diaph3	3.64361	5.2E-06	0.17686	0.14203	0.25973	2.01488	1.68241	1.10196
March1	3.64287	3.3E-07	0.34036	0.31237	0.27768	2.5443	2.57037	1.09133
Rab8b	3.6378	7.2E-07	0.75859	0.9236	1.14037	11.6319	10.0503	3.35159
Smc2	3.63393	2.7E-08	0.16914	0.25468	0.37672	3.11987	2.62224	1.00301
Fgf2	3.63352	0.0151	0.31065	0.73453	0.93436	6.95727	6.21961	2.23169
Adgre1	3.63293	2.1E-05	0.49968	0.80254	0.80718	8.89748	7.45643	2.04824
Hist1h3c	3.63128	0.02241	0.4737	2.47262	4.22024	19.7803	12.8819	9.24264
Kmo	3.63094	0.00017	0.09807	0.19689	0.21843	1.97057	1.59516	0.71287
Kctd12b	3.62455	0.00593	0.09443	0.07583	0.12943	0.87461	1.35598	0.28902
Col5a2	3.61943	8.3E-11	1.61816	4.33478	3.79031	31.5443	31.5319	14.8302
Cdkn1a	3.61572	4.2E-11	1.92771	4.85335	7.71235	53.784	37.4467	23.4009

Cdh23	3.6153	0.01335	0.02051	0	0.02811	0.1288	0.1842	0.07848
Tcf19	3.61359	0.00051	0.12999	0.31316	0.26725	2.16301	2.31257	1.24327
Klrk1	3.61059	0.02317	0.10729	0.08616	0.09191	1.14529	0.92711	0.30785
Cdc7	3.60932	3.2E-05	0	0.22531	0.07691	0.7928	1.06961	0.55817
Tnfrsf13c	3.60853	0.01743	0.05742	0.23056	0.19676	1.62255	1.78044	0.61512
Ccr7	3.60455	0.001	0.40988	0.79937	0.2809	5.91958	4.73233	1.74732
Alox5ap	3.60382	2E-05	0.40781	2.03047	1.39741	12.9576	10.6549	3.37008
Lockd	3.60373	0.01945	0	0.01607	0.06857	0.31413	0.2136	0.22967
Itgb6	3.60216	6E-12	0.43976	0.6409	0.74785	5.8091	6.63969	2.69189
Slco2a1	3.59659	1E-13	1.36482	3.18923	3.90233	27.6209	27.2492	12.4803
Slc7a8	3.5963	0.00083	0.05744	0.1845	0.13777	1.58689	1.25538	0.32961
Podxl	3.59425	1E-09	1.85716	4.3047	4.57031	39.3713	33.2224	14.6786
Adat2	3.59326	0.04228	0	0.12689	0.10829	0.5953	0.80316	0.60451
Sparcl1	3.59034	1E-06	5.56027	17.5124	14.5936	170.735	116.34	30.7773
Kit	3.58895	1.2E-07	0.58979	1.09526	1.37677	7.41845	10.4083	6.06411
Ubash3b	3.58782	5.4E-09	0.15809	0.17774	0.23836	1.74718	1.84827	0.91935
Stab2	3.58726	0.01481	0.02846	0.0457	0.04875	0.2591	0.36162	0.23952
Thy1	3.58697	3.4E-07	0.10774	0.47586	0.77527	5.27688	4.29898	1.81363
Wnt2	3.58584	0.0037	0.05136	0.20623	0.17599	1.3868	1.38367	0.74669
Ctsb	3.58399	3E-08	2.90831	7.10469	7.28576	75.1049	55.5545	15.6895
Enpp1	3.58392	1.5E-07	0.02258	0.13602	0.11608	0.92884	0.93556	0.38016
Pcdh17	3.58017	2E-06	0.29232	0.43037	0.47579	2.0803	4.82288	2.50699
Lpxn	3.57937	0.00575	0.55317	0.16658	0.23693	2.90905	3.16324	1.42851
H2-DMb2	3.57892	0.00242	1.15142	0.97904	0.2785	9.35659	8.29707	3.73139
Eif5a2	3.56768	7.6E-05	0.06912	0.22202	0.17368	1.46118	1.60441	0.6699
H2-DMa	3.56513	3.4E-06	0.77636	1.19024	2.07985	15.0239	13.2739	5.23839
Dhfr	3.56335	0.00182	0.04379	0.07033	0.09003	0.59116	0.66773	0.36855
S100a10	3.55951	0.0003	1.14672	2.68589	1.70272	23.882	20.0124	3.72908
Vcan	3.55112	2.4E-09	0.15216	0.34729	0.41712	3.81182	2.6175	1.06624
Col4a1	3.54203	1.9E-13	5.4564	14.2575	15.9867	146.06	121.051	51.0963
Il10ra	3.52897	9.5E-10	0.62909	1.03697	0.9984	7.65102	8.56647	4.73758
Nid1	3.52579	1.5E-09	0.86203	3.81511	3.29517	29.2781	23.1454	8.50148
Syt12	3.52483	0.01276	0.04182	0.10075	0.15763	0.76153	0.77588	0.41598
Ifi30	3.52074	1.2E-10	3.66367	5.78931	4.94056	60.2589	42.051	17.4528
Arhgdib	3.51288	0.00015	1.62839	1.47114	1.11596	15.8489	10.9672	2.80346
Xcr1	3.51172	0.00741	0	0.06266	0.10695	0.55529	0.52882	0.19901
Tgfb1	3.50925	6.6E-12	1.02081	1.98109	1.4866	17.1199	14.2476	5.23978
Ptpro	3.50161	0.00873	0.03445	0.08301	0.03542	0.31371	0.648	0.19773
Ctgf	3.49904	0.0005	4.08869	15.4093	8.10098	96.443	60.25	24.5015
Pxdn	3.49864	1.1E-07	0.41377	0.94797	1.1009	9.72791	7.87467	2.46763
Gapdh	3.49732	0.00114	0.22577	1.1966	0.8355	11.5681	5.7609	1.69295
Rnase6	3.49698	0.04407	0.21608	0.08676	0.37021	2.4423	2.41636	0.57868
Slc39a14	3.49625	1.6E-13	0.41717	0.55541	0.65454	5.86635	4.58136	2.62082
Klhl6	3.49134	8.8E-08	0.44443	0.67811	0.45687	4.82795	5.19586	2.21041
Serpina3g	3.49095	0.00263	1.48557	0.39766	0.71643	10.2266	8.19444	3.1996
Gimap3	3.48157	2.7E-07	0.67415	0.8662	0.89322	5.19271	7.47029	3.26696
Slc16a6	3.4773	0.00021	0.11859	0.20952	0.09753	1.53406	1.53116	0.8167
Timp3	3.45762	0.00114	2.04788	10.7353	10.1263	63.4211	41.4355	14.9515
Aff2	3.45553	0.00197	0.03188	0.07679	0.08738	0.60046	0.43746	0.28048
Pdgfc	3.45099	3.4E-05	0.11379	0.12793	0.18716	1.18614	1.43448	0.69657
Slc35f6	3.44857	2.1E-07	0.09426	0.65606	0.55987	4.025	4.26442	1.82723
Cst3	3.44338	0.00017	3.11047	11.4608	11.9122	120.578	72.4964	17.0801
Lrrc25	3.44133	0.00418	0.32039	0.25729	0.21957	2.66562	2.23923	0.98059
Tmem173	3.43558	2.9E-07	0.15094	0.76766	1.31024	8.68797	6.24005	2.7333
Grn	3.43231	5.5E-09	1.6336	3.6411	3.99835	39.2311	26.3176	9.10695
Zfp995	3.43153	0.02047	0	0.02937	0.10025	0.43631	0.44614	0.33579

Tyrobp	3.42899	2.1E-08	3.12556	4.1833	4.641	44.7043	36.3643	14.6151
Il2rb	3.42665	0.00128	0.37428	0.23377	0.342	3.55144	2.83259	1.20919
Fignl1	3.41706	0.03234	0.04787	0.03844	0.08201	0.39076	0.62047	0.27471
Fbn2	3.41101	0.00715	0.0221	0.04437	0.05302	0.36086	0.43816	0.1353
Ifit2	3.40217	1.2E-06	0.11797	0.56843	0.76806	4.75952	4.51242	1.89563
Bora	3.40215	0.00462	0	0.06959	0.23755	0.68019	1.14527	0.62993
S1pr1	3.39857	7.2E-06	1.06465	3.35881	3.51784	38.703	16.9308	6.83711
Fzd1	3.38976	8.2E-14	0.80028	2.06095	1.83445	15.8206	14.6863	5.76445
Atad5	3.38849	1.8E-06	0.03075	0.09879	0.16861	0.84004	0.9692	0.50593
Capza1	3.38585	3.3E-06	1.04148	2.09091	1.65841	15.0415	12.7209	4.07828
Ccnb2	3.37869	0.01955	0.22695	0.18225	0.25922	2.28012	1.92264	0.86826
Fjx1	3.37669	8.7E-05	0.03696	0.29677	0.45588	1.22989	2.57351	1.89457
Csf1r	3.37238	5E-12	0.94837	2.74171	2.45349	21.2298	19.9693	6.24063
Heph	3.37114	3E-05	0.03023	0.10925	0.12431	0.86375	0.88361	0.30072
Vsir	3.36948	2.9E-06	2.58711	3.39461	3.75177	10.8495	12.7512	9.98616
Angptl2	3.36753	0.00048	0.31322	0.97655	0.78287	5.0559	4.09271	1.4803
Tnfsf10	3.3668	9.7E-05	0.48386	0.52986	0.81393	4.08792	7.40084	2.77676
Rrm1	3.36322	3.4E-09	0.16124	0.53645	0.50516	4.13677	3.73507	1.39241
Dusp6	3.36181	4.4E-09	1.44981	2.92731	2.6117	27.2333	19.4553	8.17743
Chsy3	3.35678	6.3E-05	0.15806	0.38079	0.1354	2.05946	1.84789	0.99778
Loxl2	3.35644	1.5E-07	0.57856	1.66917	1.49788	11.2083	9.47605	3.26281
Gla	3.35378	5.2E-05	0.21284	0.28487	0.2431	2.40565	2.30797	0.84142
Rpl39	3.34843	0.00056	2.423	1.88093	2.71219	31.2408	27.7935	7.10695
Vcam1	3.34279	4.4E-07	0.27236	0.60147	0.7466	6.08546	4.83384	1.82347
Arhgap15	3.33354	7.8E-08	0.23642	0.27424	0.29704	2.28455	2.70389	1.03515
Cd34	3.32855	4.3E-08	1.24781	2.55068	2.92304	16.212	15.5107	6.68343
Cd69	3.31458	0.02229	0.05844	0.18771	0.16019	1.39435	1.18812	0.49184
Ptprt	3.30942	0.00126	0.01837	0.059	0.06294	0.41522	0.36879	0.25298
Hcn1	3.30643	1.9E-05	0.00639	0.06673	0.03942	0.32911	0.3119	0.19074
Tmem97	3.30571	0.00669	0.08423	0.4735	0.34635	4.07267	2.39767	0.83787
Ifi204	3.3054	0.0082	0.19515	0.54849	0.31205	3.39021	2.0004	1.14478
Itk	3.30391	5E-05	0.12979	0.20846	0.1779	1.75228	1.62737	0.59588
Ccnf	3.29974	0.00281	0.07475	0.12005	0.2049	1.43153	0.96884	0.62912
Mmp3	3.29528	0.00406	0.25723	0.723	0.66107	7.67239	3.79182	1.03333
Fam105a	3.29271	7.9E-05	0.61388	0.36152	0.36461	3.88045	4.49336	1.65968
Csf2rb2	3.28976	0.00013	0.77849	0.50417	0.44746	5.39297	5.14422	2.53641
Plek	3.28576	4.1E-10	0.67127	1.05739	1.00853	8.90029	8.84514	3.18054
Itga2	3.28528	0.00017	0.27484	0.1545	0.35788	2.7268	2.33306	0.96738
P2ry6	3.27988	0.0006	0.52995	0.50296	0.52827	6.35296	4.92227	1.14278
Olfm1	3.27972	6.6E-09	0.29338	1.07328	0.82657	6.95945	6.61122	2.66884
Ighm	3.27916	0.03013	1.78089	2.95892	1.85176	23.0981	25.7211	5.30972
Nfam1	3.27278	9E-05	0.61109	0.66394	0.5666	6.72639	5.70076	2.00785
Cyp7b1	3.26925	8.9E-05	0.26736	0.38646	0.36645	3.59266	2.71797	1.18652
Asah1	3.26917	1.6E-05	0.81996	1.39924	1.47507	14.0733	12.2084	2.92788
Cacna1e	3.26635	0.00023	0.01984	0.04779	0.04079	0.32076	0.30251	0.16697
Tspan8	3.26258	3.2E-06	0.98926	1.9555	1.25161	14.0483	12.9579	4.59988
Ptger4	3.25562	1.5E-07	0.17422	0.33577	0.35818	2.22073	2.63008	1.06643
Chil3	3.2492	0.03705	2.27096	0.96884	2.62631	25.1333	23.5557	3.96403
Pi15	3.24468	0.01818	0.06692	0.36274	0.34395	4.4016	2.84875	0.89606
Il4ra	3.23979	2.2E-17	1.71741	3.42468	3.43835	24.2703	22.6383	14.0121
Rpl29	3.23898	0.00132	1.85146	2.72583	2.3967	30.6148	15.4237	3.30556
Vangl2	3.23643	9.1E-05	0.04971	0.07984	0.19304	0.56183	1.07804	0.67194
Serpine2	3.23539	1.7E-09	0.91266	4.16104	3.79312	26.3434	26.2182	9.95686
F830016B08Rik	3.23495	0.00727	0.42154	0.39494	0.36111	2.9117	3.19623	2.12345
Esco2	3.23446	0.04787	0.02888	0.02319	0.09897	0.43527	0.44043	0.2652
Napsa	3.23019	8E-05	1.6981	2.48256	3.5509	25.6182	17.6835	7.72931

Enc1	3.22407	9E-08	0.46406	0.5688	0.87039	6.57941	5.57428	2.22392
Stk26	3.22345	0.01376	0.06592	0.01765	0.12047	0.48291	0.73715	0.26901
Sema6b	3.2147	6.8E-07	0.37859	0.74837	1.03782	4.93741	5.43268	4.47894
Rad51	3.21356	0.02524	0.05226	0.01399	0.05969	0.37189	0.27448	0.1866
Ntrk2	3.20425	9.7E-06	0.09228	0.18189	0.17247	1.338	1.45409	0.50068
Col4a2	3.2033	3.8E-14	5.28439	13.2368	15.3134	108.249	89.9213	40.9832
Hist1h1a	3.20075	0.02522	1.44323	0.57949	0.61817	9.06246	7.51939	2.62273
Tbxas1	3.20012	5.7E-07	0.12736	0.2966	0.2182	1.64742	1.72846	0.82832
Cyr61	3.20001	0.00011	3.26978	8.10774	4.08855	49.3487	39.6558	22.8684
Hist1h4i	3.19733	0.0265	0	0.12793	0.65507	3.10112	2.83425	1.82848
Emp1	3.19601	0.00012	1.03042	4.63388	6.5438	44.6235	30.4134	10.4074
Abcb1a	3.19241	0.00033	0.14794	0.28853	0.34762	2.57461	2.07339	0.64687
Abca1	3.19158	4.5E-10	0.69293	2.52199	2.63479	14.9832	17.7188	7.41426
Tnfrsf1b	3.1914	2.1E-08	1.00106	1.94885	1.24735	13.2195	13.3224	4.71188
Mki67	3.19125	6E-06	0.73675	0.75805	0.89148	8.68869	6.6882	3.36478
Ctsc	3.19073	4.6E-07	1.43578	1.76153	2.51457	20.4981	18.225	5.78292
Malt1	3.18963	2.2E-07	0.5251	0.53169	0.71972	4.88856	5.70958	2.48061
Nos2	3.1896	0.00743	0.13125	0.06324	0.21588	0.93957	1.50781	0.54233
Acer2	3.18717	3.1E-06	0.79273	2.65034	3.23745	18.4584	16.9978	9.55651
Mirt1	3.18609	0.00071	0.05347	0.12882	0.03664	0.47846	0.70667	0.30686
Nrep	3.18453	1.1E-09	1.24438	3.0349	3.50593	15.6565	22.5131	14.4586
Amigo2	3.17609	5.6E-06	0.20171	0.61552	0.69116	3.06509	3.54745	3.24109
Dpp4	3.17291	9.1E-06	0.16496	0.45629	0.40195	3.36067	3.06515	0.96769
Cd80	3.17041	0.00877	0.13618	0.38275	0.13999	1.75295	1.62666	1.14618
Hist1h1b	3.16171	0.01755	1.40292	0.75108	1.06828	12.9204	9.27045	2.62403
Cox7b	3.15936	0.04602	0.38161	0.91936	0.98072	10.0043	6.64363	1.02198
Ccl22	3.15646	0.00015	0.3487	0.32003	0.78519	5.06738	3.57026	1.98205
Zfp429	3.15645	0.00353	0.10855	0.26151	0.26036	1.90847	1.48972	0.70597
E2f1	3.15196	7E-05	0.14689	0.20642	0.30199	1.89079	1.64257	1.09582
Lat2	3.1512	0.00091	0.75121	0.35486	0.42397	3.80141	5.25597	3.07687
Gpr132	3.14528	0.00116	0.23743	0.41947	0.35798	3.10108	3.0896	1.09004
Pcdh12	3.14418	0.00051	0.08245	0.26484	0.19776	1.29429	1.72874	0.77281
Ncapg2	3.14275	3.4E-06	0.15784	0.23942	0.24038	1.89414	1.80964	0.81857
Kif4	3.13245	0.00028	0.08402	0.11807	0.20153	0.97601	0.98226	0.59466
Cyyr1	3.13102	8.5E-07	0.84644	1.4013	1.6153	12.936	10.743	4.64326
Ttyh2	3.13024	6.4E-07	0.21683	0.52238	0.46702	3.83179	3.29075	1.4458
Cxcl12	3.12924	8.4E-07	0.59496	2.37754	2.16489	13.9564	13.645	6.69857
Ptgir	3.12844	0.00217	0.10422	0.08369	0.26188	1.26518	1.16542	0.77084
Glipr2	3.12677	6.3E-06	0.59906	0.84189	0.71846	7.46071	5.22737	2.71209
Cmtm3	3.12069	4.6E-08	0.23272	0.841	1.06327	7.18494	5.59928	2.49301
Cd38	3.11916	3.5E-05	0.53128	0.71108	0.65537	4.47033	6.7513	2.35779
H2-Aa	3.11659	3.1E-06	7.16917	11.2663	14.7395	115.842	98.2655	32.8662
Scml4	3.11637	0.0005	0.10495	0.10836	0.0822	0.79081	0.89164	0.33269
Ptpn7	3.11434	0.0005	0.22777	0.21339	0.1691	1.50172	1.76556	0.85687
Txnl4a	3.11318	0.00135	0.10601	0.21283	0.47224	2.92902	1.72438	1.09508
Adgrg2	3.11234	0.04269	0.02194	0.07049	0.03008	0.50983	0.45732	0.26865
S1pr2	3.10979	5.8E-07	0.08682	0.36255	0.4641	2.78039	2.69202	1.00977
Calhm2	3.0902	0.00221	0.07967	0.29858	0.27301	1.701	1.75494	0.56899
Veph1	3.08546	0.03642	0	0.06563	0.04481	0.40027	0.29078	0.11256
Dusp12	3.08379	0.04199	0	0.47471	0.60767	1.24613	1.11604	0.84001
F630028O10Rik	3.08208	0.00572	0.2109	0.3105	0.16862	1.63331	2.51923	0.64549
Zfp385a	3.0796	5.3E-05	0.35388	0.52777	0.62362	4.50769	4.93378	1.27651
Dock10	3.07576	1.3E-11	0.49161	0.74428	0.78981	6.29036	5.7925	2.47898
Tyms	3.07563	0.00026	0.07608	0.13747	0.11732	0.95551	0.85083	0.48029
Lacc1	3.07483	5.4E-06	0.28605	0.38286	0.45742	2.79409	3.42501	1.53213
Clec1b	3.0748	0.04958	0.04539	0.09113	0.10888	0.42755	0.78449	0.43416

Trpc6	3.07249	6.3E-05	0.13717	0.47734	0.54837	3.04342	2.51011	1.8018
Gja4	3.07164	4.9E-05	0.75609	1.71114	0.8479	7.25104	10.3418	4.83863
Plxnc1	3.06914	1.1E-08	0.47297	0.70029	0.63814	5.383	5.23651	2.26188
Ndrg1	3.06668	0.00013	0.33377	0.9734	0.74642	5.47129	2.89312	2.07001
Nxpe5	3.05683	0.01361	0	0.06238	0.08872	0.32518	0.43433	0.317
Ier3	3.05628	1.6E-07	2.3553	3.86508	3.93005	26.557	25.5578	16.7682
Slc25a45	3.05473	4.2E-05	0.17482	0.52646	0.23962	2.6895	2.46593	1.17046
Knl1	3.05385	0.00273	0.10847	0.13066	0.1208	0.97059	1.02691	0.43574
Clec14a	3.04054	0.00025	0.53585	1.39339	1.74869	9.19693	11.3229	4.29533
H2-DMb1	3.03627	2.6E-06	1.40387	1.81633	2.51214	16.896	16.4919	6.20642
Cdk1	3.03331	0.03976	0.20172	0.13499	0.13824	1.20331	1.29876	0.69456
Tgm1	3.03288	0.0014	0.23118	0.1547	0.31686	1.86291	2.11514	0.82547
Haus3	3.03287	0.03516	0.20944	0.40846	0.67665	2.25449	2.47895	1.3965
Hsd3b7	3.02839	0.01476	0.20914	0.73896	0.88861	4.75386	4.16713	1.95228
Irf4	3.02606	0.00013	0.2702	0.19726	0.26935	1.92805	2.06018	0.99616
Aprt	3.02598	0.00219	0.93229	1.55494	1.17954	5.58393	6.05117	3.2924
Pirb	3.02341	2.1E-06	0.544	0.85291	0.6391	7.28726	6.64952	2.29926
Tmem184c	3.01843	1.3E-05	0.1276	0.47819	0.52468	3.63231	3.26462	1.0089
Hsd11b1	3.01574	0.00322	0.57976	1.21767	1.22253	11.8177	7.79813	2.04745
Pld3	3.01471	9.2E-07	0.83181	1.94033	1.65587	15.9679	11.9192	3.57634
Adcy3	3.01309	8.9E-07	0.18543	0.40205	0.44477	2.32873	2.49774	1.26276
Cyba	3.01044	5.3E-06	2.45238	3.31215	2.97935	31.2184	23.6279	7.42059
Mrc2	3.00992	1.3E-10	0.9371	1.89529	1.93854	14.8416	12.5523	5.56368
Ogfrl1	3.00283	4.2E-05	0.14283	0.30968	0.21534	1.98206	1.7061	0.78686
H2-Ab1	3.00036	4E-06	8.3486	11.6474	13.1884	118.041	93.5036	32.2109
Chst14	3.00031	4.7E-06	0.11083	0.71203	0.53168	4.28006	3.40834	1.56888
Itga4	2.99908	4.6E-06	0.40601	0.75855	0.45995	5.22892	5.31518	1.6167
Rhod	2.99261	0.01165	0.40543	0.27132	0.27785	2.58827	2.30127	1.13749
Hhex	2.9924	0.00305	0.60385	0.24246	0.33107	2.84383	3.06941	1.66337
Cd22	2.98949	0.01302	0.5513	0.35049	0.31485	3.20215	4.4136	1.35339
Sdc3	2.98937	1E-09	1.35371	2.96621	2.79641	16.5756	14.4598	6.96949
Fkbp10	2.98685	5.5E-05	0.90761	1.01406	0.81131	9.09388	8.02335	2.77788
Akap2	2.98203	6E-05	1.97218	6.01599	6.36889	39.3254	22.7826	11.9094
Ugcg	2.9777	3.8E-08	0.53332	1.37407	1.44674	8.34426	10.2223	3.85972
Vegfc	2.97382	0.00059	0.18917	0.45574	0.3241	3.02902	2.06735	1.3389
Gm2a	2.96952	0.00028	0.95417	1.80293	1.8271	17.111	13.0667	2.94185
Cd4	2.96492	0.00084	0.1149	0.32295	0.1378	1.49709	1.76672	0.5275
Icam1	2.96431	1.4E-08	2.76266	3.60515	4.533	39.5157	30.2458	12.602
Serpinb6b	2.96182	0.00244	0.89497	1.56424	1.80393	14.5122	12.363	3.22257
Frrs1	2.961	2.2E-06	0.15953	0.19217	0.26239	1.53266	1.75154	0.79343
Gpm6b	2.96002	6.4E-05	0.28955	0.61452	0.60947	4.90902	4.48894	1.17106
Naip2	2.95826	2.5E-06	0.2868	0.42224	0.3112	2.94147	2.7334	1.24354
Plat	2.95084	0.00434	0.29692	0.55141	0.41969	5.80318	2.36768	0.96558
Adam12	2.94343	0.00108	0.12029	0.15456	0.16488	1.28411	1.19846	0.50625
Sema7a	2.94325	3.3E-09	1.89637	3.15857	5.15034	27.5204	22.5989	15.9882
Dck	2.93914	0.00024	0.15051	0.27196	0.41259	2.29189	2.33343	0.97891
Myadm	2.93797	0.00896	1.99064	4.17293	4.55331	31.9476	25.5197	9.21812
Rps27l	2.93657	0.00386	0.28387	2.05166	2.04269	14.7948	12.5534	2.60649
Adgrg6	2.93588	5.7E-07	0.26618	0.76003	0.76346	4.90289	4.78568	2.03673
B3gnt5	2.93498	0.0028	0.08914	0.14317	0.13746	0.9096	1.22342	0.30694
Olfml3	2.93436	8.1E-07	0.37598	1.11713	1.3012	9.12472	6.96595	2.60353
Bub1	2.93389	0.02137	0	0.17089	0.07292	0.78506	0.7031	0.16283
Clec12a	2.93156	0.00904	0.12571	0.16825	0.12923	1.35507	1.28861	0.2565
Oas3	2.93119	0.00974	0.11079	0.19573	0.15185	1.4331	1.06998	0.49168
Kcnh1	2.93077	0.00051	0.09479	0.20299	0.2815	1.28965	1.50973	1.00334
Nme2	2.92918	0.00032	1.48359	4.20961	2.84685	26.5814	18.7524	6.65979

C1s1	2.92721	0.001	0.70328	2.82385	2.91719	17.8039	12.2296	4.1917
Hist1h4f	2.92543	0.00185	2.96213	4.04385	4.263	30.5041	27.7042	12.9191
Ppic	2.92429	5E-11	1.98135	4.99035	5.36971	33.3658	33.3726	14.6093
F10	2.92368	0.00093	0.38168	0.27245	0.40689	2.66299	2.37124	1.78474
Lims1	2.91851	6.5E-07	0.7104	1.8636	2.04477	13.7245	12.4339	4.51166
Hvcn1	2.91474	0.00331	0.78015	0.80922	1.04702	7.63391	5.93175	2.21365
Rrm2	2.9112	0.01546	0.45977	0.26852	0.31509	3.22819	2.80441	1.11935
Itga1	2.90957	5E-05	0.69677	1.86515	2.00289	14.9852	11.2745	4.10226
Plod2	2.90915	8.4E-05	0.33257	1.02378	0.94966	7.39618	5.18417	2.16303
Ehd3	2.90752	8.8E-07	0.31662	0.50852	0.47737	3.39972	3.36362	1.67163
Tusc5	2.90511	0.00393	0.03508	0.25351	0.26442	1.73997	1.51546	0.48309
Shroom4	2.90016	1.3E-06	0.19564	0.73318	0.581	4.42254	3.68609	1.65665
Pros1	2.89952	8.9E-06	0.67996	2.22966	2.46584	15.9934	14.401	4.74753
Natd1	2.89913	3.4E-05	0.16362	0.3942	0.41116	2.73986	2.70306	0.98073
Cd40	2.89726	0.00083	0.10201	0.30038	0.16313	1.36655	1.5729	0.67649
Capg	2.89595	5.7E-08	0.96173	1.36074	1.38095	11.7903	9.00878	4.0298
Ptpn22	2.89529	0.00125	0.46934	0.31892	0.44536	3.87662	3.17479	1.29837
Thbd	2.89232	0.00255	1.61767	7.81942	9.91363	59.6968	35.3892	21.6612
Rnf150	2.89032	6.5E-07	0.09404	0.33984	0.4005	2.43799	2.44472	0.97656
Jam2	2.88695	0.00197	0.23669	0.63357	0.66234	5.53621	3.45889	1.28282
Il21r	2.88542	0.01103	0.38328	0.16789	0.42982	2.90992	2.65665	1.14642
Gnpda1	2.8829	0.00425	0.43577	0.50548	0.53092	4.28696	3.05186	1.85244
Gimap8	2.8758	9.8E-11	0.62605	0.98036	1.33003	6.36846	8.1147	4.28732
Mamld1	2.87021	0.00099	0.12075	0.23272	0.0993	1.22828	1.11703	0.48043
Gimap9	2.86597	0.01108	0.35268	0.28322	0.33837	3.14468	2.00779	1.02545
Nlrp1b	2.8601	0.00089	0.20206	0.22127	0.30213	1.53411	1.98883	1.12445
Cyb561d2	2.8569	0.0166	0.11734	0.18847	0.28146	1.51052	1.61046	0.67341
Kcnn3	2.85521	0.0011	0.03325	0.12459	0.10633	0.76547	0.65907	0.28831
Ctsz	2.85452	1.9E-07	4.17946	5.74446	5.67343	55.7181	38.1172	14.2678
Btk	2.85155	0.00347	0.3678	0.3249	0.25206	2.44807	2.78559	0.87242
Serinc3	2.84838	0.00014	4.70515	11.2143	13.5389	98.7486	72.9606	22.5013
Angpt1	2.84743	4.4E-05	0.16328	0.74302	0.69004	4.54539	4.27426	1.68662
Ercc6l	2.84331	0.04592	0.02184	0.14034	0.07485	0.39779	0.46635	0.234
Isg15	2.84261	0.00553	0.6128	1.10725	2.2048	12.9868	8.48806	4.5717
Sema3a	2.8402	0.00015	0.05846	0.27498	0.26328	1.52606	1.79993	0.51122
Bard1	2.83977	0.035	0.10233	0.03287	0.09818	0.69398	0.45774	0.3132
Cd74	2.83781	2.9E-05	33.3494	51.9793	65.8064	515.737	394.48	127.903
Lrrn2	2.83561	0.02006	0.06443	0.12936	0.11039	0.86988	0.63866	0.41907
Slc6a2	2.83182	7.1E-05	0.14731	0.87242	1.71617	6.52108	5.24133	4.07176
Was	2.82738	0.00164	0.14593	0.42969	0.40003	3.2988	2.39837	0.70718
Dpt	2.82714	0.01179	0.73899	4.0462	3.08468	26.9138	17.4497	5.55164
Ly6a	2.82542	0.0008	4.90189	8.95353	8.29958	89.2631	54.0346	12.1348
Tspan32	2.82405	0.01858	0.488	0.06532	0.33444	1.50662	1.44698	0.77792
Top2a	2.82253	0.0004	0.39836	0.48501	0.72213	4.60742	3.82112	2.10416
Gm14326	2.8219	0.02634	0	0.29238	0.4713	1.1685	2.69373	0.83575
Dlgap5	2.82058	0.03319	0.05225	0.06993	0.10742	0.59056	0.56658	0.23988
Sirpa	2.81936	5.2E-10	2.02819	3.12491	2.34353	18.6147	20.9904	9.61816
Ifngr2	2.81849	1.4E-08	1.11869	1.7125	1.31769	11.2613	11.0528	4.62764
Tfpi	2.81826	0.00038	0.19081	0.4086	0.33417	2.4495	2.47857	1.03821
Pgf	2.81401	0.02081	0.14642	0.11758	0.40138	1.56302	1.71181	1.00832
Mthfd2	2.81145	0.0153	0.16607	0.21338	0.13658	1.71022	0.9961	0.53371
Tmem206	2.81136	0.04954	0.10313	0.28988	0.24738	1.4895	1.70376	0.8286
Aqp1	2.81089	0.00063	2.77754	9.94877	8.49022	69.7917	47.1733	17.0022
Gucy1a3	2.80481	1.7E-06	0.56513	1.45493	1.26441	8.47498	9.20076	3.25586
Igsf10	2.80407	0.00809	0.09129	0.30239	0.40664	2.57945	2.05905	0.56752
Cnr2	2.80338	0.00607	0.31994	0.1635	0.2392	2.0638	1.71499	0.82345

4930503L19Rik	2.80058	5.7E-06	0.22452	0.56665	0.4616	3.08145	3.19545	1.54614
Gpx7	2.79729	0.00351	0	0.50509	0.43104	2.48251	2.83167	0.75627
Mafb	2.79717	2.4E-06	0.7921	1.35517	1.36891	7.63377	8.98037	5.19127
Sema3c	2.79644	0.00397	0.63263	1.40685	1.67862	10.9904	9.55628	3.3512
Adam15	2.79497	6.8E-07	0.96123	2.95145	3.13875	13.8351	11.1036	6.30223
Apbb1ip	2.79215	9.8E-06	0.32806	0.45162	0.28906	2.93298	2.80302	1.13555
Ier5l	2.78873	4E-05	0.21584	0.62399	0.41417	2.87327	3.02804	1.78364
Adamts7	2.78841	0.00024	0.03687	0.16286	0.17689	0.62515	0.89029	0.46553
Aaas	2.78751	9.4E-05	0.25511	0.35851	0.78674	3.48413	3.4039	2.00079
Sulf1	2.78711	3.7E-07	0.14357	0.60941	0.54115	3.5159	3.16927	1.33394
Atp6v0c	2.78686	0.00681	2.08776	4.80984	4.19851	23.7908	17.7797	6.73034
Rpl7l1	2.78399	0.00135	0.75554	1.74882	1.97976	8.8745	6.41576	2.61849
Pkhd1l1	2.7824	0.03187	0.04395	0.10588	0.04217	0.38637	0.67468	0.1547
Sema5a	2.77977	5.3E-06	0.22502	0.80885	0.55809	4.68963	4.00863	1.3528
Mcm3	2.77348	1.2E-05	0.22001	0.40384	0.88312	3.67085	3.8502	1.94797
Pde1b	2.77134	0.0004	0.27445	0.47228	0.45678	2.53582	3.10898	1.71001
Sgk1	2.77056	4E-08	0.96851	1.80213	1.73219	13.8392	9.60577	5.22361
Myo5a	2.77013	5.9E-10	0.39903	0.63459	0.55764	4.289	4.14004	1.79001
Rpl22l1	2.76993	0.00261	0.81317	1.30604	2.46797	9.62875	12.4002	3.73326
Ggta1	2.76637	3.9E-06	0.29514	0.96781	0.89335	5.85331	5.22578	2.31479
Tgds	2.76546	0.04763	0.1534	0.24638	0.35043	1.477	1.5855	0.78252
Emilin1	2.7633	4.7E-12	1.19643	2.58096	2.47588	17.3088	13.9278	7.68272
Gng11	2.76313	8.2E-06	1.00822	1.82171	1.72738	10.9999	12.2355	4.91801
Tubb5	2.76113	1.1E-07	4.17743	7.21257	6.91859	60.8784	43.919	16.4615
Loxl1	2.7592	3.6E-06	0.9836	3.21595	2.96114	23.0068	17.4275	5.08016
Snn	2.75866	1E-06	0.47892	1.21789	1.66842	6.34036	8.60146	5.16088
Flt1	2.75028	7.5E-06	0.61994	1.74246	1.80222	13.4929	7.87289	4.26921
Nrros	2.75012	1.2E-05	0.77638	0.69027	0.7791	6.35506	5.49672	2.63082
Mcm5	2.74595	0.00081	0.37707	0.27952	0.39756	2.85951	2.12308	1.53138
Lrp12	2.74594	2.2E-06	0.12661	0.56936	0.45118	3.22768	2.67714	1.22062
Cstb	2.74362	0.00025	2.1269	2.95019	3.31273	29.0176	18.772	6.50969
Aplnr	2.74096	0.01124	0.25049	0.37716	0.68665	1.21899	4.28126	2.20413
Apobec1	2.7407	0.00095	1.11447	0.86055	0.76377	6.40594	7.69118	3.64059
Zfp277	2.74043	0.0136	0.28318	0.1895	0.32344	1.95596	2.08713	0.46947
Gap43	2.73453	0.04674	0.22814	0.24428	0.26058	2.19659	1.31426	0.93101
Tmed5	2.73368	4.5E-05	0.18078	0.45972	0.35103	2.90413	2.44276	0.88759
Themis2	2.73347	3E-06	0.52662	0.78539	0.64447	4.65316	4.93303	2.33136
Sox17	2.73185	6.3E-07	0.4002	0.98558	0.76795	5.37782	4.23125	3.30719
Naip5	2.73085	0.00013	0.13353	0.3932	0.27454	1.99843	2.05891	0.91958
Spon1	2.72481	2.9E-05	0.59126	1.82756	2.13302	13.7859	10.6436	3.55097
Runx3	2.72039	0.00083	0.34725	0.27886	0.28557	2.5875	2.02396	1.06281
Galnt10	2.71875	0.00014	0.17475	0.37422	0.30605	1.92636	2.03311	0.77255
Rab29	2.71867	0.01267	0.31558	0.57022	0.32441	3.26971	3.24835	0.78479
Abcd2	2.71612	0.00145	0.14669	0.3029	0.30158	1.94744	2.0025	0.72154
Tcf21	2.71375	0.00235	0.2841	1.06469	1.94701	9.57393	7.3168	3.47816
Sh3pxd2b	2.70532	5.3E-11	0.84696	1.48484	1.40613	11.3857	7.64618	4.31739
Eif2b1	2.70469	0.00184	0.13462	0.28829	0.49204	2.98682	2.14409	0.68671
Nox4	2.70106	0.00036	0.19141	0.538	0.51535	3.76895	3.12739	0.92062
AB124611	2.70072	0.01555	0.24012	0.57849	0.38397	3.41768	2.92933	0.97991
Zfp760	2.69979	0.03366	0	0.11049	0.43373	1.19223	1.60851	0.52638
C5ar2	2.69787	0.02672	0.06505	0.07836	0.2229	0.7148	1.20685	0.34841
P2ry2	2.69506	5.6E-06	0.19081	0.7406	0.52305	4.21341	2.84496	1.72764
Lipg	2.69135	0.00041	0.09175	0.71224	0.58686	3.43755	3.0314	1.70829
Ap3m1	2.68442	0.0015	0.19812	0.37124	0.44128	3.0376	3.28975	0.77061
Det1	2.68015	0.00891	0.10722	0.1722	0.09185	0.95936	0.76297	0.43069
Sh3bgrl3	2.67816	0.01054	4.65534	4.41819	3.86714	52.618	29.5432	6.80036

Psat1	2.67711	0.00354	0.02723	0.15305	0.48512	1.79508	1.67453	0.66663
Scamp5	2.67265	0.01309	0.28119	0.15054	0.25694	1.51063	2.00102	0.64547
Crlf1	2.67089	7.7E-05	0.07045	0.96177	0.96561	4.60069	5.08499	2.37184
B4galt6	2.66982	8.7E-05	0.19941	0.25622	0.25966	1.9033	1.7333	0.67137
Uba52	2.66868	0.00423	1.31437	3.97367	2.33138	21.2159	11.9472	5.62013
Dhcr7	2.66865	0.01316	0.14395	0.18495	0.17757	1.06658	1.2585	0.52868
Plk2	2.66569	5.1E-08	1.3227	1.75926	2.26618	13.2631	12.2911	6.83156
Manba	2.66165	1.3E-06	0.14953	0.56037	0.31881	2.38908	2.67712	1.04245
Fhl3	2.65973	0.01386	0.48172	0.49737	0.47162	3.24092	2.69346	1.52703
Slpi	2.65917	0.0072	0.29446	1.33995	1.21077	6.22487	8.63114	2.32817
Nfkbid	2.65758	0.03234	0.19748	0.19823	0.23683	1.519	1.22962	0.94438
Kif18a	2.65754	0.03968	0.01178	0.05676	0.04844	0.2293	0.2934	0.17126
Nrp2	2.65226	9.3E-10	0.55748	1.11529	0.7574	6.08611	6.09991	2.58184
Irf5	2.65184	0.00021	1.31567	0.9605	0.98362	8.43674	7.94408	3.56923
Acp2	2.65152	9.1E-07	0.44666	0.82499	0.91831	5.49724	5.36948	2.11896
Prelp	2.64631	0.01217	1.37766	5.40299	5.46718	30.9615	20.4869	6.66799
Stat4	2.6462	0.02538	0.14956	0.19217	0.123	1.22087	1.3836	0.50353
Myc	2.64615	0.00112	0.42628	1.12966	0.6427	5.08575	4.26855	3.26171
Capn6	2.64445	0.03804	0.02972	0.07159	0.0611	0.39185	0.45315	0.11369
Rpl24	2.6444	0.02494	4.04805	5.50828	3.23657	52.0381	33.6081	7.39939
Cd1d1	2.6437	0.02309	0.18026	0.43428	0.12354	1.16022	1.19117	0.72414
Scn3b	2.6429	8.7E-06	0.86955	1.66908	1.14823	8.88269	10.9851	3.09953
Nsg1	2.64284	0.00018	0.4323	0.66538	0.66658	5.47426	3.31437	1.8744
Sema6d	2.64124	5.8E-07	0.25228	0.828	0.65399	4.77311	3.65193	1.96394
Rps15a	2.6397	0.00018	0.5911	1.11254	0.9621	8.25854	6.4505	1.8657
Ptprb	2.63524	3E-05	2.17793	5.3797	6.27014	36.876	29.9694	14.1352
Fam49b	2.63397	7.7E-06	0.62864	0.98562	1.02576	7.18052	6.64946	2.38215
Tgfbr1	2.6336	9.4E-07	0.67357	1.65121	1.5792	9.75026	9.70377	3.79764
Mfsd7a	2.63266	0.01816	0.13854	0.29668	0.15824	1.13091	1.0563	1.16606
Hist1h3f	2.63155	0.04631	1.36579	1.53552	0.7488	11.7494	7.98373	3.23967
Tpgs2	2.63031	0.04163	0.08466	0.08159	0.09283	0.60605	0.60248	0.2073
Garnl3	2.62712	9.7E-05	0.07501	0.12801	0.10281	0.73009	0.62913	0.40895
Cd248	2.62503	5.4E-06	0.27145	1.08994	1.08518	5.90901	5.91013	2.63092
Lcp1	2.62128	4.5E-05	1.31613	1.82024	2.17975	16.7123	14.117	3.79509
3830406C13Rik	2.62024	0.00286	0.18703	0.63833	0.54475	3.14159	3.1848	1.71731
Rps11	2.61972	0.00063	6.82865	11.8277	14.1311	88.8689	67.719	20.9001
Clec1a	2.61456	1.9E-05	0.62605	0.93368	1.34842	6.02308	7.43279	3.66116
Cotl1	2.61438	0.00012	4.86208	4.66209	4.02833	42.8337	34.3047	12.7711
Sdk2	2.61189	0.00114	0.02995	0.10221	0.0667	0.50305	0.48333	0.18333
Slc41a3	2.60577	0.04244	0.07957	0.1917	0.21813	1.37408	1.09208	0.45665
Ctla2a	2.60409	0.0064	1.34968	2.77739	2.42801	22.8825	12.7776	4.84089
Hells	2.60388	0.00193	0.09645	0.18072	0.12118	0.95892	0.90697	0.4305
Arhgap20	2.60335	0.01457	0.05478	0.17597	0.16269	0.76252	1.19738	0.3563
Foxf2	2.60302	6.1E-05	0.19605	0.70849	0.63821	3.69325	3.88655	1.50014
Ccng1	2.60263	2.5E-06	1.18721	5.50848	4.97211	28.2456	25.9993	11.2037
Snrpf	2.59945	0.04543	0.13452	0.97222	1.10624	5.57483	4.03416	1.44098
Clstn2	2.59828	0.00297	0.10176	0.29418	0.22315	1.99355	1.26205	0.46716
Kcnq5	2.59822	0.02161	0.07531	0.26612	0.25807	0.85126	0.42877	0.25356
Stk17b	2.59765	1.6E-08	0.7386	2.03359	1.59083	9.82787	10.2797	5.05942
Frem1	2.59706	4.9E-05	0.07457	0.14639	0.29528	0.83247	1.34775	0.74812
Rab3il1	2.59425	8.9E-05	0.38241	0.42993	0.4892	3.0416	3.34334	1.30697
Heyl	2.59387	0.00027	0.31713	0.89134	0.77619	4.55161	5.69945	1.54258
Gnb4	2.59202	0.00013	0.06388	0.37373	0.39399	1.89666	1.86466	0.90771
Gdf11	2.5905	0.01476	0.11405	0.20608	0.13678	1.05634	1.20293	0.34907
Rtn4rl2	2.58671	0.01707	0.23072	0.43231	0.52704	3.33208	2.58002	1.11805
Cd83	2.58468	0.04747	0.5945	0.43401	0.51854	4.00452	4.6152	1.07519

Fam63b	2.58384	1E-04	0.47136	1.09231	0.95063	5.58131	5.27788	2.22582
Pde4b	2.58173	0.0079	0.38387	0.687	0.72157	6.61836	3.11081	1.40148
Vrk2	2.58151	0.00379	0.08786	0.14111	0.19268	0.9048	1.04505	0.36303
Arsa	2.57998	6.8E-06	0.15736	0.63185	0.73334	3.67584	2.75159	1.85429
Rap2c	2.57635	0.00039	0.32671	0.7871	0.35824	3.36449	3.96904	1.22494
Abca5	2.57582	0.00308	0.12152	0.26836	0.29842	1.7932	1.68321	0.60439
Zfhx4	2.56705	0.02222	0.02208	0.06501	0.03026	0.23569	0.23942	0.12952
Sh3kbp1	2.56621	1.6E-06	0.38989	0.5249	0.44009	3.4707	3.2525	1.36879
Rubcnl	2.56306	0.0184	0.26213	0.28067	0.17964	1.37168	1.73216	0.83573
Rpl32	2.56153	0.00022	6.73507	11.5899	11.6216	86.1689	70.9757	22.8223
Nt5dc2	2.55684	0.00296	0.82316	0.99156	1.2975	8.42528	7.02941	2.96032
Peak1	2.55628	1.4E-07	0.61512	2.01774	2.17206	14.9524	10.5247	4.94597
Sftpc	2.55111	0.02211	89.4574	148.42	209.292	1686.18	829.544	268.397
Lpl	2.55086	4.1E-05	1.49664	2.89542	2.23783	14.6948	15.6817	6.57172
St6galnac3	2.54886	0.00052	0.04091	0.09855	0.1682	0.68497	0.7624	0.3443
S100a6	2.54619	0.00059	3.7279	7.75639	3.54188	48.36	34.2375	10.4374
Fstl3	2.54572	0.00093	0.14211	0.79885	0.58434	5.65153	3.90068	1.30484
Maf	2.54571	1E-05	1.06811	1.24147	1.07872	5.44498	4.93631	2.31203
Sla	2.54521	0.01428	0.39086	0.50625	0.5962	5.9378	4.60793	1.833
Tmem123	2.5448	9.3E-05	1.49011	3.79932	3.3955	24.9594	21.0755	5.7009
Dchs1	2.5444	3.5E-05	0.25151	0.65641	0.64636	3.05987	2.51956	1.29098
Mex3a	2.53563	0.00935	0.08018	0.19316	0.08242	0.8559	0.76417	0.35276
Adgrf5	2.53477	0.0013	2.08977	5.35058	6.86318	43.5079	28.2044	10.7051
St3gal1	2.53444	1.6E-07	1.46371	2.64939	3.16855	22.1899	13.9118	6.84432
Tmem167	2.53337	0.00039	0.1286	0.42171	0.39661	2.09289	2.49496	0.77083
Mfap2	2.53319	1.9E-05	0.66948	1.53606	2.03184	10.7498	8.75049	4.82986
D430019H16Rik	2.53132	0.00172	0.10009	0.3215	0.13718	1.05582	1.36342	0.6586
Gm8113	2.52924	0.03028	0.38308	0.64945	0.32088	1.28293	2.09868	1.56332
Prkcb	2.5284	0.0089	0.65848	0.41128	0.41784	3.88976	4.38839	1.1943
Lrrc40	2.5261	0.00358	0.04855	0.17545	0.21628	1.24996	0.95013	0.48295
Marcksl1	2.52579	3.1E-06	1.15458	1.85438	1.82978	10.9203	10.7472	5.19024
Mertk	2.52416	3.4E-07	0.50751	1.78038	1.59258	9.97973	6.99233	4.33292
Cystm1	2.5231	0.01041	0.57094	0.68774	0.83845	6.27392	4.99577	1.49781
Manea	2.51933	0.00181	0.09519	0.47775	0.30986	2.36095	2.08052	0.6373
Ikzf3	2.51909	0.01452	0.4366	0.40374	0.14507	2.44248	2.78418	1.02247
Cep192	2.51885	1E-07	0.25766	0.58626	0.53955	3.07408	3.23789	1.50056
Ankrd49	2.51841	0.00074	0.08765	0.39888	0.16019	1.24757	1.4703	0.82718
Parvg	2.51655	0.00092	0.52475	0.5117	0.53943	3.29504	3.52466	2.00759
Ecscr	2.51592	0.00253	0.62046	1.16261	1.32288	8.65788	6.13243	3.27052
Tmem86a	2.51151	0.00013	0.18413	0.93649	0.71507	3.81553	4.52376	1.97248
Ikzf1	2.51041	0.00034	0.70169	0.72449	0.61828	5.94197	5.03418	1.64141
Tek	2.50442	0.00015	0.78655	2.38838	2.88047	13.968	12.7813	6.30049
Mreg	2.50415	0.02274	0.15213	0.3665	0.27801	1.56024	1.77851	1.04762
Brca1	2.50354	0.01314	0.08144	0.0763	0.13953	0.71593	0.58644	0.37388
Col5a3	2.50189	0.01305	0.30617	0.58737	0.60617	3.71695	2.94832	1.78308
Rsad2	2.50128	0.00972	0.10704	0.4298	0.36679	1.99964	1.99956	1.00333
Clcn5	2.50112	1.2E-07	0.17193	0.35735	0.42972	2.21633	2.20022	1.006
Rassf4	2.5003	3.4E-08	0.45276	0.86858	1.01705	4.89633	5.74713	2.53092
Egflam	2.49384	8.4E-06	0.24598	0.9269	1.07629	5.20407	5.12636	1.9835
Slc20a1	2.49339	1.1E-06	1.01705	1.53348	1.74962	7.48116	6.95273	4.79631
B130024G19Rik	2.49269	0.04674	0.01893	0.1976	0.0908	0.4041	0.75044	0.39104
Blm	2.49157	0.04378	0.05128	0.15099	0.05857	0.63324	0.63424	0.1831
Hbp1	2.49051	0.00117	0.35683	0.84457	0.96529	5.4129	4.67763	1.55197
Camk4	2.48552	0.00769	0.08453	0.06788	0.08368	0.51311	0.46788	0.30903
Pqlc3	2.48494	0.00263	0.19275	0.48155	0.32289	2.83751	2.27517	0.65548
Dcbld1	2.4844	0.00018	0.19144	0.4612	0.4723	1.80313	2.58839	1.72847

Uck2	2.48265	6.1E-10	0.48753	1.0043	0.97328	5.8165	5.15016	2.75723
Pik3r6	2.48019	0.00469	0.35174	0.33382	0.32871	1.7268	2.24303	1.49251
Rnf122	2.48008	0.00405	0.09642	0.15486	0.19824	1.02929	0.93122	0.54104
Prorsd1	2.47751	0.03575	0.10885	0.43707	0.2238	1.94802	1.79823	0.54139
Racgap1	2.47706	0.00299	0.17997	0.1708	0.23546	1.2431	1.11439	0.6635
Lgals9	2.4757	0.00188	1.73431	2.35024	1.26284	18.139	10.4409	4.10549
Slc6a14	2.47145	0.00222	0.23609	0.92088	1.31749	5.86638	5.89742	1.98712
Pdgfd	2.46925	0.00786	0.07344	0.29489	0.25166	1.3835	1.32526	0.73054
Tpp1	2.46793	1.5E-05	0.5607	1.76507	1.81371	11.0695	9.02907	3.1405
Iigp1	2.46752	0.02893	1.89259	1.40064	1.55135	12.49	12.8457	5.25306
Fau	2.46466	0.04269	9.94766	6.04841	12.2712	110.829	77.6524	16.7455
Scd2	2.46456	3.4E-05	3.33461	10.3532	11.0915	49.4666	54.7148	26.2303
Zfp948	2.46397	0.00082	0.26605	0.4273	0.4862	2.89568	2.36657	1.25534
Anxa3	2.46391	1.9E-14	2.51159	5.76996	5.81734	26.8905	30.2518	18.415
Brip1	2.46388	0.02256	0.03108	0.04991	0.11713	0.48783	0.45018	0.19022
Plxdc1	2.46317	0.00771	0.11975	0.13463	0.22978	1.39858	0.88867	0.4398
Zfp37	2.46208	0.03338	0.08504	0.13658	0.13598	0.71196	0.79244	0.43378
Epha4	2.46006	0.00033	0.17256	0.34644	0.30551	2.01365	1.79085	0.80324
Bcl2l11	2.4596	4.6E-05	0.24523	0.46336	0.42509	2.83514	2.64697	0.94923
Ifitm3	2.45898	0.00121	8.02876	12.895	11.824	109.411	63.7339	23.6583
March3	2.45681	0.00012	0.07714	0.30972	0.23788	1.42885	1.49972	0.66399
Unc5a	2.45498	0.00247	0.05558	0.31241	0.19043	1.20396	1.11584	0.72291
Tspan7	2.45446	0.01067	0.73749	1.44771	1.87566	10.9393	8.61369	3.44851
Daglb	2.45418	9.9E-06	0.26152	0.48565	0.44806	2.494	3.13216	1.25064
Tank	2.45386	1.8E-05	0.17099	0.47298	0.5078	2.97061	2.56886	0.94494
Adamts1	2.45385	0.00109	1.74755	6.10468	5.01508	41.8087	21.2967	8.64142
Slc31a2	2.45332	9E-05	0.34182	0.91499	0.89797	5.07975	4.34368	2.4411
Degs1	2.45274	0.00023	0.58111	2.04612	1.89931	11.5924	9.97473	3.59134
Spn	2.45135	0.00096	0.54433	0.5166	0.54261	3.51124	3.69754	1.8364
Sgpl1	2.45116	9E-07	0.52791	1.34751	1.39545	8.15699	7.16844	2.89968
Zfp709	2.44905	0.01656	0.07281	0.11693	0.15966	0.84119	0.9844	0.2674
Eno1	2.44358	0.0002	1.09812	2.14409	2.11012	16.7788	13.5385	4.05292
Armcx2	2.44314	0.00011	0.20445	0.4515	0.57796	2.98479	2.97474	0.97772
Commd3	2.4422	0.00058	0.34795	0.99795	0.88571	5.02529	5.0786	2.16797
Itga5	2.44103	3.8E-05	1.32976	2.61731	2.03704	16.4706	12.1798	5.46646
Ndufa4l2	2.43964	0.04322	0	1.16261	1.15752	5.68174	3.67946	2.95402
Pim1	2.4376	7.2E-07	3.07329	2.57378	2.73804	11.4961	11.6493	6.18127
Pdgfra	2.43709	3.7E-05	1.16697	3.44236	3.55531	22.1139	15.5459	6.71904
Stx11	2.43666	0.00347	0.29003	0.46582	0.3644	2.21578	2.53078	1.22057
Mcemp1	2.43129	0.03938	0.31009	0.43578	0.31877	2.62866	2.12788	0.88976
Adamtsl3	2.42914	3.6E-06	0.16452	0.42036	0.47148	2.2633	2.29586	1.17871
Plpp1	2.42885	0.01251	0.49586	2.0479	2.08748	13.967	7.59746	3.79415
Lef1	2.42818	0.01929	0.14497	0.10584	0.16258	0.9848	1.04505	0.35295
Tlr3	2.42782	0.00212	0.20019	0.2858	0.3506	1.96935	2.12556	0.62972
Bmpr2	2.4254	0.00111	1.6062	4.34808	4.47405	26.9172	22.1009	8.14051
Maged2	2.42455	0.00013	0.50061	0.68917	0.66165	4.49066	3.78056	1.86051
Nptx1	2.4187	0.00765	0.0222	0.17828	0.36514	1.25462	0.95918	0.81537
Akap5	2.41832	9.3E-07	1.54448	2.95763	3.39638	16.5829	17.306	8.88283
Lax1	2.41594	0.02921	0.45144	0.36253	0.48126	2.67723	3.16156	1.26656
G6pdx	2.41567	7.5E-05	0.71447	1.08711	1.10813	6.82399	5.81068	2.64711
Zfp52	2.41482	0.02311	0.15975	0.64144	0.32844	2.64155	2.54432	0.93714
Ncapd2	2.41362	5.7E-05	1.20125	1.75124	1.48183	4.5142	4.63117	3.49278
Zfp930	2.41361	0.03749	0.03859	0.21695	0.26449	1.139	1.23589	0.50202
Treml2	2.4133	0.02177	0.15412	0.24753	0.10562	1.19356	0.94006	0.53066
Dock11	2.41314	0.00109	0.21309	0.40263	0.26629	2.23531	2.02609	0.69055
Cd44	2.41268	2.6E-07	1.43578	2.41581	2.7059	17.1405	14.9437	5.29683

Hist1h2bc	2.41118	0.00673	4.09144	3.53837	4.09806	31.0272	22.6367	9.27144
Dock2	2.41024	0.00105	1.09483	0.81408	0.8962	6.49284	7.4974	3.31988
Ptprn	2.41008	0.01293	0.08177	0.24078	0.16812	1.28371	0.91445	0.43799
Rhog	2.40891	0.00115	1.14188	2.81646	2.17996	18.1816	11.9401	4.36862
Mettl23	2.40332	0.02621	0	0.27589	0.2943	1.75276	1.28788	0.32859
Rnase4	2.39746	0.00768	1.56669	3.26183	3.69824	24.5943	18.8176	4.35111
Brca2	2.39635	0.00355	0.04547	0.10225	0.11842	0.42831	0.66106	0.29923
Grb10	2.39274	1.2E-05	0.2676	1.3917	1.12654	6.44126	5.84244	2.30107
Sdcbp	2.39124	2.2E-05	1.20785	2.74823	2.39131	15.1267	15.0504	4.74936
Fkbp5	2.39007	0.01137	0.41865	2.11948	2.83163	17.7044	6.80032	3.59329
Pmp22	2.3897	0.00136	1.77124	4.18815	4.24851	28.7012	20.3574	7.52936
Psmb8	2.3867	0.00393	1.18528	1.2238	1.62459	10.2338	7.07919	2.33212
Nme1	2.38529	0.03553	0.13862	0.77925	0.855	3.30772	3.0475	1.538
Map2	2.38478	0.00027	0.05633	0.11874	0.14959	0.64993	0.78738	0.31248
Rgs10	2.38004	0.00627	0.7446	0.81539	1.20613	5.61067	6.46862	2.9005
Car2	2.3786	0.00374	0.81293	0.96004	0.6882	6.48591	5.42044	1.82949
Prmt6	2.37595	0.0243	0.22077	0.68698	0.62409	1.16099	1.41673	1.03465
Rasl11a	2.37572	0.02271	0	0.61954	0.5948	3.39106	2.20584	1.10683
Cxcr4	2.37524	0.00151	0.96089	1.95483	1.75604	13.3948	8.5637	3.82323
Sox7	2.37083	0.00979	0.42142	1.09986	1.03488	7.27703	4.62331	2.0422
Arhgef6	2.3691	6.5E-06	0.31149	0.71779	0.63112	3.3931	3.76551	1.66837
Dclk1	2.36761	0.00138	0.07572	0.17026	0.25946	1.16012	1.32399	0.57358
Rhoh	2.36749	0.04228	0.17451	0.12457	0.19933	0.94971	1.32074	0.43027
Id2	2.36574	0.00465	2.00632	2.34352	1.62496	16.9504	12.6087	4.11702
Tgfb2	2.3651	4.7E-05	0.34	0.7388	0.86349	3.96848	4.25956	1.49971
Klhl13	2.36354	0.01595	0.02723	0.21869	0.18663	0.8208	1.02433	0.45842
Lrp2	2.36198	0.00052	0.34674	0.49565	0.78893	4.97296	2.67901	1.4327
Gucy1b3	2.36194	0.00017	0.67834	1.39738	1.27336	8.01896	6.77612	3.22708
Phlda3	2.36158	7E-08	1.18865	4.5341	4.58215	19.4527	21.7888	11.8237
Ifitm2	2.36109	0.02289	2.12188	5.96391	6.42254	51.0749	28.5818	6.35903
Pop4	2.35944	0.01543	0.122	0.26126	0.33444	1.68537	1.50899	0.65345
Txndc16	2.35896	0.0002	0.06708	0.29626	0.36009	1.5514	1.61953	0.67577
Rab13	2.35885	0.01104	0.07015	0.56335	0.43268	2.11439	1.92553	1.61031
Dse	2.35859	0.0004	0.44188	0.82176	0.79692	5.30113	4.65769	1.44142
Mrps14	2.3546	0.0245	0.03555	0.25693	0.34107	1.8974	1.22874	0.40801
Kif22	2.35455	0.04005	0.09449	0.17073	0.32377	0.86031	0.98459	0.65069
Ntpcr	2.35382	0.00661	0.25998	0.34796	0.41572	1.95896	2.42269	0.96147
Mme	2.35225	0.01488	0.09031	0.33845	0.45387	2.5015	1.72401	0.52211
Celf2	2.3501	2.8E-06	0.26847	0.63601	0.56117	3.35197	2.98405	1.20516
Nmnat2	2.34852	0.00285	0.12021	0.30568	0.15103	1.23286	1.23221	0.52121
Fermt3	2.34847	0.00026	1.25355	1.15047	1.13521	8.40554	7.21384	3.63115
S1pr3	2.34737	9.3E-05	0.49076	1.18231	1.11519	7.10394	5.76372	2.19377
Plin2	2.34733	1.2E-05	1.28615	3.09852	3.05107	19.9417	13.6115	6.51028
Pik3r5	2.34587	0.00011	0.73039	0.72061	1.0011	5.72637	4.29602	3.14563
Spred3	2.34404	0.01049	0.16764	0.22437	0.19148	0.93569	1.01306	0.96915
Spi1	2.34388	0.00745	1.03141	0.79377	0.7363	6.44956	5.7233	2.23607
Cd37	2.34063	0.01309	1.15096	1.0035	1.05921	5.94695	7.60547	4.22722
Mcm4	2.34027	0.00311	0.29044	0.62196	0.59712	4.03247	3.37907	0.86423
Gpr182	2.33688	0.00311	0.23433	0.56454	0.48178	2.72218	3.11181	0.98617
Wsb1	2.33434	4.1E-07	1.22007	2.51942	2.26951	9.28459	12.359	8.49086
Actb	2.33392	3.3E-05	62.2977	123.942	104.794	839.677	583.417	204.962
Calu	2.33276	0.00084	1.4406	5.26577	5.42998	30.2883	24.0636	8.41924
Scoc	2.33128	0.00255	0.1624	0.65208	0.76053	2.97431	2.90298	1.49116
Pole	2.33081	0.0359	0.12077	0.08729	0.14898	0.69769	0.71824	0.45281
Chst15	2.3305	0.03211	0.70936	1.26014	0.98701	4.81872	3.75867	2.07242
Prex2	2.32749	8.1E-05	0.99033	2.02498	2.32695	11.3398	11.0831	5.41262

Arpc1b	2.32746	1.7E-09	1.83409	3.45487	3.61562	21.3131	18.5764	7.72723
Pecam1	2.32613	5.8E-05	2.92635	5.86424	6.8628	40.5439	30.0361	13.291
Wdr5b	2.32514	0.03694	0.06492	0.31281	0.31144	0.89685	1.64998	1.04318
Selenon	2.32453	1.6E-05	0.64006	1.2593	1.07468	7.4153	5.87249	2.54671
Ifi208	2.32321	0.01501	0.04408	0.24779	0.22656	1.30091	1.09789	0.47219
Cdk19	2.32028	0.00038	0.41605	1.20914	1.4935	5.46131	5.79046	2.77413
Naip6	2.32016	0.00309	0.20358	0.2316	0.19765	1.01201	1.44872	0.75289
Cfh	2.31889	0.04651	0.53573	2.80224	1.97465	15.3108	9.70026	2.59248
Laptm5	2.31386	3.2E-06	4.06651	5.03347	5.40608	37.9566	28.7052	14.271
H2-Eb1	2.30964	0.00216	19.075	18.5389	25.8767	165.354	145.637	56.7351
Acot9	2.30742	0.00079	0.61145	0.8593	1.08252	5.56725	5.15416	2.37829
Mad2l1	2.30652	0.00687	0.05522	0.16628	0.10406	0.71946	0.59643	0.34857
Gimap6	2.30634	0.00214	1.027	0.93115	0.86275	6.09519	7.20736	2.33212
Gjc1	2.30301	0.00032	0.1852	0.50195	0.30144	2.22409	1.70624	1.09824
Abcb1b	2.3029	0.00425	0.15767	0.21706	0.23154	1.31535	1.13346	0.70662
Adam10	2.3023	0.00017	1.06083	2.91539	2.73031	15.6318	14.1756	5.19494
Nynrin	2.29956	0.00091	0.45169	0.97562	1.09944	3.21771	3.18266	1.96643
Mob3a	2.29483	0.0442	1.31414	0.59801	0.93062	8.0593	5.94502	1.97754
Tmem229b	2.29236	0.00068	0.29371	0.37345	0.53676	2.32072	2.71216	1.1424
Zeb2	2.29228	1.3E-06	0.8944	1.50179	1.40103	8.25655	8.85048	3.43515
Zfp41	2.29176	0.02429	0.08541	0.18291	0.1756	0.94751	0.88278	0.4357
Ncf2	2.28973	0.00024	0.64096	1.11897	0.68754	5.59971	4.87289	2.26029
Snhg1	2.28904	0.00021	9.16723	4.85562	5.85472	7.52002	7.23747	6.4473
Dapp1	2.2878	0.00198	0.26398	0.37097	0.40704	2.32061	1.96238	1.03517
Naa60	2.28619	0.04651	0.63754	1.38964	1.91411	6.44337	5.33931	2.50879
Pmaip1	2.28597	0.00128	0.26263	0.66786	0.53995	2.88596	3.07039	1.50715
Enpp2	2.28559	0.00037	0.33348	0.8302	1.27984	5.42358	4.86498	2.32205
Ptbp3	2.2849	0.00023	1.20774	2.15114	2.57856	13.3105	12.4511	4.63244
Satb1	2.28479	2.7E-05	0.20612	0.29343	0.28893	1.64725	1.69061	0.75272
Ppa1	2.28366	0.0064	0.11973	0.4487	0.65643	3.15765	2.29237	1.00775
Cenpf	2.28234	0.01195	0.1225	0.3197	0.3218	1.74989	1.39058	0.75764
Dnttip2	2.28212	0.02666	0.42002	1.18053	1.12739	4.43962	3.13126	1.49977
Rab32	2.28176	0.02423	0.60437	0.57358	0.5648	3.96751	3.60266	1.4714
Zfp160	2.28058	0.00148	0.15276	0.36801	0.54437	2.11023	2.51574	0.91169
Tmem185b	2.28033	0.00908	0.24607	0.42816	0.50592	2.72981	2.6267	0.87867
Mlc1	2.27957	0.03481	0.20675	0.23244	0.82178	3.7908	2.12281	0.91753
Pold4	2.27689	0.01506	0.89092	1.22649	1.30835	6.3136	8.08672	3.01894
Mrpl30	2.27489	0.02578	0.32312	0.40363	0.27065	2.7278	1.8249	0.71424
Zdhhc13	2.27142	0.02207	0.3364	0.50171	0.36228	2.23309	2.93135	0.88253
Aim2	2.26845	0.03415	0.19849	0.18596	0.3174	1.45411	1.44614	0.68345
Ppfia4	2.26814	0.0011	0.17623	0.26889	0.28986	1.2726	1.38847	0.98437
Cfp	2.26748	0.00477	0.93403	1.17868	0.82299	7.07991	5.66331	2.39929
Runx2	2.26708	0.01222	0.30144	0.34732	0.13473	1.43198	1.37235	0.75213
Tmem100	2.26371	0.03705	1.76973	3.26346	4.71656	27.2055	17.858	6.72052
Hif1a	2.26285	0.00035	0.78575	1.40396	1.57507	11.5207	7.98797	2.82576
Pcdh19	2.26269	0.01166	0.03321	0.07111	0.15929	0.59771	0.61324	0.28795
B3gnt7	2.26253	0.04701	0.24748	0.14195	0.3392	1.17658	2.04864	0.73039
Kif11	2.25929	0.0086	0.16824	0.48251	0.31295	1.99209	1.99129	0.82754
Slc39a10	2.25903	0.00311	0.15202	0.62565	0.54695	3.57963	2.35679	0.90147
Pag1	2.2586	0.00011	0.5803	0.89319	0.75396	5.07869	4.98993	1.83161
Psap	2.25852	8E-07	33.26	45.7247	45.7031	305.164	256.897	119.819
Dhrs1	2.25752	0.00084	0.20982	0.84247	0.54641	3.29376	3.3915	1.38068
Itgae	2.2568	0.01963	0.45509	0.42637	0.43316	3.28625	2.40317	1.27679
Npc1	2.25436	0.00047	0.41036	0.8412	0.92509	5.71638	4.08943	1.66086
Sacs	2.25391	3.3E-07	0.11914	0.27337	0.27995	1.35021	1.26025	0.75102
Nnmt	2.25271	0.0428	0.14698	0.17705	0.60436	2.16887	1.75568	0.95593

Mcam	2.25252	1.1E-08	3.21528	6.82989	6.04182	35.2559	29.9629	16.4014
Fam20c	2.24901	5.2E-05	0.32911	0.55061	0.75182	3.20319	3.1227	1.86768
Eng	2.24881	0.00036	2.4892	5.85899	6.74513	40.7288	25.6253	11.3622
S100a11	2.24836	0.049	12.0126	25.4818	26.7165	163.1	120.392	31.7369
C1ra	2.24736	0.02641	0.77705	1.77843	2.0236	13.2473	7.01084	2.55665
Tmsb10	2.24568	0.00031	10.529	12.9746	10.5126	101.68	72.6664	28.6836
Cdk2	2.24558	0.00975	0.57148	0.38244	0.75065	3.55856	3.4858	1.63977
Arpc3	2.24434	0.00583	0.46126	1.65004	1.55182	11.2697	5.39263	2.31017
Spcs1	2.24379	0.03043	0.52441	1.98531	1.1295	9.36127	6.89244	2.57951
Fli1	2.23947	0.00154	1.0964	1.33403	1.02461	8.49099	7.29559	2.74556
Hyal2	2.23842	0.00153	0.31654	0.73435	0.96414	5.16791	3.48616	1.69544
Clp1	2.23504	0.02011	0.17338	0.45251	0.23764	1.76916	1.45416	0.82916
Mis18a	2.23465	0.01137	0.16182	0.42232	0.24951	1.52413	1.39827	1.14529
Gusb	2.23377	2.8E-05	0.96771	1.18613	1.58816	8.73098	7.98672	2.9813
Chst3	2.23232	0.00379	0.39218	0.4581	0.35429	2.03727	2.91774	1.1867
Tgfbr2	2.23129	3.5E-06	1.7259	4.64646	4.32397	24.7662	21.7882	7.69625
Gprc5a	2.23074	0.00136	3.28052	10.0465	12.2698	60.7507	49.4033	17.4859
Tbx5	2.22733	0.00053	0.39639	1.01464	0.781	4.21579	4.40748	2.21788
Bend4	2.2273	0.00715	0.07029	0.12231	0.15255	0.76507	0.73247	0.26893
Vps29	2.22636	0.00601	0.26246	0.54197	0.71947	3.57863	3.62109	0.97543
Cryab	2.2256	0.03711	1.56618	4.21337	2.68333	24.2421	16.2404	4.3741
Fbln2	2.22448	0.04598	0.21818	0.96367	0.97191	4.81797	4.65795	1.23818
Slfn5	2.22432	0.00047	1.22667	2.36712	2.20829	13.6117	10.4695	4.42683
Blnk	2.22369	0.04027	0.21265	0.34154	0.33122	1.96656	2.00463	0.5769
Macf1	2.22318	1E-07	4.63034	11.9916	12.3629	54.8669	54.795	29.7582
Pou2f2	2.22068	0.01946	0.30911	0.24823	0.26962	1.46458	2.07117	0.78482
Nid2	2.21981	0.00382	0.166	0.45704	0.26003	1.99536	1.61522	0.79838
Tcn2	2.21957	0.02007	1.01036	2.00707	2.33236	17.2633	8.35227	2.88892
Pip4k2a	2.21946	2.5E-05	0.93268	1.23049	1.3012	7.59264	7.11129	2.98206
Casp3	2.219	0.00272	0.41401	0.60953	0.56746	2.64301	3.96334	1.31994
Abhd18	2.21791	0.04569	0	0.17075	0.16029	0.42725	0.6701	0.26031
Axl	2.21529	4.4E-11	2.76144	6.85599	5.64584	28.0906	29.7105	16.4458
Mef2a	2.21525	0.00026	0.50846	1.66879	1.68927	9.74495	8.20872	3.0448
Arpc2	2.21515	2E-05	3.75999	8.00615	7.14474	45.1811	38.9773	13.6004
Cap1	2.21412	0.00012	2.5524	5.98939	4.86142	34.7604	25.7962	8.85191
Syngr2	2.21331	0.01744	1.63034	4.13721	3.26251	20.8842	16.1432	5.38909
Ism1	2.2132	4.4E-06	1.71873	4.01521	4.87213	22.5169	19.9944	9.92311
Samhd1	2.2132	2.3E-07	1.46831	2.2566	2.48095	14.2114	12.0638	5.77244
Brox	2.21276	0.00234	0.50859	0.94679	0.8872	5.2839	4.71206	1.53892
Dennd2c	2.21261	0.01523	0.08444	0.18987	0.11574	0.54083	0.79834	0.51689
Plvap	2.21011	0.00057	2.27325	6.27235	6.35143	41.7618	27.1691	14.5397
Cndp2	2.20982	1.6E-05	1.42109	2.94081	2.84679	16.9203	14.697	5.22773
Tspan4	2.20913	0.00069	0.85083	2.47681	2.51457	14.9927	12.7041	4.15027
Rell1	2.2089	9.4E-05	0.63076	0.98894	0.92629	5.94107	4.5039	2.3672
Tmem140	2.20885	0.00728	0.3364	0.33371	0.62381	3.18092	2.4844	1.33241
Entpd1	2.20872	5.2E-05	0.87176	1.95018	2.19058	12.9161	8.37703	4.06575
Osmr	2.20641	0.00058	0.31553	1.37444	1.78889	7.87722	5.98288	2.69966
H2-T23	2.20421	0.00011	5.69274	5.02227	5.35747	19.1822	16.6486	10.1535
Tnfaip3	2.20365	4.3E-06	0.4007	0.68218	0.68103	3.52258	3.27516	1.88868
Anxa1	2.2017	3.7E-05	3.46392	7.02558	7.36513	37.6737	40.3612	13.1164
Plekhg1	2.19973	6.8E-06	0.34458	0.48958	0.58129	2.48829	2.76875	1.65295
Nemp2	2.1981	0.02154	0.08182	0.09198	0.29156	0.68842	1.03969	0.56343
Arl15	2.19547	0.00035	0.75744	1.52065	1.46288	5.90198	7.73518	4.37307
Rpusd1	2.19516	0.04945	0.06801	0.32769	0.41947	1.7509	1.45191	0.93668
Gypc	2.19285	0.03949	0.29812	0.43891	0.61292	3.27596	2.52558	1.02649
Slc1a3	2.19276	0.02085	0.05427	0.22879	0.26033	1.31192	0.92407	0.45675

Nt5e	2.19141	0.00225	0.77027	1.2715	0.90913	6.81179	6.89533	1.85001
Gsap	2.19121	0.00088	0.32218	0.66021	0.68523	3.3625	3.28663	1.57264
Lamp1	2.18996	0.00715	4.97914	14.9162	14.569	77.8103	57.3088	18.453
Trps1	2.18971	1.1E-05	0.25103	0.7855	0.8661	3.92439	3.79716	1.56311
Nrxn1	2.18958	0.03204	0.05463	0.10236	0.07072	0.23631	0.40418	0.25544
Timp2	2.18934	0.00441	2.91332	11.1964	10.6143	53.742	46.9339	17.4921
4931406P16Rik	2.18898	9E-07	0.88562	2.28034	1.90749	13.117	11.1183	4.3993
Agpat4	2.18864	0.00098	0.4288	0.92974	1.0873	6.11213	4.25024	1.93581
Strip2	2.18727	0.04041	0.12515	0.11167	0.12389	0.75966	0.53012	0.44688
Tmco1	2.18649	0.02621	0.17018	0.52713	0.51649	3.14475	2.44679	0.61387
Slc25a40	2.1848	0.01764	0.07175	0.13829	0.21636	0.81097	0.80236	0.49411
Coro1a	2.18342	0.01855	3.62065	2.1389	2.13905	18.763	19.377	7.83351
Ckap4	2.18305	3.2E-07	1.57475	3.58305	3.95709	18.7407	17.5909	8.37721
Prim2	2.18145	0.04118	0.12977	0.28658	0.22233	1.5686	0.90698	0.64542
Lpcat2	2.17942	0.02842	0.51278	0.54905	0.32799	2.83349	2.78023	1.30786
Eif2s2	2.17682	0.00018	0.42864	1.18412	1.26903	4.68333	4.2356	2.19092
Cmklr1	2.1768	7.5E-06	1.25743	4.47577	3.40037	19.2485	16.238	8.21716
Cfl1	2.17673	1.1E-05	7.67536	14.5005	15.7127	94.4143	68.3498	27.5951
Ctps	2.17598	1.5E-07	1.36217	2.04754	2.27395	12.0187	9.35621	6.38731
Ctsh	2.17506	9.2E-08	2.96033	8.84746	8.67978	36.2194	38.3943	21.1335
Slc16a2	2.17494	0.00476	0.21735	0.82907	0.72614	4.69146	2.77578	1.30966
Slc25a53	2.17462	0.02994	0.05818	0.07008	0.16945	0.45665	0.44359	0.52306
Ap1s3	2.16818	0.03189	0.24146	0.29085	0.38611	1.97104	1.71827	0.92377
Ifi207	2.16612	0.00224	0.99815	0.90844	1.14009	7.22875	4.95527	3.10592
Ext1	2.16534	1.2E-07	0.67395	1.56607	1.36596	7.98665	6.82228	2.9734
Gimap5	2.16458	0.03804	0.54144	0.23717	0.87705	2.65808	2.95232	1.77015
Mfsd12	2.16113	0.00019	1.07915	1.75665	1.81892	6.02542	6.10803	3.74925
Mtpap	2.15785	0.00239	0.3932	0.49118	0.77846	2.93539	3.70861	1.47088
Smim13	2.15653	0.00191	0.20756	0.66673	0.51564	2.78588	2.88818	1.03232
Map3k8	2.15514	0.00056	0.33429	0.64016	0.54631	2.77729	2.7841	1.67246
Smc4	2.15372	4.6E-05	0.68914	1.46386	1.47777	7.32846	7.25439	3.1468
Stard4	2.15187	0.00115	0.29027	0.49951	0.55416	2.52576	2.84555	1.17399
Itgav	2.15175	1.4E-07	0.65757	1.55822	1.58096	7.52037	7.56166	3.25811
Upk1b	2.15152	0.03565	0.26189	0.57835	0.76277	3.16558	3.5609	1.15222
Slc39a8	2.15069	0.01334	0.52892	0.8495	1.22208	5.66513	5.89174	1.6651
Flt3	2.15068	0.01561	0.16878	0.25601	0.44981	1.53083	1.52516	1.09054
Zfp365	2.15009	0.01215	0.15576	0.39312	0.24399	1.64874	1.3912	0.74915
Mgl2	2.14957	0.01622	0.94027	0.93275	1.74363	6.80723	7.2816	3.17409
Lrrc32	2.14925	0.02302	0.29647	1.76574	1.37142	8.48576	5.02315	2.5331
Sdk1	2.14913	0.00877	0.05223	0.21811	0.08591	0.69532	0.62657	0.26378
Nlgn2	2.14889	0.00013	1.56161	2.55633	2.07179	9.76807	10.6447	8.70121
Adam11	2.14628	0.03294	0.18387	0.34454	0.23802	1.30853	1.25656	1.00048
Tcf7	2.14362	0.04526	0.35297	0.67321	0.75593	6.59384	5.31524	1.75553
Nedd9	2.13897	6.1E-05	1.76973	2.52655	2.61095	16.0825	14.0057	5.22846
Gja1	2.13805	0.00929	0.82062	2.06686	1.8661	11.6643	8.09599	3.53911
Itgb1	2.13777	0.00082	5.53412	15.6116	14.237	84.3097	64.3675	23.3006
Ttpal	2.13696	2.9E-05	0.7201	1.32847	1.00033	6.41594	5.97518	2.56137
Rcan1	2.13683	0.00038	1.71002	2.93204	2.5972	16.0775	12.0985	7.1434
Ctsl	2.1367	0.0007	2.2917	5.757	5.75867	31.8431	26.3741	9.48703
Robo4	2.13573	3.5E-06	0.56796	1.05253	1.39225	5.88449	4.58579	3.6772
Manbal	2.13573	0.00386	0.52312	1.59635	1.29062	5.9127	7.76442	2.56175
Slc25a24	2.13536	0.00088	0.60762	1.00301	1.13357	5.19322	5.86826	2.29881
Rps7	2.13523	0.00127	9.15948	17.1306	16.9318	102.77	85.7647	27.9418
Megf11	2.13499	0.00033	0.1761	0.24955	0.26266	1.21633	1.23735	0.72127
Wipf3	2.13481	0.00298	0.11396	0.353	0.24546	1.60501	1.62198	0.59795
Med22	2.13407	0.00011	0.41662	0.7206	1.62523	5.15161	5.115	2.94258

Lactb	2.12922	0.01057	0.22754	0.68523	0.62376	3.53629	2.6602	1.21875
Ptprd	2.12871	0.00012	0.20807	0.5135	0.58429	2.84888	2.36806	1.05233
Man2a2	2.12663	6.7E-09	0.72969	1.92021	2.03106	8.27572	8.91316	5.26552
Rab5c	2.12616	0.00726	1.90041	4.87511	3.65391	26.3193	20.6345	5.41256
Gucy1a2	2.12598	0.01077	0.06545	0.36793	0.47099	1.82894	1.77995	0.77628
Slc33a1	2.12311	0.04526	0.35908	0.89129	0.87248	4.14056	2.97012	1.49866
Dennd4a	2.12257	6.8E-07	0.49409	0.92768	0.77737	4.36104	4.25891	1.9968
Tmem55a	2.12106	0.01589	0.24758	0.83505	0.47508	3.0471	2.96999	1.32609
Actr3	2.12071	6.4E-06	3.02615	5.08123	6.22163	30.4034	27.4843	11.5201
Cyth4	2.12032	0.00191	2.32385	3.06585	2.27511	13.0287	15.5036	8.92239
Rftn1	2.11873	0.00398	1.00287	0.90861	1.07499	5.53843	6.01261	3.24653
Slc30a4	2.11822	0.02609	0.42254	0.66168	0.66603	2.9186	3.45797	1.1801
Picalm	2.11822	6E-05	1.21185	2.63723	2.51052	14.795	11.5203	4.79202
Skp2	2.11805	0.01789	0.10295	0.1063	0.17134	0.58181	0.69524	0.40512
Leprot	2.11791	0.00194	1.09839	3.43024	3.2619	16.3232	15.6639	5.55629
Eif3e	2.11701	0.00265	1.65349	3.9835	3.70854	18.8305	18.032	7.30358
Psmd14	2.11661	0.00279	0.57499	0.62665	0.95698	4.40999	4.42579	1.63414
Nfya	2.11586	0.00067	0.3325	0.80103	0.86806	4.4441	3.58137	1.59917
Pappa2	2.11558	0.01254	0.01125	0.10842	0.16963	0.59345	0.59478	0.22383
Nek6	2.11526	0.00192	0.36754	0.58163	0.47413	3.29216	3.02214	1.16628
Atp7a	2.11356	0.00055	0.25195	0.92647	0.8179	4.03017	3.86229	1.51185
Naa20	2.11152	0.01804	0.34386	0.7029	0.36419	2.82661	2.68536	1.10028
Sh3bgrl	2.11111	0.00857	1.7559	4.79426	4.9578	24.2788	23.4914	7.4164
Jak2	2.11054	1.4E-06	1.03616	2.92154	2.28809	12.7958	11.1774	5.4617
Peg3	2.11007	0.00039	0.2347	0.68409	0.60763	2.93656	2.33294	1.6894
Kirrel	2.10809	2.3E-05	0.35694	1.15902	0.89338	4.95041	4.6384	1.84057
Mbnl1	2.10661	0.00511	0.94267	2.2537	2.00713	11.197	11.2402	3.10544
Mfge8	2.10514	4.6E-05	1.28304	3.42789	3.38189	17.1822	14.3353	6.6456
Inpp4b	2.10445	0.00244	0.19501	0.23491	0.28551	1.39708	1.49138	0.59008
Slc36a1	2.1042	3.5E-06	0.51466	1.1407	1.05812	5.33877	5.11697	2.3943
B3gnt3	2.10399	0.00166	0.64449	0.94379	0.8314	5.30866	4.49002	1.82753
Alg13	2.10309	0.025	0.01691	0.14936	0.15063	0.57332	0.67036	0.27168
Mmrn2	2.10274	0.01329	0.32376	1.22908	0.66564	4.23235	4.38356	1.75665
Rnasel	2.10235	0.00084	0.38368	0.46217	0.49003	2.31068	2.69491	1.30776
Zfp639	2.10224	0.00077	0.12693	0.37373	0.39143	1.64713	1.62367	0.92263
Cd151	2.1018	0.00107	1.35214	3.4611	2.83785	18.5729	12.8654	5.10837
Rgs4	2.10164	0.00247	0.29908	0.75055	0.743	3.59171	2.98347	1.83077
Mdfic	2.10153	3.2E-05	0.62377	0.80147	1.19695	5.55214	4.79563	2.36258
Fndc3b	2.1009	3.5E-06	0.82692	1.78162	2.14932	10.6446	8.35014	3.75779
Rps25	2.10051	0.01291	2.03787	5.10437	5.48661	28.3349	20.3472	6.60849
Zfp738	2.09965	0.0038	0.1776	0.55267	0.44121	2.10498	2.01993	1.05319
Ass1	2.09962	0.0183	0.52776	1.19439	1.34809	6.32664	4.60923	2.12924
Id3	2.09612	7.2E-06	1.74602	5.03732	4.34313	18.5166	17.783	13.6073
Chsy1	2.09443	0.00347	1.6086	3.16265	3.44026	8.30709	6.83731	4.07451
Il1r1	2.09212	1.1E-05	0.67347	1.46171	1.52185	7.30348	6.48575	3.38438
Brcc3	2.09131	0.00918	0.08811	0.24765	0.24153	0.97743	1.21669	0.50563
Slc2a9	2.09115	0.02645	0.06836	0.14638	0.26546	0.71539	1.00763	0.54048
Tmem30a	2.09072	0.00089	1.18978	2.784	3.16311	14.4525	13.5239	5.65062
Zfp46	2.0888	6E-05	0.66665	1.01717	1.03556	4.57678	5.29752	2.82253
Mcm2	2.08848	0.00037	0.26079	0.55847	0.69503	3.0204	2.63647	1.46334
Itgb5	2.08813	2.1E-05	1.86027	6.25222	5.80778	27.4724	25.846	10.9128
Plscr4	2.08808	0.01511	0.19357	0.41452	0.79594	2.43096	2.96816	1.23427
Fkbp14	2.08708	0.0089	0.25123	0.40351	0.72696	2.82211	2.45475	1.06797
Serpinh1	2.08681	0.04606	3.12897	18.2743	13.061	86.8964	55.2084	16.07
Slc36a2	2.08658	0.02002	0.14233	0.71435	0.43893	3.1727	2.38741	0.65342
Rap1gds1	2.08483	0.0001	0.44434	0.81209	1.01854	5.01262	4.08104	1.80547

Narf	2.08331	0.00612	0.16869	0.56123	0.49546	2.79949	2.20492	0.82978
Efemp2	2.08323	1.9E-05	0.61234	1.79133	1.82846	8.70627	7.35892	3.7483
Rplp0	2.08205	0.00015	13.6833	27.2467	26.8404	153.957	129.418	43.8562
Csf1	2.0798	2.3E-05	0.54828	1.30328	1.26251	6.65154	5.16141	2.76887
Dpagt1	2.07977	0.01135	0.20289	0.70605	0.90381	3.84334	2.16578	1.16436
Ednra	2.07936	0.03992	0.40186	1.08703	1.40599	6.79982	7.10627	2.86447
Apbb2	2.07537	2.3E-05	0.68651	0.9669	1.02057	5.42495	4.62231	2.68302
Zdhhc20	2.07533	0.02328	0.38062	0.93494	1.16612	5.04711	5.25779	1.4733
Gm9938	2.07489	0.01729	0.39574	0.74153	1.31083	4.76282	4.62656	2.17009
Rap1a	2.07452	0.00332	0.97738	2.46168	2.67926	11.326	11.8781	4.0792
H1f0	2.07152	0.0028	1.82313	1.87075	2.04766	8.87215	13.4372	5.34746
Hmcn1	2.06864	3.5E-05	0.6012	1.9268	1.90651	6.83642	7.86488	4.32418
Pot1a	2.06855	0.04528	0.16032	0.08583	0.16481	0.74664	0.72664	0.41913
Lamc1	2.06828	0.00012	1.79217	5.36329	4.70173	25.4436	20.9665	8.49566
Nod2	2.06802	0.04496	0.23099	0.20405	0.17413	1.18938	0.83364	0.70698
Col3a1	2.06695	0.00044	12.212	28.5677	29.4657	133.028	116.975	68.1988
Cpne8	2.06676	0.00191	0.44503	0.53607	0.59091	2.61982	2.55901	1.63876
Wdr75	2.06675	0.04881	0.23637	0.47453	0.27538	2.256	1.63398	0.7596
Rarres2	2.06588	0.02304	0.56689	0.73538	1.61376	5.75019	4.7434	2.10207
Sesn2	2.06571	0.00011	0.49994	1.12413	1.23342	5.77624	4.35394	3.00927
Mfap3	2.06537	0.00083	0.35938	1.21852	0.83464	5.07742	4.6683	1.57351
Nck1	2.06461	0.00791	0.43505	0.89282	0.67911	4.49227	3.88217	1.29454
Myo1f	2.06433	0.01434	1.43772	1.09778	1.14682	6.73391	8.23049	3.7872
Rps6ka3	2.06418	0.00713	0.52885	1.43056	1.64049	7.81269	7.30763	1.95941
Arhgap19	2.06371	0.00337	0.38306	0.83237	0.55592	2.94301	2.87485	2.12069
Lrp1	2.06356	3E-06	3.35357	9.93765	9.74858	49.2504	37.8675	17.7271
Lyn	2.06337	5.8E-07	1.62084	2.80876	3.33238	15.6416	13.7024	6.44038
Cep170	2.06208	3.4E-05	0.48295	0.90787	0.85752	5.05196	3.97236	1.85606
Zfp945	2.0602	0.02424	0.11385	0.16457	0.39792	1.18673	1.45833	0.5401
Tram2	2.05932	0.00086	0.71289	0.96346	1.22736	5.67747	5.6211	2.55444
Nudcd2	2.05775	0.00105	0.39471	1.04148	1.12065	4.21345	4.95849	2.54558
Cd244	2.05667	0.04688	0.19002	0.17167	0.30929	1.34236	1.32811	0.47255
Tcf4	2.05269	7.2E-07	0.78393	1.71646	1.41445	7.11841	7.13205	3.73958
Clptm1	2.0501	0.00046	0.78332	1.09334	1.07364	6.38258	5.44157	2.35465
Slc2a13	2.04933	0.03637	0.06921	0.13339	0.18973	0.80836	0.74583	0.28598
Msn	2.04298	4.8E-05	6.79136	13.2743	12.382	74.347	57.9036	23.4052
Mmp2	2.04279	0.0006	2.40503	7.11843	5.58036	32.5121	25.3229	11.3832
Asah2	2.03946	0.00946	0.27211	0.55622	0.44077	2.64064	2.28846	0.9464
Fem1c	2.03923	5.8E-05	0.5259	1.33194	1.42777	6.42673	6.33335	2.46082
Zfp280c	2.03805	0.00739	0.13272	0.31974	0.3335	1.44452	1.60783	0.55853
Ston1	2.03748	9.9E-05	0.40989	1.5018	1.10606	6.20932	4.81805	2.62667
Slc37a2	2.0374	0.03707	0.26152	0.23802	0.35845	1.69691	1.74585	0.61366
Bud31	2.03731	0.0257	0.43189	1.04049	1.33193	5.83075	4.97607	1.59726
Rap1b	2.03465	0.00359	3.31893	7.9039	6.27454	38.9143	32.3443	11.4278
Slc39a13	2.0319	0.00278	0.26166	1.24164	0.9618	4.09262	3.76032	2.09311
Plxdc2	2.02867	0.01096	0.68354	2.27601	1.99946	10.5629	8.97434	3.08711
Spns2	2.0282	0.00084	0.93608	1.48067	1.53575	5.64639	6.54607	5.16573
Pla2r1	2.02796	0.00111	0.12042	0.38681	0.43738	1.88281	1.49963	0.91219
Uhrf1	2.02733	0.04173	0.27194	0.50228	0.67091	2.49308	2.64016	1.41493
Nav1	2.02659	1.8E-07	0.71394	1.38092	1.49413	6.48833	6.04439	3.50089
Capns1	2.02557	0.04292	2.01059	4.994	2.98009	26.6301	17.9442	4.32906
Hsd17b7	2.0255	0.00978	0.15884	0.29156	0.34212	1.52463	1.40719	0.67716
Arhgdia	2.02532	3.7E-05	5.38575	11.7805	13.4983	68.9579	53.6829	20.5264
Esyt1	2.0252	2.4E-06	2.79574	5.42361	5.48959	26.8063	21.8752	11.5373
Pcna	2.02515	0.01853	0.5791	1.43388	1.88511	9.3938	7.62874	2.28936
Mtmr2	2.02512	0.00589	0.40076	1.1548	0.90472	4.95894	4.79311	1.55127

Ebf1	2.0225	0.02959	0.35615	0.44807	0.29289	1.93072	2.22065	1.03554
Golga7	2.02046	0.00108	0.25829	0.95415	1.05028	3.96826	3.86874	1.79191
Vwf	2.01932	9.1E-05	1.93224	6.49046	6.8137	26.7472	24.7374	13.6263
Birc3	2.0182	0.00023	1.10987	1.29978	1.25184	6.41745	6.19389	3.27307
Papss2	2.01787	0.02328	0.22304	0.94672	0.48039	3.68133	3.04479	0.87767
Fgd3	2.01732	0.01306	0.50088	0.57241	0.38287	2.50405	2.76143	1.42984
Coro1b	2.01342	0.00033	2.09959	3.33294	3.37971	19.316	16.8771	5.71625
Rnd3	2.01222	0.00048	0.33972	1.21717	0.87754	4.87358	4.17094	1.91958
Hip1	2.01115	1.1E-06	0.86855	1.66143	1.65193	8.00307	7.33163	3.73359
Rnf115	2.01072	0.04084	0.36031	0.73944	1.07001	4.85177	4.06992	1.25594
Rraga	2.01058	0.0109	0.85898	1.83948	1.61886	8.18058	8.36861	3.17676
Raph1	2.00929	2.1E-05	0.58369	1.64056	1.89636	8.19909	6.26349	3.68046
Mpp1	2.00891	0.01172	0.23844	0.6963	0.5348	3.14426	2.77664	0.86249
Hspa13	2.0079	0.0362	0.1965	0.71011	0.53867	3.14645	2.47711	0.94725
Plscr3	2.00788	1.5E-05	1.43486	2.51135	2.26924	10.2574	11.3516	6.53112
Rps13	2.00773	0.03287	2.53933	4.38948	3.80495	24.3232	17.5892	5.20329
Nlrp3	2.00671	0.04782	0.11419	0.32096	0.23477	1.11142	1.24795	0.61163
Slc43a2	2.00574	1.5E-05	0.73814	1.14848	1.46226	6.15587	6.1146	3.02697
Bcl2	2.00524	4.5E-05	0.26561	0.68053	0.87547	3.13715	3.42671	1.82912
Fkbp3	2.00521	0.03312	0	1.17857	1.17341	5.29895	4.84896	1.68445
Rhoc	2.00453	0.00123	2.79169	4.36876	4.75845	28.8568	18.6292	8.7087
Stx12	2.00309	0.00024	1.05356	2.0714	2.21587	9.74093	9.25174	4.97587
Tmem106a	2.0021	0.02114	0.47981	0.77063	0.63235	3.91674	3.37694	1.32733
Plxna3	-2.0006	0.00239	0.30866	0.7436	0.49356	0.65527	0.67239	0.37113
Safb	-2.00112	0.00813	6.19122	12.0971	10.3371	11.2296	8.75954	10.8173
Il17rc	-2.00202	0.00514	1.43094	3.44734	3.80372	3.07677	2.82124	3.15199
Kiz	-2.00229	0.00022	1.47443	4.04208	3.03136	3.86298	2.99784	2.45088
Clec3b	-2.00271	0.02772	1.9848	7.12566	8.24135	9.01747	5.99396	3.75208
Fuom	-2.00275	0.03435	0.31857	0.59694	0.50942	0.51122	0.68371	0.3521
Nudc	-2.00402	4.8E-05	5.68936	16.4478	14.0365	19.5468	12.0221	7.83592
Echdc2	-2.00439	0.00603	1.87377	3.33481	3.71356	3.08458	3.11475	3.29372
Gpam	-2.0068	8.8E-07	2.53883	6.40769	7.24962	6.8513	6.08378	4.41715
Ncs1	-2.00835	0.00215	0.72185	1.51916	1.46703	1.4067	1.36645	1.21894
Cplx2	-2.00861	0.0009	0.51705	1.5665	1.27242	1.03304	1.54107	0.98907
BC022687	-2.01011	0.03363	1.39104	2.18558	1.94806	1.48111	2.1827	2.2213
Dnaaf5	-2.0103	0.00088	1.02174	3.25557	2.84604	2.46284	2.61353	2.06798
Arhgap33	-2.01127	0.03013	0.36899	0.90638	0.80325	0.59969	0.79437	0.78058
Sclt1	-2.01147	0.00037	0.44087	1.15392	1.37641	0.97406	1.31969	0.87459
Galnt15	-2.01198	0.00324	0.53094	1.63199	1.55584	1.49453	1.59137	0.89657
Nipal3	-2.01316	2.7E-06	1.57214	3.15013	2.43726	3.48871	2.5603	1.93872
Cc2d2a	-2.01318	5.6E-05	1.02058	1.89869	1.7369	1.53805	1.96267	1.50976
Met	-2.01355	5E-05	0.75413	1.67147	1.97425	2.29193	1.70197	0.90017
Armc9	-2.01929	0.00261	0.32949	0.72942	0.72623	0.65983	0.80119	0.53829
Jph2	-2.02117	0.00399	2.3681	5.11493	3.48831	4.37554	3.69416	3.60314
Cetn2	-2.02235	0.00039	1.97019	4.68999	4.33996	4.02071	4.61385	3.17656
Mmaa	-2.02382	0.00168	0.26921	0.52504	0.51395	0.54336	0.62556	0.25013
Nr6a1	-2.0266	0.00401	0.26751	0.56713	0.53531	0.51736	0.5439	0.40937
Filip1	-2.02743	0.00397	1.04526	2.15323	1.61954	1.68368	1.77873	1.63436
Nacad	-2.03032	0.00288	0.4735	0.79852	0.68145	0.69872	0.79426	0.61592
Bok	-2.03036	0.00341	1.13301	3.33618	3.58041	2.88533	3.03955	2.55266
Slc16a12	-2.03182	0.0025	0.48603	1.42348	1.58707	1.14897	1.67124	0.89692
Nek4	-2.03218	0.00681	0.42055	1.65484	1.52751	1.58445	1.38948	0.90101
Jag1	-2.03245	4E-06	1.94788	5.04389	6.21144	5.17958	4.53634	3.90861
Rdm1	-2.03245	0.00491	0.95597	1.77159	1.91503	1.84702	1.7568	1.46294
Fuz	-2.03364	0.00233	0.76395	1.85906	1.53891	1.39551	1.70624	1.27537
Slc16a13	-2.03408	0.01368	0.40076	1.03701	0.97652	1.06251	0.95063	0.61329

Lztfl1	-2.03468	0.0442	0.59159	0.99863	1.09217	1.1296	1.27648	0.81295
Azin2	-2.03807	0.0013	0.92103	1.64106	1.77524	1.30126	1.78487	1.49756
Slc2a12	-2.03847	0.00638	0.51937	0.81221	0.843	1.04704	0.70862	0.56473
Grtp1	-2.03873	0.00262	0.62253	0.81806	1.18293	1.2971	1.16506	0.73616
Syde2	-2.04151	0.00022	0.70486	1.32615	1.38016	1.63131	1.13628	0.95539
Ring1	-2.04158	0.0019	1.5714	2.6959	2.81464	2.57894	2.48701	2.43208
Otud1	-2.04343	0.00057	0.94062	3.18568	3.30721	3.15866	2.39061	2.22177
Tbcel	-2.04388	2E-07	2.50149	5.72515	5.12867	4.9479	5.07548	3.98761
Itgb4	-2.04391	0.00019	1.25478	3.43396	3.85833	3.01687	2.68551	2.7919
Slc35e3	-2.04682	0.00089	1.33049	3.89985	3.16854	3.11206	3.02641	2.72326
Greb1l	-2.04688	0.04054	0.05363	0.25841	0.16907	0.229	0.19627	0.09028
Ankrd54	-2.0486	0.00017	0.94934	1.65542	2.23062	1.83945	1.77855	1.53581
Spata7	-2.04865	0.01641	0.17465	0.52094	0.46167	0.50134	0.4312	0.32455
Rmdn2	-2.05094	0.02924	0.25709	1.0839	1.4976	1.57399	1.01277	0.54097
Pacsin3	-2.05171	0.00421	0.7227	1.63362	1.22902	1.31099	1.18369	1.24933
Hdc	-2.05363	0.00087	0.70699	2.11085	2.0747	2.49289	1.66782	1.05418
Agbl3	-2.0601	0.00676	0.24805	0.3569	0.36832	0.28556	0.373	0.37169
Dnal4	-2.06402	0.00135	1.33638	2.42458	2.34048	2.05125	2.59134	1.81786
Scrn3	-2.06429	8.2E-05	0.45702	1.12035	1.08798	1.31406	1.06372	0.49694
Mipep	-2.06483	0.00018	1.20494	2.36102	2.01488	2.60277	1.95993	1.43829
Zcchc3	-2.06627	0.0026	0.86685	1.69492	1.26563	1.44364	1.37935	1.26889
Eps8l1	-2.06628	0.02327	1.51586	2.77362	3.87923	2.85555	1.71659	3.23004
Pttg1	-2.07159	0.00013	1.38983	2.92977	2.69868	2.65451	2.973	1.81672
Tob1	-2.07181	2.5E-06	2.73349	7.84557	6.76474	8.42327	5.68644	4.18314
Ifrd2	-2.07218	0.00021	1.53723	2.43367	2.04678	2.17875	2.52274	1.71394
Lyrm4	-2.07306	0.04588	0.30579	1.10505	1.205	1.05609	1.20463	0.52646
Whrn	-2.07365	0.02002	0.25257	0.60848	0.51927	0.61456	0.42527	0.41067
1700112E06Rik	-2.07395	0.03163	0.46942	0.82248	1.14058	0.72351	1.01951	0.81633
Syne1	-2.08101	4.8E-07	4.92325	10.2772	8.54049	8.04472	8.25317	7.78302
Ppm1l	-2.08183	0.00962	0.1987	0.91986	0.82505	0.81467	0.79612	0.38457
Trim65	-2.08357	0.00198	0.68905	1.80231	1.38967	2.64549	1.47103	1.20511
Cdpf1	-2.08404	0.04698	1.04857	2.21996	1.43721	2.63371	1.40516	1.02114
1110051M20Rik	-2.08667	0.00048	0.37849	1.09064	1.20539	1.41202	1.36936	0.74958
Prkaa2	-2.08826	0.00173	0.45055	1.72735	1.47411	1.47844	1.34453	0.87853
Nav2	-2.08948	4.2E-08	1.06576	2.18392	2.04949	2.17786	1.88205	1.46928
Tspyl2	-2.09401	2.1E-05	3.03923	4.84832	4.53158	4.56473	4.63455	3.86536
Naa16	-2.09723	1.2E-05	0.90469	1.80114	1.30458	1.60956	1.47538	1.12488
Acaa1a	-2.09915	1.1E-05	2.83382	6.40567	6.49154	6.60698	6.06852	4.89883
Kcnq1	-2.09931	0.00028	0.9338	1.34071	1.5126	1.58139	1.48149	0.97433
Nt5dc3	-2.1049	1.7E-07	1.0897	2.48461	2.05367	2.26048	2.12657	1.45914
Ttc8	-2.1054	0.00118	0.6741	1.21801	1.35705	1.21695	1.41343	0.7737
Mib2	-2.10684	0.00019	2.30462	4.44961	4.70458	3.87955	3.76358	3.84573
Rhobtb1	-2.10873	0.00013	0.7054	1.77597	1.5548	1.52037	1.25011	1.3129
Ears2	-2.10909	0.00045	0.29225	0.64991	0.77031	0.71992	0.71989	0.36123
Ttll5	-2.10914	1.9E-06	0.35112	0.81654	0.7269	0.81761	0.70275	0.45897
Smyd3	-2.11391	0.00069	0.44138	0.93171	0.65683	0.70477	0.73717	0.63686
Mblac2	-2.116	0.00886	0.17906	0.69499	0.634	0.56217	0.68261	0.31968
Ikbkg	-2.11776	2.4E-07	1.38526	2.92444	3.22973	2.5378	2.9109	2.18546
Ybey	-2.11853	0.02608	0.17937	0.4527	0.61461	0.83667	0.67728	0.37252
Txlng	-2.11927	9.9E-07	0.81401	2.20774	2.64191	2.41103	1.92829	1.31621
Mtcl1	-2.1199	0.00025	1.67455	4.27511	4.12794	3.10742	3.0236	3.471
Acta2	-2.12102	0.00418	16.0625	49.0371	45.1904	62.4768	34.9394	18.3113
Sgms2	-2.1227	0.00197	0.72538	1.34427	0.70106	1.08616	1.18178	0.66889
Baiap2l1	-2.12429	0.0009	1.54838	3.15003	2.3345	3.56501	2.25621	1.685
Lynx1	-2.12519	9.3E-06	1.32898	2.81569	1.99595	2.41474	2.21342	1.70923
Arrdc4	-2.12536	9.5E-08	1.83521	4.51342	4.08754	4.14138	3.81884	2.72071

Arl6	-2.12721	0.00334	0.66245	1.15263	1.13498	1.17859	1.23467	0.63361
Inmt	-2.12938	0.0442	9.41031	27.9026	31.3523	41.7721	20.1266	10.3021
Des	-2.12994	0.00037	9.40992	17.5165	15.6885	18.8306	12.7027	12.3701
Pfkfb2	-2.13017	0.0004	1.58012	3.98803	4.25419	4.12237	3.81511	3.5264
Mcee	-2.13026	0.00543	0.84805	3.79429	3.15499	3.42331	3.14063	1.48319
Lncpint	-2.13113	6.2E-05	3.19589	6.98078	5.30029	5.89998	5.00341	4.79294
Fgfr3	-2.13191	0.00026	2.75067	9.29047	10.0685	8.80883	4.92651	6.05413
Arhgap32	-2.13238	1.6E-06	0.627	1.28855	1.31218	1.18113	1.29538	0.83054
Ppl	-2.13405	0.00071	2.32487	7.34942	8.0296	6.14066	4.61442	5.68967
Tprn	-2.13444	0.00542	1.06655	1.96676	1.00164	1.41385	1.56615	1.17879
P4ha2	-2.13616	0.00069	0.89106	3.80144	2.65891	3.81174	1.84002	1.63349
Efcc1	-2.13861	0.01772	0.34291	0.64255	0.47001	0.43065	0.53744	0.48104
Cgn	-2.13866	0.00031	4.59737	8.89769	10.0452	7.13855	7.59725	7.93331
Uhrf1bp1	-2.13901	1.4E-06	1.06403	2.67021	2.55219	2.33011	2.29184	1.67919
Glis3	-2.14017	3.3E-06	0.69423	1.88289	1.55299	1.52987	1.4315	1.09247
Taf1a	-2.14786	0.00181	0.96155	2.66401	2.2405	2.08307	1.75954	1.98651
Trappc6a	-2.14985	0.00489	1.60354	2.86811	2.99709	2.70034	2.66755	2.23085
Dsp	-2.15089	1.4E-06	2.3224	5.3631	4.23048	5.20606	3.74942	3.03842
Atn1	-2.15091	0.00017	3.75153	7.98661	6.66043	10.7983	8.43396	7.45569
Ift74	-2.15141	5.7E-05	0.91574	2.52132	2.12179	2.38222	2.32737	1.00099
Ank3	-2.15326	1.4E-06	0.61103	1.48788	1.31028	1.29214	1.058	0.9954
Pygb	-2.15327	1.2E-08	3.56441	7.19468	5.74377	6.82045	5.83939	4.29374
Prpf40b	-2.15341	0.00207	1.75413	3.53102	3.63846	2.40854	2.8651	3.23916
Safb2	-2.15478	0.01111	6.86546	11.0038	9.99331	8.52407	7.91681	10.4847
Atp6v0e2	-2.15767	0.0011	0.77829	1.40626	1.16676	1.13014	1.40935	0.85606
Firre	-2.1599	0.00259	3.63734	7.33062	6.83337	4.50626	5.81075	6.46897
Ltbp4	-2.16075	0.00135	5.91371	28.1055	27.6817	23.4506	17.717	15.3986
Iqcb1	-2.16116	0.03774	0.1075	0.60429	0.66304	0.66152	0.78685	0.64158
Abhd14b	-2.16164	0.01414	0.52034	1.6018	1.96129	2.04211	1.74123	1.32715
Osgin1	-2.16356	0.00163	0.77964	2.74782	2.70117	2.55657	2.02549	1.5908
Rab3ip	-2.16368	2.3E-05	1.29166	3.63045	2.27201	3.35244	2.36361	1.61428
Calcoco1	-2.16641	6.1E-08	2.45491	6.38364	6.44918	5.89077	5.22905	4.0028
St3gal5	-2.168	4.4E-05	1.32719	3.01976	2.668	2.69459	2.72094	1.69252
Sel1l3	-2.16997	0.00567	0.13485	0.39256	0.32345	0.4128	0.25704	0.20636
Fam216a	-2.17054	0.01378	1.33509	3.75251	2.91644	3.24858	2.75694	1.85158
Dusp14	-2.17137	0.00018	1.0123	2.34315	2.69338	2.28087	2.54252	1.32133
Tmem205	-2.17166	0.01834	1.06557	3.29118	2.58397	3.8602	2.16653	1.12892
Lamc3	-2.17189	0.00142	0.45636	1.84765	1.74618	1.50443	1.07285	1.1494
Rnf8	-2.17272	0.03113	1.31199	2.22425	1.79825	1.89939	1.83394	0.94811
Auts2	-2.17445	3.3E-05	0.44326	0.88416	0.81333	0.68237	0.72317	0.68927
Slain1	-2.17865	0.02884	0.84643	0.79301	0.72509	0.81939	0.8067	0.86354
Tchp	-2.17987	0.03675	1.47368	2.48764	2.18477	1.68077	1.83447	2.27822
Kank1	-2.18104	9.4E-07	1.12701	3.52968	3.14093	2.81894	2.69246	2.05526
Sh3yl1	-2.18148	0.02756	0.27198	0.65525	0.71451	0.797	0.48387	0.41622
Itgbl1	-2.18288	0.01353	0.24648	0.6378	0.76952	0.85985	0.68212	0.16764
Pla2g16	-2.18375	0.00017	0.52513	1.09063	1.06724	1.22802	1.03089	0.4988
Dnaja1	-2.18456	0.00176	3.90583	17.0302	10.6517	14.4795	7.08175	5.55612
Cspp1	-2.18511	3.5E-05	1.08044	2.95155	2.41305	2.10801	2.1036	1.91177
Nphp1	-2.18718	0.00143	0.98369	3.19417	3.22416	2.57818	2.28268	2.22533
Gm35339	-2.18722	0.01276	0.31856	0.29237	0.45222	0.40007	0.37011	0.31339
Dhx16	-2.18759	0.00204	4.07376	9.13275	7.92954	6.91023	6.0108	7.0351
Nrxn2	-2.18797	0.01228	0.1174	0.19528	0.18964	0.17376	0.15771	0.21173
Thbs3	-2.19048	2E-05	1.27274	3.25906	3.32435	2.8047	2.0995	1.96608
Per3	-2.19453	0.00023	1.89255	5.03377	4.83276	3.3835	4.03791	3.52342
Spag1	-2.19716	0.00042	0.42514	0.95106	1.20704	0.95341	0.9107	0.74354
Sec14l1	-2.19941	3.3E-10	5.56823	13.661	13.0629	12.0906	10.78	8.38536

B230118H07Rik	-2.19966	0.00336	0.27004	0.52045	0.48116	0.47478	0.52159	0.26861
Nek1	-2.19987	3.4E-06	0.71965	2.005	2.08346	1.63233	1.62743	1.35976
2210408I21Rik	-2.20029	0.00101	0.48279	1.30596	0.9926	0.79779	1.22702	0.69994
Eml2	-2.20057	4.5E-05	2.03479	6.29897	5.26304	4.43076	4.53755	3.86726
Usp20	-2.20149	5.7E-05	0.79488	1.2266	1.30312	1.03741	1.29927	0.97791
Adi1	-2.20494	0.00165	0.92115	3.45207	3.36683	3.43194	2.37233	1.87955
Slc1a5	-2.20589	2.7E-09	4.71825	11.1887	10.4994	10.3347	8.46461	6.92315
Foxa2	-2.2097	0.00621	0.48869	2.6686	3.617	2.94587	2.08658	1.45831
Rfx3	-2.21281	2.8E-05	0.56681	1.61842	1.40273	1.10071	1.34453	0.97182
Gna14	-2.21348	0.00036	1.16401	1.29217	1.6189	1.46183	1.6358	1.04783
Cep164	-2.21708	0.00397	2.06949	3.54462	3.71921	2.38998	3.01602	3.3331
Esrp2	-2.22068	1.5E-07	1.44107	3.30031	3.36512	2.81521	2.45523	2.3074
Ffar4	-2.22126	0.01088	1.08087	2.27013	3.98859	2.29717	2.19764	2.54474
Ephb2	-2.22188	0.00051	0.11942	0.29508	0.21405	0.25957	0.21012	0.15464
Sh3bgrl2	-2.22502	3.1E-06	1.0705	1.99747	1.75857	1.55199	1.70444	1.44549
Ccdc9	-2.22722	0.03823	4.41657	5.89432	6.44266	4.0807	5.12064	6.59786
Zfr2	-2.22824	0.00259	0.29408	0.52203	0.43489	0.43735	0.44057	0.35529
Fos	-2.22846	0.04615	4.43186	12.1006	5.602	6.86447	6.9334	6.89522
Tshz2	-2.22914	0.03636	0.65493	1.30291	0.79567	1.87868	1.19543	0.94532
Gnb5	-2.23308	0.00514	0.66926	1.73178	1.09568	1.05062	1.30405	0.93884
Cep78	-2.23452	0.00097	0.68182	1.13158	1.27719	0.9419	1.29388	0.79995
Ift122	-2.23455	0.00038	0.85954	1.35711	1.32787	1.11605	1.17743	1.14817
Myocd	-2.23455	0.02441	0.41467	0.75287	0.45716	0.89436	0.48571	0.3035
Rgma	-2.23519	0.00025	0.45802	1.22606	1.44391	1.45721	1.11752	0.56074
Cbarp	-2.23854	4.1E-05	0.60274	1.8989	1.44574	1.45569	1.43761	0.97561
Wdr19	-2.23886	4.7E-06	0.32862	1.11333	0.86566	0.84475	0.79344	0.57362
Pgm5	-2.23925	0.00225	1.02637	2.73025	1.9563	2.49738	1.85858	1.25163
Rgl3	-2.24137	0.00118	0.82644	1.44585	1.33502	0.98229	1.15522	1.30989
Wwc1	-2.24324	1.8E-07	5.95441	15.8827	15.8906	12.1553	11.3232	10.9704
Arhgef4	-2.24758	0.01843	0.50878	0.71987	0.41509	0.45639	0.49875	0.63956
Ppp1r14c	-2.24764	4.9E-05	2.61113	4.40698	3.79122	3.55711	3.01443	2.67521
Fam184a	-2.24933	0.00267	0.3991	0.641	0.78407	0.85206	0.43278	0.54968
Dync2li1	-2.24947	0.00258	0.69981	2.88018	2.45792	2.69153	2.08984	1.13788
1700030K09Rik	-2.25352	0.00547	0.66294	1.09606	0.98882	0.88153	1.11003	0.62661
Trim45	-2.2538	0.04054	0.13512	0.40304	0.30426	0.31515	0.32379	0.17724
Cmbl	-2.25532	0.00309	0.94315	2.6509	1.80981	1.65825	2.1094	1.44333
Slc44a3	-2.25544	1.2E-05	0.59547	1.76564	1.88348	1.5388	1.32714	1.19165
Tcte2	-2.25735	0.01614	0.20295	0.23904	0.19472	0.2209	0.24071	0.23812
B9d1	-2.25946	0.00787	0.58164	1.45965	0.9467	1.59788	0.88695	0.44505
Cep290	-2.2661	2.8E-06	1.50452	3.06698	3.0734	2.41633	2.61781	2.15851
Dnaaf2	-2.26849	0.00198	0.75163	3.10029	2.71601	1.60899	1.64979	1.77764
Bcas1	-2.2736	0.01709	0.46021	1.05944	0.58873	0.57796	0.77975	0.49299
Mob3b	-2.27432	9.4E-09	1.02743	1.63515	2.06114	1.77124	1.87059	1.00055
Cnksr1	-2.27988	0.0051	0.74349	1.55899	1.98149	1.42651	1.19674	1.39062
Abcb6	-2.28207	0.00601	0.58836	1.29495	1.22457	0.90309	0.9415	0.58358
Pomgnt2	-2.28287	0.01727	0.19948	0.5767	0.92962	0.77661	0.48671	0.42738
Thnsl1	-2.2861	0.00034	0.60158	1.01221	1.29572	1.02533	0.94648	0.83294
Tef	-2.28863	3.6E-07	3.40168	11.4236	12.6755	8.95092	8.98976	6.60288
Wdr90	-2.29064	0.00338	1.17652	2.17526	2.03448	0.96212	1.31428	1.61204
Agmo	-2.29232	0.00039	0.27642	0.59194	0.78299	0.61328	0.62759	0.35251
Tox3	-2.29425	0.01179	0.58669	0.47114	0.61493	0.60677	0.6666	0.36969
Cacnb1	-2.29513	0.00064	0.76718	1.15003	1.13916	0.89924	1.16989	0.86096
Wdpcp	-2.29777	0.00195	0.39036	0.721	0.89619	0.68633	0.64487	0.58244
Cep128	-2.30091	0.0064	1.06916	2.01408	1.78043	1.46193	1.32055	1.63617
Fam89a	-2.30338	0.0257	0.69249	1.11221	1.48305	0.76096	1.36398	0.9935
Cbr3	-2.30423	0.00024	1.18789	3.25928	3.25633	2.85932	2.91841	1.59063

Cyb5a	-2.3082	4E-05	4.26958	11.6315	10.7781	13.5068	8.15902	4.25941
Fam173b	-2.30928	5.4E-06	0.61794	1.41781	1.07383	0.97005	1.27884	0.69235
Zfp704	-2.31126	0.00038	1.54122	2.99969	2.73162	4.05703	3.89774	2.83493
Igfbp5	-2.31414	1.8E-09	7.32789	18.8154	14.2596	14.0221	14.7815	9.03027
Stx1b	-2.31502	0.02341	0.28943	0.44548	0.38017	0.31804	0.44135	0.29528
Ppp1r9a	-2.31521	1.2E-07	0.9724	2.57308	2.40342	1.75672	1.8515	1.70764
Bbs5	-2.31538	0.01549	0.18948	0.79885	0.84405	0.65439	0.69827	0.3987
Spef1	-2.31671	0.00012	2.61405	5.04488	4.21858	3.41525	2.97893	3.64548
Intu	-2.318	0.00093	0.45218	1.23961	1.07925	0.69515	0.86389	0.90673
Col11a2	-2.31923	0.03225	0.40692	0.61271	0.46478	0.46845	0.31888	0.5838
Thrb	-2.32004	3E-05	0.40638	1.06417	0.79918	0.83211	0.73645	0.56782
Osbpl3	-2.32017	8.3E-05	1.59202	2.95904	1.90932	1.91873	2.44074	1.77811
Mttp	-2.32231	0.00294	0.13064	0.43462	0.47322	0.45703	0.3415	0.17136
Gpld1	-2.32471	2.3E-05	0.64631	1.79062	1.43952	1.60306	1.05127	1.0138
Tmem30b	-2.32659	2.6E-05	0.59992	1.92705	1.62035	1.59546	1.56057	0.75606
Megf6	-2.32697	3.6E-05	2.05848	4.86474	5.72348	3.47151	3.04185	4.27521
Prr15l	-2.32716	0.01096	2.50946	7.70528	8.49774	4.93122	3.82671	4.72131
Noxo1	-2.32766	0.02061	0.49972	0.70999	0.57956	0.62757	0.66433	0.4706
1110065P20Rik	-2.32872	0.04882	1.07932	0.93342	1.47935	1.30334	1.22385	1.46113
Pex7	-2.33018	2.4E-06	0.40081	0.89901	1.02295	0.81578	0.8079	0.56049
Car8	-2.33032	3E-06	3.66726	5.47962	5.91229	5.07743	4.96579	4.23207
Selenbp1	-2.33493	0.00056	9.88514	42.4329	59.0147	41.8184	28.4644	22.6522
Pms1	-2.33834	0.03516	0.34159	0.36576	0.45519	0.26216	0.47267	0.37754
Cap2	-2.34033	0.00952	0.31378	0.78394	0.66901	0.67866	0.47848	0.50686
Prkcz	-2.34182	3.6E-06	0.91658	2.50261	2.57541	1.92233	1.95679	1.57099
Phf1	-2.34201	0.00013	5.19732	8.50058	8.49608	5.53905	6.54395	7.77117
Grk4	-2.34342	0.04353	0.52765	1.00154	0.95333	0.70783	0.88996	0.95429
Stard10	-2.34384	9E-05	3.20789	6.49792	5.28278	4.20904	4.25898	3.40707
Camkk1	-2.34423	0.00166	0.24854	1.09774	1.29875	0.87786	0.82117	0.73692
Lzts3	-2.34501	0.00769	0.65373	1.42182	0.87736	0.80389	0.88612	0.97958
Polr3g	-2.34524	0.01814	0.17655	0.42534	0.42348	0.42498	0.26923	0.2927
Fam160a1	-2.34593	0.01594	0.63493	1.63512	1.42541	1.37479	1.11288	0.88788
Fanca	-2.3539	0.0062	0.50235	0.81745	1.23213	0.65579	0.78621	0.63727
Acss2	-2.35614	0.00052	0.27351	0.75044	1.36675	0.85156	0.63141	0.49704
Krtcap3	-2.35658	0.00145	2.6834	3.89274	3.97458	2.71772	3.29996	3.64285
Ppp1r16a	-2.35776	6.7E-05	2.95242	6.39025	6.59033	4.60829	4.35925	4.96998
Shh	-2.35997	0.01011	0.97635	1.63345	2.0352	0.76635	1.32342	1.30736
Slc24a3	-2.3606	1.9E-07	1.06582	2.11939	1.77389	1.75282	1.71558	1.16504
Spice1	-2.36079	0.00018	0.57739	1.32232	1.28966	1.32938	0.82611	0.80177
Mn1	-2.36227	1.2E-05	0.55073	1.80925	1.3267	1.44614	1.15367	0.77894
Bik	-2.36316	0.0086	1.10538	2.2685	2.69346	1.3882	2.49719	1.69159
Sgsm3	-2.36481	0.00018	1.80718	3.88425	3.40586	2.98715	2.59369	2.6029
Aldh1a1	-2.36689	0.00036	24.8224	67.1402	66.8465	75.5602	46.1966	26.6768
Agbl5	-2.3677	1E-05	0.78822	2.00444	1.80061	1.39204	1.47742	1.33188
Iqce	-2.36788	7.6E-05	0.99106	1.91627	1.84634	1.25067	1.55529	1.4283
9530026P05Rik	-2.37027	0.00209	0.24171	0.46909	0.41412	0.48063	0.32763	0.29283
Cdr2	-2.3742	3E-05	1.95669	4.58022	3.48077	5.07149	2.68751	1.78388
Bcl7a	-2.37537	6.2E-08	0.69565	1.30349	1.27967	1.0269	1.2283	0.73773
Nr3c2	-2.37801	2.7E-05	0.37569	1.04875	0.93178	0.82005	0.78204	0.50648
Tmem67	-2.38784	0.00012	0.34399	1.14099	1.04546	0.93913	0.86665	0.86973
Mettl26	-2.38787	0.03347	0.73105	2.43215	3.2923	2.55755	2.38883	1.91785
Bbs2	-2.38845	0.00034	0.62964	1.32027	0.98287	1.16415	0.80012	0.74943
Agpat9	-2.39	0.00946	0.19843	0.69053	0.56663	0.47764	0.65562	0.32897
Ckmt1	-2.39009	0.00044	2.08809	3.09284	2.2578	2.88457	2.38221	1.81076
Dyrk1b	-2.39768	6.8E-07	2.14878	3.79627	3.94656	3.83196	2.79612	2.49913
Slc4a4	-2.39769	3.4E-06	0.46049	1.0578	1.26233	0.89437	0.91995	0.68012

Mmp17	-2.39891	0.00648	0.13998	0.26698	0.21585	0.23074	0.20457	0.13389
Cys1	-2.39893	0.00057	0.9192	2.39024	1.73985	1.869	1.15696	1.50715
Setd4	-2.39899	0.0209	0.39411	0.45496	0.27009	0.26294	0.40066	0.28271
Rbks	-2.39941	0.02991	0.62227	0.62464	0.79959	0.928	1.02797	0.41662
Zfp467	-2.39974	3.7E-08	1.33376	3.2788	2.77945	2.71762	2.19989	1.79116
AI661453	-2.40304	1.6E-06	1.30571	2.90652	2.33912	2.89121	1.93288	1.10425
BC039771	-2.40443	0.00089	0.49149	0.92752	1.04416	0.77155	0.72449	0.71453
Ccdc85b	-2.40935	0.00197	7.03663	13.8978	11.8603	8.00108	8.02351	12.078
Igfbp6	-2.41169	0.00624	1.56231	7.02583	7.28061	9.35238	3.5471	1.75328
Cyp4b1	-2.41273	4.6E-05	6.36595	24.9837	29.413	23.4362	15.1615	12.9238
Ica1	-2.41739	1.1E-05	1.60711	2.26571	1.93354	1.90618	2.12394	1.36634
Coq4	-2.41854	0.00209	0.2726	0.76618	0.6305	0.81306	0.41568	0.33894
Wfdc1	-2.42264	0.00094	0.97833	2.41738	2.37242	2.5518	1.53012	0.97891
Zfp69	-2.43116	0.01277	0.791	0.90267	0.97005	0.81039	0.86762	0.76452
Tmem184a	-2.44015	5.8E-07	0.48285	1.16327	1.2033	1.11632	0.94828	0.62137
Ptms	-2.44114	0.01049	79.6928	122.594	115.358	87.8195	73.4528	111.829
Plekhb1	-2.44641	3.1E-06	0.96698	4.33356	4.12577	3.17308	2.75885	2.10035
Fam84b	-2.44886	3.1E-08	4.35837	10.7825	9.69727	5.81712	7.75878	6.05659
Rmdn1	-2.44946	0.0281	0.53101	1.80047	1.2939	1.25965	1.28959	1.08349
Tmod1	-2.4506	2.5E-06	0.99794	2.33347	2.35343	3.44648	1.90966	1.50471
Gprc5c	-2.45062	1.4E-05	1.05993	3.28057	2.1489	1.67776	2.23363	1.63893
Cftr	-2.45671	0.03558	0.07458	0.2156	0.20444	0.20605	0.18954	0.05706
Slc25a33	-2.45733	0.00073	0.67515	1.28152	1.30395	1.19476	1.12314	0.70446
Slc4a3	-2.45935	0.01991	1.01017	1.79549	1.52303	0.76118	0.96533	1.75201
Cldn3	-2.46223	4.1E-07	6.80748	18.3949	18.2829	18.5426	12.2512	7.95403
Map1b	-2.46338	4.4E-09	2.24759	6.08966	4.67451	5.16668	3.45718	2.78902
Arhgap39	-2.46386	2.4E-06	1.28915	2.75226	2.58579	2.13233	2.20558	1.5157
Muc20	-2.46591	0.01337	0.54645	0.76218	0.7687	0.66821	0.42396	0.35211
Gsta4	-2.46771	7.5E-05	3.38492	9.40372	9.1536	8.15729	6.69627	4.41003
Kctd14	-2.46844	0.03315	0.81182	0.68815	0.92725	0.5664	0.87115	0.75921
Mapk12	-2.47089	8.5E-08	2.51381	3.64354	4.11781	3.58049	3.1477	2.53336
Rpusd3	-2.47234	0.04357	0.53645	1.07699	1.10291	0.37054	0.81804	0.65675
Prkar2b	-2.47242	3.3E-05	0.95142	2.26664	1.93434	2.11089	1.75711	0.80079
Mlf1	-2.47289	0.00218	0.53901	2.11014	1.52374	1.56537	1.16441	0.7733
Cep83	-2.474	2.2E-05	3.80117	8.75265	8.08084	5.91844	5.97388	5.60927
Upp2	-2.47684	0.01707	0.09853	0.29012	0.28134	0.19592	0.24206	0.15078
Cblc	-2.47694	0.00338	0.54696	1.02489	0.93711	0.80139	0.90357	0.52314
Per2	-2.47895	2.7E-06	3.71824	11.3402	9.70475	7.51355	6.08216	6.62838
Irx3	-2.4813	0.00014	1.59346	3.97358	3.39102	3.10706	2.04622	2.31017
Rsph3b	-2.48405	0.00811	2.00925	6.68464	5.50791	5.00162	4.45	4.66713
Ccdc64b	-2.48628	0.00092	0.48435	1.16686	1.07878	0.95042	0.954	0.50958
Foxp2	-2.48882	0.00021	0.25074	0.29288	0.30722	0.26718	0.23559	0.26162
Slc16a7	-2.48893	2.5E-05	0.41088	1.68907	1.29394	1.22244	0.9647	0.68118
Trpm4	-2.49776	5.2E-06	1.00563	1.9588	2.04554	1.28309	1.45737	1.46528
Eef2k	-2.49903	1.3E-08	1.0475	2.00883	1.93932	1.44314	1.52582	1.3518
Engase	-2.50016	0.00016	0.78196	0.9509	1.04116	0.85577	0.94258	0.71799
Pnma1	-2.50347	0.01727	0.34368	1.22224	1.14399	0.92488	0.6239	0.71377
Bphl	-2.5038	8.2E-06	2.01175	6.46214	7.15604	5.65448	4.82073	3.07863
Pnpo	-2.50651	0.00043	1.22749	2.79821	2.25229	2.16314	2.25422	1.69667
Tmc4	-2.50942	3E-06	3.38257	6.62795	6.30533	4.34998	4.49788	4.14115
Hmgcll1	-2.51453	1E-06	0.51012	0.87052	1.07792	0.8008	0.86432	0.45538
Slc39a4	-2.51634	6.2E-08	5.2077	5.9385	7.24492	6.63823	6.16773	4.06443
Nat9	-2.51638	0.0002	1.40149	2.8812	1.88251	1.97128	1.68122	1.80158
Tfcp2l1	-2.51663	8.2E-07	1.04308	1.61617	1.32036	1.61049	1.37853	0.69484
Fgfr2	-2.52254	2E-10	1.99964	4.3935	4.95533	4.23901	3.32574	2.28896
Rnf32	-2.52387	0.00235	0.42285	0.61123	0.52162	0.42483	0.58033	0.32355

Ptpru	-2.52589	1.4E-06	1.90508	4.26273	3.15796	2.53565	3.12027	2.27999
Adam8	-2.53031	0.01827	6.44272	7.62037	3.18313	5.58226	5.24232	5.38831
Eya2	-2.53099	1.8E-08	1.83716	4.86545	3.32172	3.90262	2.92072	1.91418
Ryr3	-2.53206	0.03373	0.12432	0.26798	0.19282	0.12737	0.0898	0.24532
Ppp1r26	-2.5325	7.2E-07	0.47408	0.92694	0.91818	0.8154	0.71245	0.48892
Efcab5	-2.53302	0.0216	0.24671	0.23414	0.32278	0.15492	0.25081	0.29174
Zscan18	-2.53384	0.03558	0.10872	0.3929	0.39118	0.22188	0.27633	0.27037
Strbp	-2.53469	1.4E-08	0.4915	2.03344	1.77796	1.2931	1.32821	0.89496
Fermt1	-2.53875	0.0015	0.30484	0.44064	0.65459	0.26799	0.55783	0.3732
Npas2	-2.54323	0.01405	0.14505	0.27178	0.36448	0.2277	0.28262	0.16648
Ric3	-2.54743	0.00877	0.06764	0.16296	0.15761	0.11893	0.15816	0.05176
Acpp	-2.54745	0.0006	0.17381	0.43142	0.57391	0.30757	0.36945	0.33852
Cacna1h	-2.55326	0.01643	0.4508	0.63829	0.29268	0.37991	0.47034	0.38124
Cep70	-2.55396	2.9E-08	0.86756	1.66432	1.55245	1.16519	1.37195	1.01418
Epha1	-2.55469	1.7E-07	0.73714	1.39702	1.49532	1.3701	1.25896	0.72196
Cckar	-2.56189	0.00094	0.97512	1.99557	2.62996	1.5999	1.23115	1.70887
Optn	-2.56276	4E-07	1.45044	2.52781	2.77055	2.1316	2.02356	1.62931
Tmem216	-2.56459	0.00107	0.46867	1.035	1.084	0.91965	1.19112	0.40343
Mzf1	-2.56531	0.00155	0.19588	0.3146	0.39313	0.24599	0.29869	0.23552
Gsn	-2.56871	0.00035	24.9101	105.883	107.984	106.198	55.608	35.1451
Map9	-2.57014	0.00041	0.29515	1.38028	0.95186	0.66501	0.76777	0.66422
Mfsd6l	-2.57195	0.00391	0.28111	0.85785	1.07886	0.9179	0.62872	0.38718
Hes6	-2.57744	8.7E-05	4.22791	7.71642	8.16558	5.43033	6.41463	4.99961
Fam46c	-2.58217	5.5E-07	2.69745	4.56119	5.7411	4.04366	3.83335	3.08144
Pfn2	-2.58724	6.4E-07	0.75229	1.91743	1.63632	1.39661	1.2968	0.95103
Spint2	-2.5881	7.8E-14	13.7413	33.9876	30.0272	26.2307	21.1937	17.1834
Rnf43	-2.58844	4E-06	0.51216	0.95023	0.93195	0.78737	0.74509	0.51351
Ttc9	-2.59015	4.2E-06	2.38218	5.83012	5.20861	3.81083	3.83442	3.38511
Ccdc136	-2.59177	0.0028	0.26723	0.30044	0.34797	0.26849	0.29884	0.20448
Cmtm8	-2.59361	4.6E-05	1.60598	3.03454	3.81974	2.78804	2.25689	2.16853
Sorbs2	-2.59746	0.00041	0.34564	0.72263	0.43699	0.38169	0.41196	0.36479
Hdac11	-2.59848	2.7E-05	0.54163	1.4257	1.05171	0.98253	0.88712	0.7598
Tle2	-2.60119	5.6E-05	1.2846	2.61638	2.7304	1.62497	1.60876	2.13681
Wdyhv1	-2.60169	3E-05	1.83979	5.06552	4.32288	2.86064	3.25084	3.08362
Mrps6	-2.60442	3.3E-05	2.0898	5.74127	3.31663	4.6274	2.7395	1.59904
Tspyl5	-2.61244	0.02677	0.11559	0.55694	0.27725	0.21774	0.33783	0.22111
Gpt	-2.61555	6E-05	0.92708	2.07395	2.31448	1.91275	1.14444	1.46941
Fads6	-2.61691	0.00272	0.2089	0.40542	0.23861	0.27328	0.21237	0.23977
B330016D10Rik	-2.6173	0.01905	0.40853	0.54679	1.30655	0.68408	0.58837	0.67729
Atp2a2	-2.62009	4.6E-05	17.8462	41.8929	23.3642	28.117	19.3721	20.7087
Nsun7	-2.62017	3.9E-05	0.32244	0.5006	0.61872	0.41843	0.46983	0.32896
P4htm	-2.62139	0.00487	0.50615	0.93248	0.61213	0.46739	0.49942	0.70623
Ctnna3	-2.62186	0.01919	0.16026	0.24452	0.16474	0.15095	0.16292	0.15941
Cep89	-2.62566	8.9E-05	1.20669	1.93806	1.54471	1.45824	1.20358	1.28915
Phf24	-2.63009	0.00078	0.14644	0.33973	0.40144	0.2963	0.26466	0.18675
Igf2	-2.63667	0.00014	0.40475	0.61757	0.7628	0.48289	0.42176	0.58843
Nek2	-2.64054	0.02681	1.87219	2.45085	1.8279	1.24002	1.5513	2.08011
Plekhg6	-2.64333	0.01387	0.52661	0.87398	1.03457	0.48499	0.58889	0.85961
Fam167a	-2.64978	8E-05	0.86715	4.39582	4.54992	2.41627	2.50692	2.36377
Odf2l	-2.64981	3.2E-05	0.8907	1.44208	1.5851	1.0825	1.10265	1.02229
Ggt6	-2.64991	0.04434	0.38224	0.5116	0.56757	0.36003	0.42097	0.43872
Epdr1	-2.64992	2.2E-05	0.63103	2.02701	1.53024	1.52402	1.13499	0.81712
Cdkl2	-2.65035	6.8E-09	0.76896	1.65276	1.98394	1.44856	1.31055	0.89988
Dzip1	-2.6504	5.6E-10	1.22069	2.33306	2.05794	1.90094	1.63807	1.15819
Sirt3	-2.65144	0.00104	0.80961	0.93912	1.47957	0.81905	1.09741	0.79156
Ankrd6	-2.65311	0.00028	0.25376	0.45851	0.52171	0.25229	0.39771	0.37215

Nrn1	-2.66009	0.04163	0.26233	0.42133	0.71912	0.41181	0.50004	0.25091
Fbxo31	-2.6608	0.0002	1.16199	2.71459	4.02512	2.12262	1.68603	1.98839
Hpn	-2.66296	0.00058	0.56483	0.81646	0.81289	0.63842	0.57421	0.60507
Nat14	-2.66516	0.00131	0.50114	1.4309	1.18296	0.55943	1.01893	0.93733
Gm26908	-2.66672	0.01547	0.21225	1.0227	1.1152	0.79968	0.57541	0.64963
Naaladl2	-2.66723	2.7E-05	0.20024	0.52709	0.44219	0.35626	0.26577	0.32346
Gm28539	-2.67127	0.00559	0.77679	1.49712	2.07615	1.75597	0.90814	1.6048
Ces1d	-2.67435	0.00027	6.01341	28.9744	37.1234	27.5985	15.7305	11.7837
Pick1	-2.67518	7.2E-07	0.84964	1.36461	1.36885	1.04831	1.03044	0.87822
Hdgfrp2	-2.6766	0.00112	7.51204	11.6519	11.6362	7.10785	7.47988	9.56677
Rpgr	-2.67864	3.2E-05	0.3516	0.94972	0.84334	0.40141	0.66613	0.61143
Epb41l4b	-2.67962	9.3E-09	0.4707	1.65899	1.63083	1.27259	0.99692	0.74034
Macrod1	-2.68182	0.0009	0.81819	1.67912	1.55755	1.71255	0.97041	0.69561
Ift43	-2.6833	9.8E-06	3.88555	10.0809	5.63292	6.7565	4.86163	4.34527
Osbpl10	-2.68339	0.00021	0.63104	1.3393	0.9576	0.91987	0.68735	0.75877
Faim2	-2.68364	0.01061	0.15374	0.43212	0.31609	0.28962	0.23444	0.21567
Usp43	-2.68548	2.5E-06	1.02516	1.86213	1.70623	1.22615	1.25311	1.13927
Ppara	-2.68732	0.02868	0.18116	0.48493	0.33107	0.28438	0.16882	0.36964
Cyb5rl	-2.68744	1.3E-05	0.31176	0.58835	0.67301	0.46983	0.41202	0.40553
Sytl2	-2.6913	1.5E-06	0.88292	0.86161	0.94209	0.89127	0.77829	0.64992
Olfml2a	-2.69375	1.4E-08	0.74735	2.55069	2.58933	1.98143	1.65671	1.06427
Proser3	-2.69618	0.00267	0.59327	1.08519	0.83574	0.68297	0.58637	0.73136
Slc1a4	-2.69943	8E-10	1.29802	3.38773	3.05281	2.46373	2.08434	1.67038
Bbs9	-2.70019	3.2E-07	1.28008	2.77104	2.80977	1.91048	1.56538	1.85957
Ccdc157	-2.7009	2.9E-05	2.05155	5.34188	4.89957	2.75863	2.79136	3.53594
Adam22	-2.70239	2.9E-08	0.27461	0.86689	0.99938	0.65407	0.62091	0.42749
Acox2	-2.70454	0.0003	0.6881	1.13027	1.09317	0.88379	0.938	0.57437
Prr29	-2.70612	0.01483	0.80617	2.26587	0.96684	1.13898	1.02444	1.02808
Tmem102	-2.70846	0.00317	0.23967	1.58783	0.90337	0.75247	0.79185	0.64185
Dalrd3	-2.71039	1.8E-05	4.19362	10.595	7.49167	5.38494	5.86797	5.84074
Ahsa2	-2.71053	3.8E-05	0.92001	4.69969	2.6446	3.36993	2.18234	1.50569
Tmem38a	-2.71565	0.00138	0.64885	1.47633	1.22283	1.12043	1.15434	0.4551
Prdm16	-2.71808	4.6E-11	0.83252	1.70631	1.61794	1.22498	0.96635	1.01732
Sec14l2	-2.72567	7.6E-06	0.39653	1.72484	1.13228	1.01671	1.14215	0.42983
Coq8b	-2.72648	0.00432	3.23754	6.46882	5.59969	3.92068	3.85945	4.80704
Abhd3	-2.73089	0.00104	0.35419	1.18513	0.8091	0.85255	0.75014	0.27101
1810064F22Rik	-2.73098	0.01993	0.25242	0.57434	0.57663	0.34342	0.36354	0.32191
Gramd1c	-2.73385	0.00017	0.41545	0.61166	0.68016	0.43479	0.51621	0.42385
Tacc2	-2.73686	6.6E-09	1.23985	3.46765	2.53736	2.26045	1.91239	1.75344
Zc2hc1c	-2.7375	1.1E-06	0.4393	0.83006	0.7615	0.64905	0.68302	0.29659
Gm10643	-2.73754	0.04179	0.22033	0.64876	0.60398	0.2767	0.5973	0.28098
Cep131	-2.7433	0.00084	1.14822	2.20814	2.19503	1.23329	1.30552	1.66467
Gm20219	-2.74678	0.01889	0.63888	1.31927	1.56369	0.80234	0.92784	1.04752
Elf5	-2.74805	0.00164	0.09854	0.39565	0.62126	0.38362	0.27047	0.24127
Slc8a2	-2.75	0.00923	0.13209	0.3359	0.22631	0.20735	0.17904	0.16845
Cyp39a1	-2.75045	4E-05	0.49317	1.85834	2.10587	1.42927	1.2534	0.81277
Faah	-2.75118	1.5E-06	0.68007	1.81648	2.00611	1.03046	1.20238	1.14254
Scube3	-2.75179	6.4E-07	0.46809	1.29954	0.93488	0.85659	0.59143	0.65493
Arhgef28	-2.75221	9.9E-08	2.25754	4.24996	3.99464	2.9281	2.52077	2.70435
Mrvi1	-2.75541	1.4E-05	0.64467	1.56672	1.51142	1.00136	1.25038	0.68799
Mum1l1	-2.75666	0.00063	0.09534	0.47854	0.52273	0.37418	0.32713	0.18238
Cds1	-2.75898	8.7E-09	2.32285	4.22659	4.61447	2.80639	3.24323	2.67729
Dnal1	-2.76033	1.5E-11	1.13515	2.8286	2.36815	1.69813	1.75647	1.46892
Dgkg	-2.76465	0.03853	0.18681	0.28336	0.14225	0.0782	0.18991	0.20646
Mgst1	-2.76808	1.2E-08	6.7892	20.2836	23.7764	14.5236	13.1898	10.3457
Tcea3	-2.76981	1.2E-05	2.73002	4.65043	4.70568	3.06489	2.94357	3.41822

Xylb	-2.77046	4.2E-05	0.36669	0.92873	0.65725	0.7262	0.54484	0.30216
Zfp239	-2.77851	0.03875	0.07962	0.19181	0.29101	0.19998	0.12141	0.14215
Ift172	-2.78353	1.4E-08	2.32794	5.11639	5.16998	3.52805	2.98939	3.02679
Ascc2	-2.78556	7.3E-09	4.45638	10.5862	10.3453	6.78329	6.28562	5.94493
Sned1	-2.78783	4.6E-08	2.20875	5.72301	5.07632	3.47884	3.31853	3.51083
Sord	-2.79378	4.2E-08	3.34851	8.10662	10.4616	5.13289	5.35649	5.23735
Ccdc171	-2.79412	4.8E-06	0.65642	1.62792	1.7068	1.03046	0.95191	1.01931
Ptpn13	-2.79685	1.3E-11	1.92362	5.472	4.56483	3.28115	2.65607	2.71676
Nr1d1	-2.80035	0.00302	2.93594	7.88926	7.5581	8.41421	3.27169	2.13127
Tpm2	-2.80184	1E-06	6.01193	13.8762	10.7777	10.1752	7.30579	6.3061
Esrp1	-2.80257	2.9E-06	1.39608	2.10947	2.12752	1.46491	1.70872	1.32123
Mettl7a1	-2.80769	3.3E-07	12.1785	41.5896	47.7165	31.4387	23.0108	19.9079
Palmd	-2.81665	1.1E-07	1.18555	2.48999	2.16661	1.81338	1.3134	1.34908
Pgbd1	-2.81698	0.02879	0.06461	0.23349	0.30996	0.20286	0.18064	0.09888
Pitpnm3	-2.81864	5.6E-06	1.27442	2.49223	2.40181	1.15591	1.43954	1.85096
Zswim5	-2.81939	0.0058	0.10374	0.24993	0.27017	0.16937	0.16875	0.15876
Mecom	-2.82127	1.3E-11	1.35717	3.05868	2.68527	1.84874	2.01949	1.64143
Ddrgk1	-2.82287	5.9E-07	5.44639	9.68468	9.40745	8.27069	5.62159	5.31553
Aspa	-2.82313	0.0459	0.09726	0.32805	0.10665	0.23208	0.13843	0.04465
Hnf1b	-2.82516	0.00031	0.54324	1.48325	0.79423	0.88691	0.58908	0.69278
Plxnb1	-2.83081	9.2E-06	4.76945	12.0624	13.6371	5.59549	6.59172	8.58304
Hsp90aa1	-2.83307	1.8E-06	14.9378	56.974	44.0176	47.9538	25.6242	16.5699
Rbpms2	-2.83596	0.00043	0.50838	0.92786	0.9502	0.78356	0.72826	0.45972
Dmkn	-2.84497	0.00076	0.73972	1.69724	1.33978	1.26076	1.20858	0.48515
Hook2	-2.85041	7.9E-05	1.83424	2.78231	2.55398	1.57226	1.76111	2.07182
Gm9899	-2.85112	0.01844	0.07204	0.30855	0.32914	0.18095	0.17089	0.147
Fam71f2	-2.85167	0.02441	0.2723	0.65602	0.74645	0.56995	0.50751	0.20836
Gm26583	-2.85785	0.03973	0.06288	0.32821	0.51709	0.39482	0.2397	0.07217
Dapk2	-2.86071	1.2E-06	0.52765	3.01907	3.07367	1.90513	1.77687	1.16075
Dmd	-2.87484	2.4E-08	0.52068	1.42243	1.18387	0.83418	0.64558	0.75957
Fam187b	-2.87747	0.00248	0.17045	0.61595	0.67166	0.34784	0.47652	0.29345
Gnao1	-2.87816	4.6E-07	1.4621	2.18986	2.95034	2.22966	1.23865	1.71363
Rasd1	-2.87846	0.04084	0.86271	1.0392	1.7737	1.53488	0.58469	0.77014
Dnajb1	-2.87873	0.00049	2.2412	9.05702	3.79028	6.94576	2.81127	1.88084
Entpd5	-2.8799	5.7E-14	1.41921	3.55771	3.61443	2.36038	2.39814	1.67292
Hid1	-2.8808	4.1E-08	1.68877	3.36005	2.59107	1.85179	2.08836	1.79363
Arhgap8	-2.88709	2.4E-06	0.65324	1.73113	1.14157	0.98445	0.89652	0.77474
Abcb9	-2.88909	3.9E-05	0.26882	0.66423	0.36845	0.45446	0.45197	0.23733
B230369F24Rik	-2.8896	0.02666	0.13573	0.29065	0.55809	0.25568	0.29896	0.2077
Grm4	-2.89022	0.01207	0.08711	0.47221	0.29851	0.20513	0.15498	0.28329
Ccdc85c	-2.89382	6.8E-05	1.123	2.58524	2.46277	1.64539	1.33193	1.66128
Sec14l3	-2.89628	1.9E-09	32.7406	125.682	125.845	72.5956	65.8285	56.8203
Bag2	-2.89985	2E-05	0.71969	1.11462	0.98644	0.87155	0.8623	0.39334
Rbp4	-2.90038	0.0015	0.62508	2.86842	2.69268	2.07469	1.13476	1.36655
Pllp	-2.90053	0.00019	0.77843	1.78605	1.14315	1.36165	1.15877	0.7658
Rec8	-2.9031	0.04175	1.47514	2.40306	1.67527	0.76749	1.47821	1.74145
B630019K06Rik	-2.90373	0.01699	0.44839	0.4681	0.55312	0.2534	0.41025	0.44602
Otud3	-2.90648	1.8E-06	2.15251	5.40878	4.23513	3.40087	2.68239	2.65649
Myh14	-2.9074	5E-10	14.2525	37.1625	33.3404	24.0088	16.6342	19.2399
Rims2	-2.90848	0.01744	0.18362	0.18432	0.25168	0.10089	0.175	0.19319
Ccl27a	-2.90928	0.02003	0.29235	0.86083	0.73463	0.85668	0.74301	0.41011
Plp1	-2.91118	0.00218	0.25037	0.25133	0.34317	0.19652	0.22272	0.20754
Aldh1a7	-2.91843	0.00033	3.08328	7.51812	9.60467	7.3219	4.2743	3.38869
Ngef	-2.92183	8.5E-08	1.03807	2.61996	2.54075	1.39679	1.44729	1.49781
Ptprn2	-2.92206	0.01628	0.25582	0.44331	0.15686	0.15218	0.22585	0.23695
Lgr4	-2.92845	8.7E-06	0.51178	0.75874	0.45865	0.63036	0.47025	0.24098

Fbxl2	-2.93059	1.5E-05	0.44305	0.98366	0.89304	0.49095	0.7551	0.59825
Nr4a1	-2.93325	0.00235	3.83879	11.9926	4.58785	6.46717	4.33087	4.08357
Cldn7	-2.936	6.3E-08	2.67549	6.54116	7.04904	5.15206	3.68701	3.13902
Itih2	-2.93711	0.00081	0.20963	0.72948	0.40704	0.43877	0.28415	0.29407
Mtus2	-2.93858	1.8E-05	0.18709	0.87328	0.55293	0.39649	0.39822	0.34894
Dqx1	-2.94379	0.00131	0.74525	1.06093	1.02147	0.80831	0.61988	0.69984
Dnajb2	-2.95726	4.6E-11	2.80757	6.87331	5.36127	4.86097	3.08974	3.04509
D2Bwg1423e	-2.95986	0.00377	0.36613	0.68605	0.71093	0.42149	0.58933	0.28015
Isg20	-2.96129	6.5E-08	2.17939	3.70034	2.77379	3.08891	2.4055	1.14349
3632451O06Rik	-2.96788	0.01217	0.12647	0.55861	0.47671	0.49636	0.27322	0.07258
Ttc30b	-2.97607	1E-06	0.80401	2.10688	1.74	1.16915	1.61321	0.71233
Chchd10	-2.97858	6.3E-06	3.33026	11.5889	10.228	9.13918	5.8934	3.39759
Ift88	-2.98009	2E-08	0.85088	2.91406	2.8127	1.69715	1.71729	1.24467
Acsl3	-2.98349	5.4E-10	1.16962	3.05261	2.33182	2.0364	1.72952	1.01699
Zmynd12	-2.9879	0.01917	0.37181	0.59717	0.38221	0.54477	0.22049	0.23708
Oscp1	-2.99062	1.4E-06	0.24248	0.90282	0.90643	0.67826	0.4482	0.37108
Pisd-ps1	-2.99067	0.00248	13.777	35.3613	24.6657	11.1759	14.5754	21.9912
Klf5	-2.9959	6.3E-07	2.96109	7.67416	5.44219	5.11323	3.31814	3.19261
Rarb	-2.99699	8.3E-07	0.61078	1.90064	1.7528	0.86293	1.22244	0.9639
Usp2	-3.00108	8.9E-05	1.08175	3.32036	2.50409	1.90193	1.09971	1.04844
Fkbp4	-3.00299	1.2E-08	5.42838	17.7698	16.1018	14.8835	7.56165	5.80235
Rsph3a	-3.00758	0.001	1.48147	2.93175	2.06682	2.15953	2.01706	0.76921
Rasef	-3.00961	6.4E-09	0.61504	1.53283	1.80228	0.98549	0.90555	0.92499
Klrg2	-3.0097	6.9E-06	0.60922	1.22308	1.15975	0.82885	0.53332	0.82871
Tmem158	-3.01092	0.00359	1.62363	1.14088	0.78817	0.93457	0.99724	0.82823
Sntb1	-3.01346	1.1E-11	1.33218	3.53361	3.40288	2.07862	2.33926	1.54445
Ift57	-3.02041	7.8E-08	2.10433	3.44476	2.93974	2.05829	2.13928	2.10559
Gspt2	-3.02409	0.00091	0.18379	0.39358	0.44084	0.30775	0.31141	0.11719
Spata24	-3.0254	0.00306	2.5152	3.36639	3.36535	1.80499	2.4961	2.38277
Neurl1a	-3.02966	7.8E-08	0.49888	1.39165	1.33158	0.80789	0.72071	0.78354
Aldh3b1	-3.03579	2.5E-16	5.49755	13.8893	12.1494	8.80472	8.82285	5.2936
Lmntd1	-3.03639	0.03717	0.11957	0.15603	0.1434	0.12201	0.08357	0.13724
Tmem231	-3.03976	6.8E-07	0.6048	1.94274	1.5543	1.13931	0.92227	0.86769
Dsg2	-3.04167	2.6E-06	0.44826	1.03493	1.152	0.69489	0.75476	0.63239
Chrm2	-3.04223	7.6E-05	0.82277	2.18193	1.58667	0.82903	1.69232	0.64419
Prodh	-3.04253	1.5E-08	0.90353	1.69302	1.74295	1.09268	1.17368	0.87059
Ppp2r2c	-3.04378	0.0127	0.13013	0.18811	0.17837	0.17977	0.1323	0.03983
Ccdc173	-3.04783	0.00389	0.49805	1.78164	1.24117	0.62548	0.73647	0.97005
Irx3os	-3.05809	0.00532	0.34202	0.64919	0.44747	0.23429	0.30028	0.47582
Gsg1l	-3.05875	0.04552	0.05905	0.21338	0.24279	0.16685	0.10505	0.11295
Elof1	-3.07355	1.4E-08	4.89952	12.2409	9.74674	7.56319	5.67268	5.46719
Gm15728	-3.07666	0.03792	0.1301	0.28731	0.17832	0.06127	0.18185	0.1742
Tctn2	-3.07743	7.6E-07	0.50126	1.35297	1.23096	0.73443	0.63698	0.72448
Sult5a1	-3.08094	0.00023	0.41935	0.442	0.80828	0.64185	0.45296	0.42114
Aoc3	-3.08191	0.01191	0.22557	0.96611	0.79012	0.48788	0.45864	0.36438
Iqck	-3.08309	4E-06	0.21638	0.47786	0.46959	0.28307	0.26581	0.27595
Espn	-3.08396	1.8E-07	0.43404	0.5397	0.47017	0.30771	0.44125	0.28926
Pxylp1	-3.09217	1.2E-07	0.49787	1.4309	1.02359	0.72397	0.74588	0.6015
Cfap52	-3.09854	4.7E-06	1.09829	3.568	2.01854	1.41064	1.97929	1.37515
Dync2h1	-3.09855	9.5E-12	1.33191	3.60701	3.45205	1.83728	1.905	1.79973
Slc23a2	-3.10179	1.4E-13	5.84121	14.9029	16.202	9.47609	7.64861	7.46771
Zfp474	-3.10663	8.6E-06	1.01979	2.22285	2.03008	1.12825	1.3823	1.15188
Tnk1	-3.10674	2.8E-06	0.7654	1.62046	1.45442	0.8957	0.91959	0.74538
Ccdc138	-3.1096	0.00361	0.57171	1.07126	0.88156	0.50858	0.50856	0.72909
D830026I12Rik	-3.11229	0.00026	0.54839	0.83673	1.20262	0.41322	0.80837	0.54549
Hmgcs2	-3.11787	4.6E-05	0.38724	1.63967	2.02654	1.19368	0.80523	0.75422

Rassf9	-3.12508	3.1E-12	3.11695	6.45527	7.04538	3.70842	3.9192	3.76575
C530008M17Rik	-3.12946	0.00234	0.05013	0.19323	0.12368	0.10702	0.06625	0.09973
Ccdc74a	-3.13114	1.6E-05	0.65778	1.13772	0.8669	0.60367	0.74586	0.50331
Barx2	-3.133	0.00033	0.70621	1.27603	1.00829	0.92386	0.56837	0.63043
Ccdc181	-3.13512	2.3E-07	2.66948	9.54934	6.11201	4.11442	3.82402	4.03876
Klf15	-3.15097	0.0028	0.58096	5.89843	5.7731	4.61216	1.47653	1.71462
Chrm3	-3.1516	0.01276	0.20558	0.41273	0.35222	0.14523	0.16981	0.23595
Etfbkmt	-3.15325	2.1E-07	1.29925	3.65178	3.62521	1.51513	2.12276	2.02379
Gm17018	-3.15495	1.9E-05	2.14803	5.68775	5.17217	2.98925	3.36408	2.39875
Snhg11	-3.16528	2.1E-12	0.84272	1.15701	1.15506	0.85349	0.75999	0.62402
Col20a1	-3.16613	0.00135	0.35909	0.79855	0.64362	0.32955	0.39313	0.48611
Gipc2	-3.1665	1.2E-08	0.96541	2.08355	1.92281	1.4208	1.1348	0.71561
Arhgef19	-3.16833	3.4E-05	0.87369	1.49094	1.55303	0.53147	0.99923	1.06545
Crocc	-3.16899	0.0036	1.56929	2.64421	2.09331	0.96781	1.26773	1.83331
Tnxb	-3.16969	3.3E-10	3.18742	8.71504	8.32802	5.40875	3.29822	4.31162
Fhl2	-3.17253	0.01357	1.16002	2.64758	0.96235	0.88176	1.27239	1.07447
Rd3	-3.17392	0.00487	0.10847	0.45731	0.37168	0.25542	0.20676	0.20749
Pdzd7	-3.17576	0.00109	0.16995	0.50041	0.50469	0.28457	0.23036	0.30342
Ehf	-3.18372	1.9E-12	0.99701	1.8515	1.89322	1.3434	1.04524	0.90591
Sec14l4	-3.19187	1.6E-11	1.49466	4.96117	5.1671	3.35788	2.34675	2.03316
Fam131a	-3.19387	1.1E-05	1.42085	3.09703	2.72645	1.24907	1.3619	2.05011
1110004E09Rik	-3.19461	7.9E-09	3.49161	8.61698	6.88677	4.65234	3.72275	4.3007
Nectin4	-3.19603	7.9E-11	1.08213	2.35873	2.66978	1.41725	1.47728	1.25384
Tbc1d30	-3.2017	3E-14	0.91581	2.07557	2.07812	1.17568	1.35515	0.91875
Cfap53	-3.20799	3.1E-06	1.15819	3.2553	2.77805	1.76221	1.40385	1.5679
Ttc41	-3.20936	0.0036	0.19943	0.92088	0.80296	0.34438	0.45617	0.41964
9230110C19Rik	-3.2098	0.00166	0.4365	1.22687	1.14672	0.50251	0.59167	0.83499
Rassf7	-3.21681	8.9E-09	1.3447	3.14567	3.68617	2.01915	1.72364	1.78941
Ppp1r36	-3.21835	0.00053	0.96852	1.87962	1.88061	1.01361	0.82051	1.35864
Hspa2	-3.22062	3.4E-12	2.11416	4.90879	4.28362	3.52087	2.28944	1.93418
Aldh5a1	-3.23348	0.00017	0.24612	1.10352	0.75901	0.50227	0.55254	0.40803
Lhfpl4	-3.23509	0.00191	0.0797	0.512	0.35501	0.2252	0.15191	0.24392
Lca5	-3.24029	8.4E-09	0.69793	1.9174	1.5356	0.92263	0.84955	0.91347
Rpgrip1l	-3.24473	1.3E-10	0.84992	1.83545	1.6923	0.98805	1.08589	0.80852
Bbs1	-3.24598	4.7E-11	0.72633	1.44987	1.39376	0.71671	0.82279	0.84159
Ppargc1a	-3.24711	4.9E-05	0.36896	0.67725	0.42143	0.3751	0.26792	0.34953
2010204K13Rik	-3.24795	0.02313	0.21949	0.52879	0.70197	0.36754	0.26033	0.39188
Calml4	-3.25493	1.9E-05	1.0545	2.61102	1.98734	1.76574	1.07201	1.00858
Aqp4	-3.25722	3.2E-07	0.61533	1.22621	0.78092	0.72984	0.6603	0.40108
Foxq1	-3.25977	1.2E-06	1.04774	2.76957	2.36354	1.61736	1.1761	1.16914
Kcnmb2	-3.26993	2.8E-05	0.44432	1.07043	1.218	0.58125	0.52698	0.70828
Adam33	-3.27172	0.00109	0.8374	1.94846	1.70695	0.45842	0.89497	1.31436
A730020E08Rik	-3.27706	0.02713	0.05947	0.14327	0.36681	0.11203	0.18138	0.09101
Mall	-3.28035	2.9E-05	1.03925	1.9937	1.38487	0.83385	0.93912	1.14862
Lrrc73	-3.28388	0.01186	0.58579	1.14245	0.4588	0.63057	0.38283	0.44823
Misp	-3.28411	5.8E-06	0.79169	1.27153	1.0532	0.81879	0.85218	0.4276
Rhpn2	-3.28789	8.1E-10	1.10389	2.25214	1.55393	1.18026	1.21322	0.89031
Ccdc191	-3.28973	8.8E-07	1.42378	2.70451	2.58947	1.35824	1.28042	1.48748
Ccdc88c	-3.28981	7.8E-07	5.9258	10.2688	8.92679	4.58499	5.01707	5.71338
Ace2	-3.2947	0.00187	0.12315	0.9642	1.39251	0.44463	0.39122	0.65959
Tmem132d	-3.30524	0.00142	0.05361	0.24397	0.28168	0.19076	0.109	0.09572
Mettl7a3	-3.30751	0.03378	0.21475	0.45987	0.49056	0.17979	0.47301	0.05477
Irx2	-3.31089	8.8E-15	2.01148	4.30753	3.74857	2.72557	2.02695	1.91714
Fam198a	-3.31099	4.1E-05	0.26928	1.20135	0.71766	0.48847	0.45625	0.48076
Wnk4	-3.31442	1.1E-05	0.55843	1.21082	1.53082	0.87664	0.53932	0.74776
Akap6	-3.31635	0.00028	0.49655	1.0875	0.63418	0.34014	0.55642	0.50946

Grhl2	-3.31792	3.8E-10	1.28314	2.63519	2.7534	1.53219	1.3793	1.40029
Gstm1	-3.31914	3E-07	11.637	26.8986	27.0504	21.2797	14.508	7.64074
Rab36	-3.31963	3.5E-05	0.73149	1.92795	1.79955	0.69487	1.04872	0.99025
Ccdc67	-3.32356	0.00038	0.41976	0.67418	0.86301	0.28555	0.48007	0.50854
Celsr1	-3.32922	2.4E-10	3.24923	5.80778	5.28674	3.26445	3.10073	2.94333
Gsta3	-3.33507	1.1E-09	4.31242	10.3893	12.7597	8.72543	5.1495	4.76624
Aox1	-3.35633	7.5E-09	1.57269	4.52735	4.79526	1.66542	2.31014	2.41692
Wfdc2	-3.35721	6.2E-11	38.708	91.5401	93.6462	62.0725	51.3833	32.1833
Gas8	-3.36165	5.8E-05	2.85795	5.70292	5.18338	2.32028	2.11303	3.09244
Entpd2	-3.37097	0.00705	0.32656	0.91785	0.96978	0.78604	0.5533	0.37481
Rap1gap	-3.37136	1E-08	1.42319	2.87894	3.50629	2.39819	1.3461	1.30953
Stum	-3.3751	0.00023	0.12563	0.34051	0.19372	0.19722	0.1357	0.09613
Appl2	-3.38219	1.9E-07	6.4636	9.55258	7.77852	4.86553	5.28447	5.33302
Slc5a1	-3.38983	0.00014	0.42332	0.4306	0.52219	0.40758	0.27255	0.23753
Grhl1	-3.39141	2.1E-09	0.59564	1.64758	1.47407	1.10128	0.7401	0.58236
Smim5	-3.39441	0.00265	1.47496	2.70735	2.50297	1.72003	1.46375	1.45103
Tsga10	-3.39695	7.9E-09	0.554	1.46553	1.39956	0.68211	0.70677	0.73144
Sox6	-3.39718	8.9E-07	0.12507	0.38024	0.34286	0.14025	0.19527	0.19824
Pir	-3.39763	2.1E-09	0.52143	2.12716	1.92965	1.11322	0.92235	0.79795
Fsd1l	-3.40045	3.6E-11	0.66597	1.68082	1.6952	1.03052	0.73748	0.76436
Myh7	-3.4031	1.6E-05	0.26979	0.68245	0.62862	0.27952	0.29484	0.41286
Ttc26	-3.4067	1.8E-10	0.54526	0.99517	0.98516	0.62252	0.56692	0.45515
Gm26671	-3.40953	0.00265	0.35991	0.98269	1.23327	0.3616	0.43907	0.88125
Ccdc92	-3.40953	1.1E-07	0.80636	2.01081	1.39608	1.22587	0.69032	0.81184
Nap1l3	-3.41135	0.00736	0.24495	0.49176	0.47562	0.20508	0.37352	0.18742
Flt3l_1	-3.41296	0.01123	2.03145	2.62712	2.20581	1.04368	1.15329	1.51402
Pxmp2	-3.41524	0.00948	0.48258	1.31762	0.79373	0.24242	0.83401	0.2954
Alpk3	-3.42857	0.03383	0.21564	0.34635	0.13547	0.13541	0.08221	0.22
Cracr2b	-3.43231	2.6E-06	5.57307	12.8543	9.39036	4.55197	4.74975	6.28424
Plekhd1	-3.43916	7.1E-06	1.05852	2.33761	2.57927	0.97855	1.01631	1.52988
Morn4	-3.44202	0.00073	0.31889	0.97311	0.69932	0.48057	0.48627	0.2928
Fam149a	-3.46045	2.6E-18	1.59509	4.06151	2.98615	2.03985	1.96765	1.414
Gp5	-3.4639	0.02564	0.31645	0.50824	0.54217	0.29806	0.13404	0.44391
RP23-478P10.3	-3.46902	0.04873	0.82336	1.5428	2.06896	1.20636	0.83703	0.63
Ankrd66	-3.473	4.5E-05	0.96443	2.53468	2.52359	1.32129	1.15871	1.27463
Capsl	-3.483	8.8E-07	2.57104	4.81757	4.11128	2.38321	2.11588	2.7167
mt-Nd5	-3.48366	0.01211	1145.81	990.511	1067.01	324.198	356.585	1180.83
Homer2	-3.48762	1.3E-05	0.10094	0.39795	0.44652	0.17287	0.16792	0.22469
Pisd-ps2	-3.48883	0.00476	4.28553	8.29489	7.56829	1.75951	4.24499	5.59134
Synpo2	-3.49711	3E-10	1.39286	3.43349	2.3743	1.36175	1.49665	1.43676
Enah	-3.50362	7.8E-14	1.84028	4.54875	4.61081	2.40654	2.14975	1.98944
Col8a2	-3.50444	1.1E-07	1.05783	3.39795	4.08446	1.6201	1.75735	1.57934
Lmod1	-3.5092	3.7E-08	0.93876	3.43953	2.69402	1.86052	1.10346	1.25703
Adamts20	-3.51047	0.0349	0.09011	0.22743	0.22055	0.04042	0.14396	0.12805
Wdr34	-3.51338	2.5E-06	0.88374	4.13985	3.29739	1.63395	1.52232	1.65295
Ttll8	-3.51633	0.00272	0.16893	0.40698	0.50939	0.19094	0.18891	0.31022
Sox2ot	-3.51878	0.02772	0.05129	0.09267	0.03515	0.04831	0.03259	0.01962
Nme5	-3.52348	1.2E-07	1.70618	5.07759	3.50782	2.52084	1.83654	1.99665
mt-Nd1	-3.52544	0.009	1305.96	1031.45	1187.69	381.186	418.128	1266.52
Alas1	-3.52699	0	9.65348	30.1033	31.471	18.3409	12.8405	10.7518
Lekr1	-3.52737	3.2E-05	0.30139	0.67423	0.41309	0.28388	0.25168	0.32944
Lrrc56	-3.53254	1.2E-05	0.74439	1.67072	1.08569	0.41948	0.75675	0.86167
Pglyrp1	-3.53303	0.0019	0.93907	2.51375	1.35864	1.44142	1.00771	0.55887
Lrrc45	-3.53826	1.1E-05	6.40118	12.71	10.6779	4.5937	5.04152	7.32001
Mlph	-3.54094	7.9E-05	2.70252	2.93419	2.60774	1.61553	2.11	1.68738
Syt10	-3.54132	0.0003	0.31387	1.20987	0.77437	0.31534	0.60626	0.48033

Gria1	-3.54154	0.00643	0.03833	0.49252	0.66987	0.37308	0.13639	0.19064
Sptlc3	-3.5417	0.0025	0.36639	0.65384	0.47428	0.30675	0.31039	0.1246
Dusp18	-3.54723	4.2E-10	1.00017	3.87536	3.4957	1.69569	1.77936	1.39665
Adcy5	-3.54864	8.7E-13	1.22315	3.61044	2.95652	1.76444	1.47031	1.37857
Xk	-3.55048	2.1E-05	0.25099	0.42143	0.32838	0.28655	0.20876	0.13967
Ccdc160	-3.55374	0.00045	0.45242	0.78718	0.87848	0.42613	0.57492	0.28848
Osbpl6	-3.55383	2.7E-15	1.26604	4.5751	4.35396	2.23317	2.06515	1.6116
Lrig1	-3.56094	1.9E-09	2.52998	3.54715	3.14081	1.75793	2.05549	1.91998
Rab11fip4	-3.56297	1.1E-09	1.08809	2.18447	2.0027	0.88821	1.3906	0.91582
Morn2	-3.56599	0.00145	0.71937	3.17731	1.60225	1.12929	1.27981	0.82566
Ptgfr	-3.56985	0.00391	0.06835	0.52144	0.3396	0.27897	0.11291	0.13075
Cux2	-3.57296	1.1E-06	0.1838	0.51661	0.53535	0.20919	0.25109	0.27249
Mycl	-3.58242	1.3E-07	0.60233	1.8657	2.61431	1.42283	0.8456	0.76813
D630039A03Rik	-3.58442	3.1E-06	0.57296	1.18315	1.4304	0.61676	0.66568	0.65761
Kif9	-3.58881	7.9E-12	1.02399	2.20728	1.86521	1.01526	1.0547	1.01033
Pacrg	-3.59286	2E-05	0.73676	2.72163	1.61573	1.0178	0.93625	1.07111
Map7	-3.59606	9.4E-07	1.89403	4.17921	3.29962	1.86734	1.55659	1.85557
Tmem125	-3.59636	8.1E-06	0.5942	1.63034	1.49312	0.65295	0.62924	0.94721
Lca5l	-3.5977	0.00015	0.15985	0.85578	0.52339	0.27882	0.31598	0.28539
Cd24a	-3.59779	0	26.8	54.1108	37.5945	23.4246	23.2975	20.6438
Ggnbp1	-3.60061	0.00018	0.23354	0.58943	0.66306	0.19902	0.50027	0.54885
Anxa8	-3.60561	1.6E-08	3.38833	9.39981	5.06636	4.6421	3.78906	2.45121
Ptprz1	-3.60735	7.7E-10	0.5047	1.17369	0.70618	0.44237	0.52912	0.45054
Myom1	-3.61433	3.8E-05	2.02153	5.42769	2.79876	1.87201	1.24551	2.49987
Ldhb	-3.619	2.3E-09	5.01637	17.8316	20.3235	9.44978	8.66196	6.60421
mt-Atp8	-3.61904	0.00387	1208.72	832.063	1007.34	351.867	390.455	1055.19
Gal3st1	-3.62335	7E-06	0.45397	1.27596	1.82779	0.64139	1.00955	0.47762
Irx5	-3.63215	1.9E-10	0.85188	2.52822	2.46216	1.18613	1.07313	1.1903
0610040J01Rik	-3.63281	3.3E-09	0.96395	1.97397	1.84972	1.39218	1.10246	0.44255
Ldb3	-3.6333	0.00508	0.38504	1.46872	0.59371	0.44729	0.28379	0.58923
Elf3	-3.63852	4.8E-06	7.83621	9.39735	6.94558	3.51002	1.91192	3.79745
Ccser1	-3.64199	1.8E-08	0.80925	1.70711	1.29957	0.59916	0.75515	0.81329
2610528J11Rik	-3.64797	0.00288	0.28907	0.61903	1.32068	0.72605	0.48978	0.14746
Ankmy1	-3.65226	0.02796	0.3107	0.38386	0.57327	0.16508	0.14578	0.42061
6430531B16Rik	-3.65716	0.02354	0.52381	1.91626	1.19658	0.32891	0.91709	0.44533
Mmp15	-3.66037	9E-16	4.84994	11.5291	10.839	5.76743	4.9523	4.844
Tyro3	-3.66312	2.1E-09	0.74746	1.89079	1.99777	0.95044	0.80737	0.90079
Synm	-3.67258	1.4E-07	1.54757	4.86557	4.0622	3.19948	2.42669	2.18954
Col26a1	-3.67364	0.03179	0.03964	0.14324	0.12224	0.04978	0.07052	0.06066
Acsl1	-3.67795	8.8E-14	1.27314	3.6472	4.29542	2.18309	1.81076	1.29264
Asb14	-3.68189	0.00097	1.5903	2.15281	2.55339	0.74181	1.03931	1.73834
Gm12216	-3.68558	0.00114	1.96897	1.44063	1.25941	0.8792	0.97859	1.13831
Gdpd2	-3.69344	0.00087	0.25129	0.98881	1.65324	0.63117	0.28101	0.82679
Clmn	-3.69625	9.3E-12	0.84236	1.72613	1.40671	0.83293	0.77268	0.71863
Fam216b	-3.70352	0.00458	1.74502	4.28408	4.33937	1.56535	1.5966	1.56412
Cdc14a	-3.7036	2.2E-09	1.73703	4.00425	2.68194	1.4757	1.55813	1.69657
Ctxn1	-3.72302	3.1E-07	3.63129	9.59247	6.54892	3.36029	3.49731	4.1678
Wdr60	-3.7313	8.6E-06	2.33193	5.79275	5.13527	1.93286	2.13359	2.97384
1700001C02Rik	-3.73327	0.00079	0.22316	2.15049	1.37641	1.12102	0.51045	0.59763
Por	-3.73879	0	9.27475	25.9758	24.8916	18.3398	9.20917	7.42729
Ttc12	-3.74069	4.6E-11	1.55445	2.59892	2.44493	1.10409	1.48959	1.2479
Wnt11	-3.74428	2E-08	1.20383	2.60793	2.39826	1.03719	1.23805	1.22102
Cetn4	-3.7472	1.8E-05	0.37342	1.5686	1.06303	0.54112	0.52564	0.54948
Cfap69	-3.7544	1.4E-11	1.27467	2.98851	2.74115	1.2052	1.28839	1.37892
Klhdc9	-3.75586	0.04676	0.37169	0.47758	0.61135	0.14004	0.18893	0.51193
Tjp3	-3.75604	7.2E-06	2.7576	3.53703	3.59593	1.70753	1.67425	2.31515

Myom3	-3.75868	0.00123	0.13427	0.12323	0.22347	0.06022	0.10725	0.11742
Cdhr4	-3.75993	7.2E-07	6.94765	12.8475	8.51895	4.64561	4.90594	5.40229
2310010J17Rik	-3.76447	0.03777	0.57621	0.46273	0.92141	0.24121	0.78105	0
Gm37240	-3.7743	0.00219	0.68215	1.11516	1.15203	0.7649	0.92877	1.30489
D330023K18Rik	-3.7769	0.02263	0.27819	0.2234	0.63549	0.29114	0.28281	0.07095
Sgsm1	-3.78184	0.00047	0.58949	0.58551	0.47841	0.14611	0.36272	0.45106
Pcsk1	-3.79245	0.00381	0.10721	0.34437	0.29388	0.13464	0.18165	0.10937
Klkb1_1	-3.79425	0.00137	0.11033	0.35439	0.31755	0.15241	0.2131	0.08442
Lrrc34	-3.79477	0.00015	0.55123	2.16414	1.17527	0.61535	0.84057	0.74983
Tekt2	-3.79529	0.00056	0.79245	2.10493	2.25586	0.95692	0.65068	0.93285
Foxc1	-3.79612	7E-05	0.38089	1.34113	0.40158	0.53353	0.31275	0.3587
1700088E04Rik	-3.79762	6.8E-07	0.72223	1.12482	0.85492	0.72836	0.68972	0.50238
Aim1l	-3.80201	6.4E-05	0.40041	0.93786	1.21198	0.35619	0.37314	0.61276
Dyrk3	-3.80347	1.5E-06	0.29155	1.08286	1.04898	0.41192	0.64836	0.30674
Sntn	-3.80547	2.1E-06	1.21423	3.67534	2.04833	1.2903	1.28188	1.3579
Muc4	-3.81075	9.4E-08	6.45246	9.00262	7.46674	5.29503	4.32134	4.14573
Stox1	-3.81161	6.1E-05	0.25178	0.75822	0.75491	0.29644	0.27196	0.43347
Prr36	-3.81181	0.00204	0.28416	0.68459	0.71405	0.28252	0.31295	0.50734
Slc2a4	-3.81937	0.00504	0.33301	0.85574	0.41079	0.18819	0.38932	0.30576
Grip1	-3.82328	2.4E-06	0.29471	0.48516	0.41403	0.16655	0.21721	0.16912
Atp7b	-3.82344	5.4E-12	1.45998	4.51817	5.27119	3.18631	1.42088	1.59394
Ryr2	-3.82447	0.00454	1.0036	3.48872	1.5216	0.58271	0.587	1.6305
Slc23a1	-3.82558	2.4E-06	0.78691	1.95324	1.69139	1.03317	1.09088	1.42318
mt-Cytb	-3.8266	0.00402	2050.93	1958.55	2065.15	627.454	593.396	1972.66
Dsc2	-3.83561	0.00086	0.17558	0.22913	0.24066	0.09647	0.16734	0.10076
Cbs	-3.838	0.00042	0.06929	0.55644	0.47486	0.32632	0.15849	0.15906
Gm20661	-3.84394	0.01698	2.57376	4.69349	3.96865	1.2121	1.33551	2.17449
Dcxr	-3.84643	6.4E-18	7.39056	17.5611	18.7331	10.4258	8.07937	6.19724
Wdr78	-3.84883	1.7E-13	1.28389	3.1949	2.76996	1.31379	1.45829	1.21282
mt-Nd6	-3.85004	0.00575	970.962	937.613	1002.18	269.465	261.802	965.093
Ugt1a6a	-3.85095	2.7E-06	1.28927	2.63151	1.84075	1.93959	1.57008	1.27423
Cpne5	-3.858	0.01993	0.01931	0.05426	0.04631	0.04849	0.04907	0
mt-Co1	-3.85837	0.00689	4152.25	3010.95	3374.3	1030.97	1094.63	3485.4
Atp6v1c2	-3.86806	1.7E-07	2.294	3.5496	3.68106	1.89721	1.27981	2.14059
Dedd2	-3.88466	1.3E-14	2.26565	5.22503	3.45042	2.74806	1.65363	1.83731
Asb2	-3.88508	3.1E-05	0.58858	1.31294	0.69468	0.55438	0.46539	0.47538
Tmem107	-3.88798	5.3E-08	0.93061	1.97023	2.14522	1.27497	0.86006	0.77681
Eva1c	-3.88997	2.1E-11	0.73034	2.0283	1.54325	1.12739	0.7734	0.53554
Fsd2	-3.898	1.4E-05	0.40689	1.79716	1.15524	0.4745	0.6057	0.66716
Inpp5j	-3.89927	0.01802	0.2502	0.27398	0.07794	0.09998	0.11562	0.15663
Kctd8	-3.90078	4.8E-05	0.51506	0.52391	0.72949	0.30186	0.36653	0.34156
Gfra4	-3.90098	0.03234	0.10699	0.2148	0.25663	0.06718	0.16315	0.08187
Synpo2l	-3.90292	0.03923	0.07506	0.24111	0.13718	0.07856	0.07631	0.09572
Zcchc12	-3.9089	0.00994	0.15239	0.40793	0.2785	0.19138	0.1291	0.15547
Atp8a2	-3.91265	0.00102	0.1263	0.23183	0.09892	0.07931	0.10089	0.06903
Rwdd3	-3.91443	0.00268	0.18293	0.66107	0.68952	0.14359	0.31383	0.38493
Nr4a2	-3.9194	5.8E-06	0.37094	2.19976	1.2124	0.73461	0.49313	0.72049
Wdr54	-3.94017	1.8E-05	0.2471	0.69452	0.45964	0.36574	0.2512	0.44567
Efcab1	-3.9431	4.9E-08	0.92878	2.94614	2.57787	0.87481	1.3691	1.17261
6820408C15Rik	-3.94767	2.2E-05	2.46286	3.90355	2.66501	1.38371	1.58132	1.98367
Cracr2a	-3.9491	9.3E-07	3.3899	2.6866	2.49562	2.11003	1.7231	1.48381
Cfap45	-3.95363	8.9E-08	1.30184	4.36203	3.53794	2.11413	1.09528	1.30526
Esyt3	-3.95611	8.3E-07	0.27416	1.12083	1.00775	0.3443	0.43073	0.53397
Casq1	-3.95772	0.01137	0.23746	0.28603	0.44752	0.18638	0.21122	0.13627
Plin4	-3.96856	0.00016	1.04251	3.17166	2.39067	1.14559	0.55029	1.50334
Kif21a	-3.97854	3.5E-18	2.59363	5.88935	4.90335	2.68441	2.24575	2.2446

Pih1h3b	-3.97885	0.03382	0.23026	0.61636	0.2104	0.19278	0.19507	0.17618
2010007H06Rik	-3.98549	1.5E-05	1.64316	1.88506	1.38348	0.63381	0.87102	1.2034
Jph1	-3.99038	0.00168	0.32442	0.31636	0.46055	0.27647	0.32981	0.24824
Barhl1	-3.99579	0.00756	0.23727	0.23818	0.30489	0.05587	0.16583	0.20425
Dnah7b	-4.0012	1.7E-06	0.713	1.64325	1.58508	0.42951	0.62761	0.81613
Gsto1	-4.00932	8.1E-08	23.9524	28.5926	18.351	13.6362	15.9144	9.23131
1700029J07Rik	-4.01501	6.9E-06	1.14128	3.66603	2.83889	0.84936	1.39658	1.55248
Magix	-4.01527	1.5E-05	0.199	0.92689	0.87283	0.34989	0.32369	0.42635
Rfx2	-4.02172	1.2E-13	1.10534	3.05128	2.39089	1.2797	1.07103	1.16293
Myo1h	-4.02288	2.5E-06	0.66439	1.39662	1.25883	0.55216	0.55623	0.80741
Pbx4	-4.0259	0.00031	0.26121	1.17467	0.57283	0.26243	0.37176	0.43971
Ift81	-4.02981	3.3E-11	2.25989	6.03155	5.26116	2.58768	1.97646	2.41577
Ttc39a	-4.03229	1.6E-09	2.19641	3.20523	3.2047	1.39402	1.60864	1.64447
Dnah7a	-4.04593	3.1E-05	0.56245	1.52987	1.51074	0.35318	0.48879	0.76355
Fam81a	-4.06269	3.6E-07	0.34243	1.39608	1.48023	0.4962	0.53556	0.60464
Rabl2	-4.06276	2.3E-10	1.36948	3.29929	2.54744	1.22848	1.21323	1.34727
Zdhhc1	-4.06404	5.7E-11	5.07858	11.1108	10.4225	4.27497	4.1722	4.97821
Mok	-4.06512	4.5E-08	1.19051	2.34912	1.65506	0.81163	0.8299	1.09312
BC049715	-4.06527	0.02893	0.31794	0.54711	0.59141	0.1426	0.36939	0.31278
Sptbn2	-4.07164	9.7E-11	0.44918	1.01452	0.94274	0.50241	0.49232	0.55851
Gm16136	-4.07222	0.00077	0.49665	1.91441	1.42953	0.49898	0.55539	0.98805
Caps2	-4.07781	0.00252	0.20307	0.40769	0.25514	0.12752	0.17204	0.18128
Obscn	-4.07838	0.04361	0.89375	1.95042	0.29115	0.28438	0.2302	0.94147
BC030870	-4.10024	0.01061	0.15371	0.74061	0.5267	0.28955	0.23439	0.23522
Tmem25	-4.10262	0.01042	0.21635	0.23165	0.14827	0.18114	0.05499	0.08277
Pla2g3	-4.10686	0.02733	0.14798	0.21787	0.16902	0.07743	0.07522	0.15097
Hs6st2	-4.11138	3.3E-10	0.22253	0.54802	0.49818	0.2981	0.19606	0.19297
Tnnt3	-4.12611	0.00962	1.17063	1.41011	0.80225	0.41348	0.48347	0.89572
AU022754	-4.12744	0.00568	0.11675	0.68756	0.58676	0.14663	0.2176	0.35734
Abcg4	-4.12758	0.03534	0.06668	0.05355	0.13709	0.04187	0.03389	0.06803
C2cd4b	-4.13252	0.00202	0.78551	1.19152	1.61497	0.82207	0.6211	0.86817
Gabre	-4.13295	1.2E-07	0.31792	1.19796	0.90501	0.42997	0.34806	0.4678
Rab44	-4.13383	0.00011	0.81741	0.74697	0.44429	0.53098	0.25789	0.36665
Ccdc189	-4.13498	1.6E-06	1.08179	2.11753	1.4364	0.95525	1.04821	0.62081
Cpxm2	-4.13555	0.00031	0.35082	0.98603	0.39498	0.34617	0.43306	0.21091
Bzrap1	-4.13646	0.00048	0.80472	1.49809	1.46229	0.35219	0.57638	0.86764
Pdlim3	-4.13913	1E-09	0.40504	1.35528	0.87901	0.50867	0.46896	0.39601
Smim22	-4.14348	6.6E-07	2.06451	6.30005	6.22533	2.98165	2.93832	1.73766
Ccdc57	-4.14764	1.4E-06	0.99017	1.8885	1.67525	0.79663	0.67632	0.87602
Pifo	-4.15212	3.1E-06	2.15812	3.13605	3.73271	1.03249	1.8283	1.88722
Arhgef38	-4.15661	4.8E-18	1.01819	1.78295	1.29865	0.83471	0.79789	0.57349
Cacna1g	-4.15955	2.6E-05	0.10283	0.37618	0.18009	0.10762	0.13357	0.09617
Ccno	-4.16142	0.00171	0.30869	0.4462	0.59234	0.31013	0.21967	0.18896
2610028H24Rik	-4.16208	1.3E-08	3.66817	8.12803	7.96057	3.36972	3.72909	3.79431
Hs3st6	-4.16318	0.0167	0.19002	0.76299	0.9767	0.11932	0.33806	0.5816
Lpar3	-4.16586	4.2E-09	0.70507	1.88735	2.93139	1.15111	0.86013	0.86319
Plch1	-4.16825	1.7E-08	0.50957	0.94764	0.87304	0.24419	0.4635	0.47199
Rph3al	-4.16882	2E-13	1.27541	1.73141	1.7065	0.93434	0.83352	0.76677
Hs6st3	-4.16934	0.00023	1.39344	0.92153	0.89877	0.56616	0.62496	0.56446
Fam19a5	-4.17164	0.01159	0.13493	0.21672	0.27742	0.05649	0.13718	0.13766
2010109I03Rik	-4.17741	0.0271	0.07015	0.22534	0.14423	0.06607	0.10697	0.05368
Fsip1	-4.17743	0.00019	0.35186	1.3186	1.28604	0.55235	0.29808	0.71794
C330002G04Rik	-4.18169	0.00018	0.33832	0.72451	0.73422	0.21244	0.31528	0.43145
Mdm1	-4.18441	1.1E-09	0.80915	2.09705	1.99126	0.83142	0.74781	0.9287
Ccdc148	-4.18775	1.2E-05	0.09991	0.4513	0.29955	0.12547	0.1587	0.14334
Dnah11	-4.19755	2.1E-10	1.6422	4.03994	3.65078	1.34306	1.51474	1.66723

Acot1	-4.20982	1.1E-05	0.52003	4.41478	5.70223	2.70566	1.54825	1.36427
Enkur	-4.21043	5.5E-07	1.03874	3.63455	2.54238	1.72381	1.01827	0.96512
Ubxn11	-4.21287	0.0005	2.36914	4.74654	3.41461	0.87419	1.5891	2.44819
Ccdc65	-4.21372	9.6E-09	1.16432	2.16528	1.88983	1.15438	0.71642	0.89089
mt-Nd4	-4.21381	0.00217	1689.88	1520.67	1603.41	446.86	420.857	1480.12
Trpm3	-4.21866	0.00093	0.02874	0.29999	0.30524	0.09022	0.10225	0.12093
Cnn1	-4.2204	8.6E-07	2.32824	7.78471	5.39597	3.93402	2.08718	1.84626
Pcyt1b	-4.22168	1.1E-05	0.34592	0.55559	0.45932	0.16291	0.30771	0.2316
Cfap126	-4.22216	6.3E-12	2.08918	5.24288	4.07156	3.03368	1.89158	1.09901
Esrrg	-4.22336	5.1E-06	0.14925	0.37455	0.42192	0.18744	0.15173	0.15703
9530053A07Rik	-4.22391	8.1E-06	0.07824	0.22775	0.12734	0.07369	0.08451	0.05986
Nphp4	-4.22651	1E-08	0.59404	1.09259	0.93241	0.33692	0.56495	0.43988
Dnaja4	-4.23542	3.2E-11	2.77527	10.6769	6.05968	4.33642	2.64093	2.568
Traf3ip1	-4.24233	3.9E-09	8.08113	17.3138	14.4975	5.66214	5.42403	7.85457
Tmc5	-4.24492	0.00061	1.68417	1.97669	0.76354	0.6996	0.89558	0.8525
Egln3	-4.24573	0.00023	0.56561	0.41927	0.50689	0.19124	0.48654	0.09987
4933406C10Rik	-4.24847	0.00408	0.2705	0.32584	0.21628	0.08493	0.20624	0.10349
Iqcg	-4.25601	2.3E-10	1.47199	3.76118	3.46196	1.44948	1.05431	1.58708
Htr1b	-4.26446	7.1E-07	0.31872	0.84661	0.45366	0.29251	0.31155	0.15008
Rgs11	-4.26572	4.3E-06	1.23915	3.53813	3.4912	1.24496	1.17575	1.64346
Vipr1	-4.26581	8E-07	0.25959	1.10648	0.39686	0.30094	0.34511	0.22919
Gck	-4.26987	0.00234	0.5728	0.7973	0.65424	0.19183	0.29115	0.52594
Adcyap1r1	-4.27232	2.4E-06	0.10527	0.38043	0.31263	0.11017	0.1427	0.1611
Rhov	-4.27266	0.00327	0.27204	0.3823	0.55929	0.29893	0.13828	0.20815
Ankrd42	-4.27671	7.9E-07	1.43099	3.48395	3.06658	0.79872	1.22384	1.61634
Bspry	-4.27678	3.9E-14	1.57577	2.8472	2.59852	1.05131	1.60194	0.94197
Hspa4l	-4.27986	0	2.15098	8.32913	6.44838	3.57094	2.46629	2.12683
Hcn4	-4.28113	0.0071	0.03786	0.10642	0.14271	0.07132	0.04811	0.02897
Cfap43	-4.2895	3.9E-09	4.93293	9.37094	7.12802	3.41897	3.32944	3.8012
Igsf11	-4.29369	4.3E-05	0.20622	0.31051	0.47698	0.12949	0.20965	0.21697
mt-Nd2	-4.3007	0.00135	1089.46	1050.96	1138.44	299.102	271.5	951.177
Pih1d2	-4.308	8.6E-10	0.57642	2.08302	1.58012	0.64634	0.89992	0.53557
F5	-4.31	0.00581	0.56117	0.37554	0.17092	0.11746	0.22978	0.2624
Mns1	-4.31016	4.9E-11	0.69107	2.77482	2.02761	1.22047	0.83428	0.77665
Rsph10b	-4.31287	3.8E-06	2.61443	4.91887	4.22333	1.57132	1.12009	2.77207
Syne4	-4.31455	4.8E-05	1.17942	2.33627	1.85904	0.74059	0.89926	0.99269
Cep126	-4.31543	1.1E-15	1.68108	4.79551	3.71416	1.81185	1.41567	1.70867
2410004P03Rik	-4.3325	1.1E-09	2.22666	4.89104	4.48815	1.56268	1.7976	2.20487
Gm13111	-4.33402	4.8E-07	0.19619	0.73525	0.94118	0.24639	0.41552	0.27522
Pi16	-4.3445	1.3E-09	0.75528	2.51942	3.02601	1.36806	0.67923	0.978
1700012B09Rik	-4.35347	0.01823	0.28283	0.79495	0.96914	0.71039	0.07188	0.32462
Phkg1	-4.35389	0.00282	0.50169	0.46044	0.22103	0.15751	0.18215	0.1371
Tspan1	-4.3568	7.4E-16	4.97291	8.34642	6.81605	4.09066	3.71579	2.62551
Efhc1	-4.36724	3.3E-08	1.41627	3.53837	4.85297	1.51513	1.54644	1.60544
Saxo2	-4.37455	1.9E-10	0.65366	2.46908	1.57618	0.63848	0.82449	0.7595
Chchd6	-4.37765	2.1E-11	1.09911	1.98595	1.75757	1.12152	0.72163	0.63075
Pnck	-4.37797	0.00062	0.29381	0.61345	0.84568	0.11069	0.32856	0.44962
Klhdc7a	-4.3872	2.2E-12	1.24617	3.90763	2.56206	1.31349	1.13113	1.23731
Sphkap	-4.38831	0.00446	0.02412	0.11624	0.08266	0.04544	0.03066	0.03692
Tctex1d4	-4.39178	6.3E-07	1.66796	7.86932	3.64358	2.09472	2.38453	1.75486
Elmod1	-4.39302	8E-13	0.57036	1.43134	1.31922	0.5596	0.70667	0.38187
Slc5a3	-4.39534	0.00352	1.16743	4.50001	2.58928	2.65234	1.86653	1.2993
Casc1	-4.39617	1.7E-08	0.5	1.62437	1.27719	0.47095	0.42166	0.64344
Rsph4a	-4.40001	2.9E-13	1.49137	5.0454	4.52317	1.87295	1.64517	1.67529
Tmem28	-4.40494	0.03899	0.02963	0.19035	0.18275	0.11163	0.03012	0.06801
Foxo6	-4.4062	0.00034	0.08858	0.60465	0.60706	0.30592	0.15759	0.20334

Cpe	-4.40668	2.4E-06	2.04204	5.56127	2.89016	2.11851	1.82774	1.25679
1700007K13Rik	-4.40868	1.9E-07	0.84014	8.578	5.67537	3.08992	2.01322	1.92853
Nov	-4.41301	3.2E-07	0.76701	1.71818	1.79827	1.01396	0.63611	0.49422
Fbxo36	-4.41716	1.2E-06	0.59312	1.0392	0.9238	0.67715	0.43852	0.16503
Morn1	-4.41993	1.6E-09	0.50939	2.19604	1.61686	0.62289	0.72226	0.67696
Agr3	-4.42181	0.00043	0.59578	1.14825	1.79651	0.82303	0.66624	0.27352
Ankrd45	-4.42811	4E-07	0.3411	1.44431	0.97755	0.36996	0.39405	0.54572
Efcab2	-4.43864	1.9E-08	0.44642	1.45641	1.45323	0.54313	0.45384	0.59778
Gm281	-4.45085	3.2E-08	0.82392	2.237	1.96281	0.96081	0.67806	0.78053
1810010H24Rik	-4.46001	2.8E-10	2.47448	11.15	8.66743	3.40972	2.69026	3.78675
D430036J16Rik	-4.46003	5.7E-06	0.46659	1.08246	0.604	0.58598	0.31623	0.07934
Iyd	-4.46136	8.2E-06	0.28544	0.97421	1.07591	0.58253	0.32646	0.27301
Ccdc96	-4.46387	8E-08	0.61383	1.58257	1.35056	0.85202	0.39412	0.4944
1810041L15Rik	-4.46434	5.5E-07	0.10608	0.49692	0.36348	0.21093	0.18872	0.06764
Ubxn10	-4.46457	2.6E-20	1.47185	3.58666	3.00866	1.45006	1.49629	0.99028
Foxa1	-4.46725	3.5E-13	2.36143	5.19308	3.63502	2.28125	1.90205	1.36211
Spata18	-4.47166	5.1E-14	5.05713	13.8608	11.7157	4.48714	4.13524	5.29957
Ak8	-4.47598	5.4E-08	0.74203	1.2911	1.27132	0.69892	0.44004	0.52046
Tst	-4.48135	9E-11	3.48726	11.7958	10.2837	5.37484	3.59889	3.15347
Mlxipl	-4.48833	0.00125	0.10457	0.21833	0.28665	0.10506	0.07441	0.06401
Gsto2	-4.49794	0.00938	0.2987	0.41977	0.4094	0.23445	0.22774	0.28569
Gfra1	-4.50116	2.4E-09	0.65566	2.37787	2.75399	0.67733	0.87081	0.98719
Lrrc51	-4.50331	1.1E-08	4.40304	9.9158	8.59327	3.66636	3.45443	3.58877
Pthlh	-4.50648	0.03753	0.14307	0.28724	0.29416	0.31444	0.03636	0
Wdr38	-4.51148	0.01185	0.32273	0.74511	0.82939	0.30398	0.24607	0.40128
Rsph9	-4.51208	1.3E-12	1.44593	3.77376	3.31959	1.49811	1.17596	1.54892
Ankub1	-4.51517	0.00313	0.6296	0.52758	0.60032	0.08594	0.34786	0.41891
Gjb2	-4.51557	0.0006	0.09628	0.5412	0.49484	0.27204	0.17128	0.1105
Proser2	-4.51605	1.1E-09	0.20589	0.79752	0.4482	0.32701	0.22162	0.14827
Spag17	-4.52106	1.6E-10	1.32341	3.07874	2.62738	0.94238	1.01251	1.26317
Cfap54	-4.52525	2.7E-10	1.44594	3.51557	3.21915	1.03837	1.20601	1.38756
Endog	-4.52746	0.00061	5.22984	11.812	9.78162	3.55763	3.21217	6.33599
St8sia6	-4.53362	4.7E-09	1.26942	1.97813	1.30493	0.75915	0.96019	0.57816
4921536K21Rik	-4.53447	0.01353	0.08944	0.35911	0.42904	0.08424	0.09092	0.27373
Efnb3	-4.54404	0.00021	0.14555	0.55519	0.47379	0.27418	0.09248	0.22273
2010300C02Rik	-4.5483	1.3E-12	2.05394	5.7363	4.9266	1.94892	1.85604	1.92084
Gm10069	-4.54884	0.00194	0.07951	0.44695	0.23612	0.14978	0.10777	0.12168
Cldn8	-4.54885	0.02398	0	0.3553	0.43797	0.06174	0.22489	0.07523
Wif1	-4.55092	0.00098	0.09544	0.53651	0.39245	0.17979	0.1698	0.14606
4930451G09Rik	-4.55517	0.00455	0.105	0.29513	0.50373	0.16484	0.18681	0.08035
Gabrp	-4.56522	1E-08	1.88631	8.59304	9.61313	3.29497	2.23143	2.99163
Fbxo16	-4.57605	0.00566	0.37726	1.1361	0.51709	0.17767	0.28764	0.50517
mt-Nd4l	-4.58362	0.00253	1062.65	932.905	932.699	260.786	308.43	937.826
Clstn3	-4.5951	0.04513	0	0.14977	0.14379	0.07319	0.0474	0.01784
Morn3	-4.60805	3.7E-05	1.45523	2.40599	2.17058	1.07504	0.95726	1.11349
Dnah7c	-4.61526	1.1E-06	0.83627	2.01471	1.45482	0.4501	0.51383	0.80865
Syngap1	-4.62326	1.4E-07	3.24232	6.63271	5.9059	2.03595	2.06444	2.8204
4932411E22Rik	-4.62387	3E-11	0.56287	1.3711	1.27295	0.57729	0.42916	0.48811
Adgb	-4.62951	2.3E-11	1.38414	3.42474	2.70473	1.15103	1.02683	1.20222
Wnk2	-4.63301	0.00186	0.17753	0.2961	0.18718	0.0686	0.09024	0.14629
Lrrc4b	-4.64915	0.00016	0.11863	0.73035	0.43358	0.2483	0.22109	0.12103
Lrriq3	-4.6588	0.00589	0.11673	0.14998	0.08	0.04398	0.05933	0.05359
Reep6	-4.66317	5.1E-10	0.87812	2.78358	2.88226	1.30594	0.82223	0.88408
1700040L02Rik	-4.66961	2.1E-06	0.82597	1.98988	1.94973	0.92204	0.51314	0.84267
Ltf	-4.67352	1.2E-05	4.5992	11.4845	7.89128	3.49929	2.96916	3.95582
Myb	-4.67931	5.7E-10	1.77386	2.56409	3.47865	0.90822	1.01263	1.33641

Adam28	-4.68854	1.2E-14	1.82541	3.31091	3.06276	1.20552	1.18383	1.34857
Klhl41	-4.68891	0.00755	0.13739	0.55165	0.47078	0.11503	0.16295	0.24529
Fam81b	-4.69777	6.5E-07	0.32727	1.15637	0.62799	0.3699	0.23289	0.37562
Cecr6	-4.69923	0.00155	0.09515	0.15282	0.13041	0.07468	0.06046	0.0364
Bdh1	-4.70161	2.3E-08	0.36574	0.80771	0.58485	0.36363	0.34083	0.16325
Ccdc121	-4.70291	3.8E-06	0.95508	2.35206	2.05087	0.71967	0.64729	1.02311
Nek11	-4.71007	1.7E-06	0.79104	1.59907	1.1777	0.34256	0.41595	0.79312
Srcin1	-4.71201	1.4E-07	1.09058	2.00458	2.05948	0.54784	0.64058	1.01063
Gm26840	-4.71231	0.00919	0.23319	0.78027	0.47943	0.12202	0.19755	0.20817
Col4a6	-4.7198	3.1E-21	1.10401	2.74138	2.45894	1.11283	0.87129	0.87809
Slc35g3	-4.72348	0.00869	0.14316	0.28742	0.49056	0.17979	0.18193	0.05477
Kcnd3	-4.72472	5.3E-06	0.08457	0.36866	0.21526	0.07586	0.09211	0.1479
Kcnh3	-4.72838	1.9E-05	0.54952	0.98642	1.2184	0.24357	0.46006	0.51941
Cdkl4	-4.73106	1.8E-10	0.47418	1.99916	1.9498	0.7692	0.66283	0.57448
Agbl2	-4.73189	1E-13	0.41799	1.27275	1.21745	0.43745	0.47805	0.39979
H2-M5	-4.73766	0.00551	0.08608	0.51844	0.35395	0.1081	0.13126	0.19759
Sh3bgr	-4.75176	2.4E-06	0.60088	0.96507	1.3898	0.5188	0.5345	0.28736
BC051019	-4.75609	1.1E-12	2.12615	6.42523	5.44491	2.07287	2.13302	1.96935
Spag6l	-4.75973	3E-14	1.01618	3.34578	2.33296	0.82952	1.1751	0.93305
Rsph14	-4.77677	9.1E-07	0.34607	0.71023	0.84327	0.24145	0.31273	0.32365
Hcar2	-4.78391	1.6E-07	0.72012	1.10839	0.90477	0.37682	0.42705	0.45917
Riiad1	-4.78431	4.4E-06	1.13967	2.28804	2.14785	0.71563	0.79654	1.09004
Myo5c	-4.80273	1.4E-13	6.03163	12.3251	12.1224	4.50742	3.74586	4.79796
Frem2	-4.80795	1.3E-06	0.2534	0.50521	0.32935	0.14265	0.10659	0.22732
4930562C15Rik	-4.80806	1.3E-08	2.89711	8.43148	6.82216	2.2432	2.17017	2.98346
Spa17	-4.81098	2.9E-05	1.63741	3.41879	2.99238	1.64508	0.8323	0.75173
Map3k19	-4.82178	5.6E-10	1.28082	3.16187	2.42199	1.02767	0.95245	1.01633
Slc6a15	-4.829	7.3E-06	0.15896	0.53615	0.50112	0.19963	0.17776	0.17028
Tbx1	-4.83268	2.6E-05	1.56424	6.33317	5.13669	1.14594	1.35831	2.54342
Hspa1a	-4.83644	0.00336	4.2549	18.0249	5.77842	8.55458	1.56752	3.49464
Prom1	-4.84227	6.6E-15	2.24374	5.05552	3.97141	1.7434	1.59178	1.66743
Nr1i2	-4.84401	0.01859	0.18146	0.4736	0.21763	0	0.1153	0.2777
Dusp26	-4.8447	0.02142	0.10049	0.20175	0.24105	0.0631	0.1277	0.03845
Cfap44	-4.84534	6.6E-06	5.23661	12.0682	11.0746	2.52611	3.02917	5.50944
Sobp	-4.8499	1.4E-06	0.41895	0.70652	0.45938	0.24992	0.31943	0.16028
Spef2	-4.85683	2.5E-12	1.35488	3.41419	2.6495	1.04879	0.97367	1.12608
Dnah12	-4.85696	7.5E-07	4.89494	10.2924	8.15402	2.0513	3.05577	4.32841
Myh11	-4.87224	0	12.0674	29.3948	23.9962	13.0605	7.5187	8.07099
Mgst2	-4.87224	3E-05	1.15725	1.15539	0.5573	0.3928	0.60413	0.40685
Stmnd1	-4.87356	3.2E-06	2.03937	2.72953	2.96993	0.90708	0.90704	1.62547
Grb7	-4.87697	8.6E-20	1.13582	2.37704	2.47672	1.10224	0.85726	0.76374
4930412C18Rik	-4.88521	6.8E-07	0.16955	0.42359	0.32276	0.16561	0.14364	0.1009
5330417C22Rik	-4.88578	1E-14	2.00258	6.60975	6.81167	3.20715	1.45684	1.78534
Rsph1	-4.88792	1.7E-12	3.88054	12.9445	9.4102	4.74845	3.23691	3.34992
Stk36	-4.88817	1.9E-09	1.25949	3.04875	2.66344	0.88126	0.82312	1.18399
Gm1661	-4.88827	0.00093	0.75238	1.13287	1.22459	0.47244	0.47805	0.43177
Acss1	-4.89228	3.3E-16	3.36684	8.04362	8.09497	2.30793	2.68116	3.17729
Tmprss13	-4.89739	0.00206	0.07138	0.25796	0.2446	0.08965	0.127	0.02731
C230062I16Rik	-4.89855	0.00035	0.31409	1.57642	1.7758	0.39445	0.71843	0.54074
Ttll3	-4.90116	4.2E-05	4.27343	7.73073	5.9203	1.88993	1.88024	3.56873
Fxyd3	-4.9071	4.6E-05	0.36421	0.60446	0.416	0.51838	0.22216	0.16721
Ccdc162	-4.90827	5.7E-07	2.15176	4.90963	3.93237	0.96511	1.23842	2.09944
Scara5	-4.91129	3.6E-05	0.19009	0.80904	0.83372	0.39389	0.13527	0.24726
Prob1	-4.91224	5.5E-09	2.6301	7.38285	6.36543	1.84494	1.74639	2.86458
Rsg1	-4.91433	0.00798	0.18492	0.2475	0.42242	0.11611	0.09399	0.18865
Fam166b	-4.91629	4.1E-08	1.11612	2.55102	3.35379	1.29387	0.91646	0.91971

Ttc6	-4.91643	1.1E-09	0.43333	1.01611	0.79587	0.25033	0.31718	0.35809
Kif17	-4.92297	0.00384	0.20222	0.20299	0.24252	0.09523	0.06424	0.13539
Fam227a	-4.92378	1.5E-09	0.70578	1.53031	1.30595	0.52074	0.42153	0.58054
Tppp3	-4.9238	1.4E-22	13.8507	31.9088	30.9675	13.6165	10.1623	9.49506
Fam84a	-4.93123	5.2E-09	2.19368	5.14727	3.19465	1.44204	1.49835	1.57361
Mak	-4.93377	3.4E-12	1.52693	2.84374	2.49362	0.9996	0.85871	1.14349
Ccdc39	-4.94312	8.6E-09	2.39637	5.46081	4.66022	1.38749	1.43164	2.22653
Prom2	-4.95325	0.00023	0.06699	0.2869	0.30605	0.0701	0.0908	0.13668
Adamdec1	-4.96114	7.3E-08	1.05073	1.76429	1.4729	0.62979	0.65547	0.62126
1700016K19Rik	-4.96429	1.9E-09	3.5211	9.59372	5.34324	3.63236	2.04548	2.40555
Vwa3a	-4.96769	1.3E-08	2.324	4.66573	4.37335	1.26792	1.4621	1.924
Ttc29	-4.96828	1.4E-08	0.98124	2.33769	1.97256	0.75992	0.51539	0.95103
Agbl4	-4.96852	0.00024	0.12066	0.21317	0.1323	0.05304	0.04293	0.11079
Cfap61	-4.96885	8.6E-11	0.75349	1.40311	1.36205	0.47314	0.44406	0.57654
Chrm1	-4.96923	0.02352	0.01785	0.10036	0.13459	0.02242	0.02723	0.06831
Dixdc1	-4.99045	5.3E-18	0.78986	1.57642	1.58412	0.54703	0.47824	0.6577
Zdbf2	-4.99271	8.2E-07	0.09149	0.18368	0.10659	0.07469	0.05116	0.042
Gm973	-4.99727	4E-08	0.99262	2.2048	1.62104	0.5172	0.72999	0.80799
Adgrv1	-4.99799	6.8E-07	0.21974	0.55943	0.49985	0.14386	0.14259	0.22538
Cdr1	-5.00695	4.6E-05	0.20029	0.56296	0.61769	0.15721	0.25452	0.19157
Cadm4	-5.01426	8.4E-09	1.44707	2.15198	1.17535	0.97597	0.79004	0.41009
Btg4	-5.02926	0.04072	0.13594	0.14555	0.15527	0	0.09213	0.10401
Clic6	-5.04656	6.6E-15	3.87642	9.02996	7.32081	2.67567	2.69238	2.96609
Zmynd10	-5.0601	2.4E-11	2.12163	4.32497	3.6909	1.43471	1.272	1.29039
Slc7a3	-5.06403	0.02842	0.03061	0.07374	0.18878	0.07688	0.04667	0
Spag8	-5.06536	7.3E-06	1.32405	2.12655	3.00872	1.00644	0.67302	1.06643
Pclo	-5.06897	6.8E-06	0.10315	0.36861	0.46301	0.1263	0.08651	0.14996
Pde6h	-5.08369	0.00339	0.17714	0.20152	0.23268	0.03708	0.09754	0.12424
Kif6	-5.09155	7.3E-10	0.65467	1.43804	1.21402	0.29018	0.66554	0.42726
Fam161b	-5.09454	2.1E-10	0.79601	1.57811	1.55132	0.57794	0.53106	0.57101
RP23-163N19.1	-5.09456	1.3E-06	0.13365	0.4293	0.53123	0.15106	0.14945	0.18407
Ngb	-5.09644	0.00092	0.13444	0.53981	0.46067	0.16884	0.20501	0.10287
Retnlg	-5.10301	0.03426	1.90492	2.29462	1.95821	0.71769	1.64607	0.43727
Klk11	-5.1048	0.01485	0.9474	1.14121	0.81158	0.34702	0.24078	0.18123
Ddo	-5.10878	3.4E-13	0.88243	2.10507	1.84092	0.5704	0.6926	0.70499
Mapk15	-5.11033	7E-05	6.37661	12.4568	10.1657	1.84584	2.68955	6.15949
1700095J03Rik	-5.12069	0.03675	0.06406	0.61731	0.70242	0.36202	0.06512	0.19606
Spire2	-5.12187	3.4E-08	1.61501	2.37153	2.11872	1.04309	0.68019	0.67083
Lrrc6	-5.12349	2.7E-08	1.16708	2.67779	2.5899	0.69795	0.70623	1.2332
Ccdc150	-5.12472	0.00197	0.55361	0.74096	0.86945	0.15933	0.26968	0.44125
P2rx6	-5.13372	1.9E-05	1.33359	3.86469	2.94048	0.60074	0.79576	1.59717
Gpr155	-5.1351	5.8E-17	1.2312	3.99663	3.4107	1.53532	1.27809	0.76958
Cerkl	-5.13951	0.00654	0.41646	0.38724	0.15021	0.05505	0.08913	0.28512
Scgb3a2	-5.14495	7.1E-09	42.2477	212.969	232.707	72.9742	56.3292	54.3808
Shank2	-5.1536	4E-13	1.53809	3.32544	2.98533	1.00633	0.90756	1.20158
Mfsd4b1	-5.15937	1.5E-05	0.6366	1.56775	1.27974	0.53299	0.25887	0.617
Rbm24	-5.16072	2.7E-08	1.00869	1.67792	1.20973	0.4298	0.49441	0.71668
Stk33	-5.17054	3.8E-12	0.69823	2.6634	1.52525	0.54805	0.73201	0.70122
Gm867	-5.17243	3E-07	5.88107	11.807	9.68597	3.0377	3.80904	4.20966
Bpifb5	-5.17405	0.00349	1.27352	0.79543	0.38789	0.31099	0.39559	0.43309
Car11	-5.183	0.01711	0.12027	0.33804	0.24727	0.11328	0.12227	0.04601
1700001L19Rik	-5.18887	9.9E-06	0.51247	1.0803	1.09752	0.36202	0.26049	0.53917
Lrrc48	-5.19071	2.1E-12	1.58902	3.88994	3.42503	1.35185	1.05523	1.19625
Galntl6	-5.20821	0.01994	0.28119	0.18818	0.06423	0.03924	0.07147	0.14344
Diras2	-5.21469	3.7E-17	0.66593	1.96798	1.71597	0.70251	0.62283	0.48917
Fam179a	-5.21673	6.6E-07	2.69778	6.63166	6.05459	1.55177	1.31895	2.74182

1700024G13Rik	-5.22309	4.2E-05	2.32802	4.44011	1.79487	1.64456	1.03543	0.89066
Rapgefl1	-5.22813	3.4E-05	0.40803	0.44235	0.29361	0.14092	0.13481	0.21855
Spag16	-5.23228	3.1E-17	0.92464	1.92508	1.95968	0.59136	0.67888	0.6944
Tmem212	-5.23237	3.1E-16	6.47035	15.1983	14.5221	4.67236	5.34446	4.8271
Gm20755	-5.23247	0.00518	0.19018	0.50908	0.26067	0.03981	0.16111	0.19402
Ttll10	-5.2522	0.0005	1.27881	2.12391	1.61335	0.4015	0.48752	1.06745
Lrrc10b	-5.26102	6.6E-09	3.17848	7.38875	5.84692	1.71574	1.95576	2.68806
Hepacam	-5.26587	1E-06	0.17302	0.47241	0.71144	0.19556	0.17589	0.21182
Dnali1	-5.2709	4.8E-16	2.00905	6.19185	5.35851	2.28441	1.68362	1.53725
Erich2	-5.27184	1.2E-07	1.88579	4.32231	3.58094	0.91278	1.21817	1.38282
Fhdc1	-5.27358	1.3E-12	1.36693	5.54788	5.94981	1.97184	1.03283	1.75262
Scn5a	-5.27454	1.5E-07	0.75327	2.09068	1.73889	0.53939	0.36946	0.79884
Ltk	-5.27856	3.3E-05	0.16113	0.77637	0.57421	0.18212	0.18018	0.24658
Dydc2	-5.28121	0.0001	0.9782	2.04242	1.87706	0.61424	0.79556	0.59879
Fbxl13	-5.28592	4.9E-06	0.46407	1.25112	0.88596	0.22896	0.37068	0.40582
Arhgdig	-5.2882	9.4E-13	1.78074	3.71806	3.85637	1.43127	0.94136	1.3353
Sox9	-5.28996	0.00056	0.53218	0.26992	0.17276	0.12312	0.21356	0.10716
Spata17	-5.29849	3.5E-10	0.68604	1.94443	1.49343	0.70952	0.43077	0.55581
Cidec	-5.30346	0.03069	0.37442	0.50112	0.59872	0.35266	0.03172	0.23874
Ovol2	-5.3168	1.5E-05	0.4385	0.88035	0.71372	0.27535	0.30648	0.25164
Eno4	-5.33087	1.8E-16	1.30691	3.36568	3.45906	1.41491	0.98501	0.93103
Il22ra1	-5.33573	1.2E-05	0.36067	0.8327	0.80331	0.11324	0.34374	0.34496
Rilp	-5.33958	7.4E-13	1.97544	2.99649	3.35944	1.47014	1.0785	0.95171
Ccdc114	-5.34533	4.2E-06	2.15044	3.88173	3.63869	0.83648	0.92863	1.80561
Bnipl	-5.35387	0.00064	0.10084	1.05278	0.44922	0.25329	0.17941	0.15432
Lrrc36	-5.36471	0.00015	3.22603	6.93277	6.81941	1.48363	1.38575	3.16378
Catip	-5.36615	8E-07	2.93346	5.84722	4.98998	1.4144	1.38458	2.4049
1700030J22Rik	-5.37869	6E-11	0.85047	2.26623	1.90749	0.55831	0.6288	0.79865
Ulk4	-5.38046	3E-09	1.41658	3.36901	2.5297	0.92372	0.58851	1.20898
1810008I18Rik	-5.40636	0.00272	0.84849	0.68138	1.74446	0.2664	0.43129	0.64923
Plin5	-5.41503	6.7E-09	0.55496	1.87176	2.0157	0.62725	0.39492	0.76434
Ccdc60	-5.41645	0.00012	0.59104	1.81943	1.45143	0.30928	0.32546	0.69721
Myl3	-5.42498	0.01378	0.11333	0.72805	0.34949	0.1779	0	0.3035
Erich3	-5.44404	3.1E-08	2.67484	4.29606	3.29429	0.86009	1.14945	1.82916
Pcsk4	-5.44805	3.5E-05	0.39155	0.6603	0.59033	0.29504	0.13932	0.23968
Dbp	-5.4519	6.3E-18	5.06927	12.2126	10.4222	4.42238	4.05195	2.99054
Tnni3	-5.45748	0.0029	5.22113	10.5046	4.30915	1.23384	1.0844	4.55346
Ccdc153	-5.45801	1.6E-10	6.48496	18.1358	15.4769	4.21498	3.70114	6.35491
Kcnk2	-5.46415	4.6E-11	0.89038	3.63153	5.44354	1.04462	1.25055	1.21914
Cyp2s1	-5.47469	0	8.2795	20.4174	20.7032	5.86169	5.79414	6.56847
Cdhr3	-5.4775	6.8E-23	5.41248	13.0395	9.32693	4.18675	3.26952	3.39117
Wdr66	-5.49295	7.4E-16	2.59719	3.95051	3.72731	1.36224	1.25027	1.48461
Zfp92	-5.49526	0.00456	0.03435	0.09654	0.10592	0.02157	0.04365	0.02628
Syt5	-5.50082	3.7E-10	0.43797	2.11028	1.6675	0.70282	0.46998	0.48406
Fam47e	-5.50209	3.3E-08	1.26248	3.83495	4.66457	1.27529	0.94864	1.38601
Dynlrb2	-5.52601	3.1E-08	2.29139	14.2189	9.70748	4.83969	3.60005	1.59389
Wdr31	-5.53443	2.3E-09	0.56223	1.39376	0.83762	0.52189	0.34791	0.39279
Ccdc87	-5.5442	2.9E-05	0.25632	0.41167	0.47679	0.11496	0.1489	0.19613
Rnf207	-5.54456	0.00013	0.21953	0.27913	0.12537	0.05744	0.09299	0.09799
Slc9a2	-5.55001	2.9E-05	0.06633	0.17313	0.1932	0.06248	0.05901	0.05076
4930547M16Rik	-5.55262	0.0011	0.14477	0.42629	0.26458	0.0303	0.17171	0.11078
Dnah3	-5.55299	3.4E-07	5.04407	11.0661	9.04979	1.92174	2.65857	4.11973
Dnah9	-5.55804	1.6E-11	2.82486	6.93381	5.98196	1.41814	1.86407	2.28372
Fam13a	-5.56188	3.8E-17	1.44591	4.3212	5.01725	1.56302	0.98615	1.17638
Jhy	-5.56701	8.4E-05	0.17323	0.91812	0.61732	0.08702	0.24654	0.2916
Ccdc40	-5.58539	8E-13	2.17005	5.15087	4.501	1.50734	1.22994	1.61636

Fstl4	-5.60029	0.00041	0.13221	0.16988	0.19934	0.08302	0.05376	0.0607
1110017D15Rik	-5.60054	1.4E-13	1.79312	4.90897	4.58028	1.74011	1.32574	1.30966
Stpg1	-5.60664	6.1E-06	0.1517	0.54818	0.49381	0.19051	0.11566	0.23214
Gm12963	-5.60911	0.01293	1.31498	0.89353	0.55457	0.3811	0.15425	0.61918
Dnaaf3	-5.61237	6.4E-05	5.62431	7.52768	8.17285	1.27532	2.06473	3.22764
Dydc1	-5.61374	0.01368	0.2328	1.12171	1.11681	0.29237	0.355	0.2672
Gm45396	-5.61557	3.2E-08	11.5937	20.2358	14.5936	4.16003	5.95328	7.24172
March4	-5.61731	3.4E-12	1.01752	2.4105	1.74331	0.57504	0.74995	0.64232
Cfap161	-5.64649	3.7E-12	0.61761	1.80883	1.49384	0.61594	0.35086	0.55596
Wdr93	-5.64878	2.4E-10	0.86167	1.87238	1.49367	0.63655	0.43799	0.58175
Gm28729	-5.6491	8.1E-13	0.70765	2.55726	1.89945	0.66653	0.65945	0.67684
RP23-312B17.1	-5.65526	0.00787	0.11212	0.40517	0.30735	0.0704	0.08549	0.17158
Dnah6	-5.66055	7.8E-14	5.56932	15.4745	11.8098	3.34342	3.47531	4.52903
Slc28a3	-5.66271	1.3E-05	0.2205	0.22429	0.14104	0.06461	0.06725	0.10123
Dcdc2b	-5.66686	0.00013	1.01373	2.30656	1.44736	0.95483	0.64411	0.96959
Zbbx	-5.67694	3.7E-10	1.48878	2.29916	2.40683	0.71911	0.85377	0.81786
Lrguk	-5.70649	5E-17	0.97497	1.4579	1.9584	0.61221	0.68356	0.41159
Wfdc13	-5.71002	0.03105	0.5392	0.6062	0.59123	0.13543	0.27408	0.24754
Osbp2	-5.71329	0.00815	0.01821	0.17546	0.1123	0.05717	0.01851	0.0418
Lsamp	-5.72457	0.0417	0.00673	0.03242	0.02767	0.0169	0.00342	0.00515
Ttc21a	-5.72874	4.2E-09	6.38424	15.6741	13.2288	2.90203	2.90642	5.11526
Tcp11	-5.73727	7.9E-12	1.27457	2.35859	2.77236	0.69594	0.84504	0.89045
Akap14	-5.73936	1.7E-07	0.45242	3.21793	2.43611	0.77109	0.45993	0.93962
Pcp4l1	-5.74848	1.8E-16	6.5364	18.3422	11.5763	6.27189	3.91978	3.54033
Efcab6	-5.75245	1.1E-09	0.66874	1.64013	1.2882	0.46532	0.36749	0.48404
Cfap99	-5.75831	1.3E-06	0.87287	1.40192	0.93468	0.23979	0.41595	0.5844
C130021I20Rik	-5.75866	0.00467	0.16785	0.29206	0.13421	0.01757	0.11376	0.08562
Ppp2r2b	-5.76286	1.2E-06	0.05813	0.58349	0.51787	0.1825	0.16251	0.08895
Kndc1	-5.76695	6E-09	1.31945	3.0311	2.37919	0.5365	0.70366	1.05924
Drc7	-5.77134	2.4E-15	3.10092	5.54067	5.1534	1.55772	1.47769	2.04646
D130043K22Rik	-5.77148	3.1E-10	0.26342	0.8638	0.69203	0.20677	0.17853	0.30234
Sgcg	-5.77245	0.01858	0.18501	0.44573	0.29585	0.1549	0.18809	0.14157
Gm32742	-5.77896	0.00287	0.2312	0.29488	0.16777	0.03416	0.09678	0.13528
Sbspon	-5.80232	0.00256	0.33717	0.98153	1.09759	0.58223	0.25708	0.16125
E230025N22Rik	-5.80255	0.00331	0.17235	0.55362	0.23623	0.21645	0.0584	0.08792
Rgs22	-5.81847	2.5E-08	3.85732	8.15374	6.08856	1.45327	1.66975	2.98956
Ccdc170	-5.82314	7.7E-10	2.29073	6.89225	5.52597	1.30416	1.53699	2.10334
Ak9	-5.83077	1E-09	1.4627	3.75078	3.09837	0.7306	0.87023	1.22095
Amy1	-5.84028	1.7E-07	0.36971	0.75573	0.36853	0.16884	0.25626	0.20574
Trim66	-5.84307	1.7E-06	0.05719	0.11023	0.1803	0.07183	0.02907	0.03501
Mycbpap	-5.84515	7.8E-13	1.76526	4.75735	3.32173	1.08967	0.97333	1.3965
4833427G06Rik	-5.85559	3.2E-07	2.1426	5.83096	3.50775	0.82219	1.33111	1.45728
Efhb	-5.85797	3.9E-08	1.13164	1.78258	1.28261	0.32796	0.53097	0.69937
Gm45055	-5.86375	0.01401	0.8043	0.25836	0.77168	0.10101	0.40883	0.12308
Rhpn1	-5.87678	7.2E-06	0.32938	1.02498	0.62076	0.23268	0.35578	0.12602
Rp1	-5.89003	8.4E-13	0.70558	2.10782	2.06314	0.47259	0.43994	0.70545
Sspo	-5.8982	0.00083	0.54029	0.99089	0.59043	0.16963	0.22639	0.34078
Pon1	-5.90222	9.4E-09	0.67941	2.95266	4.35483	1.30496	0.62975	0.82566
Kif19a	-5.90571	8.4E-09	2.16378	3.08657	2.49748	0.84025	1.04197	1.02389
Gm15411	-5.92332	6.4E-06	0.19923	0.47998	0.59166	0.0834	0.28693	0.22867
Six4	-5.92413	1.7E-20	0.55482	1.70509	1.43317	0.60298	0.40133	0.33473
Nek5	-5.92944	6.2E-12	1.95897	4.75156	3.94536	1.30541	0.97543	1.46834
A430108G06Rik	-5.93988	2.4E-05	0.53905	1.29866	1.47769	0.29013	0.23486	0.70708
Crocc2	-5.94591	0.00083	1.99744	2.8299	2.49293	0.45887	0.53654	1.41654
Kcnmb1	-5.9468	0.01577	0.63561	1.39813	0.94695	0.46853	0.42141	0.25375
Nme9	-5.94776	7.4E-09	3.27527	7.27504	6.59053	1.61906	1.70026	2.55944

Gas2l2	-5.9555	3.4E-06	1.90813	3.813	4.22714	0.52942	1.21803	1.56189
Tcte1	-5.95568	1.8E-14	1.88385	3.26771	2.32387	1.18292	0.88096	0.83604
Bcan	-5.95643	0.00376	0.19949	0.04577	0.27343	0.12527	0.01449	0.06542
Cfap70	-5.97401	2.6E-12	0.93944	2.60812	2.17057	0.43821	0.65488	0.86259
Ccpg1os	-5.98821	3.6E-05	1.14027	4.57848	2.56762	1.32973	1.07641	1.37106
Actc1	-5.99092	0.00012	10.5225	36.7896	20.4564	10.0235	2.87443	8.87301
Ccdc89	-5.99452	0.00057	0.27668	0.39994	0.26546	0.13899	0.14064	0.04234
Ldlrad1	-5.99866	1.2E-05	5.72208	11.2325	10.3119	1.92961	2.96242	4.21617
Gm7173	-6.00755	4.4E-16	0.37669	1.32343	0.95193	0.21436	0.39491	0.30624
Fank1	-6.01642	1.2E-13	1.20907	2.79147	1.89887	0.72758	0.61458	0.8095
Katnal2	-6.02053	5.8E-08	0.31372	0.7558	0.56681	0.14327	0.18846	0.26187
1700003E16Rik	-6.05943	8.5E-07	1.30368	2.88951	1.64392	0.55666	0.74219	0.75812
Ppil6	-6.07197	5.2E-08	3.26949	6.20588	6.58612	1.43088	1.35973	2.65331
Tmem88b	-6.07504	2.4E-06	0.1001	0.69669	0.48022	0.14667	0.13569	0.17872
Dnah1	-6.07519	3.9E-09	1.51162	3.33825	2.80568	0.68364	0.64488	1.14975
Hydin	-6.07999	2.6E-21	1.88783	5.36797	4.18934	1.33058	1.19786	1.30693
Lrrc71	-6.08428	1E-06	4.1473	7.41699	5.66701	1.42193	1.6425	2.72559
Iqub	-6.12312	9.9E-11	0.91996	2.19084	1.6305	0.53783	0.48374	0.6311
Foxj1	-6.13501	7.8E-14	18.0153	53.2592	35.1928	11.3955	10.4815	12.8049
Smoc1	-6.14016	0.00045	0.2319	1.01096	0.74923	0.20803	0.25259	0.20279
Tcap	-6.14459	0.03245	4.27602	11.4789	3.51652	0.61372	0.3726	4.39364
Dlec1	-6.14511	2.8E-09	2.1448	4.22261	3.14125	1.01009	0.82645	1.31025
Wdr63	-6.15867	2.6E-15	1.85395	5.02825	3.5719	1.16415	1.20908	1.20445
Ccdc17	-6.16934	1.9E-06	13.9681	27.6144	23.8651	3.38049	4.75803	9.94569
Gm42984	-6.17749	0.00213	0.35203	0.8481	0.48251	0.29474	0.11929	0.17958
Ccdc187	-6.17905	7.4E-09	3.17038	7.49596	7.01894	1.1767	1.6894	2.5972
Ccdc78	-6.21002	1.2E-06	6.24343	11.5486	8.07672	2.11439	2.49606	3.91841
Dnaic1	-6.21057	2.9E-08	1.16569	3.41009	1.99716	0.67968	0.61368	0.89194
Efcab12	-6.21227	6.6E-08	0.91333	1.64153	1.40087	0.46427	0.3095	0.63229
Vwa3b	-6.21318	2.6E-09	0.81714	1.36422	1.13474	0.31057	0.48094	0.37844
Dzank1	-6.23679	2.2E-13	0.67288	2.10513	1.69083	0.40491	0.45604	0.53631
A830010M20Rik	-6.23992	0.0282	0.27407	0.27188	0.33146	0.23284	0.14751	0.23438
Gm2568	-6.24196	0.0241	0.39732	0.55837	0.20422	0.12474	0	0.30402
Drc1	-6.26528	1.6E-16	1.64038	6.55186	4.94047	1.32821	1.42625	1.5194
Mgat3	-6.27476	1.4E-14	3.2772	8.65289	8.35415	1.93313	2.04441	2.48859
Ttll9	-6.30247	1.1E-08	0.39812	1.30548	0.93219	0.33332	0.15177	0.40617
Slc6a17	-6.30742	9E-07	0.13516	0.29848	0.13122	0.06365	0.06298	0.06895
9030622O22Rik	-6.31025	2.2E-06	0.96401	1.93538	1.55726	0.25943	0.56001	0.68494
A930001A20Rik	-6.31611	0.00377	0.16957	0.34044	0.40674	0.10648	0.12929	0.06488
Meig1	-6.32618	0.00045	0.5255	1.6036	0.50418	0.52796	0.21369	0.16084
Lrriq1	-6.33034	9E-11	1.48192	4.02272	3.44727	0.82569	0.74266	1.22973
Gm1673	-6.34676	2.7E-05	0.51937	2.78053	2.1356	0.54354	0.44	0.92727
9330158H04Rik	-6.36403	0.00036	0.0757	0.17224	0.2248	0.13468	0.13467	0.1255
Cfap58	-6.36783	4E-13	1.00411	2.19662	1.28136	0.47832	0.52799	0.52987
Gm6225	-6.39268	0.03874	0.20439	0.32827	0.51359	0.2139	0.38093	0.31278
Gjb1	-6.3963	0.00778	0.47495	0.21795	0.37199	0.17042	0.10346	0.10383
Dner	-6.40697	0.0001	0.02421	0.25274	0.28205	0.12162	0.04922	0.01852
Lhb	-6.4187	3.2E-05	0.6833	1.92053	1.75604	0.64359	0.26049	0.65354
Nwd1	-6.42486	4E-06	1.70337	1.90247	1.14722	0.4303	0.54736	0.69662
1700013F07Rik	-6.42534	9.9E-08	0.55836	1.47328	1.14796	0.20035	0.40545	0.48827
Cfap77	-6.42712	3.1E-15	0.97105	3.36731	2.05692	0.91462	0.6282	0.60792
Hspa1b	-6.42926	0.0005	7.35491	38.8891	14.555	14.7372	2.54136	3.82558
Ankrd65	-6.43716	7.2E-09	1.57696	4.36198	3.002	0.66015	0.9797	1.27367
Tekt4	-6.45478	1.3E-12	1.89	3.58193	4.19661	0.80701	1.26812	1.15693
Ascl1	-6.45625	0.01052	0.05127	0.41172	0.21082	0.09658	0.05212	0.07846
Alox12e	-6.46818	0.03918	0.32787	0.13165	0.08426	0.05147	0.02083	0.12544

Lnx1	-6.47351	1.3E-08	0.36297	0.43723	0.55969	0.18994	0.29213	0.2083
Slco1a5	-6.47979	6.4E-07	0.15559	0.29987	0.72506	0.11724	0.15817	0.11905
Ak7	-6.48704	3E-21	3.55733	8.90092	7.36503	2.02213	2.20791	2.17755
Kif27	-6.49093	4.2E-12	1.68719	4.33568	4.19812	0.84755	0.83122	1.52931
Armc4	-6.5049	8.2E-15	0.86606	1.92598	2.123	0.39741	0.60954	0.6117
Ccdc180	-6.53629	5.5E-07	4.20206	7.9089	6.88439	1.26433	1.2682	3.0478
4430402I18Rik	-6.5481	9.5E-15	0.6838	1.40714	1.25942	0.33545	0.32586	0.32701
Ribc2	-6.56406	2.1E-05	0.42065	0.90081	0.52851	0.13207	0.21382	0.26822
Gm10044	-6.58051	3.1E-09	0.52765	1.09463	0.81362	0.2485	0.29056	0.40374
Ckm	-6.59066	0.04897	0.23856	1.11751	0.43597	0.04993	0.04042	0.33465
BC021891	-6.59243	1E-08	0.24413	0.44111	0.47403	0.14052	0.12409	0.12453
Hmcn2	-6.60724	4.1E-14	0.27382	0.81592	0.80494	0.20814	0.15017	0.23157
Crnde	-6.6723	1.1E-12	0.86132	1.56782	1.49538	0.32451	0.52538	0.43937
Ccdc151	-6.67409	3.8E-12	1.47423	2.9597	2.14692	0.57857	0.81961	0.74027
5330413P13Rik	-6.68279	8.1E-10	0.21177	0.95853	0.55413	0.14507	0.137	0.25042
Slc44a4	-6.68434	3E-19	2.27023	3.58645	3.36672	1.05163	1.21072	0.82583
Mhrt	-6.69488	0.04111	0.38161	1.22581	0.52305	0	0	0.36499
Tsnaxip1	-6.70383	1.5E-08	1.40418	3.717	3.56412	0.68579	0.74019	1.19381
Cilp2	-6.71556	0.03057	0.08054	0.17248	0.0184	0.03372	0.02729	0.02054
Hsph1	-6.72245	1.2E-09	4.90948	19.892	10.7928	7.66698	2.16331	1.87828
Dnah5	-6.72413	0	5.16481	12.776	8.9631	2.4847	2.46333	3.11845
Armc2	-6.72755	1.4E-10	0.85398	1.78564	1.42447	0.37436	0.38494	0.50549
Armc3	-6.73775	5.3E-12	0.61388	1.97191	1.48649	0.30838	0.47846	0.50103
Rnf186	-6.74333	3E-12	2.04005	4.84032	3.6223	1.28101	0.84843	1.27716
Cfap57	-6.76059	5.1E-19	1.21005	3.05401	2.15215	0.83219	0.45398	0.77157
Cyp2f2	-6.79358	3.3E-10	59.8828	279.531	287.742	95.7831	37.3869	46.9142
Dnah2	-6.80997	4E-09	1.76422	4.19434	3.80214	0.69966	0.82203	1.2611
Ccdc81	-6.8199	4.2E-11	0.95478	2.23354	1.36556	0.44313	0.52752	0.47645
Fam83e	-6.82622	2.3E-05	1.77812	2.12978	1.96212	0.39741	0.56681	0.87629
Ccdc113	-6.83358	1.9E-22	2.8775	5.99357	4.74512	1.69393	1.41693	1.30729
Atp2c2	-6.85736	1.3E-08	2.013	3.81041	2.09395	0.7223	0.65778	1.07269
Ccdc108	-6.87063	2.3E-07	7.94155	17.8236	15.0785	2.23286	2.72629	5.69112
Gm14963	-6.88154	0.00209	0.04528	0.50904	0.24823	0.02843	0.06904	0.13858
Unc79	-6.90826	6.1E-14	0.369	0.93527	0.75864	0.23895	0.15239	0.24705
Frmpd2	-6.91722	5.6E-10	1.70904	3.78507	2.41645	0.68908	0.63096	1.01863
Ttc25	-6.92918	1.3E-10	0.81019	1.7703	1.4462	0.3431	0.26816	0.59109
Gm45123	-6.93091	0.00144	0.72842	2.45683	0.69888	0.36592	0.37026	0.44589
Ccdc146	-6.94561	4.2E-12	1.8548	4.08549	3.66812	0.96502	0.9428	1.21648
Gm12695	-6.94728	1.5E-05	2.18986	3.51713	2.41787	0.84032	0.68024	1.02398
Daw1	-6.9724	2.9E-06	0.70357	1.47771	1.03852	0.20391	0.24759	0.53835
Dnaic2	-6.9795	1.3E-13	1.36914	3.82785	2.5195	0.74828	0.67016	0.87301
Rbbp8nl	-6.99049	0.02241	0.14983	0.12032	0.06845	0	0.07616	0
Shisa9	-7.03794	0.03124	0.04543	0.05472	0.1401	0	0.02309	0.05214
9130230L23Rik	-7.04427	8.8E-11	0.4025	1.55149	1.40678	0.22747	0.29666	0.43117
Abcc8	-7.05188	0.00251	0.10021	0.20694	0.04906	0	0.05094	0.05477
Cfap206	-7.05636	2.3E-24	2.56443	5.69135	4.69719	1.75667	1.30351	0.99894
Mylk4	-7.05715	0.03432	0.0756	0.60711	0.15543	0.0356	0.02882	0.13015
Lrrc74b	-7.08388	7E-16	1.44003	3.28044	2.85992	0.66433	0.61247	0.96693
Timp4	-7.11864	0.00232	0.1572	0.59965	0.404	0.17275	0.44948	0.19546
Vtcn1	-7.11915	1.8E-08	1.28099	2.41212	1.05952	0.55474	0.4266	0.50698
Slc4a5	-7.12699	2.1E-17	1.07757	3.65368	4.74542	1.02749	0.57816	1.03828
Tex26	-7.15435	8.4E-05	0.28043	0.99089	0.80718	0.21131	0.11404	0.30041
Tekt1	-7.17868	4.5E-22	1.92794	4.58736	3.76801	1.30028	0.94368	0.92882
Bves	-7.19417	0.0024	0.10049	0.14123	0.24105	0.04733	0.02554	0.07689
Ccdc142	-7.20255	0.00428	0.7123	0.38134	0.74849	0.44727	0.43447	0.18167
Nek10	-7.20922	3.9E-10	0.76983	1.18762	1.15234	0.25442	0.32952	0.35653

Ppp1r32	-7.21154	2.7E-08	0.79437	2.29651	1.30655	0.44893	0.44416	0.42548
Pklr	-7.21156	0.00419	0.25054	0.20119	0.09811	0.04495	0.09096	0.05477
Fam161a	-7.24035	4.6E-11	4.00467	11.1289	8.75703	1.63783	2.06237	2.74167
Ankef1	-7.26285	7.8E-08	0.17732	0.41423	0.38665	0.08098	0.06555	0.14801
Wdr49	-7.26879	4E-10	0.77731	1.19783	0.74867	0.26384	0.34172	0.24112
Slc7a10	-7.27282	2.5E-10	0.49378	1.14554	1.09039	0.55122	0.11155	0.29386
Gm37381	-7.27392	0.00209	0.42456	0.68188	0.17457	0.05332	0.17264	0.19491
Adra2a	-7.27428	0.00047	1.43212	0.70962	0.60558	0.4018	0.41819	0.20984
Rac3	-7.29755	5.4E-17	1.52276	4.38788	4.54261	1.40615	0.68296	1.09662
Ooep	-7.29832	0.00014	0.16252	0.30452	0.63112	0.06803	0.13768	0.12435
Vwc2	-7.30917	4.4E-05	0.05752	0.13857	0.15767	0.03612	0.03898	0.02934
Gm29538	-7.3168	0.00021	0.2459	2.96206	2.02224	0.61763	0.24998	0.75262
Ect2l	-7.3361	2.6E-14	0.76179	1.44199	1.32381	0.35876	0.30424	0.39553
Crip3	-7.33807	0.00716	0.0762	0.30595	0.41775	0	0.11619	0.11661
Gm9800	-7.35089	0.01538	36.3677	42.9427	20.1472	4.77415	0.38646	0
Maats1	-7.36401	4.4E-22	1.27723	2.50266	2.22328	0.64161	0.62325	0.56683
Lgr6	-7.38147	3.3E-11	1.42006	2.17215	1.29759	0.40339	0.45543	0.59503
Fhad1	-7.40847	7.9E-09	5.41282	10.7988	9.15096	1.98958	1.6489	3.2614
Slc38a11	-7.41674	4.1E-05	0.21453	0.27995	0.12864	0.03368	0.08178	0.06156
Lrrc26	-7.41706	1.4E-08	1.26525	4.92399	4.8691	1.2834	0.93996	0.89365
A830018L16Rik	-7.41797	3E-14	0.13874	0.4659	0.37167	0.09504	0.08975	0.10616
Cyp2b19	-7.43523	0.00018	0.05556	0.31233	0.22847	0.03489	0.04236	0.10629
Colq	-7.43861	3E-10	0.36546	0.46468	1.14791	0.26773	0.20124	0.11651
Cdh26	-7.45885	0.03378	0.42298	0.92638	0.02635	0.09658	0.19546	0.11769
Scin	-7.54272	0.01965	1.0041	0.31013	0.29113	0.16975	0.1963	0.20685
Gm11992	-7.56708	6.9E-10	0.43432	1.12385	0.8268	0.36363	0.17171	0.22155
Aldh3a1	-7.58659	8.4E-11	1.6699	3.92724	2.41143	0.97368	0.75787	0.59324
Calml3	-7.59471	1.5E-05	2.76147	3.78296	1.50285	1.02	0.49541	0.93219
Trp73	-7.61653	7E-23	0.98365	1.88236	1.73548	0.59138	0.41489	0.3523
Cnksr2	-7.62335	2E-15	0.12498	0.48175	0.48821	0.10202	0.11435	0.09563
0610043K17Rik	-7.66172	0.04365	0.30083	0.36237	0.41233	0	0.15291	0.11509
Gfy	-7.70118	0.01124	0.06264	0.35211	0.30049	0.03933	0.03184	0.14379
Aox3	-7.71228	5E-24	2.66781	7.15451	9.42363	2.22325	1.1719	1.68849
Cfap74	-7.73369	1.7E-09	1.51306	3.71997	3.14269	0.59198	0.6981	0.89057
Mdh1b	-7.76682	5.9E-10	0.40426	1.94782	1.47757	0.42307	0.17124	0.51554
Gm16194	-7.78309	8.7E-08	1.10605	2.28398	1.84085	0.49609	0.40158	0.60451
Bmpr1b	-7.81811	2.6E-20	0.4468	1.73163	1.55764	0.35375	0.3456	0.31215
Ttll6	-7.82229	1E-08	2.31218	3.13485	2.737	0.52796	0.61054	1.0799
Dnah10	-7.82361	2.3E-08	4.3416	9.69057	7.16459	1.23878	1.35046	2.59482
D830044I16Rik	-7.85036	0.00019	0.89401	1.36408	0.88839	0.16841	0.15905	0.51305
1700007G11Rik	-7.8561	2.4E-08	2.31958	2.69776	2.68596	0.85383	0.60985	0.85683
Kcnma1	-7.86927	3.3E-11	0.25015	0.54908	0.29715	0.10472	0.1102	0.08932
Cfap100	-7.9001	5.5E-09	3.8775	7.15277	5.37241	1.05861	1.11401	2.02094
Noxa1	-7.92151	0.0063	0.07163	0.2876	0.63813	0.04498	0.10922	0.10961
Gm31105	-7.95408	0.00482	0.50576	0.54153	0.69321	0.31758	0.08569	0
Atp2b2	-7.96246	7.3E-16	0.37651	0.65634	0.98807	0.14416	0.1447	0.24593
Sec14l5	-7.99518	0.00114	0.8425	0.79597	0.88305	0.12448	0.10076	0.45505
Ccdc24	-8.00209	3.1E-10	0.9542	2.56804	1.87342	0.92304	0.61611	0.65119
Ccdc30	-8.03181	2.3E-10	1.75298	3.91349	3.44786	0.56138	0.60591	1.14012
Acp7	-8.03311	0.00779	0.31134	0.58339	0.10668	0.03258	0.13188	0.07941
Ckmt2	-8.04161	0.00286	0.7794	1.75252	0.53414	0.09788	0.27732	0.41746
Col19a1	-8.05422	0.00066	0.05045	0.09453	0.0922	0.03168	0.00855	0.02574
Iqca	-8.08127	1.8E-19	0.8122	2.52197	2.245	0.578	0.31651	0.58003
Gm12576	-8.09242	0.00716	0.11445	0.24508	0.02614	0.02395	0.03878	0.02919
Tas1r1	-8.10889	2E-07	0.32363	0.61724	0.91488	0.10161	0.20563	0.18572
Slc16a11	-8.10911	1.2E-10	6.07665	12.928	11.4988	1.70853	1.6366	3.88626

Lrrc46	-8.11309	6.3E-09	0.69353	1.74043	1.60409	0.54436	0.1322	0.46433
Gm29266	-8.11883	0.00038	1.73729	2.01519	1.98432	0.36363	0.19624	1.03391
Defb23	-8.11907	0.03856	0.37122	0.59621	0.8904	0	0.09435	0.42606
Cfap46	-8.13007	7E-10	3.60365	7.82466	7.22824	1.04863	1.34669	2.06564
Dcdc5	-8.13699	3.2E-10	1.06412	2.40412	1.7794	0.32964	0.41829	0.56453
Ccdc33	-8.20266	4.4E-07	2.55834	2.82055	1.79785	0.42204	0.57306	0.7797
Fmo3	-8.20974	1.3E-12	1.58393	5.97445	8.81556	2.10975	0.63434	1.28542
Ces1b	-8.21248	0.0334	0	0.09471	0.32331	0.07406	0.02998	0
Plch2	-8.25145	3.5E-06	0.71388	0.92871	0.95889	0.08069	0.14515	0.40421
T2	-8.25513	4E-06	2.35564	2.36463	2.73545	0.32871	0.63195	0.90123
Ttc16	-8.2595	6.5E-12	1.34167	3.26142	2.36081	0.43262	0.47923	0.77689
Cbr2	-8.36553	0	192.625	478.57	597.241	157.29	61.4888	78.5364
Fn3k	-8.3914	1.5E-07	0.21063	0.23257	0.30674	0.0496	0.06691	0.08058
Ccdc155	-8.41143	4.8E-06	0.68561	1.96636	1.07397	0.30751	0.24893	0.37472
Rab39b	-8.44769	0.00934	0.06811	0.21878	0.11669	0.14969	0.05193	0
Kcnip4	-8.48485	0.01049	0.06387	0.20515	0.28449	0.24062	0.48694	0.04887
Gm43634	-8.48516	0.02061	4.97594	2.75581	4.11563	0	1.39547	1.3129
Lrrc23	-8.48595	4.9E-08	8.88348	26.5509	17.852	2.4326	2.68214	6.38845
Ky	-8.50915	1.4E-07	0.70922	1.53459	0.85057	0.16082	0.21029	0.37685
Dnaaf1	-8.54096	3.7E-16	0.98535	3.5278	2.3916	0.54139	0.54259	0.65971
Gm12474	-8.58365	1E-05	0.65713	1.12138	0.84439	0.25789	0.16701	0.25141
Ces1e	-8.59765	4.4E-11	0.5291	1.52961	1.48666	0.33223	0.21515	0.36436
Bbox1	-8.63934	8.1E-07	0.15783	0.29573	0.1983	0.01652	0.08022	0.06038
Pm20d2	-8.64263	2.7E-08	0.56836	0.7607	0.95213	0.15862	0.2247	0.19328
Dpp6	-8.65822	2.2E-08	0.11547	0.38019	0.12662	0.07976	0.04109	0.03534
Ano7	-8.69156	0.00718	0.34885	0.23346	0.03985	0.07302	0.05911	0.04449
Gm6970	-8.69185	0.00078	0.1739	1.53618	0.95343	0.2184	0.17679	0.26613
Wdr72	-8.69674	2.1E-10	0.51056	0.77031	0.39231	0.10686	0.13369	0.18941
Lypd6b	-8.70309	1.1E-13	0.80059	1.46442	1.1278	0.33514	0.20347	0.34032
Gm21887	-8.7124	0.03794	90.3359	22.425	49.8826	20.3135	24.9757	9.34077
Ttc34	-8.75673	6.9E-10	0.72548	2.21762	1.60382	0.35268	0.45203	0.48348
K230010J24Rik	-8.76673	0.00109	0.37482	0.86	1.02748	0.06725	0.08165	0.36874
Bbof1	-8.79228	5.5E-13	2.79957	6.36988	5.25837	1.1394	1.13315	1.46775
Rassf10	-8.798	8.6E-16	0.96074	1.91551	1.24872	0.24963	0.37047	0.40559
D630024D03Rik	-8.85112	1.6E-08	0.44347	1.36516	1.01306	0.04641	0.26299	0.39588
Ccdc13	-8.90857	1.2E-07	1.21986	2.77556	2.02032	0.21277	0.4306	0.7519
Cfap73	-8.95757	2.1E-05	2.38932	4.94834	3.01634	0.31586	0.70313	1.34711
Crb1	-8.97286	0.00038	0.01059	0.04254	0.07986	0.00665	0.02154	0
C530043A13Rik	-8.99206	0.00757	1.54505	1.14531	0.9774	0.11194	0.18124	0.59111
Odf3b	-9.08824	7.2E-08	11.2587	19.4253	15.1259	2.86985	2.26646	5.11765
1810020O05Rik	-9.11328	2.7E-06	0.77434	2.48734	1.59201	0.27785	0.05623	0.84642
Cfap157	-9.15293	3.2E-08	1.95311	5.43727	4.0155	0.57233	0.46329	1.44463
Dytn	-9.37752	0.00015	0.11312	0.18169	0.07753	0	0.02875	0.05771
Gm5737	-9.39762	2.7E-05	0.36421	0.7312	0.624	0.17152	0.13885	0.06967
Ttll13	-9.39925	3.7E-08	0.16625	0.51177	0.30382	0.0522	0.05634	0.12721
Fam92b	-9.48765	1.2E-07	1.32839	2.73359	2.84491	0.41707	0.37982	0.63527
Xlr3a	-9.49045	0.01645	0.14316	0.13796	0.27471	0.07192	0.05822	0.13145
Mypn	-9.50398	0.00417	0.33116	0.62053	0.27234	0.08318	0.03367	0.15204
Alms1-ps1	-9.57794	0.0257	0	0.17176	0.62296	0.03358	0.1359	0
Proc	-9.60828	0.02301	0.14792	0.17818	0.15206	0	0.03759	0.05659
Rhbg	-9.66743	0.00013	0.41855	0.33612	0.61466	0.11264	0.12157	0
Foxn4	-9.66876	0.00041	0.13743	0.16554	0.28255	0.02157	0.03493	0.07887
Nell1	-9.67264	0.00168	0.06372	0.05117	0.03275	0.01	0.01619	0
Cadm2	-9.72721	4.3E-06	0.02361	0.17068	0.10519	0.0519	0.03601	0.0271
Cyp2e1	-9.79221	0.00017	0.16101	0.77579	0.99308	0.2022	0.16368	0.04107
Itpka	-9.80342	1.1E-21	1.14672	3.47887	2.61957	0.52004	0.51812	0.58495

Bpifa1	-9.82778	0.00548	15.4426	70.3133	11.0454	6.96665	4.80116	7.39939
Dyx1c1	-9.92827	0.00067	0.29321	1.50698	1.16548	0.22094	0.23847	0.17948
Ptpn20	-9.92875	0.02196	0.14993	0.2709	0.07706	0	0	0.11472
Acsm1	-9.99151	4.3E-09	1.4338	2.0896	2.21997	0.40014	0.10797	0.69076
Sult1d1	-9.99863	1.1E-11	1.4601	3.93367	2.35633	0.53236	0.45488	0.64871
Gm20662	-10.0483	0.0006	1.93761	2.45948	1.54205	0.62797	0.82604	0.86086
Mst1	-10.0747	1.4E-08	1.53606	1.27465	0.52635	0.38582	0.23424	0.27425
Erich5	-10.1632	1.4E-09	0.5362	1.09606	0.76834	0.24487	0.09911	0.18649
Slc27a2	-10.1707	1.7E-09	0.27767	0.66895	0.57088	0.13561	0.07841	0.11803
D430001F17Rik	-10.2004	2.2E-05	0.81036	0.65076	0.46992	0.03914	0.19011	0.19079
Gm15997	-10.2261	5.4E-05	0.46607	0.43666	0.26617	0.09755	0.11845	0
Six1	-10.2308	1E-09	0.42279	1.42176	1.34009	0.18252	0.10745	0.34373
Lrrc43	-10.2575	3E-06	3.53176	6.07752	6.64643	0.63363	0.76937	1.6729
Plk5	-10.2664	1.6E-08	0.21624	1.14611	0.59278	0.1901	0.08793	0.13237
Vpreb3	-10.292	4.9E-18	2.42694	9.74479	5.08209	1.52395	1.23363	0.82534
Slc35d3	-10.3319	0.0004	0.26423	0.45974	0.09054	0.08296	0.02238	0.06739
4932443I19Rik	-10.4839	2.2E-10	0.72402	1.20161	0.7112	0.13639	0.15948	0.25853
Tmem232	-10.531	4E-13	1.94982	3.40997	2.13799	0.43532	0.35239	0.76254
Wdr27	-10.6404	3.1E-08	0.40496	1.33695	1.3568	0.08476	0.11436	0.41315
Galnt5	-10.6459	6.6E-10	0.1817	0.41689	0.33798	0.0489	0.07916	0.05958
Tcerg1l	-10.7986	0.01486	0.06007	0.1206	0.04117	0	0.01527	0.02298
Iqch	-11.1961	9.3E-08	0.08376	0.37666	0.30996	0.05259	0.04257	0.08972
Gm20422	-11.2476	0.01657	1.36459	0.54792	0.46759	0.10711	0.43351	0.13052
Flg	-11.4757	0.00361	0.24766	0.21813	0.27923	0.0301	0.02843	0.07947
Lmx1b	-11.5006	0.00019	0.11867	0.15247	0.06506	0.02981	0.01206	0.01816
Wdr95	-11.5559	6E-06	1.83206	1.89923	1.18706	0.14641	0.18625	0.58622
Slc13a2	-11.6742	0.04631	0.48018	0.30849	0	0.09046	0.09763	0.03674
Atp6v1b1	-11.7031	1.6E-12	0.42299	0.97658	0.81529	0.1162	0.08063	0.22251
Ces1f	-11.7861	1.6E-09	0.38783	0.56626	0.55573	0.13283	0.07169	0.08093
Adh7	-11.8339	6E-09	1.00925	2.13672	1.55099	0.30727	0.18655	0.39782
Slc5a9	-11.8863	0.00094	0.2681	0.2153	0.09187	0.02806	0.03407	0.05129
Dcdc2a	-11.8873	1.6E-20	0.30758	0.98802	0.74736	0.15802	0.07817	0.13907
Cfap221	-12.1326	1.4E-17	0.9933	1.4039	1.36146	0.24949	0.18176	0.27361
Cryaa	-12.1827	1.3E-06	0.39062	1.09791	0.60232	0.12264	0.14892	0.07472
Dmrt2	-12.2024	2E-07	0.49348	0.59443	0.30437	0.03099	0.10033	0.11328
Lypd6	-12.2313	0.00033	0.09318	0.24942	0.12771	0.0195	0.01579	0.04753
AF529169	-12.2514	0.03425	0.01594	0.03839	0.02184	0	0.0081	0
Capn13	-12.2944	2.7E-05	0.22154	0.42697	0.33401	0.05564	0.02252	0.10171
Mpz	-12.3604	0.00322	0.49378	0.39653	0.3008	0.0689	0.02789	0.12594
Klkb1_2	-12.4091	0.04163	0	0.37163	0.49013	0	0	0.3541
Cyp4f15	-12.4532	7.5E-19	0.60904	1.9156	1.80867	0.35056	0.12899	0.31068
Tymp	-12.5198	1.3E-07	5.17405	10.4104	8.80623	0.89257	0.89593	2.30579
Adprhl1	-12.5239	0.00382	0.09889	0.30177	0.05422	0.02484	0.02011	0.03027
Gm7269	-12.7271	0.00063	0.28341	0.60691	0.32371	0	0.09604	0.07228
Gp2	-12.7472	6.3E-08	1.85496	3.67752	1.54932	0.47319	0.47144	0.31048
Styxl1	-12.7542	8E-06	0.26174	0.57802	0.40359	0.16435	0	0.05007
Fut9	-12.828	1.1E-05	0.18432	0.19511	0.04593	0.03157	0.02981	0.01923
Lgals12	-12.9087	4.4E-11	0.5012	0.7379	0.62972	0.07868	0.06369	0.19175
Mcidas	-12.9266	0.00011	0.34441	0.19755	0.23603	0	0.05002	0.07529
RP24-170O17.2	-12.9763	0.00197	0.05762	0.23136	0.39489	0	0.05858	0.04409
Atp10b	-13.0469	3.2E-09	0.38937	0.56851	0.29918	0.08891	0.09596	0.14445
Serpina9	-13.1269	5.5E-08	0.63048	1.13919	0.45368	0.07918	0.09614	0.14473
1700019D03Rik	-13.1703	0.00288	0.0535	0.15036	0.09165	0.11757	0.10877	0.08187
Kcnk12	-13.2298	6.8E-15	1.91437	3.55508	2.7059	0.71374	0.3649	0.32043
Ccdc129	-13.2603	5.1E-16	0.90358	2.07319	1.23847	0.18237	0.21324	0.29631
4930444P10Rik	-13.2879	8.1E-07	1.01054	0.99185	0.50017	0.07051	0.22829	0.12887

Iqcd	-13.3868	5.8E-06	1.7235	3.10029	2.92924	0.43289	0.49059	0.633
Olig3	-13.9865	2.6E-07	0.39128	0.58355	0.42138	0.0351	0.05683	0.12831
Tacr1	-14.0202	4.2E-06	0.71372	0.48718	0.35462	0.07843	0.05442	0.13653
Pax9	-14.1928	3.7E-06	0.45815	0.49668	0.14129	0.07192	0.02329	0.08764
Gm12996	-14.2076	0.02091	0.18211	0.43872	0.3744	0	0.09256	0
Slc5a8	-14.4349	0.00224	0.34826	0.80406	0.13425	0.01367	0.08851	0.08327
Fer1l4	-14.4614	2.8E-11	0.45347	0.68785	0.37983	0.09492	0.11952	0.0257
Klhl34	-14.487	1E-05	0.05985	0.38453	0.57427	0.07517	0.03042	0.0458
Acnat1	-14.4996	3.7E-05	0.32656	0.56819	0.2238	0.03418	0.05533	0.08329
Dthd1	-14.5795	0.00027	0.62037	0.53651	0.13082	0.0899	0.04851	0.07303
Bcl2l10	-14.6227	0.01389	0.2848	0.07624	0.19518	0	0.04826	0
Ccdc110	-15.0115	0.00028	0.17443	0.28015	0.17931	0	0	0.1001
Fmo6	-15.1841	0.00156	0.05918	0.28516	0.24335	0	0.03008	0.04528
Olfr315	-15.4018	0.00206	0.13988	0.22466	0.12781	0	0	0.07135
Lars2	-15.6447	0.00754	90.2526	43.1894	40.5585	7.97127	6.56549	9.92849
Krt79	-15.9843	4E-12	0.77323	0.57659	0.83272	0.27745	0.02807	0
Prickle4	-16.2306	0.0105	0.35419	0.71108	0.44501	0.03707	0.30006	0.13551
BC048546	-16.2353	2.5E-09	6.61642	5.9967	1.21013	0.73474	0.72454	0.73254
Hhatl	-16.3796	1.4E-13	0.44122	1.16925	0.93736	0.08312	0.06728	0.20256
Ppp1r1b	-16.5959	1.9E-08	0.94934	1.01649	0.67771	0.19871	0.18096	0.06053
Dio1	-16.9944	1.6E-05	0.47033	0.33049	0.12087	0.03692	0.05977	0
Slc38a3	-17.1084	0.02072	0.08779	0.05288	0	0.01378	0	0
Aspg	-17.4173	2.6E-06	0.08479	0.68088	1.01686	0.10648	0.06465	0.03244
Foxa3	-17.4888	7.7E-05	0.45486	0.41094	0.23379	0.07141	0.0578	0
D930020B18Rik	-17.5082	5.6E-12	0.40107	0.60837	1.03835	0.08395	0.02265	0.17049
Reg3g	-17.5717	2.6E-06	18.9562	14.3429	3.90482	1.68571	1.64305	2.09604
Oit1	-17.822	2.3E-06	1.38872	1.33825	0.44413	0	0.18824	0.21252
Cyp2a5	-17.8414	2.2E-07	12.4591	24.3177	5.70505	1.8243	1.47675	1.75275
Kcna1	-17.8576	4.8E-06	0.14538	0.11675	0.14945	0	0.01232	0.03708
Uox	-17.8894	0.00039	0.07209	0.28948	0.07411	0.04527	0	0
Pigr	-18.5531	3.4E-20	16.4017	15.5284	13.1532	2.22244	1.56503	1.98158
Chst13	-18.8524	0.02503	0.2102	0.28133	0.04802	0	0	0.05361
Ccr3	-19.8882	0.0002	0.17699	0.2274	0.07277	0.02223	0	0.02708
Gcnt3	-20.1076	0.01728	0.21543	0.06487	0	0.01691	0.01369	0
Pck1	-20.9208	0.00032	0.33776	0.46111	0.34721	0.02121	0.06867	0.02584
Alox15	-21.6143	3.4E-05	1.32149	0.79592	0.32344	0	0.09596	0.18056
Gm45218	-21.6444	0.00307	0	0.27714	0.28381	0	0.03508	0
4930404N11Rik	-22.0444	0.00084	0.5953	1.64664	1.3599	0.62301	1.47935	0.91101
Otx1	-22.2726	1.6E-07	0.3845	0.70331	0.19031	0	0.04343	0.08172
Cfd	-22.376	4.7E-05	2.0077	4.43378	5.33166	0.15759	0.19135	0.6721
Susd5	-22.4624	0.00078	0.02837	0.27335	0.13608	0.03562	0	0
Spdef	-22.9266	3.7E-08	1.37628	1.06271	0.6167	0.19943	0.05381	0.08101
Scgb3a1	-23.0911	5E-13	103.036	269.694	97.2916	18.1207	12.6997	13.1893
E030044B06Rik	-24.7013	7.2E-06	0.27445	1.02853	0.25078	0.05745	0	0.07
Cyp4a12b	-25.3216	2.4E-06	0.67702	1.1262	0.36455	0.06073	0	0.074
Lypd2	-26.6432	3.3E-30	11.2578	18.2182	16.8331	1.49951	1.12714	1.30516
Chad	-27.5869	4.5E-20	6.96294	13.5084	6.23645	0.69263	0.98119	0.94951
Tbata	-29.1358	3.6E-06	0.57289	1.45685	0.29446	0	0.02427	0.14612
Sost	-31.1843	0.01353	0.22424	0.31513	0.07684	0	0.0285	0
Pla2g2c	-31.5993	2.6E-05	0.19239	0.12875	0.13185	0	0.0163	0
Asgr1	-31.6556	2E-05	0.0728	0.40921	0.39911	0	0.037	0
Bpifb1	-31.9186	3.8E-10	21.2298	48.4972	15.9755	2.71442	1.33466	1.86209
Cdc20b	-34.9678	0.00022	0.69694	0.20352	0.52105	0	0	0.09696
Sox21	-35.9768	3.1E-08	0.76217	1.32205	0.33429	0.07657	0.0155	0.11664
Itln1	-36.3561	0.02953	1.01507	0.16303	0	0	0	0.07767
Car3	-37.283	0.00014	0.80851	1.36348	1.44063	0.07615	0	0.12373

Gmnc	-38.6286	2E-10	0.14183	0.22779	0.34992	0	0.01442	0.0217
BC027072	-39.0055	0.03432	0.02315	0.05576	0.03172	0	0	0
Muc5b	-39.9879	0	35.1931	37.8648	29.9319	2.4835	1.74285	1.90104
Plin1	-40.3585	0.00038	0.1993	0.22007	0.17073	0	0.02533	0
Gm45572	-41.7454	0.0331	0.46328	0.37203	0	0	0	0
Gm15319	-44.5685	0.0364	0	0.47943	0.40914	0.08651	0.11672	0.03514
Gm8941	-45.2218	0.02075	0.12883	0.31037	0.26487	0	0	0
Ern2	-46.4753	3.1E-06	1.05503	0.56483	0.29457	0.02454	0.01986	0.0598
Gm14280	-52.845	0.00488	0.24062	0.12882	0.1649	0	0	0
Muc5ac	-54.4799	1.1E-07	6.92631	1.90308	1.70528	0.15557	0.16426	0.22254
Gm44963	-57.0071	0.01082	0.15546	0.37453	0.5327	0	0	0
Gm15751	-61.1736	1.6E-07	0.49761	1.19882	0.80993	0.78116	0.85367	0.23797
Fer1l6	-65.6376	1.6E-05	4.38655	1.3282	0.94621	0.07225	0.0731	0.13206
Pdzrn4	-66.8454	0.00127	0.11086	0.06677	0.05698	0	0	0
Tff2	-77.4136	1.5E-13	14.4774	17.9965	9.78571	0.37359	0.30242	0.60699
Chil4	-79.4234	0.00681	4.66588	1.38999	0	0	0.15301	0.05758
Cyp2g1	-81.1367	0.00033	0.05399	0.08672	0.37004	0	0	0
Fcgbp	-93.445	1.6E-08	15.0718	6.91119	2.17015	0.23556	0.18508	0.28704
Capn9	-98.1402	0.00184	1.1035	0.03853	0.03288	0.03013	0	0
Clca1	-308.366	1.9E-07	86.0748	31.3476	15.3618	0.40436	0.48319	0.37541
