# Supplementary material for: Transcriptomic Evaluation of Pulmonary Fibrosis-Related Genes: Utilization of Transgenic Mice with Modifying p38 Signal in the Lungs
Source: Int J Mol Sci. 2020 Sep 14;21(18):6746. doi: 10.3390/ijms21186746 (PMC7555042; doi:10.3390/ijms21186746)
Supplement: Supplementary file 1 [file ijms-21-06746-s001.zip › Supplementary Table S4.pdf]

Supplemantary Table S4. DEGs altered by BLM in p38-DN group.

Name (symbol)	Fold change	FDR p-value correction	DN_PBS_Rep1 - RPKM	DN_PBS_Rep2 - RPKM	DN_PBS_Rep3 - RPKM	DN_BLM_Rep1 - RPKM	DN_BLM_Rep2 - RPKM	DN_BLM_Rep3 - RPKM
Srp54a	176.258	0.00318	0	0	0	5.80514	0	1.2457
Bcl2a1b	114.443	1.5E-06	0	0.64171	0.30174	5.52783	4.05585	1.87683
Ighv5-4	107.489	0.00405	0	0	0	1.86429	5.39777	1.11014
Gm10718	87.8238	0.00179	0.12806	0	0.12067	4.30484	4.36677	3.81049
Gm10719	87.0811	0.00084	0	0	0.04565	2.465	1.36888	3.47303
Chst5	77.4373	5.9E-05	0	0	0	0.47205	0.42833	0.21623
Chodl	76.9338	5.2E-05	0	0	0	0.29038	0.11121	0.24702
Igkv6-32	71.3888	0.01055	0	0	0	2.3813	3.71431	0.32227
Ighv5-9	66.473	0.01317	0	0	0	1.89551	1.42286	1.69309
Krt5	64.4926	0.01866	0	0	0	0	0.11639	0.75403
Nog	62.1541	0.00126	0	0	0	0.51419	0.30076	0.34794
Gm10721	59.387	0.00081	0.52142	0.11195	0	4.85579	1.90504	5.11301
Ccl3	53.2722	0.00676	0	0	0	1.34467	0.66552	0.15398
Lrrc15	50.542	0.00856	0	0	0	0.04476	0.19201	0.06664
Prss22	45.875	0.00443	0	0	0	0.17967	0.51377	0.44577
Igkv2-137	44.4187	0.02062	0	0	0	0.94889	3.73099	0.47087
Igkv8-24	43.8552	0.0485	0	0	0	1.30245	1.86224	0.64631
Saa3	43.1204	1.2E-07	0	0.08622	0.22073	11.0366	4.62971	1.26733
Gm10801	41.3367	2.2E-08	0.51909	0.29721	0.16304	14.1486	9.60913	18.2545
Sectm1a	40.5833	0.01446	0	0	0	0.18731	0.25108	0.34856
Wnt10a	40.2727	0.0116	0	0	0	0.12842	0.17213	0.23896
C430002N11Rik	40.1223	0.02727	0	0	0	0.03753	0.40249	0.16762
Ighv1-53	39.8135	0.04913	0	0	0.23411	2.48306	2.42065	0
Gm10800	38.3819	9.8E-07	3.03905	1.92916	1.74307	93.7582	53.6824	97.9358
Adamts4	36.4213	9.5E-15	0.01813	0.01557	0.05125	0.97187	0.81255	0.58853
Tm4sf5	34.4253	0.03625	0	0	0	0.21129	0.45315	0.31454
Mmp12	34.0246	1.4E-10	0.01205	0.0414	0.02271	0.96344	0.85705	0.63564
Frzb	31.9678	1.9E-07	0	0.02314	0	0.39163	0.52496	0.36439
Thbs4	31.3749	2E-09	0.02651	0	0.04997	1.15632	0.90416	1.00415
Gm21738	27.8503	1.5E-08	0.25612	0	0.362	11.2857	5.11536	13.1635
Cemip	27.2849	1.3E-13	0.02139	0.01837	0.01344	0.58297	0.56264	0.64607
Ighg1	24.97	0.00264	0	0	0.06341	0.79477	0.91783	0.45506
A2m	24.8999	0.00029	0	0.01521	0	0.22523	0.25878	0.16765
Chl1	24.8557	6.8E-35	0.02838	0.03046	0.03342	1.08926	0.98837	0.75801
Lcn2	21.8718	9.2E-12	0.6317	0.51397	0.90851	28.9079	20.9578	6.74525
Gm26870	21.3429	1.2E-10	0.29156	0.36169	0.79365	12.5971	9.24767	13.8896
Sprr1a	21.2436	0.00124	0	0.09292	0	1.57285	1.3704	0.43903
Spp1	21.2347	9.5E-24	2.59294	1.63609	2.89202	73.6553	79.2421	35.0706
Slc26a4	21.1033	4.7E-05	0.25524	0.02436	0.05345	3.29812	4.28278	1.57269
Atg9b	18.7484	2.7E-13	0.0221	0.01898	0.0833	0.8634	1.05506	1.01631
Igkv10-96	18.7259	0.00311	0.48584	0	0.22889	6.40038	11.1235	2.2999
H2-Eb2	17.1445	1.4E-06	0	0.03391	0.0744	0.53802	1.00004	0.96113
Megf10	17.0724	9.2E-09	0.0226	0	0.01065	0.26691	0.2532	0.19868
Ighg3	17.0438	0.04198	0	0.06922	0	0.29291	0.94231	0.76309
Ighv1-72	17.0139	0.0292	0.88088	0	0	4.20171	7.50952	1.78715
Clec4n	16.531	9.2E-12	0.12283	0.10549	0.40508	5.97032	4.78676	2.24275
Stra6l	15.5646	2.6E-06	0	0.00783	0.02577	0.25674	0.31972	0.11096
Gpnmb	15.3938	9.9E-17	0.53832	0.16952	0.52415	7.90693	12.3776	3.49487
A430093F15Rik	15.2094	0.00398	0.02796	0	0	0.25403	0.21793	0.15127
Ighv1-19	14.3855	0.03948	0	0	0.40579	2.73889	1.46853	0.29124

Ighv1-64	14.3269	0.00355	0	0.18493	0.2029	2.15199	4.61538	1.4562
Svop	14.1302	0.00362	0	0.01644	0	0.13913	0.11189	0.15534
Ighv3-6	14.1053	0.00145	0.49833	0.21399	0	3.62205	8.25373	2.02203
Eya4	14.0485	0.00347	0	0.01787	0	0.15121	0.14188	0.14069
Clec7a	13.8319	1.2E-18	0.19835	0.3407	0.6853	8.62004	8.79281	3.44287
Lama1	13.7314	8.6E-20	0.10995	0.05508	0.1554	1.73149	1.61574	1.8217
Ighv8-8	13.4211	0.00814	0.2436	0.41842	0	1.77055	8.06929	2.6358
Igkv1-110	13.0121	0.01292	0.43495	0.56032	0.61476	4.74208	16.7387	2.05901
Acod1	13.0067	0.00641	0.03325	0.02855	0	0.69476	0.35631	0.08994
Timp1	12.8862	1.1E-06	0.58924	0.75909	1.11045	13.4731	9.56812	6.90709
Ighv10-3	12.7994	0.0225	0	0	0.21797	1.89149	2.92982	0
Gm14548	12.7159	0.02762	0	0	0.03862	0.4468	0.55898	0.33257
Pdyn	12.5316	0.01534	0.03021	0	0	0.16467	0.26487	0.12257
Ccnb1	12.4927	6.8E-09	0.04136	0.05328	0.03897	0.78913	0.74548	0.67129
Cxcl10	12.0376	2.3E-05	0.10847	0.13973	0.25551	3.35062	2.48343	0.80688
Pla1a	11.9083	2E-06	0	0.07596	0.08334	0.76339	0.77554	1.01683
Hist1h2bl	11.6783	0.02909	0	0.39316	0	2.28755	3.34508	0.61917
Ch25h	11.4146	3.6E-07	0.0646	0.05548	0.66956	1.64334	3.90209	1.74743
Cd177	10.7203	5.8E-08	0.49131	0.15823	0.37615	4.43589	4.42775	4.40243
Morc1	10.645	0.04114	0	0.02456	0	0.10391	0.195	0.15469
Ptger3	10.5206	2.7E-05	0.01533	0.02633	0.04334	0.52933	0.3585	0.24884
Ighg2b	10.45	0.00028	0.13026	0.19577	0.15342	1.7456	3.36306	1.2773
Syt12	10.3253	5.1E-06	0.09447	0.05409	0.11868	0.67231	0.85901	0.48978
Cxcl9	10.2175	0.00255	0.05783	0	0.05449	0.57795	0.73246	0.15643
Celf5	10.0959	2.9E-05	0	0.0337	0.03697	0.35647	0.21662	0.21227
Arg1	10.0911	8.3E-05	0	0.15223	0.27836	1.44937	2.36014	1.59826
Reg3g	9.7685	0.00098	0.16501	0.60229	0.11661	0.52472	3.53686	5.97013
Ccl7	9.65168	0.00058	0.10887	0	0.20518	1.187	1.27288	1.47256
BC030867	9.46972	0.00512	0	0.0247	0.0271	0.31358	0.2522	0.11671
Trem2	9.40588	6.7E-25	0.17632	0.15143	0.18275	1.87428	2.11296	1.83629
Ereg	9.39191	4.9E-05	0	0	0.07963	0.24952	0.4734	0.22859
Piwil2	9.38846	0.00412	0	0.03049	0	0.16127	0.06918	0.21607
Fst	9.28326	4.7E-10	0.14926	0.19229	0.28129	2.47495	2.50859	2.06933
Gzmb	9.25948	0.02332	0.0615	0	0.1159	1.56453	0.29959	0.24955
Tnc	9.25517	2.2E-16	0.74057	1.14677	1.43793	11.9582	11.9598	13.158
Ccr5	9.1774	9.3E-13	0.19646	0.16873	0.25332	3.20349	2.13571	1.25868
Kcnj10	9.05407	0.00058	0.04839	0	0	0.17584	0.23571	0.13089
Gm21541	8.82262	0.01536	0	0	2.64466	12.5679	10.4497	0
Tnfsf8	8.70841	3.4E-05	0	0.01967	0.10792	0.45787	0.69186	0.24786
Gp2	8.67676	0.00458	0.08729	0	0.08226	0.07931	0.59535	1.12166
Iglc2	8.59316	0.00381	0.1425	0.36714	0.26854	4.0134	3.74845	1.15639
Ccl6	8.57893	2.8E-06	1.06884	1.37693	3.11297	23.7918	18.981	7.09683
Clec5a	8.57781	3.2E-11	0.06813	0.07802	0.1284	1.238	1.10631	0.5529
Sfrp1	8.55417	2.8E-20	0.99734	0.9936	1.52242	14.8787	12.7875	8.71419
Has2	8.49316	2.2E-06	0	0.08838	0.09697	0.5236	0.72191	0.25055
Hist1h2bf	8.27947	0.00675	0	0.16247	0.53475	2.9218	3.50181	1.2793
Ackr1	8.26436	0.0196	0.06207	0	0.05849	0.50754	0.60473	0.25185
Ddias	8.18246	2.6E-06	0.02356	0.02023	0.13318	0.8347	0.68853	0.25489
Igkv1-135	8.15066	0.00614	0	0.57028	0.41712	2.81536	9.05717	0.89811
Trip13	8.09385	0.00303	0.02579	0	0.07289	0.39827	0.25123	0.34876
Msr1	8.07855	3.5E-11	0.10997	0.14167	0.09067	1.43627	1.31251	0.70652
Hist1h2bk	8.03271	0.00354	0.91605	0.78673	0.69053	9.4879	6.24742	1.98238
AA467197	7.96657	1.3E-05	0.07249	0.24903	0.13661	1.91001	1.90694	0.88243
Upk3a	7.91822	0.02079	0.07885	0	0.0743	0.71639	0.53775	0.31994
Tnfrsf9	7.86259	0.02404	0.16019	0	0.06038	0.7277	0.78035	0.56333
Mmp8	7.82175	0.00538	0.03596	0.03089	0.06777	0.62079	0.38541	0.34048

Ighv1-81	7.79854	0.00723	0.24845	0.42676	2.80934	2.7088	23.7224	4.70463
Bcl2a1a	7.76584	0.04353	0.31944	0.27435	0	1.54788	1.76362	0.43206
Fn1	7.72741	3.1E-12	3.75399	4.89697	9.2546	65.4268	59.8135	38.6511
Clec4e	7.70697	0.00081	0.01756	0.06034	0.01655	0.46279	0.42782	0.07127
Ctss	7.6613	6.4E-16	8.7336	7.83161	13.3728	101.112	115.81	61.7554
Dcstamp	7.6415	0.00616	0.04376	0.03758	0.08246	0.31804	0.63947	0.65101
Egr3	7.63795	0.00013	0.12317	0.21157	0.34819	0.83933	3.96023	1.66599
Ccl9	7.51758	4.6E-11	0.17184	0.31976	0.53973	3.85103	3.54369	1.66566
Col24a1	7.50475	2.6E-08	0.12587	0.04914	0.05391	0.71736	0.62433	0.72743
Adamts19	7.48541	0.00444	0.01869	0.01605	0.01761	0.16981	0.21851	0.1264
Oas3	7.48193	1.6E-07	0.06674	0.07164	0.12577	0.98529	0.82901	0.33849
Egr2	7.47538	1.1E-09	0.07699	0.30858	0.29019	1.51563	3.55062	1.24964
Tnip3	7.43796	5.7E-07	0.0959	0.06589	0.07229	1.13264	0.59795	0.41505
2410018L13Rik	7.41942	0.00496	0.02116	0.01817	0.01994	0.15377	0.22674	0.25754
Klrc1	7.41857	0.01937	0.13187	0	0.12425	1.07826	0.42825	0.05945
Gm26545	7.28633	0.03337	0.04995	0.0429	0	0.31766	0.24331	0.40533
Fcrlb	7.24959	0.03243	0.06792	0	0.064	0.37024	0.4632	0.45931
Ms4a6d	7.18464	0.00226	0.05026	0.1295	0.09472	1.23301	0.83251	0.47589
Cdh3	7.18418	0.00778	0.01989	0.01708	0.01874	0.14458	0.27133	0.10762
Isg15	7.14778	3.6E-08	0.69212	0.89163	1.19564	13.2054	7.1928	3.12042
Shcbp1	7.08655	0.003	0.01385	0.0119	0.02611	0.21397	0.17546	0.09369
Lilrb4a	7.08351	2.8E-10	0.87793	0.53059	0.61278	6.8538	5.79738	4.8377
Igkv1-117	7.07736	0.00486	0.21586	0.92694	1.017	11.1788	10.5155	0.87588
Cdca3	7.06658	4.8E-05	0.06086	0.10453	0.08602	1.02289	0.97831	0.24693
Lrg1	6.94463	5.1E-22	2.45292	2.27296	3.22367	23.8694	22.0745	20.0806
Gas2l3	6.94034	2.2E-08	0.03877	0.14429	0.07306	1.05674	0.79324	0.40203
Tnfrsf8	6.87219	0.01457	0.05042	0.02165	0	0.20616	0.29476	0.1364
Shc4	6.86933	8.7E-05	0.01055	0.01813	0.0895	0.26849	0.20565	0.28549
Psrc1	6.81284	1.1E-09	0.34744	0.37299	0.38195	2.47272	3.5261	3.05453
Il1rn	6.81107	1.4E-07	0.11188	0.09608	0.23719	1.75338	0.92649	0.98356
Rgs16	6.74813	0.0025	0.18642	0.1601	0.35132	2.7777	2.61542	2.72313
C3ar1	6.74447	2E-08	0.12154	0.31316	0.40085	2.79752	3.15781	1.04115
Jchain	6.74372	0.00105	1.3166	1.81514	1.69766	12.686	23.3594	5.38914
Chrna7	6.58137	2.7E-06	0.01936	0.04988	0.10944	0.54522	0.54694	0.36655
Hist1h3d	6.56353	0.00793	0.46718	1.06995	0.29348	7.2158	5.76548	3.37005
Clca3a1	6.54254	4.5E-10	0.17893	0.11526	0.33721	2.07276	1.83048	1.17982
Ckap2	6.47557	4.3E-13	0.13563	0.20966	0.28115	1.99621	1.7178	1.24739
Cybb	6.44427	6.5E-11	0.57057	0.58029	1.00453	6.90281	7.62168	2.64011
Gpr141	6.42948	0.00178	0.05081	0	0.07181	0.36927	0.44549	0.13743
Col28a1	6.41584	2.2E-10	0.14226	0.22909	0.20108	1.42179	1.49	1.53934
Fcgr1	6.37553	5.7E-05	0.16842	0.08679	0.31739	1.86681	1.21425	1.27565
Cdkn1a	6.36126	2.6E-20	3.21683	3.97562	4.10312	30.3314	29.2772	26.5299
Hist1h2bm	6.25556	0.00577	0.22889	0.78632	0.43136	5.40693	2.89907	1.85751
Gdf15	6.21036	2.4E-05	0.14127	0.08088	0.31059	1.32624	1.74334	0.95533
Wisp1	6.19064	1.4E-07	0.22126	0.1493	0.34251	2.18255	2.0479	1.13292
Hist1h2bg	6.18986	0.00019	1.09695	1.57016	2.58406	16.6105	14.428	7.41836
Tmem37	6.1042	0.00016	0.19956	0.28565	0.3134	3.02183	2.0739	0.98968
Glp2r	6.07253	0.03635	0.015	0.02576	0.02826	0.14988	0.1315	0.08114
Fcgr3	5.99221	7.5E-08	0.55258	0.73344	0.99403	5.70509	5.92209	3.1934
Cthrc1	5.9675	0.00249	0.11785	0.15182	0.05552	0.96364	0.80372	0.71727
Eln	5.96554	1.1E-18	16.4813	17.8156	15.7183	114.745	122.237	120.345
Cenpn	5.94828	6.2E-05	0.13237	0.02274	0.09979	0.84187	0.61905	0.35808
Pif1	5.92809	0.00114	0.05962	0.01707	0.03745	0.30696	0.30981	0.21504
Sirpb1a	5.90731	0.01866	0.1142	0.06538	0.03587	0.51876	0.5563	0.30891
Cgref1	5.90704	0.00035	0.08377	0.07195	0.11841	0.76112	0.48971	0.79314
Kif18b	5.9029	0.00034	0.14649	0.20968	0.13803	0.8429	0.92767	0.66042

I830077J02Rik	5.88032	0.00065	0.09132	0.13071	0.02868	0.96793	0.7414	0.16468
Ccr1	5.80853	0.00036	0.06157	0.15862	0.17403	1.59415	1.04969	0.24981
Igkv8-30	5.74121	0.03485	1.18489	0.20352	1.11648	5.59793	9.4662	1.60261
Osm	5.73287	0.01286	0	0.0989	0.03617	0.48823	0.33657	0.20766
Il6	5.72188	0.04025	0.03592	0	0.10153	0.65265	0.20996	0.09716
Gm4841	5.70838	0.02156	0	0.02639	0.14477	0.7538	0.23951	0.29093
Etv4	5.69208	0.00172	0.16857	0.12065	0	0.53604	0.84855	0.684
Gm11427	5.68773	0.0129	0.08845	0	0.1389	0.34821	0.6032	0.23925
Foxp3	5.66093	0.01181	0.34979	0.30041	0.2884	0.41712	0.4899	0.97579
Ano5	5.64253	0.0316	0	0.00958	0.03152	0.12158	0.14125	0.03017
Gm6377	5.62568	0.02227	0.0624	0.05359	0	0.42522	0.36479	0.0844
Cxcl15	5.60906	9.8E-06	4.96103	4.57761	10.9719	68.4296	58.6805	15.1946
Cxcl13	5.60468	0.00422	0.2275	0.32564	0.14291	2.06693	1.40377	1.43594
Troap	5.55759	0.00678	0	0.06616	0.25407	0.76993	0.82563	0.57309
Ccl17	5.54529	0.00264	0.33933	0.14571	1.11909	4.00786	3.96723	2.75374
Ube2c	5.51242	0.00283	0.06708	0.05761	0.25284	1.34085	0.81697	0.40829
Mis18bp1	5.47775	5.3E-05	0.04366	0.01875	0.08228	0.40657	0.40409	0.14762
C1qb	5.46918	4.3E-10	4.24816	4.54337	6.34426	33.1348	34.8294	30.4637
2810417H13Rik	5.46173	0.00095	0.65229	0.4824	0.46098	1.30051	1.11215	0.78422
Tnfrsf11b	5.37691	3.7E-05	0.09284	0.18605	0.08748	1.18089	0.78392	0.50228
Nrcam	5.24978	6.1E-09	0.35661	0.27663	0.34686	2.5397	2.43205	1.36918
Tnf	5.21138	0.01783	0.05321	0	0.20054	0.67679	0.51839	0.43179
Csmd1	5.17652	5.3E-14	0.08249	0.0551	0.09932	0.57873	0.46434	0.42768
Slc7a8	5.14172	2.2E-06	0.21624	0.09285	0.14263	1.27699	1.05337	0.49719
Stag3	5.12161	0.03167	0.03042	0.02613	0	0.12438	0.1482	0.10287
Cldn2	5.1147	0.0136	0.13114	0.02253	0.04943	0.30978	0.53662	0.42569
Trp53cor1	5.11297	0.00168	0.08851	0.10135	0.0556	0.40206	0.57486	0.43893
RP23-181A8.7	5.08356	0.00278	0.02964	0.07637	0.08379	0.56556	0.25992	0.40092
Slfn2	5.06995	2.6E-05	3.19691	3.64581	4.83953	14.3323	15.0118	12.3339
Ly6c2	5.04494	0.00048	0.22279	0.43735	0.71977	1.56151	1.82954	0.99011
Lair1	5.03314	5.9E-06	0.12833	0.12024	0.21986	1.31436	1.18212	0.42605
Ms4a1	5.03171	0.03855	0.22552	0.12105	0.18594	0.79399	0.8377	0.74351
Tk1	5.02235	0.00171	0.72142	0.86453	1.45439	2.3474	1.87975	1.45227
Igha	5.01662	3.9E-06	11.8652	15.1309	15.677	79.1432	140.122	47.835
Ctla4	5.00656	0.01779	0.07486	0.06429	0.07053	0.40806	0.36466	0.55685
Serpina3i	4.9795	0.00034	0.27448	0.10103	0.22169	0.99753	0.76407	1.11375
Hpgds	4.97885	1.1E-06	0.17023	0.10443	0.13749	0.99427	0.99513	0.49338
Gm2115	4.97437	0.03366	0.01188	0	0.05598	0.09715	0.21994	0.09642
C1qc	4.94844	2.3E-08	1.30969	1.62903	1.87241	10.5041	10.2961	7.94083
Ms4a6c	4.92336	3.1E-07	0.46794	0.40188	0.96203	3.47849	4.18608	1.43844
S100a14	4.9226	5.3E-08	0.85844	0.73726	0.61856	4.08319	4.28022	4.78091
Ccl5	4.92124	0.04043	0.18168	0.31207	0.68478	5.11709	1.32758	0.7372
Gtse1	4.89393	1.2E-07	0.2564	0.08258	0.302	1.33947	1.62373	0.78028
Ccna2	4.89303	4E-07	0.12815	0.14675	0.32201	1.76589	1.41504	0.86666
Gcnt4	4.88469	0.00352	0.05137	0.04412	0.01613	0.24891	0.25024	0.18528
Plau	4.87487	3.8E-07	0.52109	0.47949	0.42086	3.31403	3.08238	2.16472
Hist1h1a	4.8674	1.3E-05	1.35837	1.04995	0.89597	7.89855	5.42611	6.24667
Atp6v0d2	4.86534	3.8E-06	0.3532	0.52395	0.6051	4.37586	3.50371	1.12914
Cd3g	4.85869	0.00063	0.16006	0.11456	0.07541	1.11492	0.70176	0.28866
Socs3	4.84946	8.3E-06	3.71032	3.16064	3.80879	18.472	11.2855	29.1718
Cdsn	4.82409	0.02573	0.03256	0.11187	0	0.4438	0.25382	0.22022
Icos	4.8236	0.00032	0.12712	0.06551	0.11979	0.71609	0.66882	0.44705
Htr7	4.81792	0.00699	0	0.0661	0.12088	0.39628	0.34996	0.38172
Cd300lb	4.81172	0.00224	0.08969	0.1027	0.16902	1.005	0.72819	0.40436
Adamts12	4.78764	1.1E-06	0.36371	0.32438	0.22408	1.97	2.37149	1.13524
Lilra5	4.77444	0.03958	0.04135	0.07103	0.07793	0.48839	0.48344	0.16778

Wfdc17	4.73545	0.02354	0	0.98363	0.46251	3.27038	4.78226	0.88519
Fcer1g	4.7156	5.9E-06	1.29714	1.54249	2.44451	12.873	11.9573	5.66817
Ptafr	4.70885	6.9E-10	0.2158	0.32949	0.76819	2.87563	2.92016	1.55669
Serpina3n	4.70389	4.3E-07	3.21113	4.89877	7.5644	37.8503	30.7092	19.6015
Ccne1	4.68948	0.00309	0.03276	0.16881	0.06174	0.65481	0.57452	0.35448
Cd52	4.67556	0.00042	1.02856	0.75717	1.59224	8.87775	6.80006	2.98109
Gpr176	4.64761	0.03805	0.04473	0.03842	0.0843	0.75188	0.69733	0.24201
Oas1g	4.63997	0.00226	0.26088	0.25606	0.52675	1.3544	1.77918	1.71383
Fbxw10	4.63491	0.00989	0.08667	0.11165	0.04083	0.43307	0.40108	0.5861
Ly86	4.62586	0.00039	0.8747	0.90146	0.8242	6.35766	6.2211	2.48444
Mab21l3	4.60875	0.00665	0.07247	0.02075	0.06829	0.35117	0.35304	0.19604
Rhof	4.59969	0.00023	0.3411	0.3255	0.58925	1.20518	0.96006	0.79455
Ccl8	4.59239	0.02351	0.34132	0.73284	0.32162	4.80666	2.66035	0.69248
Csf2rb2	4.58065	7.5E-08	0.47272	0.60086	0.6236	3.1954	4.14508	2.02042
Scd1	4.57793	4.1E-07	21.4232	17.7794	28.0368	149.945	156.946	72.0492
Prrx2	4.57244	0.00515	0.08894	0.15277	0.0419	0.76768	0.30329	0.60149
Tlr8	4.54314	0.00074	0.12159	0.06266	0.25207	1.08265	0.99513	0.32892
Gatm	4.53841	9.5E-05	0.05812	0.32444	0.27382	1.45211	1.41561	0.82539
H19	4.53611	0.00415	0.1103	0.03158	0.27714	0.73487	0.78804	0.7459
Pglyrp2	4.53043	0.00181	0.03897	0.03347	0.25702	0.77887	0.60743	0.42163
Pdcd1lg2	4.53019	0.02066	0.21205	0.04553	0.19981	0.43349	0.77476	1.29066
Tnfrsf26	4.52052	0.00049	0.0271	0.18619	0.10214	0.51705	1.00331	0.29323
Cd200r1	4.50493	5E-05	0.30012	0.14729	0.1616	1.32443	1.12785	0.98583
Rsad2	4.50059	3.8E-06	0.32239	0.38071	0.2848	2.3982	1.7472	1.33541
Tns4	4.50027	0.03491	0.03376	0.04349	0.03181	0.35269	0.21377	0.04566
P4ha3	4.49972	7.5E-08	0.25036	0.31058	0.28833	1.76914	1.3822	1.50497
Igsf6	4.48071	0.01577	0.60143	0.51653	0.71784	2.47715	2.50012	0.705
Ildr2	4.45257	1.6E-07	0.28537	0.31771	0.41829	2.19903	1.84326	1.38666
Lif	4.45206	5E-07	0.48115	0.55096	0.47226	2.47715	3.26187	2.41327
Ms4a14	4.45144	0.00077	0.10727	0.12897	0.18193	0.60423	0.79426	0.84148
F830016B08Rik	4.45058	0.00011	0.44966	0.56792	0.47355	4.1815	2.31933	1.71722
Csf2rb	4.43861	7.5E-14	1.15325	1.27343	1.32816	6.66922	7.93648	4.90228
Serpina3f	4.42197	0.00324	0.11425	0.13082	0.39472	1.66076	1.11308	0.82412
Tlr13	4.4179	5.8E-06	0.25776	0.16603	0.38455	1.61977	1.94624	0.78441
E2f8	4.4129	5.4E-05	0.11215	0.17888	0.10568	0.77151	0.96782	0.45508
Pdcd1	4.40351	0.0132	0	0.11642	0.12773	0.41053	0.61633	0.36669
C1qa	4.40346	3.9E-09	4.10882	4.17693	8.6914	27.6551	30.6364	28.8075
Slc11a1	4.38599	2.7E-09	0.47076	0.22237	0.48794	1.98883	2.66017	1.59179
Nuf2	4.3794	4.7E-05	0.08252	0.07087	0.09071	0.5248	0.53597	0.24182
Olr1	4.37762	5.8E-06	0.1709	0.20968	0.27606	1.75234	0.95145	0.79251
Neil3	4.36501	8.8E-05	0.09419	0.09438	0.11834	0.62758	0.55063	0.40344
Plcd4	4.34824	0.00136	0.08045	0.04146	0.15161	0.58474	0.3919	0.32644
Hmmr	4.34764	0.00354	0.12846	0.11032	0.13617	0.86074	0.67271	0.39093
Sirpb1c	4.34722	0.01677	0.18379	0.13154	0.2309	0.97405	1.19375	0.4143
Trac	4.34424	0.0034	0.74616	0.24031	1.05463	4.57598	4.45271	1.76612
Tmem26	4.30956	0.00514	0.03452	0.04448	0.13012	0.39208	0.52136	0.18678
Hist1h2ak	4.30351	0.00263	0.88761	0.95288	2.30001	6.8547	8.64783	3.90172
Kifc1	4.2892	0.01682	0.61432	0.43966	0.77181	1.97674	1.97013	2.14647
Igfbp2	4.28731	4.1E-06	3.82448	3.44679	5.33881	27.5404	24.4728	14.0495
Cldn4	4.28297	9.2E-10	1.87285	1.52598	1.80998	8.42061	8.04734	10.0025
Ifi211	4.27744	0.00199	0.68398	0.46155	0.46035	4.43878	2.47517	3.03966
Ctsd	4.27719	1.1E-05	18.7324	21.0577	30.4785	138.547	142.283	64.0683
Cd96	4.27632	0.03149	0.03993	0.03429	0.1505	0.5079	0.31123	0.37805
Hist1h2ab	4.27622	0.00428	0.73749	0.95006	1.73728	5.86285	7.00556	2.99244
Ngfr	4.25892	0.00052	0.02531	0.15214	0.07154	0.3219	0.51778	0.54766
Slfn10-ps	4.24935	0.00052	0.16859	0.14479	0.09077	0.72208	1.03244	0.45604

Prc1	4.24346	6.7E-05	0.29688	0.23676	0.35966	1.77249	1.51852	0.74571
Slamf7	4.24053	1.7E-06	0.41918	0.40909	0.66428	3.35833	3.2486	1.39161
Pilra	4.23402	0.00072	0.28223	0.14543	0.29253	1.58977	0.79741	0.9543
Fcgr2b	4.21659	9.7E-12	0.81334	0.69852	1.36246	4.94004	5.679	3.44281
Bcl11a	4.20001	0.0002	0.17872	0.23308	0.22453	0.9141	0.79967	0.78784
Serpina3g	4.18275	0.00023	0.74572	0.32022	1.13208	3.1994	4.35924	3.24998
Cep55	4.182	0.00011	0.18835	0.18872	0.17748	1.02677	1.2234	0.59443
Tgfbi	4.17021	2.3E-12	2.70285	3.91544	4.87884	24.2904	19.7342	13.8741
Thbs1	4.16698	7.8E-08	5.60036	10.2608	11.4066	38.1223	58.9844	38.262
Car13	4.13992	0.00111	0.07587	0.19547	0.21446	1.13731	1.03481	0.30783
Cd3e	4.11993	6.9E-06	0.32062	0.257	0.34239	1.94197	1.45773	0.78056
Prr11	4.11641	0.00687	0.06842	0.17627	0.17191	1.05671	0.66657	0.40099
Cd200r4	4.11109	0.01895	0.17058	0.18312	0.08037	1.0461	1.12179	0.11536
Gpr132	4.10722	3.1E-06	0.42907	0.42991	0.60644	2.11156	3.20491	1.98279
Ifi204	4.10229	0.00303	0.26939	0.16826	0.27691	1.31275	1.33615	0.9937
Ccr2	4.09697	7.5E-08	0.98044	0.79772	1.32498	6.69255	6.49806	2.58238
Slc7a11	4.08616	4.6E-05	0.08084	0.10099	0.1385	0.78792	0.67307	0.17893
Ticrr	4.08374	8.8E-05	0.03736	0.03209	0.08802	0.26309	0.26392	0.25268
B430306N03Rik	4.07349	0.0001	0.088	0.04319	0.10661	0.51398	0.34295	0.32306
Ogdhl	4.06951	0.02874	0	0.08686	0.04765	0.20675	0.36952	0.1368
Trbc2	4.04491	0.0023	1.26023	1.40702	1.18747	6.52636	6.13908	2.21586
Lhfpl2	4.02981	1.1E-07	0.30558	0.3077	0.48653	2.09665	2.39208	0.9549
Il9r	4.01744	0.01087	0.10577	0.13626	0.06644	0.27228	0.60113	0.64376
Cyp7b1	4.00276	6.7E-08	0.28183	0.62241	0.41732	2.30454	2.54973	1.68814
Rad54b	3.99127	0.00582	0.10558	0.11658	0.04264	0.4111	0.36737	0.1632
Ndnf	3.98308	1E-08	2.65572	2.66589	2.65678	17.1485	16.2134	6.68246
Lrrn2	3.95862	4.6E-05	0.07277	0.10417	0.22858	0.61711	0.63812	0.72182
Aspm	3.95852	1.6E-06	0.13544	0.13571	0.18434	0.88188	0.93835	0.3969
Pdpn	3.94703	3E-07	2.92772	3.21516	4.47724	23.2857	19.0318	9.28295
Lamc2	3.94211	1.9E-07	1.46449	1.49067	2.14658	10.5163	9.42763	4.68051
Mreg	3.93814	1.2E-05	0.38182	0.36071	0.28782	1.97734	1.7484	1.23942
Esco2	3.90091	0.0018	0.10874	0.07471	0.10246	0.63228	0.42377	0.29414
Slfn9	3.89696	0.00037	0.17544	0.36412	0.52348	2.43076	1.93717	0.57345
Oas2	3.87158	0.00168	0.70534	0.70673	0.73847	3.84502	3.22603	2.99451
Iigp1	3.87054	0.00021	1.70447	1.43986	2.60658	13.4804	9.9094	4.27061
Cx3cr1	3.86913	0.00483	0.54202	0.43226	0.63842	2.88437	2.64042	1.83277
Eda2r	3.86697	5.8E-06	0.19245	0.24156	0.15344	1.02218	1.12498	0.66074
Mki67	3.85728	9.8E-08	1.27418	0.93053	1.62533	7.53656	5.99593	4.33776
1500009L16Rik	3.84874	0.03127	0.1462	0.12556	0.27552	1.19546	0.78341	0.59322
Zwilch	3.8412	0.00192	0.14253	0.12241	0.21104	0.77697	0.47611	0.35801
Hist1h1b	3.82573	0.00048	1.2911	0.80642	2.98611	9.27754	5.9464	7.46129
Rad51ap1	3.82282	0.01787	0.05436	0.04668	0.08537	0.36217	0.33541	0.17155
Nlrp1c-ps	3.82079	0.00099	0.3784	0.25998	0.35655	1.44392	1.67128	1.46715
Ifi27l2a	3.81792	0.00093	1.50683	2.26469	2.30724	13.348	12.6622	3.05707
Fscn1	3.80357	1.5E-09	1.56348	1.6163	1.99158	8.28625	8.18055	7.12724
Il4i1	3.80141	0.00475	0.36337	0.20193	0.38267	1.3788	1.06206	1.06966
Cd86	3.79817	1E-05	0.14292	0.22504	0.38157	1.29854	1.34607	0.80546
Il2rb	3.78402	5.4E-05	0.28182	0.43029	0.70814	2.13372	3.14233	1.27058
Mx1	3.78253	0.03595	0.26311	0.34922	0.45076	2.17313	1.42152	1.19699
Kif14	3.77417	0.00769	0.05052	0.06508	0.11107	0.37482	0.45116	0.21636
Mpeg1	3.77086	1.8E-07	3.8826	4.59574	6.31227	25.0217	30.066	13.3053
Cd300lf	3.76718	0.00079	0.71135	0.64002	0.43089	2.83137	3.23424	2.40531
Ptger4	3.75489	3E-11	0.38042	0.47317	0.84052	2.31213	1.82761	1.68553
Bst1	3.75228	0.00082	0.39437	0.20322	0.44592	1.67207	1.7162	0.92455
Fignl1	3.73441	0.00061	0.10813	0.1393	0.16981	0.6877	0.91303	0.31688
Col5a3	3.73412	0.00011	0.67881	0.65998	0.48273	2.23419	2.18371	3.58585

Lgals3	3.73098	4E-07	2.11213	2.82173	3.93618	17.0575	15.868	7.55451
Cd53	3.71894	0.00057	1.15142	1.89176	1.5095	9.32412	10.1938	2.09903
F7	3.71785	2.7E-06	0.51602	0.56404	0.53044	2.38677	2.83369	2.09382
Tpx2	3.70819	1.6E-07	0.46615	0.3523	0.33382	2.16843	1.70765	1.26098
Wnt7a	3.70755	0.00024	0.08248	0.14167	0.23315	0.97417	0.66965	0.40904
Kynu	3.70618	6.3E-05	0.14182	0.0609	0.16036	0.69581	0.60798	0.30692
Gzma	3.68762	0.03887	0.59595	0.68243	1.49746	8.66321	2.22573	1.6121
Lpxn	3.68295	0.00095	0.52064	0.26829	0.34341	1.89211	2.43482	0.77461
5430427O19Rik	3.6826	0.00054	0.09292	0.15961	0.13134	0.52765	0.81479	0.40846
Ncapg	3.68234	0.00012	0.09873	0.13779	0.18607	0.62794	0.84171	0.38394
Itgb6	3.6816	3.7E-13	0.87073	0.97952	0.9938	5.11428	5.07806	2.72031
Slc28a2	3.68073	3.4E-05	0.12435	0.30258	0.23433	1.20505	1.11052	0.53258
Mmp14	3.67802	6.5E-17	2.65993	2.84388	3.75956	15.3385	15.0731	10.7196
Cd8b1	3.67483	0.0469	0.29402	0.15151	0.05541	0.85483	0.85939	0.55675
Tnfrsf12a	3.66419	2.2E-05	0.90558	1.47771	1.70661	6.33529	6.52898	5.2668
AI506816	3.6521	3.5E-06	1.27104	1.35441	1.55253	5.496	4.83876	3.24726
Knl1	3.64626	5.2E-05	0.2246	0.13152	0.20202	1.02959	0.90515	0.55234
Scimp	3.6406	0.04345	0.1615	0.20805	0.30435	1.17381	0.78671	1.09215
Sv2c	3.63137	0.0032	0.05624	0.0483	0.0265	0.24699	0.17353	0.16483
Dlgap5	3.62989	0.00085	0.13114	0.06758	0.13593	0.61956	0.47274	0.37248
Mest	3.62542	1.9E-05	0.19895	0.26579	0.49991	1.7875	1.98144	0.53818
Slc7a5	3.61787	0.00422	0.30574	0.54536	0.77564	2.22226	2.10808	1.30421
Cd28	3.61777	0.00095	0.1282	0.11011	0.10738	0.76359	0.54127	0.2312
P2ry10	3.60779	0.00367	0.2632	0.32651	0.22045	1.22223	1.68106	0.79109
S100a4	3.59518	2.3E-05	1.64261	2.82145	4.22123	16.959	12.9484	8.68481
Igf1	3.58302	1.4E-07	0.70442	1.00578	1.24455	6.02402	4.87279	2.17944
Tnfrsf1b	3.57515	1.8E-10	1.62172	2.23631	2.60423	10.8535	11.0379	6.27141
Tfec	3.57185	0.01133	0.07795	0.02678	0.17627	0.43908	0.57717	0.18977
Tnfrsf13b	3.5672	7.6E-05	0.369	0.36973	0.37668	1.64834	1.82752	1.3725
Itgax	3.56415	3.7E-07	1.72491	1.82447	2.17281	9.61741	9.14571	6.16405
Glipr2	3.5577	2E-08	0.63902	0.93621	1.4522	5.32769	3.95526	3.60973
Chsy3	3.55646	1.4E-07	0.32729	0.40885	0.42054	1.51382	1.62335	1.89142
Capn6	3.55363	0.00017	0.04475	0.08647	0.08433	0.36592	0.3488	0.22698
Cxcl16	3.55174	9.7E-10	2.56917	3.07421	3.9441	12.3792	14.0624	10.1905
Cd68	3.54765	0.00307	0.90191	1.43001	1.1767	6.68148	6.35378	2.53357
Gadd45g	3.54588	0.00022	2.00734	2.49017	4.27329	10.7399	8.54719	15.9884
Lox	3.54488	6.6E-05	1.77679	1.85295	2.63091	10.7565	10.2276	6.03247
Ly6c1	3.5336	8.8E-06	2.23609	3.43891	5.78202	20.0325	17.074	11.0426
B2m	3.53028	0.00645	10.2418	15.0664	27.0407	105.213	97.5121	27.0192
Tifab	3.52163	1.7E-06	0.47368	0.70657	0.49332	2.71809	2.67185	1.85459
Mtfr2	3.51321	0.04498	0.163	0.105	0.0768	0.62942	0.83377	0.38582
Mgp	3.51164	0.00211	19.9835	24.1032	44.3057	173.75	144.448	60.5162
Cenpa	3.50967	1.2E-05	0.45239	0.2391	0.29512	1.2963	1.76304	1.12963
Slfn1	3.50277	0.00055	0.42107	0.28126	0.52901	2.63541	1.68654	0.8859
Sh2b2	3.4978	8.8E-05	0.2102	0.58672	0.47041	1.90979	1.25438	2.1323
Grrp1	3.49438	1.9E-06	2.18874	2.23222	2.06239	7.58144	7.73014	11.4714
Col12a1	3.48764	2.4E-05	0.61277	0.65414	0.82562	3.31956	3.30865	2.22302
Mdga1	3.48164	0.00043	0.05569	0.03986	0.13994	0.26144	0.32557	0.3892
Tlr1	3.47974	0.00329	0.41701	0.20146	0.22103	1.08927	1.4474	1.1633
Mt2	3.46782	0.00074	1.92806	3.69388	4.75151	17.3826	13.1493	10.4311
Tnfaip8l1	3.45148	0.00022	0.14663	0.22038	0.37995	1.19898	1.14287	0.79329
Ptpn7	3.44837	8.2E-06	0.25725	0.25776	0.37706	1.37637	1.04431	1.19846
Tlr2	3.4454	9.8E-06	0.72335	0.64609	1.03603	4.20609	4.20032	1.76106
Ncapg2	3.4446	2.3E-08	0.26411	0.32889	0.39817	1.67967	1.64681	0.83945
Ncf4	3.43978	0.01061	0.94932	0.88942	0.65055	2.86193	3.23716	4.14378
Nos2	3.43268	0.00034	0.27672	0.18673	0.33525	0.93383	1.25175	1.09611

Slamf9	3.42877	1.7E-06	0.7306	0.62747	0.87618	3.01722	3.23553	3.05435
Cd84	3.42191	8.2E-08	0.83426	0.91949	1.00882	4.62357	4.47041	2.59524
Ulbp1	3.41787	0.03529	0.23971	0.20587	0.09035	1.08895	0.65394	0.51875
Kntc1	3.4166	8.9E-06	0.15294	0.16888	0.14411	0.82379	0.61731	0.50236
Zbp1	3.41352	0.03049	0.69193	0.59425	0.53556	2.85137	2.81692	2.00542
Ms4a7	3.41341	0.00049	0.33293	0.25229	0.25835	0.94303	1.41195	1.13899
Fabp5	3.41305	0.00075	0.7579	0.44094	1.38222	3.99827	5.28798	1.65338
Adam11	3.41291	7.9E-05	0.23075	0.2246	0.20293	0.72677	0.89926	1.08194
Ifi207	3.40855	6.3E-08	0.72652	0.73154	0.96785	2.73136	3.78327	3.35456
Serpine1	3.405	6.4E-05	1.39496	2.18352	2.12005	6.48004	9.14096	7.48611
Aim2	3.40469	1.6E-05	0.42346	0.25672	0.30513	1.42577	1.62601	1.04442
Ear2	3.4004	0.03061	0.48516	0.83334	1.71432	6.06088	4.37232	2.2967
Ccnf	3.3914	0.00029	0.25327	0.24168	0.29168	1.30392	1.17893	0.76123
Susd3	3.38745	0.00766	0.21518	0.132	0.14482	0.6982	0.62893	0.7068
Ccng1	3.38275	3.9E-09	4.54413	4.90497	5.91965	24.3427	26.733	12.9975
Sh2d1b1	3.37377	0.01672	0.13712	0.17664	0.32301	1.1835	1.03534	0.37092
Pld4	3.36109	6E-08	1.10222	1.59032	1.95255	6.76959	7.86076	4.35313
Gda	3.35453	6.1E-09	0.59702	0.95223	1.27245	4.40395	4.00242	2.94159
C330027C09Rik	3.35173	0.00036	0.18398	0.079	0.17336	0.86364	0.64232	0.24884
Aoah	3.34966	0.00156	0.1795	0.25694	0.31009	1.00569	1.19505	0.84974
Slfn8	3.34528	1.5E-05	0.40502	0.476	0.64276	2.88575	2.22225	1.06679
Birc5	3.33597	0.00253	0.15381	0.13209	0.38647	1.25766	0.87412	0.55474
Ptges	3.32639	3.8E-06	0.5768	0.83832	1.17062	3.78928	4.02022	2.19041
Ms4a4b	3.32243	0.01219	0.2811	0.09941	0.29604	1.51734	0.83773	0.4473
Serpinb9	3.31558	2.4E-05	1.39772	1.60769	2.21073	9.84168	8.72994	2.97074
Kctd12b	3.30794	0.00269	0.10665	0.1374	0.28474	0.85595	0.98716	0.28851
Gyg	3.3069	0.00231	1.22775	1.53372	2.80455	9.70122	10.2581	2.76764
Lax1	3.29731	0.00022	0.45322	0.42168	0.605	1.71573	2.39182	1.78792
Hexb	3.29587	0.00044	1.46567	2.36019	3.23687	10.2786	11.6024	5.82325
Col5a2	3.29373	7E-09	5.05551	5.09932	5.53476	19.2071	18.9756	22.7147
Malt1	3.29002	6.3E-09	1.10019	0.88583	1.44164	5.66949	5.14178	2.92961
Samsn1	3.28896	0.00274	0.26928	0.11563	0.16311	0.83881	0.86202	0.49428
Slc15a3	3.28834	2.8E-08	0.60351	0.4774	0.61357	2.55402	2.19723	2.10513
Cmah	3.28746	1.3E-09	0.76084	0.67596	0.80756	3.39272	2.81171	2.60223
Slc27a6	3.26422	0.00346	0.45622	0.21098	0.56215	1.97682	2.15404	0.75945
Espl1	3.26083	3.5E-09	0.27622	0.24853	0.30985	1.29066	1.21745	0.78279
Atp1a3	3.25933	8.3E-05	0.13173	0.23884	0.48272	1.01068	1.08381	1.16804
Fcrls	3.24752	9E-07	0.82201	0.93059	1.021	3.80206	4.14996	2.98165
Gla	3.23719	2.3E-06	0.29381	0.43585	0.52853	1.86857	2.00377	1.08378
Ercc6l	3.23343	0.01499	0.08224	0.0565	0.07749	0.29888	0.41665	0.17797
Iqgap3	3.21765	0.00628	0.2528	0.16448	0.2743	1.09272	1.00758	0.62167
Ptgir	3.20985	3.8E-05	0.3662	0.33697	0.39435	2.04378	1.35067	0.88446
Nlrp1b	3.20136	3.7E-05	0.37345	0.48704	0.32583	1.75932	1.40148	1.4592
Ifi205	3.20052	0.00301	0.08351	0.41241	0.33444	1.63131	0.91535	0.87539
Siglec1	3.19942	2.6E-06	0.39068	0.62912	0.5637	2.4514	2.52171	1.2715
Nckap1l	3.19572	1.1E-09	2.16309	2.01651	2.33437	9.13766	9.02426	7.05161
Mmp25	3.19386	0.002	0.20684	0.15543	0.12181	0.61074	0.75569	0.52454
Plk1	3.1897	0.002	0.2776	0.30653	0.29895	1.44124	1.04323	1.01914
Hrh2	3.17759	0.02698	0.08816	0.11358	0.06923	0.5607	0.40085	0.13912
Clec4a2	3.17729	0.0208	0.17883	0.10239	0.14042	0.7582	0.58076	0.3628
Melk	3.17647	0.00657	0.08803	0.126	0.19354	0.58651	0.60036	0.39688
Il21r	3.17487	0.00099	0.52471	0.49571	0.59331	1.59704	1.89152	2.55491
Zc3h12d	3.17089	0.00691	0.19176	0.31108	0.18069	0.7356	1.16248	0.77808
Cotl1	3.16904	1.5E-06	5.027	5.72519	7.5171	24.8718	25.9792	18.7718
Cd3d	3.16814	0.0108	0.14414	0.16506	0.58857	1.57155	1.31075	0.5199
Tmem151a	3.16753	0.02918	0.16328	0.06232	0.10257	0.28021	0.56561	0.41714

Hvcn1	3.16252	0.00029	2.00702	1.19818	1.8681	4.55869	4.53083	3.77393
Cenpe	3.16187	0.00904	0.37776	0.35224	0.41697	1.86314	1.5668	1.02186
Cdk1	3.16119	0.00586	0.02531	0.17393	0.47706	1.10396	1.01119	0.4451
Nxpe5	3.15725	0.01766	0.03899	0.11721	0.07348	0.30113	0.22794	0.39555
Themis	3.15537	0.02522	0.0676	0.05805	0.09099	0.51763	0.31047	0.19591
Fbn2	3.14695	0.02182	0.04161	0.03573	0.06273	0.17389	0.22701	0.13506
Nsl1	3.13657	0.00274	0.09867	0.21185	0.12396	0.41835	0.73701	0.53382
Selp	3.13636	0.00111	0.07905	0.18669	0.20483	0.66431	0.80864	0.34747
Thy1	3.13146	3.8E-07	1.13573	0.90573	1.64346	5.7858	4.82126	3.29168
Hbegf	3.11411	2.1E-11	1.6905	1.70435	2.87416	8.01338	10.0254	5.51734
Tmem59l	3.11262	0.02535	0.2622	0.13511	0.29648	0.80995	0.97074	0.85113
Clec4a3	3.11032	0.03304	0.39431	0.05644	0.49539	1.43299	1.08847	0.97775
Sh2d2a	3.10627	0.00154	0.11747	0.16394	0.26289	0.73376	0.70101	0.59582
Pik3cg	3.10068	1.3E-06	0.44979	0.48563	0.71444	2.40522	2.3664	1.4253
Naip5	3.09967	1.1E-06	0.50273	0.24467	0.55266	1.87271	1.66534	1.22394
Il2ra	3.09818	0.00626	0.05023	0.17254	0.1262	0.4259	0.55458	0.3623
Fbn1	3.09672	3E-09	4.99028	5.47768	6.97876	25.7988	24.1206	15.4053
9330175E14Rik	3.09116	0.01098	0.13218	0.06307	0.08303	0.41364	0.34341	0.29796
Klrk1	3.09113	0.01019	0.14137	0.20814	0.36158	1.50464	0.88546	0.30048
Naip2	3.08858	5.3E-08	0.4139	0.44821	0.61045	2.50156	1.9111	1.14398
Ppfia4	3.08723	2.5E-07	0.31847	0.20513	0.38761	1.10916	1.12476	1.1307
Cd300a	3.08698	8.9E-06	0.36812	0.31615	0.45093	1.689	1.56014	0.99579
Cr2	3.08281	0.02147	0.15051	0.12003	0.2026	0.60557	0.56559	0.56708
Sash3	3.08031	1.1E-05	1.00988	0.58753	1.07436	3.90686	3.77692	2.29118
F10	3.07503	8.7E-05	0.31932	0.52107	0.5416	2.00184	1.6489	1.2957
Lgmn	3.0748	4.2E-11	6.15671	5.93241	8.02984	27.5591	28.8581	18.6347
Tlr7	3.07114	0.00681	0.19228	0.16513	0.40261	1.02874	1.37376	0.43343
Fjx1	3.07111	0.00039	0.50087	0.52576	0.5244	1.9214	1.95197	1.88181
Adgre1	3.06977	0.00034	0.94061	0.8309	1.3168	3.66251	5.8127	2.18093
Cxcr3	3.06819	0.02658	0.2519	0.30288	0.3323	1.09854	1.42344	0.81769
Slc39a14	3.0615	3E-11	0.63649	0.65313	0.89573	2.71868	2.77042	2.4932
Lrp2	3.05938	2.7E-06	0.91902	0.91934	1.20546	4.62556	4.45149	2.36596
Apobec1	3.05215	5.1E-05	1.25873	0.88701	1.64225	4.89704	5.18846	3.79786
Btk	3.0502	7.7E-05	0.60926	0.4519	0.60019	1.98773	2.21249	1.9103
Cd180	3.03904	0.00352	0.4255	0.29899	0.45561	1.59905	1.77128	0.62782
Ccnb2	3.0352	0.0236	0.34177	0.34244	0.37571	1.70782	1.83138	0.38521
Cstb	3.02797	1.6E-05	4.51325	3.12591	4.11552	16.7988	17.7306	9.05807
Cd276	3.02655	2.2E-06	0.54833	0.42997	0.5616	2.14434	1.85817	1.64449
Praf2	3.02562	0.018	0.65129	0.39154	1.16601	2.66277	2.03053	1.32134
Itgb2	3.01542	6.6E-06	3.37172	3.60741	4.71177	17.9649	15.6734	9.23673
Msln	3.01134	0.00296	1.7326	0.70858	0.73855	2.32375	4.17992	4.96581
Tnfrsf13c	3.01121	0.03505	0.21618	0.25993	0.57036	1.13919	0.92674	1.63741
C1qtnf6	2.97284	3.3E-08	1.37964	1.55905	1.50525	5.77254	7.07451	3.38829
Tgm1	2.97157	0.00012	0.29011	0.3239	0.62874	1.50241	1.55458	1.37336
Nusap1	2.96414	0.00446	0.10742	0.22142	0.30367	0.93697	0.9001	0.46495
Trim59	2.96351	0.01356	0.3122	0.11172	0.31869	1.25278	1.16599	0.28151
Ifit1	2.96215	0.00012	1.05069	1.07156	1.73257	6.41376	4.67051	2.70898
Loxl2	2.95533	7.5E-06	1.6296	2.20325	2.06763	7.9159	7.35681	6.19766
Gpr65	2.94549	0.00888	0.29793	0.14621	0.52135	1.35341	1.1196	0.92105
Bub1	2.94367	0.03599	0.10015	0.06881	0.18873	0.65511	0.39028	0.21672
Spc25	2.93883	0.00039	0.27355	0.25843	0.25776	1.01898	1.19932	0.59198
Hist1h4f	2.93851	0.00042	4.01467	3.83103	6.30485	19.0482	21.7302	12.9717
Rrm2	2.93745	0.00243	0.50354	0.62166	0.74137	2.94512	2.45297	1.31956
Cenpf	2.92694	0.00039	0.36894	0.32346	0.56491	1.94135	1.52766	0.98761
Lpcat2	2.92573	4.6E-05	0.54054	0.46424	0.65486	2.12814	1.85579	1.9148
Il2rg	2.92148	0.00209	1.20564	1.09819	1.68684	5.84201	5.44601	2.17423

Bhlhe40	2.91551	7.3E-08	2.07891	2.92994	3.742	11.5991	10.8803	8.11099
Pou2af1	2.91347	0.00133	0.47897	0.35259	0.70922	1.77177	2.39995	1.24939
Top2a	2.91335	0.00026	0.89985	0.79775	1.07583	4.28116	3.45026	2.17242
Slc29a3	2.90749	0.00016	0.59793	0.65474	0.94371	3.35454	3.02926	1.47591
Tmem173	2.90358	2.8E-05	1.02285	1.39903	1.35647	5.74795	4.8351	2.86938
Ctsz	2.90319	7.6E-08	6.53603	8.00423	10.1505	34.0902	32.4883	20.3817
Mmp19	2.89953	0.0001	1.02296	0.85659	1.39767	3.99647	4.18595	2.66343
Mthfd2	2.89207	0.00449	0.15006	0.27922	0.18852	0.90889	0.85282	0.43974
Runx3	2.88911	3.3E-05	0.43578	0.38923	0.55844	2.04297	1.61337	1.17881
Irf7	2.8859	0.0022	1.29945	1.71122	1.36048	5.69318	5.79562	3.9838
S100a6	2.88204	8E-05	5.0398	5.42412	8.89659	28.3428	22.9971	15.7902
Ccl22	2.88016	0.00034	0.63765	0.90198	0.77755	3.20337	2.81389	2.13074
Dusp5	2.87272	0.00117	0.67001	0.90857	1.32913	2.69127	3.74492	3.4341
Mms22l	2.87184	0.00055	0.16398	0.25818	0.23176	0.94354	0.87867	0.48053
Basp1	2.86293	0.0012	1.74699	1.94642	1.95756	5.9199	5.9802	7.28019
Tlr9	2.85799	0.00404	0.30089	0.53836	0.51978	1.75414	1.83219	1.15307
Itm2a	2.85746	0.0061	0.45004	0.81596	0.801	2.77131	3.84875	0.74396
Sh3pxd2b	2.85596	5.5E-12	1.3563	1.49655	1.54038	5.22285	5.59198	4.36139
Cd8a	2.85388	0.00732	0.28773	0.51482	0.45187	2.35279	1.35495	0.77835
Nxpe4	2.85259	0.00014	0.32458	0.39279	0.37535	1.38063	1.62427	0.75828
Rasgrp1	2.84849	0.00015	0.45161	0.60185	0.60163	2.40527	1.92688	1.41121
Gpr35	2.84723	0.0002	0.28835	0.47981	0.2717	1.40814	1.51002	0.75563
Psat1	2.84501	0.00483	0.164	0.36973	0.40565	1.63903	1.07854	0.55455
Mcm10	2.84413	0.01482	0.11337	0.10711	0.08546	0.32962	0.46393	0.27602
Cd1d1	2.84193	0.00343	0.23753	0.23314	0.51158	1.17153	1.55383	0.59664
Ifi44	2.83412	0.00546	0.15652	0.15886	0.24133	0.84028	0.77632	0.30792
Lipa	2.83021	0.0039	1.82111	2.24513	3.3766	10.4077	12.2482	3.49605
Zranb3	2.82836	0.01343	0.20171	0.07424	0.19912	0.81161	0.70188	0.1559
Plek	2.82462	1.4E-08	1.2636	1.61948	2.17981	7.11784	6.55325	3.73365
Pole	2.82407	0.00074	0.2819	0.24211	0.17994	0.8262	0.84168	0.73797
Enc1	2.80865	1.1E-06	0.79079	1.31093	1.29966	3.95994	4.89148	2.78587
Col1a1	2.80582	0.00155	95.9775	94.0574	79.3561	219.045	275.349	388.686
Serpinb6b	2.7953	0.00522	1.50631	2.48521	3.88455	12.8212	11.1613	3.21687
Btla	2.79071	6.5E-05	0.77649	0.66687	0.78212	2.84623	2.99999	1.73831
Kif4	2.78936	6.6E-05	0.22142	0.21733	0.31296	1.02022	0.84749	0.72731
Mex3a	2.78875	0.0013	0.09056	0.19444	0.128	0.49366	0.54408	0.38787
Tbxas1	2.7856	3.6E-06	0.20138	0.25531	0.48794	1.33303	1.07444	0.86901
Litaf	2.78169	1E-05	1.71643	2.35292	3.63901	10.6163	10.8698	4.64307
Fhl3	2.78123	0.00125	0.41453	0.53402	0.87886	1.55357	2.06985	2.31278
Pde1b	2.77918	7.4E-05	0.70852	0.58315	0.72326	2.27987	2.67493	1.83676
Gm9930	2.77579	0.04704	0.22141	0.04754	0.20863	0.65377	0.51232	0.41176
Sema6b	2.7746	2.4E-05	1.18411	1.29944	0.99178	3.44661	3.5678	4.50809
Hsd11b2	2.77085	0.02839	0.23698	0.12211	0.35727	0.81816	0.87736	0.70515
Bmper	2.7664	0.00016	0.50524	0.43391	0.57809	1.86892	1.98656	1.24468
Ccdc80	2.76499	0.00112	1.32707	1.66437	2.46123	6.41131	6.40315	5.09985
Brinp1	2.76128	0.00075	0.21163	0.13329	0.39883	1.05113	0.86601	0.53432
Hsd17b7	2.76127	1.1E-05	0.20503	0.29348	0.43469	1.16427	1.34839	0.60085
Acsbg1	2.75927	0.03988	0.07259	0.10391	0.1596	0.2858	0.4715	0.39274
Ect2	2.75733	0.00022	0.27526	0.2503	0.42719	1.19157	1.13581	0.83219
Phlda3	2.75683	1.5E-10	3.97161	5.38065	5.58715	16.8731	17.8759	14.8291
Ackr3	2.75081	0.00197	0.57305	0.58163	0.98176	2.29556	2.46165	2.21952
Tyrobp	2.74561	2.3E-06	7.06029	5.83902	8.50068	22.9263	26.3684	21.2207
Hpse	2.74496	0.00026	0.24803	0.36213	0.51417	1.39716	1.57074	0.77158
Kif22	2.73852	0.00035	0.33795	0.54992	0.30168	1.14736	0.97043	0.81794
Itga4	2.73618	3.3E-05	0.87345	0.85195	1.15224	4.08122	4.08473	1.88176
Myc	2.73323	0.00057	0.96291	0.85454	1.3005	2.21629	2.65809	4.94903

Tnfaip8l2	2.73067	0.02966	0.6218	0.46727	1.02533	2.75406	1.59025	2.52303
Scarb1	2.72636	1.9E-05	1.26468	1.37261	2.02978	6.89416	5.49733	3.12032
Racgap1	2.72049	0.0001	0.3819	0.24334	0.38306	1.37669	1.17623	0.78312
Diaph3	2.7172	4.5E-05	0.43756	0.44115	0.51986	1.74574	1.57548	1.2351
P2ry6	2.71137	0.00332	0.7618	0.74772	0.99127	3.88911	2.72142	1.6682
Ikzf1	2.71129	2.2E-05	0.75478	1.27053	1.3513	3.94996	4.10338	2.98095
Cd48	2.70853	0.00337	0.73345	0.62991	2.00423	4.66464	3.35858	2.77767
Irf5	2.70674	3.9E-05	1.20079	1.44379	2.06494	5.31852	5.44009	4.42573
Ctsb	2.70669	6.6E-05	7.34244	10.0199	14.0626	41.2295	44.9656	18.2939
Psap	2.70609	2.4E-09	50.1825	55.2991	79.1486	205.184	249.538	147.87
Ms4a6b	2.70458	0.00094	0.66111	0.53438	0.73287	2.82657	2.4817	1.07827
Lacc1	2.70201	1.8E-05	0.81368	0.51384	0.58631	1.89166	2.7047	1.71555
Sema4d	2.7012	4.7E-06	1.21704	1.33557	1.46533	4.41067	5.34901	3.43851
Cks1b	2.69894	0.01291	0.3098	0.93122	0.58382	1.89989	2.49009	1.99031
Pidd1	2.69505	0.0014	0.30815	0.49622	0.39925	1.20738	1.48238	1.25037
Aif1	2.68996	0.00338	0.85386	0.73332	0.59002	2.22388	3.10577	1.84781
Cd83	2.68969	0.01937	0.85458	0.76889	0.65186	2.62505	3.25111	1.70626
Cyth4	2.68702	8.2E-06	3.68703	3.40805	3.70972	11.6961	10.5635	12.2981
Ptprc	2.68106	4.2E-05	1.73002	1.62489	2.37932	7.65167	7.45218	3.77374
Dtl	2.67677	0.00573	0.11398	0.10605	0.11635	0.40561	0.40719	0.28264
Cfb	2.67245	0.00294	1.45089	2.33307	2.70517	8.27381	8.15062	3.71601
Hmox1	2.66652	0.00087	1.39452	1.6254	2.67497	8.0092	6.84183	3.57022
Vcan	2.66612	1E-05	0.37387	0.55932	0.6648	2.20242	1.87414	0.99503
Ubash3a	2.66397	0.03471	0.18343	0.15754	0.17284	0.64281	0.66379	0.35442
E2f1	2.65865	7.7E-06	0.41475	0.73614	0.57318	1.93433	1.88571	1.79508
Slc40a1	2.65152	0.0043	0.36116	0.27694	0.35247	1.11331	1.45776	0.66294
Ltbp2	2.6481	7.2E-09	6.47877	6.14072	8.26351	22.8964	24.2935	19.8172
Lgals3bp	2.64662	3.5E-05	5.05418	6.39266	7.33123	22.3754	24.5315	13.7548
Kcnab2	2.64472	0.00165	0.50029	0.52366	0.61873	1.77555	2.02588	1.41678
Apoe	2.6432	0.01053	17.337	21.6235	33.329	74.1269	84.6254	66.5173
Zfp618	2.63663	7.9E-05	0.10258	0.14416	0.24603	0.55069	0.59962	0.41621
Brip1	2.63536	0.00065	0.14039	0.12057	0.16536	0.44644	0.51294	0.37978
Lyz2	2.62792	0.0101	107.873	129.501	257.035	615.834	668.377	269.91
Plac8	2.62594	0.00021	1.09557	1.36432	1.85819	6.17136	5.44373	2.22271
Foxm1	2.62293	0.0022	0.3789	0.33843	0.48556	1.37699	1.53569	0.86096
Gimap9	2.62293	0.00154	0.42488	0.50174	0.9008	2.26792	1.86281	1.65219
Naip6	2.619	1.6E-05	0.43431	0.30718	0.48146	1.18378	1.45611	0.91569
Mybl1	2.61464	0.00074	0.19372	0.15251	0.19775	0.63069	0.62914	0.45853
Atf3	2.60995	0.02674	0.44535	1.70378	1.06818	2.722	3.82618	3.5046
Armcx4	2.60879	4.5E-05	3.13014	2.86623	3.87435	10.3054	11.1253	9.36711
Skap1	2.60219	0.00293	0.24542	0.0803	0.24227	0.74327	0.54654	0.50582
Itgam_1	2.60184	0.00021	0.97206	1.01373	1.57019	3.94269	4.14339	2.48863
Ccr7	2.60036	0.01805	0.83769	0.79516	0.9555	2.16307	4.03777	2.02748
Dck	2.59848	0.00019	0.45332	0.48666	0.98779	2.08506	2.76039	1.18795
Mcm5	2.59332	0.00022	0.546	0.75028	0.78201	2.71846	2.55342	1.32928
Clec2i	2.58539	0.04104	0.19629	0.28899	0.26422	1.07002	0.84692	0.37927
Inhba	2.58337	0.01195	0.22022	0.60523	0.37351	0.80033	1.33026	1.72758
Lag3	2.57822	0.01264	0.53755	0.31962	0.3117	1.50275	1.08774	1.06263
Fgr	2.57605	3.1E-05	0.903	0.83369	0.65943	2.85097	2.72732	1.98469
Icam1	2.57265	1.8E-06	7.42065	6.37309	9.51777	33.4011	27.0095	15.3392
Mafb	2.56843	9.1E-06	1.60775	2.65022	2.54119	5.4188	6.94777	8.13703
Rad54l	2.56805	0.003	0.2037	0.3013	0.24526	0.59635	0.6836	0.35205
Ltf	2.56311	0.03369	1.97165	2.86561	1.9531	3.23819	5.95986	10.6328
Chaf1b	2.56255	0.02278	0.12207	0.22465	0.16431	0.6971	0.50969	0.40096
Bard1	2.56153	0.00873	0.12328	0.19852	0.14521	0.67205	0.54051	0.25012
B4galt6	2.55872	9E-06	0.37538	0.42555	0.46689	1.28234	1.62381	1.03573

Kmo	2.55626	0.00714	0.31383	0.33294	0.3305	0.97279	1.07915	0.97378
Lrrc25	2.55098	0.00695	0.54279	0.41437	0.56828	1.31506	1.82152	1.63142
Rac2	2.55	0.00509	0.94791	1.18414	1.51571	5.35001	3.80607	2.21451
Tifa	2.54882	2.3E-05	0.35439	0.5619	0.66787	1.83282	1.99199	1.06927
Lamb3	2.54788	1.5E-05	1.26273	1.22004	2.0376	5.43512	5.36701	3.13827
Trim6	2.54686	0.02595	0.17819	0.10713	0.15112	0.43712	0.45139	0.43382
Itgae	2.54508	0.00208	0.64725	0.44143	0.78926	1.93714	2.15148	1.64788
Cd300c2	2.54325	0.00011	1.50476	1.76228	1.48234	5.7793	5.13121	3.97798
Pirb	2.54103	3.2E-05	1.05328	0.88784	1.4152	3.8987	4.33281	3.08664
Col5a1	2.54031	8.4E-05	6.97455	7.42792	6.97843	19.8034	18.7043	25.2298
Col8a1	2.53859	0.00393	0.25609	0.29325	0.48261	1.19438	1.3307	0.64656
Clu	2.53456	1.5E-06	4.46438	7.05833	9.46498	25.2354	22.9094	15.576
Myo1f	2.53291	0.00034	1.96989	1.92043	2.34112	6.33665	7.15822	5.52074
Stat4	2.53245	0.00424	0.29279	0.2708	0.382	0.81852	1.18494	0.97481
Myo5a	2.53161	6.1E-09	0.94849	1.07769	1.13243	3.7253	3.82839	2.24701
Cd274	2.53027	0.00903	0.8427	1.03391	1.27049	3.74067	3.073	2.73549
Kif11	2.52895	0.00087	0.41622	0.55951	0.59682	2.20318	1.6397	1.02801
Sdk2	2.52721	0.00036	0.10148	0.11136	0.15936	0.41488	0.42293	0.29738
Scml4	2.52463	0.00034	0.2032	0.24238	0.18083	0.69745	0.67092	0.56495
Nfkbie	2.5193	0.00317	1.44026	1.48433	1.59461	4.54719	5.12175	4.09082
Lck	2.51891	0.00799	1.13141	0.76712	1.20637	3.57073	3.30693	2.5773
Phex	2.51469	0.02403	0.19407	0.34526	0.33961	1.05794	1.4046	0.29999
Capg	2.51446	1.7E-06	1.75863	1.98421	1.55963	5.38867	6.11452	4.80389
Shisa4	2.51358	0.0003	0.50492	0.52657	0.57773	2.06436	1.65151	1.17074
Ntrk2	2.51278	0.00057	0.20213	0.26582	0.31546	0.70589	0.99083	0.67494
Prune2	2.51107	0.00242	0.20213	0.2574	0.243	0.76623	0.79451	0.58448
Plppr3	2.50578	0.00214	0.86067	0.94545	1.37678	2.18221	1.95008	1.35359
Cdc20	2.50364	0.02913	0.42017	0.68161	0.92379	3.43566	1.59195	0.94715
Gm4951	2.50329	0.03564	0.75833	0.76454	0.86989	3.5647	2.18435	1.5608
Rap2b	2.49942	0.00028	0.53919	0.5182	0.83468	2.55438	2.20136	1.04182
Cdca2	2.49758	0.00155	0.27467	0.19658	0.23006	0.79024	0.77308	0.55725
Ndc1	2.49691	0.00898	0.07084	0.25858	0.45058	0.98155	0.93178	0.43118
Adam12	2.49594	0.00548	0.24456	0.21003	0.25604	0.71595	0.68833	0.7228
Irf4	2.49528	0.00013	0.40691	0.55597	0.76683	1.81486	2.18044	1.2258
Epha3	2.495	0.04826	0.09246	0.23823	0.39206	0.84007	0.99093	0.35433
Ttyh2	2.48893	1.6E-05	0.60642	0.88137	1.03293	2.64885	2.88594	2.05051
Spi1	2.48321	0.00101	1.19728	0.9171	1.64651	4.55696	3.94086	2.75732
Sgk1	2.48301	1.5E-06	1.63637	2.29135	3.13408	7.04574	8.8552	5.19633
Slc37a2	2.47947	0.00097	0.28881	0.36078	0.47006	1.21657	1.47087	0.67472
Arhgdib	2.47719	0.00793	1.43047	1.6673	2.5995	8.49415	6.07248	3.52405
Plat	2.47691	0.03533	0.2096	0.66004	0.6715	2.15823	1.52477	0.94498
Prcp	2.47417	0.00211	1.07428	1.29168	1.79958	4.92355	5.86125	1.90505
Rab3il1	2.47209	2.6E-05	0.67186	0.84079	0.75969	2.38935	2.13207	2.25882
Ctps	2.46156	4.6E-11	2.44581	2.75555	3.44454	9.31863	9.35229	6.90068
Dok2	2.45053	0.00883	0.96163	0.54198	0.82117	2.75757	1.69813	2.35741
St8sia4	2.44663	0.02086	0.37678	0.37236	0.49607	1.83825	1.6293	0.39094
Il10ra	2.44242	1.1E-05	1.96953	1.88419	2.13773	6.41016	6.94682	4.48473
Sfn	2.44171	0.00183	3.08173	4.17898	5.04349	11.1996	9.74489	13.7476
Plbd1	2.44141	0.04262	1.49045	1.19471	1.96617	6.0184	6.09889	2.01589
Selplg	2.43986	0.00697	1.67546	1.90659	2.48651	7.57312	6.48867	3.96572
Tmem86a	2.4373	5.8E-05	0.97051	1.23042	1.13222	3.52703	4.32254	2.06275
Map2	2.43581	6.8E-05	0.20146	0.1457	0.20482	0.72734	0.60951	0.30117
Col3a1	2.43441	1.9E-05	41.2495	40.7674	40.2578	112.811	131.464	114.143
Cyp4f18	2.42942	0.00209	1.22232	0.88826	0.97456	2.26378	3.20622	2.92495
Tacc3	2.42634	0.00444	0.7611	0.82235	0.94851	2.56523	2.29635	2.42412
Epsti1	2.42587	0.00215	1.24206	1.09905	1.13489	3.93255	3.41033	2.90171

Pgf	2.42586	0.00686	0.33075	0.61546	0.62331	1.75293	1.23527	1.71485
Spry4	2.42305	6.3E-05	1.90119	2.18796	2.38261	6.01108	7.22396	5.63145
Rubcnl	2.42258	0.00329	0.49344	0.31077	0.43396	1.25528	1.21791	1.15683
Cers6	2.41688	2.3E-05	1.0194	1.36501	1.71452	4.73043	4.63484	2.66859
Hcn1	2.41641	0.00333	0.06738	0.07027	0.06802	0.23175	0.24852	0.12368
Rhoh	2.41332	0.01183	0.1752	0.22571	0.33018	0.8357	0.58322	0.69116
Lrp8	2.41299	0.00957	0.14101	0.09261	0.1016	0.30897	0.34749	0.32534
Tcf19	2.41274	0.00233	0.68513	0.7145	0.96837	2.97899	2.19326	1.72096
March1	2.41251	0.00039	0.61018	0.41924	0.66695	1.3416	1.43867	1.17193
Scube1	2.40935	1.9E-06	0.78368	0.56536	0.76797	2.28792	2.29057	1.61113
Lgals9	2.40917	0.002	2.04042	2.52343	3.53764	10.3443	9.02528	4.4708
Stc2	2.40685	0.01264	0.15167	0.17367	0.15879	0.5818	0.49255	0.34189
Cd6	2.40517	0.04025	0.48676	0.35204	0.53108	1.39657	1.59746	1.00488
Ptpro	2.40349	0.02606	0.12972	0.1114	0.19556	0.44784	0.55607	0.26317
Ifitm3	2.40209	0.00226	8.48924	16.4596	21.5735	62.8716	51.1293	22.4422
Lat2	2.3986	0.00238	0.96491	1.02872	1.31678	3.11367	4.44113	3.5102
Zfp385a	2.39626	0.00075	0.64711	1.14421	1.14777	2.97424	3.37486	2.21385
Ptpn22	2.39332	0.00284	0.84272	0.79379	0.81968	2.32164	2.67502	2.13255
Vstm4	2.38938	0.00041	0.85903	0.84706	1.0193	2.60157	3.37876	1.93646
Gm5431	2.38853	0.00817	0.45162	0.27151	0.4681	1.18992	1.47402	0.85517
Wdhd1	2.38779	0.00156	0.33952	0.20828	0.37705	1.04659	0.88604	0.72162
Nrros	2.38298	5.3E-05	1.39883	1.61377	1.61317	5.46299	4.86153	3.1627
Ifi30	2.38221	1.1E-05	8.27582	9.47672	11.6552	31.8546	29.9978	22.6275
Prkcb	2.38215	0.00675	0.82635	0.9778	1.12471	2.58602	3.38142	2.43405
Ppa1	2.38025	0.00493	0.24041	0.41294	1.01938	1.96579	1.66885	1.09742
Sparc	2.37929	3.3E-05	42.9822	46.6424	56.9008	167.459	159.231	98.8641
Emcn	2.37779	0.00192	0.45144	0.4154	0.5621	1.75779	1.5551	0.82865
Lrrc8c	2.37691	9.6E-06	1.70906	1.96685	2.40486	6.97708	6.77144	3.89885
Tspan32	2.37531	0.0132	0.55117	0.47336	0.4905	1.33538	1.22316	1.03539
Pdgfc	2.37027	0.00422	0.11996	0.22076	0.35524	0.5605	0.88488	0.55627
Kif2c	2.37009	0.02684	0.1599	0.1545	0.20718	0.6356	0.4479	0.43255
Havcr2	2.36782	0.00643	0.73715	0.71566	0.72479	2.56246	2.5605	1.21376
Kif15	2.36326	0.04325	0.26262	0.16333	0.15359	0.70758	0.60878	0.3552
H2-Eb1	2.36187	0.00238	28.0626	31.8184	46.0836	98.7058	121.204	79.7968
B3gnt7	2.36086	0.00722	0.34606	0.3658	0.4515	1.08836	1.11523	1.26017
Col4a1	2.35946	3.9E-06	23.1431	24.0304	34.6877	93.0446	91.3547	62.604
Tnfaip3	2.35901	4.2E-08	0.8448	1.01576	1.0917	2.73026	3.35109	2.35055
Atp6v0c	2.35639	0.03798	4.61293	5.843	8.11048	15.898	18.2032	8.60937
Lrp12	2.35617	2.9E-05	0.66731	0.8351	0.79047	2.37316	2.82349	1.41831
Hells	2.35444	0.00095	0.22997	0.23909	0.33075	0.95673	0.89623	0.42564
Ptprn	2.35312	0.018	0.18472	0.22915	0.19339	0.52212	0.73987	0.4164
Clmp	2.3531	0.00463	0.3989	0.50573	0.64436	1.58775	1.73964	1.2846
Tnfrsf10b	2.35205	0.0013	0.49503	0.51962	0.75151	1.47421	2.22393	1.33909
March3	2.35173	0.00039	0.23232	0.24941	0.34205	0.76515	0.91953	0.62845
H2-Ab1	2.35053	0.00107	18.3258	21.3664	29.3655	70.5201	72.2487	51.8066
Cd74	2.34546	0.00212	73.9718	79.5711	131.535	318.334	296.594	188.89
Tmem106a	2.34243	0.0004	1.00047	0.93084	1.15221	3.38341	2.89715	2.44323
Spn	2.3424	0.00037	0.93151	1.00801	1.61503	4.11311	3.32164	2.545
Ubash3b	2.34073	2.1E-05	0.44044	0.43961	0.44867	1.2762	1.58889	0.86942
Themis2	2.33916	7.1E-06	1.18958	1.33788	1.6013	4.3489	3.8357	3.44776
Rtn4rl2	2.33764	0.04449	0.23163	0.54705	0.6002	0.947	1.63611	1.40978
Irgm1	2.33566	0.00586	0.99065	1.30199	1.27291	4.41846	3.62673	2.19256
Fermt3	2.3329	3.7E-05	1.92147	1.82392	2.50935	5.84977	6.14165	5.47129
Sh3bp2	2.33266	0.00061	1.17738	1.02861	1.18593	2.89559	3.38198	3.21238
Runx2	2.33183	0.00238	0.29605	0.33901	0.38125	1.11178	0.92301	0.68072
Slco5a1	2.32512	0.00839	0.11949	0.08552	0.07506	0.27141	0.32338	0.19753

Pld3	2.31448	0.00032	3.0123	2.71513	4.35597	11.0828	10.083	6.97868
Ifi209	2.31165	0.02275	0.86052	0.73904	0.71818	3.06028	2.61099	1.69595
Nme2	2.31039	0.00517	1.71287	6.6518	5.0525	15.6976	15.5999	10.8786
Pik3r5	2.31007	8.7E-05	1.27276	1.13357	1.27331	3.52619	3.50576	3.20914
H2-T23	2.3089	9.5E-06	7.51634	7.93298	9.24413	17.8814	19.0281	13.3507
H2-Aa	2.30456	0.00229	16.0082	19.4236	28.9404	68.7892	67.7362	42.0447
Bcl3	2.30157	0.00307	0.91612	1.30124	1.89247	4.09767	3.43293	3.81259
Anxa1	2.30134	2.3E-05	7.49022	8.09849	9.38943	28.1931	29.9071	13.161
Gnpda1	2.29815	0.00934	0.80208	1.12721	1.30543	2.98115	3.23236	1.97244
Ifit3b	2.29387	0.01417	1.04649	0.93621	1.39694	3.48622	3.22867	1.94621
Sirpa	2.28803	1.1E-06	4.86558	5.12427	5.57192	16.2143	15.7266	11.3605
Elovl1	2.28726	0.00085	5.88635	4.94549	8.1088	21.2177	20.6334	11.0769
Hck	2.28663	0.00184	2.00094	1.61107	2.19967	5.71897	5.88906	4.45421
Tspan8	2.28006	0.00107	2.75031	3.64149	4.48119	13.0666	12.1139	5.26986
Vav1	2.27428	0.00359	2.17546	1.64497	1.96075	5.37095	5.50613	4.98929
Eef1e1	2.27354	0.02475	0.42252	0.87089	0.9555	2.45682	2.38758	1.37153
Rcan1	2.27285	8.1E-05	3.16676	3.40711	3.54138	6.82928	9.79843	10.5903
Olfm1	2.2671	0.00011	1.32542	1.53883	1.73459	5.44123	4.08922	3.1206
Pmaip1	2.26647	0.00082	0.62621	0.53781	0.90061	2.06615	2.31197	1.24817
Ly6a	2.26643	0.02315	5.71734	9.69617	15.0708	37.0847	31.8001	14.6828
Kcnk13	2.263	0.01274	0.25221	0.33694	0.52811	1.09479	0.81907	1.13707
Dock11	2.25898	0.00181	0.50965	0.53498	0.56027	1.80931	1.89424	0.79145
Pim1	2.25724	1.3E-06	3.63641	4.51424	3.80028	10.9628	10.1455	9.74735
Fam105a	2.24773	0.00287	0.83202	1.05861	0.98724	3.13569	3.03232	1.75052
Rab8b	2.24258	0.0051	1.60301	2.24706	2.91678	7.416	7.52172	3.56373
Dock2	2.2413	0.00359	2.00749	1.67165	2.2728	6.32471	5.68168	4.23285
Rpl39	2.23681	0.04834	3.46641	4.90955	6.01687	15.6365	13.5683	6.16903
Nme1	2.23626	0.04374	0.73064	1.12054	1.15564	2.58418	3.20334	2.2941
Kif20b	2.23393	0.00788	0.36056	0.47122	0.4136	1.6664	1.11494	0.63609
Serpine2	2.23361	0.00021	4.16752	5.48303	6.66326	16.1123	16.3278	11.4234
Plcl2	2.23054	1.5E-05	0.81824	0.70273	1.07535	2.58239	2.76923	1.63094
Fstl1	2.22948	0.00023	6.15813	8.68765	10.7357	25.3788	25.1402	15.4581
Rad18	2.22807	0.00555	0.25578	0.25844	0.2552	0.72452	0.7916	0.56982
Arpc1b	2.22382	1.8E-08	3.81906	4.7149	5.78345	13.9257	14.302	9.1087
Mcemp1	2.21676	0.01077	0.81721	0.70184	0.77003	2.12135	2.44544	1.57901
Sesn2	2.21287	3.2E-06	1.7316	1.74579	1.89176	5.15295	5.28127	3.97133
Ugcg	2.21062	0.00019	1.60628	2.14114	2.88521	7.02328	7.17279	3.64356
Stx11	2.20922	0.00176	0.94633	0.40637	0.8231	1.98411	2.05675	1.723
Itk	2.20868	0.00269	0.48865	0.44765	0.49114	1.21349	1.44412	1.18965
Myo1g	2.20245	0.00324	1.94296	1.55025	2.14971	5.05665	4.87293	4.88287
Txk	2.20172	0.04198	0.1148	0.10955	0.13221	0.27813	0.43495	0.24153
Zmynd15	2.20087	0.01499	1.12743	0.78141	0.89461	1.95879	2.23541	2.67525
Laptm5	2.19948	2E-05	7.25195	7.75066	9.17615	24.2633	22.2954	17.6243
Fkbp10	2.19349	0.00199	1.51536	1.76077	2.51979	5.56111	4.95026	3.85805
Syn2	2.19122	0.00564	0.31008	0.20239	0.36231	0.8001	0.78548	0.73814
Srxn1	2.18724	0.00078	1.17514	1.46075	1.74837	4.32687	4.00721	3.22067
Cd4	2.18722	0.01317	0.41095	0.48296	0.3057	1.17905	1.28543	0.7606
Eno1	2.18638	0.00182	2.01036	3.11895	4.99547	10.1342	9.90388	5.41627
Nrep	2.18449	1E-04	6.17706	7.15289	9.41739	20.7461	23.8362	15.3483
H2-DMa	2.18448	0.00999	2.39143	3.05794	4.70706	10.6222	8.69842	7.11593
Il4ra	2.18398	6.1E-08	5.44866	5.49059	6.39368	16.7784	14.3543	14.7391
Adamts7	2.18311	0.00239	0.22211	0.32191	0.2747	0.69369	0.79798	0.56328
Fgd3	2.18138	0.0011	0.72528	0.66027	0.92944	1.93732	2.27534	1.84422
Tbc1d2	2.18008	1.5E-06	0.78768	0.9388	0.93912	2.74575	2.41191	1.89158
Grn	2.18001	0.00093	5.37203	6.74803	10.6915	23.5599	23.9927	12.5625
Napsa	2.17986	0.02637	4.16367	4.42059	9.4839	16.4124	18.8456	8.38079

Glrx	2.17931	0.01154	1.03741	1.55919	1.40519	4.24143	4.42197	2.01702
Cenpl	2.17843	0.03952	0.13437	0.14425	0.20575	0.47307	0.36002	0.45436
Ier3	2.17656	0.00032	8.1733	9.86703	9.15458	23.3283	22.9879	24.4038
Trim30a	2.17412	0.00011	1.78191	2.11336	2.13878	6.82273	6.20031	3.44301
Crlf2	2.17343	0.00028	3.20831	2.95579	3.13303	7.15476	7.50192	9.46771
Rab32	2.17261	0.01798	0.66192	1.13695	0.50676	2.70623	2.09591	1.39886
Slc35f6	2.16925	0.00238	0.75709	1.07692	1.62741	3.89068	3.08879	2.048
Rnf180	2.16904	0.01825	0.35327	0.82352	0.66576	1.55897	1.86844	1.5017
Arhgap22	2.16849	0.04719	0.58498	0.46376	0.44521	1.63534	1.1618	1.58243
Tlr3	2.16733	0.00282	0.43545	0.66166	0.6786	1.68903	2.12127	0.8608
Sbno2	2.16657	0.00208	6.64321	6.2724	7.65939	15.7827	15.1558	21.012
Ncf2	2.16648	0.00041	1.44786	1.0092	1.12701	3.46975	3.65946	2.5543
Uhrf1	2.1656	0.01577	0.71666	0.63308	1.21552	2.84633	2.59343	1.21857
Casp3	2.15984	0.00063	0.80531	1.07091	1.27286	3.02107	3.41682	1.82708
Ncapd2	2.15968	0.00012	2.76916	2.70092	2.33395	4.79161	5.05695	3.21841
Ikzf3	2.15857	0.04325	0.97628	0.81279	0.79789	2.33515	2.18381	2.22322
Pappa2	2.1577	0.02002	0.14402	0.13824	0.19957	0.44642	0.58602	0.22917
Cd248	2.15567	0.00055	1.05603	1.25803	1.73334	3.74496	3.48489	3.22525
Psmb9	2.15402	0.00205	1.14402	1.28956	1.54959	3.74616	3.64567	2.99796
Dhfr	2.1532	0.03599	0.14837	0.21237	0.23301	0.71893	0.44972	0.37905
Lpl	2.15319	0.00238	2.15138	2.46356	3.58377	8.854	6.88702	5.30004
Hcls1	2.15307	0.00216	3.212	2.96621	3.15675	8.62926	7.50383	7.98803
Fam49b	2.15221	0.00059	1.14954	1.35507	1.63539	4.44389	4.17248	2.25599
Apbb1ip	2.15202	0.00052	0.88219	0.74755	0.86451	2.96026	2.07427	1.5273
Klhdc8a	2.14754	0.00064	0.81712	0.75441	0.71221	2.13439	1.87086	1.93409
Lhx6	2.14721	0.02813	0.21779	0.23381	0.35913	0.72554	0.63657	0.73643
Tap1	2.14253	0.02511	2.67547	2.73545	3.4514	8.79716	7.77345	6.24654
Cdh11	2.14018	0.01419	1.06757	1.58138	1.8919	5.14334	4.68528	2.21226
Plxnc1	2.14	0.00013	1.13517	1.42415	1.57299	3.52885	4.4239	2.76967
Crlf1	2.13731	0.00679	1.11397	1.36673	1.29958	3.85559	3.61773	2.36765
Fam20c	2.13692	1.8E-05	0.97058	1.06413	1.32318	2.7205	3.23925	2.68136
Nlrc5	2.13533	0.04564	2.14605	2.12529	2.54463	6.2785	5.64232	5.47192
Mirt1	2.13479	0.00536	0.21138	0.31985	0.21814	0.8322	0.57859	0.54456
Hn1	2.13359	1.6E-05	2.31309	3.17152	4.28264	8.81178	9.44933	6.75108
Cfp	2.13351	0.00489	1.1554	1.03544	1.75139	3.24049	4.16015	2.71778
Sdcbp	2.13326	0.00062	2.47577	3.33043	4.28481	10.0991	10.9037	4.647
Nfam1	2.133	0.01936	1.05559	1.25525	1.96379	5.06575	3.87643	2.12328
H2-DMb1	2.13063	0.00172	4.22825	3.48005	4.48218	12.0582	10.9854	8.8166
Ctsc	2.12763	0.0035	4.39942	3.946	5.94228	16.4658	14.8337	6.45811
Fmn1	2.12378	0.00288	0.31718	0.23349	0.35758	0.84908	0.81119	0.61286
Arhgap19	2.12135	0.00135	0.96709	0.78685	1.15106	2.06559	3.00849	2.31773
Flt3	2.12012	0.01684	0.48009	0.40019	0.57213	1.18027	1.34821	1.165
Abcb1b	2.11923	0.00174	0.18656	0.5098	0.52737	1.01698	1.00795	0.96343
Snx20	2.11446	0.03359	1.84784	1.85148	1.35424	3.91731	3.95069	4.99855
Nav3	2.11021	0.00379	0.12002	0.10307	0.11904	0.32138	0.26463	0.29048
Zfp365	2.10869	0.00394	0.48588	0.58996	0.58413	1.40046	1.82826	1.04242
Hist1h1d	2.10806	0.03599	4.10251	4.53004	6.29553	16.7197	8.67921	11.8903
Frrs1	2.1063	0.0002	0.62463	0.54677	0.58857	1.84439	1.61504	1.05604
Plcg2	2.10554	0.01443	2.11019	1.84715	1.95013	4.97737	4.68513	5.21425
Fras1	2.10432	0.02624	0.22653	0.09727	0.16705	0.29977	0.62852	0.33303
Ctsk	2.10221	0.0155	1.61496	2.03093	2.98911	7.54593	6.46228	2.9644
Prox1	2.10078	0.00733	0.23907	0.22317	0.28403	0.66106	0.6785	0.44988
Slc25a45	2.0974	0.00215	0.62526	0.9892	0.77522	2.57131	2.34054	1.20177
Ifit3	2.09641	0.03545	6.44687	5.5797	7.72287	20.5232	15.8244	7.36777
Tgfb1	2.09444	7.6E-05	3.07452	2.97056	4.2852	9.54396	10.1097	6.49753
Pou2f2	2.09379	0.01852	0.61362	0.43613	0.4785	1.13422	1.33998	1.3737

Vmp1	2.09314	1.5E-07	4.31296	5.64343	7.97902	16.7615	18.2822	10.2925
Stat1	2.09257	0.00455	2.78622	3.03592	3.71254	9.75767	9.06452	5.22939
Nek6	2.09021	0.00111	0.60233	0.86683	1.08143	2.15201	2.5139	1.28807
Bgn	2.08809	0.01389	19.4339	23.9846	35.0787	88.9723	75.1879	36.4482
Cmtm3	2.08629	0.00089	1.6063	1.35446	1.92636	4.19247	5.03643	3.12063
Metrnl	2.08555	0.04704	1.5396	0.85003	1.70978	2.79761	4.33932	3.12357
Itga5	2.08406	0.00149	2.04178	3.79373	4.77278	7.77696	9.52556	8.57687
C5ar1	2.0831	0.004	0.78046	0.93257	1.21501	2.46637	2.38034	2.47837
Arhgap11a	2.07314	0.02637	0.93226	1.06754	0.86303	2.42214	2.51771	2.25639
Nlrp3	2.06965	0.01469	0.23645	0.35076	0.4456	0.80072	0.90054	0.90128
Enpp1	2.0659	0.00431	0.14454	0.26287	0.24835	0.51756	0.73724	0.41399
Disp2	2.06436	0.00443	0.48373	0.80736	1.0234	2.28866	1.90293	1.51838
Tuba1c	2.06344	0.00181	2.29378	2.34881	3.61612	7.69479	7.09115	7.04012
Sgpl1	2.06323	0.00017	1.64677	2.01171	2.40781	5.66221	6.8326	2.89935
Gusb	2.06165	0.00012	1.76409	2.05851	2.51145	5.15672	6.44523	4.17552
Dab2	2.05874	0.02839	1.11652	1.59138	1.66764	4.48933	4.30495	2.26522
Marcksl1	2.0579	6.6E-05	2.77108	3.26652	4.04466	8.29347	8.52296	8.01044
Ifit2	2.05644	0.01486	1.26581	1.18248	1.44384	4.17649	3.93777	1.77213
Rnf149	2.05467	2.4E-05	2.39683	2.50711	2.92442	6.53289	7.57439	6.02643
Psmb8	2.05184	0.02258	2.03994	2.13521	2.67303	7.21083	6.52143	4.18177
Dpp7	2.05146	0.00075	0.78401	0.83044	0.83725	2.32687	2.29153	1.48456
Rab11fip5	2.05027	8.9E-07	1.50947	1.69997	2.16033	4.20485	4.80043	4.16029
Adap1	2.05007	0.00539	0.8477	0.9328	0.62404	2.21427	2.21962	1.61234
Cpxm1	2.04978	0.02231	2.04393	2.40211	2.87201	6.28776	7.40648	4.51049
Nrp2	2.04951	9.4E-06	1.55386	1.65706	2.21358	5.07162	5.01524	3.35668
Clec10a	2.04933	0.04952	0.24066	0.64588	0.56691	1.1479	1.23095	1.17993
Nt5e	2.04915	0.00666	1.1922	1.4805	1.62434	4.5376	4.45779	1.89577
Plekho2	2.04865	4.5E-05	1.69793	2.55192	2.94798	5.65639	6.08096	5.7413
Cd38	2.04865	0.01405	1.1201	1.05359	2.06061	4.04642	4.33919	2.12818
Tgfb2	2.04761	0.00067	1.12944	1.15106	1.73116	4.00882	3.60928	2.09792
Slc36a1	2.04752	3E-06	1.42608	1.41114	2.11787	4.07006	4.92331	3.22869
Col1a2	2.04698	0.00242	37.349	42.814	40.3317	78.6317	98.4478	112.723
Mfsd7a	2.04535	0.01902	0.62589	0.8063	0.65529	1.76914	1.79551	1.64606
Gja4	2.04462	0.01173	3.20883	2.84474	4.73045	12.8841	8.16873	5.67009
Ckap4	2.04449	4E-06	3.81127	5.26144	6.06523	14.1074	12.075	10.8444
Panx1	2.04326	0.01947	0.31817	0.3602	0.42245	0.94606	1.09905	0.68463
Gimap3	2.04102	0.00229	1.89508	1.91819	2.39154	5.16532	5.83577	4.94327
Rhod	2.03784	0.04933	0.86495	0.43697	1.00679	2.2651	1.58628	1.30752
Psd4	2.03467	0.01317	0.76952	0.70537	0.76693	2.0235	1.90317	1.60125
Gja1	2.03311	0.02221	2.55584	3.25638	3.51982	10.6919	9.19427	3.7228
Cdkn2c	2.03027	0.00619	0.71428	0.80864	1.49906	2.27136	3.19488	1.88828
B3gnt5	2.02704	0.02179	0.35239	0.30264	0.49016	1.03671	1.07902	0.68088
Serinc2	2.0251	0.03794	0.82733	0.61364	0.815	1.98166	2.12504	1.42417
Scn3b	2.02385	0.00259	2.37941	2.85268	3.43078	8.35704	8.33935	4.70857
Sh3kbp1	2.02011	0.00028	0.98432	0.78604	1.02513	2.36912	2.52371	1.95029
Trpv2	2.01205	0.00119	1.04999	0.88507	1.31917	2.50858	2.70903	2.60362
Ncaph	2.00994	0.02996	0.40496	0.38257	0.53421	1.08537	1.42036	0.71204
Coro1a	2.00951	0.0473	5.60911	4.8442	6.20064	13.4094	13.708	12.8844
Fzd1	2.00801	4.8E-05	3.28301	3.53337	5.48213	10.0055	11.6202	7.92529
Tmsb10	2.00678	0.00247	16.9494	19.722	31.9419	67.4344	50.8058	47.4211
Bend4	2.00395	0.00848	0.16761	0.19698	0.30755	0.58507	0.70475	0.33407
Arhgap15	2.00091	0.00248	0.6725	0.56907	0.65232	1.61735	1.59948	1.31087
Phf24	-2.00086	0.01939	0.45331	0.49454	0.76193	0.31168	0.37004	0.34799
Tef	-2.00483	6E-05	20.269	19.3027	20.6556	13.4891	15.3185	8.32337
Aldh5a1	-2.00604	0.02355	1.359	1.32631	1.41151	1.01022	0.82753	0.75195
Sec14l2	-2.0063	0.00353	1.46797	1.38895	2.20379	1.2207	1.01812	0.77401

Fam131a	-2.00638	0.02981	3.36237	3.04522	3.22587	1.61075	2.11444	2.06715
Plpp3	-2.00832	0.00158	14.8797	19.3702	24.1915	13.6694	13.6094	7.99041
Tox2	-2.00995	0.03533	2.10262	1.91591	2.41615	1.23473	1.1242	1.45662
Gnao1	-2.0101	0.00381	3.01439	2.81397	2.72511	1.81787	1.91535	1.48902
Hs6st2	-2.01033	0.00219	0.51384	0.72911	0.83152	0.42625	0.45709	0.3626
Ift88	-2.01331	0.00085	3.65563	4.1915	3.67542	2.08866	2.75383	2.11539
Fermt1	-2.01359	0.04388	0.59679	0.69653	0.60559	0.37538	0.40254	0.37254
Ppp1r26	-2.01432	0.00018	1.27265	1.39956	1.66716	0.84605	0.78629	0.98661
Abcc3	-2.01442	0.00061	4.85167	5.32347	6.72038	3.9491	3.92169	2.31846
Myh11	-2.0147	0.00276	20.9077	29.577	37.4438	17.3231	17.5387	16.4336
Rfx2	-2.01532	0.00049	2.75694	3.10487	3.45554	1.74864	1.82448	2.00515
Foxa1	-2.01653	0.00331	3.82969	4.86312	5.28404	3.20607	2.85171	2.29392
Cdhr3	-2.01714	0.00026	10.8677	10.551	11.7708	6.43947	7.39451	6.0314
Jag1	-2.01933	5.1E-06	7.54293	9.30585	9.80099	5.3302	5.87624	4.67597
Chchd10	-2.02851	0.00991	11.5162	13.3203	18.7274	10.2943	9.95329	5.65265
D630039A03Rik	-2.02858	0.01458	2.15708	1.58792	1.59701	1.0639	1.32101	0.83358
Cyb5rl	-2.03149	0.00179	0.96245	0.7661	0.80735	0.58651	0.5489	0.381
1700007K13Rik	-2.03154	0.04345	5.33186	5.58815	6.30137	3.69477	4.40233	2.20014
Adam28	-2.0354	0.00129	3.81533	3.09356	3.57275	2.02387	2.44736	1.82697
Cracr2b	-2.03875	0.02487	14.5136	13.1421	13.1109	8.11242	6.60906	8.23615
Adam22	-2.04111	8.4E-05	1.06951	1.19409	1.40415	0.73849	0.89612	0.54005
Wdpcp	-2.04135	0.0071	1.02874	0.99711	1.31555	0.82784	0.84478	0.29816
Kank3	-2.04562	0.01013	25.9273	22.8197	28.4365	14.4082	16.4316	16.3423
Kif21a	-2.04564	3.9E-05	5.45468	5.36711	6.56117	3.29166	3.73977	3.16967
Cyp2b10	-2.04762	0.01449	17.7966	19.327	24.5897	14.3684	15.2068	7.42784
Map9	-2.04991	0.01282	1.37263	1.54935	1.4412	1.03331	0.86608	0.68957
Tle2	-2.05019	0.00563	4.9905	4.44251	4.5439	2.98029	2.71789	2.63548
Ppp1r9a	-2.05187	9.8E-06	4.51912	4.38113	4.72195	3.05893	2.70138	2.28907
Rbp4	-2.05215	0.04025	3.02571	3.7535	4.18152	2.99336	2.35832	1.0913
Rsph9	-2.05384	0.00094	3.15733	3.5064	4.36	2.42346	2.54577	1.47256
Mtcl1	-2.0559	0.00074	8.40272	8.06505	8.75108	4.38565	5.18884	5.06526
Ctxn1	-2.05663	0.02585	6.69167	7.72449	6.30537	3.6609	4.4866	4.08744
Mcf2l	-2.05957	0.00032	4.42872	4.36702	4.57877	2.11121	2.84992	2.79165
Clcn2	-2.06061	0.00324	0.81096	0.80842	0.9006	0.61839	0.40916	0.4505
Lrguk	-2.06211	0.00178	2.17703	1.58707	1.81282	1.01197	1.15917	1.12987
Ccdc191	-2.06213	0.01098	3.40172	3.18709	3.12763	1.49848	1.76759	2.175
Prom1	-2.0664	0.00212	3.92586	4.00841	4.11152	1.91041	2.7473	2.46589
Rasef	-2.07734	0.00028	1.53304	2.11208	2.00129	1.14619	1.10465	1.03675
Tmem212	-2.07816	0.0009	10.7183	14.4354	14.9197	8.63145	10.3239	4.44791
Zc2hc1c	-2.07859	0.00045	1.16744	1.23658	1.63173	0.7955	0.72036	0.84214
Tcea3	-2.07963	0.00274	6.22911	7.16868	6.45647	3.90502	4.43031	3.2858
Mmp15	-2.08127	3.1E-05	11.7149	13.9607	17.3725	7.95699	9.47728	7.2996
Gsta4	-2.08246	0.00211	9.7127	14.6125	23.0423	10.4517	10.9394	5.77647
Cry2	-2.08437	0.00014	8.25161	7.9637	7.82726	3.97415	5.18931	4.68448
Abca8a	-2.08534	0.00054	6.09007	7.36787	9.47246	5.50786	5.17484	2.74569
Glul	-2.0901	0.00067	27.8445	35.7917	49.2842	23.6529	24.0602	16.6829
Rgs9	-2.09318	0.00104	2.00618	1.64093	2.17328	1.17794	1.36954	0.83065
Irx2	-2.10277	2.8E-06	5.15487	4.63255	6.68502	3.30738	3.10658	2.98294
Wfdc1	-2.10524	0.00837	2.09661	2.82262	4.05794	1.90487	1.93228	1.07299
Kif9	-2.10752	0.00019	1.94787	2.17824	2.17956	1.27199	1.48264	0.87819
Drc7	-2.10929	0.00409	4.34871	4.63718	4.51018	2.65168	2.36014	2.68431
Pxylp1	-2.11222	0.00046	1.67707	1.54199	1.72898	1.00385	1.11492	0.72052
Bik	-2.11317	0.02484	2.58941	4.44774	4.09559	1.59641	2.70302	2.12639
Enkur	-2.11386	0.02233	3.07266	4.12628	3.36905	2.94393	1.85061	1.28455
Sult5a1	-2.11401	0.02531	0.9078	0.77964	1.11573	0.78892	0.61527	0.53384
Dmd	-2.11551	0.00026	1.30073	1.83772	2.01627	1.06193	1.12449	0.75327

Cldn3	-2.11613	6.7E-05	18.7715	25.0449	34.0702	17.0548	14.577	12.1793
Lca5	-2.11642	0.00052	2.06057	2.37541	1.94161	1.39467	1.30695	0.95394
Dnaic2	-2.12024	0.01936	2.84456	2.88569	2.33856	1.37026	1.674	1.5751
Slc4a4	-2.12412	8.8E-05	1.42725	1.62744	1.99829	0.88646	1.17058	0.80707
Mecom	-2.1272	2.1E-06	4.24418	4.19002	4.47286	3.00996	2.56934	2.05095
4430402I18Rik	-2.12799	0.01219	1.07803	1.03639	0.83386	0.61398	0.56434	0.60935
Cfap43	-2.12821	0.01282	8.50614	7.8997	8.4113	4.22625	4.40401	5.1778
Ccdc171	-2.12907	0.00174	2.50037	2.22231	2.43821	1.05661	1.47297	1.51397
Eya1	-2.13404	0.03925	0.22338	0.27818	0.27363	0.13192	0.16323	0.15107
Grhl2	-2.13479	0.00023	3.23108	3.90399	3.6863	1.71243	2.54617	1.82091
Tmem147os	-2.13732	0.0437	3.01778	2.59176	2.55341	1.51079	2.04012	1.58269
Tmem107	-2.1379	0.00297	1.91105	2.35249	2.76112	1.4469	1.61365	0.86159
Dusp18	-2.13818	0.00057	3.99137	4.46276	4.59475	2.46318	2.93488	2.0117
Per3	-2.13845	0.00031	10.91	9.08925	9.921	6.91056	8.13569	4.61593
Foxq1	-2.13902	0.00665	1.97228	2.65371	3.12834	1.07515	1.44117	1.42271
Klf5	-2.13959	0.00208	7.49955	6.70197	9.072	4.23554	4.39389	4.38636
Ccdc181	-2.14545	0.00156	9.91307	9.6122	9.42689	6.39171	5.47443	4.57226
Ccdc157	-2.14584	0.00398	6.21017	6.84777	6.51328	3.40235	3.89175	3.65104
Prdx6	-2.1463	9.5E-05	40.0523	56.3102	83.382	39.0658	37.865	23.4233
Myh14	-2.14632	4.9E-05	43.2261	48.5958	47.9165	24.8057	24.0771	28.0892
Itih2	-2.14746	0.00393	0.86813	1.0167	0.86759	0.59753	0.5895	0.35581
Tox3	-2.14773	0.0188	0.93548	1.4283	1.46913	0.84993	0.7342	0.56234
Rap1gap	-2.14832	0.00185	3.33692	2.93575	3.4127	1.72539	1.65199	1.99981
Epb41l4b	-2.14941	8.4E-06	2.00839	2.29983	2.96855	1.58321	1.52511	0.98537
Tmod1	-2.15064	5.9E-05	3.86754	2.96094	3.56098	2.6906	2.30391	1.91306
Pacrg	-2.15392	0.01782	2.10807	2.14399	2.97956	1.36086	1.83766	0.82536
Acsf2	-2.1593	6.9E-07	5.74269	5.25935	6.00976	4.04011	3.56496	2.40578
Rassf9	-2.16064	3.8E-06	8.11138	7.46138	10.0874	5.3869	5.33538	3.61986
Wdr54	-2.16195	0.03866	0.69107	0.70765	0.73883	0.4709	0.33665	0.44937
Ttc12	-2.16252	0.00043	3.31945	3.14747	2.82047	1.91761	1.79464	1.53117
Armc4	-2.16358	0.00571	1.95634	1.594	1.72524	0.7292	1.12407	1.11947
Calcoco1	-2.17073	3.7E-08	11.5529	9.69518	11.2592	6.2778	6.49618	5.38715
Tnk1	-2.17304	0.0034	1.80755	1.66487	1.72788	0.83302	1.07195	1.02752
Gm4956	-2.17507	0.00208	6.96383	5.26746	9.69223	3.07642	4.79289	4.14776
Slc23a2	-2.17869	2.5E-06	18.24	21.0909	19.6157	10.9416	12.9515	9.04926
Map7	-2.18246	0.01129	4.79822	4.69321	4.88543	2.434	2.46727	2.84829
Mturn	-2.18369	0.00029	2.5879	2.9722	3.46296	1.50256	2.05885	1.34625
Lamc3	-2.18683	0.00051	2.51987	3.09866	3.58859	2.2504	1.82735	1.00698
Rsph1	-2.18736	0.00179	10.339	12.3541	11.7189	8.10021	6.27748	4.76266
Foxj1	-2.18879	0.00721	27.562	31.7804	32.4558	14.5294	15.5159	19.1565
Naaladl2	-2.18893	0.00036	0.75388	0.67624	0.85244	0.44902	0.45702	0.33989
Rab6b	-2.19116	0.00037	2.73878	3.98973	5.27568	2.75604	2.31369	1.50048
Wnt11	-2.19261	0.00478	2.33987	2.57078	2.34383	1.11082	1.17065	1.65366
Strbp	-2.19396	3.1E-06	2.74515	2.86857	3.03691	1.81737	1.79368	1.22919
Tyro3	-2.19684	0.00095	1.95577	2.38055	2.43948	1.38062	1.12409	1.19893
Traf3ip1	-2.19693	0.00698	16.6333	16.5652	15.6731	7.98299	8.58347	9.88228
Ccdc65	-2.19862	0.00248	1.79952	2.81358	3.08693	1.80264	1.21378	1.18576
Tcte1	-2.20073	0.00269	2.95042	2.82629	3.42166	1.72692	1.63075	1.5732
Megf6	-2.21034	0.00019	9.73156	9.52729	10.6094	5.00923	6.20221	5.04631
Tnfaip8l3	-2.21212	0.04261	0.32334	0.35937	0.41221	0.29377	0.20384	0.07718
Fam198a	-2.21222	0.00192	1.41929	1.97351	2.22893	1.28949	1.05356	0.67035
Mfsd6l	-2.21279	0.00514	1.27001	1.30887	1.39615	0.84617	0.98988	0.28629
Col8a2	-2.21299	0.0038	2.99175	4.02096	3.36821	1.65913	1.72239	2.20716
Nectin4	-2.21384	1.8E-05	2.86346	2.95908	3.15882	1.58634	1.79184	1.54288
Wnk4	-2.21398	0.00656	1.80807	2.00423	2.04046	1.08878	0.9832	1.08056
Cyb5a	-2.21597	0.00019	13.0628	15.7972	24.8092	13.0513	9.07931	6.82503

1700016K19Rik	-2.21786	0.01274	7.38566	6.42436	8.92217	3.95731	4.24363	4.09822
Erich3	-2.22025	0.04782	3.3373	3.45947	3.27299	1.45859	1.88642	2.01346
Olfml2a	-2.22219	3.6E-06	3.59522	3.93345	4.53654	2.64155	2.57377	1.39538
Iyd	-2.22787	0.00991	1.23584	1.33826	1.72144	0.92755	0.73291	0.65408
Shh	-2.23368	0.005	3.33882	3.57778	3.95424	2.08726	1.99953	1.3672
Abca6	-2.2354	0.00849	0.88855	0.666	0.91337	0.57245	0.4722	0.28406
Pih1d2	-2.23638	0.003	1.70509	1.67739	1.81114	0.92949	0.87593	1.00634
Spag16	-2.24068	8.4E-05	2.06286	2.31422	2.1503	1.25338	1.39431	0.90679
Adamdec1	-2.24105	0.02548	1.5104	2.1002	1.86372	0.52277	1.33141	1.11872
Pcolce2	-2.24177	0.04291	3.97855	6.41278	9.16787	4.5398	3.94972	1.98924
Col4a6	-2.24224	2.9E-06	2.53758	2.74299	2.92703	1.46082	1.59849	1.36104
Dzank1	-2.24232	0.00255	1.5094	1.99434	1.63113	0.63294	0.91526	0.65672
Unc79	-2.24237	0.0073	0.62514	0.66366	0.68723	0.29188	0.47372	0.30533
Efcab6	-2.24327	0.02509	1.53784	1.39087	1.25671	0.56877	0.5834	1.0676
Usp2	-2.24595	0.01177	2.73316	3.09349	3.15526	1.46361	1.39316	1.34648
Acss2	-2.24623	0.00059	1.67327	1.73184	2.13458	0.86537	0.99487	0.80081
Nrxn2	-2.24654	0.00227	0.33464	0.42838	0.32722	0.22372	0.15379	0.16226
Kcna2	-2.24756	0.01053	0.29299	0.42777	0.49003	0.25954	0.25691	0.12879
Ppl	-2.25238	0.00039	9.53073	10.8342	12.5806	5.68019	5.10304	6.38898
Rab36	-2.25469	0.01627	2.47147	2.32877	2.56833	1.14197	1.34843	1.41351
Ehf	-2.25788	1.1E-06	2.28341	2.43119	3.22739	1.59147	1.24948	1.37498
Otud1	-2.26645	1.8E-05	4.06476	4.78683	5.80319	2.54595	2.76016	2.45733
Agbl2	-2.26667	0.00013	1.2196	1.43036	1.37161	0.83402	0.81771	0.49664
Ccdc189	-2.27273	0.03018	1.83273	2.27357	2.4225	0.90194	1.2648	1.61814
Aldh1a1	-2.27395	0.00152	75.7385	122.25	180.277	79.8884	80.4539	40.1599
Lrrc10b	-2.27566	0.0188	6.38207	6.85141	5.49932	2.63218	3.31351	3.91848
Iqck	-2.28043	0.00042	0.58383	0.68798	0.58851	0.3454	0.34393	0.22037
Slc44a4	-2.28058	0.00111	2.15777	2.71958	2.93099	1.14571	1.50163	1.47819
Usp43	-2.28533	0.00021	1.89302	2.46236	2.16473	1.11877	1.28925	1.14347
Sox6	-2.28879	0.00044	0.45743	0.5084	0.68457	0.31781	0.30148	0.24566
Fam161b	-2.29349	0.0044	1.70444	1.75338	1.39426	0.79981	0.89418	0.88667
Ddo	-2.29358	0.0009	2.45256	2.03081	1.97952	1.05643	1.1995	1.163
Maats1	-2.29578	0.00013	2.15422	2.41176	2.37422	1.39801	1.19933	1.0406
Sec14l3	-2.29791	1.3E-05	174.572	190.766	224.943	115.262	116.064	80.2022
Magix	-2.29875	0.00254	1.01892	1.33836	1.24248	0.81683	0.72994	0.32427
Tsga10	-2.29946	0.00025	1.84852	1.69294	1.48747	0.88432	1.10768	0.70802
Mgst1	-2.30126	8E-06	26.6865	32.1836	44.3727	21.0971	21.2729	11.9534
Ttc29	-2.30153	0.01045	2.21652	2.4747	2.18138	1.07403	1.27171	1.26579
Thbs3	-2.30456	5.1E-07	5.78612	6.00975	6.40621	3.15418	3.98135	2.76354
Myom3	-2.30491	0.0114	0.34664	0.42174	0.38106	0.24932	0.21108	0.13674
Wdr63	-2.30622	0.00104	4.60928	3.91336	3.87165	2.29728	2.1299	2.10183
Fkbp4	-2.30678	7.2E-05	12.2309	19.4544	23.9317	9.72332	10.1836	8.60901
C530008M17Rik	-2.31221	0.01313	0.18873	0.25285	0.3201	0.14403	0.11032	0.16337
Cyp1b1	-2.31412	0.00029	1.36049	1.25607	1.81076	1.06611	0.69589	0.52904
Eno4	-2.31469	0.0001	3.42722	3.73026	4.02873	1.57231	2.21504	2.0653
Lekr1	-2.31605	0.01082	0.74564	0.70999	0.51931	0.324	0.41061	0.35079
Gpr179	-2.31952	0.01541	0.28332	0.19623	0.24974	0.11625	0.14247	0.12361
A530020G20Rik	-2.31962	0.00265	2.7274	3.13641	2.87487	1.65899	1.44123	1.34427
Nr3c2	-2.32181	3.8E-06	1.96668	1.90886	2.4624	1.06475	1.41739	0.74699
Hmgcs2	-2.32191	0.00482	1.5374	1.93502	2.52264	1.20414	1.21378	0.68118
Aox1	-2.32242	0.00022	5.77035	6.19686	6.25046	3.1547	3.44162	2.87754
Gm15411	-2.32249	0.02315	0.47504	0.77302	0.68321	0.40888	0.34103	0.30435
Jdp2	-2.32463	0.00012	4.17261	5.017	6.24709	2.86433	2.39401	2.6964
Enah	-2.32477	2.7E-06	4.33496	5.12595	5.92941	2.44858	2.5762	2.78545
Rph3al	-2.32488	4.9E-05	1.60056	2.10119	2.49924	1.1218	1.0693	1.02056
Ccdc85c	-2.32612	0.00098	4.39702	3.53423	4.56813	1.79259	2.36166	2.36361

Mdm1	-2.32692	0.00049	2.46474	2.90168	2.84436	1.50967	1.21417	1.53573
Pcyt1b	-2.33043	0.03279	0.56977	0.46138	0.55222	0.35497	0.28549	0.17615
Kif6	-2.33346	0.00579	1.55132	1.34478	1.66669	0.59276	0.84753	0.90204
Per2	-2.33746	3E-05	16.545	14.2861	17.0072	6.53504	8.85051	8.90279
Plekhd1	-2.33824	0.0039	4.5386	4.22114	4.46435	2.25286	2.35115	2.2159
Dpm1	-2.33899	0.04075	1.14119	1.49013	1.48129	1.39654	1.56565	1.134
Cdkl4	-2.33902	0.00089	2.1125	2.32535	1.90644	0.91911	1.27549	1.1268
Slc9a2	-2.34141	0.03616	0.16233	0.2681	0.35298	0.11345	0.15815	0.13511
Gm26671	-2.34268	0.01896	1.6802	1.30336	1.53213	0.88638	0.8449	0.58646
Lrrc45	-2.34387	0.01237	15.3865	13.783	11.6036	6.03927	6.45042	8.23836
Ccdc81	-2.34395	0.02132	1.06274	2.25495	1.41373	0.65317	0.79179	1.01464
Hspa2	-2.34426	1E-06	4.01432	5.55781	5.83691	2.86117	3.30419	1.77864
Slc16a7	-2.3454	0.00011	1.42166	2.21419	2.10309	1.1578	1.28463	0.55793
Ppargc1a	-2.34835	0.00598	0.59532	0.74988	0.84766	0.3726	0.41245	0.33997
Ppp2r2c	-2.35295	0.02731	0.19597	0.42077	0.68324	0.28488	0.22912	0.13253
Cfap54	-2.35448	0.00172	3.97	3.82801	3.69547	1.86905	1.89293	2.07461
Arhgdig	-2.35515	0.00135	3.40571	3.75405	3.15856	1.53494	1.61987	1.88607
Rassf10	-2.35572	0.0172	0.89802	1.04975	1.03422	0.45327	0.38886	0.70852
Plin5	-2.35574	0.01852	1.2118	1.72259	1.22059	0.53151	0.85494	0.79124
Fign	-2.35583	3.9E-06	0.75232	0.80389	0.94793	0.52456	0.46876	0.28396
Dync2h1	-2.3563	1.1E-06	4.35825	4.531	4.67465	2.42815	2.54146	2.00579
Macrod1	-2.35744	0.00493	1.77975	2.5867	3.09601	0.87068	1.5339	1.48134
Gm10044	-2.36157	0.00422	1.09258	1.16582	1.21669	0.63169	0.77417	0.44781
Cacnb1	-2.36843	0.00027	2.17586	1.78601	2.28612	1.04967	0.95677	1.17197
Scube3	-2.36871	2.7E-05	1.07751	1.61728	1.80288	0.80513	0.89282	0.58567
Tmem232	-2.369	0.03674	2.02277	2.04544	1.99823	0.74104	1.20788	1.14731
Vwa3b	-2.37016	0.02365	1.40866	1.47402	0.99169	0.55901	0.78876	0.65699
Wdr78	-2.37087	1.1E-05	3.42529	3.5055	3.57617	1.68072	1.72093	1.92093
Mok	-2.37098	0.00259	2.12577	2.2436	2.24438	1.02385	1.17279	1.17779
Kyat1	-2.37161	1.5E-05	1.73043	2.15235	2.10845	1.11137	0.95923	1.00883
D830026I12Rik	-2.37384	0.00522	1.23874	1.1348	1.24505	0.67528	0.80459	0.33509
Cyp39a1	-2.37408	0.00016	2.99928	2.79667	4.01042	1.79071	2.44904	0.77269
Mdh1b	-2.37444	0.01839	1.11609	1.65566	1.43409	0.5531	0.98854	0.61755
Agmo	-2.37686	2.5E-05	1.04066	1.13209	1.37282	0.69336	0.7976	0.30028
Nme9	-2.37719	0.02574	5.56198	5.81328	4.35093	2.38364	2.81171	2.83879
1810019D21Rik	-2.38069	0.00712	4.55436	4.00801	4.76827	3.21833	1.86254	2.35752
Adcyap1r1	-2.3819	0.00785	0.33028	0.40847	0.39836	0.16804	0.20594	0.21443
Ankrd42	-2.38226	0.01251	3.35217	3.15803	2.85245	1.72481	1.61632	1.5036
Smim5	-2.3828	0.01713	2.8558	3.36104	4.28557	2.25829	2.42168	1.7167
Snhg11	-2.38296	6.7E-08	1.96501	1.94251	2.26625	1.08792	1.18658	0.85823
Spata24	-2.38639	0.04977	3.06624	4.18244	4.07892	2.70389	1.66942	1.34175
Fsd2	-2.38857	0.02275	1.00666	1.10888	1.27848	0.43742	0.74624	0.53278
1700040L02Rik	-2.38973	0.01481	2.4877	1.48369	2.14875	1.06731	1.00988	1.02811
Cfap77	-2.39048	0.00093	1.89437	2.39761	2.44265	1.238	1.1333	1.03388
4930412C18Rik	-2.39116	0.01868	0.45391	0.32892	0.37424	0.2062	0.2073	0.17267
Spata18	-2.39309	4E-05	14.1138	15.996	15.171	7.97211	8.30696	6.66173
Dnah7a	-2.39559	0.03446	1.56423	1.56634	1.55117	0.70749	0.81191	0.81302
Aldh1a7	-2.39712	0.00711	10.0462	14.6458	24.5406	11.812	9.70558	3.42551
Frem2	-2.40849	0.02028	0.44739	0.50854	0.52695	0.20324	0.26922	0.26696
Htr1b	-2.41077	0.00514	0.73842	0.68174	0.74798	0.31867	0.35972	0.07491
Tmem139	-2.41138	0.02548	1.14047	1.82834	1.7194	0.89801	0.74076	0.71985
Fam187b	-2.41877	0.0051	0.60962	0.90934	1.14885	0.46642	0.43764	0.43397
Fgfr3	-2.42317	2.1E-05	11.8299	12.6711	15.7734	6.20974	7.03886	6.36063
Wdr60	-2.42581	0.01081	6.74249	5.5695	5.31644	2.61626	2.4406	3.57814
Syt10	-2.42652	0.0076	0.99261	1.13664	2.04876	0.773	0.64472	0.63931
Ascc2	-2.43166	1.8E-06	14.599	15.8575	16.4711	7.82108	8.52388	6.93834

Map3k19	-2.43195	0.002	3.00038	3.17502	2.50744	1.34677	1.42681	1.54596
B330016D10Rik	-2.43266	0.00595	1.9482	1.49705	1.59419	1.11792	0.74925	0.62408
Ltbp4	-2.43305	0.00026	38.7394	55.4859	68.4289	25.6711	30.3155	23.3422
Gm9899	-2.43399	0.02293	0.34355	0.41928	0.44298	0.19714	0.26425	0.14674
Saxo2	-2.43485	0.00025	2.07811	1.98827	1.94096	1.0434	1.36754	0.61639
Armc2	-2.43495	0.0135	1.39522	1.28162	1.31468	0.65035	0.59102	0.72202
Fam227a	-2.43655	0.00256	1.767	1.48333	1.38958	0.72424	0.81548	0.77269
Tnxb	-2.43708	5.5E-06	12.2715	14.1953	17.3581	6.49615	7.95437	6.9463
Ngef	-2.43775	8.2E-06	3.64016	3.85512	3.85088	1.8261	1.9147	1.6915
Fam167a	-2.44397	0.00046	5.63156	6.13333	7.22909	3.13296	3.33975	2.84255
4933408B17Rik	-2.44597	0.04236	0.78824	0.70773	0.54017	0.22787	0.41889	0.38768
Ccdc121	-2.44602	0.04178	1.10268	2.38813	2.03287	0.82761	0.79406	1.1672
Homer2	-2.4472	0.00124	0.42149	0.59343	0.86594	0.27622	0.28948	0.34579
Nek10	-2.44925	0.02848	1.02202	0.91705	0.84804	0.37419	0.40127	0.59832
Fsd1l	-2.4519	2.4E-06	1.78194	2.02257	1.8647	1.10644	1.17777	0.55712
Dnah5	-2.45472	0.00059	9.55211	10.0869	9.91021	4.0706	5.11529	5.12408
Crnde	-2.45499	0.00094	1.72945	1.85664	1.9148	0.6678	1.09523	0.99414
Akr1c14	-2.45767	0.00091	4.57598	5.34077	7.32456	3.35798	3.34372	1.78535
1700001C02Rik	-2.46158	0.02169	1.84833	1.87602	2.05829	1.22131	0.9004	0.6818
Gm20219	-2.46595	0.0395	2.19909	1.65257	2.59017	0.6868	1.27213	1.20833
Tst	-2.46845	0.00021	8.83216	12.3006	18.0691	6.72318	8.83759	3.55147
Fam46c	-2.4743	3.8E-06	6.45082	7.78817	9.15132	3.80762	4.84956	2.80515
Lrrc48	-2.47627	0.0004	4.32275	4.24288	3.855	1.85851	2.2186	1.9837
Bmx	-2.47661	0.00041	1.77687	1.45336	1.91348	1.23	0.68697	0.57221
Rims2	-2.47967	0.02706	0.25924	0.43044	0.45598	0.17272	0.20206	0.187
Ccser1	-2.48299	0.0002	2.05537	2.21042	1.86816	1.03285	1.04556	0.91026
March4	-2.49029	0.0012	1.87708	1.82595	1.9853	0.7657	1.06369	0.93263
Gstm1	-2.49401	0.00037	29.1876	35.084	63.5964	23.8826	23.7071	13.7731
1700088E04Rik	-2.49898	0.00015	1.54904	1.59927	1.64595	0.98816	0.8991	0.69095
Stmnd1	-2.50108	0.02276	2.81521	3.46184	3.73789	1.16262	1.74543	1.90385
Spag6l	-2.50114	1.8E-05	2.62063	3.15424	3.65895	1.58152	1.80776	1.16425
Dnaja4	-2.5056	0.00016	6.00175	7.22047	7.67011	2.95824	3.64575	4.2067
Catip	-2.50608	0.03684	5.0881	3.69233	4.57137	1.57676	1.4987	3.14752
Cfap70	-2.50668	0.00096	2.44545	2.46473	2.78037	1.2119	1.12236	1.33943
Syde2	-2.51212	1.7E-07	2.22909	2.4093	3.25778	1.35016	1.31488	1.12804
Cfap206	-2.51306	4.5E-06	4.91508	5.15593	6.18615	2.61556	2.59957	2.56417
D130043K22Rik	-2.51448	0.00058	0.77687	0.76657	0.90334	0.52561	0.43481	0.20121
Esrrg	-2.51521	0.01064	0.47762	0.36194	0.29121	0.12763	0.2053	0.228
Ppp2r2b	-2.51612	0.01066	0.50333	0.75179	0.7011	0.31812	0.27718	0.35519
Cetn4	-2.52387	0.0064	1.31936	1.17026	1.18206	0.66813	0.84291	0.61433
Rgs11	-2.52512	0.01188	3.81507	3.70388	3.51669	1.46936	1.77768	2.32763
Dnah7c	-2.52613	0.01176	1.88905	1.86009	1.71479	0.92416	0.82922	0.9359
Dnah11	-2.52628	0.00019	4.68067	4.61564	4.1547	1.79202	2.35655	2.27615
Cdkn1c	-2.52696	0.00027	5.47453	6.28118	7.93925	3.34182	2.6252	3.06594
Ptgfr	-2.53184	0.03026	0.74624	0.67405	1.11536	0.36238	0.63931	0.19142
Ccdc160	-2.53757	0.01323	1.13552	1.02398	0.90947	0.619	0.44253	0.38396
Ankrd45	-2.53778	0.00249	1.14409	1.46384	1.05604	0.50912	0.6597	0.56844
Ccdc153	-2.54045	0.00196	15.634	16.8481	16.023	7.1994	7.92895	7.82097
Mtus2	-2.54096	0.00012	1.10935	1.39888	1.32738	0.58394	0.60046	0.63114
Nphp4	-2.54101	0.00105	1.01002	1.02853	1.14205	0.40639	0.56231	0.54643
Ggnbp1	-2.54119	0.00737	0.76619	0.62567	1.08886	0.45647	0.47726	0.56062
Syngap1	-2.5413	0.00621	7.07992	5.81561	5.30306	2.3465	2.8042	3.38892
Pitpnm3	-2.54276	8.7E-05	4.06939	4.23512	4.01866	1.97778	2.20744	1.70649
Nthl1	-2.54416	0.02495	2.66468	1.45632	2.2065	0.807	0.94405	1.31058
Grb7	-2.54426	2.7E-08	2.69527	3.27186	3.36996	1.67177	1.43923	1.332
Ccdc173	-2.54595	0.02446	2.18189	1.87388	1.4777	0.68143	0.8636	1.10666

Tekt4	-2.54919	0.00129	3.92776	2.73774	3.48646	2.12045	1.3865	1.30887
Gfra1	-2.55025	0.00047	2.70732	3.24151	3.64647	1.47584	1.76881	1.33547
Rsph4a	-2.55189	8.3E-06	5.66252	6.03277	5.47071	3.27783	2.88648	2.0682
Ift81	-2.55358	2.6E-05	6.25593	6.59779	6.72005	3.1602	3.46198	2.6738
Hspa4l	-2.55383	2.7E-08	5.56141	7.17718	7.2473	3.51412	3.30003	2.63071
Zdhhc1	-2.55548	5.8E-05	13.0016	12.1958	12.1147	5.38982	5.73951	6.31835
Wdr72	-2.5584	0.02913	0.45433	0.46023	0.4281	0.17993	0.23835	0.22059
Dapk2	-2.5595	5.2E-06	5.09042	5.26751	6.08344	3.04566	2.75797	1.98154
Ptpn13	-2.56546	1.1E-09	7.96989	8.25584	8.82028	4.17701	4.41604	3.23583
Vtcn1	-2.56593	0.04911	0.76498	1.02833	1.81771	0.36262	0.81012	0.49484
Adgb	-2.56804	0.00017	2.8872	2.85459	3.2779	1.36916	1.59172	1.27629
Frmpd2	-2.57082	0.01129	3.12907	2.78041	2.62934	1.11993	1.24056	1.59394
Plin4	-2.57097	0.03379	1.49447	2.51514	3.15777	1.11093	1.27955	0.91879
Fmo2	-2.5712	1.4E-05	42.6943	50.5676	73.9837	26.5285	33.068	18.7579
Lrrc46	-2.57199	0.01106	1.37078	2.01817	1.96822	0.88958	0.76316	0.88287
Rorc	-2.5746	0.00021	2.35233	2.73576	2.63213	1.29123	1.71887	0.69598
1700003E16Rik	-2.57638	0.03279	2.86635	3.27104	2.2569	1.14157	1.26242	1.54012
Morn3	-2.58556	0.03127	3.09384	2.82316	2.6723	1.17121	1.50714	1.74356
Dlec1	-2.58807	0.00884	3.55551	3.54574	3.08027	1.05312	1.57342	1.97291
Wdr31	-2.58846	0.00224	1.32523	1.644	1.12732	0.60202	0.8249	0.74685
Ccdc96	-2.58904	0.00169	1.72712	2.02649	1.67326	0.77354	0.8532	0.92124
Arhgef38	-2.59134	6.9E-09	2.1722	2.48741	2.55851	1.08352	1.29678	0.99373
Gsn	-2.59318	0.00063	111.24	174.182	224.031	93.8891	88.1407	54.4738
Myo5c	-2.59969	4.2E-05	11.9161	11.74	13.1772	5.87481	5.74786	5.36421
Znrd1as	-2.60129	0.01611	0.76364	0.83073	0.86347	0.74006	0.1984	0.13771
1700013F07Rik	-2.60261	0.0051	1.68169	1.28954	1.30164	0.54568	0.87774	0.56864
BC039771	-2.60548	6.1E-05	1.40628	1.90698	1.86559	0.97506	0.9735	0.45048
Ccdc40	-2.6058	0.00032	4.72838	5.01712	4.91868	2.22024	2.04276	2.4447
Efcab2	-2.60705	0.00123	1.32356	1.38931	1.40552	0.43901	0.7164	0.76721
Lrrc6	-2.60859	0.00214	2.55262	3.19856	2.64185	1.29266	1.67157	0.96218
Tmem88b	-2.60873	0.02792	0.45224	0.43156	0.40246	0.22827	0.19583	0.16991
Ccdc113	-2.60893	5.1E-07	4.80725	6.86165	6.6989	3.1373	3.16639	2.19786
Dnah6	-2.61079	0.00017	11.6852	12.6138	13.5291	5.35911	5.8623	5.90338
Rnf32	-2.61296	0.00059	0.8915	0.90237	0.96004	0.52069	0.43429	0.30145
Selenbp1	-2.62283	0.00011	71.2854	82.1129	123.025	52.0082	42.6133	29.0599
P4htm	-2.62304	0.003	1.32269	1.00119	1.3097	0.67215	0.54605	0.45483
Lhfpl4	-2.62333	0.01427	0.34505	0.48961	0.3534	0.1363	0.23386	0.18262
Cyp4b1	-2.62393	1.2E-05	37.6121	45.8477	59.2623	24.6196	23.0867	17.4627
Cntfr	-2.6256	0.0012	1.53601	1.37302	1.6541	0.45569	0.82461	0.84796
Rhov	-2.62655	0.04456	0.66571	1.18744	0.67553	0.3722	0.34924	0.48483
Dnah9	-2.62673	0.00078	6.38651	6.33962	5.99722	2.59819	2.6743	3.15791
Fank1	-2.637	0.00011	2.57943	2.47592	2.39478	1.20623	1.10872	1.12873
Ttc21a	-2.64142	0.00486	14.2756	11.5931	11.7129	4.23513	5.12502	6.67693
Gabrp	-2.64247	0.00084	8.44444	10.5791	14.3566	6.31137	6.28997	2.82917
Cys1	-2.64498	2E-05	4.21866	4.33076	4.81362	1.73676	2.11931	2.22887
Dnah3	-2.64586	0.01747	9.68093	9.16248	8.34657	3.11833	4.15767	5.16993
Mycbpap	-2.64878	0.00037	3.35674	3.85028	3.41769	1.37137	1.58033	1.85871
Dnah12	-2.65305	0.00991	10.5463	9.20601	8.07144	3.80561	4.16517	4.60243
Por	-2.65395	6.6E-08	27.9776	31.5903	37.7658	15.4484	16.9676	11.8509
Ulk4	-2.65433	0.0025	3.1076	3.25906	2.90887	1.24863	1.25904	1.63687
Adgrv1	-2.65697	0.01077	0.5175	0.50189	0.5208	0.17272	0.22294	0.29997
Ccdc162	-2.66048	0.00878	5.07489	4.74132	4.55576	1.77578	2.21328	2.43487
Ppp1r32	-2.66114	0.03533	1.49533	1.13013	1.23993	0.43475	0.6993	0.6472
Osbpl6	-2.6631	1.9E-09	6.36963	6.78781	6.81851	3.2833	3.46175	2.40288
Gm28729	-2.66497	6.3E-05	1.82053	2.40249	2.13383	0.84719	1.16805	0.90085
Lrrc74b	-2.66536	0.00012	3.3635	3.24977	3.31529	1.34701	1.59538	1.55633

Vnn3	-2.66801	0.00503	0.96309	1.36477	1.54273	0.74376	0.70374	0.26052
Ccdc148	-2.66842	0.00694	0.40435	0.28266	0.35442	0.16233	0.14659	0.16534
Irx5	-2.66871	2.7E-07	2.78886	3.80831	3.75784	1.69765	1.902	1.09389
Ccdc87	-2.67474	0.04109	0.386	0.5683	0.36371	0.1503	0.26862	0.18646
2010300C02Rik	-2.67534	1.9E-05	5.67064	5.8294	5.79665	2.32623	2.59499	2.78901
Gpr88	-2.68178	0.0188	0.37332	0.49162	0.44557	0.1809	0.24248	0.16831
Galnt13	-2.68588	0.00174	0.22332	0.23975	0.39982	0.15217	0.17406	0.04531
BC051019	-2.68648	2.4E-05	5.09764	5.68057	6.11337	2.56452	2.75007	2.27926
Zbbx	-2.68816	0.00116	3.0468	2.59201	2.84383	1.14905	1.26019	1.39956
Gm15728	-2.68832	0.04193	0.31837	0.31549	0.25383	0.24475	0.09544	0.03312
Gm7173	-2.694	1.2E-05	1.19657	1.18751	1.31123	0.63618	0.56994	0.45555
Myo1h	-2.69663	0.00187	1.82449	1.89548	1.46961	0.64167	1.03214	0.71643
Flt3l_1	-2.69967	0.04952	4.74775	4.46992	4.6047	1.89508	2.57411	2.01512
Ccdc114	-2.70127	0.02719	3.81157	3.48271	2.91643	1.15867	1.32627	1.97685
Tppp3	-2.70191	3.6E-09	35.4871	45.4325	43.3099	19.0792	19.5401	16.7226
Iqcg	-2.70199	2.7E-05	3.47621	3.85084	4.05882	1.60204	1.93883	1.70352
Nek5	-2.70364	0.00045	4.96695	3.49018	4.25474	1.75039	1.93569	1.99504
Rd3	-2.70371	0.02339	0.28586	0.36826	0.57719	0.16696	0.3183	0.08285
Grip1	-2.70563	0.00045	0.80994	0.60032	0.51228	0.22177	0.31348	0.30013
Ccdc67	-2.71796	0.00257	1.15891	1.06317	1.16646	0.52646	0.59021	0.39187
Dixdc1	-2.71933	3.4E-08	2.3702	2.85435	2.69488	1.1681	1.45713	0.9227
Acsm1	-2.71989	0.03599	1.35949	1.68269	1.84617	0.58125	0.85705	0.54082
Mak	-2.73043	3.4E-05	3.08223	3.13032	2.4433	1.37795	1.45382	1.05875
Fads6	-2.73336	0.00021	0.43256	0.59665	0.62992	0.262	0.33204	0.14183
Cyp2s1	-2.73808	1.1E-07	20.4862	23.1606	23.1809	9.4668	10.9432	8.98135
Rsph10b	-2.74093	0.00421	6.80562	6.01396	5.22029	2.2229	3.04131	2.51042
Fbxo31	-2.74416	2.4E-05	3.977	4.57687	4.60182	1.98008	1.99936	2.04404
Spef2	-2.75189	3.4E-05	3.25402	3.28561	3.26505	1.28645	1.4395	1.53447
Tnn	-2.75915	0.0208	0.56969	0.6824	0.84758	0.38138	0.35055	0.18249
Ak7	-2.75963	9E-07	7.72547	7.86984	8.79384	3.71441	3.65352	3.27962
Spag17	-2.7607	6.3E-05	3.36955	3.36148	3.32991	1.45603	1.58139	1.36168
Rac3	-2.76326	2.5E-06	4.04676	5.96625	6.29169	3.06389	2.36561	1.64202
Dnali1	-2.76519	1.5E-06	6.21451	6.77687	6.7033	2.56307	3.42568	2.65433
Pcsk4	-2.76747	0.03658	1.12031	0.65832	0.63894	0.29464	0.34469	0.43863
Katnal2	-2.77068	0.00559	0.49391	0.5164	0.58681	0.29266	0.27199	0.11618
Sec14l4	-2.77143	1.1E-09	8.12804	8.119	9.61476	4.29449	4.06915	2.94288
Fsip1	-2.77208	0.00466	1.54545	1.66858	1.83069	0.76223	1.03248	0.35833
Ankrd66	-2.77335	0.00235	2.90472	2.21118	1.80395	0.89969	1.35069	0.80361
Morn2	-2.77369	0.00657	2.43748	3.95417	2.29677	1.84547	1.31933	0.54947
Cfap45	-2.77847	0.00024	3.44774	4.61573	3.24871	1.75048	1.38315	1.69156
2010310C07Rik	-2.77868	0.02481	0.61824	0.68713	0.85669	0.42953	0.31888	0.14756
Nov	-2.78455	0.00121	1.15507	2.3495	3.06467	0.9942	0.88844	0.90448
Rasgrf1	-2.78467	0.02475	0.52027	0.56868	0.60908	0.27931	0.16896	0.27721
Ccno	-2.79376	0.00389	0.79026	1.03801	1.22647	0.88693	0.18116	0.18862
1810010H24Rik	-2.79588	5.3E-05	8.17733	9.7788	10.2412	4.0439	3.7314	4.27007
Cfap100	-2.79659	0.01713	5.66998	4.77871	5.62175	1.69152	1.85513	3.11906
Trpm3	-2.80649	0.02063	0.4111	0.29732	0.27524	0.08846	0.2108	0.13169
Shank2	-2.80903	2.2E-05	3.3205	3.18561	3.40353	1.45691	1.45974	1.33638
Tbc1d30	-2.80932	1.8E-13	3.739	4.07977	4.15848	1.92129	2.14988	1.11921
Prob1	-2.80989	0.00067	6.54771	6.49677	6.94303	2.48006	2.92023	3.06462
1700007G11Rik	-2.81884	0.03832	2.2886	2.12069	2.15648	0.76606	0.82148	1.30334
Lrriq1	-2.82093	0.00112	3.77182	3.26635	3.53927	1.32792	1.28619	1.82805
Cfap58	-2.8301	8E-05	1.82497	2.32863	2.13724	0.94747	0.86362	0.88155
Ctf1	-2.83271	0.01346	0.81279	0.73911	0.90102	0.26063	0.46582	0.32333
Gmnc	-2.83476	0.00951	0.34174	0.22012	0.54339	0.15524	0.18728	0.11555
Amy1	-2.83536	0.00081	1.1135	0.71723	1.00153	0.39087	0.41915	0.41074

Vwa3a	-2.84092	0.00096	4.74765	4.89046	4.43303	1.53773	2.11015	2.231
Sh3bgr	-2.84232	0.01183	0.90488	0.82571	1.22567	0.41106	0.4959	0.30597
Cep126	-2.84386	1.7E-08	4.91204	5.80871	5.58977	2.16275	2.44808	2.29975
Gspt2	-2.8444	0.00186	0.41516	0.53483	0.67373	0.23051	0.29213	0.15598
Ttc25	-2.84442	0.0016	1.70244	1.87638	1.72447	0.77338	0.4976	0.94024
Hydin	-2.84741	1.6E-07	4.37561	4.8666	4.70616	1.84935	1.98787	2.01415
Zmynd10	-2.84965	5E-05	5.61176	4.36223	4.47726	1.48863	1.75597	2.3269
Adhfe1	-2.85429	0.0059	0.66905	0.87181	0.80434	0.44018	0.44955	0.31204
Scgb3a2	-2.86406	0.00092	208.605	258.734	440.841	126.231	155.061	95.5678
Faim2	-2.86447	0.00011	0.6174	0.96106	1.16351	0.43823	0.48874	0.20876
Tekt1	-2.86575	1.2E-07	4.46253	4.57138	4.40757	2.44243	1.57148	1.67256
Abca4	-2.86977	0.00022	0.31918	0.23051	0.32126	0.11863	0.16962	0.13736
Kcnh3	-2.87128	0.01723	0.92489	0.79433	1.39899	0.30959	0.3557	0.62548
Cep70	-2.87203	4.3E-13	3.41137	3.53757	3.74447	1.6651	1.52039	1.1535
Stk36	-2.87557	0.00031	2.61474	2.52487	2.52762	0.96009	0.97676	1.24603
Cckar	-2.87676	4.1E-05	5.25804	6.87536	8.94934	3.87339	3.36906	1.66581
Gm5148	-2.87751	0.02414	0.33533	0.59518	0.25278	0.24373	0.17424	0.12095
Efhb	-2.87788	0.00141	1.93358	2.67388	1.69843	0.89326	1.08561	0.70922
Acss1	-2.87858	1.7E-07	7.33068	10.106	10.5702	3.55087	4.38409	3.74314
Lhb	-2.88088	0.0405	2.058	1.54654	1.57559	0.70117	0.50127	1.04382
Cfap44	-2.88451	0.00975	12.2191	11.3319	10.5172	4.08616	4.75195	5.23582
Dnah1	-2.88514	0.00237	3.05552	3.00238	2.71953	0.92331	1.23434	1.44782
Elmod1	-2.88552	8.1E-08	1.74469	1.65976	1.79572	0.75599	0.81068	0.61717
Gm867	-2.8867	0.00416	11.2848	11.6547	8.07599	3.89348	4.17518	4.92674
Stpg1	-2.88987	0.00346	0.51399	0.76025	0.64576	0.33727	0.19475	0.30898
Dnah7b	-2.89947	0.00077	2.05579	2.02423	1.84664	0.79708	0.94831	0.73203
Ttll8	-2.90489	0.00717	0.55968	0.56806	0.50339	0.20802	0.29743	0.17204
Pm20d2	-2.90684	0.02698	0.99856	0.98011	0.71689	0.21601	0.27797	0.70745
Nek11	-2.90842	0.00276	1.33502	1.79921	1.35472	0.63446	0.58031	0.63893
Pnma1	-2.90972	0.00286	1.99627	1.84146	1.67203	0.60457	1.18857	0.65001
Tekt2	-2.91109	0.0025	3.5801	3.23238	2.50845	1.45954	1.38627	0.99328
Cfap221	-2.91498	0.00067	1.04709	1.41315	1.21216	0.27181	0.64124	0.64742
1700029J07Rik	-2.919	0.00184	3.3419	3.30748	3.35891	1.01209	1.3644	1.72192
Cfap69	-2.92122	1.3E-07	3.27649	3.14557	2.91063	1.42327	1.5155	0.9848
Srcin1	-2.92328	0.00094	2.93794	2.85231	2.39006	0.8787	1.12895	1.33279
Iqca	-2.92485	3E-06	2.40545	2.64362	2.42025	0.98161	1.07249	1.04773
Hsph1	-2.92535	0.00318	4.33278	6.92686	7.65638	2.34447	2.58426	2.64604
Ccdc146	-2.92741	0.00044	3.87057	3.76968	3.77873	1.57703	1.51619	1.75403
Igfbp6	-2.93199	0.00059	9.25404	10.6418	13.9665	4.56013	4.96647	4.24287
Grm4	-2.93457	0.00444	0.42636	0.38026	0.43265	0.14899	0.2077	0.15526
Dnaic1	-2.93838	0.00407	2.53911	2.18067	2.8356	0.68353	1.16056	1.22955
Ldhb	-2.9392	1.3E-06	23.8293	27.9551	44.07	16.553	12.8259	9.76671
Gm26908	-2.94031	0.00036	2.02437	1.9216	1.70671	0.67761	0.83045	0.79259
Gm1673	-2.94176	0.03894	1.30355	2.12711	1.84245	0.82904	0.63501	0.70524
1700001L19Rik	-2.94192	0.00333	1.59173	1.57416	1.49984	0.43823	0.56393	0.97858
Spag8	-2.94817	0.00767	3.20076	3.51501	2.37323	1.19182	1.3803	1.20649
Syt5	-2.95216	2.8E-05	1.79545	2.8637	2.17517	1.03202	1.03529	0.74339
Armc3	-2.96452	0.00017	1.44832	1.58792	1.85833	0.78392	0.54041	0.66687
RP23-163N19.1	-2.96522	2E-05	0.86543	0.65684	0.83444	0.40229	0.37257	0.16333
Gm13111	-2.96992	2.6E-05	1.25567	1.03612	1.43838	0.40265	0.74362	0.36631
Akap14	-2.97124	0.00197	2.33592	2.5077	3.76016	1.238	1.09051	1.11896
Tmem132d	-2.97829	4.2E-05	0.44404	0.47381	0.82414	0.31786	0.22287	0.1456
Mettl7a1	-2.98178	1.3E-07	72.7579	81.5391	110.844	38.5011	44.4453	26.8101
Pir	-2.98899	3.3E-08	3.12521	2.95379	3.6403	1.25562	1.40767	1.25323
Fam179a	-2.98914	0.00376	7.1019	6.97828	5.66913	2.18368	2.32656	3.27178
Gpr155	-2.99103	8E-08	3.05533	3.9921	5.3273	1.50659	2.11172	1.53643

Chst8	-2.99429	0.00354	0.40302	0.58575	0.67187	0.2535	0.27184	0.12579
Ccdc170	-2.9968	0.00053	5.86444	6.65617	5.93762	2.38198	2.1062	2.8306
P2rx6	-2.99765	0.0138	3.55816	3.29958	3.16763	0.97181	1.04212	1.80102
Rsph14	-3.00133	0.00015	1.18709	0.89518	1.41866	0.55242	0.45134	0.39161
Dcdc5	-3.00421	0.00449	1.72001	1.6607	1.55025	0.39796	0.62451	0.91032
Trp73	-3.00506	2.3E-07	1.63889	1.94888	1.93034	0.73019	0.78302	0.70336
Cfap61	-3.0051	3.2E-05	1.60338	1.5677	1.4101	0.5827	0.57679	0.6784
Kndc1	-3.01466	0.00105	2.6466	2.79053	2.48614	0.91743	0.96794	1.2336
Rgs22	-3.01983	0.00192	8.28229	6.51765	6.44461	2.21319	3.24962	3.01596
Mns1	-3.02332	1E-06	1.78871	2.84895	2.77333	1.3001	1.29911	1.09968
Drc1	-3.02671	1.7E-06	5.55814	5.35973	5.05352	2.09672	2.15342	2.15417
Ccdc30	-3.03258	0.00282	3.40541	3.09472	3.29588	1.09129	1.27312	1.55317
Kcnk12	-3.04113	0.00478	2.56758	2.16643	1.78268	0.49111	0.92162	1.21851
Rilp	-3.04219	3.9E-06	3.41559	3.50117	4.20469	0.95099	2.36163	1.11767
9230110C19Rik	-3.04318	0.00073	0.93124	1.45842	1.65172	0.84607	0.5337	0.14818
9530026P05Rik	-3.04536	1.5E-07	0.86449	0.91179	1.10041	0.49606	0.35464	0.3077
Asb14	-3.05007	0.01106	3.2844	2.46815	2.83689	0.96359	1.23331	1.20312
Ttc6	-3.05341	1.6E-05	1.43562	1.13208	1.0945	0.52173	0.38146	0.49426
Gsg1l	-3.05872	0.01593	0.24453	0.32456	0.37704	0.08079	0.12995	0.1804
Wdr66	-3.06467	5.1E-07	4.31379	4.40627	4.15148	1.60952	1.84926	1.63368
Reep6	-3.06963	4.2E-06	2.26197	4.1244	4.75464	1.89702	1.4918	0.94136
Ttc34	-3.093	0.00442	1.51543	1.92198	1.51096	0.59236	0.68672	0.715
Mapk15	-3.09388	0.02094	10.6924	10.499	10.2356	3.00099	3.54988	5.29655
Fam47e	-3.10261	0.00075	3.38911	4.01105	4.24497	1.65224	1.65098	1.22983
Gm10069	-3.10733	0.04586	0.19956	0.29136	0.31967	0.09065	0.09721	0.10797
Grhl1	-3.10742	2E-08	1.91857	1.88312	2.32433	0.86024	0.75255	0.77511
Casc1	-3.11326	4.4E-05	1.72841	1.8004	1.53994	0.55973	0.65858	0.74067
A830018L16Rik	-3.11579	3.1E-06	0.41785	0.48936	0.47427	0.14668	0.25907	0.14129
Pi16	-3.12643	2.7E-06	2.49352	3.8134	5.15257	1.80841	1.49173	1.06503
Fam92b	-3.1265	0.00781	2.56309	2.73816	2.76856	0.79516	1.52267	0.50732
Aim1l	-3.14064	0.00062	1.84666	1.98516	1.84659	0.69622	0.74659	0.73062
Gpt	-3.14326	3.2E-09	4.33794	3.68274	4.93321	1.67615	2.38038	0.87671
Cyp3a13	-3.15037	0.01663	0.41303	0.48141	0.75057	0.37525	0.14372	0.07981
Sptbn2	-3.15357	1.6E-08	1.45857	1.57037	1.54367	0.7106	0.77231	0.54323
Fbxl13	-3.17007	0.00482	1.19804	0.94318	1.12888	0.2948	0.48635	0.47262
Ccdc180	-3.17185	0.00837	6.85532	5.95833	5.79188	1.84157	2.00692	3.03126
Tsnaxip1	-3.17863	0.00169	3.56349	3.36312	3.32088	1.35197	1.22087	1.32411
Spata17	-3.19187	3.8E-05	1.97509	1.80065	1.48885	0.55214	0.74011	0.73977
Acsl1	-3.1924	4.6E-11	5.49368	6.23809	6.86152	2.53751	2.88275	1.68307
Ttll9	-3.20622	0.00054	1.09915	1.13707	1.08278	0.40853	0.41375	0.43924
Rmdn2	-3.20842	1.1E-07	2.8069	2.74316	4.0585	1.40701	1.36736	0.78547
BC021891	-3.20875	0.00042	0.64337	0.61833	0.46189	0.18093	0.25372	0.22791
Tcp11	-3.21366	3.6E-06	3.79708	3.54774	3.1847	1.0994	1.50416	1.41091
Ccdc108	-3.21674	0.00687	16.2545	14.0078	13.1086	3.67933	4.61943	6.9561
Ttc41	-3.22406	0.00316	1.05115	1.25741	0.83128	0.4434	0.36576	0.38082
Alas1	-3.22461	1.8E-12	33.6263	45.2443	54.6008	18.1455	18.5318	13.0471
Pomgnt2	-3.22563	3.2E-06	1.29174	1.36739	1.21717	0.57316	0.61463	0.24379
Kctd8	-3.22956	0.00038	1.00833	1.24347	1.14502	0.42282	0.40304	0.4546
Klhl34	-3.23902	0.02197	0.63096	0.34836	0.80687	0.32758	0.13173	0.18287
Col23a1	-3.24089	1.6E-11	7.61698	8.14776	10.3003	3.5638	3.43652	2.67325
Rp1	-3.24475	6.1E-06	2.2172	2.07455	2.18265	0.79805	0.9041	0.71858
Colq	-3.25772	0.00058	1.03191	0.92562	0.97234	0.37501	0.22342	0.49625
9030622O22Rik	-3.27294	0.00248	3.11085	2.9834	2.54043	1.03633	0.90926	1.26227
Atp6v1c2	-3.29079	2.6E-06	6.48791	5.57202	7.34398	2.29659	3.16053	1.70945
Elf5	-3.29214	1.3E-06	1.09807	1.29989	1.53804	0.56623	0.44817	0.42147
Inmt	-3.29218	0.00037	46.438	61.3685	104.052	27.2856	36.2163	14.4491

Tmie	-3.29784	1.2E-05	1.15563	1.47663	1.24827	0.40973	0.57668	0.41935
Ppil6	-3.29849	0.00097	6.78862	6.34283	4.85026	2.16889	2.61654	1.9171
Lgals12	-3.29986	0.00093	0.69187	0.70224	0.91863	0.28573	0.33704	0.21268
Bnipl	-3.30393	0.01098	0.64542	0.71734	1.32364	0.31044	0.51785	0.1027
Gm26549	-3.30425	0.02606	1.51477	1.54873	1.22342	0.39321	0.70277	0.48781
Kif27	-3.31094	2.6E-05	4.67114	4.2635	4.53039	1.50936	1.67569	1.85043
Six1	-3.31387	0.00325	1.41267	1.2816	1.21863	0.3977	0.46524	0.56514
5330413P13Rik	-3.31728	0.00019	0.50736	0.74697	0.57368	0.21072	0.25422	0.17646
Ak9	-3.33511	0.0001	4.35537	4.11674	3.23127	1.20531	1.34129	1.65891
Myb	-3.33835	1.6E-06	4.43846	4.81781	4.5114	1.86692	1.72172	1.44522
Cfap157	-3.34048	0.00775	4.85304	4.21004	4.20335	1.24705	1.194	2.25428
Slurp1	-3.35606	0.03652	0.47573	0.33197	0.3362	0.1891	0.20278	0
Cux2	-3.36292	7E-07	0.86498	0.88402	1.00251	0.33793	0.28653	0.36268
Cfap46	-3.36405	0.00148	7.10027	5.75691	5.88861	1.69219	1.86989	2.94112
Esyt3	-3.36481	5.7E-07	2.12061	2.11135	2.12197	0.73316	0.96904	0.58379
Myh7	-3.36736	4.4E-06	1.05632	0.98571	1.11019	0.38758	0.39583	0.35718
Igfbp3	-3.37373	0.00017	1.42095	1.89036	3.20292	0.68347	0.76007	0.82905
Ccdc57	-3.38459	6.6E-05	3.51813	2.70132	2.5686	0.80439	0.97609	1.35504
Prr36	-3.38874	0.00273	0.7667	0.94942	0.65524	0.35639	0.50378	0.36174
Ccdc39	-3.38943	4E-05	5.84665	6.19862	6.07219	2.2142	2.00911	2.26617
Daw1	-3.39677	0.00712	1.34479	1.08495	1.45915	0.2962	0.59555	0.49606
Cadm2	-3.39914	0.00863	0.17781	0.13744	0.20943	0.04847	0.05197	0.0962
Lpar3	-3.40328	3E-08	4.24712	4.65065	5.10248	1.54349	1.99999	1.4361
Kcnk2	-3.40872	4.4E-06	4.26952	4.43956	5.93483	2.13188	2.13143	0.97836
Dnah2	-3.43254	0.00065	3.58316	3.82289	3.06338	0.8099	1.14096	1.61546
Calcoco2	-3.4429	0.04191	0.53799	0.60065	0.40554	0.09776	0.26208	0.14553
Ect2l	-3.44443	1.4E-06	1.84371	1.88253	1.40913	0.53975	0.73848	0.55415
Entpd3	-3.45441	0.00098	0.55739	0.45875	0.56898	0.1899	0.20364	0.15706
Hepacam	-3.45673	4.6E-05	0.75561	0.7832	0.78564	0.18938	0.3554	0.28193
Cfap99	-3.46068	0.00016	1.97172	2.152	1.8966	0.63446	0.60032	0.83339
Fam161a	-3.46149	0.00014	9.54205	9.67728	8.76689	2.45123	3.01513	4.05158
Rnf186	-3.47316	4.7E-06	4.11949	4.85719	5.85542	2.09341	1.70066	1.32212
Ccdc187	-3.47738	0.00029	8.94528	9.16107	8.60448	2.39463	2.80997	3.7929
Ccdc151	-3.4857	1E-05	2.80979	3.00898	2.77833	1.16611	1.08149	0.75069
Cfap161	-3.50081	1.5E-07	2.02427	2.25536	1.98474	0.77045	0.66629	0.73997
Acot1	-3.50901	0.00034	3.91567	5.33261	6.74674	1.37221	1.85299	2.42108
T2	-3.52008	0.01911	3.84305	2.24267	1.94987	1.07434	0.81605	0.79967
Mgat3	-3.52722	4.7E-07	7.58977	9.16742	8.96595	2.41179	3.47873	2.85714
Fhad1	-3.5331	0.00109	11.0909	9.58619	8.88504	2.41271	2.83631	4.55212
Cfap57	-3.53895	1.7E-09	2.99369	2.99959	2.90264	1.00519	1.26813	0.79221
Synm	-3.5575	2E-08	6.35396	8.29878	8.62855	3.74538	4.31626	3.39002
Odf3b	-3.57306	0.00767	16.1993	14.1287	12.496	3.12659	4.41589	6.58442
Hmcn2	-3.58853	4.2E-07	0.9278	1.02049	0.95604	0.28591	0.35417	0.33757
9330159F19Rik	-3.58981	0.03835	0.31756	0.2987	0.25648	0.09617	0.14733	0
Tex26	-3.59058	0.01066	1.22471	1.1969	0.83566	0.42206	0.28802	0.39984
Pgbd1	-3.61421	0.00115	0.38921	0.39694	0.41259	0.13261	0.1659	0.0658
Gsta3	-3.62016	2.5E-11	20.3587	22.6638	34.0931	9.22504	9.84227	7.11057
Foxo6	-3.62308	0.00112	0.66698	0.68739	0.50278	0.21209	0.22744	0.18042
Slc7a10	-3.64187	8.1E-08	1.98293	2.12875	2.49128	0.78819	0.72448	0.50287
Ccdc13	-3.64852	0.0069	2.27077	2.12551	2.13968	0.32453	0.67117	1.10429
Slc6a15	-3.64966	8.3E-06	0.67028	0.9046	0.90226	0.43498	0.25655	0.09713
Gm16136	-3.65595	6.2E-05	3.06648	3.01899	2.67803	1.22314	1.23877	0.40464
Ankef1	-3.65744	0.00063	0.52191	0.45866	0.33167	0.07719	0.18921	0.18058
Aldh3a1	-3.66538	0.00011	2.2004	3.81811	5.33153	1.01998	1.22503	1.39696
Gria1	-3.6806	0.00175	0.8803	1.0163	1.53658	0.3409	0.47804	0.29278
Gdpd2	-3.6831	0.00092	1.21095	1.72251	1.44414	0.46415	0.4793	0.48624

Chad	-3.68466	0.00128	5.29475	5.88474	7.23907	1.41486	2.32642	2.10628
Fn3k	-3.68847	0.00169	0.23789	0.28944	0.35492	0.07205	0.15452	0.05363
Zbtb16	-3.70342	0.00204	19.462	22.7398	26.2867	2.44461	8.04135	10.4266
5330417C22Rik	-3.70982	2.9E-10	8.88123	11.263	13.374	4.32359	3.98947	2.52594
Tbx1	-3.74325	0.00112	5.49648	5.35277	5.50288	1.42681	1.57785	2.19044
Hs3st6	-3.76164	0.002	1.57389	1.59747	0.94375	0.38999	0.5576	0.38704
Gm44276	-3.7617	0.00441	0.22044	0.20195	0.19387	0.08011	0.07159	0.03975
Gm16194	-3.76867	0.03089	1.42768	1.32832	0.78474	0.21619	0.34774	0.64367
Crocc2	-3.7701	0.03548	4.54869	4.45264	3.06479	0.78877	1.13175	1.81922
Pclo	-3.77769	0.00021	0.7223	0.52361	0.83794	0.2505	0.21566	0.17858
Ccdc17	-3.77913	0.00171	31.1508	26.1574	19.0636	6.50315	7.18715	9.73053
Ccdc150	-3.79229	0.02848	1.28527	0.88008	0.70373	0.22092	0.27075	0.42285
Gm26910	-3.79679	0.01497	0.87457	1.93143	1.7659	0.56757	0.24345	0.67594
Dnah10	-3.79873	0.0011	9.55781	8.5531	7.26921	1.82245	2.22897	3.68829
Ccdc24	-3.79891	5.7E-05	2.42729	2.28476	2.10419	0.79391	1.02162	0.70913
Cfap74	-3.82887	0.00027	4.05759	3.21384	3.24531	0.79622	1.16975	1.35127
Cldn8	-3.85533	0.00306	0.55523	0.60401	1.01147	0.26904	0.32457	0.05006
Vpreb3	-3.8845	1.6E-07	6.90351	9.59096	12.436	2.21371	2.86843	3.84476
Zfp354b	-3.88522	0.02696	0.51334	0.54461	0.51216	0.13718	0.1471	0.16337
Plk5	-3.9023	0.00405	0.5536	0.97886	0.58301	0.14793	0.22209	0.30831
Ccna1	-3.90623	0.00419	0.66534	0.5306	0.7165	0.34543	0.13891	0.06428
Wdr93	-3.91108	4.1E-06	1.56478	1.96665	1.36656	0.34675	0.59494	0.61944
Dner	-3.93277	0.00389	0.27344	0.43836	0.36071	0.08281	0.10656	0.14793
Ace2	-3.94323	1.3E-05	2.38766	2.94649	3.18907	1.09518	0.99374	0.53301
Dnaaf1	-3.9444	3E-07	2.81315	2.97357	2.47597	0.8426	0.87344	0.91987
Mlxipl	-3.96921	0.00016	0.42517	0.4463	0.48966	0.14307	0.09205	0.19169
Abhd12b	-3.98872	0.00599	0.33192	0.45611	0.25021	0.14073	0.1078	0.05986
Atp7b	-4.00495	2.1E-12	6.75675	6.48222	7.45307	2.16808	2.37719	1.72259
Ces1d	-4.01351	1.6E-07	55.4389	65.8863	93.4465	26.3216	25.9306	12.9951
Ccdc155	-4.07068	0.00824	1.80684	1.96347	1.32033	0.20101	0.46704	0.84786
Pon1	-4.09907	3.7E-06	5.44671	5.60821	8.44983	2.35127	2.31616	1.09893
Noxa1	-4.13518	0.04709	0.97077	0.46318	0.66064	0.392	0.05254	0.14589
Fam13a	-4.1415	2.6E-12	6.03618	6.36764	6.98628	2.10351	2.22885	1.23022
Krt79	-4.16294	0.00101	0.87332	0.71432	1.05802	0.11335	0.2431	0.44998
Six4	-4.16996	7.5E-14	2.0165	1.81463	1.78654	0.61313	0.65749	0.38031
Tas1r1	-4.20487	0.00211	0.67012	0.70633	0.68884	0.16605	0.11871	0.32959
Stk33	-4.20803	2.6E-11	2.69443	3.21711	3.62256	0.74635	1.21653	0.79997
Ttc16	-4.21123	8.7E-06	2.89418	2.79045	2.68851	0.70699	0.77144	0.90477
Cfap73	-4.22169	0.01842	4.16627	4.71662	3.39043	0.68823	0.73802	2.04911
Lrrc43	-4.22308	0.01266	6.37384	5.58281	4.77289	1.22722	1.02813	2.34077
Bmpr1b	-4.25748	1.1E-10	1.40422	1.46979	1.29559	0.46513	0.42753	0.29676
Lrrc23	-4.26643	0.0007	24.8446	22.6331	21.0883	5.20904	4.89994	8.63892
Ooep	-4.27502	0.00132	0.65263	0.52547	0.61495	0.29647	0.11922	0.05517
Mycl	-4.2977	3.8E-10	4.18977	3.93217	4.1514	1.02033	1.36769	1.08079
Cbs	-4.35208	5.1E-08	1.06955	1.14261	1.32736	0.45032	0.3304	0.17642
Ces1g	-4.36536	0.0022	0.47475	1.41137	2.23671	0.59723	0.39138	0.09879
Scn5a	-4.39579	7.1E-06	2.90559	2.46978	2.46595	0.49724	0.71741	0.90173
Nr1i2	-4.39809	0.00678	0.54654	0.38138	0.35406	0.15517	0.13312	0.0462
Trim66	-4.42192	0.00014	0.17226	0.15534	0.17043	0.0313	0.05035	0.05825
Mettl7a3	-4.43847	0.00097	0.75458	0.97208	1.52361	0.29382	0.36759	0.2187
Tymp	-4.44634	0.00599	8.60517	6.65503	6.29309	0.93352	1.9187	3.18476
9130230L23Rik	-4.463	5.1E-07	1.96994	1.79596	1.85622	0.42679	0.63484	0.4714
Foxn4	-4.46867	0.01128	0.2587	0.31105	0.34127	0.07051	0.10082	0.06998
Itpka	-4.48165	3.2E-11	2.7822	3.37818	3.61598	0.91522	0.88797	0.84344
Plch2	-4.58903	0.00176	1.60182	1.51418	1.38779	0.24418	0.42943	0.50891
Ccdc89	-4.63366	0.00493	0.54166	0.39363	0.23557	0.18928	0.08119	0

Bbof1	-4.63714	8.9E-07	7.3779	7.04042	6.54737	1.52506	1.6354	2.37593
Fhdc1	-4.67079	7.2E-11	7.49679	8.52891	8.3877	2.28725	2.42563	1.65546
Cyp4f15	-4.6939	5.4E-10	2.10185	2.16616	2.59267	0.45137	0.67019	0.67194
Cbr2	-4.70454	2.2E-08	626.751	706.298	1178.17	240.742	234.214	151.373
Cecr6	-4.74959	1.1E-05	0.39404	0.49224	0.27003	0.09764	0.12215	0.07268
B430010I23Rik	-4.75233	0.0002	14.1079	13.5822	15.0274	4.55525	3.53371	2.34461
Klf15	-4.78093	4E-05	4.90562	5.74271	8.83266	0.9936	1.88743	1.85951
Cyp2f2	-4.78469	1.1E-06	341.294	431.664	701.171	162.651	126.294	75.6639
Tmem25	-4.79049	0.00028	0.43441	0.46636	0.43491	0.12334	0.15871	0.03672
Mcidas	-4.9411	0.00573	0.40751	0.50907	0.55853	0.10098	0.07219	0.20043
Ttll6	-4.98661	2E-05	4.31855	4.05844	4.21838	0.69844	1.01332	1.28441
Lrrc26	-5.08607	6.9E-06	3.88404	6.67147	7.803	1.26505	1.42798	1.58591
Gm12474	-5.10376	0.00078	1.97918	1.27484	0.99074	0.44954	0.30129	0.25096
Wdr27	-5.13559	0.00011	1.52461	1.19299	1.08543	0.18469	0.3631	0.36659
Ccl28	-5.14292	0.00032	0.32688	0.42111	0.17601	0.08485	0.09099	0.03158
Panx2	-5.2023	0.01421	0.19385	0.18729	0.20549	0.06605	0.02361	0.03277
Hhatl	-5.22372	0.00015	0.73088	0.65624	0.59478	0.06037	0.19421	0.22467
2010001K21Rik	-5.23522	0.02469	1.84833	2.59757	1.89996	0.45799	0.49113	0.45454
Aox3	-5.25528	1.5E-16	11.1598	12.5769	15.3761	3.26708	3.43097	2.29765
Fmo3	-5.29073	2E-08	8.16781	10.2118	15.3926	2.42983	2.85212	2.24856
Lypd2	-5.31728	1.2E-08	9.76109	10.0377	15.2486	1.75035	3.37858	2.77943
C330002G04Rik	-5.32947	1.9E-07	1.18881	1.45855	1.2402	0.23145	0.37229	0.28713
Cntd1	-5.34107	0.01889	0.19726	0.35576	0.39033	0.21506	0.11531	0.16008
Gm20755	-5.34603	0.0085	0.2864	0.49193	0.5847	0.17347	0	0.12912
Slc4a5	-5.42386	1.3E-13	5.60449	5.76823	7.07902	1.61086	1.41999	1.07709
Gm6970	-5.43855	0.01124	1.17848	1.46195	0.49353	0.23793	0.25515	0.1771
E030044B06Rik	-5.44359	0.0158	0.48219	0.5916	0.58417	0.06258	0.26845	0
Bcan	-5.44407	0.00081	0.40771	0.53445	0.46506	0.13647	0.08363	0.08707
Cnksr2	-5.55179	5E-14	0.56463	0.64657	0.91333	0.16245	0.21088	0.07637
Ces1f	-5.55618	5E-06	0.47785	0.84359	1.2007	0.24119	0.20692	0.07181
A430108G06Rik	-5.57666	3.8E-05	1.62355	1.94213	0.87418	0.26341	0.33896	0.39213
Gabre	-5.58273	1.9E-12	2.06234	2.00842	2.09944	0.38479	0.46645	0.49811
Atp2b2	-5.59184	4.2E-13	1.94302	1.76968	2.29996	0.47117	0.47158	0.35539
Acaa1b	-5.63812	0.00048	0.62228	0.41991	0.75388	0.28268	0.17322	0
Ttll13	-5.63949	0.00013	0.31295	0.34044	0.27522	0.03791	0.08131	0.08465
Slc16a11	-6.09944	6.9E-08	15.3655	16.8327	14.8645	2.57489	2.99406	3.60229
D930020B18Rik	-6.13332	1.1E-05	0.77175	0.72044	0.66396	0.18291	0.06538	0.18153
Dcdc2a	-6.47085	5.4E-13	0.91587	1.17535	0.92251	0.21998	0.12308	0.22782
Crb1	-6.50811	7.6E-06	0.09572	0.18496	0.21797	0.03624	0.03109	0.02158
Ccdc129	-6.76271	7.8E-11	2.52705	2.60855	2.79329	0.59606	0.52082	0.29578
Fam205c	-6.78452	0.01098	0.4661	0.36027	0.57095	0.0847	0.13623	0
4930547M16Rik	-6.80607	0.00494	0.25436	0.31207	0.34239	0.06603	0	0.09829
Cpne5	-6.81767	0.00239	0.03634	0.08115	0.08904	0.05943	0.00708	0.02949
Ces1e	-7.31084	1.6E-11	1.99195	2.53191	3.15329	0.36196	0.42696	0.48496
AI593442	-7.36382	0.00028	0.13479	0.2058	0.25401	0.04082	0.01459	0.04051
Bex2	-7.43035	5.7E-05	1.35206	1.32707	1.365	0.43872	0.09409	0
Cidec	-7.48343	0.01403	0.37589	0.60531	0.92976	0.04269	0.18311	0.06355
1700095J03Rik	-7.59899	0.00603	0.57881	0.62138	0.6363	0.13147	0.14098	0.06524
Cyp2g1	-7.62237	0.02157	0.16262	0.27933	0.19155	0.07388	0	0
Asgr1	-7.63629	0.00055	0.60294	0.28245	0.67143	0.0498	0.10681	0.07414
Gm14062	-7.90793	0.00555	0.12657	0.19929	0.13914	0.01917	0.02055	0.02853
Mfsd4b5	-8.12503	0.01181	0.14469	0.11184	0.09544	0.03944	0.07049	0.03914
Gm2568	-8.60391	0.01345	0.52354	0.44964	0.91617	0	0	0.30348
Dpy19l2	-8.62496	0.00775	0.20269	0.1088	0.11937	0	0.04937	0
Iqcd	-8.88702	0.002	3.21838	2.45197	2.88585	0.80176	0.60689	1.26377
Cyp2b19	-8.9259	5.6E-06	0.2301	0.34134	0.25624	0.07602	0.02038	0

Gfy	-9.83721	0.0078	0.28299	0.28355	0.35554	0	0.04595	0.06379
Micalcl	-10.5048	0.01779	0.27416	0.36389	0.68106	0.13587	0	0
Gm16124	-11.4775	0.03716	0.67603	1.01604	0.637	0	0	0.22859
Baiap2l2	-11.6995	0.00123	0.22377	0.21964	0.30122	0.02904	0	0.04324
Chrna9	-12.1535	0.0009	0.20174	0.22276	0.27156	0.02618	0	0.03898
Atp6v1b1	-12.4553	5.7E-11	0.6768	0.68383	1.06913	0.01809	0.13576	0.08077
Gm15889	-16.2609	0.00474	1.04681	2.00554	1.66926	0.3658	0.47072	0.32674
Gm5930	-19.8138	0.00174	0.64931	0.21448	0.65889	0.09076	0	0
Plin1	-22.5008	0.0228	0.01876	0.25776	0.24746	0	0	0.02537
Gm11771	-48.5818	0.01059	0.23827	0.61391	1.12259	0	0	0
Ncam2	-52.2368	0.00257	0.05661	0.04862	0.12002	0	0	0
